# Increased frequency of single base substitutions in a population of transcripts expressed in cancer cells

**DOI:** 10.1186/1471-2407-12-509

**Published:** 2012-11-08

**Authors:** Laurent Bianchetti, David Kieffer, Rémi Féderkeil, Olivier Poch

**Affiliations:** 1 Plate-forme Bioinformatique de Strasbourg (BIPS), Institut de Génétique et de Biologie Moléculaire et Cellulaire (CNRS/INSERM/ULP), BP 163, Illkirch, Cedex, 67404, France; 2 Laboratoire de Bioinformatique et Génomique Intégratives (LBGI), Institut de Génétique et de Biologie Moléculaire et Cellulaire (CNRS/INSERM/ULP), BP 163, Illkirch, Cedex, 67404, France; 3 IRMA-UdS, Equipe Statistique, 7 rue René Descartes, Strasbourg, Cedex, 67084, France

**Keywords:** Cancer, Bioinformatics, Transcripts, Substitutions, ESC, Biomarker, Long-SAGE, Tag-seq, Patient, Genetic integrity

## Abstract

**Background:**

Single Base Substitutions (SBS) that alter transcripts expressed in cancer originate from somatic mutations. However, recent studies report SBS in transcripts that are not supported by the genomic DNA of tumor cells.

**Methods:**

We used sequence based whole genome expression profiling, namely Long-SAGE (L-SAGE) and Tag-seq (a combination of L-SAGE and deep sequencing), and computational methods to identify transcripts with greater SBS frequencies in cancer. Millions of tags produced by 40 healthy and 47 cancer L-SAGE experiments were compared to 1,959 Reference Tags (RT), i.e. tags matching the human genome exactly once. Similarly, tens of millions of tags produced by 7 healthy and 8 cancer Tag-seq experiments were compared to 8,572 RT. For each transcript, SBS frequencies in healthy and cancer cells were statistically tested for equality.

**Results:**

In the L-SAGE and Tag-seq experiments, 372 and 4,289 transcripts respectively, showed greater SBS frequencies in cancer. Increased SBS frequencies could not be attributed to known Single Nucleotide Polymorphisms (SNP), catalogued somatic mutations or RNA-editing enzymes. Hypothesizing that Single Tags (ST), i.e. tags sequenced only once, were indicators of SBS, we observed that ST proportions were heterogeneously distributed across Embryonic Stem Cells (ESC), healthy differentiated and cancer cells. ESC had the lowest ST proportions, whereas cancer cells had the greatest. Finally, in a series of experiments carried out on a single patient at 1 healthy and 3 consecutive tumor stages, we could show that SBS frequencies increased during cancer progression.

**Conclusion:**

If the mechanisms generating the base substitutions could be known, increased SBS frequency in transcripts would be a new useful biomarker of cancer. With the reduction of sequencing cost, sequence based whole genome expression profiling could be used to characterize increased SBS frequency in patient’s tumor and aid diagnostic.

## Background

In mammalian cells, genetic integrity is maintained by stability genes [1] which are in charge of correct chromosomal segregation and recombination; damaged DNA repair; accurate genomic DNA replication and transcriptional fidelity. In healthy cells, base substitutions occur at an extremely low incidence during both DNA replication [2] and RNA synthesis [3]. Single base variations can be the result of Single Nucleotide Polymorphisms (SNP) [4] or RNA-editing carried out by ADAR [5] or APOBEC [6] enzymes. By contrast, genetic instability is a hallmark of cancer [7,8]. Major mutational events such as chromosome region translocations, deletions and gene copy number variations have been reported in almost all cancer cells [9]. Somatic mutations, i.e. acquired or inherited SBS which differentially alter cancer cell genomes and consequently transcript sequences, were reported on a genome wide scale using deep sequencing [10,11]. Now, a census of cancer related somatic mutations that alter 422 human genes has been made available [12]. A growing body of cancer studies reports SBS in transcripts that are not supported by the genome of tumor cells. Using EST alignments on reference mRNA sequences, Brulliard M. et al. proved that 15 abundantly expressed transcripts, namely GAPDH, VIM, FTH1, ENO1, HSPA8, TPT1, RPS4X, ATP5A1, FTL, RPL7A, TPI1, RPS6, ALDOA, LDHA and CALM2 had statistically greater SBS frequencies in cancer than in healthy cells whereas ALB and TSMB4X showed the opposite [13]. Since most EST are 3’ fragments of mRNA sequences, increased SBS in cancer was detected at the 3’ boundary of mRNA. These SBS could not be explained by known SNP and were also unlikely the result of somatic mutations or RNA-editing enzymes. The possibility that instruments generated more sequencing errors when EST originated from cancer cells does not seem rational. As a working hypothesis, the concept of transcriptional infidelity (TI) was proposed: i) TI introduces non-random base variations in RNA sequences that are not supported by the genome ii) TI exists in both healthy and cancer cells, but is greater in cancer. Increased TI in cancer has been speculated to originate from a defective proofreading activity of RNA polymerases. Recently, in a study carried out at both genomic and transcriptional levels, SRP9 and COG3 mRNA expressed in tumor cells were clearly shown to carry SBS that were conflicting with the genome sequence [14]. SRP9 sequencing chromatogram traces showed an adenine (in tumor DNA) and a guanine (in tumor RNA) substitution which might be attributed to ADAR, in fact ADAR carries out adenosine to inosine editions and inosine is read as guanosine by sequencing instruments. Intriguingly, in the case of COG3, a thymine (in tumor DNA) was replaced by a cytidine (in tumor RNA) which cannot be carried out by ADAR nor APOBEC enzymes because APOBEC converts cytosine to uracile in RNA sequences. Personalized omics profiling also concurred on the fact that RNA-editing is extensively carried out in peripheral blood mononuclear cells with more than 2300 target sites and approximately 50% of them were not typical ADAR or APOBEC edits [15]. Deregulation of RNA-editing, e.g. adenosine to inosine hypoediting, was also reported in tumors [16]. To identify mRNA with greater SBS frequencies in cancer, we performed a bioinformatics analysis of 7.6 million tags produced by 87 human L-SAGE [17] experiments (a molecular biology method using Sanger sequencing), and 67.8 million tags generated by 15 human Tag-seq experiments (a combination of L-SAGE and deep sequencing) [18,19]. Both L-SAGE and Tag-seq generate short sequences that are likely localized on the 3’ boundary of transcripts. Therefore, L-SAGE and Tag-seq may prove useful to detect SBS introduced in the 3’ boundary of transcripts. Briefly, tags are short sequences of 17 bases which are signatures of 3' polyadenylated transcripts expressed in cells. The most 3’ *NlaIII* “CATG” motif in the transcript sequence is directly followed by the 17 base tag. Moreover, tag counts and mRNA expression levels are correlated. Comparing tags to RT sequences, i.e. tags matching the human genome exactly once, we showed that a plethora of transcripts had greater SBS frequencies in cancer cells. Although the genomic sequences of the tumor and the healthy cells were not simultaneously available in our study, these SBS could not be attributed to known SNP, catalogued cancer related somatic mutations, and known APOBEC1 or ADAR editing. ST proportions, i.e. proportions of tags sequenced only once were calculated for each experiment and were used as an indicator of SBS frequency. Interestingly, among healthy cells, ESC had the lowest ST proportions which might indicate that transcriptional fidelity could be increased in ESC. Conversely, the greatest ST proportions were observed in cancer cells. Finally, focusing on a series of 4 L-SAGE experiments carried out on the biopsies of a single patient at 1 healthy and 3 consecutive tumor stages, we were able to demonstrate that SBS frequencies significantly increased during cancer progression.

## Methods

### L-SAGE and Tag-seq experiments

The GPL1485 platform of the NCBI Gene Expression Omnibus (GEO) server is a repository of L-SAGE and Tag-seq experiments carried out on human cells. In the GPL1485, the GSE1902 (L-SAGE) and GSE15314 (Tag-seq) series of experiments were selected. All experiments were carried out using the *NlaIII* anchoring enzyme which cuts 3’ polyadenylated transcripts at CATG sites. Experiments were separated into 2 groups, namely healthy and cancer using a dictionary of cancer related terms: adenocarcinoma, cancer, carcinoma, dysplasia, fibroadenoma, glioblastoma, leukemia, lymphoma, medulloblastoma, melanoma, tumor, retinoblastoma and rhabdomyosarcoma. The Sybase system was used to store the tags of L-SAGE and Tag-seq experiments. Programs were run on a 6 × 4 Sun AMD Opteron processors (2.6 GHz) under the linux operating system.

### Reference tags (RT)

RT were selected among the tags produced by the L-SAGE and Tag-seq experiments. Tags should fulfill 2 criteria to be selected i) presence in at least 75% of L-SAGE or 90% of Tag-seq experiments ii) exactly one match on the human genome sequence. Tags that fulfilled the first criteria were selected using a JAVA program and were subsequently aligned on the human genome using a blastn tool. Two distinct lists of RT were thus created, 1 for the L-SAGE and 1 for the Tag-seq experiments.

### Single base substituted RT (sbsRT)

For each RT, and for each of the 17 base positions, a nucleotide was replaced by a "_" metacharacter. Thus, 17 distinct patterns were generated (Additional file 1). A Java program was written to automatically i) generate the 17 distinct patterns ii) retrieve from the database of L-SAGE and Tag-seq experiments all the tags that matched the patterns and iii) sum the tag counts. The risk that a sbsRT could match by chance a RT was calculated (Additional file 2) and equaled 6.5 × 10^-5^. Thus, any tag that was identical to a RT except at 1 base position, was very likely the result of a SBS that had occurred in this RT.

### Testing for SBS frequency equality in transcripts expressed in healthy and cancer cells

Let C and H be the number of cancer and healthy L-SAGE experiments (or Tag-seq experiments). For each RT, 4 sum of counts (Sc) were calculated (i, ii, iii and iv):

i. Sum of counts of the RT across all healthy experiments Sc _ H _ RT = ∑ _*k* = 1_^*H*^RT count in exp. *k*

ii. Sum of counts of the RT across all cancer experiments Sc _ C _ RT = ∑ _*k* = 1_^C^RT count in exp. *k*

iii. Sum of counts of sbsRT (associated with the RT) across all healthy experiments Sc _ H _ sbsRT = ∑ _*k* = 1_^*H*^ ∑ _*i* = 1_^51^sbsRT*i* count

iv. Sum of counts of sbsRT (associated with the RT) across all cancer experiments Sc _ H _ sbsRT = ∑ _*k* = 1_^*H*^ ∑ _*i* = 1_^51^sbsRT*i*count

Then, for each RT, 2 sbsRT proportions were calculated (i, ii):

i) sbsRT proportion across all healthy experiments $\text{sbsRT}\_\text{prop}\_\text{H}=\frac{\text{Sc}\_\text{H}\_\text{sbsRT}}{\text{Sc}\_\text{H}\_\text{RT}+\text{Sc}\_\text{H}\_\text{sbsRT}}$

ii) sbsRT proportion across all cancer experiments $\text{sbsRT}\_\text{prop}\_\text{C}=\frac{\text{Sc}\_\text{C}\_\text{sbsRT}}{\text{Sc}\_\text{C}\_\text{RT}+\text{Sc}\_\text{C}\_\text{sbsRT}}$

Finally, for each RT, two 1-side Pearson’s chi-squared proportion tests (i, ii) were carried out using a 0.025 α type I error

i) Pearson’s chi-squared proportion test (Cancer > Healthy):

H_0_ : "sbsRT_prop_C *equals* sbsRT_prop_H"

H_1_: "sbsRT_prop_C *is greater than* sbsRT_prop_H"

ii) Pearson’s chi-squared proportion test (Healthy > Cancer)

H_0_ : "sbsRT_prop_C *equals* sbsRT prop_H"

H_1_: "sbsRT_prop_H *is greater than* sbsRT_prop_C"

A script was written in the R environment to carry out the Pearson’s chi-squared proportion tests. For a RT, and thus a transcript, the H_o_ hypothesis was rejected when a p-value less than 0.025 was obtained. Three lists of RT were thus produced according to the decision of the Pearson’s chi-squared proportion test i) RT for which proportions of sbsRT were greater in cancer than in healthy, ii) RT for which proportions of sbsRT were greater in healthy than in cancer iii) RT for which proportions of sbsRT in cancer and healthy were not significantly different.

### Global proportions of sbsRT

Global proportions were calculated for selected experiments. Across a set of experiments (*e*. *g*. same healthy tissue), RT that were present in 100% of the experiments were selected. Let *N* be the number of RT that were present in all experiments. The sum of RT counts and the sum of their associated sbsRT counts were calculated. Finally a global proportion of sbsRT across the set of experiments was computed as follows:

$$global\;sbsRT\;proportion=\frac{\sum_{1}^{N}sbsRT\;counts}{\sum_{1}^{N}RT\;counts+\sum_{1}^{N}sbsRT\;counts}$$

Global sbsRT proportions were tested for equality across different healthy tissues using the Analysis of Variance (Anova).

### Single tags (ST)

ST are tags that were sequenced only 1 in a L-SAGE experiment, i.e. ST were associated with a count of 1. For each L-SAGE experiment, a list of ST could thus be defined and the proportion of ST on total tags could be calculated. ST was not reported in Tag-seq experiments. In fact, counts were greater than 1 which showed that ST had been discarded from Tag-seq experiments.

### ST proportions

For each L-SAGE experiment, the proportion of ST was calculated:

$$ST\;proportion=\frac{n}{total\_tags}$$

where *n* is the number of ST and total_tags is the sum of counts.

### Known SNP that altered 17 base *NlaIII* tags of transcripts

A file of 17 base *NlaIII* tags associated with known SNP was provided by Dr. Anamaria Camargo. In this file, each line recorded a Genbank mRNA accession number, the *NlaIII* 17 base tag associated with the mRNA and the sequence of the tag with the known SNP. The file contained 4,697 entries. It was thus possible to identify sbsRT that were the result of known SNP.

### Census of genes with cancer related somatic mutations

A census of somatically mutated genes in cancer was downloaded from the COSMIC database (v56). Known somatic mutations were recorded for 422 distinct genes which were identified by NCBI Gene ID. In our study, transcripts were identified with Genbank or RefSeq ID and thus were converted to NCBI gene ID using the Synergizer tool [20]. Area proportional Venn diagrams were drawn to determine whether known somatically mutated genes were present among the genes with greater SBS frequencies. Bases that were somatically mutated in cancer and recorded by COSMIC were localized on transcript sequences and their proximity or inclusion to the 17 base *NlaIII* tag was determined.

### Validated and predicted APOBEC1 and ADAR RNA-editing targets

APOBEC1 RNA-editing targets. A series of 32 editing sites in 30 distinct transcripts are known substrates for the Apoliprotein B-editing enzyme, catalytic polypeptide-1 (APOBEC1) in mouse. Using an APOBEC1 specific editing sequence pattern, namely WCWN_2-4_WRAUYANUAU (mooring sequence), which is located directly 3’ to the edited cytosine, Rosenberg B. R. et al. predicted 376 editing sites in 363 distinct mouse transcripts. Out of these 363 transcripts, ten were previously experimentally validated, in particular, the prototypic ApoB editing site. Thus 383 distinct mouse transcripts either predicted or validated APOBEC1 RNA-editing targets are available. However, our study was carried out on human sequences. Therefore, conservation of RNA-editing targets between human and mouse organisms was hypothesized. Human orthologues of mouse RNA-editing targets were retrieved from RefSeq by sequence similarity searches using blastn. Top scoring human transcripts were assumed to be orthologues of mouse transcripts targeted by the APOBEC1. RefSeq ID were then converted to NCBI gene ID with the Synergizer tool. A list of 361 unique NCBI gene ID was thus produced for the human transcripts. Venn diagrams were drawn to identify human transcripts which could be APOBEC1 RNA-editing targets and showing greater SBS frequencies in cancer or healthy cells. These transcripts were compared with the mouse orthologues to determine the local level of similarity between mouse and human mooring sequences. Pairwise sequence comparison was carried out using the Smith and Waterman local algorithm implemented in the water program of the EMBOSS package (gap opening penalty 10, gap extension penalty 0.5, EDNAFULL matrix). When the mooring sequences were conserved between mouse and human, the 17 base *NlaIII* tag was localized on the human transcript. Finally, proximity between the 17 base *NlaIII* tag and the mooring sequence was determined and the possibility that the 17 base *NlaIII* tag could be edited by the APOBEC1 enzyme was assessed.

ADAR RNA-editing targets. Most A-to-I susbstitutions occur within interspersed repetitive elements mainly in *Alu* sequences. Since RT match the human genome exactly once, they are very unlikely located in *Alu* repeats. Therefore, sbsRT may not be the result of ADAR RNA-editing.

## Results

### Groups of healthy and cancer experiments

**Figure 1:**
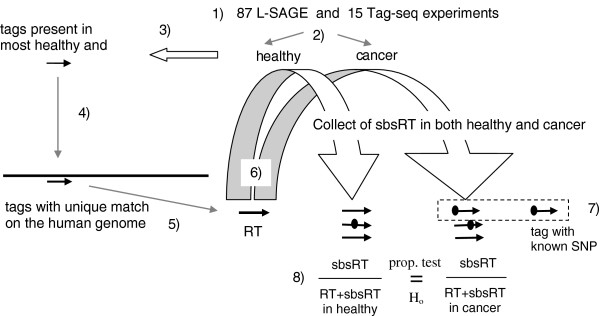
**Bioinformatics workflow.** 1) L-SAGE and Tag-seq experiments were processed separately 2) Experiments were divided in 2 groups, namely healthy and cancer 3) tags (thin black arrow) present in at least k% of healthy and at least k% of cancer experiments (k = 75% for L-SAGE and k = 90% for Tag-seq) were selected 4) The tags 5' boundaries were extended with the CATG (*NlaIII*) motif generating 4 + 17 = 21 base sequences and aligned on the human genome (long and thick black line) using blastn 5) tags matching the human genome exactly once were selected as RT (thick black arrow) 6) For each RT, sbsRT (thick black arrows carrying an ellipse) were searched among all the tags and were collected with their counts. 7) sbsRT matching a known SNP were excluded from SBS accounting (discontinuous rectangle) 8) For each RT, i.e. for each transcript, 2 proportions of sbsRT were calculated, i.e. 1 in healthy and 1 in cancer. Finally, both proportions were statistically tested for equality.

87 L-SAGE and 15 Tag-seq experiments were selected on the NCBI Gene Expression Omnibus (GEO) repository [21]. L-SAGE experiments were grouped into 40 healthy and 47 cancers. Sixteen different tissues or cell types were represented (Additional file 3). Tag-seq experiments were grouped into 7 healthy and 8 cancers. All selected Tag-seq experiments originated from skin or foreskin biopsies. Since the total number of tags produced by L-SAGE and Tag-seq experiments were dramatically different and because the sequencing error rates of Sanger and deep sequencing methods may be unequal, L-SAGE and Tag-seq tags were processed using the same bioinformatics workflow but separately (Figure 1).

### Reference Tags (RT)

2,930 tags were present in at least 75% of the 40 healthy and at least 75% of the 47 cancer L-SAGE experiments. Among these 2,930 tags, 1,966 matched the human genome sequence exactly once. Seven tags had a sequence composition bias and were discarded. Thus, 1,959 distinct tags were selected as RT (= L-SAGE list of RT). 11,967 tags were present in at least 90% of the 7 healthy and at least 90% of the 8 cancer Tag-seq experiments. Among these 11,967 tags, 8,806 matched the human genome sequence exactly once, 234 were discarded because of sequence composition bias and 8,572 distinct tags were selected as RT (=Tag-seq list of RT). 1,878 tags were common to both L-SAGE and Tag-seq lists of RT. In theory, a RT can generate 51 (= 3 × 17) possible distinct sequences by SBS, therefore each RT may be associated with 51 sbsRT. For each RT, the frequencies of sbsRT in both cancer and healthy cells were calculated. COG3 (alias SEC34) and SRP9 3’ polyadenylated transcripts were recorded in genbank with AF332595 and EF488978 accession numbers respectively. The 17 base *NlaIII* tags of SRP9 and COG3 transcripts were determined using genbank sequence records. However, SRP9 and COG3 17 base *NlaIII* tags were not present among the L-SAGE and Tag-seq lists of RT. Conversely, GAPDH, VIM, ENO1, HSPA8, TPT1, ATP5A1, FTL, TPI1, ALDOA and LDHA 17 base *NlaIII* tags were present among the L-SAGE or Tag-seq lists of RT.

### Increased SBS frequencies in transcripts expressed in cancer cells

For each of the 1,959 RT that were selected using L-SAGE experiments, sbsRT proportions in cancer and healthy cells were tested for equality (H_o_) against the alternative hypothesis that sbsRT proportions were greater in cancer cells (H_1_): H_o_ was rejected for 529 out of 1,959 RT by multiple 1-side Pearson’s chi-squared proportion tests with α/2 = 0.025 risk of type I error. A Benjamini-Hochberg False Discovery Rate (FDR) was applied and 372 out of 529 RT passed FDR at 2.5%. As a result, 372 RT (19% of 1,959) showed significantly greater SBS in cancer than in healthy cells (Additional file 4). The same H_o_ was tested against the alternative hypothesis that sbsRT proportions were greater in healthy cells (H_1_): H_o_ was rejected for 66 RT by multiple 1-side Pearson’s chi-squared proportion tests with α/2 = 0.025 and 17 RT passed FDR at 2.5%, i.e. ~0.9% of 1,959. No difference between cancer and healthy cells was detected for 1,570 RT (80%). RT were associated with transcripts using the Sagettarius tool [22]. Among the RT with top ranking SBS frequencies in cancer, GAPDH and TPI1 were present, i.e. 2 mRNA that had been previously reported by Brulliard M. et al. using EST aligned on reference transcripts. Interestingly, no somatic mutation was recorded in the COSMIC database [23] for both genes, indicating that SBS observed at the transcript level were not supported by any known base variation at the genome level. Since known SNP occurring in *NlaIII* tags [24] were excluded from sbsRT accounting, SNP could not support the increased SBS frequency in cancer. Further transcripts identified by the EST study, namely ATP5A1, TPT1, LDHA and ENO1 were also present among the 372 RT. In conclusion, 6 mRNA out of the 15 identified by Brulliard M. et al. were confirmed (Table 1). SBS frequencies were calculated for each of the 8,572 RT selected using the Tag-seq experiments. SBS frequencies in healthy and cancer cells were tested for equality (H_o_) against the alternative hypothesis that sbsRT proportions were greater in cancer (H_1_): H_o_ was rejected by multiple 1-side Pearson’s chi-squared proportion tests with a α/2 = 0.025 for 4,465 RT. 4,289 RT passed FDR at 2.5%. As a result, 4,289 RT (50% of 8,572) showed significantly greater SBS in cancer than in healthy cells (Additional file 5). The same H_o_ was tested against the alternative hypothesis that sbsRT proportions were greater in healthy cells (H_1_): H_o_ was rejected for 1,417 RT by multiple 1-side Pearson’s chi-squared proportion tests with a α/2 = 0.025, 1,123 RT passed FDR at 2.5% (13%). For 3,160 RT (37%), no difference was observed between healthy and cancer cells. Using Tag-seq experiments, the list of RT showing greater SBS frequencies in cancer was 11.5 times longer than the list produced by L-SAGE experiments. Thus, both L-SAGE and Tag-seq experiments concurred with the notion that a population of transcripts had more SBS in cancer than in healthy cells. Nine out of the 15 transcripts identified by the EST study were confirmed by the Tag-seq experiments (Table 1).

**Table 1:** **Testing SBS frequency equality in healthy** (**H**) **and cancer** (**C**) **cells for the 17 mRNA selected by Brulliard**, **M**. **et al**. (**2007**)

**Gene**	**COSMIC**	**L**-**SAGE**	**Tag**-**seq**	**Brulliard**, **M**. **et al**. **TI study using EST**
GAPDH	0	C > H (3.67×10^-115^)	C > H (~0)	C > H
VIM	13	C = H	C > H (2.32×10^-78^)	C > H
ENO1	7	C > H (3.48×10^-3^)	C < H (0.76×10^-2^)	C > H
HSPA8	10	*RT*	C > H (9×10^-9^)	C > H
TPT1	0	C > H (4.05×10^-4^)	C > H (~0)	C > H
ATP5A1	5	C > H (1.51×10^-15^)	C > H (1.35x10^-83^)	C > H
FTL	0	*RT*	C > H (1.5×10 ^-7^)	C > H
TPI1	3	C > H (1.14×10^-52^)	C > H (~0)	C > H
ALDOA	4	C = H	C < H (5.55×10^-23^)	C > H
LDHA	4	C > H (6.98×10^-14^)	C > H (1.84×10^-3^)	C > H
FTH1	5	*RT*	*RT*	C > H
RPS4X	2	*RT*	*RT*	C > H
RPL7A	1	3’ polyadenylated RNA record not available		C > H
RPS6	0	*RT*	*RT*	C > H
CALM2	1	*RT*	*RT*	C > H
TMSB4X	1	*RT*	*RT*	C < H
ALB	17	*RT*	*RT*	C < H

C > H: greater SBS frequency in cancer. C < H: greater SBS frequency in healthy. C = N: SBS frequencies are not significantly different between H and C. *RT*: the 17 base *NlaIII* tag associated with this transcript was not present in either L-SAGE or Tag-seq lists of RT and therefore equality between SBS frequencies in H and C could not be tested. COSMIC: number of cancer related somatic mutations recorded by the COSMIC database. 3’ polyadenylated RNA record not available: the sequence recorded in RefSeq-rna does not have a 3’ polyadenylated boundary, therefore *NlaIII* tag cannot be determined.

### Known cancer somatic mutations do not support increased SBS frequencies in mRNA

**Figure 2:**
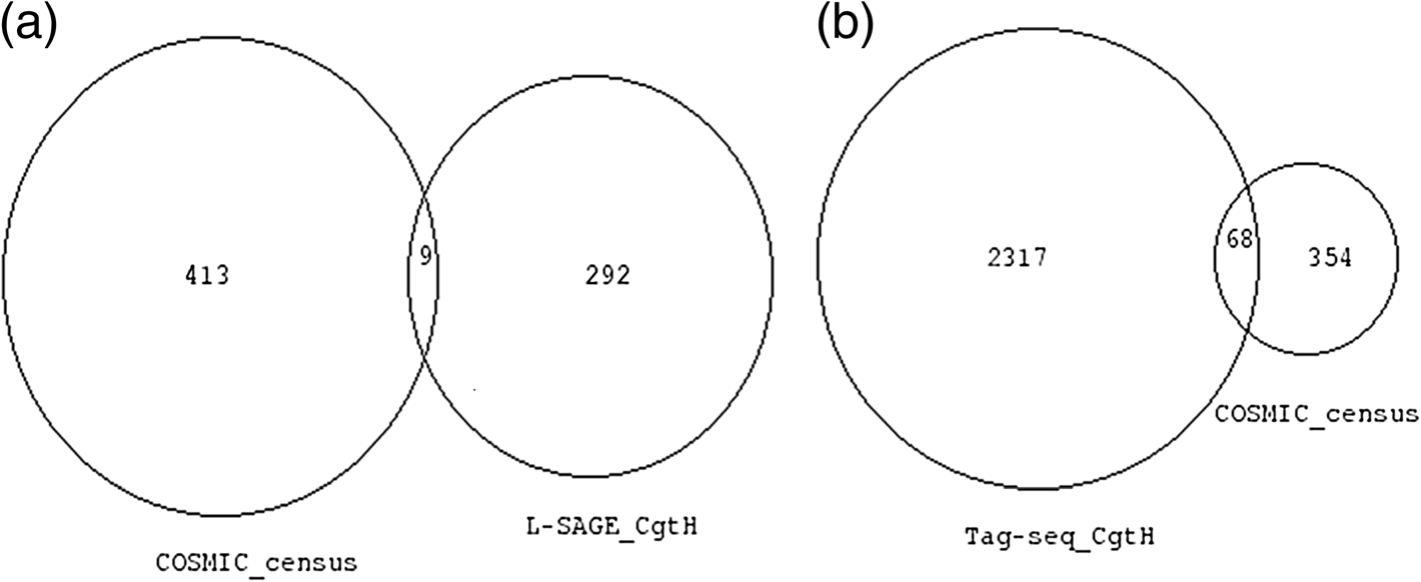
**Venn diagrams of transcripts with greater SBS frequencies and genes with cancer somatic mutations.** Numbers represent NCBI gene ID. Venn diagram areas are proportional to gene ID numbers. (**a**) Left circle = COSMIC (v56) census of 422 somatically mutated genes in cancer, right circle = transcripts with greater SBS frequencies in cancer than in healthy cells in L-SAGE experiments. Nine transcripts were common to both lists. For each of these 9 transcripts, no known cancer-related somatic mutations altered the 17 base *NlaIII* tag. (**b**) Right circle = COSMIC (v56) census of 422 somatically mutated genes in cancer. Left circle = transcripts with greater SBS frequencies in cancer than in healthy cells in Tag-seq experiments. Sixty eight transcripts were common to both lists. For each of these 68 transcripts, no known cancer related somatic mutations altered the 17 base *NlaIII* tag.

A census of 422 genes that are somatically mutated in cancer has been made available by the COSMIC database. Venn diagrams were drawn between the census of somatically mutated genes and the transcripts that had greater SBS in cancer than in healthy cells using L-SAGE (Figure 2a) and Tag-seq (Figure 2b) experiments. Nine genes were common to the census and L-SAGE transcripts and 68 were common to the census and the Tag-seq transcripts. We thoroughly checked whether known somatic mutations altered the 17 base *NlaIII* tag for each of the 9 (Additional file 6) and 68 (Additional file 7) genes. The 17 base *NlaIII* tag was generally located in the vicinity of the transcript 3’ polyadenylated boundary, i.e. in the 3’ UTR. For a majority of genes, the most 3’ known somatic mutation altered the coding sequence of the gene and was thus 5’ to the 17 base *NlaIII* tag. No known somatic mutation altered the 17 base *NlaIII* tags. Therefore, the increased SBS frequencies that were observed in transcripts expressed in cancer cells could not be attributed to known somatic mutations.

### APOBEC1 or ADAR RNA-editing do not support increased SBS frequencies

**Figure 3:**
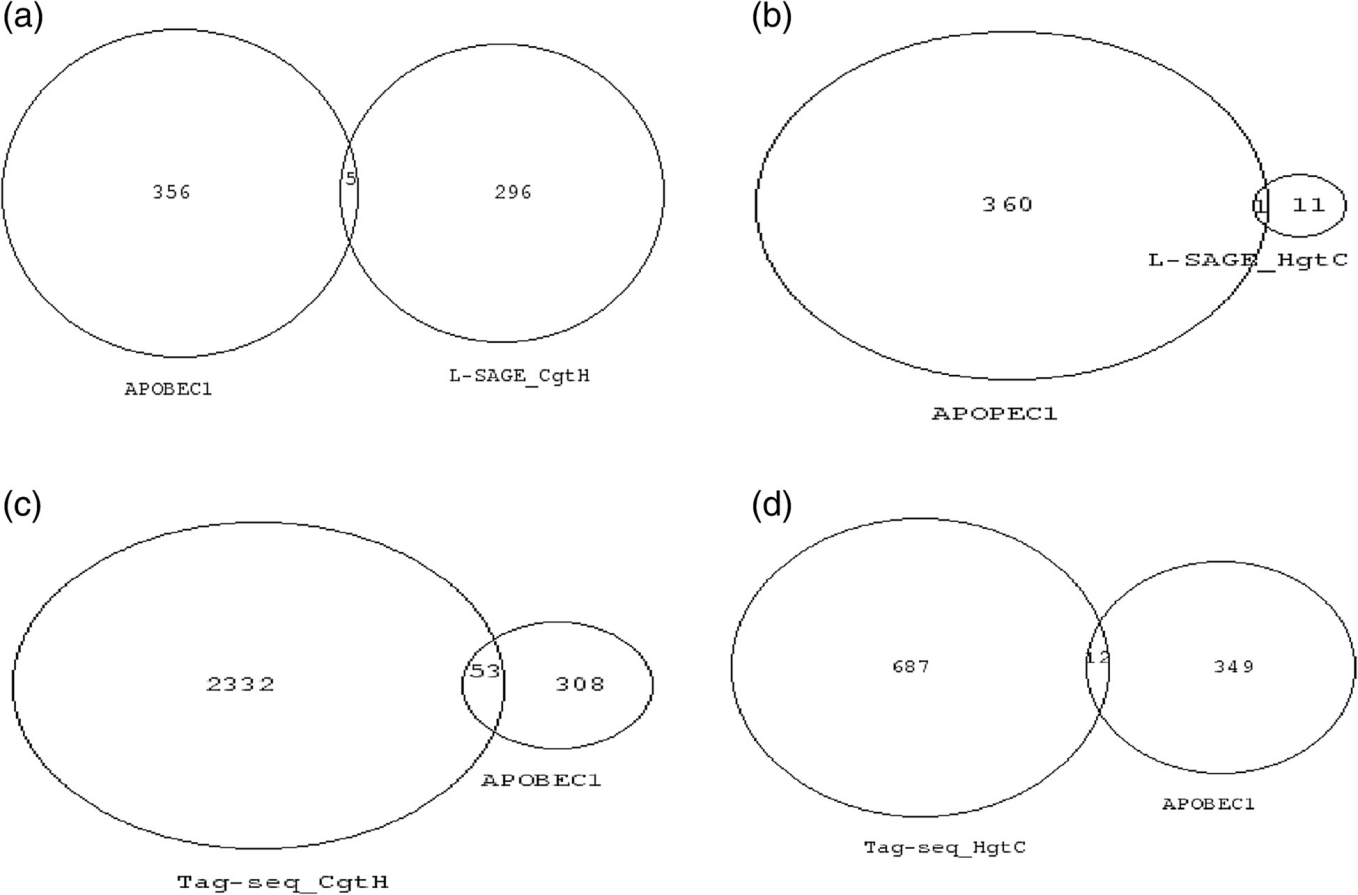
**Venn diagrams of transcripts with greater SBS frequencies and APOBEC1 RNA**-**editing targets.** Numbers represent NCBI gene ID. Venn diagram areas are proportional to gene ID numbers. (**a**) Left circle = APOBEC1 RNA-editing targets, right circle = transcripts with greater SBS frequencies in cancer cells in L-SAGE experiments. (**b**) Left circle = APOBEC1 RNA-editing targets, right circle = transcripts with greater SBS frequencies in healthy cells in L-SAGE experiments. (**c**) Right circle = APOBEC1 RNA-editing targets, left circle = transcripts with greater SBS frequencies in cancer cells in Tag-seq experiments. (**d**) Right circle = APOBEC1 RNA-editing targets, left circle = transcripts with greater SBS frequencies in healthy cells in Tag-seq experiments.

Rosenberg B.R. et al. published a list of 32 experimentally validated APOBEC1 mRNA-editing sites in 30 distinct mouse transcripts. Comparing the transcript sequences surrounding the C to U edition position, a mooring pattern, i.e. WCWN_2-4_WRAUYANUAU, had been defined. Moreover, the RNA-editing site occurs in a 16 base region directly 5’ to the mooring sequence. Rosenberg B.R. et al. used the mooring pattern to predict additional transcripts that could be edited by APOBEC1. Finally, a list of 383 transcripts (=361 NCBI gene ID after synergizer conversion) was proposed as either experimentally validated or predicted targets of APOBEC1. We crossed this APOBEC1 list of RNA-editing targets with the 372 transcripts (=301 NCBI gene ID after synergizer conversion) that showed greater SBS frequencies in cancer than in healthy cells using L-SAGE experiments. Five transcripts were common to both lists (Figure 3a). For each of the 5 transcripts, the positions of the 17 base *NlaIII* tag and the mooring sequence were determined. None of the 17 base *NlaIII* tags could be potentially edited by APOBEC1 because the mooring sequence and the tag were distant from each other in the transcript sequence (Additional file 8). The beta-2 microglobulin was the only transcript common to both APOBEC1 RNA-editing targets and transcripts with greater SBS in healthy than cancer cells (Figure 3b). However, the mooring sequence was not conserved between mouse and human beta-2 microglobulin transcripts. Using Tag-seq, 53 transcripts that had greater SBS in cancer were present among APOBEC1 RNA-editing targets (Figure 3c), and 8 of them had been experimentally validated. However, none of these 8 transcripts had a 17 base *NlaIII* tag that overlapped the APOBEC1 editing site (Additional file 9). Finally, for transcripts with greater SBS in healthy than cancer obtained using Tag-seq, only 1, namely FARSB, had a 17 base *NlaIII* tag that overlapped the APOBEC-1 RNA-editing site. We thus cannot exclude that increased SBS frequency observed for FARSB could be the result of an APOBEC1 edition. Adenosine to inosine conversions carried out by the ADAR family of RNA-editing enzymes occur in non-coding repetitive sequences, mostly *Alu* elements. *Alu* sequences are dispersed along the genome and can also be integrated in mRNA. Since RT mono-localize on the genome, they cannot match *Alu* sequences, which excludes that increased SBS frequencies that were observed in transcripts expressed in cancer or healthy could originate from ADAR editions.

### Wide range of molecular functions potentially affected by increased SBS frequencies

For L-SAGE, 1,879 (96%) RT out of 1,959 could be associated with a transcript (=L-SAGE background list). 355 RT out of the 372 that showed greater SBS frequencies in cancer (=L-SAGE query list) associated with a transcript. GO analysis using DAVID [25] determined that the “Translation” biological process was over-represented among the 355 transcripts (p-value = 6×10^-7^, Benjamini-Hochberg = 10^-3^). The “Ribosome” cellular localization was also enriched (p-value = 1.8×10^-5^, Benjamini-Hochberg = 5.7×10^-3^). For Tag-seq experiments, 7,830 (91%) RT out of 8,572 were mapped to a transcript (=Tag-seq background list). Among the 4,289 RT that showed greater SBS in cancer, 3,953 could be associated with a transcript (=Tag-seq query list n°1). 1,053 (94%) out of the 1,123 RT that showed greater SBS in healthy cells associated with a transcript (=Tag-seq query list n°2). However, no GO term enrichment was present in both Tag-seq query lists. As a result, many different biological processes or molecular functions could be potentially represented among transcripts with greater SBS in cancer.

### Increased diversity of SBS in transcripts expressed in cancer cells

**Figure 4:**
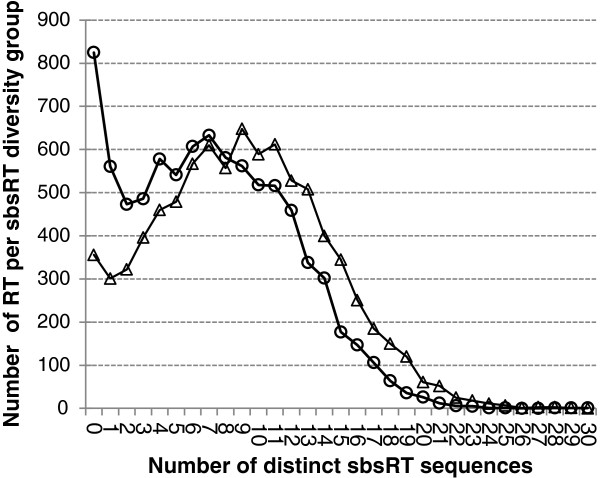
**Diversity of sbsRT in healthy and cancer cells.** SBS were tracked for 8,572 RT in 7 healthy (circles) and 8 cancer (triangles) Tag-seq experiments. The 8,572 RT were distributed into sequence diversity groups according to the number of distinct sbsRT sequences. For example, group 0, i.e. RT for which 0 sbsRT were observed, contained approximately 350 RT in cancer and slightly more than 800 RT in healthy cells. RT associated with more than 30 distinct sbsRT were rare.

The diversity of sbsRT sequences was not studied using L-SAGE experiments because the sums of total tags in healthy (4.7 million) and cancer (2.9 million) were unbalanced. By contrast, in the Tag-seq experiments, the sums of total tags counts in healthy and cancer cells were quite balanced, i.e. 33.4 and 34.4 million (+3%), respectively. For each of the 8,572 RT, the number of distinct sbsRT sequences i) in healthy and ii) in cancer was determined. In healthy and cancer, the 8,572 RT generated 60,854 and 76,967 (+26%) distinct sbsRT sequences, respectively. Thus, sbsRT diversity was greater in cancer and this could not be explained by the +3% difference in the sums of total tags counts. The 8,572 RT were separated into groups according to the number of distinct sbsRT observed in cancer or healthy cells, i.e. the *i*^*t*h^ group contained the RT for which exactly *i* distinct sbsRT were observed. All RT associated with 0 to 44 distinct sbsRT sequences. Maximal diversity of 51 distinct sbsRT was never observed for any RT. In experiments carried out in healthy and cancer cells, 825 and 356 RT were associated with 0 sbsRT, respectively (Figure 4). 5,281 and 4,048 RT had 8 or less distinct sbsRT sequences in healthy and cancer, respectively. Conversely, 3,291 and 4,524 RT had more than 8 sbsRT in healthy and cancer respectively. RT seemed thus heterogeneously distributed between cancer and healthy cells when analyzing sbsRT diversity (*χ*^2^ test at α = 5%, p-value < 2.2 x 10^-16^). We concluded that i) cancer introduced heterogeneity in sbsRT diversity ii) sbsRT diversity was greater in cancer.

### Heterogeneity of ST proportions across healthy and cancer cells

**Figure 5:**
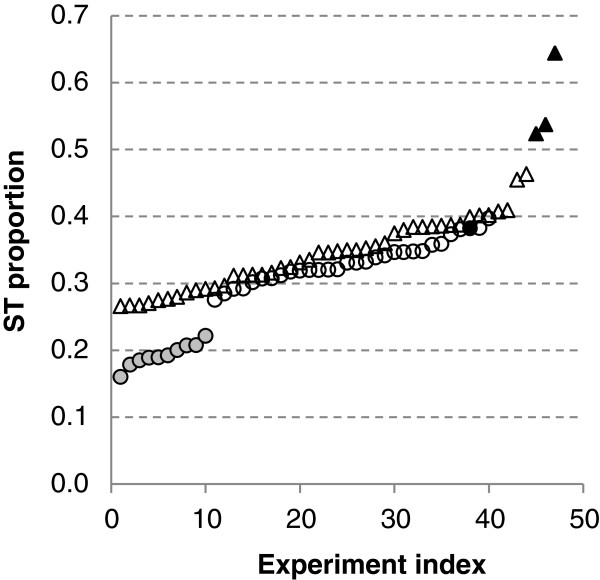
**ST proportions calculated for each L**-**SAGE experiment.** Each symbol represents 1 experiment: 47 cancer (triangles) and 40 healthy (circles), circles filled with gray = ESC, triangles filled with black = L-SAGE experiments carried out on the tumor cells of a single patient, circle filled with black = L-SAGE experiment carried out on the healthy cells of the same patient. Experiments were sorted in ascending ST proportion order.

ST were not reported in Tag-seq experiments on GEO records. In fact, tags counts were greater or equal than 2. Conversely, ST were reported in L-SAGE. First, ST proportions were calculated for each of the 87 L-SAGE experiments. Second, these proportions were sorted in ascending order (Figure 5). Most experiments had ST proportions in the range of 0.25 to 0.42. However, a group of 10 experiments carried out in healthy cells, namely ESC, showed ST proportions that were lower than any other (~0.2). Furthermore, the greatest ST proportions were observed in cancer cells. Interestingly, within a series of 4 L-SAGE experiments carried out on the biopsies of a single patient at 1 healthy and 3 disease stages, the ST proportions were greater in cancer cells than in healthy cells.

### Lowest ST proportions in transcripts expressed in ESC

**Figure 6:**
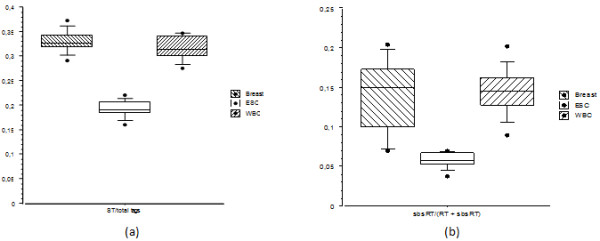
**Distribution of ST proportions and global sbsRT proportions in 3 distinct healthy tissues.** 32 L-SAGE experiments were used, namely "Breast" (12 experiments), "ESC" (10 experiments) and "White Blood Cells" (WBC) (10 experiments). For each experiment, the (**a**) ST proportion and the (**b**) global sbsRT proportion were calculated. 583 RT were used for Breast, 1.478 for ESC, and 860 for WBC to calculate the global sbsRT proportion, respectively.

Remarkably, ESC had ST proportions ranging between 0.15 and 0.23. To test the significance of this difference, experiments carried out in most represented healthy tissues were separated into 3 groups, namely breast (12 experiments), White Blood Cells (WBC) (10 experiments) and ESC (10 experiments). For breast, WBC and ESC, the median of ST proportions were 0.33, 0.31 and 0.19, respectively (Figure 6a). A one way-Analysis of variance (Anova) with the "cell type" factor at 3 modalities (breast, WBC, ESC,) was carried out. A mathematical transformation was applied to the proportions, i.e. the arcsin(square root(ST proportion)). The transformed proportions were considered independent since the experiments were carried out on different cell types (Anova 1^st^ condition). A Shapiro-Wilk test was applied to check the distribution normality of the transformed proportions for each of the 3 cell types (breast: p-value = 0.8978; WBC: p-value = 0.6676; ESC: p-value = 0.9206), the hypothesis of "normal distribution" were thus accepted (Anova 2^nd^ condition). The hypothesis of variance equality between breast, ESC and WBC was accepted using the Bartlett test (Anova 3^rd^ condition) (p-value = 0.88). Finally, the equality of ST transformed proportion means across the 3 cell types was rejected (p-value = 1.47 x 10^-15^). This showed that at least one cell type, obviously ESC, had a ST proportion significantly different than breast and WBC.

### Lowest SBS frequency in transcripts expressed in ESC

1,748; 583; and 860 RT out of the 1,959 that were selected in the L-SAGE experiments were present in 100% of the 10 ESC, 100% of the 12 breast and 100% of the 10 WBC experiments, respectively. For each experiment, a global sbsRT proportion was calculated and the means were determined, i.e. 0.14 (breast), 0.15 (WBC) and 0.058 (ESC) (Figure 6b). ESC had thus the lowest mean. We tested the significance of the differences between global sbsRT proportion means across the 3 cell types. The hypothesis of normal distributions for the transformed global proportions calculated on breast, WBC and ESC were accepted with a Shapiro-Wilk test (p-value = 0.30, 0.79 and 0.22 respectively). However, the equality of variance was rejected by a Bartlett test (p-value = 0.005). A non-parametric Kruskal-Wallis test rejected the equality between the transformed global sbsRT proportion means in breast, WBC and ESC with a 5.4 x 10^-5^ p-value. This showed that ESC had a SBS frequency in transcripts that was significantly different from the other two cell types.

### ST proportions and SBS frequencies correlate and increase during cancer progression

**Figure 7:**
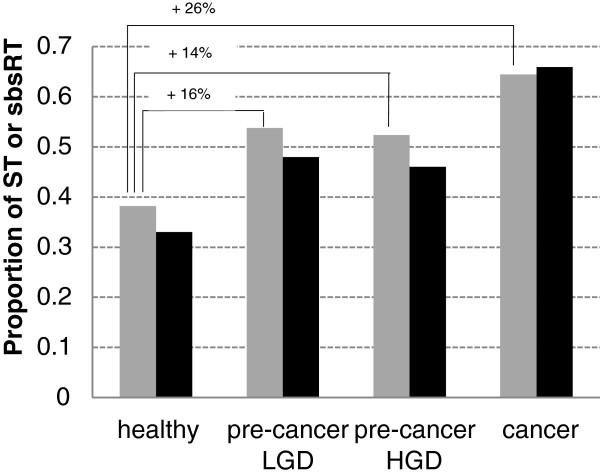
**Single patient personalized monitoring of ST and global sbsRT proportions during disease progression.** ST and global sbsRT proportions were calculated for 4 L-SAGE experiments carried out on the biopsies of a single patient at 1 healthy and 3 consecutive tumor stages. Gray bars = ST proportions, black bars = global sbsRT proportions. Low-grade dysplasia (LGD), High-grade dysplasia (HGD) and adenocarcinoma (cancer). The global sbsRT proportions were calculated using 1.435 RT present in all 4 experiments. Significance of ST proportion differences between healthy, LGD, HGD and adenocarcinoma was calculated using the Pearson’s chi-squared proportion test (p-value < 2.2 x 10^-16^).

In the previously mentioned series of 4 L-SAGE experiments carried out on the biopsies of a single patient, 1 healthy and 3 consecutive tumor stages, i.e. Low-Grade Dysplasia (LGD), High-Grade Dysplasia (HGD) and Adenocarcinoma (AC), were recorded [26]. The sums of total tags counts for each 4 experiments were balanced (mean = 75,735 tags, standard deviation = 2,061 tags). In these 4 experiments, the ST proportions dramatically increased from 0.38 (healthy) to ~0,52 (LGD, HGD) and 0.64 (AC) (Figure 7). Of particular note, the percent of ST that could not be associated with any transcript also increased from 83% in healthy to 87% in both LGD and HGD, and 92% in AC. In the 4 experiments, 2,271 tags were co-present and among them 1,435 belonged to the list of the 1,959 L-SAGE RT. A global sbsRT proportion was calculated for each experiment using the co-present RT. Global sbsRT proportions increased from 0.33 in healthy, to ~0.47 (LGD, HGD) and 0.66 in AC, showing that SBS frequencies was increasing with the tumor stage. Since SBS occurring in RT is a possible cause of tag with transcript association failure, this may explain why the percent of unassociated ST increased from healthy to AC. No significant difference was observed between LGD and HGD suggesting that these phenotypes were not related to an increase of SBS in mRNA sequences. The Pearson's coefficient calculated between the ST and global sbsRT proportions on the 4 experiments showed a strong correlation (0.98). ST proportion seemed thus to be an accurate indicator of SBS frequency in transcripts.

## Discussion

In the present study, we provide evidence for an increased frequency of SBS that occur in a population of transcripts expressed in cancer cells. Known SNP, catalogued cancer related somatic mutations and predicted or validated targets of RNA-editing enzymes did not support the increased SBS frequency in cancer. However, the transcripts but not the genome of healthy and tumors cells were available and thus transcript and genome sequences both originating from the same patient could not be directly compared. To fully confirm that increased base conflicts exist between transcript and genome sequences in patient’s tumors, back-to-back exome sequencing and RNA-seq would be required. Using Tag-seq, 1,123 RT had greater SBS in healthy than in cancer cells, therefore questioning the reliability of this result. In fact, ST had been removed from Tag-seq experiments recorded in GEO and thus 30% of the tags data was unavailable. As ST represent a reservoir of SBS, their removal may have introduced a bias in sbsRT accounting. Moreover, slight heterogeneity of sequencing quality between platforms cannot be excluded. Some Tag-seq experiments carried out in healthy cells may have been produced with poor sequencing quality and thus may have introduced more SBS than in cancer cells. Finally sequence biases such as read redundancy have been reported in deep sequencing. Using RNA-seq, read redundancy can be cleaned by bioinformatics programs. Conversely, tag redundancy produced by deep sequencing bias cannot be cleaned in Tag-seq experiments. ST have been considered as low quality sequences, i.e. enriched in sequencing errors and may be excluded from analysis by standard bioinformatics procedures. Here, we agree with previous statements that in fact valuable information is available in ST [27]. Furthermore, L-SAGE and Tag-seq may be so sensitive that they can detect base errors introduced by the cell transcriptional machinery or RNA-editing. ST are thus an archive of mRNA sequence alterations either due to sequencing errors, TI, or RNA-editing and should not be sacrificed for the benefit of disk space sparing. Moreover, the proportion of ST per experiment has proved to be an accurate indicator of SBS frequency in transcripts. An unexpected high level of SBS in tags produced by L-SAGE experiments had already been reported in a previous study [28]. Using 29 publicly available L-SAGE libraries - that were also used in our study - and aligning the tags on the human genome sequence, the conclusion that the sequencing error rate might have been underestimated was drawn since a large number of tags did not match the genome after having taken into account the currently accepted 1% base error rate of L-SAGE tags. However, in this previous study both healthy and cancer experiments were mixed, i.e. cancer was not suspected to introduce additional SBS in transcripts. The molecular mechanism underlying increased TI in cancer is still elusive. Brulliard et al. speculated that increased TI might be due to defective transcription assisted proofreading activity. In fact, transcriptional fidelity relies i) on the ability of RNA polymerases to select the correct base before incorporation, ii) to impair RNA extension beyond a mismatch, iii) to cleave a mismatched base at the RNA 3’ boundary and resume RNA synthesis [29,30]. Dysfunction at any of these 3 crucial steps is likely to compromise RNA sequence integrity. However, cancer related somatic mutations have not been reported so far in genes coding for RNA polymerases. Conversely, mice deficient for DNA polymerase δ proofreading activity have been associated with a high incidence of epithelial cancer [31]. Mutations in genes that code for proteins involved in mRNA synthesis could be searched in patients showing an increased SBS frequency. In ESC, the transcription of the genome is globally hyperactive [32]. No information has been made available on transcriptional fidelity in ESC. Comparing SBS frequencies across different cell types, we uncovered that ESC had a very low SBS frequency. This finding is in favor of a transcriptional fidelity which might be greater in ESC than in differentiated cells. We provided strong evidences that SBS frequency is significantly increased for a population of transcripts expressed in cancer cells. However, further investigations are required to determine whether this feature is common to all cancers or whether it is only present in some malignancies or in a subset of patients.

## Conclusions

SBS frequency in transcript sequences is heterogenously distributed across cells, i.e. ESC have the lowest, cancer cells have the greatest and healthy differentiated cells may lie “in between”. Therefore, SBS frequency in transcript sequences could represent a new cancer specific biomarker which may be useful to characterize patient’s tumors. With the reduction of sequencing cost, cancer diagnostic could be aided by the determination of SBS frequency in transcripts expressed in tumors. In the future, drugs or gene therapies which may prove particularly efficient to treat patient’s tumors showing increased SBS frequency in transcripts could be valuable and thus intensively searched.

## Abbreviations

L-SAGE: Long serial analysis of gene expression; SBS: Single base substitution; RT: Reference tag; sbsRT: Single base substituted reference tag; ST: Single tag; SNP: Single nucleotide polymorphism; ESC: Embryonic stem cells; WBC: White blood cells; GEO: Gene expression omnibus; TI: Transcriptional infidelity; EST: Expressed sequence tag; LGD: Low grade dysplasia; HGD: High-grade dysplasia; AC: Adenocarcinoma.

## Competing interests

The authors declare that they have no competing interests.

## Authors’ contributions

LB and OP designed the study. LB, DK and RF performed the experiments. LB, DK and OP interpreted the results. LB drafts the manuscript. LB and OP wrote the final version of the manuscript. All authors read and approved the final manuscript.

## Pre-publication history

The pre-publication history for this paper can be accessed here:

http://www.biomedcentral.com/1471-2407/12/509/prepub

## Supplementary Material

Additional file 1 Generation of 17 distinct patterns for each RT. Click here for file

Additional file 2 Risk that a sbsRT matches by chance the RT of a transcript. Click here for file

Additional file 3 L-SAGE and Tag-seq experiment sizes (sequencing effort). Click here for file

Additional file 4 RT with greater SBS frequency in cancer than in healthy cells (L-SAGE). Click here for file

Additional file 5 RT with greater SBS frequency in cancer than in healthy cells (Tag-seq). Click here for file

Additional file 6 Manual checking of possible cancer related somatic mutations altering RT with greater SBS frequencies in cancer than in healthy cells (L-SAGE). Click here for file

Additional file 7 Manual checking of possible cancer related somatic mutations altering RT with greater SBS frequencies in cancer than in healthy cells (Tag-seq). Click here for file

Additional file 8 Manual checking of possible APOBEC1 RNA-editing targets altering RT with greater SBS frequencies in cancer than in healthy cells (L-SAGE). Click here for file

Additional file 9 Manual checking of possible APOBEC1 RNA-editing targets altering RT with greater SBS frequencies in cancer than in healthy cells (Tag-seq). Click here for file
