# Supplementary material for: Increased frequency of single base substitutions in a population of transcripts expressed in cancer cells
Source: BMC Cancer. 2012 Nov 8;12:509. doi: 10.1186/1471-2407-12-509 (PMC3522053; doi:10.1186/1471-2407-12-509)
Supplement: Additional file 1 — Generation of 17 distinct patterns for each RT. [file 1471-2407-12-509-S1.pdf]

Additional_method_file_1

Generation of 17 distinct patterns for each RT

In Sybase Structured Query Language (SQL), the "_" meta-character means "any character".

Example of SBS automatically carried out in a RT:

GCCTCTGCCAGATGGCT (RT)

Base position	Pattern		possible sbsRT
1	_CCTCTGCCAGATGGCT	=>	[A,C,T]CCTCTGCCAGATGGCT
2	G_CTCTGCCAGATGGCT	=>	G[A,T,G]CTCTGCCAGATGGCT
3	GC_TCTGCCAGATGGCT	=>	GC[A,T,G]TCTGCCAGATGGCT
4	GCC_CTGCCAGATGGCT	=>	GCC[A,C,G]CTGCCAGATGGCT
5	GCCT_TGCCAGATGGCT	=>	GCCT[A,T,G]TGCCAGATGGCT
6	GCCTC_GCCAGATGGCT	=>	GCCTC[A,C,G]GCCAGATGGCT
7	GCCTCT_CCAGATGGCT	=>	GCCTCT[A,C,T]CCAGATGGCT
8	GCCTCTG_CAGATGGCT	=>	GCCTCTG[A,T,G]CAGATGGCT
9	GCCTCTGC_AGATGGCT	=>	GCCTCTGC[A,C,G]AGATGGCT
10	GCCTCTGCC_GATGGCT	=>	GCCTCTGCC[C,T,G]GATGGCT
11	GCCTCTGCCA_ATGGCT	=>	GCCTCTGCCA[A,C,T]ATGGCT
12	GCCTCTGCCAG_TGGCT	=>	GCCTCTGCCAG[C,T,G]TGGCT
13	GCCTCTGCCAGA_GGCT	=>	GCCTCTGCCAGA[A,C,G]GGCT
14	GCCTCTGCCAGAT_GCT	=>	GCCTCTGCCAGAT[A,C,T]GCT
15	GCCTCTGCCAGATG_CT	=>	GCCTCTGCCAGATG[A,C,T]CT
16	GCCTCTGCCAGATGG_T	=>	GCCTCTGCCAGATGG[A,T,G]T
17	GCCTCTGCCAGATGGC_	=>	GCCTCTGCCAGATGGC[A,C,G]

For each RT, the 17 patterns were used to match sbsRT that were present in L-SAGE and Tag-seq experiments.
