# Supplementary material for: Increased frequency of single base substitutions in a population of transcripts expressed in cancer cells
Source: BMC Cancer. 2012 Nov 8;12:509. doi: 10.1186/1471-2407-12-509 (PMC3522053; doi:10.1186/1471-2407-12-509)
Supplement: Additional file 2 — Risk that a sbsRT matches by chance the RT of a transcript. [file 1471-2407-12-509-S2.pdf]

Additional_method_file_2

Risk that a sbsRT matches by chance the RT of a transcript

In order to compute frequencies of SBS, a sbsRT must be specific to a unique RT. Here we calculated the risk that a sbsRT matches the RT of a transcript. A random 17 base string can generate 4^{17} = 17.2 x 10^{9} distinct sequences. Assuming 22,000 genes on the human genome and a 1 to 1 gene <-> tag association, a repertoire of 22,000 ET would be created (without taking into account mRNA 3' alternative splicing, alternative 3' poly-adenylation or redundance of ET associated with distinct genes). The probability that a random 17 base string matches the ET of a human transcript is thus p = 22.000 / (17.2 x 10^{9}) = 1.28 x 10^{-6}. A 17 base tag, which is the signature of a transcript, can generate 3 x 17 = 51 distinct sequences by SBS. The probability P that "At least 1 of the 51 tag resulting from SBS matches the tag of a transcript" is equal to 1 minus the probability of the complementary event, *i.e.* "none of the 51 tags matches the tag of a transcript" or 0 success in 51 identical and independent trials, thus P is given by a binomially distributed random variable, B (51, p). $P = 1 - ({}^{51}{}_{0}) . p^{0} . (1 - p)^{51 - 0} = 6.5 \times 10^{-5}$. This risk is thus very low, and we can trust that a sbsRT is specific to a unique RT.
