# Supplementary material for: Increased frequency of single base substitutions in a population of transcripts expressed in cancer cells
Source: BMC Cancer. 2012 Nov 8;12:509. doi: 10.1186/1471-2407-12-509 (PMC3522053; doi:10.1186/1471-2407-12-509)
Supplement: Additional file 6 — Manual checking of possible cancer related somatic mutations altering RT with greater SBS frequencies in cancer than in healthy cells (L-SAGE). [file 1471-2407-12-509-S6.pdf]

Additional_result_file_1 providing further information to Figure 2a:

The COSMIC census of somatically mutated genes in cancer was downloaded from http://www.sanger.ac.uk/genetics/CGP/Census/Table_1_full-2011-15.xls. In this census, 422 Gene ID (NCBI) were unique. Using the LongSAGE_CgtH_372_RT_FDR_mapped list of transcripts (Additional_tabular_file_2), 372 Genbank and RefSeq transcript ID were converted to 301 unique Gene ID. Nine genes (here below) were common to the census and the LongSAGE_CgtH_372_RT_FDR_mapped list.

**index**	**Gene ID**	**Description**	**Alteration of the 17 base *NlaIII* tag by known somatic mutations**
1	3181	HNRNPA2B1, heterogeneous nuclear ribonucleoprotein A2/B1	Known cancer-related somatic mutations do not alter the 17 base *NlaIII* tag
2	3326	HSP90AB1 heat shock protein 90kDa alpha (cytosolic), class B member 1	Known cancer-related somatic mutations do not alter the 17 base *NlaIII* tag
3	7913	DEK oncogene	Known cancer-related somatic mutations do not alter the 17 base *NlaIII* tag
4	4478	MSN, Moesin	Known cancer-related somatic mutations do not alter the 17 base *NlaIII* tag
5	833	CARS, cysteinyl-tRNA synthetase	Known cancer-related somatic mutations do not alter the 17 base *NlaIII* tag
6	1277	COL1A1, collagen, type I, alpha 1	Known cancer-related somatic mutations do not alter the 17 base *NlaIII* tag
7	1499	CTNNB1, catenin (cadherin-associated protein), beta 1, 88kDa	Known cancer-related somatic mutations do not alter the 17 base *NlaIII* tag
8	6146	RPL22, ribosomal protein L22	COSMIC does not report any somatic mutations in RPL22 although this gene has been recorded in the census
9	6794	STK11, serine/threonine kinase 11	Known cancer-related somatic mutations do not alter the 17 base *NlaIII* tag

For each of the 9 transcript sequences, the positions of the cancer related somatic mutations and the position of the *NlaIII* 17 base tag were located.

Legend:

Red nucleotide = cancer related somatically mutated base
Green nucleotide = 17 base *NlaIII* tag
Blue nucleotide = *NlaIII* “CATG” recognition site
Underlined nucleotides = Somatic mutation neighbor bases displayed on the COSMIC snapshot

1) Entrez Gene ID 3181 = HNRNPA2B1, heterogeneous nuclear ribonucleoprotein A2/B1 = Genbank BC000506

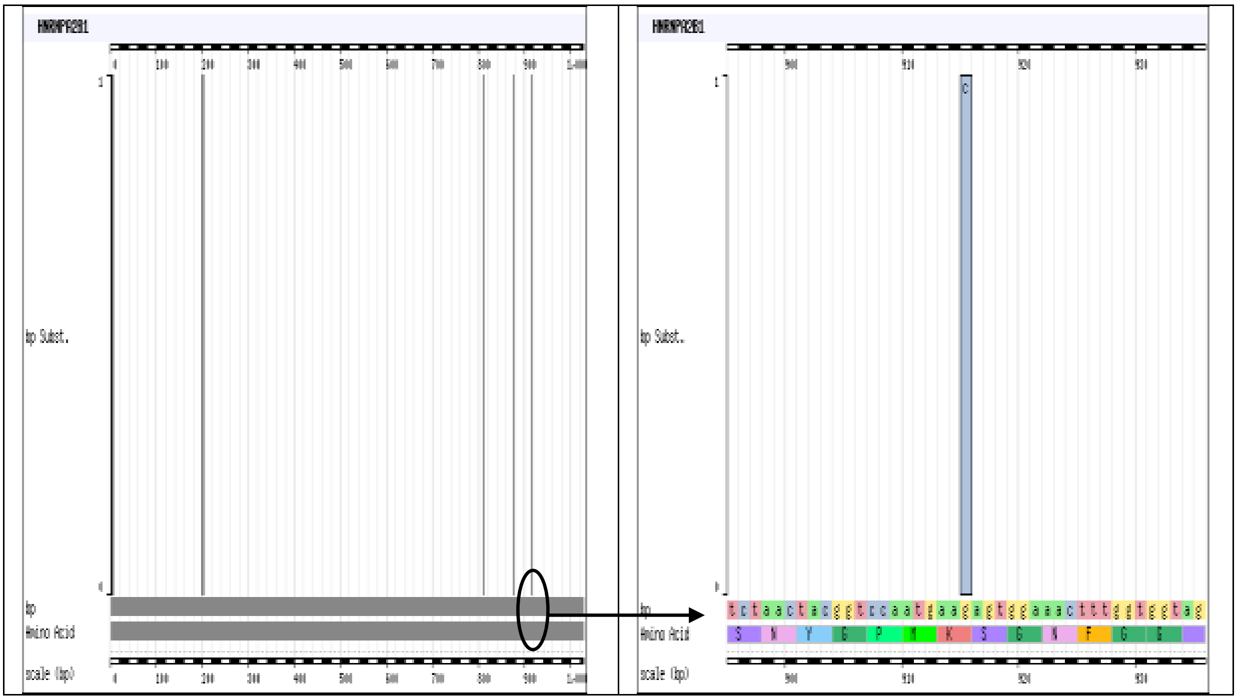

>gi|33875522|gb|BC000506.2| Homo sapiens heterogeneous nuclear ribonucleoprotein A2/B1, mRNA (cDNA clone MGC:8493 IMAGE:2822109), complete cds

```
GTGCGGAGGTGCTCCTCGCAGAGTTGTTTCTCGAGCAGCGGCAGTTCTCACTACAGCGCCAGGACGAGTC
CGGTTCGTGTTCGTCCGCGGAGATCTCTCTCATCTCGCTCGGCTGCGGGAAATCGGGCTGAAGCGACTGA
GTCCGCGATGGAGAGAGAAAAGGAACAGTTCCGTAAGCTCTTTATTGGTGGCTTAAGCTTTGAAACCACA
GAAGAAAGTTTGAGGAACTACTACGAACAATGGGGAAAGCTTACAGACTGTGTGGTAATGAGGGATCCTG
CAAGCAAAAGATCAAGAGGATTTGGTTTTGTAACTTTTTCATCCATGGCTGAGGTTGATGCTGCCATGGC
TGCAAGACCTCATTCAATTGATGGGAGAGTAGTTGAGCCAAAACGTGCTGTAGCAAGAGAGGAATCTGGA
AAACCAGGGGCTCATGTAACTGTGAAGAAGCTGTTTGTTGGCGGAATTAAAGAAGATACTGAGGAACATC
ACCTTAGAGATTACTTTGAGGAATATGGAAAAATTGATACCATTGAGATAATTACTGATAGGCAGTCTGG
AAAGAAAAGAGGCTTTGGCTTTGTTACTTTTGATGACCATGATCCTGTGGATAAAATCGTATTGCAGAAA
TACCATACCATCAATGGTCATAATGCAGAAGTAAGAAAGGCTTTGTCTAGACAAGAAATGCAGGAGGACC
TGGAGGTGGCAATTTTGGAGGTAGCCCCGGTTATGGAGGAGGAAGAGGAGGATATGGTGGTGGAGGACCT
GGATATGGCAACCAGGGTGGGGGCTACGGAGGTGGTTATGACAACTATGGAGGAGGAAATTATGGAAGTG
GAAATTACAATGATTTTGGAAATTATAACCAGCAACCTTCTAACTACGGTCCAATGAAGAGTGGAAACTT
TGGTGGTAGCAGGAACATGGGGGGACCATATGGTGGAGGAAACTATGGTCCAGGAGGCAGTGGAGGAAGT
GGGGGTTATGGTGGGAGGAGCCGATACTGAGCTTCTTCCTATTTGCCATGGGCTTCACTGTATAAATAGG
AGAGGATGAGAGCCCAGAGGTAACAGAACAGCTTCAGGTTATCGAAATAACAATGTTAAGGAAACTCTTA
TCTCAGTCATGCATAAATATGCAGTGATATGGCAGAAGACACCAGAGCAGATGCAGAGAGCCATTTTGTG
AATGGATTGGATTATTTAATAACATTACCTTACTGTGGAGGAAGGATTGTAAAAAAAAATGCCTTTGAGA
CAGTTTCTTAGCTTTTTAATTGTTGTTTCTTTCTAGTGGTCTTTGTAAGAGTGTAGAAGCATTCCTTCTT
TGATAATGTTAAATTTGTAAGTTTCAGGTGACATGTGAAACCTTTTTTAAGATTTTTCTCAAAGTTTTGA
AAAGCTATTAGCCAGGATCATGGTGTAATAAGACATAACGTTTTTCCTTTAAAAAAATTTAAGTGCGTGT
GTAGAGTTAAGAAGCTGTTGTACATTTATGATTTAATAAAATAATTCTAAAGGAAAAAAAAAAAAAAAAA
```

2) Entrez Gene ID 3326 = HSP90AB1 heat shock protein 90kDa alpha (cytosolic), class B member 1 = AY359878

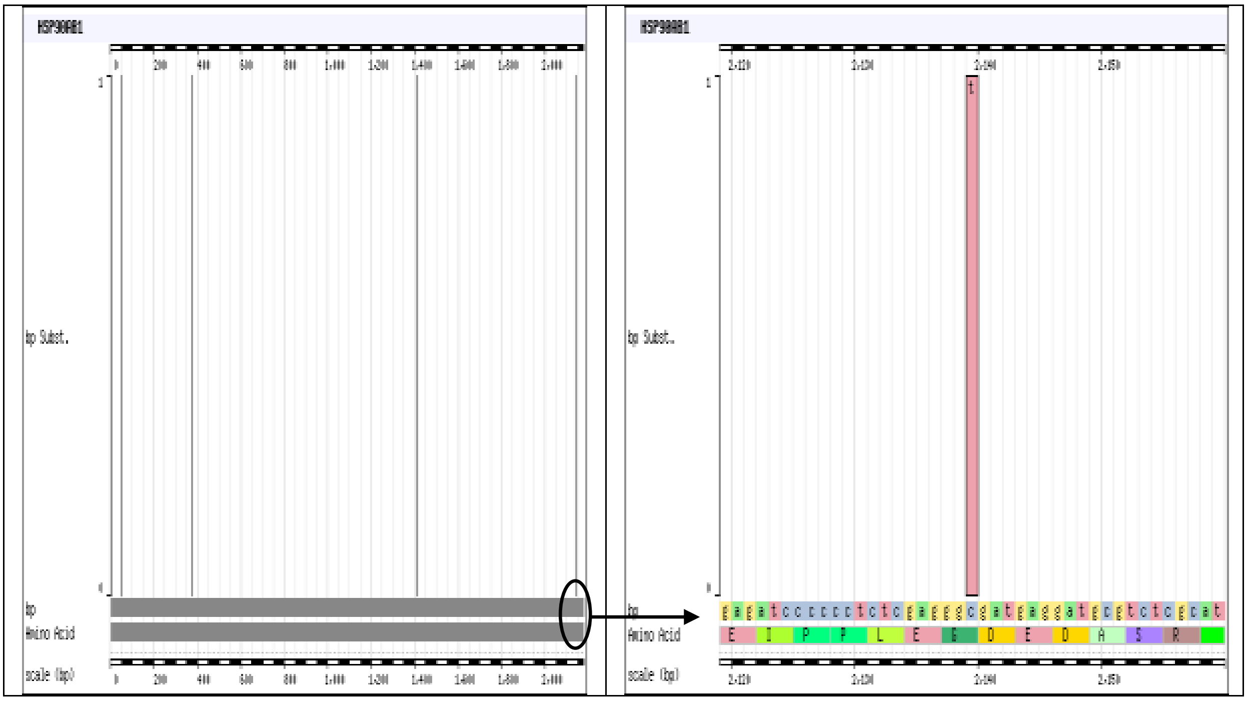

>gi|34304589|gb|AY359878.1| Homo sapiens heat shock 90kDa protein 1 beta (HSPCB) mRNA, complete cds

AGCTCTCTCGAGTCACTCCGGCGCAGTGTTGGGACTGTCTGGGTATCGGAAAGCAAGCCTACGTTGCTCA
CTATTACGTATAATCCTTTTCTTTTCAAGATTTTTATTTTAGATGCCTGAGGAAGTGCACCATGGAGAGG
AGGAGGTGGAGACTTTTGCCTTTCAGGCAGAAATTGCCCAACTCATGTCCCTCATCATCAATACCTTCTA
TTCCAACAAGGAGATTTTCCTTCGGGAGTTGATCTCTAATGCTTCTGATGCCTTGGACAAGATTCGCTAT
GAGAGCCTGACAGACCCTTCGAAGTTGGACAGTGGTAAAGAGCTGAAAATTGACATCATCCCCAACCCTC
AGGAACGTACCCTGACTTTGGTAGACACAGGCATTGGCATGACCAAAGCTGATCTCATAAATAATTTGGG
AACCATTGCCAAGTCTGGTACTAAAGCATTCATGGAGGCTCTTCAGGCTGGTGCAGACATCTCCATGATT
GGGCAGTTTGGTGTTGGCTTTTATTCTGCCTACTTGGTGGCAGAGAAAGTGGTTGTGATCACAAAGCACA
ACGATGATGAACAGTATGCTTGGGAGTCTTCTGCTGGAGGTTCCTTCACTGTGCGTGCTGACCATGGTGA
GCCCATTGGCAGGGGTACCAAAGTGATCCTCCATCTTAAAGAAGATCAGACAGAGTACCTAGAAGAGAGG
CGGGTCAAAGAAGTAGTGAAGAAGCATTCTCAGTTCATAGGCTATCCCATCACCCTTTATTTGGAGAAGG
AACGAGAGAAGGAAATTAGTGATGATGAGGCAGAGGAAGAGAAAGGTGAGAAAGAAGAGGAAGATAAAGA
TGATGAAGAAAAACCCAAGATCGAAGATGTGGGTTCAGATGAGGAGGATGACAGCGGTAAGGATAAGAAG
AAGAAAACTAAGAAGATCAAAGAGAAATACATTGATCAGGAAGAACTAAACAAGACCAAGCCTATTTGGA
CCAGAAACCCTGATGACATCACCCAAGAGGAGTATGGAGAATTCTACAAGAGCCTCACTAATGACTGGGA
AGACCACTTGGCAGTCAAGCACTTTTCTGTAGAAGGTCAGTTGGAATTCAGGGCATTGCTATTTATTCCT
CGTCGGGCTCCCTTTGACCTTTTTGAGAACAAGAAGAAAAAGAACAACATCAAACTCTATGTCCGCCGTG
TGTTCATCATGGACAGCTGTGATGAGTTGATACCAGAGTATCTCAATTTTATCCGTGGTGTGGTTGACTC
TGAGGATCTGCCCCTGAACATCTCCCGAGAAATGCTCCAGCAGAGCAAAATCTTGAAAGTCATTCGCAAA
AACATTGTTAAGAAGTGCCTTGAGCTCTTCTCTGAGCTGGCAGAAGACAAGGAGAATTACAAGAAATTCT
ATGAGGCATTCTCTAAAAATCTCAAGCTTGGAATCCACGAAGACTCCACTAACCGCCGCCGCCTGTCTGA
GCTGCTGCGCTATCATACCTCCCAGTCTGGAGATGAGATGACATCTCTGTCAGAGTATGTTTCTCGCATG
AAGGAGACACAGAAGTCCATCTATTACATCACTGGTGAGAGCAAAGAGCAGGTGGCCAACTCAGCTTTTG
TGGAGCGAGTGCGGAAACGGGGCTTCGAGGTGGTATATATGACCGAGCCCATTGACGAGTACTGTGTGCA
GCAGCTCAAGGAATTTGATGGGAAGAGCCTGGTCTCAGTTACCAAGGAGGGTCTGGAGCTGCCTGAGGAT
GAGGAGGAGAAGAAGAAGATGGAAGAGAGCAAGGCAAAGTTTGAGAACCTCTGCAAGCTCATGAAAGAAA
TCTTAGATAAGAAGGTTGAGAAGGTGACAATCTCCAATAGACTTGTGTCTTCACCTTGCTGCATTGTGAC
CAGCACCTACGGCTGGACAGCCAATATGGAGCGGATCATGAAAGCCCAGGCACTTCGGGACAACTCCACC
ATGGGCTATATGATGGCCAAAAAGCACCTGGAGATCAACCCTGACCACCCCATTGTGGAGACGCTGCGGC
AGAAGGCTGAGGCCGACAAGAATGATAAGGCAGTTAAGGACCTGGTGGTGCTGCTGTTTGAAACCGCCCT
GCTATCTTCTGGCTTTTCCCTTGAGGATCCCCAGACCCACTCCAACCGCATCTATCGCATGATCAAGCTA
GGTCTAGGTATTGATGAAGATGAAGTGGCAGCAGAGGAACCCAATGCTGCAGTTCCTGATGAGATCCCCC
CTCTCGAGGG**C**GATGAGGATGCGTCTCGCATGGAAGAAGTCGATTAGGTTAGGAGTTCATAGTTGGAAAA
CTTGTGCCCTTGTATAGTGTCCC**CATGGGCTCCCACTGCAGCCT**CGAGTGCCCCTGTCCCACCTGGCTCC
CCCTGCTGGTGTCTAGTGTTTTTTTCCCTCTCCTGTCCTTGTGTTGAAGGCAGTAAACTAAGGGTGTCAA

GCCCCATTCCCTCTCTACTCTTGACAGCAGGATTGGATGTTGTGTATTGTGGTTTATTTTATTTTCTTCA
TTTTGTTCTGAAATTAAAGTATGCAAAATAAAGAATATGCCGTTTTTATACGAAAAAAAAAAAAAAAAAA
AAAAAAAAA

3)Entrez Gene ID 7913 = DEK oncogene = BC035259

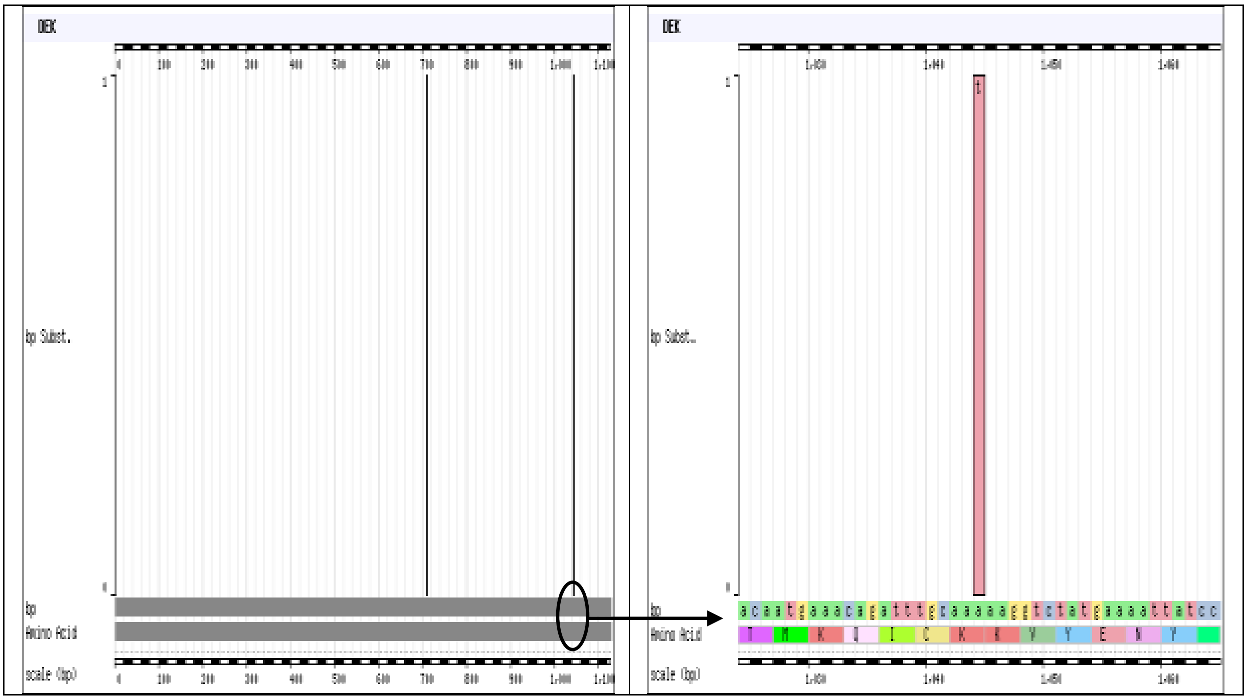

>gi|23273865|gb|BC035259.1| Homo sapiens DEK oncogene, mRNA (cDNA clone MGC:29866 IMAGE:5122743), complete cds
TTTTGAAAATCTTGTTGATTCTGGGGAGCCGAGCGCGCGGCGCGAGCGTCACGCCAGACAGCGGCCCGCG
CGCCTTCTCCTCGGCGTCGGCCGCCGCCGCCTCCCAGAACCTCCTCGTGCCCTCGCGTGCCAGGCCCGCG
GCGGCCGAAATCCGCGGTTCACAGCATGTCCGCCTCGGCCCCTGCTGCGGAGGGGGAGGGAACCCCCACC
CAGCCCGCGTCCGAGAAAGAACCCGAAATGCCCGGTCCCAGAGAGGAGAGCGAGGAGGAAGAGGACGAGG
ACGACGAGGAGGAGGAGGAGGAGGAAAAAGAAAAGAGTCTCATCGTGGAAGGCAAGAGGGAAAAGAAAAA
AGTAGAGAGGTTGACAATGCAAGTCTCTTCCTTACAGAGAGAGCCATTTACAATTGCACAAGGAAAGGGG
CAGAAACTTTGTGAAATTGAGAGGATACATTTTTTTCTAAGTAAGAAGAAAACCGATGAACTTAGAAATC
TACACAAACTGCTTTACAACAGGCCAGGCACTGTGTCCTCATTAAAGAAGAATGTGGGTCAGTTCAGTGG
CTTTCCATTTGAAAAAGGAAGTGTCCAATATAAAAAGAAGGAAGAAATGTTGAAAAAATTTAGAAATGCC
ATGTTAAAGAGCATCTGTGAGGTTCTTGATTTGGAGAGATCAGGTGTAAATAGTGAACTAGTGAAGAGGA
TCTTGAATTTCTTAATGCATCCAAAGCCTTCTGGCAAACCATTGCCGAAATCTAAAAAAACTTGTAGCAA
AGGCAGTAAAAAGGAACGGAACAGTTCTGGAATGGCAAGGAAGGCTAAGCGAACCAAATGTCCTGAAATT
CTGTCAGATGAATCTAGTAGTGATGAAGATGAAAAGAAAAACAAGGAAGAGTCTTCAGATGATGAAGATA
AAGAAAGTGAAGAGGAGCCACCAAAAAAGACAGCCAAAAGAGAAAAACCTAAACAGAAAGCTACTTCTAA
AAGTAAAAAATCTGTGAAAAGTGCCAATGTTAAGAAAGCAGATAGCAGCACCACCAAGAAGAATCAAAAC
AGTTCCAAAAAAGAAAGTGAGTCTGAGGATAGTTCAGATGATGAACCTTTAATTAAAAAGTTGAAGAAAC
CCCCTACAGATGAAGAGTTAAAGGAAACAATAAAGAAATTACTGGCCAGTGCTAACTTGGAAGAAGTCAC
AATGAAACAGATTTGCAA**A**AAGGTCTATGAAAATTATCCTACTTATGATTTAACTGAAAGAAAAGATTTC
ATAAAAACAACTGTAAAAGAGCTAATTTCTTGAGATAGAGGACAGAGAAGATGACTCGTTCCCATAGATT
TGAAGATCTGATTTATACCATTATACCAGCAAAGAGAATGTATTTCCTTTTCTAAATCCTTGTTAAGCAA
CGTTAGTAGAACTTACTGCTGACCTTTTTATCTTGAGTGTTATGTGAATTTGAGTTTGCTGTTTTAAATT
GCATTTCTATGCCATTTTTAGTTTAAAATCTTGCATGGCATTAATTGTTCCTTGCTTTTATAGTTGTATT
TTGTACATTTTGGATTTCTTTATATAAGGTCATAGATTCTTGAGCTGTTGTGGTTTTTAGTGCACTTAAT
ATTAGCTTGCTTAAGGCATACTTTTAATCAAGTAGAACAAAAACTATTATCACCAGGATTTATACATACA
GAGATTGTAGTATTTAGTATATGAAATATTTTGAATACACATCTCTGTCAGTGTGAAAATTCAGCGGCAG
TGTGTCCATCATATTAAAAATATACAAGCTACAGTTGTCCAGATCACTGAATTGGAACTTTTCTCCTGCA

TGTGTATATATGTCAAATTGTCAGCATGACAAAAGTGACAGATGTTATTTTTGTATTTTTAAAAAACAAT
TGGTTGTATATAAAGTTTTTTTATTTCTTTTGTGCAGATCACTTTTTAAACTCACATAGGTAGGTATCTT
TATAGTTGTAGACTATGGAATGTCAGTGTTCAGCCAAACAGTATGATGGAACAGTGAAAGTCAATTCAGT
GATGGCAACACTGAAGGAACAGTTACCCTGCTTTGCCTCGAAAGTGTCATCAATTTGTAATTTTAGTATT
AACTCTGTAAAAGTGTCTGTAGGTACGTTTTATATTATATAAGGACAGACCAAAAATCAACCTATCAAAG
CTTCAAAAACTTTGGGAAAGGGTGGGATTAAGTACAAGCACATTTGGCTTACAGTAAATGAACTGATTTT
TATTAACTGCTTTTGCCCATATAAAATGCTGATATTTACTGGAAACCTAGCCAGCTTCACGATTATGACT
AAAGTACCAGATTATAATGCCAGAATATAATGTGCAGGCAATCGTGGATGTCTCTGACAAAGTGTGTCTC
AAAAATAATATACTTTTACATTAAAGAAATTTAATGTTTCTCTGGAGTTGGGGCTCTTGGCTTTCAGAGT
TTGGTTAATCAGTGTTGATTCTAGATGATCAACATAATGGACCACTCCTGAATGAGACTTAATTTTGTCT
TTCAAATTTACTGTCTTAAATCAGTTTATTAAATCTGAATTTTAAAA**CATGCTGTTTATGACACAATG**AC
ACATTTGTTGCACCAATTAAGTGTTGAAAAATATCTTTGCATCATAGAACAGAAATATATAAAAATATAT
GTTGAATGTTAACAGGTATTTTCACAGGTTTGTTTCTTGATAGTTACTCAGACACTAGGGAAAGGTAAAT
ACAAGTGAACAAAATAAGCAACTAAATGAGACCTAATAATTGGCCTTCGATTTTAAATATTTGTTCTTAT
AAACCTTGTCAATAAAAATAAATCTAAATCAAAAAAAAAAAAAAAAAAAAAAAAAAA

4)Entrez Gene ID 4478 = MSN, Moesin = M69066

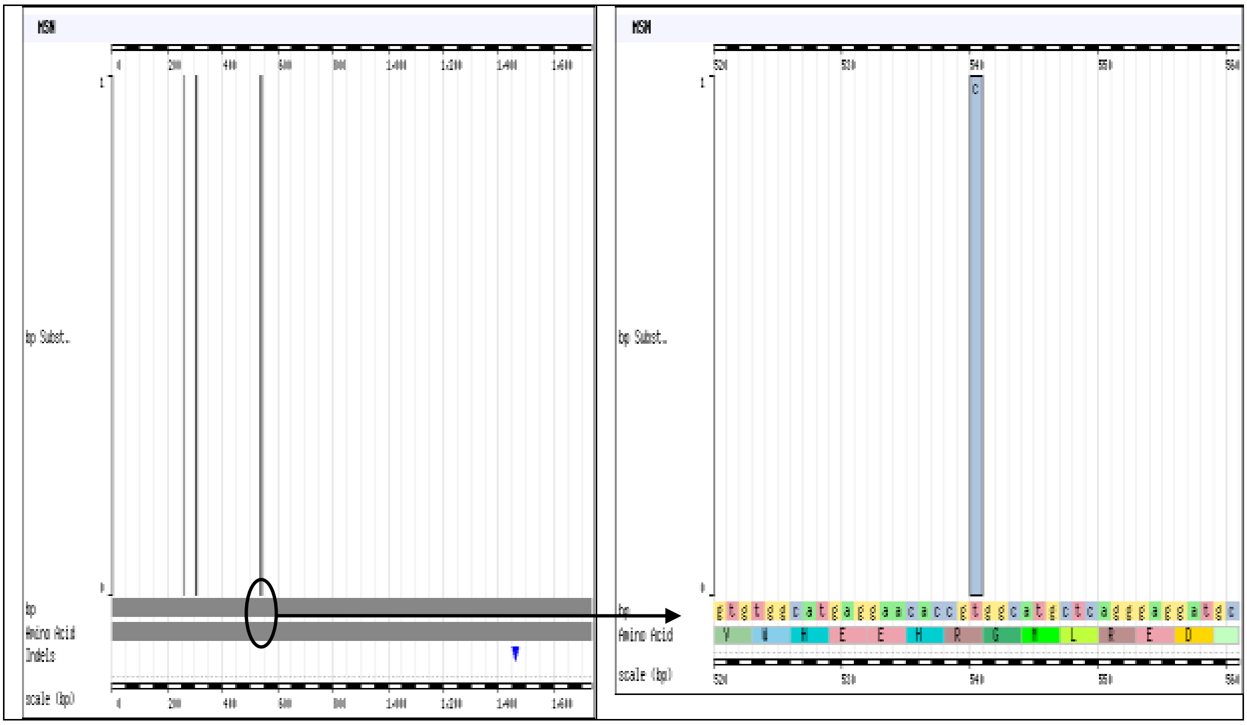

>gi|188625|gb|M69066.1|HUMMOESIN Human moesin mRNA, complete cds
GGCACGAGGCCAGCCGAATCCAAGCCGTGTGTACTGCGTGCTCAGCACTGCCCGACAGTCCTAGCTAAAC
TTCGCCAACTCCGCTGCCTTTGCCGCCACCATGCCCAAAACGATCAGTGTGCGTGTGACCACCATGGATG
CAGAGCTGGAGTTTGCCATCCAGCCCAACACCACCGGGAAGCAGCTATTTGACCAGGTGGTGAAAACTAT
TGGCTTGAGGGAAGTTTGGTTCTTTGGTCTGCAGTACCAGGACACTAAAGGTTTCTCCACCTGGCTGAAA
CTCAATAAGAAGGTGACTGCCCAGGATGTGCGGAAGGAAAGCCCCCTGCTCTTTAAGTTCCGTGCCAAGT
TCTACCCTGAGGATGTGTCCGAGGAATTGATTCAGGACATCACTCAGCGCCTGTTCTTTCTGCAAGTGAA
AGAGGGCATTCTCAATGATGATATTTACTGCCCGCCTGAGACCGCTGTGCTGCTGGCCTCGTATGCTGTC
CAGTCTAAGTATGGCGACTTCAATAAGGAAGTGCATAAGTCTGGCTACCTGGCCGGAGACAAGTTGCTCC
CGCAGAGAGTCCTGGAACAGCACAAACTCAACAAGGACCAGTGGGAGGAGCGGATCCAGGTGTGGCATGA
GGAACACCG**T**GGCATGCTCAGGGAGGATGCTGTCCTGGAATATCTGAAGATTGCTCAAGATCTGGAGATG
TATGGTGTGAACTACTTCAGCATCAAGAACAAGAAAGGCTCAGAGCTGTGGCTGGGGGTGGATGCCCTGG
GTCTCAACATCTATGAGCAGAATGACAGACTAACTCCCAAGATAGGCTTCCCCTGGAGTGAAATCAGGAA
CATCTCTTTCAATGATAAGAAATTTGTCATCAAGCCCATTGACAAAAAAGCCCCGGACTTCGTCTTCTAT
GCTCCCCGGCTGCGGATTAACAAGCGGATCTTGGCCTTGTGCATGGGGAACCATGAACTATACATGCGCC
GTCGCAAGCCTGATACCATTGAGGTGCAGCAGATGAAGGCACAGGCCCGGGAGGAGAAGCACCAGAAGCA

GATGGAGCGTGCTATGCTGGAAAATGAGAAGAAGAAGCGTGAAATGGCAGAGAAGGAGAAAGAGAAGATT
GAACGGGAGAAGGAGGAGCTGATGGAGAGGCTGAAGCAGATCGAGGAACAGACTAAGAAGGCTCAGCAAG
AACTGGAAGAACAGACCCGTAGGGCTCTGGAACTTGAGCAGGAACGGAAGCGTGCCCAGAGCGAGGCTGA
AAAGCTGGCCAAGGAGCGTCAAGAAGCTGAAGAGGCCAAGGAGGCCTTGCTGCAGGCCTCCCGGGACCAG
AAAAAGACTCAGGAACAGCTGGCCTTGGAAATGGCAGAGCTGACAGCTCGAATCTCCCAGCTGGAGATGG
CCCGACAGAAGAAGGAGAGTGAGGCTGTGGAGTGGCAGCAGAAGGCCCAGATGGTACAGGAAGACTTGGA
GAAGACCCGTGCTGAGCTGAAGACTGCCATGAGTACACCTCATGTGGCAGAGCCTGCTGAGAATGAGCAG
GATGAGCAGGATGAGAATGGGGCAGAGGCTAGTGCTGACCTACGGGCTGATGCTATGGCCAAGGACCGCA
GTGAGGAGGAACGTACCACTGAGGCAGAGAAGAATGAGCGTGTGCAGAAGCACCTGAAGGCCCTCACTTC
GGAGCTGGCCAATGCCAGAGATGAGTCCAAGAAGACTGCCAATGACATGATCCATGCTGAGAACATGCGA
CTGGGCCGAGACAAATACAAGACCCTGCGCCAGATCCGGCAGGGCAACACCAAGCAGCGCATTGACGAAT
TTGAGTCTATGTAATGGGCACCCAGCCTCTAGGGACCCCTCCTCCCTTTTTCCTTGTCCCCACACTCCTA
CACCTAACTCACCTAACTCATACTGTGCTGGAGCCACTAACTAGAGCAGCCCTGGAGTCATGCCAAGCAT
TTAATGTAGCCATGGGACCAAACCTAGCCCCTTAGCCCCCACCCACTTCCCTGGGCAAATGAATGGCTCA
CTATGGTGCCAATGGAACCTCCTTTCTCTTCTCTGTTCCATTGAATCTGTATGGCTAGAATATCCTACTT
CTCCAGCCTAGAGGTACTTTCCACTTGATTTTGCAAATGCCCTTACACTTACTGTTGTCCTATGGGAGTC
AAGTGTGGAGTAGGTTGGAAGCTAGCTCCCCTCCTCTCCCCTCCACTGTCTTCTTCAGGTCCTGAGATTA
CACGGTGGAGTGTATGCGGTCTAGGAATGAGACAGGACCTAGATATCTTCTCCAGGGATGTCAACTGACC
TAAAATTTGCCCTCCCATCCCGTTTAGAGTTATTTAGGCTTTGTAACGATTGGGGGAATAAAAAGATGTT
CAGTCATTTTTGTTTCTACCTCCCAGATCGGATCTGTTGCAAACTCAGCCTCAATAAGCCTTGTCGTTGA
CTTTAGGGACTCAATTTCTCCCCAGGGTGGATGGGGGAAATGGTGCCTTCAAGACCTTCACCAAACATAC
TAGAAGGGCATTGGCCATTCTATTGTGGCAAGGCTGAGTAGAAGATCCTACCCCAATTCCTTGTAGGAGT
ATAGGCCGGTCTAAAGTGAGCTCTATGGGCAGATCTACCCCTTACTTATTATTCCAGATCTGCAGTCACT
TCGTGGGATCTGCCCCTCCCTGCTTCAATACCCAAATCCTCTCCAGCTATAACAGTAGGGATGAGTACCC
AAAAGCTCAGCCAGCCCCATCAGGACTCTTGTGAAAAGAGAGGATATGTTCACACCTAGCGTCAGTATTT
TCCCTGCTAGGGGTTTTAGGTCTCTTCCCCTCTCAGAGCTACTTGGGCCATAGCTCCTGCTCCACAGCCA
TCCCAGCCTTGGCATCTAGAGCTTGATGCCAGTAGGCTCAACTAGGGAGTGAGTGCAAAAAGCTGAGTAT
GGTGAGAGAAGCCTGTGCCCTGATCCAAGTTTACTCAACCCTCTCAGGTGACCAAAATCCCCTTCTCATC
ACTCCCCTCAAAGAGGTGACTGGGCCCTGCCTCTGTTTGACAAACCTCTAACCCAGGTCTTGACACCAGC
TGTTCTGTCCCTTGGAGCTGTAAACCAGAGAGCTGCTGGGGGATTCTGGCCTAGTCCCTTCCACACCCCC
ACCCCTTGCTCTCAACCCAGGAGCATCCACCTCCTTCTCTGTCTCATGTGTGCTCTTCTTCTTTCTACAG
TATTATGTACTCTACTGATATCTAAATATTGATTTCTGCCTTCCTTGCTAATGCACCATTAGAAGATATT
AGTCTTGGGGCAGGATGATTTTGGCCTCATTACTTTACCACCCCCACACCTGGAAAGCATATACTATATT
ACAAAATGACATTTTGCCAAAATTATTAATATAAGAAGCTTTCAGTATTAGTGATGTCATCTGTCACTAT
AGGTCATACAATCCATTCTTAAAGTACTTGTTATTTGTTTTTATTATTACTGTTTGTCTTCTCCCCAGGG
TTCAGTCCCTCAAGGGGCCATCCTGTCCCACCATGCAGTGCCCCCTAGCTTAGAGCCTCCCTCAATTCCC
CCTGGCCACCACCCCCCACTCTGTGCCTGACCTTGAGGAGTCTTGTGTGCATTGCTGTGAATTAGCTCAC
TTGGTGATATGTCCTATATTGGCTAAATTGAAACCTGGAATTGTGGGGCAATCTATTAATAGCTGCCTTA
AAGTCAGTAACTTACCCTTAGGGAGGCTGGGGGAAAAGGTTAGATTTTGTATTCAGGGGTTTTTTGTGTA
CTTTTTGGGTTTTTAAAAAATTGTTTTTGGAGGGGTTTATGCTCAATC**CATGTTCTATTTCAGTGCCAA**T
AAAATTTAGGTGACTTCAAAAAAAAAAAA

5) Entrez Gene ID 833 = CARS, cysteinyl-tRNA synthetase = BC002880

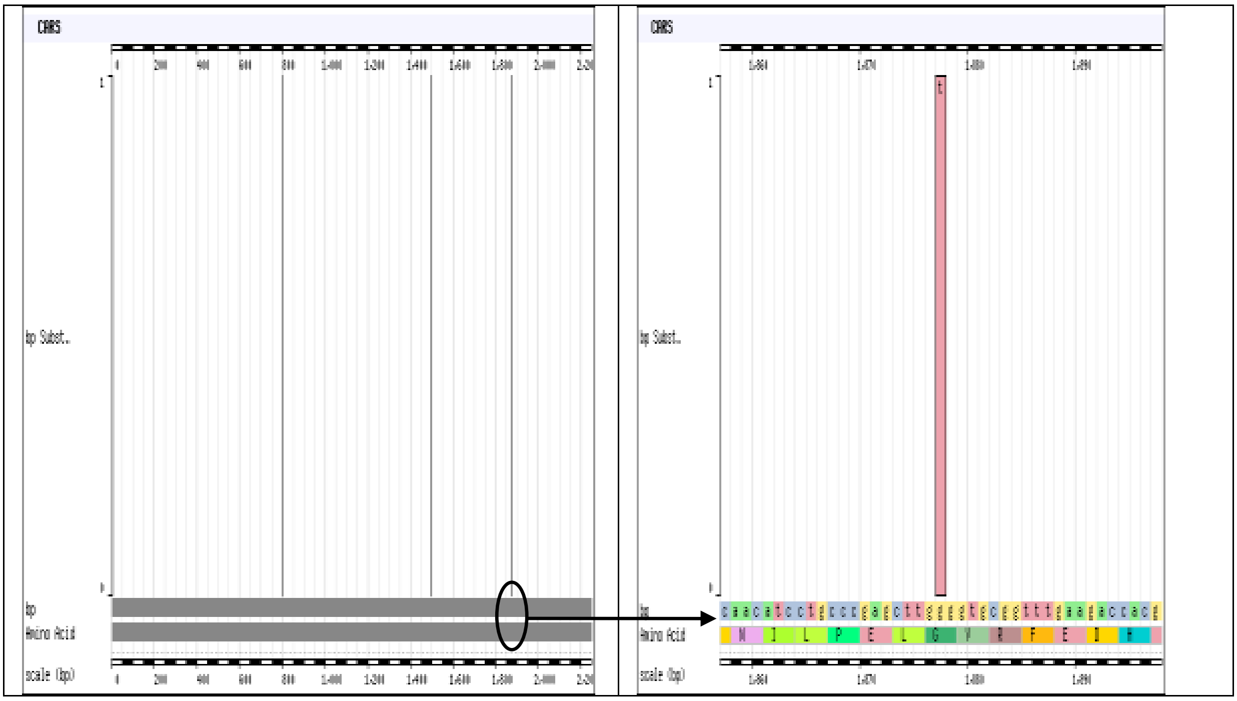

>gi|33877280|gb|BC002880.2| Homo sapiens cysteinyl-tRNA synthetase, mRNA (cDNA clone MGC:11246 IMAGE:3940167), complete cds

CCGGGGCGGCGGTTGCATCAGATTCTAGGAAGTGTCTGTAGCCGCAGCTGCGGGTCCGGGATTCCCAGCC
ATGGCAGATTCCTCCGGGCAGCAGGGCAAAGGCCGGCGTGTGCAGCCCCAGTGGTCCCCTCCTGCTGGGA
CCCAGCCATGCAGACTCCACCTTTACAACAGCCTCACCAGGAACAAGGAAGTGTTCATACCTCAAGATGG
GAAAAAGGTGACGTGGTATTGCTGTGGGCCAACCGTCTATGACGCATCTCACATGGGGCACGCCAGGTCC
TACATCTCTTTTGATATCTTGAGAAGAGTGTTGAAGGATTACTTCAAATTTGATGTCTTTTATTGCATGA
ACATTACGGATATTGATGACAAGATCATCAAGAGGGCCCGGCAGAACCACCTGTTCGAGCAGTATCGGGA
GAAGAGGCCTGAAGCGGCACAGCTCTTGGAGGATGTTCAGGCCGCCCTGAAGCCATTTTCAGTAAAATTA
AATGAGACCACGGATCCCGATAAAAAGCAGATGCTCGAACGGATTCAGCACGCAGTGCAGCTTGCCACAG
AGCCACTTGAGAAAGCTGTGCAGTCCAGACTCACGGGAGAGGAAGTCAACAGCTGTGTGGAGGTGTTGCT
GGAAGAAGCCAAGGATTTGCTCTCTGACTGGCTGGATTCTACACTTGGCTGTGATGTCACTGACAATTCC
ATCTTCTCCAAGCTGCCCAAGTTCTGGGAGGGGGACTTCCACAGAGACATGGAAGCTCTGAATGTTCTCC
CTCCAGATGTCTTAACCCGGGTTAGTGAGTATGTGCCAGAAATTGTGAACTTTGTCCAGAAGATTGTGGA
CAACGGTTACGGCTATGTCTCCAATGGGTCTGTCTACTTTGATACAGCGAAGTTTGCTTCTAGCGAGAAG
CACTCCTATGGGAAGCTGGTGCCTGAGGCCGTTGGAGATCAGAAAGCCCTTCAAGAAGGGGAAGGTGACC
TGAGCATCTCTGCAGACCGCCTGAGTGAGAAGCGCTCTCCCAACGACTTTGCCTTATGGAAGGCCTCTAA
GCCCGGAGAACCGTCCTGGCCGTGCCCTTGGGGAAAGGGTCGTCCGGGCTGGCATATCGAGTGCTCGGCC
ATGGCAGGCACCCTCCTAGGGGCTTCGATGGACATTCACGGAGGTGGGTTCGACCTCCGGTTCCCCCACC
ATGACAATGAGCTGGCACAGTCGGAGGCCTACTTTGAAAACGACTGCTGGGTCAGGTACTTCCTGCACAC
AGGCCACCTGACCATTGCAGGCTGCAAAATGTCAAAGTCACTAAAAAACTTCATCACCATTAAAGATGCC
TTGAAAAAGCACTCAGCACGGCAGTTGCGGCTGGCCTTCCTCATGCACTCGTGGAAGGACACCCTGGACT
ACTCCAGCAACACCATGGAGTCAGCGCTTCAATATGAGAAGTTCTTGAATGAGTTTTTCTTAAATGTGAA
AGATATCCTTCGCGCTCCTGTTGACATCACTGGTCAGTTTGAGAAGTGGGGAGAAGAAGAAGCAGAACTG
AATAAGAACTTTTATGACAAGAAGACAGCAATTCACAAAGCCCTCTGTGACAATGTTGACACCCGCACCG
TCATGGAAGAGATGCGGGCCTTGGTCAGTCAGTGCAACCTCTATATGGCAGCCCGGAAAGCCGTGAGGAA
GAGGCCCAACCAGGCTCTGCTGGAGAACATCGCCCTGTACCTCACCCATATGCTGAAGATCTTTGGGGCC
GTAGAAGAGGACAGCTCCCTGGGATTCCCGGTCGGAGGGCCTGGAACCAGCCTCAGTCTCGAGGCCACAG
TCATGCCCTACCTTCAGGTGTTATCAGAATTCCGAGAAGGAGTGCGGAAGATTGCCCGAGAGCAAAAAGT
CCCTGAGATTCTGCAGCTCAGCGATGCCCTGCGGGACAACATCCTGCCCGAGCTTG**G**GGTGCGGTTTGAA
GACCACGAAGGACTGCCCACAGTGGTGAAACTGGTAGACAGAAACACCTTATTAAAAGAGAGAGAAGAAA
AGAGACGGGTTGAAGAGGAGAAGAGGAAGAAGAAAGAGGAGGCGGCCCGGAGGAAACAGGAACAAGAAGC
AGCAAAGCTGGCCAAGATGAAGATTCCCCCCAGTGAGATGTTCTTGTCAGAAACCGACAAATACTCCAAG
TTTGATGAAAATGGTCTGCCCACACATGACATGGAGGGCAAAGAGCTCAGCAAAGGGCAAGCCAAGAAGC
TGAAGAAGCTCTTCGAGGCTCAGGAGAAGCTCTACAAGGAATATCTGCAGATGGCCCAGAATGGAAGCTT
CCAGTGAGGGGGCACAGGACTGACTTTTTAAACCATTGTGGACTAGTGGCTGCTGTCTGCCTCAGTGACA
ATGTCCCAGCGCTCCTATCATGTTTACAGTCACCCTTGGGTCCTAAATTAAGAGTTGTGTT**CATGTAGGT**

TCGTGTCGTCGTTGGCTCTGAGACATTGATAATAAATTTTTCTCAACAGTGAAAAAAAAAAAAAAAAAAA
AAAA

6) Entrez Gene ID 1277 = COL1A1, collagen, type I, alpha 1 = BC036531

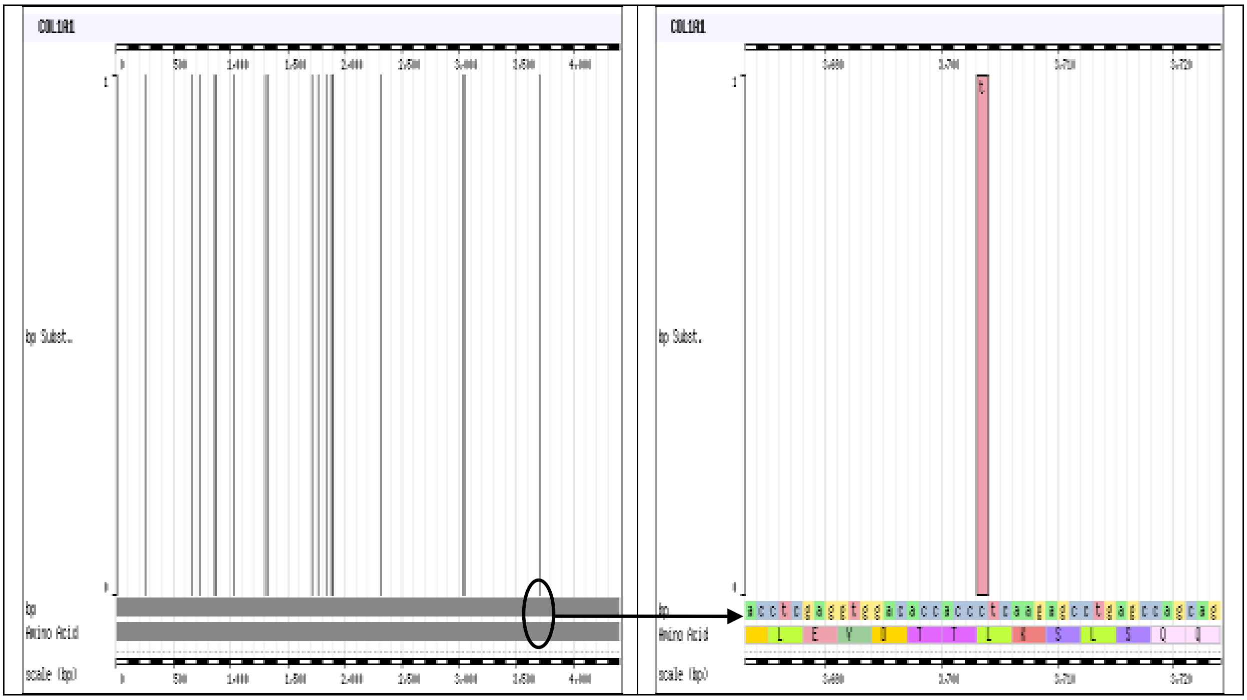

>gi|34193787|gb|BC036531.2| Homo sapiens collagen, type I, alpha 1, mRNA (cDNA clone MGC:33668 IMAGE:5264710), complete cds
AGACGGGAGTTTCTCCTCGGGGTCGGAGCAGGAGGCACGCGGAGTGTGAGGCCACGCATGAGCGGACGCT
AACCCCCTCCCCAGCCACAAAGAGTCTACATGTCTAGGGTCTAGACATGTTCAGCTTTGTGGACCTCCGG
CTCCTGCTCCTCTTAGCGGCCACCGCCCTCCTGACGCACGGCCAAGAGGAAGGCCAAGTCGAGGGCCAAG
ACGAAGACATCCCACCAATCACCTGCGTACAGAACGGCCTCAGGTACCATGACCGAGACGTGTGGAAACC
CGAGCCCTGCCGGATCTGCGTCTGCGACAACGGCAAGGTGTTGTGCGATGACGTGATCTGTGACGAGACC
AAGAACTGCCCCGGCGCCGAAGTCCCCGAGGGCGAGTGCTGTCCCGTCTGCCCCGACGGCTCAGAGTCAC
CCACCGACCAAGAAACCACCGGCGTCGAGGGACCCAAGGGAGACACTGGCCCCCGAGGCCCAAGGGGACC
CGCAGGCCCCCCTGGCCGAGATGGCATCCCTGGACAGCCTGGACTTCCCGGACCCCCCGGACCCCCCGGA
CCTCCCGGACCCCCTGGCCTCGGAGGAAACTTTGCTCCCCAGCTGTCTTATGGCTATGATGAGAAATCAA
CCGGAGGAATTTCCGTGCCTGGCCCCATGGGTCCCTCTGGTCCTCGTGGTCTCCCTGGCCCCCCTGGTGC
ACCTGGTCCCCAAGGCTTCCAAGGTCCCCCTGGTGAGCCTGGCGAGCCTGGAGCTTCAGGTCCCATGGGT
CCCCGAGGTCCCCCAGGTCCCCCTGGAAAGAATGGAGATGATGGGGAAGCTGGAAAACCTGGTCGTCCTG
GTGAGCGTGGGCCTCCTGGGCCTCAGGGTGCTCGAGGATTGCCCGGAACAGCTGGCCTCCCTGGAATGAA
GGGACACAGAGGTTTCAGTGGTTTGGATGGTGCCAAGGGAGATGCTGGTCCTGCTGGTCCTAAGGGTGAG
CCTGGCAGCCCTGGTGAAAATGGAGCTCCTGGTCAGATGGGCCCCCGTGGCCTGCCTGGTGAGAGAGGTC
GCCCTGGAGCCCCTGGCCCTGCTGGTGCTCGTGGAAATGATGGTGCTACTGGTGCTGCCGGGCCCCCTGG
TCCCACCGGCCCCGCTGGTCCTCCTGGCTTCCCTGGTGCTGTTGGTGCTAAGGGTGAAGCTGGTCCCCAA
GGGCCCCGAGGCTCTGAAGGTCCCCAGGGTGTGCGTGGTGAGCCTGGCCCCCCTGGCCCTGCTGGTGCTG
CTGGCCCTGCTGGAAACCCTGGTGCTGATGGACAGCCTGGTGCTAAAGGTGCCAATGGTGCTCCTGGTAT
TGCTGGTGCTCCTGGCTTCCCTGGTGCCCGAGGCCCCTCTGGACCCCAGGGCCCCGGCGGCCCTCCTGGT
CCCAAGGGTAACAGCGGTGAACCTGGTGCTCCTGGCAGCAAAGGAGACACTGGTGCTAAGGGAGAGCCTG
GCCCTGTTGGTGTTCAAGGACCCCCTGGCCCTGCTGGAGAGGAAGGAAAGCGAGGAGCTCGAGGTGAACC
CGGACCCACTGGCCTGCCCGGACCCCCTGGCGAGCGTGGTGGACCTGGTAGCCGTGGTTTCCCTGGCGCA
GATGGTGTTGCTGGTCCCAAGGGTCCCGCTGGTGAACGTGGTTCTCCTGGCCCTGCTGGCCCCAAAGGAT
CTCCTGGTGAAGCTGGTCGTCCCGGTGAAGCTGGTCTGCCTGGTGCCAAGGGTCTGACTGGAAGCCCTGG
CAGCCCTGGTCCTGATGGCAAAACTGGCCCCCCTGGTCCCGCCGGTCAAGATGGTCGCCCCGGACCCCCA
GGCCCACCTGGTGCCCGTGGTCAGGCTGGTGTGATGGGATTCCCTGGACCTAAAGGTGCTGCTGGAGAGC
CCGGCAAGGCTGGAGAGCGAGGTGTTCCCGGACCCCCTGGCGCTGTCGGTCCTGCTGGCAAAGATGGAGA

GGCTGGAGCTCAGGGACCCCCTGGCCCTGCTGGTCCCGCTGGCGAGAGAGGTGAACAAGGCCCTGCTGGC
TCCCCCGGATTCCAGGGTCTCCCTGGTCCTGCTGGTCCTCCAGGTGAAGCAGGCAAACCTGGTGAACAGG
GTGTTCCTGGAGACCTTGGCGCCCCTGGCCCCTCTGGAGCAAGAGGCGAGAGAGGTTTCCCTGGCGAGCG
TGGTGTGCAAGGTCCCCCTGGTCCTGCTGGTCCCCGAGGGGCCAACGGTGCTCCCGGCAACGATGGTGCT
AAGGGTGATGCTGGTGCCCCTGGAGCTCCCGGTAGCCAGGGCGCCCCTGGCCTTCAGGGAATGCCTGGTG
AACGTGGTGCAGCTGGTCTTCCAGGGCCTAAGGGTGACAGAGGTGATGCTGGTCCCAAAGGTGCTGATGG
CTCTCCTGGCAAAGATGGCGTCCGTGGTCTGACCGGCCCCATTGGTCCTCCTGGCCCTGCTGGTGCCCCT
GGTGACAAGGGTGAAAGTGGTCCCAGCGGCCCTGCTGGTCCCACTGGAGCTCGTGGTGCCCCCGGAGACC
GTGGTGAGCCTGGTCCCCCCGGCCCTGCTGGCTTTGCTGGCCCCCCTGGTGCTGACGGCCAACCTGGTGC
TAAAGGCGAACCTGGTGATGCTGGTGCTAAAGGCGATGCTGGTCCCCCTGGCCCTGCCGGACCCGCTGGA
CCCCCTGGCCCCATTGGTAATGTTGGTGCTCCTGGAGCCAAAGGTGCTCGCGGCAGCGCTGGTCCCCCTG
GTGCTACTGGTTTCCCTGGTGCTGCTGGCCGAGTCGGTCCTCCTGGCCCCTCTGGAAATGCTGGACCCCC
TGGCCCTCCTGGTCCTGCTGGCAAAGAAGGCGGCAAAGGTCCCCGTGGTGAGACTGGCCCTGCTGGACGT
CCTGGTGAAGTTGGTCCCCCTGGTCCCCCTGGCCCTGCTGGCGAGAAAGGATCCCCTGGTGCTGATGGTC
CTGCTGGTGCTCCTGGTACTCCCGGGCCTCAAGGTATTGCTGGACAGCGTGGTGTGGTCGGCCTGCCTGG
TCAGAGAGGAGAGAGAGGCTTCCCTGGTCTTCCTGGCCCCTCTGGTGAACCTGGCAAACAAGGTCCCTCT
GGAGCAAGTGGTGAACGTGGTCCCCCTGGTCCCATGGGCCCCCCTGGATTGGCTGGACCCCCTGGTGAAT
CTGGACGTGAGGGGGCTCCTGGTGCCGAAGGTTCCCCTGGACGAGACGGTTCTCCTGGCGCCAAGGGTGA
CCGTGGTGAGACCGGCCCCGCTGGACCCCCTGGTGCTCCTGGTGCTCCTGGTGCCCCTGGCCCCGTTGGC
CCTGCTGGCAAGAGTGGTGATCGTGGTGAGACTGGTCCTGCTGGTCCCGCCGGTCCTGTCGGCCCTGTTG
GCGCCCGTGGCCCCGCCGGACCCCAAGGCCCACGTGGTGACAAGGGTGAGACAGGCGAACAGGGCGACAG
AGGCATAAAGGGTCACCGTGGCTTCTCTGGCCTCCAGGGTCCCCCTGGCCCTCCTGGCTCTCCTGGTGAA
CAAGGTCCCTCTGGAGCCTCTGGTCCTGCTGGTCCCCGAGGTCCCCCTGGCTCTGCTGGTGCTCCTGGCA
AAGATGGACTCAACGGTCTCCCTGGCCCCATTGGGCCCCCTGGTCCTCGCGGTCGCACTGGTGATGCTGG
TCCTGTTGGTCCCCCCGGCCCTCCTGGACCTCCTGGTCCCCCTGGTCCTCCCAGCGCTGGTTTCGACTTC
AGCTTCCTGCCCCAGCCACCTCAAGAGAAGGCTCACGATGGTGGCCGCTACTACCGGGCTGATGATGCCA
ATGTGGTTCGTGACCGTGACCTCGAGGTGGACACCACC**C**TCAAGAGCCTGAGCCAGCAGATCGAGAACAT
CCGGAGCCCAGAGGGCAGCCGCAAGAACCCCGCCCGCACCTGCCGTGACCTCAAGATGTGCCACTCTGAC
TGGAAGAGTGGAGAGTACTGGATTGACCCCAACCAAGGCTGCAACCTGGATGCCATCAAAGTCTTCTGCA
ACATGGAGACTGGTGAGACCTGCGTGTACCCCACTCAGCCCAGTGTGGCCCAGAAGAACTGGTACATCAG
CAAGAACCCCAAGGACAAGAGGCATGTCTGGTTCGGCGAGAGCATGACCGATGGATTCCAGTTCGAGTAT
GGCGGCCAGGGCTCCGACCCTGCCGATGTGGCCATCCAGCTGACCTTCCTGCGCCTGATGTCCACCGAGG
CCTCCCAGAACATCACCTACCACTGCAAGAACAGCGTGGCCTACATGGACCAGCAGACTGGCAACCTCAA
GAAGGCCCTGCTCCTCCAGGGCTCCAACGAGATCGAGATCCGCGCCGAGGGCAACAGCCGCTTCACCTAC
AGCGTCACTGTCGATGGCTGCACGAGTCACACCGGAGCCTGGGGCAAGACAGTGATTGAATACAAAACCA
CCAAGACCTCCCGCCTGCGCATCATCGATGTGGCCCCCTTGGACGTTGGTGCCCCAGACCAGGAATTCGG
CTTCGACGTTGGCCATGTCTGCTTCCTGTAAACTCCCTCCATCCCAACCTGGCTCCCTCCCACCCAACCA
ACTTTCCCCCCAACCCGGAAACAGACAAGCAACCCAAACTGAACCCCCTCAAAAGCCAAAAAATGGGAGA
CAATTTCACATGGACTTTGGAAAATATTTTTTTCCTTTGCATTCATCTCTCAAACTTAGTTTTTATCTTT
GACCAACCGAA**CATGACCAAAAACCAAAAGTG**CATTCAACCTTACCAAAAAAAAAAAAAAAA

7) Entrez GeneID 1499 = CTNNB1, catenin (cadherin-associated protein), beta 1, 88kDa = BC058926

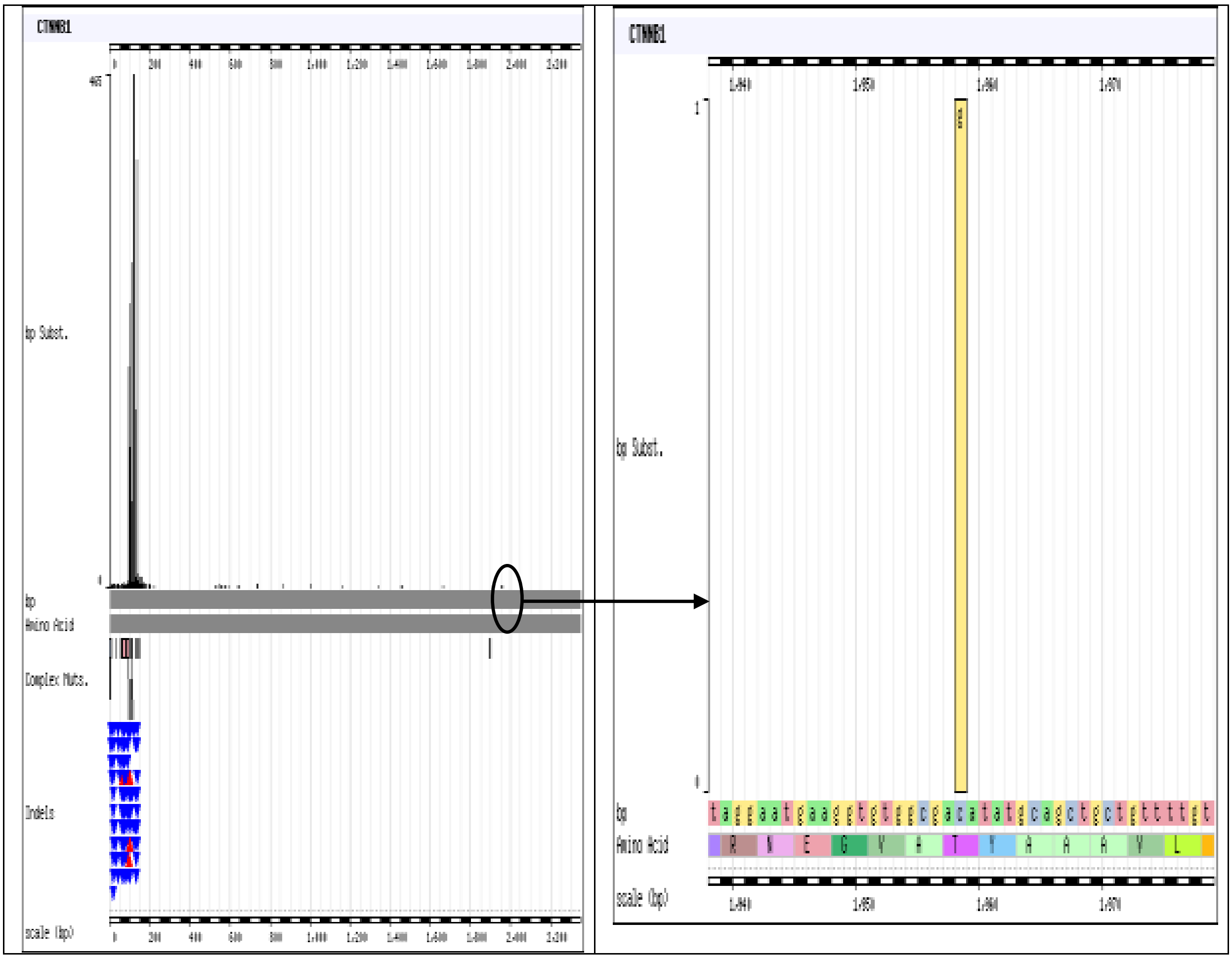

>gi|37590637|gb|BC058926.1| Homo sapiens catenin (cadherin-associated protein), beta 1, 88kDa, mRNA (cDNA clone MGC:65131 IMAGE:6151332), complete cds

CCCACGCGTCCGGGCAGCAGCGTTGGCCCGGCCCCGGGAGCGGAGAGCGAGGGGAGGCGGAGACGGAGGA
AGGTCTGAGGAGCAGCTTCAGTCCCCGCCGAGCCGCCACCGCAGGTCGAGGACGGTCGGACTCCCGCGGC
GGGAGGAGCCTGTTCCCCTGAGGGTATTTGAAGTATACCATACAACTGTTTTGAAAATCCAGCGTGGACA
ATGGCTACTCAAGCTGATTTGATGGAGTTGGACATGGCCATGGAACCAGACAGAAAAGCGGCTGTTAGTC
ACTGGCAGCAACAGTCTTACCTGGACTCTGGAATCCATTCTGGTGCCACTACCACAGCTCCTTCTCTGAG
TGGTAAAGGCAATCCTGAGGAAGAGGATGTGGATACCTCCCAAGTCCTGTATGAGTGGGAACAGGGATTT
TCTCAGTCCTTCACTCAAGAACAAGTAGCTGATATTGATGGACAGTATGCAATGACTCGAGCTCAGAGGG
TACGAGCTGCTATGTTCCCTGAGACATTAGATGAGGGCATGCAGATCCCATCTACACAGTTTGATGCTGC
TCATCCCACTAATGTCCAGCGTTTGGCTGAACCATCACAGATGCTGAAACATGCAGTTGTAAACTTGATT
AACTATCAAGATGATGCAGAACTTGCCACACGTGCAATCCCTGAACTGACAAAACTGCTAAATGACGAGG
ACCAGGTGGTGGTTAATAAGGCTGCAGTTATGGTCCATCAGCTTTCTAAAAAGGAAGCTTCCAGACACGC
TATCATGCGTTCTCCTCAGATGGTGTCTGCTATTGTACGTACCATGCAGAATACAAATGATGTAGAAACA
GCTCGTTGTACCGCTGGGACCTTGCATAACCTTTCCCATCATCGTGAGGGCTTACTGGCCATCTTTAAGT
CTGGAGGCATTCCTGCCCTGGTGAAAATGCTTGGTTCACCAGTGGATTCTGTGTTGTTTTATGCCATTAC
AACTCTCCACAACCTTTTATTACATCAAGAAGGAGCTAAAATGGCAGTGCGTTTAGCTGGTGGGCTGCAG
AAAATGGTTGCCTTGCTCAACAAAACAAATGTTAAATTCTTGGCTATTACGACAGACTGCCTTCAAATTT
TAGCTTATGGCAACCAAGAAAGCAAGCTCATCATACTGGCTAGTGGTGGACCCCAAGCTTTAGTAAATAT
AATGAGGACCTATACTTACGAAAAACTACTGTGGACCACAAGCAGAGTGCTGAAGGTGCTATCTGTCTGC
TCTAGTAATAAGCCGGCTATTGTAGAAGCTGGTGGAATGCAAGCTTTAGGACTTCACCTGACAGATCCAA
GTCAACGTCTTGTTCAGAACTGTCTTTGGACTCTCAGGAATCTTTCAGATGCTGCAACTAAACAGGAAGG
GATGGAAGGTCTCCTTGGGACTCTTGTTCAGCTTCTGGGTTCAGATGATATAAATGTGGTCACCTGTGCA
GCTGGAATTCTTTCTAACCTCACTTGCAATAATTATAAGAACAAGATGATGGTCTGCCAAGTGGGTGGTA
TAGAGGCTCTTGTGCGTACTGTCCTTCGGGCTGGTGACAGGGAAGACATCACTGAGCCTGCCATCTGTGC
TCTTCGTCATCTGACCAGCCGACACCAAGAAGCAGAGATGGCCCAGAATGCAGTTCGCCTTCACTATGGA
CTACCAGTTGTGGTTAAGCTCTTACACCCACCATCCCACTGGCCTCTGATAAAGGCTACTGTTGGATTGA

TTCGAAATCTTGCCCTTTGTCCCGCAAATCATGCACCTTTGCGTGAGCAGGGTGCCATTCCACGACTAGT
TCAGTTGCTTGTTCGTGCACATCAGGATACCCAGCGCCGTACGTCCATGGGTGGGACACAGCAGCAATTT
GTGGAGGGGGTCCGCATGGAAGAAATAGTTGAAGGTTGTACCGGAGCCCTTCACATCCTAGCTCGGGATG
TTCACAACCGAATTGTTATCAGAGGACTAAATACCATTCCATTGTTTGTGCAGCTGCTTTATTCTCCCAT
TGAAAACATCCAAAGAGTAGCTGCAGGGGTCCTCTGTGAACTTGCTCAGGACAAGGAAGCTGCAGAAGCT
ATTGAAGCTGAGGGAGCCACAGCTCCTCTGACAGAGTTACTTCACTCTAGGAATGAAGGTGTGGCGA**C**AT
ATGCAGCTGCTGTTTTGTTCCGAATGTCTGAGGACAAGCCACAAGATTACAAGAAACGGCTTTCAGTTGA
GCTGACCAGCTCTCTCTTCAGAACAGAGCCAATGGCTTGGAATGAGACTGCTGATCTTGGACTTGATATT
GGTGCCCAGGGAGAACCCCTTGGATATCGCCAGGATGATCCTAGCTATCGTTCTTTTCACTCTGGTGGAT
ATGGCCAGGATGCCTTGGGTATGGACCCCATGATGGAACATGAGATGGGTGGCCACCACCCTGGTGCTGA
CTATCCAGTTGATGGGCTGCCAGATCTGGGGCATGCCCAGGACCTCATGGATGGGCTGCCTCCAGGTGAC
AGCAATCAGCTGGCCTGGTTTGATACTGACCTGTAAATCATCCTTTAGGTAAGAAGTTTTAAAAAGCCAG
TTTGGGTAAAATACTTTTACTCTGCCTACAGAACTTCAGAAAGACTTGGTTGGTAGGGTGGGAGTGGTTT
AGGCTATTTGTAAATCTGCCACAAAAACAGGTATATACTTTGAAAGGAGATGTCTTGGAACATTGGAATG
TTCTCAGATTTCTGGTTGTTATGTGATCATGTGTGGAAGTTATTAACTTTAATGTTTTTTGCCACAGCTT
TTGCAACTTAATACTCAAATGAGTAACATTTGCTGTTTTAAACATTAATAGCAGCCTTTCTCTCTTTATA
CAGCTGTATTGTCTGAACTTGCATTGTGATTGGCCTGTAGAGTTGCTGAGAGGGCTCGAGGGGTGGGCTG
GTATCTCAGAAAGTGCCTGACACACTAACCAAGCTGAGTTTCCTATGGGAACAATTGAAGTAAACTTTTT
GTTCTGGTCCTTTTTGGTCGAGGAGTAACAATACAAATGGATTTTGGGAGTGACTCAAGAAGTGAAGAAT
GCACAAGAATGGATCACAAGATGGAATTTAGCAAACCCTAGCCTTGCTTGTTAAAATTTTTTTTTTTTTT
TTTTAAGAATATCTGTAATGGTACTGACTTTGCTTGCTTTGAAGTAGCTCTTTTTTTTTTTTTTTTTTTT
TTTTTTTTGCAGTAACTGTTTTTTAAGTCTCTCGTAGTGTTAAGTTATAGTGAATACTGCTACAGCAATT
TCTAATTTTTAAGAATTGAGTAATGGTGTAGAACACTAATTAATTCATAATCACTCTAATTAATTGTAAT
CTGAATAAAGTGTAACAATTGTGTAGCCTTTTTGTATAAAATAGACAAATAGAAAATGGTCCAATTAGTT
TCCTTTTTAATATGCTTAAAATAAGCAGGTGGATCTATTT**CATGTTTTTGATCAAAAACTA**TTTGGGATA
TGTATGGGTAGGGTAAATCAGTAAGAGGTGTTATTTGGAACCTTGTTTTGGACAGTTTACCAGTTGCCTT
TTATCCCAAAGTTGTTGTAACCTGCTGTGATACGATGCTTCAAGAGAAAATGCGGTTATAAAAAATGGTT
CAGAATTAAACTTTTAATTCATTCAAAAAAAAAAAAAAAAAAAAAAAAAAAAAAAAA

8) Entrez Gene ID 6146 = RPL22, ribosomal protein L22 = NM_000983

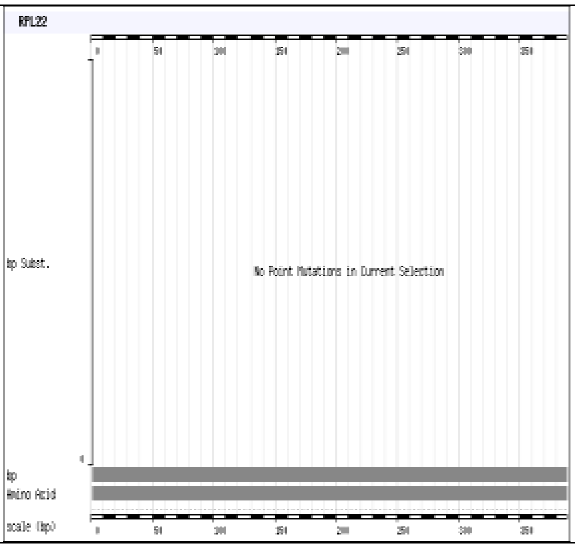

9)Entrez Gene ID 6794 = STK11, serine/threonine kinase 11 = BC007981

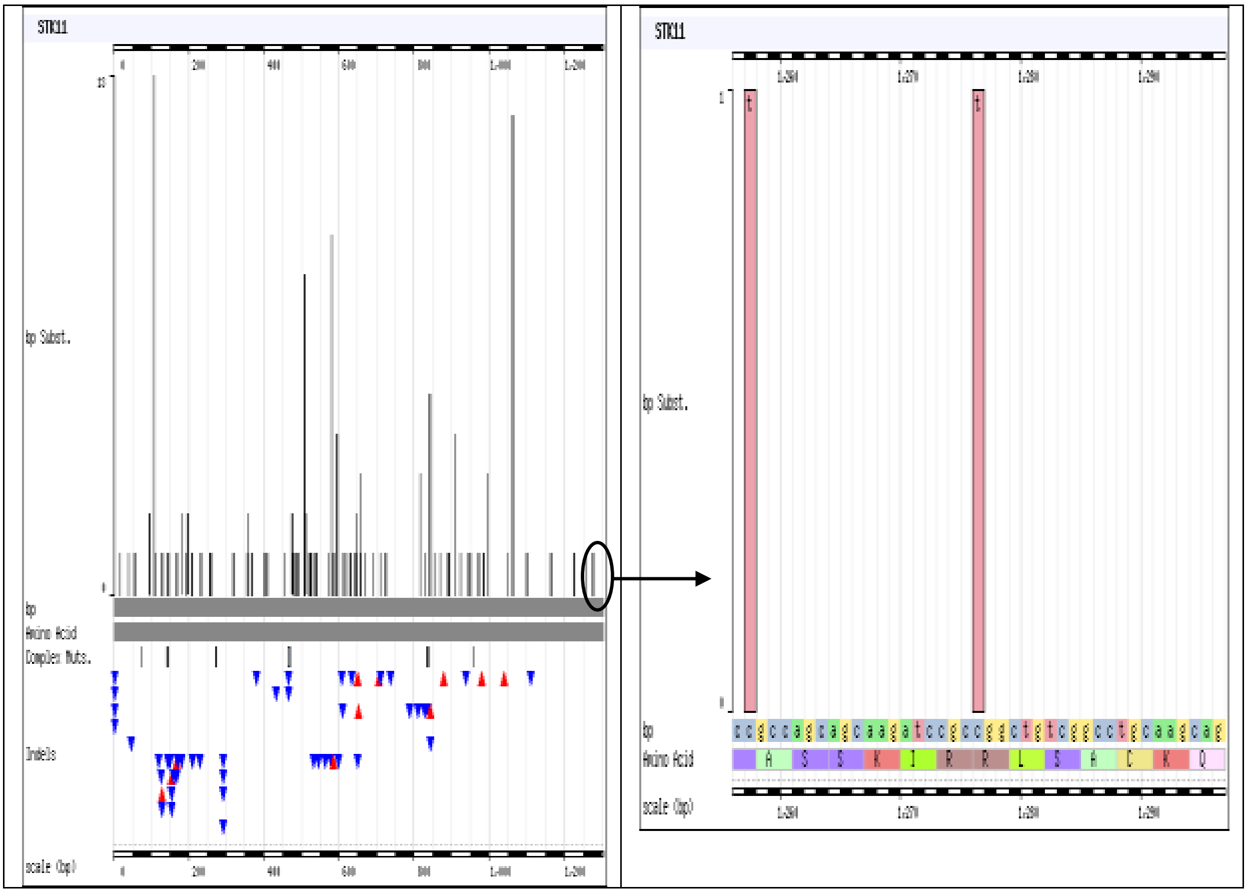

>gi|33872385|gb|BC007981.2| Homo sapiens serine/threonine kinase 11, mRNA (cDNA clone MGC:16254 IMAGE:3689780), complete cds

GGCCTGTGGGATGGGCGGCCCGGAGAAGACTGCGCTCGGCCGTGTTCATACTTGTCCGTGGGCCTGAGGT
CCCCGGAGGATGACCTAGCACTGAAAAGCCCCGGCCGGCCTCCCCAGGGTCCCCGAGGACGAAGTTGACC
CTGACCGGGCCGTCTCCCAGTTCTGAGGCCCGGGTCCCACTGGAACTCGCGTCTGAGCCGCCGTCCCGGA
CCCCCGGTGCCCGCCGGTCCGCAGACCCTGCACCGGGCTTGGACTCGCAGCCGGGACTGACGTGTAGAAC
AATCGTTTCTGTTGGAAGAAGGGTTTTTCCCTTCCTTTTGGGGTTTTTGTTGCCTTTTTTTTTTCTTTTT
TCTTTGTAAAATTTTGGAGAAGGGAAGTCGGAACACAAGGAAGGACCGCTCACCCGCGGACTCAGGGCTG
GCGGCGGGACTCCAGGACCCTGGGTCCAGCATGGAGGTGGTGGACCCGCAGCAGCTGGGCATGTTCACGG
AGGGCGAGCTGATGTCGGTGGGTATGGACACGTTCATCCACCGCATCGACTCCACCGAGGTCATCTACCA
GCCGCGCCGCAAGCGGGCCAAGCTCATCGGCAAGTACCTGATGGGGGACCTGCTGGGGGAAGGCTCTTAC
GGCAAGGTGAAGGAGGTGCTGGACTCGGAGACGCTGTGCAGGAGGGCCGTCAAGATCCTCAAGAAGAAGA
AGTTGCGAAGGATCCCCAACGGGGAGGCCAACGTGAAGAAGGAAATTCAACTACTGAGGAGGTTACGGCA
CAAAAATGTCATCCAGCTGGTGGATGTGTTATACAACGAAGAGAAGCAGAAAATGTATATGGTGATGGAG
TACTGCGTGTGTGGCATGCAGGAAATGCTGGACAGCGTGCCGGAGAAGCGTTTCCCAGTGTGCCAGGCCC
ACGGGTACTTCTGTCAGCTGATTGACGGCCTGGAGTACCTGCATAGCCAGGGCATTGTGCACAAGGACAT
CAAGCCGGGGAACCTGCTGCTCACCACCGGTGGCACCCTCAAAATCTCCGACCTGGGCGTGGCCGAGGCA
CTGCACCCGTTCGCGGCGGACGACACCTGCCGGACCAGCCAGGGCTCCCCGGCTTTCCAGCCGCCCGAGA
TTGCCAACGGCCTGGACACCTTCTCCGGCTTCAAGGTGGACATCTGGTCGGCTGGGGTCACCCTCTACAA
CATCACCACGGGTCTGTACCCCTTCGAAGGGGACAACATCTACAAGTTGTTTGAGAACATCGGGAAGGGG
AGCTACGCCATCCCGGGCGACTGTGGCCCCCCGCTCTCTGACCTGCTGAAAGGGATGCTTGAGTACGAAC
CGGCCAAGAGGTTCTCCATCCGGCAGATCCGGCAGCACAGCTGGTTCCGGAAGAAACATCCTCCGGCTGA
AGCACCAGTGCCCATCCCACCGAGCCCAGACACCAAGGACCGGTGGCGCAGCATGACTGTGGTGCCGTAC
TTGGAGGACCTGCACGGCGCGGACGAGGACGAGGACCTCTTCGACATCGAGGATGACATCATCTACACTC
AGGACTTCACGGTGCCCGGACAGGTCCCAGAAGAGGAGGCCAGTCACAATGGACAGCGCCGGGGCCTCCC
CAAGGCCGTGTGTATGAACGGCACAGAGGCGGCGCAGCTGAGCACCAAATCCAGGGCGGAGGGCCGGGCC
CCCAACCCTGCCCGCAAGGCCTGCTC**C**GCCAGCAGCAAGATCCGC**C**GGCTGTCGGCCTGCAAGCAGCAGT
GAGGCTGGCCGCCTGCAGCCCGTGTCCAGGAGCCCCGCCAGGTGCCCGCGCCAGGCCCTCAGTCTTCCTG
CCGGTTCCGCCCGCCCTCCCGGAGAGGTGGCCGCCATGCTTCTGTGCCGACCACGCCCCAGGACCTCCGG
AGCGCCCTGCAGGGCCGGGCAGGGGGACAGCAGGGACCGGGCGCAGCCCTCCCCCCTCGGCCGCCCGGCA
GTGCACGCGGCTTGTTGACTTCGCAGCCCCGGGCGGAGCCTTCCCGGGCGGGCGTGGAGGAGGGAGGCG

GCCTCCATGCACTTTATGTGGAGACTACTGGCCCCGCCCGTGGCCTCGTGCTCCGCAGGGCGCCCAGCGC
CGTCCGGCGGCCCCGCCGCAGACCAGCTGGCGGGTGTGGAGACCAGGCTCCTGACCCCGCCATG**CATGCA**
GCGCCACCTGGAAGCCGCGCGGCCGCTTTGGTTTTTTGTTTGGTTGGTTCCATTTTCTTTTTTTCTTTTT
TTTTTTAAGAAAAAATAAAAGGTGGATTTGAAAAAAAAAAAAAAAAAAAAA
