# Supplementary material for: Increased frequency of single base substitutions in a population of transcripts expressed in cancer cells
Source: BMC Cancer. 2012 Nov 8;12:509. doi: 10.1186/1471-2407-12-509 (PMC3522053; doi:10.1186/1471-2407-12-509)
Supplement: Additional file 7 — Manual checking of possible cancer related somatic mutations altering RT with greater SBS frequencies in cancer than in healthy cells (Tag-seq). [file 1471-2407-12-509-S7.pdf]

Additional_result_file_2 providing further information to Figure 2b:

Using the Tag-seq_CgtH_4289_RT_FDR_mapped transcripts with greater SBS in cancer than in healthy (Additional_tabular_file_3), a list of 5194 unique Genbank/RefSeq ID was obtained. These ID were converted to Gene ID using the Synergizer tool and produced a list of 2385 unique ID. Sixty eight genes (here below) were common to the census of somatically mutated genes in cancer and the Tag-seq_CgtH_4289_RT_FDR_mapped list.

Index	Gene ID	Description	Alteration of the 17 base *NlaIII* tag by known somatic mutations
1	207	AKT1, v-akt murine thymoma viral oncogene homolog 1	Known cancer-related somatic mutations do not alter the 17 base *NlaIII* tag
2	602	BCL3 B-cell CLL/lymphoma 3	Known cancer-related somatic mutations do not alter the 17 base *NlaIII* tag
3	604	BCL6 B-cell CLL/lymphoma 6	Known cancer-related somatic mutations do not alter the 17 base *NlaIII* tag
4	833	cars, cysteinyl-tRNA synthetase	Known cancer-related somatic mutations do not alter the 17 base *NlaIII* tag
5	1213	CLTC, clathrin, heavy chain	Known cancer-related somatic mutations do not alter the 17 base *NlaIII* tag
6	1387	CREBBP, CREB binding protein	Known cancer-related somatic mutations do not alter the 17 base *NlaIII* tag
7	1655	DDX5, DEAD (Asp-Glu-Ala-Asp) box polypeptide 5	Known cancer-related somatic mutations do not alter the 17 base *NlaIII* tag
8	1662	DDX10, DEAD (Asp-Glu-Ala-Asp) box polypeptide 10	Known cancer-related somatic mutations do not alter the 17 base *NlaIII* tag
9	2033	EP300, E1A binding protein p300	Known cancer-related somatic mutations do not alter the 17 base *NlaIII* tag
10	2060	EPS15, epidermal growth factor receptor pathway substrate 15	Known cancer-related somatic mutations do not alter the 17 base *NlaIII* tag
11	2120	ETV6, ets variant 6	Could not locate known somatic mutations on transcript
12	2130	EWSR1, Ewing sarcoma breakpoint region 1	Known cancer-related somatic mutations do not alter the 17 base *NlaIII* tag
13	2181	ACSL3, acyl-CoA synthetase long-chain family member 3	Known cancer-related somatic mutations do not alter the 17 base *NlaIII* tag
14	2313	FLI1, Friend leukemia virus integration 1	Known cancer-related somatic mutations do not alter the 17 base *NlaIII* tag
15	2956	MSH6, mutS homolog 6	Known cancer-related somatic mutations do not alter the 17 base *NlaIII* tag
16	3181	HNRNPA2B1 heterogeneous nuclear ribonucleoprotein A2/B1	Known cancer-related somatic mutations do not alter the 17 base *NlaIII* tag
17	3265	HRAS, v-Ha-ras Harvey rat sarcoma viral oncogene homolog	Known cancer-related somatic mutations do not alter the 17 base *NlaIII* tag
18	3718	JAK3, Janus kinase 3	Could not locate known somatic mutations on transcript
19	4089	SMAD4, SMAD family	Known cancer-related somatic mutations do not alter the

		member 4	17 base *NlaIII* tag
20	4299	AFF1, AF4/FMR2 family, member 1	Known cancer-related somatic mutations do not alter the 17 base *NlaIII* tag
21	4302	MLLT6, myeloid/lymphoid or mixed-lineage leukemia (trithorax homolog, Drosophila); translocated to, 6	Could not locate known somatic mutations on transcript
22	4303	FOXO4, forkhead box O4	Known cancer-related somatic mutations do not alter the 17 base *NlaIII* tag
23	4615	MYD88, myeloid differentiation primary response gene (88)	Known cancer-related somatic mutations do not alter the 17 base *NlaIII* tag
24	4780	NFE2L2, nuclear factor (erythroid-derived 2)-like 2	Known cancer-related somatic mutations do not alter the 17 base *NlaIII* tag
25	4841	NONO, non-POU domain containing, octamer-binding	Known cancer-related somatic mutations do not alter the 17 base *NlaIII* tag
26	4851	NOTCH1, notch 1	Known cancer-related somatic mutations do not alter the 17 base *NlaIII* tag
27	4853	NOTCH2, notch 2	Could not locate known somatic mutations on transcript
28	4928	NUP98, nucleoporin 98kDa	Could not locate known somatic mutations on transcript
29	5049	PAFAH1B2, platelet-activating factor acetylhydrolase 1b, catalytic subunit 2 (30kDa)	Known cancer-related somatic mutations do not alter the 17 base *NlaIII* tag
30	5518	PPP2R1A, protein phosphatase 2, regulatory subunit A, alpha	Known cancer-related somatic mutations do not alter the 17 base *NlaIII* tag
31	5546	PRCC, papillary renal cell carcinoma (translocation-associated)	Known cancer-related somatic mutations do not alter the 17 base *NlaIII* tag
32	5573	PRKAR1A, protein kinase, cAMP-dependent, regulatory, type I, alpha (tissue specific extinguisher 1)	Known cancer-related somatic mutations do not alter the 17 base *NlaIII* tag
33	5728	PTEN, phosphatase and tensin homolog	Known cancer-related somatic mutations do not alter the 17 base *NlaIII* tag
34	5894	RAF1, v-raf-1 murine leukemia viral oncogene homolog 1	Known cancer-related somatic mutations do not alter the 17 base *NlaIII* tag
35	5925	RB1, retinoblastoma 1	Known cancer-related somatic mutations do not alter the 17 base *NlaIII* tag
36	6146	RPL22, ribosomal protein L22	COSMIC does not report any somatic mutations in this gene
37	6416	MAP2K4, mitogen-activated protein kinase kinase 4	Known cancer-related somatic mutations do not alter the 17 base *NlaIII* tag
38	6418	SET, SET nuclear oncogene	COSMIC does not report any somatic mutations in this gene
39	6428	SRSF3,	Known cancer-related somatic mutations do not alter the

		serine/arginine-rich splicing factor 3	17 base *NlaIII* tag
40	6597	SMARCA4, SWI/SNF related, matrix associated, actin dependent regulator of chromatin, subfamily a, member 4	Known cancer-related somatic mutations do not alter the 17 base *NlaIII* tag
41	6794	STK11, serine/threonine kinase 11	Known cancer-related somatic mutations do not alter the 17 base *NlaIII* tag
42	6938	TCF12, transcription factor 12	Known cancer-related somatic mutations do not alter the 17 base *NlaIII* tag
43	7175	TPR, translocated promoter region (to activated MET oncogene)	Known cancer-related somatic mutations do not alter the 17 base *NlaIII* tag
44	7403	KDM6A, lysine (K)-specific demethylase 6A	Known cancer-related somatic mutations do not alter the 17 base *NlaIII* tag
45	7514	XPO1, exportin 1 (CRM1 homolog, yeast)	Known cancer-related somatic mutations do not alter the 17 base *NlaIII* tag
46	7750	ZMYM2, zinc finger, MYM-type 2	Known cancer-related somatic mutations do not alter the 17 base *NlaIII* tag
47	8028	MLLT10, myeloid/lymphoid or mixed-lineage leukemia (trithorax homolog, Drosophila); translocated to, 10	Known cancer-related somatic mutations do not alter the 17 base *NlaIII* tag
48	8030	CCDC6, coiled-coil domain containing 6	Known cancer-related somatic mutations do not alter the 17 base *NlaIII* tag
49	8242	KDM5C, lysine (K)-specific demethylase 5C	Known cancer-related somatic mutations do not alter the 17 base *NlaIII* tag
50	9709	HERPUD1, homocysteine-inducible, endoplasmic reticulum stress-inducible, ubiquitin-like domain member 1	COSMIC does not report any somatic mutations in this gene
51	9968	MED12, mediator complex subunit 12	Known cancer-related somatic mutations do not alter the 17 base *NlaIII* tag
52	10006	ABI1, abl-interactor 1	Known cancer-related somatic mutations do not alter the 17 base *NlaIII* tag
53	10142	AKAP9, A kinase (PRKA) anchor protein (yotiao) 9	Known cancer-related somatic mutations do not alter the 17 base *NlaIII* tag
54	10342	TFG, TRK-fused gene	Known cancer-related somatic mutations do not alter the 17 base *NlaIII* tag
55	10397	NDRG1, N-myc downstream regulated 1	Known cancer-related somatic mutations do not alter the 17 base *NlaIII* tag
56	10892	MALT1, mucosa associated lymphoid tissue lymphoma translocation gene 1	Known cancer-related somatic mutations do not alter the 17 base *NlaIII* tag
57	10962	MLLT11, myeloid/lymphoid or	Known cancer-related somatic mutations do not alter the 17 base *NlaIII* tag

		mixed-lineage leukemia (trithorax homolog, Drosophila); translocated to, 11	
58	10978	CLP1, cleavage and polyadenylation factor I subunit, homolog (S. cerevisiae)	Known cancer-related somatic mutations do not alter the 17 base *NlaIII* tag
59	23365	ARHGEF12, Rho guanine nucleotide exchange factor (GEF) 12	Could not locate known somatic mutations on transcript
60	23512	SUZ12, suppressor of zeste 12 homolog (Drosophila)	COSMIC does not report any somatic mutations in this gene
61	26039	SS18L1, synovial sarcoma translocation gene on chromosome 18-like 1	Known cancer-related somatic mutations do not alter the 17 base *NlaIII* tag
62	27086	FOXP1, forkhead box P1	Known cancer-related somatic mutations do not alter the 17 base *NlaIII* tag
63	51517	NCKIPSD, NCK interacting protein with SH3 domain	Known cancer-related somatic mutations do not alter the 17 base *NlaIII* tag
64	57120	GOPC, golgi-associated PDZ and coiled-coil motif containing	Known cancer-related somatic mutations do not alter the 17 base *NlaIII* tag
65	79145	CHCHD7, coiled-coil-helix-coiled-coil-helix domain containing 7	Could not locate known somatic mutations on transcript
66	81608	FIP1L1, FIP1 like 1 (S. cerevisiae)	Could not locate known somatic mutations on transcript
67	84441	MAML2, mastermind-like 2 (Drosophila)	Known cancer-related somatic mutations do not alter the 17 base *NlaIII* tag
68	116028	RMI2, RecQ mediated genome instability 2, homolog (S. cerevisiae)	Known cancer-related somatic mutations do not alter the 17 base *NlaIII* tag

Legend:

Red nucleotide = cancer related somatically mutated base
Green nucleotide = 17 base *NlaIII* tag
Blue nucleotide = *NlaIII* “CATG” recognition site
Underlined nucleotides = Somatic mutation neighbor bases displayed in the COSMIC snapshot

1) Entrez Gene ID 207 = AKT1, v-akt murine thymoma viral oncogene homolog 1 = BC000479

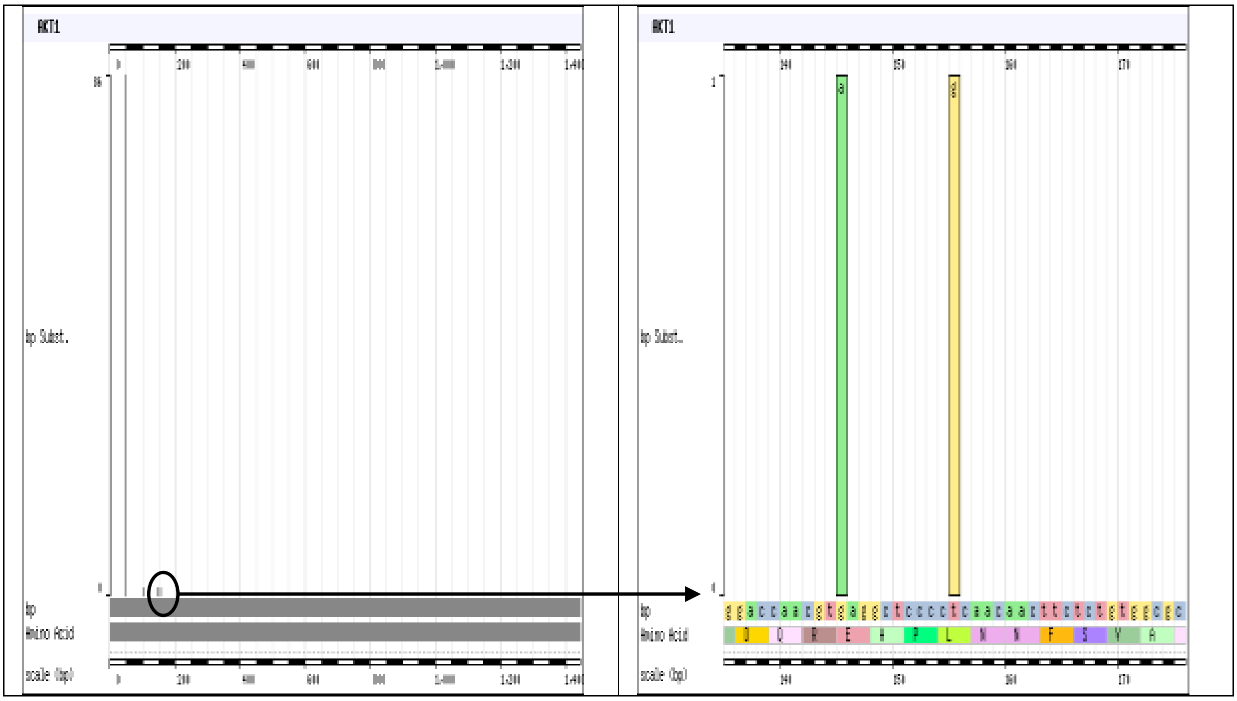

>gi|33875493|gb|BC000479.2| Homo sapiens v-akt murine thymoma viral oncogene homolog 1, mRNA (cDNA clone MGC:8686 IMAGE:2964603), complete cds

GGCGGCCGAGCACCGAGCGCTGGGCACCGGGCACCGAGCGGCGGCGGCACGCGAGGCCCGGCCCCGAGCA
GCGCCCCCGCCCGCCGCGGCCTCCAGCCCGGCCCCGCCCAGCGCCGGCCCGCGGGGATGCGGAGCGGCGG
GCGCCGGAGGCCGCGGCCCGGCTAGGCCCGCGCTCGCGCCCGGACGCGGCGGCCCGAGGCTGTGGCCAGG
CCAGCTGGGCTCGGGGAGCGCCAGCCTGAGAGGAGCGCGTGAGCGTCGCGGGAGCCTCGGGCACCATGAG
CGACGTGGCTATTGTGAAGGAGGGTTGGCTGCACAAACGAGGGGAGTACATCAAGACCTGGCGGCCACGC
TACTTCCTCCTCAAGAATGATGGCACCTTCATTGGCTACAAGGAGCGGCCGCAGGATGTGGACCAACGT**G**
AGGCTCCCC**T**CAACAACTTCTCTGTGGCGCAGTGCCAGCTGATGAAGACGGAGCGGCCCCGGCCCAACAC
CTTCATCATCCGCTGCCTGCAGTGGACCACTGTCATCGAACGCACCTTCCATGTGGAGACTCCTGAGGAG
CGGGAGGAGTGGACAACCGCCATCCAGACTGTGGCTGACGGCCTCAAGAAGCAGGAGGAGGAGGAGATGG
ACTTCCGGTCGGGCTCACCCAGTGACAACTCAGGGGCTGAAGAGATGGAGGTGTCCCTGGCCAAGCCCAA
GCACCGCGTGACCATGAACGAGTTTGAGTACCTGAAGCTGCTGGGCAAGGGCACTTTCGGCAAGGTGATC
CTGGTGAAGGAGAAGGCCACAGGCCGCTACTACGCCATGAAGATCCTCAAGAAGGAAGTCATCGTGGCCA
AGGACGAGGTGGCCCACACACTCACCGAGAACCGCGTCCTGCAGAACTCCAGGCACCCCTTCCTCACAGC
CCTGAAGTACTCTTTCCAGACCCACGACCGCCTCTGCTTTGTCATGGAGTACGCCAACGGGGGCGAGCTG
TTCTTCCACCTGTCCCGGGAGCGTGTGTTCTCCGAGGACCGGGCCCGCTTCTATGGCGCTGAGATTGTGT
CAGCCCTGGACTACCTGCACTCGGAGAAGAACGTGGTGTACCGGGACCTCAAGCTGGAGAACCTCATGCT
GGACAAGGACGGGCACATTAAGATCACAGACTTCGGGCTGTGCAAGGAGGGGATCAAGGACGGTGCCACC
ATGAAGACCTTTTGCGGCACACCTGAGTACCTGGCCCCCGAGGTGCTGGAGGACAATGACTACGGCCGTG
CAGTGGACTGGTGGGGGCTGGGCGTGGTCATGTACGAGATGATGTGCGGTCGCCTGCCCTTCTACAACCA
GGACCATGAGAAGCTTTTTGAGCTCATCCTCATGGAGGAGATCCGCTTCCCGCGCACGCTTGGTCCCGAG
GCCAAGTCCTTGCTTTCAGGGCTGCTCAAGAAGGACCCCAAGCAGAGGCTTGGCGGGGGCTCCGAGGACG
CCAAGGAGATCATGCAGCATCGCTTCTTTGCCGGTATCGTGTGGCAGCACGTGTACGAGAAGAAGCTCAG
CCCACCCTTCAAGCCCCAGGTCACGTCGGAGACTGACACCAGGTATTTTGATGAGGAGTTCACGGCCCAG
ATGATCACCATCACACCACCTGACCAAGATGACAGCATGGAGTGTGTGGACAGCGAGCGCAGGCCCCACT

TCCCCCAGTTCTCCTACTCGGCCAGCGGCACGGCCTGAGGCGGCGGTGGACTGCGCTGGACGATAGCTTG
GAGGGATGGAGAGGCGGCCTCGTGCCATGATCTGTATTTAATGGTTTTTATTTCTCGGGTGCATTTGAGA
GAAGCCACGCTGTCCTCTCGAGCCCAGATGGAAAGACGTTTTTGTGCTGTGGGCAGCACCCTCCCCCGCA
GCGGGGTAGGGAAGAAAACTATCCTGCGGGTTTTAATTTATTTCATCCAGTTTGTTCTCCGGGTGTGGCC
TCAGCCCTCAGAACAATCCGATTCACGTAGGGAAATGTTAAGGACTTCTGCAGCTATGCGCAATGTGGCA
TTGGGGGGCCGGGCAGGTCCTGCC**CATGTGTCCCCTCACTCTGTC**AGCCAGCCGCCCTGGGCTGTCTGTC
ACCAGCTATCTGTCATCTCTCTGGGGCCCTGGGCCTCAGTTCAACCTGGTGGCACCAGATGCAACCTCAC
TATGGTATGCTGGCCAGCACCCTCTCCTGGGGGTGGCAGGCACACAGCAGCCCCCCAGCACTAAGGCCGT
GTCTCTGAGGACGTCATCGGAGGCTGGGCCCCTGGGATGGGACCAGGGATGGGGGATGGGCCAGGGTTTA
CCCAGTGGGACAGAGGAGCAAGGTTTAAATTTGTTATTGTGTATTATGTTGTTCAAATGCATTTTGGGGG
TTTTTAATCTTTGTGACAGGAAAGCCCTCCCCCTTCCCCTTCTGTGTCACAGTTCTTGGTGACTGTCCCA
CCGGGAGCCTCCCCCTCAGATGATCTCTCCACGGTAGCACTTGACCTTTTCGACGCTTAACCTTTCCGCT
GTCGCCCCAGGCCCTCCCTGACTCCCTGTGGGGGTGGCCATCCCTGGGCCCCTCCACGCCTCCTGGCCAG
ACGCTGCCGCTGCCGCTGCACCACGGCGTTTTTTTACAACATTCAACTTTAGTATTTTTACTATTATAAT
ATAATATGGAACCTTCCCTCCAAATTCTTCAATAAAAGTTGCTTTTCAAAAAAAAAAAAAAAAAAAAA

2) Entrez Gene ID 602 = BCL3 B-cell CLL/lymphoma 3 = BC064993

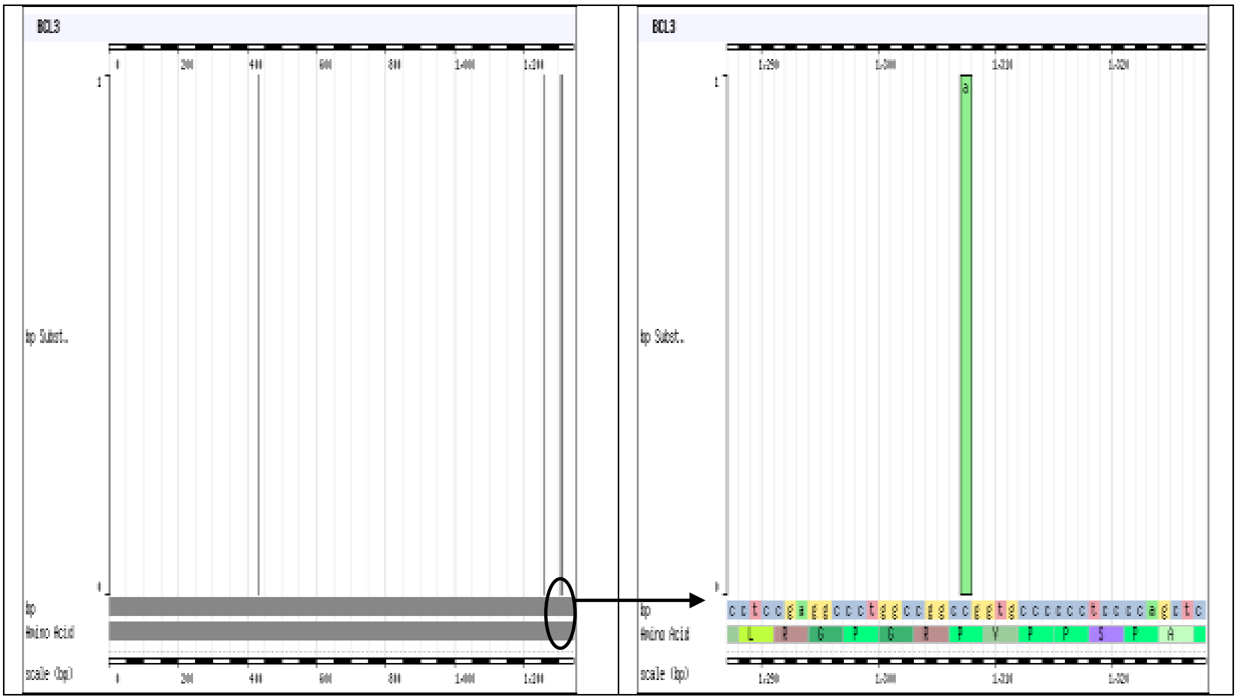

>gi|40675564|gb|BC064993.1| Homo sapiens B-cell CLL/lymphoma 3, mRNA (cDNA clone MGC:71163 IMAGE:5806689), complete cds
GCCCGGCGAAACCACCCTCCCGTGCAGCCGAGCCCAGCCGCTCTCCGGCCGCCGTCCCCGGCGGCCCCAT
GCCCCGATGCCCCGCGGGGGCCATGGACGAGGGGCCCGTGGACCTGCGCACCCGGCCCAAGGCCGCCGGA
CTCCCGGGCGCCGCGCTGCCGCTCCGCAAGCGCCCGCTGCGCGCGCCCTCCCCGGAGCCCGCCGCTCCCC
GCGGCGCTGCGGGCCTTGTCGTCCCCCTGGACCCTCTGCGCGGCGGCTGCGACCTGCCGGCGGTCCCCGG
GCCCCCCCACGGCCTGGCCCGGCCGGAGGCGCTTTACTACCCCGGAGCCTTACTGCCTTTGTACCCCACT
CGGGCCATGGGCTCCCCGTTTCCTCTGGTGAACCTGCCTACACCCCTATACCCCATGATGTGCCCCATGG
AACACCCCCTTTCTGCTGACATCGCCATGGCCACCCGTGCAGATGAGGACGGAGACACGCCTCTCCATAT
TGCTGTGGTGCAGGGTAACCTGCCAGCTGTGCACCGGCTGGTCAACCTCTTCCAGCAGGGGGGCCGGGAG
CTCGACATCTACAACAACCTACGGCAGACACCGCTCCACCTGGCTGTGATCACCACATTACCGTCTGTGG
TCCGGCTCCTGGTGACAGCTGGTGCCAGCCCCATGGCGCTGGACCGCCATGGCCAGACGGCCGCTCACCT
GGCGTGCGAGCACCGCAGCCCGACCTGCCTGCGAGCCCTGCTGGACAGCGCAGCTCCGGGCACGTTGGAC
CTGGAGGCCCGCAATTATGACGGGCTCACCGCCCTGCACGTGGCAGTGAACACCGAGTGCCAAGAAACCG
TGCAGCTCTTGCTAGAGCGCGGTGCCGACATCGACGCAGTGGACATTAAGAGCGGCCGCTCCCCGCTCAT
CCACGCCGTGGAAAACAACAGCCTTAGCATGGTGCAGCTGCTGCTGCAGCACGGCGCCAACGTGAACGCG

CAAATGTACTCCGGCAGCTCCGCCCTGCACTCAGCGTCCGGCCGCGGGCTCCTCCCGCTGGTGCGCACGC
TGGTCCGCAGCGGCGCTGACAGCAGCCTCAAGAACTGCCACAACGACACGCCGCTCATGGTGGCGCGCAG
CCGCAGGGTCATCGACATCCTGAGGGGGAAGGCCACCCGGCCTGCTTCCACCTCCCAGCCAGACCCCTCC
CCTGACCGGAGCGCCAACACCTCCCCCGAGAGCAGCAGCCGCCTCAGCTCCAATGGTCTTCTCTCCGCAT
CACCATCCTCCTCACCCTCCCAGTCTCCCCCCAGGGACCCCCCTGGATTCCCCATGGCTCCTCCCAATTT
CTTCCTTCCTTCCCCATCTCCACCCGCCTTCCTGCCCTTTGCTGGGGTCCTCCGAGGCCCTGGCCGGC**C**G
GTGCCCCCCTCCCCAGCTCCAGGAGGCAGCTGAGGGGGATGGGGGGGCAGATCTTGGACTCATGAGGAGG
GGCCCCCCTGCCCTGTGGGGTCAACCCTTCTGGAAACTGTGAAGATCTCACTCTGCCCCCCCCCCCCATC
TTCGGGACCAGGATTTGCACAGAAGCACATGCACCTACCCATACACCCCCTCTTCTGAGCACAGATGTTC
CCCCATCTCGCTCCCTCCCAGGACTCTGACCCCAGCATTCTCAGGCACCAGTCCCTGTCCGGAATGCCAC
CCACATCTTCCATTTC**CATGTCCCCTCCCAGAGCTGG**TGGACCCAGGGAACAGCCACTCCCCTCCACTCT
CTACCAGATAACTGAGGAGGGGAGAGGTGGGCCGTAACGGGCACGGATCACGATGTAAATTATTAAGCAT
TTTGGTTGGATTTCTTTTGTAATAAACTATTTTTGTACCATAAAAAAAAAAAAAAAAAA

3) Entrez Gene ID 604 = BCL6 B-cell CLL/lymphoma 6 = BC142705

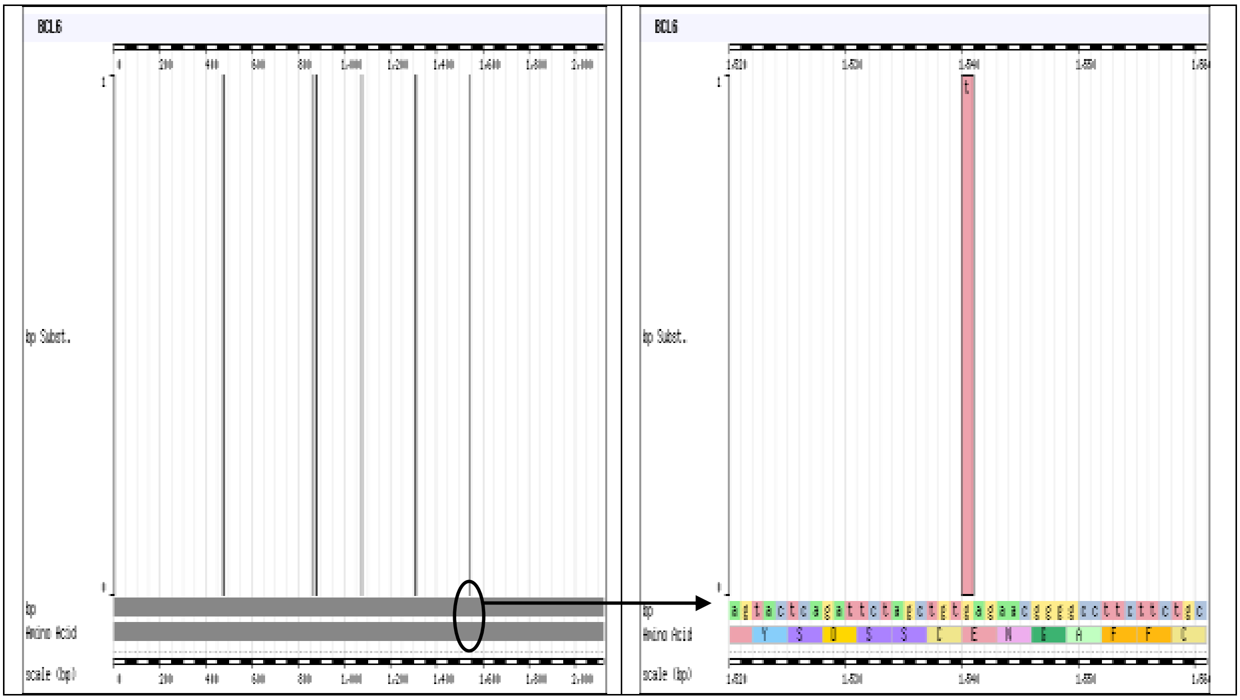

>gi|148744385|gb|BC142705.1| Homo sapiens B-cell CLL/lymphoma 6, mRNA (cDNA clone MGC:165045 IMAGE:40148809), complete cds
CAAGAAGTTTCTAGGAAAGGCCGGACACCAGGTTTTGAGCAAAATTTTGGACTGTGAAGCAAGGCATTGG
TGAAGACAAAATGGCCTCGCCGGCTGACAGCTGTATCCAGTTCACCCGCCATGCCAGTGATGTTCTTCTC
AACCTTAATCGTCTCCGGAGTCGAGACATCTTGACTGATGTTGTCATTGTTGTGAGCCGTGAGCAGTTTA
GAGCCCATAAAACGGTCCTCATGGCCTGCAGTGGCCTGTTCTATAGCATCTTTACAGACCAGTTGAAATG
CAACCTTAGTGTGATCAATCTAGATCCTGAGATCAACCCTGAGGGATTCTGCATCCTCCTGGACTTCATG
TACACATCTCGGCTCAATTTGCGGGAGGGCAACATCATGGCTGTGATGGCCACGGCTATGTACCTGCAGA
TGGAGCATGTTGTGGACACTTGCCGGAAGTTTATTAAGGCCAGTGAAGCAGAGATGGTTTCTGCCATCAA
GCCTCCTCGTGAAGAGTTCCTCAACAGCCGGATGCTGATGCCCCAAGACATCATGGCCTATCGGGGTTGT
GAGGTGGTGGAGAACAACCTGCCACTGAGGAGCGCCCCTGGGTGTGAGAGCAGAGCCTTTGCCCCCAGCC
TGTACAGTGGCCTGTCCACACCGCCAGCCTCTTATTCCATGTACAGCCACCTCCCTGTCAGCAGCCTCCT
CTTCTCCGATGAGGAGTTTCGGGATGTCCGGATGCCTGTGGCCAACCCCTTCCCCAAGGAGCGGGCACTC
CCATGTGATAGTGCCAGGCCAGTCCCTGGTGAGTACAGCCGGCCGACTTTGGAGGTGTCCCCCAATGTGT
GCCACAGCAATATCTATTCACCCAAGGAAACAATCCCAGAAGAGGCACGAAGTGATATGCACTACAGTGT
GGCTGAGGGCCTCAAACCTGCTGCCCCCTCAGCCCGAAATGCCCCCTACTTCCCTTGTGACAAGGCCAGC
AAAGAAGAAGAGAGACCCTCCTCGGAAGATGAGATTGCCCTGCATTTCGAGCCCCCCAATGCACCCCTGA
ACCGGAAGGGTCTGGTTAGTCCACAGAGCCCCCAGAAATCTGACTGCCAGCCCAACTCGCCCACAGAGTC

CTGCAGCAGTAAGAATGCCTGCATCCTCCAGGCTTCTGGCTCCCCTCCAGCCAAGAGCCCCACTGACCCC
AAAGCCTGCAACTGGAAGAAATACAAGTTCATCGTGCTCAACAGCCTCAATCAGAATGCCAAACCAGAGG
GGCCTGAGCAGGCTGAGCTGGGCCGCCTTTCCCCACGAGCCTACACGGCCCCACCTGCCTGCCAGCCACC
CATGGAGCCTGAGAACCTTGACCTCCAGTCCCCAACCAAGCTGAGTGCCAGCGGGGAGGACTCCACCATC
CCACAAGCCAGCCGGCTCAATAACATCGTTAACAGGTCCATGACGGGCTCTCCCCGCAGCAGCAGCGAGA
GCCACTCACCACTCTACATGCACCCCCCGAAGTGCACGTCCTGCGGCTCTCAGTCCCCACAGCATGCAGA
GATGTGCCTCCACACCGCTGGCCCCACGTTCCCTGAGGAGATGGGAGAGACCCAGTCTGAGTACTCAGAT
TCTAGCTGTG**A**GAACGGGGCCTTCTTCTGCAATGAGTGTGACTGCCGCTTCTCTGAGGAGGCCTCACTCA
AGAGGCACACGCTGCAGACCCACAGTGACAAACCCTACAAGTGTGACCGCTGCCAGGCCTCCTTCCGCTA
CAAGGGCAACCTCGCCAGCCACAAGACCGTCCATACCGGTGAGAAACCCTATCGTTGCAACATCTGTGGG
GCCCAGTTCAACCGGCCAGCCAACCTGAAAACCCACACTCGAATTCACTCTGGAGAGAAGCCCTACAAAT
GCGAAACCTGCGGAGCCAGATTTGTACAGGTGGCCCACCTCCGTGCCCATGTGCTTATCCACACTGGTGA
GAAGCCCTATCCCTGTGAAATCTGTGGCACCCGTTTCCGGCACCTTCAGACTCTGAAGAGCCACCTGCGA
ATCCACACAGGAGAGAAACCTTACCATTGTGAGAAGTGTAACCTGCATTTCCGTCACAAAAGCCAGCTGC
GACTTCACTTGCGCCAGAAGCATGGCGCCATCACCAACACCAAGGTGCAATACCGCGTGTCAGCCACTGA
CCTGCCTCCGGAGCTCCCCAAAGCCTGCTGAAGCATGGAGTGTTGATGCTTTCGTCTCCAGCCCCTTCTC
AGAATCTACCCAAAGGATACTGTAACACTTTACAATGTTCATCCCATGATGTAGTGCCTCTTTCATCCAC
TAGTGCAAATCATAGCTGGGGGTTGGGGGTGGTGGGGGTCGGGGCCTGGGGGACTGGGAGCCGCAGCAGC
TCCCCCTCCCCCACTGCCATAAAACATTAAGAAAATCATATTGCTTCTTCTCCTATGTGTAAGGTGAACC
ATGTCAGCAAAAAGCAAAATCATTTTATATGTCAAAGCAGGGGAGTATGCAAAAGTTCTGACTTGACTTT
AGTCTGCAAAATGAGGAATGTATATGTTTTGTGGGAACAGATGTTTCTTTTGTATGTAAATGTGCATTCT
TTTAAAAGACAAGACTTCAGTATGTTGTCAAAGAGAGGGCTTTAATTTTTTTAACCAAAGGTGAAGGAAT
ATATGGCAGAGTTGTAAATATATAAATATATATATATATATATAAAATAAATATATATAAACCTAAAAAAGA
TATATTAAAAATATAAAACTGCGTTAAAGGCTCGATTTTGTATCTGCAGGCAGACACGGATCTGAGAATC
TTTATTGAGAAAGAGCACTTAAGAGAATATTTTAAGTATTGCATCTGTATAAGTAAGAAAATATTTTGTC
TAAAATGCCTCAGTGTATTTGTATTTTTTTGCAAGTGAAGGTTTACAATTTACAAAGTGTGTATTAAAAA
AAACAAAAAGAACAAAAAAATCTGCAGAAGGAAAAATGTGTAATTTTGTTCTAGTTTTCAGTTTGTATAT
ACCCGTACAACGTGTCCTCACGGTGCCTTTTTTCACGGAAGTTTTCAATGATGGGCGAGCGTGCACCATC
CCTTTTTGAAGTGTAGGCAGACACAGGGACTTGAAGTTGTTACTAACTAAACTCTCTTTGGGAATGTTTG
TCTCATCCCATTCTGCGT**CATGCTTGTGTTATAACTACT**CCGGAGACAGGGTTTGGCTGTGTCTAAACTG
CATTACCGCGTTGTAAAATATAGCTGTACAAATATAAGAATAAAATGTTGAAAAGTCAAAAAAAAAAAAA
AAAAAAAAAAA

4) Entrez Gene ID 833 = cars, cysteinyl-tRNA synthetase = BX647906 or BC002880

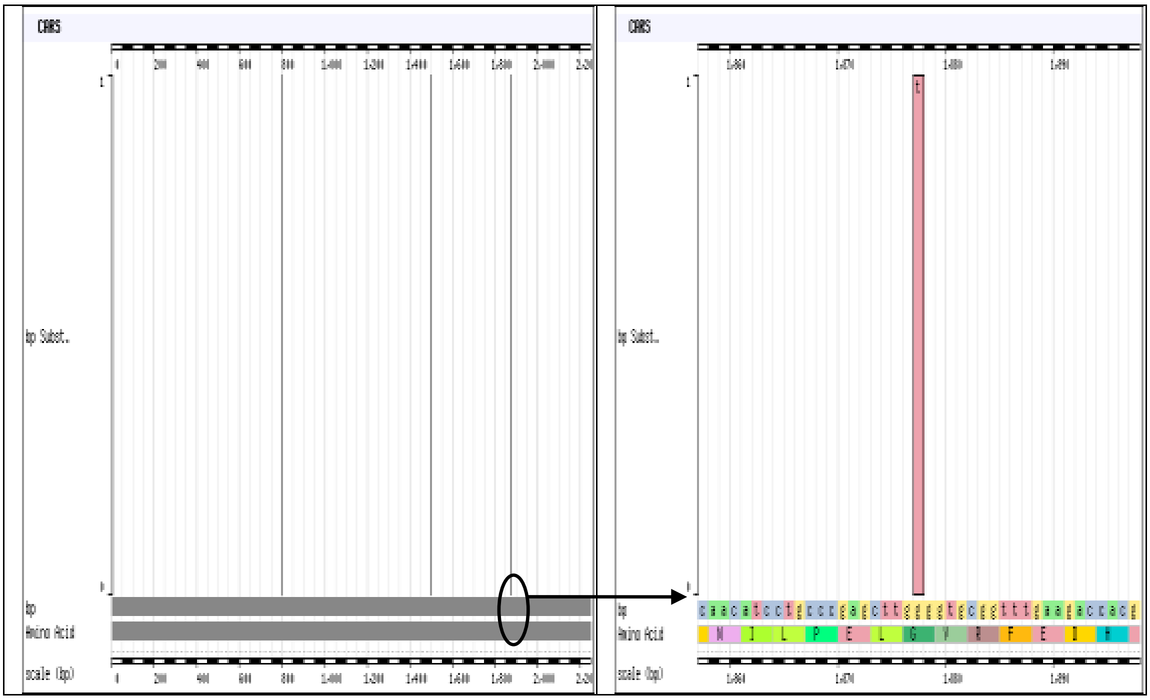

>gi|34367065|emb|BX647906.1| Homo sapiens mRNA; cDNA DKFZp686F1612 (from clone DKFZp686F1612)

GGGCATCAGATTCTAGGAAGTGTCTGTAGCCGCAGCTGCGGGTCCGGGATTCCCAGCCATGGCAGATTCC
TCCGGGCAGCAGGCTCCTGACTACAGGTCCATTCTGAGCATTAGTGACGAGGCAGCCAGGGCACAAGCCC
TGAACGAGCACCTCAGCACGCGTAGCTATGTCCAGGGGTACTCACTGTCCCAGGCAGACGTGGACGCGTT
CAGGCAGCTCTCGGCCCCGCCCGCTGACCCCCAGCTCTTCCACGTGGCTCGGTGGTTCAGGCACATAGAA
GCGCTCCTGGGTAGCCCCTGTGGCAAAGGCCAGCCCTGCAGGCTCCAAGCAAGCAAAGGCCGGCGTGTGC
AGCCCCAGTGGTCCCCTCCTGCTGGGACCCAGCCATGCAGACTCCACCTTTACAACAGCCTCACCAGGAA
CAAGGAAGTGTTCATACCTCAAGATGGGAAAAAGGTGACGTGGTATTGCTGTGGGCCAACCGTCTATGAC
GCATCTCACATGGGGCACGCCAGGTCCTACATCTCTTTTGATATCTTGAGAAGAGTGTTGAAGGATTACT
TCAAATTTGATGTCTTTTATTGCATGAACATTACGGATATTGATGACAAGATCATCAAGAGGGCCCGGCA
GAACCACCTGTTCGAGCAGTATCGGGAGAAGAGGCCTGAAGCGGCACAGCTCTTGGAGGATGTTCAGGCC
GCCCTGAAGCCATTTTCAGTAAAATTAAATGAGACCACGGATCCCGATAAAAAGCAGATGCTCGAACGGA
TTCAGCACGCAGTGCAGCTTGCCACAGAGCCACTTGAGAAAGCTGTGCAGTCCAGACTCACGGGAGAGGA
AGTCAACAGCTGTGTGGAGGTGTTGCTGGAAGAAGCCAAGGATTTGCTCTCTGACTGGCTGGATTCTACA
CTTGGCTGTGATGTCACTGACAATTCCATCTTCTCCAAGCTGCCCAAGTTCTGGGAGGGGGACTTCCACA
GAGACATGGAAGCTCTGAATGTTCTCCCTCCAGATGTCTTAACCCGGGTTAGTGAGTATGTGCCAGAAAT
TGTGAACTTTGTCCAGAAGATTGTGGACAACGGTTACGGCTATGTCTCCAATGGGTCTGTCTACTTTGAT
ACAGCGAAGTTTGCTTCTAGCGAGAAGCACTCCTATGGGAAGCTGGTGCCTGAGGCCGTTGGAGATCAGA
AAGCCCTTCAAGAAGGGGAAGGTGACCTGAGCATCTCTGCAGACCGCCTGAGTGAGAAGCGCTCTCCCAA
CGACTTTGCCTTATGGAAGGCCTCTAAGCCCGGAGAACCGTCCTGGCCGTGCCCTTGGGGAAAGGGTCGT
CCGGGCTGGCATATCGAGTGCTCGGCCATGGCAGGCACCCTCCTAGGGGCTTCGATGGACATTCACGGAG
GTGGGTTCGACCTCCGGTTCCCCCACCATGACAATGAGCTGGCACAGTCGGAGGCCTACTTTGAAAACGA
CTGCTGGGTCAGGTACTTCCTGCACACAGGCCACCTGACCATTGCAGGCTGCAAAATGTCAAAGTCACTA
AAAAACTTCATCACCATTAAAGATGCCTTGAAAAAGCACTCAGCACGGCAGTTGCGGCTGGCCTTCCTCA
TGCACTCGTGGAAGGACACCCTGGACTACTCCAGCAACACCATGGAGTCAGCGCTTCAATATGAGAAGTT
CTTGAATGAGTTTTTCTTAAATGTGAAAGATATCCTTCGCGCTCCTGTTGACATCACTGGTCAGTTTGAG
AAGTGGGGAGAAGAAGAAGCAGAACTGAATAAGAACTTTTATGACAAGAAGACAGCAATTCACAAAGCCC
TCTGTGACAATGTTGACACCCGCACCGTCATGGAAGAGATGCGGGCCTTGGTCAGTCAGTGCAACCTCTA
TATGGCAGCCCGGAAAGCCGTGAGGAAGAGGCCCAACCAGGCTCTGCTGGAGAACATCGCCCTGTACCTC
ACCCATATGCTGAAGATCTTTGGGGCCGTAGAAGAGGACAGCTCCCTGGGATTCCCGGTCGGAGGGCCTG
GAACCAGCCTCAGTCTCGAGGCCACAGTCATGCCCTACCTTCAGGTGTTATCAGAATTCCGAGAAGGAGT
GCGGAAGATTGCCCGAGAGCAAAAAGTCCCTGAGATTCTGCAGCTCAGCGATGCCCTGCGGGACAACATC
CTGCCCGAGCTTG**G**GGTGCGGTTTGAAGACCACGAAGGACTGCCCACAGTGGTGAAACTGGTAGACAGAA
ACACCTTATTAAAAGAGAGAGAAGAAAAGAGACGGGTTGAAGAGGAGAAGAGGAAGAAGAAAGAGGAGGC
GGCCCGGAGGAAACAGGAACAAGAAGCAGCAAAGCTGGCCAAGATGAAGATTCCCCCCAGTGAGATGTTC
TTGTCAGAAACCGACAAATACTCCAAGTTTGATGAAAATGGTCTGCCCACACATGACATGGAGGGCAAAG
AGCTCAGCAAAGGGCAAGCCAAGAAGCTGAAGAAGCTCTTCGAGGCTCAGGAGAAGCTCTACAAGGAATA
TCTGCAGATGGCCCAGAATGGAAGCTTCCAGTGAGGGGGCACAGGACTGACTTTTTAAACCATTGTGGAC
TAGTGGCTGCTGTCTGCCTCAGTGACAATGTCCCAGCGCTCCTATCATGTTTACAGTCACCCTTGGGTCC
TAAATTAAGAGTTGTGTT**CATGTAGGTTCGTGTCGTCGT**TGGCTCTGAGACATTGATAATAAATTTTTCT
CAACAGTGAAAAAAAAAAAAA

5) Entrez Gene ID 1213 = CLTC, clathrin, heavy chain = BC015854

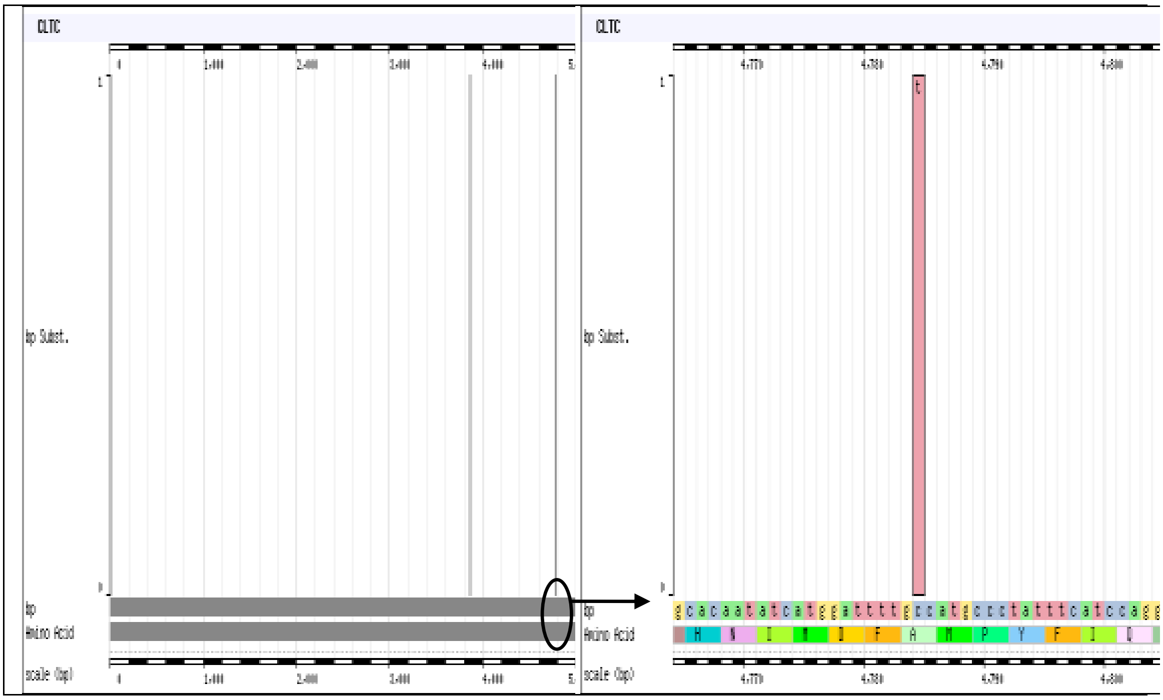

>gi|16198393|gb|BC015854.1| Homo sapiens clathrin, heavy chain (Hc), mRNA (cDNA clone IMAGE:4663039), with apparent retained intron

GGGGAATTCAAGCCTCTGTTGTTAAATGATTTGCTGATGGTGCTGTCTCCACGGTTGGATCACACTCGTG
CAGTCAATTATTTCAGCAAGGTTAAACAGCTACCACTGGTGAAACCGTATTTGCGTTCAGTTCAGAACCA
TAACAACAAATCTGTGAATGAATCATTGAACAATCTTTTTATTACAGAAGAAGATTATCAGGCTCTGCGA
ACATCAATAGATGCTTATGACAACTTTGACAATATCTCGCTTGCTCAGCGTTTGGAAAAACATGAACTCA
TTGAGTTCAGGAGAATTGCTGCTTATCTCTTCAAAGGCAACAATCGCTGGAAACAGAGTGTAGAGCTGTG
CAAGAAAGACAGCCTTTACAAGGATGCAATGCAGTATGCTTCTGAATCTAAAGATACTGAATTGGCTGAA
GAACTCCTGCAGTGGTTTTTGCAGGAAGAAAAAAGAGAGTGCTTTGGAGCTTGTCTGTTTACCTGTTACG
ATCTTTTAAGGCCAGATGTCGTCCTAGAAACTGCATGGAGGCACAATATCATGGATTTTG**C**CATGCCCTA
TTTCATCCAGGTCATGAAGGAGTACTTGACAAAGGTGGATAAATTAGATGCTTCAGAATCACTGAGAAAA
GAAGAAGAACAAGCTACAGAGACACAACCCATTGTTTATGGTCAGCCCCAGTTGATGCTGACAGCAGGAC
CCAGTGTTGCCGTCCCTCCCCAGGCACCTTTTGGTTATGGTTATACCGCACCACCGTATGGACAGCCACA
GCCTGGCTTTGGGTACAGCATGTGAGATGAAGCGCTGATCCTGTAGTCACCTATTTTCGTACTGAAACAT
CGTCTTTACCCACTTCTCAGTTTATAATGGGGGAAAACAGGCAACGTGTCCTTGTAACCTTTATTTCATG
AAGGACTTCTTTTTGTTTCTAACTATAAACTTGGATCACCTATGTTAAAACCTTATTTCACATTCCACAT
CATTTTAGAATTTATTTTCGAAGGGGAATAGTTTCAATGTTTTATTCACTTGGGCTTTTTTTCTTCCCCC
TCTTTCTTTAAAGAACTGCTCAATATTCAATCTGTTGTGAAGAACCTGATTTGCACTCTGTAGTGTTTAA
AGAAACAAAGAAACTCTAATATTGAATCTCTTAAATTTAGTGTATGTAAACAGCTTACAAATACGTATTG
TCTAAATGCATTTAAATCTGTTTTATTCAAAGAAAAGCTAAAGCAAAAACACTGGCATATGACCATGCAA
GACTGTCAGTGCCAACAAAGACAACACTAATCAGCACATCGTACACTGGATTGCAGTGCTTCCCAGATTA
TTGAAAAATGTTACAGACAACTTGCCTGATTTTTAAATGAGCGTAAAAGGCCCTCTAACCTATGCAGGTT
TCCCCATTATGCATATAGAAAATGCTAGTATGTTTTGCTCACTTCATATGTAACAGGTGCCCTTATGTTG
TGCTGTATCCTGTGCTTTTTCTGTGGGACCATTCCATTCAGGAGCAAAGAGCAC**CATGATTCCAATCTTG**
TGTGTGTTTACTAACCCTTCCCTGAGGTTTGTGTATGTTGGATATTGTGGTGTTTTAGATCACTGAGTGT

ACAGAAGAGAGAAATTCAAACAAAATATTGCTGTTCTTCAGTTTTGTTTGTGGAATTTGAAATTACTCAA
ATTTAAAATAAATTACTGGACTGTGGAAATAACATAGAATTGAAGTTTTAATTAAATACCACTCAAACGA
AAAAAAAAAAAAAAAAAAAAAAAAAA

6) Entrez Gene ID 1387 = CREBBP, CREB binding protein = U47741

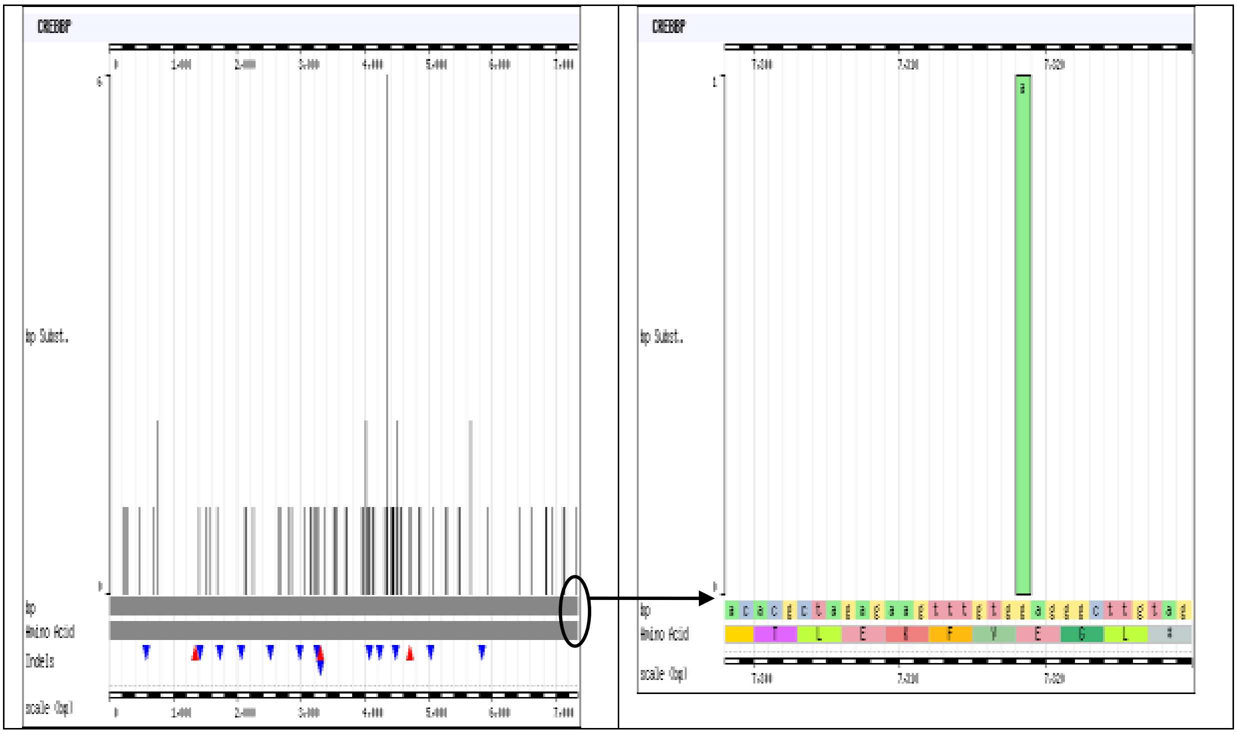

>gi|2443858|gb|U47741.1|HSU47741 Human CREB-binding protein (CBP) mRNA, complete cds

TGAGGAATCAACAGCCGCCATCTTGTCGCGGACCCGACCGGGGCTTCGAGCGCGATCTACTCGGCCCCGC
CGGTCCCGGGCCCCACAACCGCCCGCGCTCGCTCCTCTCCCTCGCAGCCGGCAGGGCCCCCGACCCCCGT
CCGGGCCCTCGCCGGCCCGGCCGCCCGTGCCCGGGGCTGTTTTCGCGAGCAGGTGAAAATGGCTGAGAAC
TTGCTGGACGGACCGCCCAACCCCAAAAGAGCCAAACTCAGCTCGCCCGGTTTCTCGGCGAATGACAGCA
CAGATTTTGGATCATTGTTTGACTTGGAAAATGATCTTCCTGATGAGCTGATACCCAATGGAGGAGAATT
AGGCCTTTTAAACAGTGGGAACCTTGTTCCAGATGCTGCTTCCAAACATAAACAACTGTCGGAGCTTCTA
CGAGGAGGCAGCGGCTCTAGTATCAACCCAGGAATAGGAAATGTGAGCGCCAGCAGCCCCGTGCAGCAGG
GCCTGGGTGGCCAGGCTCAAGGGCAGCCGAACAGTGCTAACATGGCCAGCCTCAGTGCCATGGGCAAGAG
CCCTCTGAGCCAGGGAGATTCTTCAGCCCCCAGCCTGCCTAAACAGGCAGCCAGCACCTCTGGGCCCACC
CCCGCTGCCTCCCAAGCACTGAATCCGCAAGCACAAAAGCAAGTGGGGCTGGCGACTAGCAGCCCTGCCA
CGTCACAGACTGGACCTGGTATCTGCATGAATGCTAACTTTAACCAGACCCACCCAGGCCTCCTCAATAG
TAACTCTGGCCATAGCTTAATTAATCAGGCTTCACAAGGGCAGGCGCAAGTCATGAATGGATCTCTTGGG
GCTGCTGGCAGAGGAAGGGGAGCTGGAATGCCGTACCCTACTCCAGCCATGCAGGGCGCCTCGAGCAGCG
TGCTGGCTGAGACCCTAACGCAGGTTTCCCCGCAAATGACTGGTCACGCGGGACTGAACACCGCACAGGC
AGGAGGCATGGCCAAGATGGGAATAACTGGGAACACAAGTCCATTTGGACAGCCCTTTAGTCAAGCTGGA
GGGCAGCCAATGGGAGCCACTGGAGTGAACCCCCAGTTAGCCAGCAAACAGAGCATGGTCAACAGTTTGC
CCACCTTCCCTACAGATATCAAGAATACTTCAGTCACCAACGTGCCAAATATGTCTCAGATGCAAACATC
AGTGGGAATTGTACCCACACAAGCAATTGCAACAGGCCCCACTGCAGATCCTGAAAAACGCAAACTGATA
CAGCAGCAGCTGGTTCTACTGCTTCATGCTCATAAGTGTCAGAGACGAGAGCAAGCAAACGGAGAGGTTC
GGGCCTGCTCGCTCCCGCATTGTCGAACCATGAAAAACGTTTTGAATCACATGACGCATTGTCAGGCTGG
GAAAGCCTGCCAAGTTGCCCATTGTGCATCTTCACGACAAATCATCTCTCATTGGAAGAACTGCACACGA
CATGACTGTCCTGTTTGCCTCCCTTTGAAAAATGCCAGTGACAAGCGAAACCAACAAACCATCCTGGGGT

CTCCAGCTAGTGGAATTCAAAACACAATTGGTTCTGTTGGCACAGGGCAACAGAATGCCACTTCTTTAAG
TAACCCAAATCCCATAGACCCCAGCTCCATGCAGCGAGCCTATGCTGCTCTCGGACTCCCCTACATGAAC
CAGCCCCAGACGCAGCTGCAGCCTCAGGTTCCTGGCCAGCAACCAGCACAGCCTCAAACCCACCAGCAGA
TGAGGACTCTCAACCCCCTGGGAAATAATCCAATGAACATTCCAGCAGGAGGAATAACAACAGATCAGCA
GCCCCCAAACTTGATTTCAGAATCAGCTCTTCCGACTTCCCTGGGGGCCACAAACCCACTGATGAACGAT
GGCTCCAACTCTGGTAACATTGGAACCCTCAGCACTATACCAACAGCAGCTCCTCCTTCTAGCACCGGTG
TAAGGAAAGGCTGGCACGAACATGTCACTCAGGACCTGCGGAGCCATCTAGTGCATAAACTCGTCCAAGC
CATCTTCCCAACACCTGATCCCGCAGCTCTAAAGGATCGCCGCATGGAAAACCTGGTAGCCTATGCTAAG
AAAGTGGAAGGGGACATGTACGAGTCTGCCAACAGCAGGGATGAATATTATCACTTATTAGCAGAGAAAA
TCTACAAGATACAAAAAGAACTAGAAGAAAAACGGAGGTCGCGTTTACATAAACAAGGCATCTTGGGGAA
CCAGCCAGCCTTACCAGCCCCGGGGGCTCAGCCCCCTGTGATTCCACAGGCACAACCTGTGAGACCTCCA
AATGGACCCCTGTCCCTGCCAGTGAATCGCATGCAAGTTTCTCAAGGGATGAATTCATTTAACCCCATGT
CCTTGGGGAACGTCCAGTTGCCACAAGCACCCATGGACCTCGTGCAGCCTCCCCAATGAACCACTCTGT
CCAGATGAACAGCATGGGCTCAGTGCCAGGGATGGCCATTTCTCCTTCCCGAATGCCTCAGCCTCCGAAC
ATGATGGGTGCACACACCAACAACATGATGGCCCAGGCGCCCGCTCAGAGCCAGTTTCTGCCACAGAACC
AGTTCCCGTCATCCAGCGGGGCGATGAGTGTGGGCATGGGGCAGCCGCCAGCCCAAACAGGCGTGTCACA
GGGACAGGTGCCTGGTGCTGCTCTTCCTAACCCTCTCAACATGCTGGGGCCTCAGGCCAGCCAGCTACCT
TGCCCTCCAGTGACACAGTCACCACTGCACCCAACACCGCCTCCTGCTTCCACGGCTGCTGGCATGCCAT
CTCTCCAGCACACGACACCACCTGGGATGACTCCTCCCCAGCCAGCAGCTCCCACTCAGCCATCAACTCC
TGTGTCGTCTTCCGGGCAGACTCCCACCCCGACTCCTGGCTCAGTGCCCAGTGCTACCCAAACCCAGAGC
ACCCCTACAGTCCAGGCAGCAGCCCAGGCCCAGGTGACCCCGCAGCCTCAAACCCCAGTTCAGCCCCCGT
CTGTGGCTACCCCTCAGTCATCGCAGCAACAGCCGACGCCTGTGCACGCCCAGCCTCCTGGCACACCGCT
TTCCCAGGCAGCAGCCAGCATTGATAACAGAGTCCCTACCCCCTCCTCGGTGGCCAGCGCAGAAACCAAT
TCCCAGCAGCCAGGACCTGACGTACCTGTGCTGGAAATGAAGACGGAGACCCAAGCAGAGGACACTGAGC
CCGATCCTGGTGAATCCAAAGGGGAGCCCAGGTCTGAGATGATGGAGGAGGATTTGCAAGGAGCTTCCCA
AGTTAAAGAAGAAACAGACATAGCAGAGCAGAAATCAGAACCAATGGAAGTGGATGAAAAGAAACCTGAA
GTGAAAGTAGAAGTTAAAGAGGAAGAAGAGAGTAGCAGTAACGGCACAGCCTCTCAGTCAACATCTCCTT
CGCAGCCGCGCAAAAAAATCTTTAAACCAGAGGAGTTACGCCAGGCCCTCATGCCAACCCTAGAAGCACT
GTATCGACAGGACCCAGAGTCATTACCTTTCCGGCAGCCTGTAGATCCCCAGCTCCTCGGAATTCCAGAC
TATTTTGACATCGTAAAGAATCCCATGGACCTCTCCACCATCAAGCGGAAGCTGGACACAGGGCAATACC
AAGAGCCCTGGCAGTACGTGGACGACGTCTGGCTCATGTTCAACAATGCCTGGCTCTATAATCGCAAGAC
ATCCCGAGTCTATAAGTTTTGCAGTAAGCTTGCAGAGGTCTTTGAGCAGGAAATTGACCCTGTCATGCAG
TCCCTTGGATATTGCTGTGGACGCAAGTATGAGTTTTCCCCACAGACTTTGTGCTGCTATGGGAAGCAGC
TGTGTACCATTCCTCGCGATGCTGCCTACTACAGCTATCAGAATAGGTATCATTTCTGTGAGAAGTGTTT
CACAGAGATCCAGGGCGAGAATGTGACCCTGGGTGACGACCCTTCACAGCCCCAGACGACAATTTCAAAG
GATCAGTTTGAAAAGAAGAAAAATGATACCTTAGACCCCGAACCTTTCGTTGATTGCAAGGAGTGTGGCC
GGAAGATGCATCAGATTTGCGTTCTGCACTATGACATCATTTGGCCTTCAGGTTTTGTGTGCGACAACTG
CTTGAAGAAAACTGGCAGACCTCGAAAAGAAAACAAATTCAGTGCTAAGAGGCTGCAGACCACAAGACTG
GGAAACCACTTGGAAGACCGAGTGAACAAATTTTTGCGGCGCCAGAATCACCCTGAAGCCGGGGAGGTTT
TTGTCCGAGTGGTGGCCAGCTCAGACAAGACGGTGGAGGTCAAGCCCGGGATGAAGTCACGGTTTGTGGA
TTCTGGGGAAATGTCTGAATCTTTCCCATATCGAACCAAAGCTCTGTTTGCTTTTGAGGAAATTGACGGC
GTGGATGTCTGCTTTTTTGGAATGCACGTCCAAGAATACGGCTCTGATTGCCCCCCTCCAAACACGAGGC
GTGTGTACATTTCTTATCTGGATAGTATTCATTTCTTCCGGCCACGTTGCCTCCGCACAGCCGTTTACCA
TGAGATCCTTATTGGATATTTAGAGTATGTGAAGAAATTAGGGTATGTGACAGGGCACATCTGGGCCTGT
CCTCCAAGTGAAGGAGATGATTACATCTTCCATTGCCACCCACCTGATCAAAAAATACCCAAGCCAAAAC
GACTGCAGGAGTGGTACAAAAAGATGCTGGACAAGGCGTTTGCAGAGCGGATCATCCATGACTACAAGGA
TATTTTCAAACAAGCAACTGAAGACAGGCTCACCAGTGCCAAGGAACTGCCCTATTTTGAAGGTGATTTC
TGGCCCAATGTGTTAGAAGAGAGCATTAAGGAACTAGAACAAGAAGAAGAGGAGAGGAAAAAGGAAGAGA
GCACTGCAGCCAGTGAAACCACTGAGGGCAGTCAGGGCGACAGCAAGAATGCCAAGAAGAAGAACAACAA
GAAAACCAACAAGAACAAAAGCAGCATCAGCCGCGCCAACAAGAAGAAGCCCAGCATGCCCAACGTGTCC
AATGACCTGTCCCAGAAGCTGTATGCCACCATGGAGAAGCACAAGGAGGTCTTCTTCGTGATCCACCTGC
ACGCTGGGCCTGTCATCAACACCCTGCCCCCCATCGTCGACCCCGACCCCCTGCTCAGCTGTGACCTCAT
GGATGGGCGCGACGCCTTCCTCACCCTCGCCAGAGACAAGCACTGGGAGTTCTCCTCCTTGCGCCGCTCC
AAGTGGTCCACGCTCTGCATGCTGGTGGAGCTGCACACCCAGGGCCAGGACCGCTTTGTCTACACCTGCA
ACGAGTGCAAGCACCACGTGGAGACGCGCTGGCACTGCACTGTGTGCGAGGACTACGACCTCTGCATCAA
CTGCTATAACACGAAGAGCCATGCCCATAAGATGGTGAAGTGGGGGCTGGGCCTGGATGACGAGGGCAGC
AGCCAGGGCGAGCCACAGTCAAAGAGCCCCCAGGAGTCACGCCGGGTGAGCATCCAGCGCTGCATCCAGT
CGCTGGTGCACGCGTGCCAGTGCCGCAACGCCAACTGCTCGCTGCCATCCTGCCAGAAGATGAAGCGGGT
GGTGCAGCACACCAAGGGCTGCAAACGCAAGACCAACGGGGGCTGCCCGGTGTGCAAGCAGCTCATCGCC
CTCTGCTGCTACCACGCCAAGCACTGCCAAGAAAACAAATGCCCCGTGCCCTTCTGCCTCAACATCAAAC
ACAAGCTCCGCCAGCAGCAGATCCAGCACCGCCTGCAGCAGGCCCAGCTCATGCGCCGGCGGATGGCCAC

CATGAACACCCGCAACGTGCCTCAGCAGAGTCTGCCTTCTCCTACCTCAGCACCGCCCGGACCCCCACA
CAGCAGCCCAGCACACCCCAGACGCCGCAGCCCCCTGCCCAGCCCCAACCCTCACCCGTGAGCATGTCAC
CAGCTGGCTTCCCCAGCGTGGCCCGGACTCAGCCCCCCACCACGGTGTCCACAGGGAAGCCTACCAGCCA
GGTGCCGGCCCCCCCACCCCCGGCCCAGCCCCCTCCTGCAGCGGTGGAAGCGGCTCGGCAGATCGAGCGT
GAGGCCCAGCAGCAGCAGCACCTGTACCGGGTGAACATCAACAACAGCATGCCCCCAGGACGCACGGGCA
TGGGGACCCCGGGGAGCCAGATGGCCCCCGTGAGCCTGAATGTGCCCCGACCCAACCAGGTGAGCGGGCC
CGTCATGCCCAGCATGCCTCCCGGGCAGTGGCAGCAGGCGCCCCTTCCCCAGCAGCAGCCCATGCCAGGC
TTGCCCAGGCCTGTGATATCCATGCAGGCCCAGGCGGCCGTGGCTGGGCCCCGGATGCCCAGCGTGCAGC
CACCCAGGAGCATCTCACCCAGCGCTCTGCAAGACCTGCTGCGGACCCTGAAGTCGCCCAGCTCCCCTCA
GCAGCAACAGCAGGTGCTGAACATTCTCAAATCAAACCCGCAGCTAATGGCAGCTTTCATCAAACAGCGC
ACAGCCAAGTACGTGGCCAATCAGCCCGGCATGCAGCCCCAGCCTGGCCTCCAGTCCCAGCCCGGCATGC
AACCCCAGCCTGGCATGCACCAGCAGCCCAGCCTGCAGAACCTGAATGCCATGCAGGCTGGCGTGCCGCG
GCCCGGTGTGCCTCCACAGCAGCAGGCGATGGGAGGCCTGAACCCCCAGGGCCAGGCCTTGAACATCATG
AACCCAGGACACAACCCCAACATGGCGAGTATGAATCCACAGTACCGAGAAATGTTACGGAGGCAGCTGC
TGCAGCAGCAGCAGCAACAGCAGCAGCAACAACAGCAGCAACAGCAGCAGCAGCAAGGGAGTGCCGGCAT
GGCTGGGGGCATGGCGGGGCACGGCCAGTTCCAGCAGCCTCAAGGACCCGGAGGCTACCCACCGGCCATG
CAGCAGCAGCAGCGCATGCAGCAGCATCTCCCCCTCCAGGGCAGCTCCATGGGCCAGATGGCGGCTCAGA
TGGGACAGCTTGGCCAGATGGGGCAGCCGGGGCTGGGGGCAGACAGCACCCCCAACATCCAGCAAGCCCT
GCAGCAGCGGATTCTGCAGCAACAGCAGATGAAGCAGCAGATTGGGTCCCCAGGCCAGCCGAACCCCATG
AGCCCCCAGCAACACATGCTCTCAGGACAGCCACAGGCCTCGCATCTCCCTGGCCAGCAGATCGCCACGT
CCCTTAGTAACCAGGTGCGGTCTCCAGCCCCTGTCCAGTCTCCACGGCCCCAGTCCCAGCCTCCACATTC
CAGCCCGTCACCACGGATACAGCCCCAGCCTTCGCCACACCACGTCTCACCCCAGACTGGTTCCCCCCAC
CCCGGACTCGCAGTCACCATGGCCAGCTCCATAGATCAGGGACACTTGGGGAACCCCGAACAGAGTGCAA
TGCTCCCCCAGCTGAACACCCCCAGCAGGAGTGCGCTGTCCAGCGAACTGTCCCTGGTCGGGGACACCAC
GGGGGACACGCTAGAGAAGTTTGTG**G**AGGGCTTGTAGCATTGTGAGAGCATCACCTTTTCCCTTTCATGT
TCTTGGACCTTTTGTACTGAAAATCCAGGCATCTAGGTTCTTTTTATTCCTAGATGGAACTGCGACTTCC
GAGCCATGGAAGGGTGGATTGATGTTTAAAGAAACAATACAAAGAATATATTTTTTTGTTAAAAACCAGT
TGATTTAAATATCTGGTCTCTCTCTTTGGTTTTTTTTTGGCGGGGGGGTGGGGGGGGTTCTTTTTTTTCC
GTTTTGTTTTTGTTTGGGGGGAGGGGGGTTTTGTTTGGATTCTTTTTGTCGTCATTGCTGGTGACTCATG
CCTTTTTTTAACGGGAAAAACAAGTTCATTATATTCATATTTTTTATTTGTATTTTCAAGACTTTAAACA
TTTATGTTTAAAAGTAAGAAGAAAAATAATATTCAGAACTGATTCCTGAAATAATGCAAGCTTATAATGT
ATCCCGATAACTTTGTGATGTTTCGGGAAGATTTTTTTCTATAGTGAACTCTGTGGGCGTCTCCCAGTAT
TACCCTGGATGATAGGAATTGACTCCGGCGTGCACACACGTACACACCCACACACATCTATCTATACATA
ATGGCTGAAGCCAAACTTGTCTTGCAGATGTAGAAATTGTTGCTTTGTTTCTCTGATAAAACTGGTTTTA
GACAAAAAATAGGGATGATCACTCTTAGACCATGCTAATGTTACTAGAGAAGAAGCCTTCTTTTCTTTCT
TCTATGTGAAACTTGAAATGAGGAAAAGCAATTCTAGTGTAAATCATGCAAGCGCTCTAATTCCTATAAA
TACGAAACTCGAGAAGATTCAATCACTGTATAGAATGGTAAAATACCAACTCATTTCTTATATCATATTG
TTAAATAAACTGTGTGCAACAGACAAAAAGGGTGGTCCTTCTTGAATTCATGTACATGGTATTAACACTT
AGTGTTCGGGGTTTTTTGTTATGAAAATGCTGTTTTCAACATTGTATTTGGACTATGCATGTGTTTTTTC
CCCATTGTATATAAAGTACCGCTTAAAATTGATATAAATTACTGAGGTTTTTAA**CATGTATTCTGTTCTT**
TAAGATCCCCTGTAAGAATGTTTAAGGTTTTTATTTATTTATATATATTTTTTGGTCTGTTCTTTGTAAA
AAAAAAAAAAAAAA

7) Entrez Gene ID 1655 = DDX5,DEAD (Asp-Glu-Ala-Asp) box polypeptide 5 = BC016027

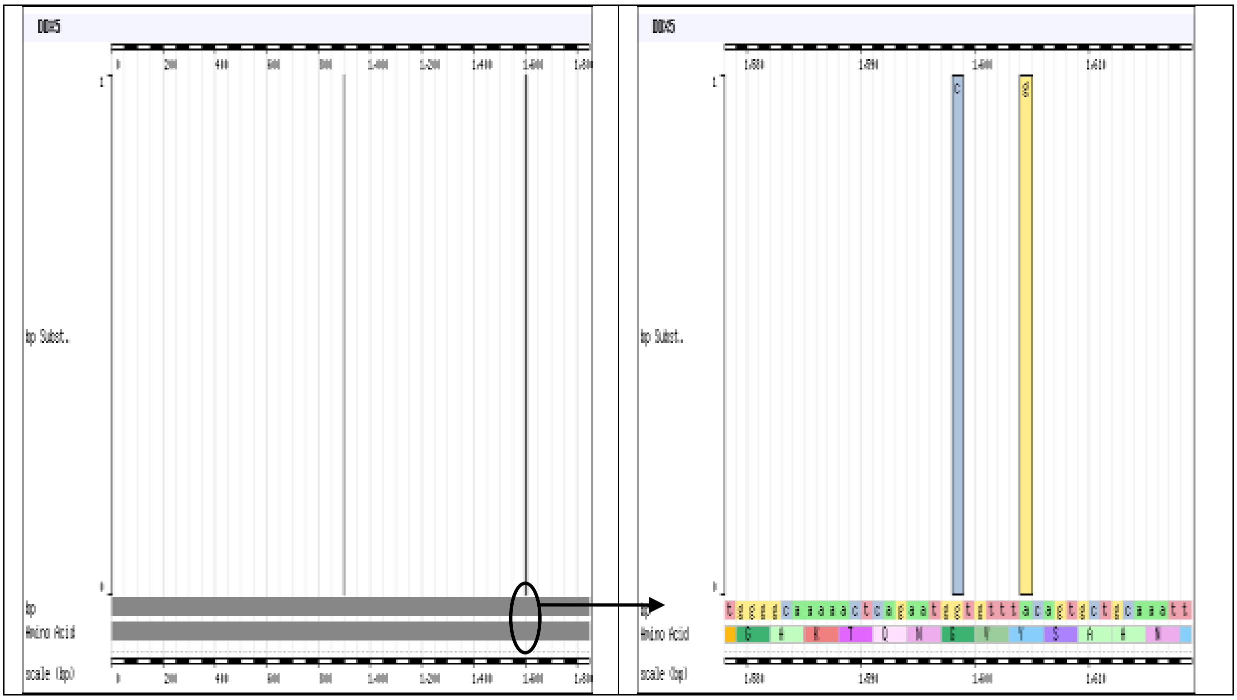

>gi|16359121|gb|BC016027.1| Homo sapiens DEAD (Asp-Glu-Ala-Asp) box polypeptide 5, mRNA (cDNA clone MGC:1516 IMAGE:3528578), complete cds
GTGCAGCTTCGGCTGGTGTCATCGGTGTCCTTCCTCCGCTGCCGCCCCCGCAAGGCTTCGCCGTCATCGA
GGCCATTTCCAGCGACTTGTCGCACGCTTTTCTATATACTTCGTTCCCCGCCAACCGCAACCATTGACGC
CATGTCGGGTTATTCGAGTGACCGAGACCGCGGCCGGGACCGAGGGTTTGGTGCACCTCGATTTGGAGGA
AGTAGGGCAGGGCCCTTATCTGGAAAGAAGTTTGGAAACCCTGGGGAGAAATTAGTTAAAAAGAAGTGGA
ATCTTGATGAGCTGCCTAAATTTGAGAAGAATTTTTATCAAGAGCACCCTGATTTGGCTAGGCGCACAGC
ACAAGAGGTGGAAACATACAGAAGAAGCAAGGAAATTACAGTTAGAGGTCACAACTGCCCGAAGCCAGTT
CTAAATTTTTATGAAGCCAATTTCCCTGCAAATGTCATGGATGTTATTGCAAGACAGAATTTCACTGAAC
CCACTGCTATTCAAGCTCAGGGATGGCCAGTTGCTCTAAGTGGATTGGATATGGTTGGAGTGGCACAGAC
TGGATCTGGGAAAACATTGTCTTATTTGCTTCCTGCCATTGTCCACATCAATCATCAGCCATTCCTAGAG
AGAGGCGATGGGCCTATTTGTTTGGTGCTGGCACCAACTCGGGAACTGGCCCAACAGGTGCAGCAAGTAG
CTGCTGAATATTGTAGAGCATGTCGCTTGAAGTCTACTTGTATCTACGGTGGTGCTCCTAAGGGACCACA
AATACGTGATTTGGAGAGAGGTGTGGAAATCTGTATTGCAACACCTGGAAGACTGATTGACTTTTTAGAG
TGTGGAAAAACCAATCTGAGAAGAACAACCTACCTTGTCCTTGATGAAGCAGATAGAATGCTTGATATGG
GCTTTGAACCCCAAATAAGGAAGATTGTGGATCAAATAAGACCTGATAGGCAAACTCTAATGTGGAGTGC
GACTTGGCCAAAAGAAGTAAGACAGCTTGCTGAAGATTTCCTGAAAGACTATATTCATATAAACATTGGT
GCACTTGAACTGAGTGCAAACCACAACATTCTTCAGATTGTGGATGTGTGTCATGACGTAGAAAAGGATG
AAAAACTTATTCGTCTAATGGAAGAGATCATGAGTGAGAAGGAGAATAAAACCATTGTTTTTGTGGAAAC
CAAAAGAAGATGTGATGAGCTTACCAGAAAAATGAGGAGAGATGGGTGGCCTGCCATGGGTATCCATGGT
GACAAGAGTCAACAAGAGCGTGACTGGGTTCTAAATGAATTCAAACATGGAAAAGCTCCTATTCTGATTG
CTACAGATGTGGCCTCCAGAGGGCTAGATGTGGAAGATGTGAAATTTGTCATCAATTATGACTACCCTAA
CTCCTCAGAGGATTATATTCATCGAATTGGAAGAACTGCTCGCAGTACCAAAACAGGCACAGCATACACT
TTCTTTACACCTAATAACATAAAGCAAGTGAGCGACCTTATCTCTGTGCTTCGTGAAGCTAATCAAGCAA
TTAATCCCAAGTTGCTTCAGTTGGTCGAAGACAGAGGTTCAGGTCGTTCCAGGGGTAGAGGAGGCATGAA
GGATGACCGTCGGGACAGATACTCTGCGGGCAAAAGGGGTGGATTTAATACCTTTAGAGACAGGGAAAAT
TATGACAGAGGTTACTCTAGCCTGCTTAAAAGAGATTTTGGGGCAAAAACTCAGAATG**G**TGTTT**A**CAGTG
CTGCAAATTACACCAATGGGAGCTTTGGAAGTAATTTTGTGTCTGCTGGTATACAGACCAGTTTTAGGAC

TGGTAATCCAACAGGGACTTACCAGAATGGTTATGATAGCACTCAGCAATACGGAAGTAATGTTCCAAAT
ATGCACAATGGTATGAACCAACAGGCATATGCATATCCTGCTACTGCAGCTGCACCTATGATTGGTTATC
CAATGCCAACAGGATATTCCCAATAAGACTTTAGAAGTATATGTAAATGTCTGTTTTTCATAATTGCTCT
TTATATTGTGTGTTATCTGACAAGATAGTTATTTAAGAAACATGGGAATTGCAGAAATGACTGCAGTGCA
GCAGTAATTATGGTGCACTTTTTCGCTATTTAAGTTGGATATTTCTCTACATTCCTGAAACAATTTTTAG
GTTTTTTTTGTACTAGAAAATGCAGGCAGTGTTTTCACAAAAGTAAATGTACAGTGATTTGAAATACAAT
AAATGAAGGCAATG**CATGGCCTTCCAATAAAAAAT**ATTTGAAGACTGAAAAAAAAAAAAAAAAAAAAAA
AAAAAAAAAAAAAAAAAAAAAAAAAAAAAAAAAAAAAAAAAAAAAAAAAAAAA

8) Entrez Gene ID 1662 = DDX10, DEAD (Asp-Glu-Ala-Asp) box polypeptide 10 = AB040537

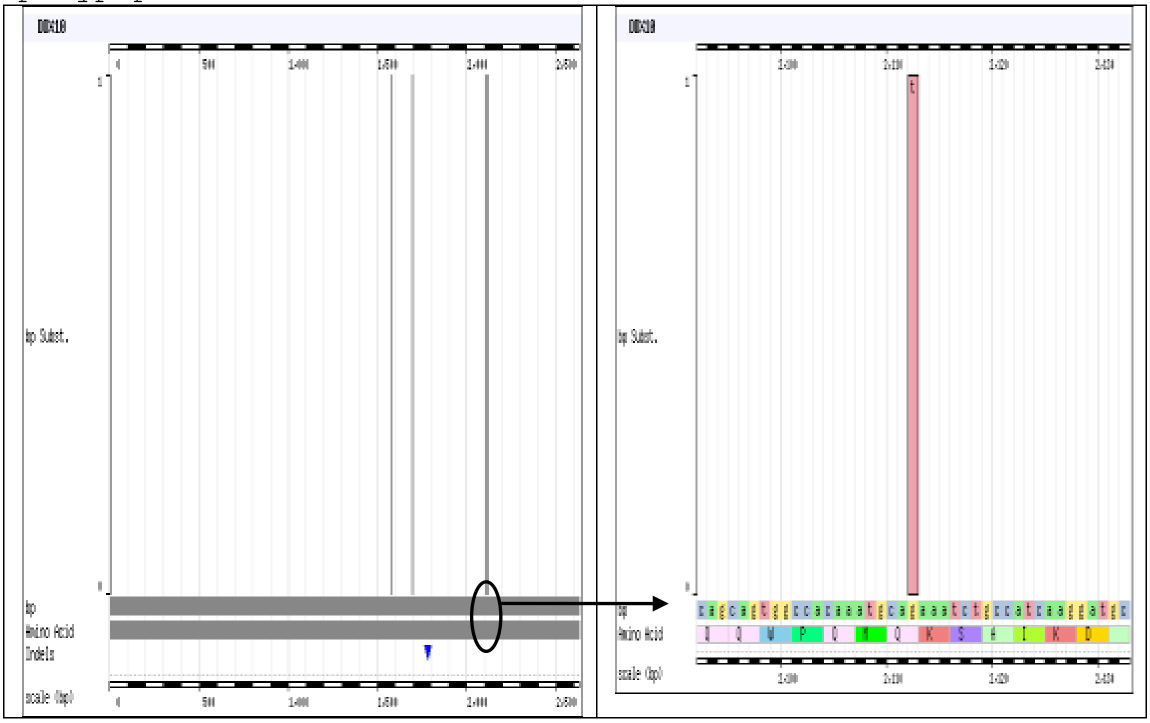

>gi|11414893|dbj|AB040537.1| Homo sapiens DDX10 mRNA for RNA helicase, complete cds
CCGTGAGTCTGGCCTTAGGTGTCTCGTGTCTGGGGTTGATCCGAGCTGTCGCCGCCGCCGCCGCAATGGG
CAAAACGGCCAACTCTCCGGGTTCGGGAGCCCGACCCGACCCGGTGCGGAGCTTCAATCGCTGGAAGAAA
AAACACAGCCATAGGCAGAACAAAAAGAAGCAGTTGAGGAAGCAACTGAAGAAACCCGAATGGCAGGTCG
AGCGCGAGAGTATCAGCCGCCTCATGCAGAACTATGAAAAGATAAATGTAAATGAAATCACAAGATTTTC
AGATTTTCCCTTGTCCAAAAAAACATTGAAAGGTTTGCAAGAAGCTCAGTACCGTTTGGTGACTGAGATA
CAGAAGCAGACCATTGGATTGGCTTTGCAAGGTAAAGATGTACTTGGAGCGGCCAAAACTGGATCTGGCA
AGACTCTGGCTTTTCTTGTTCCAGTGCTGGAAGCCTTATATCGTCTGCAATGGACTTCAACAGATGGGCT
GGGGGTTCTCATAATATCACCTACGAGAGAACTGGCCTATCAGACCTTTGAGGTTCTCCGAAAAGTAGGA
AAGAATCATGACTTCTCAGCTGGTCTCATCATTGGTGGAAAGGATCTAAAACACGAAGCTGAGAGGATCA
ACAACATAAATATACTCGTGTGCACACCAGGTCGGCTTCTTCAACACATGGATGAAACAGTATCTTTTCA
TGCTACCGACCTCCAAATGTTAGTTCTTGATGAAGCAGATAGAATCTTGGATATGGGCTTTGCTGATACC
ATGAATGCTGTTATTGAAAATCTCCCCAAGAAACGTCAGACTTTACTTTTCTCAGCAACACAAACTAAAT
CTGTAAAGGACCTTGCACGCTTGAGTTTGAAAAACCCTGAGTATGTCTGGGTTCATGAAAAAGCAAAATA
TAGCACCCCTGCCACTTTGGAACAGAACTACATAGTCTGTGAGCTGCAGCAAAAAATAAGTGTGCTGTAT
TCCTTTTTGAGAAGCCATCTGAAGAAGAAGAGCATTGTATTTTTTTCCAGTTGCAAAGAGGTCCAGTATC
TGTACCGAGTGTTTTGCCGGCTACGTCCTGGTGTTTCTATCCTTGCACTCCATGGTCGACAGCAGCAAAT
GAGAAGAATGGAAGTCTATAATGAGTTTGTCCGTAAGAGAGCTGCAGTACTCTTTGCTACTGATATTGCA
GCCAGGGGTCTGGATTTCCCGGCCGTGAATTGGGTTCTTCAGTTTGATTGTCCTGAGGATGCCAACACAT
ATATTCACAGAGCAGGTAGAACTGCCAGGTACAAAGAGGATGGTGAAGCTTTGCTAATTTTGCTACCCTC
AGAAAAAGCTATGGTGCAGCAGCTTCTTCAGAAGAAAGTACCTGTGAAGGAAATCAAAATCAATCCAGAA
AAACTTATAGATGTCCAGAAAAAATTGGAATCTATTTTAGCTCAAGATCAAGATTTAAAAGAAAGAGCTC
AAAGGTGTTTCGTCTCCTATGTACGATCTGTATATCTGATGAAGGATAAAGAAGTATTTGATGTGAGCAA

GTTACCTATACCTGAATATGCCCTGTCTCTTGGTCTTGCTGTGGCACCACGCGTAAGATTTCTTCAGAAA
ATGCAGAAACAACCCACCAAAGAATTGGTAAGGAGCCAAGCCGATAAAGTAATTGAGCCAAGGGCTCCCT
CCCTCACCAATGACGAAGTGGAAGAATTTAGAGCCTACTTCAATGAGAAAATGTCCATCCTTCAAAAAGG
TGGAAAAAGACTCGAAGGGACAGAGCACAGACAGGATAATGATACTGGTAATGAAGAACAGGAAGAAGAA
GAAGACGATGAAGAAGAAATGGAAGAGAAACTGGCAAAAGCAAAAGGATCTCAAGCCCCATCTCTTCCTA
ACACCAGTGAGGCACAGAAGATCAAGGAAGTTCCTACACAGTTCTTGGACAGAGATGAGGAGGAAGAAGA
TGCTGATTTCTTGAAGGTGAAGCGGCATAATGTGTTTGGATTGGACCTTAAAGACGAGAAAACATTACAG
AAGAAAGAACCTTCTAAATCCAGCATCAAGAAAAAAATGACCAAAGTTGCAGAAGCAAAAAAAGTAATGA
AGAGAAATTTTAAAGTGAATAAGAAGATAACATTTACTGATGAAGGGGAGTTGGTTCAGCAGTGGCCACA
AATG**C**AGAAATCTGCCATCAAGGATGCTGAGGAAGATGATGACACAGGTGGTATCAACTTACATAAAGCA
AAGGAAAGACTTCAGGAAGAGGACAAATTTGACAAAGAAGAATATAGGAAAAAAATTAAGGCAAAGCATC
GGGAGAAAAGACTGAAAGAAAGGGAAGCCAGAAGAGAAGCCAACAAGAGACAAGCAAAGGCCAAAGATGA
AGAGGAAGCCTTTCTGGATTGGAGTGATGATGATGATGATGATGATGATGGATTTGATCCAAGCACACTC
CCAGATCCAGATAAATACAGAAGCTCTGAAGATTCAGATAGTGAAGATATGGAAAATAAAATAAGTGATA
CCAAGAAGAAGCAGGGGATGAAGAAGAGGAGCAACAGTGAAGTGGAAGACGTGGGACCAACAAGTCATAA
CAGAAAGAAGGCCAGGTGGGACACTTTAGAGCCTTTGGATACCGGCCTGTCTTTAGCAGAGGATGAAGAG
CTGGTGTTACATCTGCTAAGAAGTCAAAGCTAAATACTTCCTGCGCCTGCCTTCTCCTTGAAACCTTGGT
TATGACTGCGTAGGCAAGAAGTTGAAAAACAGTTGATTTGGGGGCACTTAGGTACCATATGCCCCATTCC
CAAAGGGCACATTTCTGGATAGAAGCGATCGTATCTCCAAGTCCCTCTCACAGGACATGCTTTGTGCCAT
CACTGAGCATACTCAGATCGAGGGTGGATGATACCATTTCCTGACCCCGTTTTCCAGCATGTGTTCTGTT
AGATTTTTATCCATGGGTTCCTACGCCTTGTCATTGGAAACACTGCCTTTGTCTTACTGGCAAGTTCTGG
AGCTCTTGTGTCATTGTTAGAAATCCCTGTCTTGCTTACTGTACAGAAGTTTCTGTTGCTGTTAAAATTG
CT**CATGATTTCGACGTATTTAAT**ATTTTCAAAGAGACTATGATGGACCAGCCCTGAGAAAGAATGAGTAT
TTTTGAATTGAGATGATCAATAATAAACATATTTCCTATAAAAAAAAAAA

9) Entrez Gene ID 2033 = EP300, E1A binding protein p300 = U01877

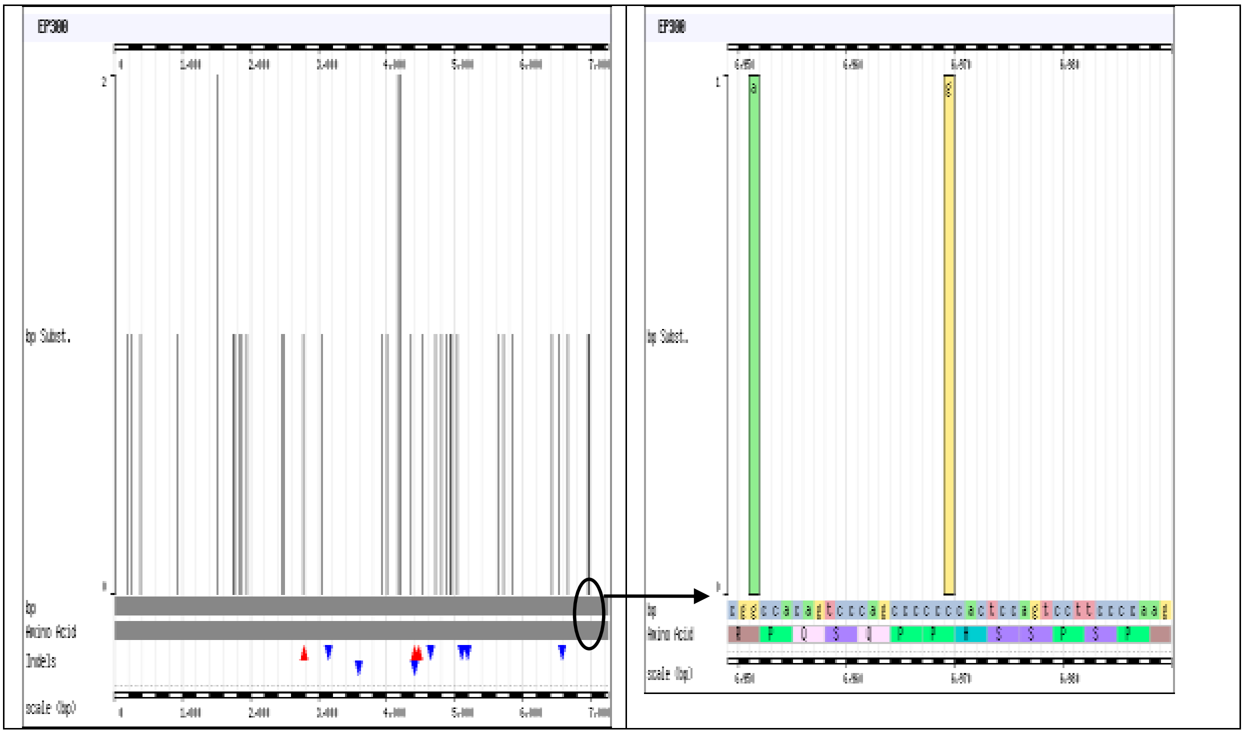

>gi|495300|gb|U01877.1|HSU01877 Human p300 protein mRNA, complete cds
CCTTGTTTGTGTGCTAGGCTGGGGGGGAGAGAGGGCGAGAGAGAGCGGGCGAGAGTGGGCAAGCAGGACG
CCGGGCTGAGTGCTAACTGCGGGACGCAGAGAGTGCGGAGGGGAGTCGGGTCGGAGAGAGGCGGCAGGGG
CCAGAACAGTGGCAGGGGGCCCGGGGCGCACGGGCTGAGGCGACCCCCAGCCCCCTCCCGTCCGCACACA
CCCCCACCGCGGTCCAGCAGCCGGGCCGGCGTCGACGCTAGGGGGGACCATTACATAACCCGCGCCCCGG

CCGTCTTCTCCCGCCGCCGCGGCGCCCGAACTGAGCCCGGGGCGGGCGCTCCAGCACTGGCCGCCGGCGT
GGGGCGTAGCAGCGGCCGTATTATTATTTCGCGGAAAGGAAGGCGAAGGAGGGGAGCGCCGGCGCGAGGA
GGGGCCGCCTGCGCCCGCCGCCGGAGCGGGGCCTCCTCGGTGGGCTCCGCGTCGGCGCGGGCGTGCGGGC
GGCGCTGCTCGGCCCGGCCCCCTCGGCCCTCTGGTCCGGCCAGCTCCGCTCCCGGCGTCCTTGCCGCGCC
TCCGCCGGCCGCCGCGCGATGTGAGGCGGCGGCGCCAGCCTGGCTCTCGGCTCGGGCGAGTTCTCTGCGG
CCATTAGGGGCCGGTGCGGCGGCGGCGCGGAGCGCGGCGGCAGGAGGAGGGTTCGGAGGGTGGGGGCGCA
GGCCCGGGAGGGGGCACCGGGAGGAGGTGAGTGTCTCTTGTCGCCTCCTCCTCTCCCCCCTTTTCGCCCC
CGCCTCCTTGTGGCGATGAGAAGGAGGAGGACAGCGCCGAGGAGGAAGAGGTTGATGGCGGCGGCGGAGC
TCCGAGAGACCTCGGCTGGGCAGGGGCCGGCCGTGGCGGGCCGGGGACTGCGCCTCTAGAGCCGCGAGTT
CTCGGGAATTCGCCGCAGCGGACCGGCCTCGGCGAATTTGTGCTCTTGTGCCCTCCTCCGGGCTTGGGCC
AGGCCGGCCCCTCGCACTTGCCCTTACCTTTTCTATCGAGTCCGCATCCCTCTCCAGCCACTGCGACCCG
GCGAAGAGAAAAAGGAACTTCCCCCACCCCCTCGGGTGCCGTCGGAGCCCCCCAGCCCACCCCTGGGTGC
GGCGCGGGGACCCCGGGCCGAAGAAGAGATTTCCTGAGGATTCTGGTTTTCCTCGCTTGTATCTCCGAAA
GAATTAAAAATGGCCGAGAATGTGGTGGAACCGGGGCCGCCTTCAGCCAAGCGGCCTAAACTCTCATCTC
CGGCCCTCTCGGCGTCCGCCAGCGATGGCACAGATTTTGGCTCTCTATTTGACTTGGAGCACGACTTACC
AGATGAATTAATCAACTCTACAGAATTGGGACTAACCAATGGTGGTGATATTAATCAGCTTCAGACAAGT
CTTGGCATGGTACAAGATGCAGCTTCTAAACATAAACAGCTGTCAGAATTGCTGCGATCTGGTAGTTCCC
CTAACCTCAATATGGGAGTTGGTGGCCCAGGTCAAGTCATGGCCAGCCAGGCCCAACAGAGCAGTCCTGG
ATTAGGTTTGATAAATAGCATGGTCAAAAGCCCAATGACACAGGCAGGCTTGACTTCTCCCAACATGGGG
ATGGGCACTAGTGGACCAAATCAGGGTCCTACGCAGTCAACAGGTATGATGAACAGTCCAGTAAATCAGC
CTGCCATGGGAATGAACACAGGGACGAATGCGGGCATGAATCCTGGAATGTTGGCTGCAGGCAATGGACA
AGGGATAATGCCTAATCAAGTCATGAACGGTTCAATTGGAGCAGGCCGAGGGCGACAGGATATGCAGTAC
CCAAACCCAGGCATGGGAAGTGCTGGCAACTTACTGACTGAGCCTCTTCAGCAGGGCTCTCCCCAGATGG
GAGGACAAACAGGATTGAGAGGCCCCCAGCCTCTTAAGATGGGAATGATGAACAACCCCAATCCTTATGG
TTCACCATATACTCAGAATCCTGGACAGCAGATTGGAGCCAGTGGCCTTGGTCTCCAGATTCAGACAAAA
ACTGTACTATCAAATAACTTATCTCCATTTGCTATGGACAAAAAGGCAGTTCCTGGTGGAGGAATGCCCA
ACATGGGTCAACAGCCAGCCCCGCAGGTCCAGCAGCCAGGTCTGGTGACTCCAGTTGCCCAAGGGATGGG
TTCTGGAGCACATACAGCTGATCCAGAGAAGCGCAAGCTCATCCAGCAGCAGCTTGTTCTCCTTTTGCAT
GCTCACAAGTGCCAGCGCCGGGAACAGGCCAATGGGGAAGTGAGGCAGTGCAACCTTCCCCACTGTCGCA
CAATGAAGAATGTCCTAAACCACATGACACACTGCCAGTCAGGCAAGTCTTGCCAAGTGGCACACTGTGC
ATCTTCTCGACAAATCATTTCACACTGGAAGAATTGTACAAGACATGATTGTCCTGTGTGTCTCCCCCTC
AAAAATGCTGGTGATAAGAGAAATCAACAGCCAATTTTGACTGGAGCACCCGTTGGACTTGGAAATCCTA
GCTCTCTAGGGGTGGGTCAACAGTCTGCCCCCAACCTAAGCACTGTTAGTCAGATTGATCCCAGCTCCAT
AGAAAGAGCCTATGCAGCTCTTGGACTACCCTATCAAGTAAATCAGATGCCGACACAACCCCAGGTGCAA
GCAAAGAACCAGCAGAATCAGCAGCCTGGGCAGTCTCCCCAAGGCATGCGGCCCATGAGCAACATGAGTG
CTAGTCCTATGGGAGTAAATGGAGGTGTAGGAGTTCAAACGCCGAGTCTTCTTTCTGACTCAATGTTGCA
TTCAGCCATAAATTCTCAAAACCCAATGATGAGTGAAAATGCCAGTGTGCCCTCCCTGGGTCCTATGCCA
ACAGCAGCTCAACCATCCACTACTGGAATTCGGAAACAGTGGCACGAAGATATTACTCAGGATCTTCGAA
ATCATCTTGTTCACAAACTCGTCCAAGCCATATTTCCTACGCCGGATCCTGCTGCTTTAAAAGACAGACG
GATGGAAAACCTAGTTGCATATGCTCGGAAAGTTGAAGGGGACATGTATGAATCTGCAAACAATCGAGCG
GAATACTACCACCTTCTAGCTGAGAAAATCTATAAGATCCAGAAAGAACTAGAAGAAAAACGAAGGACCA
GACTACAGAAGCAGAACATGCTACCAAATGCTGCAGGCATGGTTCCAGTTTCCATGAATCCAGGGCCTAA
CATGGGACAGCCGCAACCAGGAATGACTTCTAATGGCCCTCTACCTGACCCAAGTATGATCCGTGGCAGT
GTGCCAAACCAGATGATGCCTCGAATAACTCCACAATCTGGTTTGAATCAATTTGGCCAGATGAGCATGG
CCCAGCCCCCTATTGTACCCCGGCAAACCCCTCCTCTTCAGCACCATGGACAGTTGGCTCAACCTGGAGC
TCTCAACCCGCCTATGGGCTATGGGCCTCGTATGCAACAGCCTTCCAACCAGGGCCAGTTCCTTCCTCAG
ACTCAGTTCCCATCACAGGGAATGAATGTAACAAATATCCCTTTGGCTCCGTCCAGCGGTCAAGCTCCAG
TGTCTCAAGCACAAATGTCTAGTTCTTCCTGCCCGGTGAACTCTCCTATAATGCCTCCAGGGTCTCAGGG
GAGCCACATTCACTGTCCCCAGCTTCCTCAACCAGCTCTTCATCAGAATTCACCCTCGCCTGTACCTAGT
CGTACCCCCACCCCTCACCATACTCCCCCAAGCATAGGGGCTCAGCAGCCACCAGCAACAACAATTCCAG
CCCCTGTTCCTACACCACCAGCCATGCCACCTGGGCCACAGTCCCAGGCTCTACATCCCCCTCCAAGGCA
GACACCTACACCACCAACAACACAACTTCCCCAACAAGTGCAGCCTTCACTTCCTGCTGCACCTTCTGCT
GACCAGCCCCAGCAGCAGCCTCGCTCACAGCAGAGCACAGCAGCGTCTGTTCCTACCCCAAACGCACCGC
TGCTTCCTCCGCAGCCTGCAACTCCACTTTCCCAGCCAGCTGTAAGCATTGAAGGACAGGTATCAAATCC
TCCATCTACTAGTAGCACAGAAGTGAATTCTCAGGCCATTGCTGAGAAGCAGCCTTCCCAGGAAGTGAAG
ATGGAGGCCAAAATGGAAGTGGATCAACCAGAACCAGCAGATACGCAGCCGGAGGATATTTCAGAGTCTA
AAGTGGAAGACTGTAAAATGGAATCTACCGAAACAGAAGAGAGAAGCACTGAGTTAAAAACTGAAATAAA
AGAGGAGGAAGACCAGCCAAGTACTTCAGCTACCCAGTCATCTCCGGCTCCAGGACAGTCAAAGAAAAAG
ATTTTCAAACCAGAAGAACTACGACAGGCACTGATGCCAACATTGGAGGCACTTTACCGTCAGGATCCAG
AATCCCTTCCCTTTCGTCAACCTGTGGACCCTCAGCTTTTAGGAATCCCTGATTACTTTGATATTGTGAA
GAGCCCCATGGATCTTTCTACCATTAAGAGGAAGTTAGACACTGGACAGTATCAGGAGCCCTGGCAGTAT

GTCGATGATATTTGGCTTATGTTCAATAATGCCTGGTTATATAACCGGAAAACATCACGGGTATACAAAT
ACTGCTCCAAGCTCTCTGAGGTCTTTGAACAAGAAATTGACCCAGTGATGCAAAGCCTTGGATACTGTTG
TGGCAGAAAGTTGGAGTTCTCTCCACAGACACTGTGTTGCTACGGCAAACAGTTGTGCACAATACCTCGT
GATGCCACTTATTACAGTTACCAGAACAGGTATCATTTCTGTGAGAAGTGTTTCAATGAGATCCAAGGGG
AGAGCGTTTCTTTGGGGGATGACCCTTCCCAGCCTCAAACTACAATAAATAAAGAACAATTTTCCAAGAG
AAAAAATGACACACTGGATCCTGAACTGTTTGTTGAATGTACAGAGTGCGGAAGAAAGATGCATCAGATC
TGTGTCCTTCACCATGAGATCATCTGGCCTGCTGGATTCGTCTGTGATGGCTGTTTAAAGAAAAGTGCAC
GAACTAGGAAAGAAAATAAGTTTTCTGCTAAAAGGTTGCCATCTACCAGACTTGGCACCTTTCTAGAGAA
TCGTGTGAATGACTTTCTGAGGCGACAGAATCACCCTGAGTCAGGAGAGGTCACTGTTAGAGTAGTTCAT
GCTTCTGACAAAACCGTGGAAGTAAAACCAGGCATGAAAGCAAGGTTTGTGGACAGTGGAGAGATGGCAG
AATCCTTTCCATACCGAACCAAAGCCCTCTTTGCCTTTGAAGAAATTGATGGTGTTGACCTGTGCTTCTT
TGGCATGCATGTTCAAGAGTATGGCTCTGACTGCCCTCCACCCAACCAGAGGAGAGTATACATATCTTAC
CTCGATAGTGTTCATTTCTTCCGTCCTAAATGCTTGAGGACTGCAGTCTATCATGAAATCCTAATTGGAT
ATTTAGAATATGTCAAGAAATTAGGTTACACAACAGGGCATATTTGGGCATGTCCACCAAGTGAGGGAGA
TGATTATATCTTCCATTGCCATCCTCCTGACCAGAAGATACCCAAGCCCAAGCGACTGCAGGAATGGTAC
AAAAAAATGCTTGACAAGGCTGTATCAGAGCGTATTGTCCATGACTACAAGGATATTTTTAAACAAGCTA
CTGAAGATAGATTAACAAGTGCAAAGGAATTGCCTTATTTCGAGGGTGATTTCTGGCCCAATGTTCTGGA
AGAAAGCATTAAGGAACTGGAACAGGAGGAAGAAGAGAGAAAACGAGAGGAAAACACCAGCAATGAAAGC
ACAGATGTGACCAAGGGAGACAGCAAAAATGCTAAAAAGAAGAATAATAAGAAAACCAGCAAAAATAAGA
GCAGCCTGAGTAGGGGCAACAAGAAGAAACCCGGGATGCCCAATGTATCTAACGACCTCTCACAGAAACT
ATATGCCACCATGGAGAAGCATAAAGAGGTCTTCTTTGTGATCCGCCTCATTGCTGGCCCTGCTGCCAAC
TCCCTGCCTCCCATTGTTGATCCTGATCCTCTCATCCCCTGCGATCTGATGGATGGTCGGGATGCGTTTC
TCACGCTGGCAAGGGACAAGCACCTGGAGTTCTCTTCACTCCGAAGAGCCCAGTGGTCCACCATGTGCAT
GCTGGTGGAGCTGCACACGCAGAGCCAGGACCGCTTTGTCTACACCTGCAATGAATGCAAGCACCATGTG
GAGACACGCTGGCACTGTACTGTCTGTGAGGATTATGACTTGTGTATCACCTGCTATAACACTAAAAACC
ATGACCACAAAATGGAGAAACTAGGCCTTGGCTTAGATGATGAGAGCAACAACCAGCAGGCTGCAGCCAC
CCAGAGCCCAGGCGATTCTCGCCGCCTGAGTATCCAGCGCTGCATCCAGTCTCTGGTCCATGCTTGCCAG
TGTCGGAATGCCAATTGCTCACTGCCATCCTGCCAGAAGATGAAGCGGGTTGTGCAGCATACCAAGGGTT
GCAAACGGAAAACCAATGGCGGGTGCCCCATCTGCAAGCAGCTCATTGCCCTCTGCTGCTACCATGCCAA
GCACTGCCAGGAGAACAAATGCCCGGTGCCGTTCTGCCTAAACATCAAGCAGAAGCTCCGGCAGCAACAG
CTGCAGCACCGACTACAGCAGGCCCAAATGCTTCGCAGGAGGATGGCCAGCATGCAGCGGACTGGTGTGG
TTGGGCAGCAACAGGGCCTCCCTTCCCCCACTCCTGCCACTCCAACGACACCAACTGGCCAACAGCCAAC
CACCCCGCAGACGCCCCAGCCCACTTCTCAGCCTCAGCCTACCCCTCCCAATAGCATGCCACCCTACTTG
CCCAGGACTCAAGCTGCTGGCCCTGTGTCCCAGGGTAAGGCAGCAGGCCAGGTGACCCCTCCAACCCCTC
CTCAGACTGCTCAGCCACCCCTTCCAGGGCCCCCACCTACAGCAGTGGAAATGGCAATGCAGATTCAGAG
AGCAGCGGAGACGCAGCGCCAGATGGCCCACGTGCAAATTTTTCAAAGGCCAATCCAACACCAGATGCCC
CCGATGACTCCCATGGCCCCCATGGGTATGAACCCACCTCCCATGACCAGAGGTCCCAGTGGGCATTTGG
AGCCAGGGATGGGACCGACAGGGATGCAGCAACAGCCACCCTGGAGCCAAGGAGGATTGCCTCAGCCCCA
GCAACTACAGTCTGGGATGCCAAGGCCAGCCATGATGTCAGTGGCCCAGCATGGTCAACCTTTGAACATG
GCTCCACAACCAGGATTGGGCCAGGTAGGTATCAGCCCACTCAAACCAGGCACTGTGTCTCAACAAGCCT
TACAAAACCTTTTGCGGACTCTCAGGTCTCCCAGCTCTCCCCTGCAGCAGCAACAGGTGCTTAGTATCCT
TCACGCCAACCCCCAGCTGTTGGCTGCATTCATCAAGCAGCGGGCTGCCAAGTATGCCAACTCTAATCCA
CAACCCATCCCTGGGCAGCCTGGCATGCCCCAGGGGCAGCCAGGGCTACAGCCACCTACCATGCCAGGTC
AGCAGGGGGTCCACTCCAATCCAGCCATGCAGAACATGAATCCAATGCAGGCGGGCGTTCAGAGGGCTGG
CCTGCCCCAGCAGCAACCACAGCAGCAACTCCAGCCACCCATGGGAGGGATGAGCCCCCAGGCTCAGCAG
ATGAACATGAACCACAACACCATGCCTTCACAATTCCGAGACATCTTGAGACGACAGCAAATGATGCAAC
AGCAGCAGCAACAGGGAGCAGGGCCAGGAATAGGCCCTGGAATGGCCAACCATAACCAGTTCCAGCAACC
CCAAGGAGTTGGCTACCCACCACAGCCGCAGCAGCGGATGCAGCATCACATGCAACAGATGCAACAAGGA
AATATGGGACAGATAGGCCAGCTTCCCCAGGCCTTGGGAGCAGAGGCAGGTGCCAGTCTACAGGCCTATC
AGCAGCGACTCCTTCAGCAACAGATGGGGTCCCCTGTTCAGCCCAACCCCATGAGCCCCCAGCAGCATAT
GCTCCCAAATCAGGCCCAGTCCCCACACCTACAAGGCCAGCAGATCCCTAATTCTCTCTCCAATCAAGTG
CGCTCTCCCCAGCCTGTCCCTTCTCCACCG**G**CCACAGTCCCAGCCCCC**C**CACTCCAGTCCTTCCCCAAGGA
TGCAGCCTCAGCCTTCTCCACACCACGTTTCCCCACAGACAAGTTCCCCACATCCTGGACTGGTAGCTGC
CCAGGCCAACCCCATGGAACAAGGGCATTTTGCCAGCCCGGACCAGAATTCAATGCTTTCTCAGCTTGCT
AGCAATCCAGGCATGGCAAACCTCCATGGTGCAAGCGCCACGGACCTGGGACTCAGCACCGATAACTCAG
ACTTGAATTCAAACCTCTCACAGAGTACACTAGACATACACTAGAGACACCTTGTATTTTGGGAGCAAAA
AAATTATTTTCTCTTAACAAGACTTTTTGTACTGAAAACAATTTTTTTGAATCTTTCGTAGCCTAAAAGA
CAATTTTCCTTGGAACACATAAGAACTGTGCAGTAGCCGTTTGTGGTTTAAAGCAAA**CATGCAAGATGAA**
CCTGAGGGATGATAGAATACAAAGAATATATTTTTGTTATGGCTGGTTACCACCAGCCTTTCTTCCCCT
TTGTGTGTGTGGTTCAAGTGTGCACTGGGAGGAGGCTGAGGCCTGTGAAGCCAAACAATATGCTCCTGCC
TTGCACCTCCAATAGGTTTATTATTTTTTTTAAATTAATGAACATATGTAATATTAATGAACATATGTA

ATATTAATAGTTATTATTTACTGGTGCAGATGGTTGACATTTTTCCCTATTTTCCTCACTTTATGGAAGA
GTTAAAACATTTCTAAACCAGAGGACAAAAGGGGTTAATGTTACTTTGAAATTACATTCTATATATATAT
AAATATATATAAATATATATTAAAATACCAGTTTTTTTTCTCTGGGTGCAAAGATGTTCATTCTTTTAAA
AAATGTTTAAAAAAAA

10) Entrez Gene ID 2060 = EPS15, epidermal growth factor receptor pathway substrate 15 = BC054006

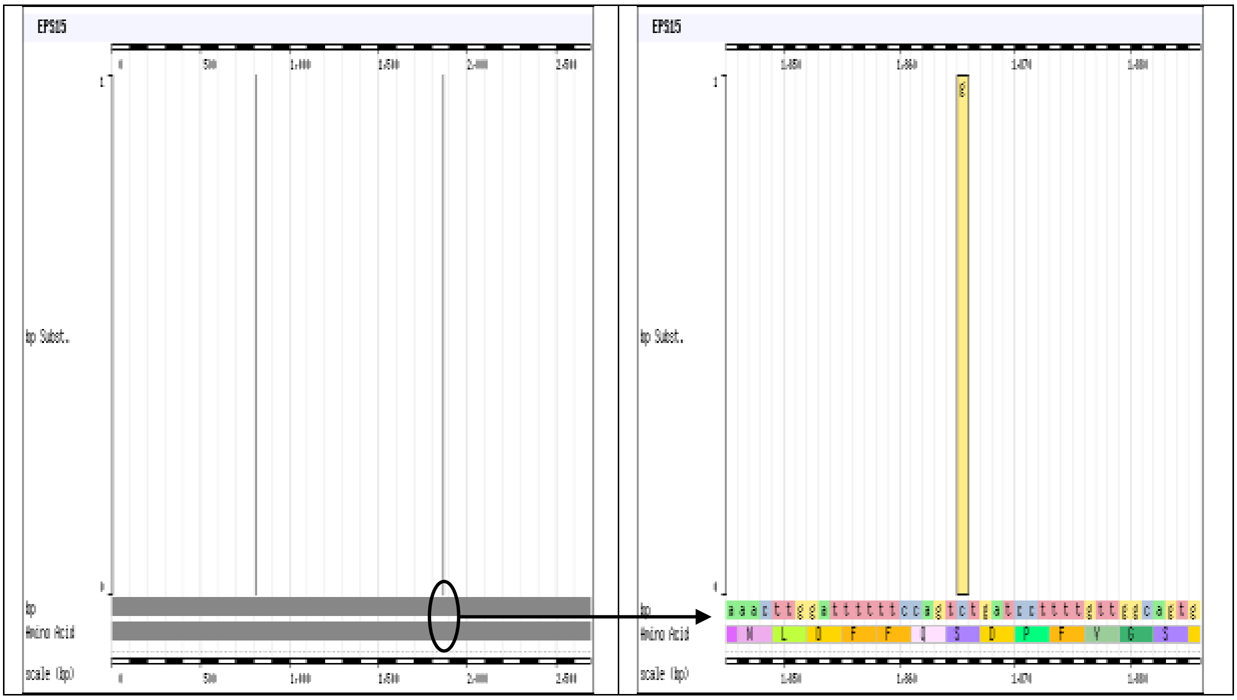

>gi|32450329|gb|BC054006.1| Homo sapiens epidermal growth factor receptor pathway substrate 15, mRNA (cDNA clone MGC:61536 IMAGE:6164633), complete cds

TGGAAACACCATGGCTGCGGCGGCCCAGCTCTCTCTGACACAGTTATCAAGTGGGAATCCTGTATATGAA
AAATACTATAGACAGGTTGATACAGGCAATACTGGAAGGGTGTTGGCTTCTGATGCTGCTGCTTTCCTGA
AAAAATCAGGGCTTCCAGACTTGATACTTGGAAAGATTTGGGATTTAGCCGACACAGATGGCAAAGGTAT
CCTGAACAAACAAGAATTCTTTGTTGCTTTGCGTCTTGTGGCATGTGCCCAGAATGGATTGGAAGTTTCA
CTAAGTAGTTTGAACCTGGCTGTTCCTCCACCAAGATTTCATGATACCAGTAGTCCTTTGCTAATCAGTG
GAACCTCTGCAGCTGAGCTCCCATGGGCTGTAAAACCTGAAGATAAGGCCAAATATGATGCAATATTTGA
TAGTTTAAGCCCAGTGAATGGATTTCTGTCTGGTGATAAAGTGAAACCAGTGTTGCTCAACTCTAAGTTA
CCTGTGGATATCCTTGGAAGAGTTTGGGAGTTGAGTGATATTGACCATGATGGAATGCTTGACAGAGATG
AGTTTGCAGTTGCTATGTTTTTGGTATACTGTGCACTGGAGAAAGAACCTGTGCCAATGTCCTTGCCTCC
AGCCTTGGTGCCACCATCTAAGAGAAAAACGTGGGTTGTATCCCCTGCAGAAAAAGCTAAATATGATGAA
ATCTTCCTGAAAACTGATAAAGATATGGACGGATTTGTGTCTGGATTGGAGGTCCGTGAAATATTCTTGA
AAACAGGTTTACCTTCTACCTTACTAGCCCATATATGGTCATTATGCGACACAAAGGACTGTGGGAAGCT
TTCAAAGGATCAGTTTGCCTTGGCTTTTCACTTAATCAGTCAGAAGTTAATCAAGGGCATTGATCCTCCT
CACGTTCTTACTCCTGAAATGATTCCACCATCAGACAGGGCCAGTTTACAAAAGAACATCATAGGATCAA
GTCCTGTTGCAGATTTCTCTGCTATTAAGGAACTAGATACTCTTAACAATGAAATAGTTGACCTACAGAG
GGAAAAGAATAATGTGGAACAGGACCTTAAGGAGAAGGAAGATACTATTAAACAGAGGACAAGTGAGGTT
CAGGATCTTCAAGATGAAGTTCAAAGGGAGAATACTAATCTGCAAAAACTACAGGCCCAGAAACAGCAGG
TACAGGAACTCCTTGATGAACTGGATGAGCAGAAAGCCCAGCTGGAGGAGCAACTCAAGGAAGTCAGAAA
GAAATGTGCTGAGGAGGCCCAACTGGCAAGAAGTAGTCCTGAACTACTGCCTTCTGGTGTGACTGATGAA
AATGAGGTGACTACAGCTGTTACTGAAAAAGTTTGTTCTGAACTCGACAATAATAGACATTCAAAAGAGG
AAGATCCATTTAATGTAGACTCAAGTTCGCTGACAGGTCCAGTTGCAGATACAAACTTGGATTTTTTCCA
GT**C**TGATCCTTTTGTTGGCAGTGATCCTTTCAAGGATGATCCTTTTGGAAAAATCGATCCATTTGGTGGT
GATCCTTTCAAAGGTTCAGATCCATTTGCATCAGACTGTTTCTTCAGGCAATCTACTGATCCTTTTGCCA
CTTCAAGCACTGACCCTTTCAGTGCAGCCAACAATAGCAGTATTACATCGGTAGAAACGTTGAAGCACAA

TGATCCTTTTGCTCCTGGTGGAACAGTTGTTGCAGCAAGCGATTCAGCCACAGACCCCTTTGCTTCTGTT
TTTGGGAATGAATCATTTGGAGGTGGATTTGCTGACTTCAGCACATTGTCAAAGGTCAACAATGAAGATC
CTTTTCGTTCAGCCACATCGAGCTCTGTCAGCAACGTAGTGATTACAAAAAATGTATTTGAGGAAACATC
GGTCAAAAGTGAAGATGAACCCCCAGCACTGCCACCAAAGATCGGAACTCCAACAAGACCCTGCCCTCTA
CCACCTGGGAAAAGATCCATCAACAAATTGGATTCTCCTGATCCCTTTAAACTGAATGATCCATTTCAGC
CTTTCCCAGGCAACGATAGCCCCAAAGAAAAAGATCCTGAAATGTTTTGTGATCCATTCACTTCTGCTAC
TACCACTACCAATAAAGAGGCTGATCCAAGCAATTTTGCCAACTTCAGTGCTTATCCCTCTGAAGAAGAT
ATGATCGAATGGGCCAAGAGGGAAAGTGAGAGAGAGGAAGAGCAGAGGCTTGCCCGACTAAATCAGCAGG
AACAAGAAGACTTAGAACTGGCTATTGCACTCAGCAAATCTGAGATATCAGAAGCATGAAGAATTCTCTT
GTTCTTTGGCAACAATATAGTATTCTTCTTCCTGAATACTGAAACTATTTACAATGTGTATCAAAACTAC
CTGTGAGCATGGGAATACAAAAGGTTTGAGATTCCTGTAAATGTGACAAAATTTTAGGATTTTTTTTTTT
CTTCATTACAGATTCGTCTTTTTTTTTTTTCTTATAAAAGCCGTAACCCAGTCAGACAAATTCACCTTCA
CTTAGGCCCCTGTTCTGGTATACATTTACTGTGAGCTTTTGCCTGCCTGTGCTATTTTACTTGTAAAGCT
AGAGCACCCAAGCTTCTGCCTTCTGGAATATAGAGAAATAGTTTCACCCTGCACTACCCTGTTCTGTAGT
TATTCTGATGATAGCCAGTGAGGTTCTTAAAGTTTGCAGTATTCTCCCCTGATTGGAATGGTTGAGTGAG
GGTAAGGGAAAGAATATCTTATTTCTTTTATGATTGGTGCAAATTGGCTAAAGTGCATTTTTAAATTTCC
TCTACTTAATTTGTTTTTCAGAGATAAGGAAAAATATTTTGCACAGATTTACTCCACTATGGAAAAGGGA
TGCTGTAGGTTGAACCATTATAGCCTCAGATTCGATCTTTTCCTAACTAAAAATATTAAAGCCTCATGTG
TGAAATAAATTTTTAAAAAGATTTATCTGGATTTAGAGAATTTTAGATCAACAGATACCTCTCAGTGTGT
TTGCTAATTAATAAAAATCAGTTTCTTACAAATAAAGTTTGTAAGAAAATGTTCATTTTAAGTGATAGAT
AGTGGAGAAAATTTATCACCTAAAATATACCCATCAGTATAAGGCAAGCAAAAGTCTTAACATGGCAGCC
ATTCTGCCTTTGCCGTGGCCCTGTCCTGTTTAGTTCTTAGTGGGTTAATTTTTGTACTTTTGCAGAAGAA
ACTTCAGCAAGCTAGAACTGGAAGGTACTTTAATTTTTCATATATATTTGTTTTTTTTTTTTTAATGAAG
GCTCATTTACTTGAAATGTAAAAACTTTCACTGAATACAAATAGAAAAAGTGATGTGTTTTATATCATAT
TGCTTTTTGTCCATCTTTGTGGTTTAGTTTATTTACTCACTTCATGTTTTTCACCTATAAAATTGTCAAG
CTAGCAAAAAAAACTCTTGTTTTTTTAATTGGGAGAGAAGAGACCTGCCAGATTATCAGACCTCTT**CATG**
TTAAAAGACCATCTCCTGTAAAACTGACCTAGTGGACAAGCTGAATTTGAAATAGACTGTGAAGTAAGCT
GTAACTGTCATTTTAATTTTGTTTAACACGGTTACTGACTTAGATGATGTATTAAATACCAAGATAAAGA
AAAATGCACCTAAAATCTAATTAGAATTCTCTGGGTCACCAAGTCAAGGTGGTATTGATCTGTGTTAATC
TGAGTAACTTATTGCCTAGCCTATAAATAAATTCCAAAATAAAAAAAAAAAAAAAAAAAAAAAAAAAAAA
AAAAAAAAAAAAAAAAAAAAAAAAAAAAAAAAAAAAAAAAAAAAAAAAAAAAAAAAAAAAAAAAAAA

11) Entrez Gene ID 2120 = ETV6, ets variant 6 = BC020284

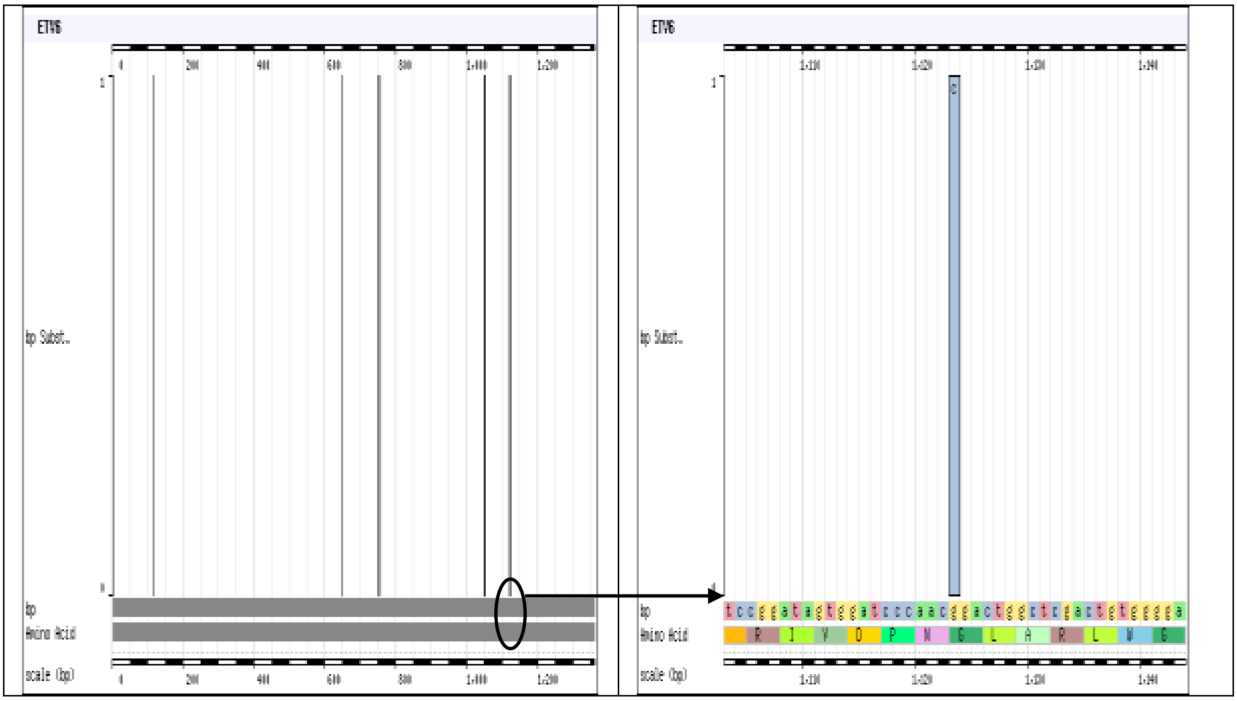

>gi|17946778|gb|BC020284.1| Homo sapiens ets variant 6, mRNA (cDNA clone IMAGE:4480192)
GAAGGCGCTCATTCCAATATAGTCTTTATTTCCCATTCAGATACAGGTTGAGCATCCCTAATCTGAACAG
TTAAAACCCCCAAATGCCCCAAAATCCAAACCTTCCTGAACGCTATGACACCATGAGTGGAAAATTCCAC
ACCTAACAAACACATTTGCTTTCTTATGGTTCAATGTACACAAACTGTTTTATATAGAAAATGATTTCAA
ATATCATAAAATTACCTTCAGGCTATGTGTATAAAGTATATATGAGCCATAAATGAATTTTGTGTTTAGA
CTTTGTGTCCATCCCCAAGATCTCTCATTTTATATATATATATATATATATATATATATATATATATACA
CACACACACACACATACACAAATATTCCAGGATACAAAAAAAAACATTTAAAAATCCGAGACCCAGAACA
CTTCTGGTCCCAAGCATTTCAGATAAGGGATATCAATCTGTACTACCAATAAGGATTTCGTAATTCCCCT
AACTGCAAATGTCCTCTTCATTTGTTCTTTATGAGAAAACCCGGGTAGTGCCAGCACCTGGATACAGTAT
TTACACCCTGCAGACCCTAAAGATTTCAGATTCAGTTAGCAAACCTTGATGAAGCACCTGCTGGACACTG
AGGGACCCAAAGCTCAATCAGCCATAATCCCTGCTTTCAGAGTTTATATTGTACCTGCCTAATCCACCCG
GCGTGACTCATTTCAACACTAAGTACTAGGGGTGTTGTCAGGAGACAAATCTGAAGTCAGGAGAGGAAAA
TGCAAAGGAGCCCTGCCGTGTGATGGATGTGCATTCTCACTTGGGTCTTGAAGTTCTCATTCCTACATCT
CAAGCTAGCCAGGCAGTCTCCTCTCTATCAGAAGAAAGCACTGGTAATTGGCTAGACTGGCTATGTTGAA
GGTAACATGAACTCTAAGATCTTGACCCAGGGCGACTTGGTTTTGCTTAAGGTGGCATCACCAATGTTCC
AAATCCTTTAGGGAGATGAGGGTATCCCCACAGAAAAAGAGGAATAATAGACCAATGGATTTTCTCCTTT
CACCAGTATGTTTGGAACCCTCTGATCCAATGTCCTTTGATACTGATCTCTTGTCCAAATGAGAATGTCG
CTTTAGCTGAAATTCAAATGGCTGTGACAATTTACCGAAATGATGAAGTAACCACCATTCCCACCTTTCA
CTGCCTAGGCTCCAAGTCTGAATACATTTTTGAAATAGGAACTCCCTTTTGCAAAAAAGAAACCTGGGTG
TCAGGGAGGTGAAGTGACTTGCCCTAGGAGCAGACAGCATGCCAAGAATGGAATTAGGCTCAGGATCCAG
CCTGGGCTCACCCTGTGTGGCTCATTCCCACCCAGGAAACTGAAGATAAAAGATTTGGGAAAACACACCA
AGAAAAAGGGGCAGTTTTCTTTGCCCAAGCATTTGGTGCTAGTTAGAGGCTGTTCACTCTCTCCTGCTCC
TCTTCGGAGTAGAAATAAAGGCTGTGACACAAGGAAGCCAGTGGGGTGGGAGGGAGGCACCATAATCCCT
CCCTAAAACCCACAGAAGACTAACCTGATACTCTTTTGACCCAACTGCATCAACACTAAACAGCTGCAGA
CCCCCTGAATCTTTCACA**CATGCCAAGTGAACATTCTTG**ATGATTTCTCTTTGTGACCGCAACCACCTGC
AAACCAGAACGACTCTAGAATTTCCTTCCCCGCCCCCCTTTTTGTTTAGTTTCTAATCTCTTGTTTATGA
GGTGTGGGGTTTATAAGGGACTGAATCAAATGAATGTAAAAAAAAAAAAAAAAAAAAAAAAAAAAAAAA

12) Entrez Gene ID 2130 = EWSR1,Ewing sarcoma breakpoint region 1 = BC011048

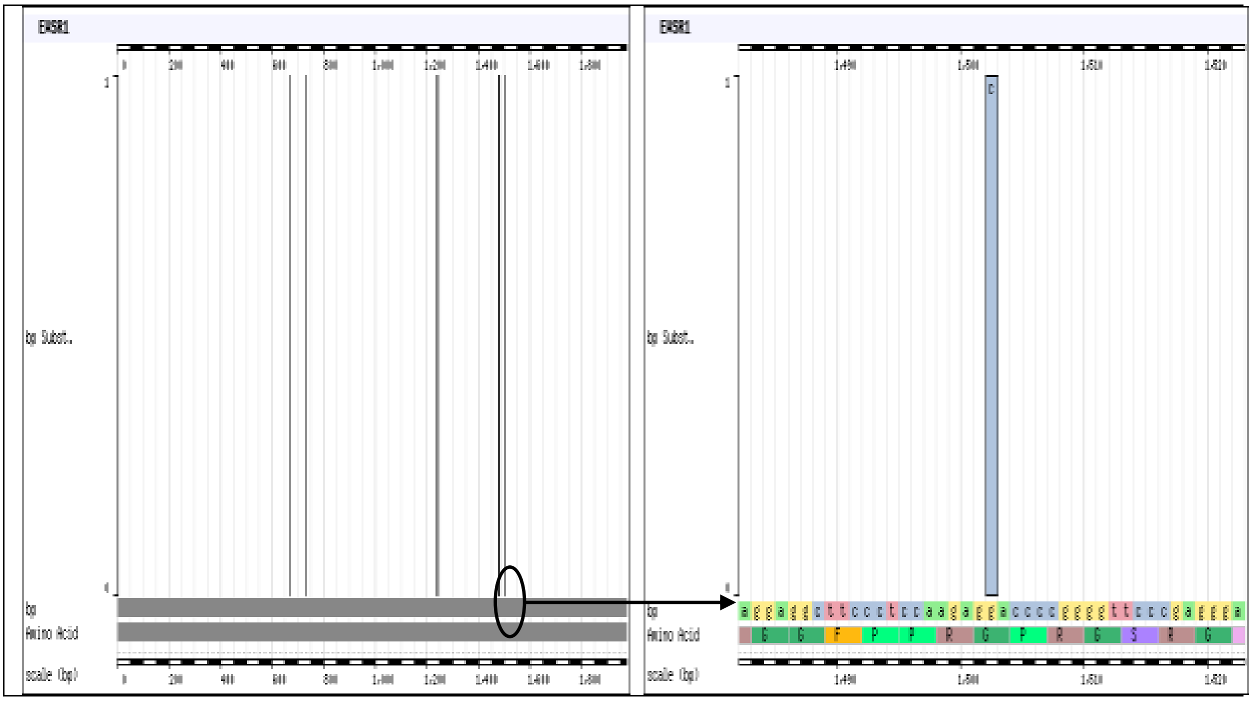

>gi|15029674|gb|BC011048.1| Homo sapiens Ewing sarcoma breakpoint region 1, mRNA (cDNA clone MGC:16900 IMAGE:4340116), complete cds
CTAGTTCTAGATCGCGAGCGGCGGAAAATGGCGTCCACGGATTACAGTACCTATAGCCAAGCTGCAGCGC

AGCAGGGCTACAGTGCTTACACCGCCCAGCCCACTCAAGGATATGCACAGACCACCCAGGCATATGGGCA
ACAAAGCTATGGAACCTATGGACAGCCCACTGATGTCAGCTATACCCAGGCTCAGACCACTGCAACCTAT
GGGCAGACCGCCTATGCAACTTCTTATGGACAGCCTCCCACTGGTTATACTACTCCAACTGCCCCCCAGG
CATACAGCCAGCCTGTCCAGGGGTATGGCACTGGTGCTTATGATACCACCACTGCTACAGTCACCACCAC
CCAGGCCTCCTATGCAGCTCAGTCTGCATATGGCACTCAGCCTGCTTATCCAGCCTATGGGCAGCAGCCA
GCAGCCACTGCACCTACAAGACCGCAGGATGGAAACAAGCCCACTGAGACTAGTCAACCTCAATCTAGCA
CAGGGGGTTACAACCAACCCAGCCTAGGATATGGACAGAGTAACTACAGTTATCCCCAGGTACCTGGGAG
CTACCCCATGCAGCCAGTCACTGCACCTCCATCCTACCCTCCTACCAGCTATTCCTCTACACAGCCGACT
AGTTATGATCAGAGCAGTTACTCTCAGCAGAACACCTATGGGCAACCGAGCAGCTATGGACAGCAGAGTA
GCTATGGTCAACAAAGCAGCTATGGGCAGCAGCCTCCCACTAGTTACCCACCCCAAACTGGATCCTACAG
CCAAGCTCCAAGTCAATATAGCCAACAGAGCAGCAGCTACGGGCAGCAGAGTTCATTCCGACAGGACCAC
CCCAGTAGCATGGGTGTTTATGGGCAGGAGTCTGGAGGATTTTCCGGACCAGGAGAGAACCGGAGCATGA
GTGGCCCTGATAACCGGGGCAGGGGAAGAGGGGGATTTGATCGTGGAGGCATGAGCAGAGGTGGGCGGGG
AGGAGGACGCGGTGGAATGGGCGCTGGAGAGCGAGGTGGCTTCAATAAGCCTGGTGGACCCATGGATGAA
GGACCAGATCTTGATCTAGGCCCACCTGTAGATCCAGATGAAGACTCTGACAACAGTGCAATTTATGTAC
AAGGATTAAATGACAGTGTGACTCTAGATGATCTGGCAGACTTCTTTAAGCAGTGTGGGGTTGTTAAGAT
GAACAAGAGAACTGGGCAACCCATGATCCACATCTACCTGGACAAGGAAACAGGAAAGCCCAAAGGCGAT
GCCACAGTGTCCTATGAAGACCCACCCACTGCCAAGGCTGCCGTGGAATGGTTTGATGGGAAAGATTTTC
AAGGGAGCAAACTTAAAGTCTCCCTTGCTCGGAAGAAGCCTCCAATGAACAGTATGCGGGGTGGTCTGCC
ACCCCGTGAGGGCAGAGGCATGCCACCACCACTCCGTGGAGGTCCAGGAGGCCCAGGAGGTCCTGGGGGA
CCCATGGGTCGCATGGGAGGCCGTGGAGGAGATAGAGGAGGCTTCCCTCCAAGAG**G**ACCCCGGGGTTCCC
GAGGGAACCCCTCTGGAGGAGGAAACGTCCAGCACCGAGCTGGAGACTGGCAGTGTCCCAATCCGGGTTG
TGGAAACCAGAACTTCGCCTGGAGAACAGAGTGCAACCAGTGTAAGGCCCCAAAGCCTGAAGGCTTCCTC
CCGCCACCCTTTCCGCCCCCGGGTGGTGATCGTGGCAGAGGTGGCCCTGGTGGCATGCGGGGAGGAAGAG
GTGGCCTCATGGATCGTGGTGGTCCCGGTGGAATGTTCAGAGGTGGCCGTGGTGGAGACAGAGGTGGCTT
CCGTGGTGGCCGGGGCATGGACCGAGGTGGCTTTGGTGGAGGAAGACGAGGTGGCCCTGGGGGGCCCCCT
GGACCTTTGATGGAACAGATGGGAGGAAGAAGAGGAGGACGTGGAGGACCTGGAAAAATGGATAAAGGCG
AGCACCGTCAGGAGCGCAGAGATCGGCCCTACTAGATGCAGAGACCCCGCAGAGCTGCATTGACTACCAG
ATTTATTTTTAAACCAGAAAATGTTTTAAATTTATAATTCCATATTTATAATGTTGGCCACAACATAAT
GATTATTCCTTGTCTGTACTTTAGTATTTTTCACCATTTGTGAAGAAACATTAAAACAAGTTAAATGGTA
GTGTGCGGAGTTTTTTTTTCTTCCTTCTTTTAAAAATGGTTGTTTAAGACTTTAACAATGGGAACCCCTT
GTGAGCATGCTCAGTATCATTGTGGAGAACCAAGAGGGCCTCTTAACTGTAACAATGTT**CATGGTTGTGA**
TGTTTTTTTTTTTTTTTTTAAATAAAATTCCAAATGTTAAAAAAAAAAAAAAAA

13) Entrez Gene ID 2181 = ACSL3, acyl-CoA synthetase long-chain family member 3 = D89053

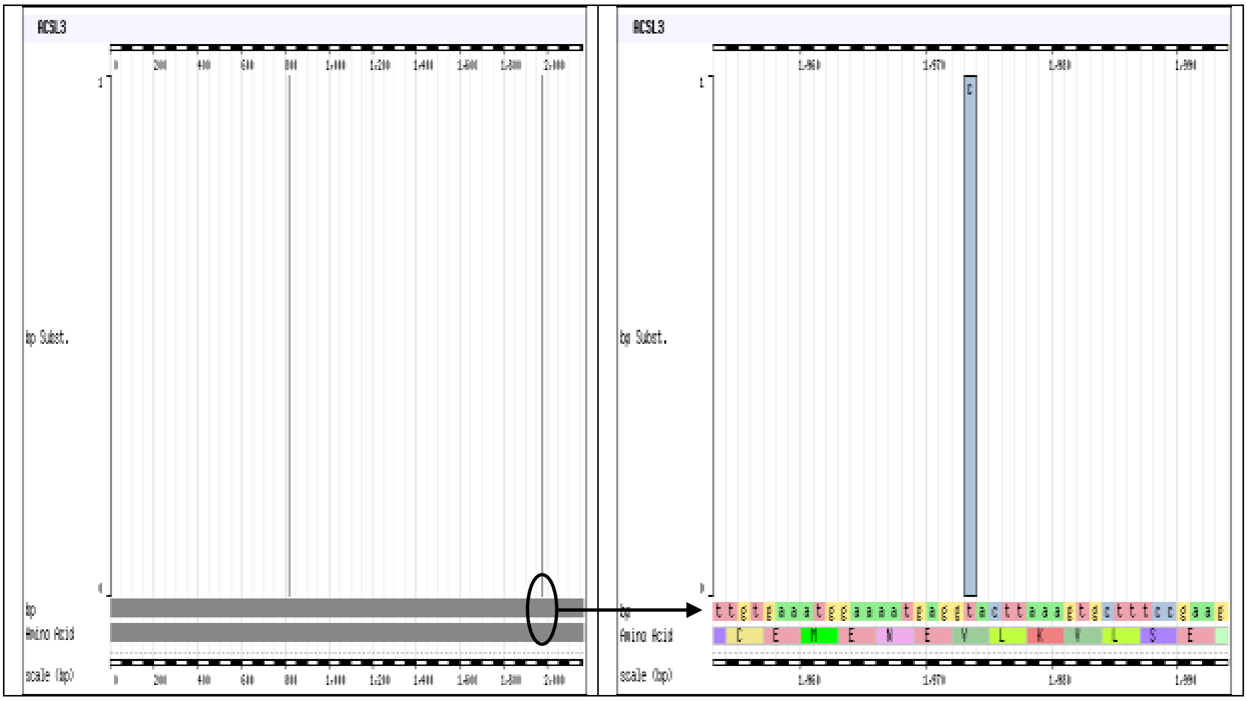

>gi|4165017|dbj|D89053.1| Homo sapiens mRNA for Acyl-CoA synthetase 3, complete cds

GAATTCGTTGTTGGGAAGGACTGGGGAAACAGCTGTAACATTTGCCACCCTCAGAAGCTGCTGGTCCTGT
GTCACACCACCTTAGCCTCTTGATCGAGGAAGATTCTCGCTGAAGTCTGTTAATTCTACTTTTTGAGTAC
TTATGAATAACCACGTGTCTTCAAAACCATCTACCATGAAGCTAAAACATACCATCAACCCTATTCTTTT
ATATTTTATACATTTTCTAATATCACTTTATACTATTTTAACATACATTCCGTTTTATTTTTTCTCCGAG
TCAAGACAAGAAAAATCAAACCGAATTAAAGCAAAGCCTGTAAATTCAAAACCTGATTCTGCATACAGAT
CTGTTAATAGTTTGGATGGTTTGGCTTCAGTATTATACCCTGGATGTGATACTTTAGATAAAGTTTTTAC
ATATGCAAAAAACAAATTTAAGAACAAAAGACTCTTGGGAACACGTGAAGTTTTAAATGAGGAAGATGAA
GTACAACCAAATGGAAAAATTTTTAAAAAGGTTATTCTTGGACAGTATAATTGGCTTTCCTATGAAGATG
TCTTTGTTCGAGCCTTTAATTTTGGAAATGGATTACAGATGTTGGGTCAGAAACCAAAGACCAACATCGC
CATCTTCTGTGAGACCAGGGCCGAGTGGATGATAGCTGCACAGGCGTGTTTTATGTATAATTTTCAGCTT
GTTACATTATATGCCACTCTAGGAGGTCCAGCCATTGTTCATGCATTAAATGAAACAGAGGTGACCAACA
TCATTACTAGTAAAGAACTCTTACAAACAAAGTTGAAGGATATAGTTTCTTTGGTCCCACGCCTGCGGCA
CATCATCACTGTTGATGGAAAGCCACCGACCTGGTCCGACTTCCCCAAGGGCATCATTGTGCATACCATG
GCTGCAGTGGAGGCCCTGGGAGCCAAGGCCAGCATGGAAAACCAACCTCATAGCAAACCATTGCCCTCAG
ATATTGCAGTAATCATGTACACAAGTGGATCCACAGGACTTCCAAAGGGAGTCATGATCTCACATAGTAA
CATTATTGCTGGTATAACTGGGATGGCAGAAAGGATTCCAGAACTAGGAGAGGAAGATGTCTACATTGGA
TATTTGCCTCTGGCCCATGTTCTAGAATTAAGTGCTGAGCTTGTCTGTCTTTCTCACGGATGCCGCATTG
GTTACTCTTCACCACAGACTTTAGCAGATCAGTCTTCAAAAATTAAAAAAGGAAGCAAAGGGGATACATC
CATGTTGAAACCAACACTGATGGCAGCAGTTCCGGAAATCATGGATCGGATCTACAAAAATGTCATGAAT
AAAGTCAGTGAAATGAGTAGTTTTCAACGTAATCTGTTTATTCTGGCCTATAATTACAAAATGGAACAGA
TTTCAAAAGGACGTAATACTCCACTGTGCGACAGCTTTGTTTTCCGGAAAGTTCGAAGCTTGCTAGGGGG
AAATATTCGTCTCCTGTTGTGTGGTGGCGCTCCACTTTCTGCAACCACGCAGCGATTCATGAACATCTGT
TTCTGCTGTCCTGTTGGTCAGGGATACGGGCTCACTGAATCTGCTGGGGCTGGAACAATTTCCGAAGTGT
GGGACTACAATACTGGCAGAGTGGGAGCACCATTAGTTTGCTGTGAAATCAAATTAAAAAACTGGGAGGA
AGGTGGATACTTTAATACTGATAAGCCACACCCCAGGGGTGAAATTCTTATTGGGGGCCAAAGTGTGACA
ATGGGGTACTACAAAAATGAAGCAAAAACAAAAGCTGATTTCTCTGAAGATGAAAATGGACAAAGGTGGC
TCTGTACTGGGGATATTGGAGAGTTTGAACCCGATGGATGCTTAAAGATTATTGATCGTAAAAAGGACCT
TGTAAAACTACAGGCAGGGGAATATGTTTCTCTTGGGAAAGTAGAGGCAGCTTTGAAGAATCTTCCACTA
GTAGATAACATTTGTGCATATGCAAACAGTTATCATTCTTATGTCATTGGATTTGTTGTGCCAAATCAAA
AGGAACTAACTGAACTAGCTCGAAAGAAAGGACTTAAAGGGACTTGGGAGGAGCTGTGTAACAGTTGTGA
AATGGAAAATGAGG**T**ACTTAAAGTGCTTTCCGAAGCTGCTATTTCAGCAAGTCTGGAAAAGTTTGAAATT
CCAGTAAAAATTCGTTTGAGTCCTGAACCGTGGACCCCTGAAACTGGTCTGGTGACAGATGCCTTCAAGC
TGAAACGCAAAGAGCTTAAAACACATTACCAGGCGGACATTGAGCGAATGTATGGAAGAAAATAATTATT
CTCTTCTGGCATCAGTTTGCTACAGTGAGCTCACATCAAATAGGAAAATACTTGAAATGCATGTCTCAAG
CTGCAAGGCAAACTCCATTCCTCATATTAAACTATTACTTCTCATGACGTCACCATTTTTAACTGACAGG

ATTAGTAAAACATTAAGACAGCAAACTTGTGTCTGTCTCTTCTTTCATTTTCCCCGCCACCAACTTACTT
TACCACCTATGACTGTACTTGTCAGTATGAGAATTTTTCTGAATCATATTGGGGAAGCAGTGATTTTAAA
ACCTCAAGTTTTTAAA**CATGATTTATATGTTCTGTAT**AATGTTCAGTTTGTAACTTTTTAAAAGTTTGGA
TGTATAGAGGGATAAATAGGAAATATAAGAATTGGTTATTTGGGGGCTTTTTTACTTACTGTATTTAAAA
ATACAAGGGTATTGATATGAAATTATGTAAATTTCAAATGCTTATGAATCAAATCATTGTTGAACAAAAG
ATTTGTTGCTGTGTAATTATTGTCTTGTATGCATTTGAGAGAAATAAATATACCCATACTTATGTTTTAA
GAAGTTGAGATCTTGTGAAAAAAAAAAAAAAAAAA

14) Entrez Gene ID 2313 = FLI1, Friend leukemia virus integration 1 = M98833

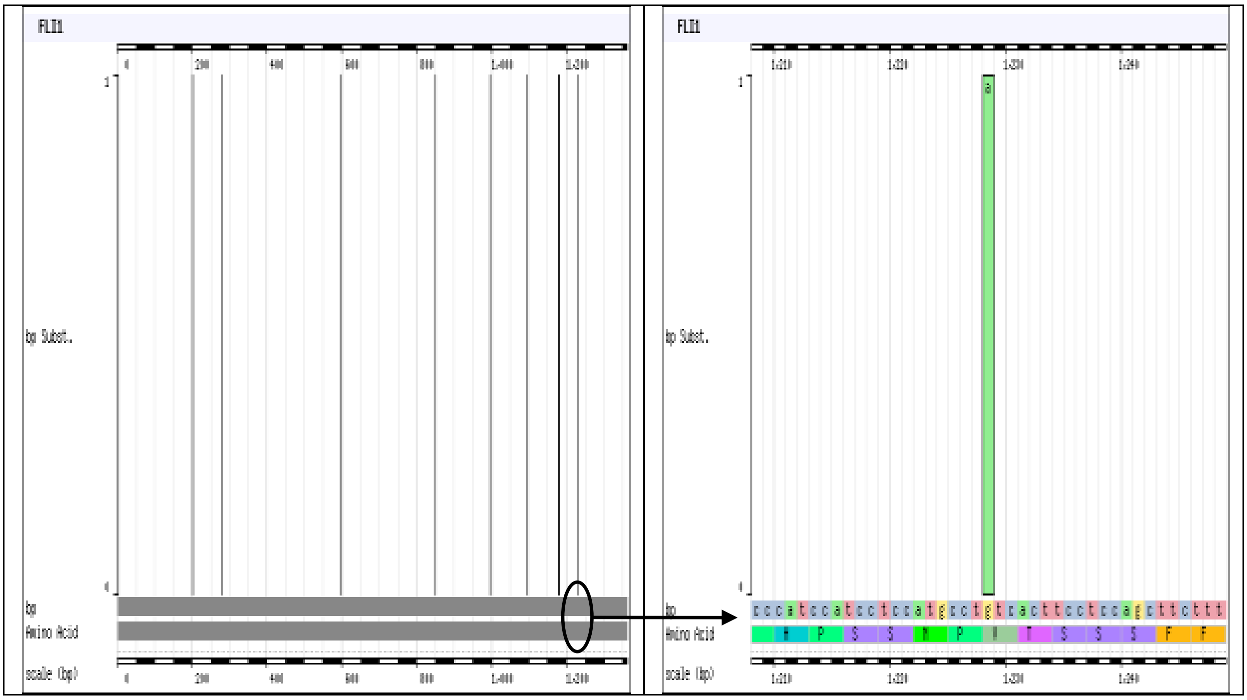

>gi|7025922|gb|M98833.3|HUMERGBFLI Homo sapiens ERGB transcription factor mRNA, complete cds
GAATTCCCAAACGTGCACAGGGGAGTGAGGGCAGGGCGCTCGCAGGGGGCACGCAGGGAGGGCCCAGGGC
GCCAGGGAGGCCGCGCCGGGCTAATCCGAAGGGGCTGCGAGGTCAGGCTGTAACCGGGTCAATGTGTGGA
ATATTGGGGGGCTCGGCTGCAGACTTGGCCAAATGGACGGGACTATTAAGGAGGCTCTGTCGGTGGTGAG

CGACGACCAGTCCCTCTTTGACTCAGCGTACGGAGCGGCAGCCCATCTCCCCAAGGCCGACATGACTGCC
TCGGGGAGTCCTGACTACGGGCAGCCCCACAAGATCAACCCCCTCCCACCACAGCAGGAGTGGATCAATC
AGCCAGTGAGGGTCAACGTCAAGCGGGAGTATGACCACATGAATGGATCCAGGGAGTCTCCGGTGGACTG
CAGCGTTAGCAAATGCAGCAAGCTGGTGGGCGGAGGCGAGTCCAACCCCATGAACTACAACAGCTATATG
GACGAGAAGAATGGCCCCCCTCCTCCCAACATGACCACCAACGAGAGGAGAGTCATCGTCCCCGCAGACC
CCACACTGTGGACACAGGAGCATGTGAGGCAATGGCTGGAGTGGGCCATAAAGGAGTACAGCTTGATGGA
GATCGACACATCCTTTTTCCAGAACATGGATGGCAAGGAACTGTGTAAAATGAACAAGGAGGACTTCCTC
CGCGCCACCACCCTCTACAACACGGAAGTGCTGTTGTCACACCTCAGTTACCTCAGGGAAAGTTCACTGC
TGGCCTATAATACAACCTCCCACACCGACCAATCCTCACGATTGAGTGTCAAAGAAGACCCTTCTTATGA
CTCAGTCAGAAGAGGAGCTTGGGGCAATAACATGAATTCTGGCCTCAACAAAAGTCCTCCCCTTGGAGGG
GCACAAACGATCAGTAAGAATACAGAGCAACGGCCCCAGCCAGATCCGTATCAGATCCTGGGCCCGACCA
GCAGTCGCCTAGCCAACCCTGGAAGCGGGCAGATCCAGCTGTGGCAATTCCTCCTGGAGCTGCTCTCCGA
CAGCGCCAACGCCAGCTGTATCACCTGGGAGGGGACCAACGGGGAGTTCAAAATGACGGACCCCGATGAG
GTGGCCAGGCGCTGGGGCGAGCGGAAAAGCAAGCCCAACATGAATTACGACAAGCTGAGCCGGGCCCTCC
GTTATTACTATGATAAAAACATTATGACCAAAGTGCACGGCAAAAGATATGCTTACAAATTTGACTTCCA
CGGCATTGCCCAGGCTCTGCAGCCACATCCGACCGAGTCGTCCATGTACAAGTACCCTTCTGACATCTCC
TACATGCCTTCCTACCATGCCCACCAGCAGAAGGTGAACTTTGTCCCTCCCCATCCATCCTCCATGCCT**G**
TCACTTCCTCCAGCTTCTTTGGAGCCGCATCACAATACTGGACCTCCCCCACGGGGGGAATCTACCCCAA
CCCCAACGTCCCCCGCCATCCTAACACCCACGTGCCTTCACACTTAGGCAGCTACTACTAGAAGCTTCTT
CTAGCTGAAGCCCATCCTGCACACTTACTGGATGCTTTGGACTCAACAGGACATATGTGGCCTTGAAGGG
AAGACAAAACTGGATGTTCTTTCTTGTTGGATAGAACCTTTGTATTTGTTCTTTAAAAACATTTTTTTTA
ATGTTGGTAACTTTTGCTTCCTCTACCTGAACAAAGAGATGAATAATTCCATGGGCCAGTATGCCAGTTT
GAATTCTCAGTCTCCTAGCATCTTGTGAGTTGCATATTAAGATTACTGGAATGGTTAAGTCATGGTTCTG
AGAAAGAAGCTGTACGTTTTCTTTATGTTTTTATGACCAAAGCAGTTTCTTGTCAATACACGGGGTTCAG
TATGACACAGAATCATGGACTTAACCCGTCATGTTCTGGTTTGAGATTTAGTGACAAATAGAGGTGGGAA
GCTTATAATCTAATTTTAGGAGGACCAAATTCAGCGGATGGCAACTGGAACATTGATTGTAAGGCCAGTG
AAGTTTTCACCCAACTGGAATTTGATGGAAAGAAGGTTTGTGTGTTTAAGACGCCAAGGGCATTGCAGAA
TCCCTCTCAGTGGACAGTATGCACTCAGCTGACCACTCTCTCTAGAAATAGTCAAGATATGAACTAAGAA
ATTTTAATGCAAATACATACATTCCTGAAAGACGGGGAATTAAATTACTAATTTTTTTTTTTTAAATGAT
GACAGTGGTCCCAGAACTTGGAAAAGTTGTAGGGATTTCTAAACTCAAGCAGATTCGCAAGTGCTGTGCG
CTTGTCAGACCATCAGACCAGGGCCAACCAATCAGAAGGCAACTTACTGTATAAATTATGCAGAGTTATT
TTCCTATATCTCACAGTATTAAAAAATAAATAATTAAAAATTAAGAATAAATAAACGAGTTGACCTCGGT
CACAAAAGCAGTTTTACTATCGAATCAATCGCTGTTATTTTTTTTTAATGTAATTTGTACATCTTTTTTC
AATCTGTACATTTGGGCTGTCTTGTATGTTTTTATGCTCCTTTTTAAAAAGCATAATATGCCTATAGCTG
AAAAGGAAACAGGGCTGTTTAAGTCACTGACTTATGAGAAAGCAAAGCACTGGTACAGTTATTTAACAGG
CATACACAAGCAGGGAAAAGATAATCCATTTAGATCTTTAATGCTTTGGAAATGCGTGTAACAGTACTGC
AATAATCACAGCTCTGGGAAAAACAACGAAACTTTCCCTTGTGGAGAGGAGGGATTTTCCTGCTCTATAT
AAGCAACATATTTTTAGACATTAAAATATATATAATTTTGCAGGTAATTGTTGACTTTTTTAACTATATT
AAGTGTTAAGCTGACAACTGTCAAAGAAGAC**CATGTTGTAAAATAATTTGAC**TAAATAAATGGTTCCTTC
TCTCAAAAAAAAAAAAA

15) Entrez Gene ID 2956 = MSH6, **mutS homolog 6** = BC004246

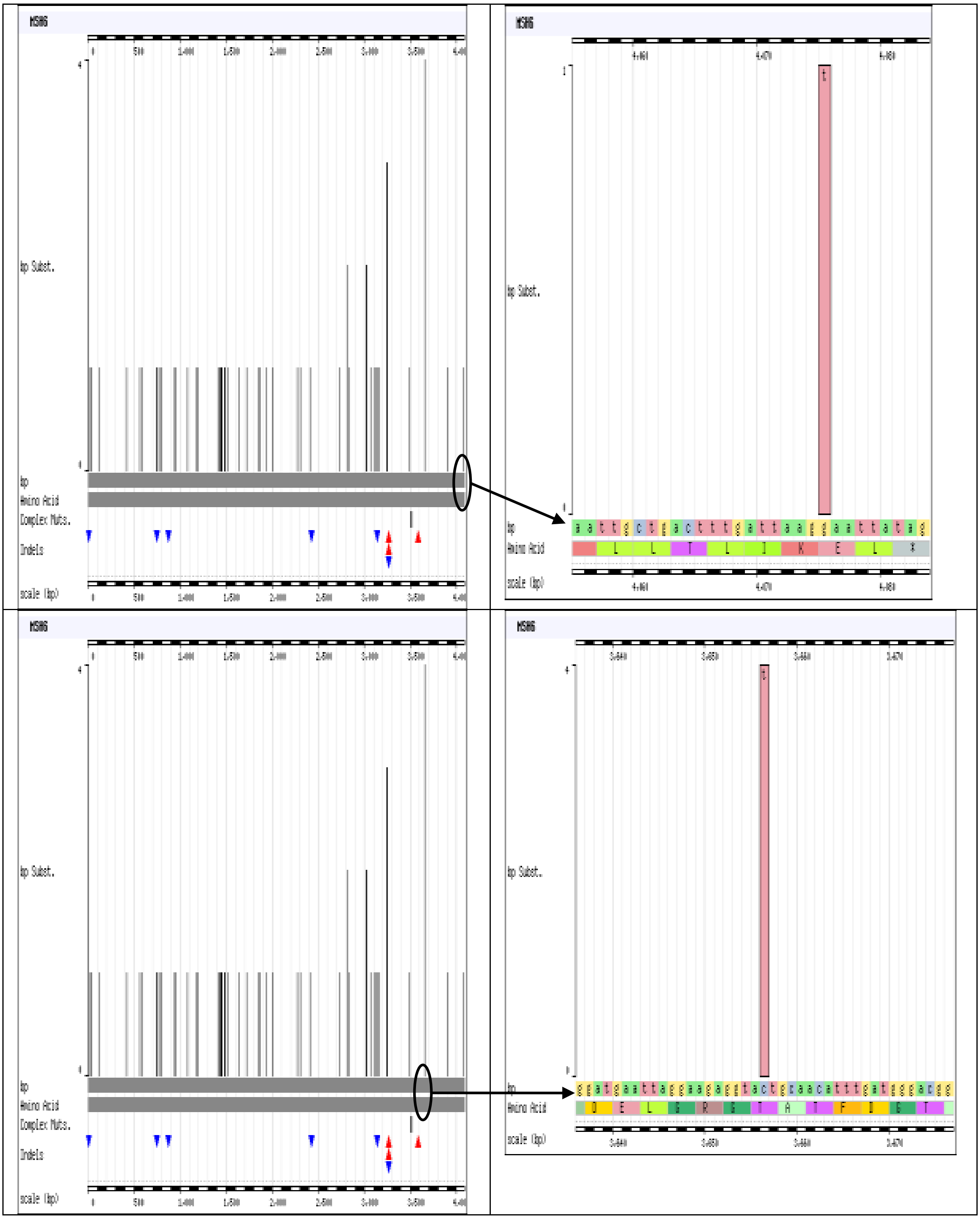
MSH6
bp Subst.
bp
Amino Acid
Complex Muts.
Indels
scale (bp)

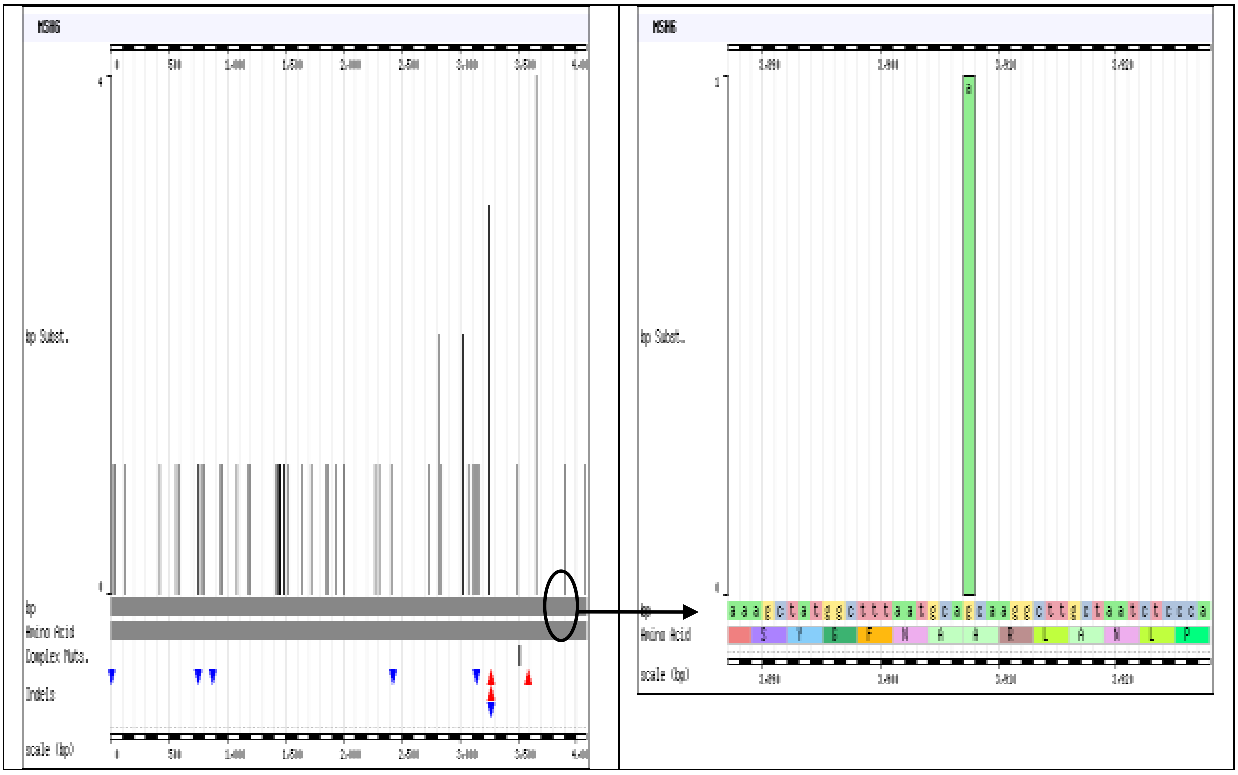

>gi|13279007|gb|BC004246.1| Homo sapiens mutS homolog 6 (E. coli), mRNA (cDNA clone MGC:10498 IMAGE:3629489), complete cds

```
TGCTTTTAGGAGCTCCGTCCGACAGAACGGTTGGGCCTTGCCGGCTGTCGGTATGTCGCGACAGAGCACC
CTGTACAGCTTCTTCCCCAAGTCTCCGGCGCTGAGTGATGCCAACAAGGCCTCGGCCAGGGCCTCACGCG
AAGGCGGCCGTGCCGCCGCTGCCCCCGGGGCCTCTCCTTCCCCAGGCGGGGATGCGGCCTGGAGCGAGGC
TGGGCCTGGGCCCAGGCCCTTGGCGCGATCCGCGTCACCGCCCAAGGCGAAGAACCTCAACGGAGGGCTG
CGGAGATCGGTAGCGCCTGCTGCCCCCACCAGTTGTGACTTCTCACCGGGAGATTTGGTTTGGGCCAAGA
TGGAGGGTTACCCCTGGTGGCCTTGTCTGGTTTACAACCACCCCTTTGATGGAACATTCATCCGCGAGAA
AGGGAAATCAGTCCGTGTTCATGTACAGTTTTTTGATGACAGCCCAACAAGGGGCTGGGTTAGCAAAAGG
CTTTTAAAGCCATATACAGGTTCAAAATCAAAGGAAGCCCAGAAGGGAGGTCATTTTTACAGTGCAAAGC
CTGAAATACTGAGAGCAATGCAACGTGCAGACGAAGCCTTAAATAAAGACAAGATTAAGAGGCTTGAATT
GGCAGTTTGTGATGAGCCCTCAGAGCCAGAAGAGGAAGAAGAGATGGAGGTAGGCACAACTTACGTAACA
GATAAGAGTGAAGAAGATAATGAAATTGAGAGTGAAGAGGAAGTACAGCCTAAGACACAAGGATCTAGGC
GAAGTAGCCGCCAAATAAAAAAACGAAGGGTCATATCAGATTCTGAGAGTGACATTGGTGGCTCTGATGT
GGAATTTAAGCCAGACACTAAGGAGGAAGGAAGCAGTGATGAAATAAGCAGTGGAGTGGGGGATAGTGAG
AGTGAAGGCCTGAACAGCCCTGTCAAAGTTGCTCGAAAGCGGAAGAGAATGGTGACTGGAAATGGCTCTC
TTAAAAGGAAAAGCTCTAGGAAGGAAACGCCCTCAGCCACCAAACAAGCAACTAGCATTTCATCAGAAAC
CAAGAATACTTTGAGAGCTTTCTCTGCCCCTCAAAATTCTGAATCCCAAGCCCACGTTAGTGGAGGTGGT
GATGACAGTAGTCGCCCTACTGTTTGGTATCATGAAACTTTAGAATGGCTTAAGGAGGAAAAGAGAAGAG
ATGAGCACAGGAGGAGGCCTGATCACCCCGATTTTGATGCATCTACACTCTATGTGCCTGAGGATTTCCT
CAATTCTTGTACTCCTGGGATGAGGAAGTGGTGGCAGATTAAGTCTCAGAACTTTGATCTTGTCATCTGT
TACAAGGTGGGGAAATTTTATGAGCTGTACCACATGGATGCTCTTATTGGAGTCAGTGAACTGGGGCTGG
TATTCATGAAAGGCAACTGGGCCCATTCTGGCTTTCCTGAAATTGCATTTGGCCGTTATTCAGATTCCCT
GGTGCAGAAGGGCTATAAAGTAGCACGAGTGGAACAGACTGAGACTCCAGAAATGATGGAGGCACGATGT
AGAAAGATGGCACATATATCCAAGTATGATAGAGTGGTGAGGAGGGAGATCTGTAGGATCATTACCAAGG
GTACACAGACTTACAGTGTGCTGGAAGGTGATCCCTCTGAGAACTACAGTAAGTATCTTCTTAGCCTCAA
AGAAAAAGAGGAAGATTCTTCTGGCCATACTCGTGCATATGGTGTGTGCTTTGTTGATACTTCACTGGGA
AAGTTTTTCATAGGTCAGTTTTCAGATGATCGCCATTGTTCGAGATTTAGGACTCTAGTGGCACACTATC
CCCCAGTACAAGTTTTATTTGAAAAAGGAAATCTCTCAAAGGAAACTAAAACAATTCTAAAGAGTTCATT
GTCCTGTTCTCTTCAGGAAGGTCTGATACCCGGCTCCCAGTTTTGGGATGCATCCAAAACTTTGAGAACT
CTCCTTGAGGAAGAATATTTTAGGGAAAAGCTAAGTGATGGCATTGGGGTGATGTTACCCCAGGTGCTTA
AAGGTATGACTTCAGAGTCTGATTCCATTGGGTTGACACCAGGAGAGAAAAGTGAATTGGCCCTCTCTGC
TCTAGGTGGTTGTGTCTTCTACCTCAAAAAATGCCTTATTGATCAGGAGCTTTTATCAATGGCTAATTTT
GAAGAATATATTCCCTTGGATTCTGACACAGTCAGCACTACAAGATCTGGTGCTATCTTCACCAAAGCCT
ATCAACGAATGGTGCTAGATGCAGTGACATTAAACAACTTGGAGATTTTTCTGAATGGAACAAATGGTTC
```

TACTGAAGGAACCCTACTAGAGAGGGTTGATACTTGCCATACTCCTTTTGGTAAGCGGCTCCTAAAGCAA
TGGCTTTGTGCCCCACTCTGTAACCATTATGCTATTAATGATCGTCTAGATGCCATAGAAGACCTCATGG
TTGTGCCTGACAAAATCTCCGAAGTTGTAGAGCTTCTAAAGAAGCTTCCAGATCTTGAGAGGCTACTCAG
TAAAATTCATAATGTTGGGTCTCCCCTGAAGAGTCAGAACCACCCAGACAGCAGGGCTATAATGTATGAA
GAAACTACATACAGCAAGAAGAAGATTATTGATTTTCTTTCTGCTCTGGAAGGATTCAAAGTAATGTGTA
AAATTATAGGGATCATGGAAGAAGTTGCTGATGGTTTTAAGTCTAAAATCCTTAAGCAGGTCATCTCTCT
GCAGACAAAAAATCCTGAAGGTCGTTTTCCTGATTTGACTGTAGAATTGAACCGATGGGATACAGCCTTT
GACCATGAAAAGGCTCGAAAGACTGGACTTATTACTCCCAAAGCAGGCTTTGACTCTGATTATGACCAAG
CTCTTGCTGACATAAGAGAAAATGAACAGAGCCTCCTGGAATACCTAGAGAAACAGCGCAACAGAATTGG
CTGTAGGACCATAGTCTATTGGGGGATTGGTAGGAACCGTTACCAGCTGGAAATTCCTGAGAATTTCACC
ACTCGCAATTTGCCAGAAGAATACGAGTTGAAATCTACCAAGAAGGGCTGTAAACGATACTGGACCAAAA
CTATTGAAAAGAAGTTGGCTAATCTCATAAATGCTGAAGAACGGAGGGATGTATCATTGAAGGACTGCAT
GCGGCGACTGTTCTATAACTTTGATAAAAATTACAAGGACTGGCAGTCTGCTGTAGAGTGTATCGCAGTG
TTGGATGTTTTACTGTGCCTGGCTAACTATAGTCGAGGGGGTGATGGTCCTATGTGTCGCCCAGTAATTC
TGTTGCCGGAAGATACCCCCCCCTTCTTAGAGCTTAAAGGATCACGCCATCCTTGCATTACGAAGACTTT
TTTTGGAGATGATTTTATTCCTAATGACATTCTAATAGGCTGTGAGGAAGAGGAGCAGGAAAATGGCAAA
GCCTATTGTGTGCTTGTTACTGGACCAAATATGGGGGGCAAGTCTACGCTTATGAGACAGGCTGGCTTAT
TAGCTGTAATGGCCCAGATGGGTTGTTACGTCCCTGCTGAAGTGTGCAGGCTCACACCAATTGATAGAGT
GTTTACTAGACTTGGTGCCTCAGACAGAATAATGTCAGGTGAAAGTACATTTTTTGTTGAATTAAGTGAA
ACTGCCAGCATACTCATGCATGCAACAGCACATTCTCTGGTGCTTGTGGATGAATTAGGAAGAGGTA**C**TG
CAACATTTGATGGGACGGCAATAGCAAATGCAGTTGTTAAAGAACTTGCTGAGACTATAAAATGTCGTAC
ATTATTTTCAACTCACTACCATTCATTAGTAGAAGATTATTCTCAAAATGTTGCTGTGCGCCTAGGACAT
ATGGCATG**CATGGTAGAAAATGAATGTGA**AGACCCCAGCCAGGAGACTATTACGTTCCTCTATAAATTCA
TTAAGGGAGCTTGTCCTAAAAGCTATGGCTTTAATGCA**G**CAAGGCTTGCTAATCTCCCAGAGGAAGTTAT
TCAAAAGGGACATAGAAAAGCAAGAGAATTTGAGAAGATGAATCAGTCACTACGATTATTTCGGGAAGTT
TGCCTGGCTAGTGAAAGGTCAACTGTAGATGCTGAAGCTGTCCATAAATTGCTGACTTTGATTAAG**G**AAT
TATAGACTGACTACATTGGAAGCTTTGAGTTGACTTCTGACAAAGGTGGTAAATTCAGACAACATTATGA
TCTAATAAACTTTATTTTTAAAAAATGAAAAAAAAAAAAAAAAAAAAAA

16) Entrez Gene ID 3181 = HNRNPA2B1 heterogeneous nuclear ribonucleoprotein A2/B1 = BC000506

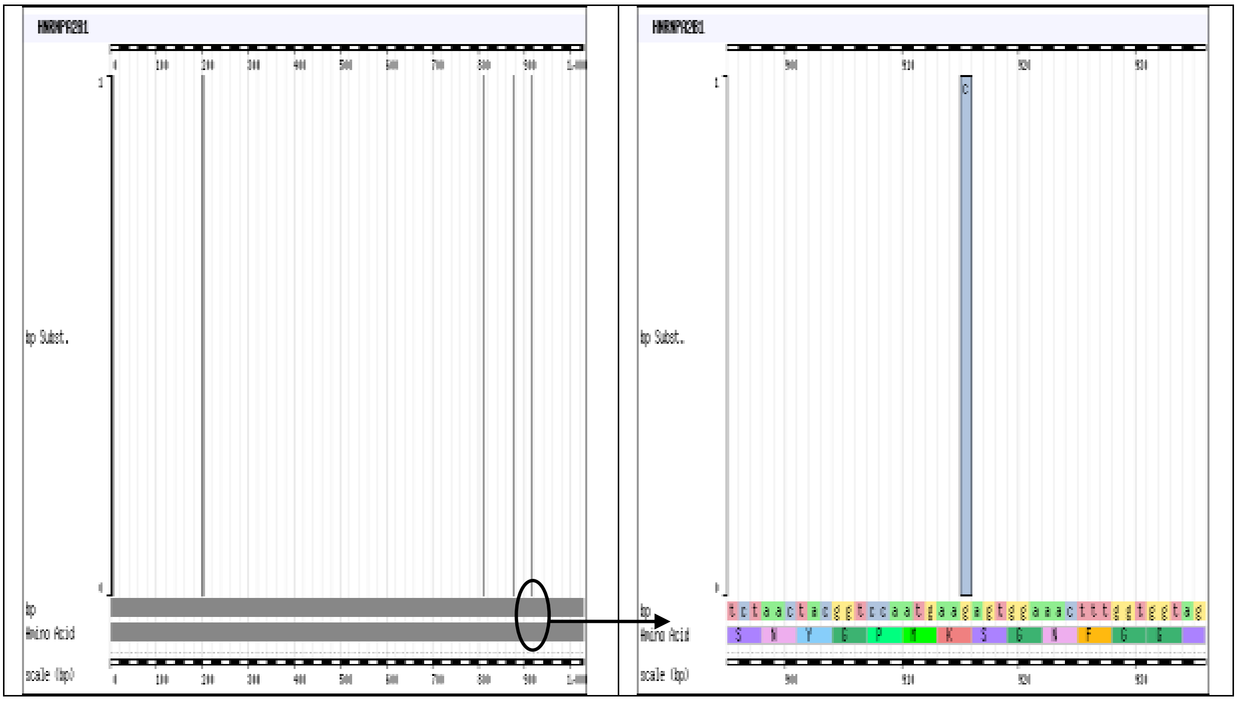

>gi|33875522|gb|BC000506.2| Homo sapiens heterogeneous nuclear ribonucleoprotein A2/B1, mRNA (cDNA clone MGC:8493 IMAGE:2822109), complete cds

GTGCGGAGGTGCTCCTCGCAGAGTTGTTTCTCGAGCAGCGGCAGTTCTCACTACAGCGCCAGGACGAGTC
CGGTTCGTGTTCGTCCGCGGAGATCTCTCTCATCTCGCTCGGCTGCGGGAAATCGGGCTGAAGCGACTGA
GTCCGCGATGGAGAGAGAAAAGGAACAGTTCCGTAAGCTCTTTATTGGTGGCTTAAGCTTTGAAACCACA
GAAGAAAGTTTGAGGAACTACTACGAACAATGGGGAAAGCTTACAGACTGTGTGGTAATGAGGGATCCTG
CAAGCAAAAGATCAAGAGGATTTGGTTTTGTAACTTTTTCATCCATGGCTGAGGTTGATGCTGCCATGGC
TGCAAGACCTCATTCAATTGATGGGAGAGTAGTTGAGCCAAAACGTGCTGTAGCAAGAGAGGAATCTGGA
AAACCAGGGGCTCATGTAACTGTGAAGAAGCTGTTTGTTGGCGGAATTAAAGAAGATACTGAGGAACATC
ACCTTAGAGATTACTTTGAGGAATATGGAAAAATTGATACCATTGAGATAATTACTGATAGGCAGTCTGG
AAAGAAAAGAGGCTTTGGCTTTGTTACTTTTGATGACCATGATCCTGTGGATAAAATCGTATTGCAGAAA
TACCATACCATCAATGGTCATAATGCAGAAGTAAGAAAGGCTTTGTCTAGACAAGAAATGCAGGAGGACC
TGGAGGTGGCAATTTTGGAGGTAGCCCCGGTTATGGAGGAGGAAGAGGAGGATATGGTGGTGGAGGACCT
GGATATGGCAACCAGGGTGGGGGCTACGGAGGTGGTTATGACAACTATGGAGGAGGAAATTATGGAAGTG
GAAATTACAATGATTTTGGAAATTATAACCAGCAACCTTCTAACTACGGTCCAATGAA**G**AGTGGAAACTT
TGGTGGTAGCAGGAACATGGGGGGACCATATGGTGGAGGAAACTATGGTCCAGGAGGCAGTGGAGGAAGT
GGGGGTTATGGTGGGAGGAGCCGATACTGAGCTTCTTCCTATTTGCCATGGGCTTCACTGTATAAATAGG
AGAGGATGAGAGCCCAGAGGTAACAGAACAGCTTCAGGTTATCGAAATAACAATGTTAAGGAAACTCTTA
TCTCAGTCATGCATAAATATGCAGTGATATGGCAGAAGACACCAGAGCAGATGCAGAGAGCCATTTTGTG
AATGGATTGGATTATTTAATAACATTACCTTACTGTGGAGGAAGGATTGTAAAAAAAAATGCCTTTGAGA
CAGTTTCTTAGCTTTTTAATTGTTGTTTCTTTCTAGTGGTCTTTGTAAGAGTGTAGAAGCATTCCTTCTT
TGATAATGTTAAATTTGTAAGTTTCAGGTGACATGTGAAACCTTTTTTAAGATTTTTCTCAAAGTTTTGA
AAAGCTATTAGCCAGGAT**CATGGTGTAATAAGACATAAC**GTTTTTCCTTTAAAAAAATTTAAGTGCGTGT
GTAGAGTTAAGAAGCTGTTGTACATTTATGATTTAATAAAATAATTCTAAAGGAAAAAAAAAAAAAAAAA

17) Entrez Gene ID 3265 = HRAS, v-Ha-ras Harvey rat sarcoma viral oncogene homolog = BC006499

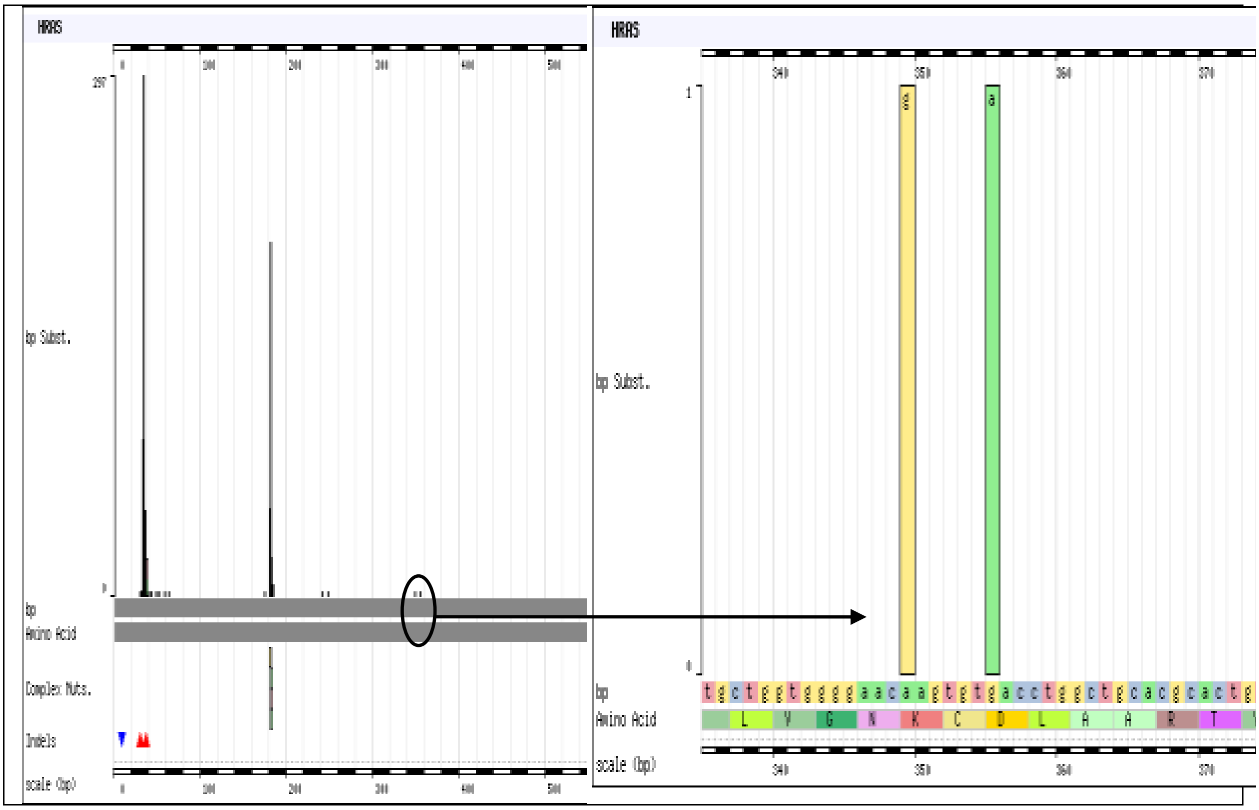

>gi|33873529|gb|BC006499.2| Homo sapiens v-Ha-ras Harvey rat sarcoma viral oncogene homolog, mRNA (cDNA clone MGC:2359 IMAGE:2819996), complete cds
GCAACCCGAGCCGCACCCGCCGCGGACGGAGCCCATGCGCGGGGCGAACCGCGCGCCCCCGCCCCCGCCC
CGCCCCGGCCTCGGCCCCGGCCCTGGCCCCGGGGGCAGTCGCGCCTGTGAACGGTGGGGCAGGAGACCCT
GTAGGAGGACCCCGGGCCGCAGGCCCCTGAGGAGCGATGACGGAATATAAGCTGGTGGTGGTGGGCGCCG
GCGGTGTGGGCAAGAGTGCGCTGACCATCCAGCTGATCCAGAACCATTTTGTGGACGAATACGACCCCAC
TATAGAGGATTCCTACCGGAAGCAGGTGGTCATTGATGGGGAGACGTGCCTGTTGGACATCCTGGATACC
GCCGGCCAGGAGGAGTACAGCGCCATGCGGGACCAGTACATGCGCACCGGGGAGGGCTTCCTGTGTGTGT
TTGCCATCAACAACACCAAGTCTTTTGAGGACATCCACCAGTACAGGGAGCAGATCAAACGGGTGAAGGA
CTCGGATGACGTGCCCATGGTGCTGGTGGGGAAC**A**AGTGT**G**ACCTGGCTGCACGCACTGTGGAATCTCGG
CAGGCTCAGGACCTCGCCCGAAGCTACGGCATCCCCTACATCGAGACCTCGGCCAAGACCCGGCAGGGCA
GCCGCTCTGGCTCTAGCTCCAGCTCCGGGACCCTCTGGGACCCCCCGGGACCCATGTGACCCAGCGGCCC
CTCGCGCTGGAGTGGAGGATGCCTTCTACACGTTGGTGCGTGAGATCCGGCAGCACAAGCTGCGGAAGCT
GAACCCTCCTGATGAGAGTGGCCCCGGCTGCATGAGCTGCAAGTGTGTGCTCTCCTGACGCAGCACAAGC
TCAGGA**CATGGAGGTGCCGGATGCAGG**AAGGAGGTGCAGACGGAAGGAGGAGGAAGGAAGGACGGAAGCA
AGGAAGGAAGGAAGGGCTGCTGGAGCCCAGTCACCCCGGGACCGTGGGCCGAGGTGACTGCAGACCCTCC
CAGGGAGGCTGTGCACAGACTGTCTTGAACATCCCAAATGCCACCGGAACCCCAGCCCTTAGCTCCCCTC
CCAGGCCTCTGTGGGCCCTTGTCGGGCACAGATGGGATCACAGTAAATTATTGGATGGTCTTGAAAAAAA
AAAAAAAAAAAAAAAAAAAAAAAAAAA

18) Entrez Gene ID 3718 = JAK3, Janus kinase 3 = BC028068

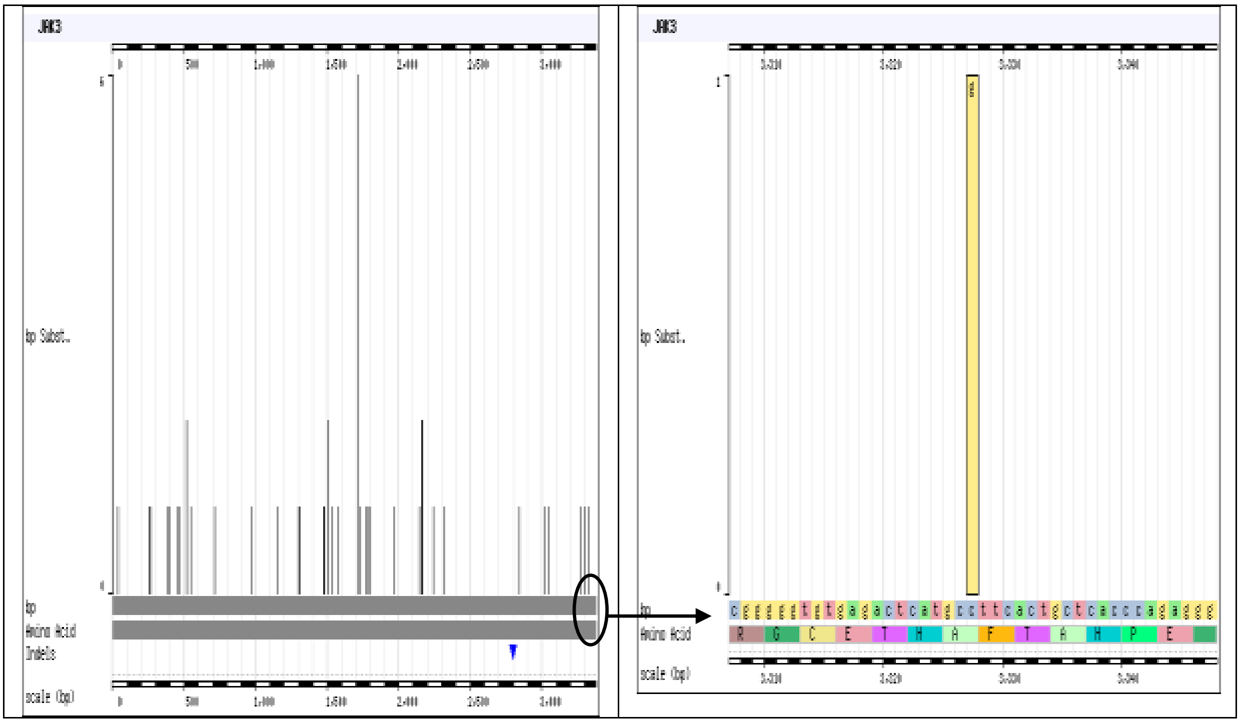

>gi|20380124|gb|BC028068.1| Homo sapiens Janus kinase 3 (a protein tyrosine kinase, leukocyte), mRNA (cDNA clone MGC:39993 IMAGE:5212575), complete cds

CCCGGCTCTGCCCGCCCTTCGAAAGTCCAGGGTCCCTGCCCGCTAGGACTGAGGGGCTTTTTCTCTCTGT
GCCCCAGGCAAGTTGCACTCATGGCACCTCCAAGTGAAGAGACGCCCCTGATCCCTCAGCGTTCATGCAG
CCTCTTGTCCACGGAGGCTGGTGCCCTGCATGTGCTGCTGCCCGCTCGGGGCCCCGGGCCCCCCCAGCGC
CTATCTTTCTCCTTTGGGGACCACTTGGCTGAGGACCTGTGCGTGCAGGCTGCCAAGGCCAGCGGCATCC
TGCCTGTGTACCACTCCCTCTTTGCTCTGGCCACGGAGGACCTGTCCTGCTGGTTCCCCCCGAGCCACAT
CTTCTCCGTGGAGGATGCCAGCACCCAAGTCCTGCTGTACAGGATTCGCTTTTACTTCCCCAATTGGTTT
GGGCTGGAGAAGTGCCACCGCTTCGGGCTACGCAAGGATTTGGCCAGTGCTATCCTTGACCTGCCAGTCC
TGGAGCACCTCTTTGCCCAGCACCGCAGTGACCTGGTGAGTGGGCGCCTCCCCGTGGGCCTCAGTCTCAA
GGAGCAGGGTGAGTGTCTCAGCCTGGCCGTGTTGGACCTGGCCCGGATGGCGCGAGAGCAGGCCCAGCGG
CCGGGAGAGCTGCTGAAGACTGTCAGCTACAAGGCCTGCCTACCCCCAAGCCTGCGCGACCTGATCCAGG
GCCTGAGCTTCGTGACGCGGAGGCGTATTCGGAGGACGGTGCGCAGAGCCCTGCGCCGCGTGGCCGCCTG
CCAGGCAGACCGGCACTCGCTCATGGCCAAGTACATCATGGACCTGGAGCGGCTGGATCCAGCCGGGGCC
GCCGAGACCTTCCACGTGGGCCTCCCTGGGGCCCTTGGTGGCCACGACGGGCTGGGGCTGCTCCGCGTGG
CTGGTGACGGCGGCATCGCCTGGACCCAGGGAGAACAGGAGGTCCTCCAGCCCTTCTGCGACTTTCCAGA
AATCGTAGACATTAGCATCAAGCAGGCCCCACGCGTTGGCCCGGCCGGAGAGCACCGCCTGGTCACTGTT
ACCAGGACAGACAACCAGATTTTAGAGGCCGAGTTCCCAGGGCTGCCCGAGGCTCTGTCGTTCGTGGCGC
TCGTGGACGGCTACTTCCGGCTGACCACGGACTCCCAGCACTTCTTCTGCAAGGAGGTGGCACCGCCGAG
GCTGCTGGAGGAAGTGGCCGAGCAGTGCCACGGCCCCATCACTCTGGACTTTGCCATCAACAAGCTCAAG
ACTGGGGGCTCACGTCCTGGCTCCTATGTTCTCCGCCGCAGCCCCCAGGACTTTGACAGCTTCCTCCTCA
CTGTCTGTGTCCAGAACCCCCTTGGTCCTGATTATAAGGGCTGCCTCATCCGGCGCAGCCCCACAGGAAC
CTTCCTTCTGGTTGGCCTCAGCCGACCCCACAGCAGTCTTCGAGAGCTCCTGGCAACCTGCTGGGATGGG
GGGCTGCACGTAGATGGGGTGGCAGTGACCCTCACTTCCTGCTGTATCCCCAGACCCAAAGAAAAGTCCA
ACCTGATCGTGGTCCAGAGAGGTCACAGCCCACCCACATCATCCTTGGTTCAGCCCCAATCCCAATACCA
GCTGAGTCAGATGACATTTCACAAGATCCCTGCTGACAGCCTGGAGTGGCATGAGAACCTGGGCCATGGG
TCCTTCACCAAGATTTACCGGGGCTGTCGCCATGAGGTGGTGGATGGGGAGGCCCGAAAGACAGAGGTGC
TGCTGAAGGTCATGGATGCCAAGCACAAGAACTGCATGGAGTCATTCCTGGAAGCAGCGAGCTTGATGAG
CCAAGTGTCGTACCGGCATCTCGTGCTGCTCCACGGCGTGTGCATGGCTGGAGACAGTGAGAGCCCCCCA
CCCACCCACCCCACCCCTGCCTCACCCAAGTCTAGGCTGTTCTTCCCACCTCTGTTCTGAGCCGCTATAT
GACAGCCCCAGCAACACACTGGGCCACCCTGGATGGGAGCCGTGTTCATTACCCTTTATTTATGTCTCTC
CATCATCACTCCTTGGAAAGCGGCTCCAGGTTCTCACCCATATCCAGCCCCAGAATGACCTGAAGTCAGA
CAAACCTGGCTTTCTAATCTCTGCAGCTTTGTACAGGTCACGTAACCTTTCTGAGCCTTGGTTTCATTGG
TTGGGAGTCTAGGATGGGCCAGGAGCTGGGACAGAGCCTAGAATGTGACAGGCAGGGTGTGATGAGGTGT
GAGGAGGGCAGCACGGAGCACTGTGGATGGTCAGAGAGGCATTCCTGGCAGAGGGAACAGCATGCCAAGC

GTGAGAAGGCTCAGAGTAAGGAGGTTAAGAGCCCAAGTATTGGAGCCTACAGTTTTGCCCCTTCCATGCA
GTGTGACAGTGGGCAAGTTCCTTTCCCTCTCTGGGTCTCAGTTCTGTCCCCTGCAAAATGGTCAGAGCTT
ACCCCTTGGCTGTGCAGGGTCAACTTTCTGACTGGTGAGAGGGATTCT**CATGCAGGTTAAGCTTCTGCT**G
CTCCTCCTCACCTGCAAAGCTTTTCTGCCACTTTTGCCTCCTTGGAAAACTCTTATCCATCTCTCAAAAC
TCCAGCTACCACATCCTTGCAGCCTTCCCTCATATACCCCCACTACTACTGTAGCCCTGTCCTTCCCTCC
AGCCCCACTCTGGCCCTGGGGCTGGGGAAGTGTCTGTGTCCAGCTGTCTCCCCTGACCTCAGGGTTCCTT
GGGGGCTGGGCTGAGGCCTCAGTACAGAGGGGGCTCTGGAAATGTTTGTTGACTGAATAAAGGAATTCAG
TGGAAAAAAAAAAAAAAAAAAAAAAAAAAAAAAAAAAAAAAAAAAAAAAAAAAAAAAAAAAAAAAAAAAA
AAAAAAAAAAA

19) Entrez Gene ID 4089 = SMAD4, SMAD family member 4 = BC002379

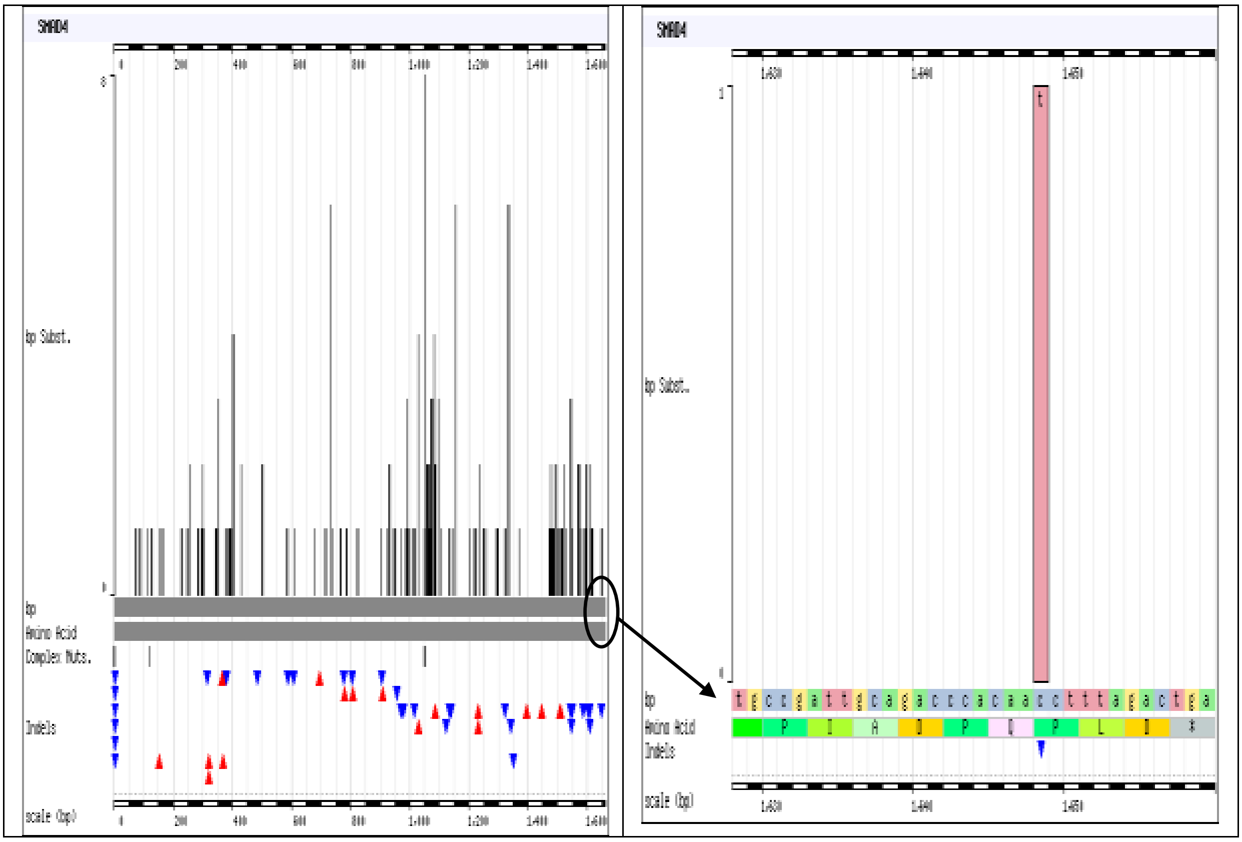

>gi|33876626|gb|BC002379.2| Homo sapiens SMAD family member 4, mRNA (cDNA clone MGC:8602 IMAGE:2961238), complete cds
CAACAACACGGCCCTGGTCGTCGTCGCCGCTGCGGTAACGGAGCGGTTTGGGTGGCGGAGCCTGCGTTCG
CGCCTTCCCACTCCCCTCGCCACCGCCCGAGCCCAGGTTATCCTGAATACATGTCTAACAATTTTCCTTG
CAACGTTAGCTGTTGTTTTTCACTGTTTCCAAAGGATCAAAATTGCTTCAGAAATTGGAGACATATTTGA
TTTAAAAGGAAAAACTTGAACAAATGGACAATATGTCTATTACGAATACACCAACAAGTAATGATGCCTG
TCTGAGCATTGTGCATAGTTTGATGTGCCATAGACAAGGTGGAGAGAGTGAAACATTTGCAAAAAGAGCA
ATTGAAAGTTTGGTAAAGAAGCTGAAGGAGAAAAAAGATGAATTGGATTCTTTAATAACAGCTATAACTA
CAAATGGAGCTCATCCTAGTAAATGTGTTACCATACAGAGAACATTGGATGGGAGGCTTCAGGTGGCTGG
TCGGAAAGGATTTCCTCATGTGATCTATGCCCGTCTCTGGAGGTGGCCTGATCTTCACAAAAATGAACTA
AAACATGTTAAATATTGTCAGTATGCGTTTGACTTAAAATGTGATAGTGTCTGTGTGAATCCATATCACT
ACGAACGAGTTGTATCACCTGGAATTGATCTCTCAGGATTAACACTGCAGAGTAATGCTCCATCAAGTAT
GATGGTGAAGGATGAATATGTGCATGACTTTGAGGGACAGCCATCGTTGTCCACTGAAGGACATTCAATT
CAAACCATCCAGCATCCACCAAGTAATCGTGCATCGACAGAGACATACAGCACCCCAGCTCTGTTAGCCC
CATCTGAGTCTAATGCTACCAGCACTGCCAACTTTCCCAACATTCCTGTGGCTTCCACAAGTCAGCCTGC
CAGTATACTGGGGGGCAGCCATAGTGAAGGACTGTTGCAGATAGCATCAGGGCCTCAGCCAGGACAGCAG
CAGAATGGATTTACTGGTCAGCCAGCTACTTACCATCATAACAGCACTACCACCTGGACTGGAAGTAGGA
CTGCACCATACACACCTAATTTGCCTCACCACCAAAACGGCCATCTTCAGCACCACCCGCCTATGCCGCC

CCATCCCGGACATTACTGGCCTGTTCACAATGAGCTTGCATTCCAGCCTCCCATTTCCAATCATCCTGCT
CCTGAGTATTGGTGTTCCATTGCTTACTTTGAAATGGATGTTCAGGTAGGAGAGACATTTAAGGTTCCTT
CAAGCTGCCCTATTGTTACTGTTGATGGATACGTGGACCCTTCTGGAGGAGATCGCTTTTGTTTGGGTCA
ACTCTCCAATGTCCACAGGACAGAAGCCATTGAGAGAGCAAGGTTGCACATAGGCAAAGGTGTGCAGTTG
GAATGTAAAGGTGAAGGTGATGTTTGGGTCAGGTGCCTTAGTGACCACGCGGTCTTTGTACAGAGTTACT
ACTTAGACAGAGAAGCTGGGCGTGCACCTGGAGATGCTGTTCATAAGATCTACCCAAGTGCATATATAAA
GGTCTTTGATTTGCGTCAGTGTCATCGACAGATGCAGCAGCAGGCGGCTACTGCACAAGCTGCAGCAGCT
GCCCAGGCAGCAGCCGTGGCAGGAAACATCCCTGGCCCAGGATCAGTAGGTGGAATAGCTCCAGCTATCA
GTCTGTCAGCTGCTGCTGGAATTGGTGTTGATGACCTTCGTCGCTTATGCATACTCAGGATGAGTTTTGT
GAAAGGCTGGGGACCGGATTACCCAAGACAGAGCATCAAAGAAACACCTTGCTGGATTGAAATTCACTTA
CACCGGGCCCTCCAGCTCCTAGACGAAGTACTTCATACCATGCCGATTGCAGACCCACAA**C**CTTTAGACT
GAGGTCTTTTACCGTTGGGGCCCTTAACCTTATCAGGATGGTGGACTACAAAATACAATCCTGTTTATAA
TCTGAAGATATATTTCACTTTTGTTCTGCTTTATCTTTTCATAAAGGGTTGAAAATGTGTTTGCTGCCTT
GCTCCTAGCAGACAGAAACTGGATTAAAACAATTTTTTTTTTCCTCTTCAGAACTTGTCAGGCATGGCTC
AGAGCTTGAAGATTAGGAGAAACACATTCTTATTAATTCTTCACCTGTTATGTATGAAGGAATCATTCCA
GTGCTAGAAAATTTAGCCCTTTAAAACGTCTTAGAGCCTTTTATCTGCAGAACATCGATATGTATATCAT
TCTACAGAATAATCCAGTATTGCTGATTTTAAAGGCAGAGAAGTTCTCAAAGTTAATTCACCTATGTTAT
TTTGTGTACAAGTTGTTATTGTTGAACATACTTCAAAAATAATGTGCCATGTGGGTGAGTTAATTTTACC
AAGAGTAACTTTACTCTGTGTTTAAAAAGTAAGTTAATAATGTATTGTAATCTTTCATCCAAAATATTTT
TTGCAAGTTATATTAGTGAAGATGGTTTCAATTCAGATTGTCTTGCAACTTCAGTTTTATTTTTGCCAAG
GCAAAAAACTCTTAATCTGTGTGTATATTGAGAATCCCTTAAAATTACCAGACAAAAAAATTTAAAATTA
CGTTTGTTATTCCTAGTGGATGACTGTTGATGAAGTATACTTTTCCCCTGTTAAACAGTAGTTGTATTCT
TCTGTATTTCTAGGCACAAGGTTGGTTGCTAAGAAGCCTATAAGAGGAATTTCTTTTCCTTCATTCATAG
GGAAAGGTTTTGTATTTTTTAAAACACTAAAAGCAGCGTCACTCTACCTAATGTCTCACTGTTCTGCAAA
GGTGGCAATGCTTAAACTAAATAATGAATAAACTGAATATTTTGGAAACTGCTAAATTCTATGTTAAATA
CTGTGCAGAATAATGGAAACATTACAGTTCATAATAGGTAGTTTGGATATTTTTGTACTTGATTTGATGT
GACTTTTTTTGGTATAATGTTTAAAT**CATGTATGTTATGATATTGTT**TAAAATTCAGTTTTTGTATCTTG
GGGCAAGACTGCAAACTTTTTTATATCTTTTGGTTATTCTAAGCCCTTTGCCATCAATGATCATATCAAT
TGGCAGTGACTTTGTATAGAGAATTTAAGTAGAAAAGTTGCAGATGTATTGACTGTACCACAGACACAAT
ATGTATGCTTTTTACCTAGCTGGTAGCATAAATAAAACTGAATCTCAACATAAAAAAAAAAAAAAAAAAA

20) Entrez Gene ID 4299 = AFF1, AF4/FMR2 family, member 1 = L13773

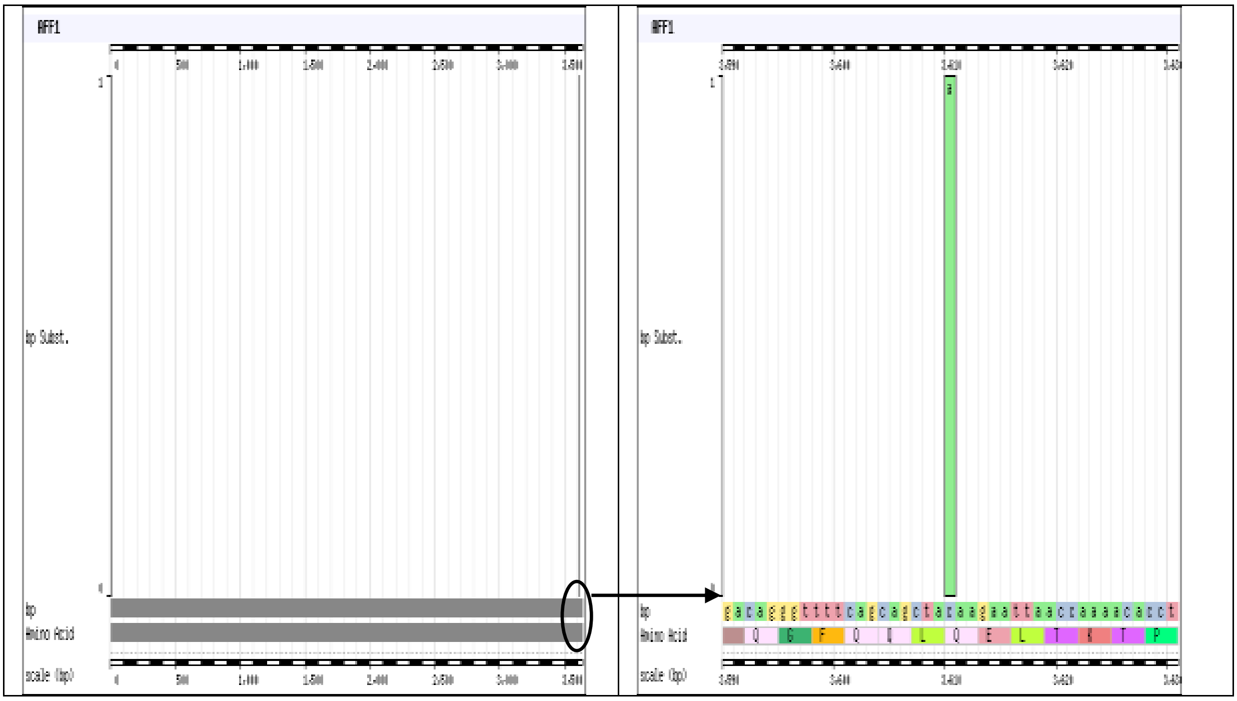

>gi|306446|gb|L13773.1|HUMAF4Y Human AF-4 mRNA, complete cds
GGCAATTTCTTTTCCTTTCTAACTGTGGCCCGCGTTGTGCTGTTGCTGGGCAGGCGTTGGGCGCCGGCGG
TCTTCGAGCGTGGGGGCCCGCTGGCTTTCCCTTCTCAGAAACTGCGCCGGGGGCGCTCGCTTGCCCCGGA
TTCGGACGCGGCGCTCCCCGGGCTCGTCTGAAGTGCAGATCGCCGCAGAGGCCCCAGTGCCCGGATGTCC
ATCAGGATTAGCGCGAGCCAATACGGGCCGAGCCCGGGGCTGCGCCGAGGACGCCCGGGGCTCGAGAGCA
GGTAGTCCCGTAACATCGGGGCGCCGCGCCGGGACGCGTCCCCGCCCGGCTCCGCCAAATGGTGAGCGCG
GCGCTGGCAGCAGGGCCCGCGGGGTGAAGGCGCTCATGGACGGAAGACCCCTGGCTCTATAAGCTGAATT
ATGGCAGCCCAGTCAAGTTTGTACAATGACGACAGAAACCTGCTTCGAATTAGAGAGAAGGAAAGACGCA
ACCAGGAAGCCCACCAAGAGAAAGAGGCATTTCCTGAAAAGATTCCCCTTTTTGGAGAGCCCTACAAGAC
AGCAAAAGGTGATGAGCTGTCTAGTCGAATACAGAACATGTTGGGAAACTACGAAGAAGTGAAGGAGTTC
CTTAGTACTAAGTCTCACACTCATCGCCTGGATGCTTCTGAAAATAGGTTGGGAAAGCCGAAATATCCTT
TAATTCCTGACAAAGGGAGCAGCATTCCATCCAGCTCCTTCCACACTAGTGTCCACCACCAGTCCATTCA
CACTCCTGCGTCTGGACCACTTTCTGTTGGCAACATTAGCCACAATCCAAAGATGGCGCAGCCAAGAACT
GAACCAATGCCAAGTCTCCATGCCAAAAGCTGCGGCCCACCGGACAGCCAGCACCTGACCCAGGATCGCC
TTGGTCAGGAGGGGTTCGGCTCTAGTCATCACAAGAAAGGTGACCGAAGAGCTGACGGAGACCACTGTGC
TTCGGTGACAGATTCGGCTCCAGAGAGGGAGCTTTCTCCCTTAATCTCTTTGCCTTCCCCAGTTCCCCCT
TTGTCACCTATACATTCCAACCAGCAAACTCTTCCCCGGACGCAAGGAAGCAGCAAGGTTCATGGCAGCA
GCAATAACAGTAAAGGCTATTGCCCAGCCAAATCTCCCAAGGACCTAGCAGTGAAAGTCCATGATAAAGA
GACCCCTCAAGACAGTTTGGTGGCCCCTGCCCAGCCGCCTTCTCAGACATTTCCACCTCCCTCCCTCCCC
TCAAAAAGTGTTGCAATGCAGCAGAAGCCCACGGCTTATGTCCGGCCCATGGATGGTCAAGATCAGGCCC
CTAGTGAATCCCCTGAACTGAAACCACTGCCGGAGGACTATCGACAGCAGACCTTTGAAAAAACAGACTT
GAAAGTGCCTGCCAAAGCCAAGCTCACCAAACTGAAGATGCCTTCTCAGTCAGTTGAGCAGACCTACTCC
AATGAAGTCCATTGTGTTGAAGAGATTCTGAAGGAAATGACCCATTCATGGCCGCCTCCTTTGACAGCAA
TACATACGCCTAGTACAGCTGAGCCATCCAAGTTTCCTTTCCCTACAAAGGACTCTCAGCATGTCAGTTC
TGTAACCCAAAACCAAAAACAATATGATACATCTTCAAAAACTCACTCAAATTCTCAGCAAGGAACGTCA
TCCATGCTCGAAGACGACCTTCAGCTCAGTGACAGTGAGGACAGTGACAGTGAACAAACCCCAGAGAAGC
CTCCCTCCTCATCTGCACCTCCAAGTGCTCCACAGTCCCTTCCAGAACCAGTGGCATCAGCACATTCCAG
CAGTGCAGAGTCAGAAAGCACCAGTGACTCAGACAGTTCCTCAGACTCAGAGAGCGAGAGCAGTTCAAGT
GACAGCGAAGAAAATGAGCCCCTAGAAACCCCAGCTCCGGAGCCTGAGCCTCCAACAACAAACAAATGGC
AGCTGGACAACTGGCTGACCAAAGTCAGCCAGCCAGCTGCGCCACCAGAGGGCCCCAGGAGCACAGAGCC
CCCACGGCGGCACCCAGAGAGTAAGGGCAGCAGCGACAGTGCCACGAGTCAGGAGCATTCTGAATCCAAA
GATCCTCCCCCCTAAAAGCTCCAGCAAAGCCCCCCGGGCCCCACCCGAAGCCCCCCACCCCGGAAAGAGGA
GCTGTCAGAAGTCTCCGGCACAGCAGGAGCCCCCACAAAGGCAAACCGTTGGAACCAAACAACCCAAAAA
ACCTGTCAAGGCCTCTGCCCGGGCAGGTTCACGGACCAGCCTGCAGGGGGAAAGGGAGCCAGGGCTTCTT
CCCTATGGCTCCCGAGACCAGACTTCCAAAGACAAGCCCAAGGTGAAGACGAAAGGACGGCCCCGGGCCG
CAGCAAGCAACGAACCCAAGCCAGCAGTGCCCCCCTCCAGTGAGAAGAAGAAGCACAAGAGCTCCCTCCC
TGCCCCCTCTAAGGCTCTCTCAGGCCCAGAACCCGCGAAGGACAATGTGGAGGACAGGACCCCTGAGCAC

TTTGCTCTTGTTCCCCTGACTGAGAGCCAGGGCCCACCCCACAGTGGCAGCGGCAGCAGGACTAGTGGCT
GCCGCCAAGCCGTGGTGGTCCAGGAGGACAGCCGCAAAGACAGACTCCCATTGCCTTTGAGAGACACCAA
GCTGCTCTCACCGCTCAGGGACACTCCTCCCCCACAAAGCTTGATGGTGAAGATCACCCTAGACCTGCTC
TCTCGGATACCCCAGCCTCCCGGGAAGGGGAGCCGCCAGAGGAAAGCAGAAGATAAACAGCCGCCCGCAG
GGAAGAAGCACAGCTCTGAGAAGAGGAGCTCAGACAGCTCAAGCAAGTTGGCCAAAAAGAGAAAGGGTGA
AGCAGAAAGAGACTGTGATAACAAGAAAATCAGACTGGAGAAGGAAATCAAATCACAGTCATCTTCATCT
TCATCCTCCCACAAAGAATCTTCTAAAACAAAGCCCTCCAGGCCCTCCTCACAGTCCTCAAAGAAGGAAA
TGCTCCCCCCGCCACCCGTGTCCTCGTCCTCCCAGAAGCCAGCCAAGCCTGCACTTAAGAGGTCAAGGCG
GGAAGCAGACACCTGTGGCCAGGACCCTCCCAAAAGTGCCAGCAGTACCAAGAGCAACCACAAAGACTCT
TCCATTCCCAAGCAGAGAAGAGTAGAGGGGAAGGGCTCCAGAAGCTCCTCGGAGCACAAGGGTTCTTCCG
GAGATACTGCAAATCCTTTTCCAGTGCCTTCTTTGCCAAATGGTAACTCTAAACCAGGGAAGCCTCAAGT
GAAGTTTGACAAACAACAAGCAGACCTTCACATGAGGGAGGCAAAAAAGATGAAGCAGAAAGCAGAGTTA
ATGACGGACAGGGTTGGAAAGGCTTTTAAGTACCTGGAAGCCGTCTTGTCCTTCATTGAGTGCGGAATTG
CCACAGAGTCTGAAAGCCAGTCATCCAAGTCAGCTTACTCTGTCTACTCAGAAACTGTAGATCTCATTAA
ATTCATAATGTCATTAAAATCCTTCTCAGATGCCACAGCGCCAACACAAGAGAAAATATTTGCTGTTTTA
TGCATGCGTTGCCAGTCCATTTTGAACATGGCGATGTTTCGTTGTAAAAAAGACATAGCAATAAAGTATT
CTCGTACTCTTAATAAACACTTCGAGAGTTCTTCCAAAGTCGCCCAGGCACCTTCTCCATGCATTGCAAG
CACAGGCACACCATCCCCTCTTTCCCCAATGCCTTCTCCTGCCAGCTCCGTAGGGTCCCAGTCAAGTGCT
GGCAGTGTGGGGAGCAGTGGGGTGGCTGCCACTATCAGCACCCCAGTCACCATCCAGAATATGACATCTT
CCTATGTCACCATCACATCCCATGTTCTTACCGCCTTTGACCTTTGGGAACAGGCCGAGGCCCTCACGAG
GAAGAATAAAGAATTCTTTGCTCGGCTCAGCACAAATGTGTGCACCTTGGCCCTCAACAGCAGTTTGGTG
GACCTGGTGCACTATACACGACAGGGTTTTCAGCAGCTA**C**AAGAATTAACCAAAACACCTTAATGGAGCC
CCAGGTTGATTCAATGCCTTGGGAACTATTTTTGCACATTGGAAGCCTCAAAAACAGTCCAGACGTTTGT
TTCATCAGGACACCAAACTCTAAAAAAGAAGCACCACGAGATGGCCAGGACATTTGTCCACTTAAACTCT
CAACAACAGTGTGATCATTGGTTGGACACTGTGGTTATGCAGAAGCAGAGATGAGGAGGCTGGCCCCAGA
GATGATCTTGCCCTTCCTAACTAAAGGACAGAAGTGCAATTTAGCTTAAATGGGTGTATGAATGGTCTAG
AAACATTTCTATTTTTTTTTTAAACCAGCAGGATACAAGTTGCAAATGAAATGAGGAGAAACAGTTTCAA
CTCTGAAAGTGAATTTCACGTCATCTCAGTAGCCACGCTAGTCCATTCCCAGAAGGAAATTTTTTTTTTT
AACAATGACTTTTGGTAAAGGGTTTTGTGGATGATTTTTTTTCTTTTGAGTTTTGGGAGAAATATTTGTT
TAATAACTTCTAATGGCCATCTGTAAACCATAAGTAATGAAGGACTCCACTGTGCCCCACTTTCTGCCAA
TGAACAGTGGCTTGATAATACCAAGTATTGTTGTAATTTATAAAATTGAAGGCAACCCCCGCTCCTGCCG
CCCCCAATCTCCCCATTGCCTAGAGCGCTGCACATTGACCCCAGCTCTGACTTCTCATTACTGTGCTGAA
AGTCAGCCCACGTCGGAGCGGTGAGGAGGAGCCACAGCACATGGGGTGCCACCTCGAGGTCTGCACAGGA
GGACTTGGCGCTGCCATTTCCTACCCCTGCCATTTCCCACCCCTGCTTCAGCGAAAGGGACTCTCTAACA
GGGCAGTCACTGTTGACTCTATTCTGAATTTCCTCCCTTGGGGAAGAAGGGAACCAACATTTATACCTGA
CCAGATGGCTAAAGTGCTTTTAAAGTTTTGTTTAAGTAGAGCTGGAATTTGAGGTGCTGATCTGTGGTCT
ACAGTTATGTGGTAACTCATGTTGTCCAGCCAACTCAGAGTTTCGTCAGTGAACAAGAAACATGAAATCT
GCTTCTTAGAGAGGCTATATTTTTCTGCTACAAATATTTTATATTTATAGCAAAACTAGACTTTCAGAGT
CCTTGATTGTCTAGGGGAAGTTAACTCCCTGAGAGGATGTAGAGATTTGGGGTGGTTGATTAGACTTTTG
AAAAACTCATCACCACATGCCTTCACTCCAGAGTGTTCTCAGCTAGATTTGATTTGGTTGAGGAGGAACT
GTGGCCCTCCGTAAGTTATTGCCATAGTGTATGCATTAAACCAAGTCCATTTTGAATGACCTAAAATGAA
GTAACACAATCAGAAATCCCATGTGCCCATAAGCACAGATTTTTCTTTTTCATTGAAACTTTAAAGGTTA
TTATTGGAAACATTACTTTGAGTGCAGTGTTTTTAAAAGCCAATTCTTTTTTATCCCTTTTAGAAGTAGA
ATTTGCACACTTACTACAATTGAGGAGTGTCATCTCTATAACTTTTTCTCCGCCTTTGTCCCATTCTGCC
CCTGGACATGTTTCCTACCAAGCATGTTTCACATTTTCCTATTAGTGGAGGAGGGAGAACCATATTTATT
TATAATGAAGACATCTAAGATCCCTATGATGAATGCAGGAACTCTCTTGGTAGTTTGTAAATACACAAAG
GGATGTGTCGAGGGATGGGAGCGATGCTTATCTCTCACAGTGTGAGTGGTCTGTGTGAGGCTGTTCCTTC
AGTTCTTCTCCAGACTGTTCTTTGGTTGTCACTTAAGTCAGAGGTCTGGTCCCTCATGTTTAGGTGAAAG
CCAGAGAATGACAGCTGTAGTCATATCTGAGCATAAGACCTTGATGTGTGATTCCTGATGACCGGTTTCA
TTTATTCATGTAATAAAGCAAAGGCCCTGGTCCTTTTTAAACTACTAGTTTTAAAAACCTGTGTTAAATG
AACAGTAATTGCCTGGTAGGTTTGGTGTGTGTGTAGCATTGTGTGTCCATCTGTTATATGTAAAGGACAA
GGCACCAGAATCAGGCTTTATTTCGATATTGAAGATGTTATTTAACATCTTTCTTTTTTCCTTACTCCCT
TAGCCATCCCCTCCCCTTTTGTCCTATCATTCCCTAGAACAAGCCACCTGTCAATTGTGAAGGGTTGTGT
TCTTTATGGCAGGTTCTATGCAGATTGTGCCAGAGCATGTGCGTGTTCTGTTGGCAAGCCACAGTGCTCC
CTTGACTGAAGACATTTCCAGGTAGATTTCTCAGCCAGCTCTAAAACAGATTGCTTTTTCAGTGGCCTTA
CTCTTTGTGGGTTTTTTTTTTTCTCTGAACTTGATATAAAGATTTTATTTGTCCCTTGAAAAAGTAACAA
ATGTGCATAGATCAATTTGTACTACTTTGGTCATTGGATATTTCTGATCCTTATTGCATTGTACCTAAAG
GAGAGTAACTAATGGTAACCTTTTTAATAGAGTATGTGAAAGGTAGTGGCTGATGAATCCTTAACGTTCA
TAGGGTCTTTTTGCTGTTACGGTTGTATATAGAGGTCTGAAGGATTTTTAAAATGATTTGCACTTTTTCA
CTGCATGCTTACAATTCCCAAAGGCAAAATCTGTACTGAGGTAGATCATTTGAAAGGGCTAGATTATAAA
ATTAAGCCTTAGAGTATGGAAAGTTCTTATAACAATAATAGTACACACTTCAGAGTAAGACAAATGCAAA

GCATCTTAAGGAGTGAAAATAGAGTCTAAATCTTGCCTTTGGCACTACAAGGTGTGTGTGTGTGTGTGTG
TTGTGTGTCTTTAGTAGGAAATGGAAGAACACTGTTTTATTTTTTAAAGTGTTTAATGTTTCTGTCCTTT
CTGTGAATTATTGAATTTAAGAGCCCTGCTAAATAATGAAAAAACACTTTACTAAAATTTATCAAATTAT
ACTGGGTTCGGATTGTGAAAACATTGGCCACCTAGTAGCAGTGGTGAGGAGTGGGAGGGCCCAGCAAGCA
TTTATCAGAAATAGAATCACAATAGGAGGAGAATTTGGCTGTCTGATATTATGATTTGATTACAATACTG
AATGGGAAAAGTATCTAATATTTTGTAACAAAAAGACCTTCATATTATCTGTTTTGACCAAAATATGTAG
CTATTTCCCTTACACAGATTGGACCGCACTTATCTCCCTTGTCCTGTATCCTTTAATTTCAGGTCTCAGG
ATGTTTAGAAAGCTAAAACCCCCTACCCCTTTCTGGCTGAAAACTTGCCTTATTTGGTATCTTACACATT
AATGTTACTAGCATCAGGAGCTTACTGTTTTATTATGATTCATCTTCAGTAATTTTTAGAAGCAAGAAGA
AAGCCATTGTGTCCTCTACAATTAACAAAACTTATCTCTGATATACAAAGGGATATAAATATATACACTT
AAATAGAGAAAAAGAGGTTGATTGAATTGTGCCTTTGAGTGAACCCAGTTTTTAAATACCGCTGTGTTTG
TTTCGCCATGGCTTCAGGGATGCTACATGGCTCTTGCACCTTTTACTCCTCTGCTTTATGAAGTTTGAGT
TGTATTTGTGCATCTTAAAGTAGGTTGAGGCTTGAGGCTGGGCTTTCGGGTTTTTTTGTTTTTTGTTTTG
TTTTGTTTTGTTTTGTTTTCTTGTACTTAAACCTGCTTGCTTCCTACCACAGATTCTTTATTTTCCCAAA
CACTACAAAAAAACTTTTAAAACTTTGCCATTTCATCTGTTTACACTCTTTGCCACTGATTAGCAGTATT
TAAATCTTGCAAGAATATTTTGTGCTTTCTTTAGAAACACAAGAGTATAGATTTTTCTCACTGAAAAGTG
AGAGTTACGCATTGCAGCCATGAAGGGATGCTAGGATCAATTATGGCAGTACCTTTTTTCCCCTCCTGTT
CTTGAGCCAGTTGTCTCTTTTGTTTTGGGTCCCACTTAGGATTAACGGATGTAAGGTATTTTCCTGTGCC
TTTATTTTGTGTCATTCTATTGGAAGGAGGTGTAACGGCAGAATAGCATCGTGTTGGGGGTTTTCCTTCA
AACACTGCAAGTGATATTGCCACCATGTGAACCTCAAATATGCAATCCAGTTGTGTTGGTTTCTCGGTGA
CTTGGAGTGTTCATCTCTTCATGAATTGTGAGCACTGACCATGTTCTTCAGTTCTTAATTATGGTGAGTT
GACAAATACCAACTACTGCTTTTCTTTAGGTGGCTATAAATTTCTTACTGTCAGGAGGAAATGACATTAT
ATTCTGTTCCACTGAACGTCAGAGATCAGCAGGCACTGTACTGGGTAGAGAAGTGCCTATACTTCTCTAC
CTAAGAGGGCAGGAGGGAAACCCTACAGCTCCTTGTGAGCCTATATATTAGTATATCGGCCTGGAGAGGA
CAAGGGAATAAGACCACTCATAGTGAGGCTGGCCAAGCTGCACTGGTCGGACCAGGCAGTGGCTGACCTA
AGGAAGGCAACTTGCTTTGCTTAAAAGTAGATTTTTTAAGCAATGCTTAACACAGGCAGCATTCACCTTT
GTTCAGGCCATCGACATGTATTGTTAAAATTACTGCATATCCCCCTCAGATATCAAGTATACACTGTTCA
TGTTGGGGTTGTGTGTGTGTATGTGTGTATGTACGCACGCATGTGTCCCAAATCTTGTTTTAATTTTTTT
TTTCTGAATGTGATCATGTTTTGGATAATACCTGAGCAGGGTTGCCTTTTTTTTATTTATTACCATTATA
TATTATATTATATTATATATTTTTTGCTTTCTTATAACTTTGGAGGAAAGTCAAATCTTGGTATTATTAA
AATTGTTTTAAAAAGGAGTAAATTTTCCAGTTGATAAATGAAAATCACTGGCCTATGTTTAATAAGTTTT
TCTTTAATTACTGTGGAATAACGTGCCAGCTATCATCAACACAATGATTTTGTACATAGGGTAGGGAAGC
AGTGATGCTCTCAATGGGAAGATGTGCAACACAAATTAAGGGGAACTC**CATGTATTTTACCTACTTCAG**C
AATGGAACTGCAACTTGGGGCTTTGTGAATAAAATTTAGCTGCCTTGTATAGTCGTTTGAAAGAATATGT
GATCTGTGAGAGAATTATAGTTTTTTTTTAGAAGAAAAATCTGCAAAAGATCTTTCCAAAGACAATGTGC
CACAGATCTTTTGTTCTCTGTAATGAGGATTAATTGCTGTTTAAACAAAAATGTAATTGTTCATCTTTAA
ATTCTTTCCTTTTCATAAGAGGATCAAGCTGTAAAAAAACAAAAAAATTAATAAAAATTTCGAGAAATCA
AAAAAAAAAA

21) Entrez Gene ID 4302 = MLLT6, myeloid/lymphoid or mixed-lineage leukemia (trithorax homolog, Drosophila); translocated to, 6 = AL832481

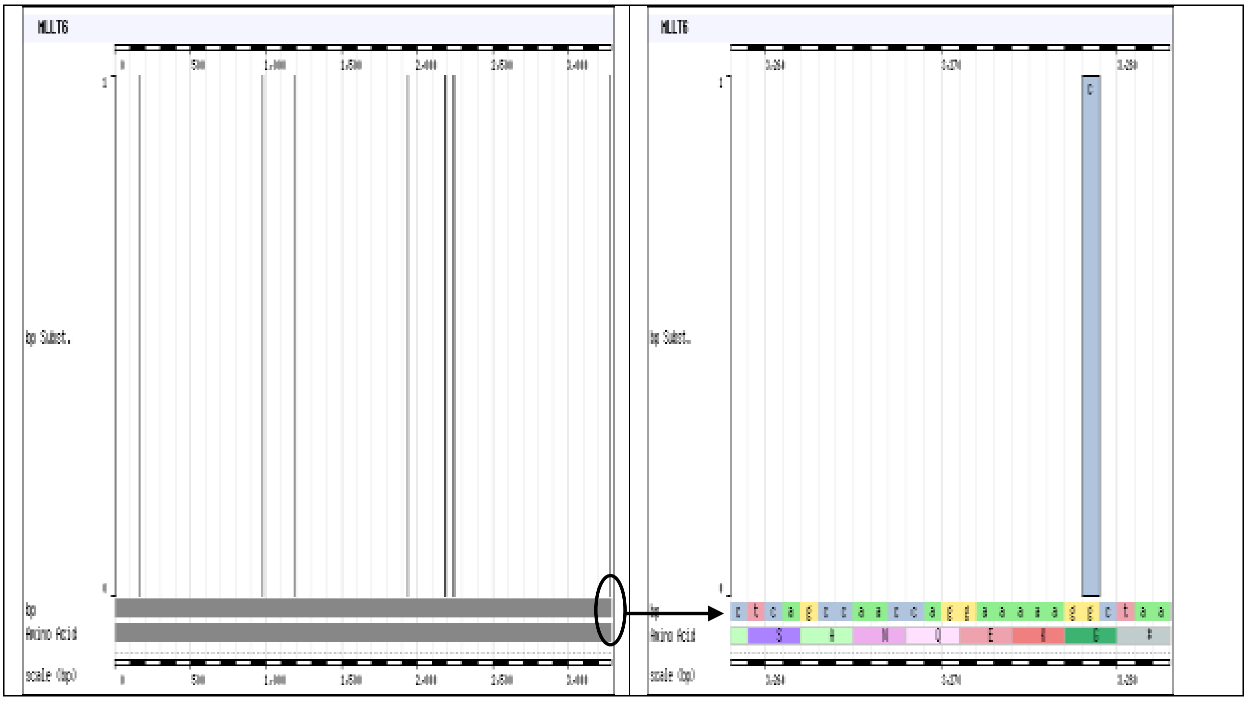

>gi|21733049|emb|AL832481.1| Homo sapiens mRNA; cDNA DKFZp686J1719 (from clone DKFZp686J1719)
GATACTGAAAATAATATTAATATTCCTGTTGATAAGACTTTGTAAGATGTTAGGGAGCTGATAATGGAGG
GGGGTGGGAATCCTTCAAAGGCAATTTCTTAGGCACTTGCAAGGGCTTGGGGGAGGGGGAGGCAGTTGTG
ATGACCTCAGAAATACTCACTTTTTATTAATGCTAAATATGTTAGAAAGAAATGATAGCATTCAGCATTT
TATTCTTCTTAATCTATTAAGCTGTGTAACTCCCTGCCCCAAACCACTGAAAAGAAAAGTAACCTTCAGG
CCAGGCGCGGTGGCTCACGCCTGTAATCCCAACACTTTGGGAGGCTGAGGCGGGCGGATCACTTAAGGTC
AGGAGTTCAAGACCAGCCTGGCCAACATGGTGAAACCCCGTCTCTATCAAAAATTAGCCGGGCATGGTGG
CACGTGCCTGTAATCCCAGCTACTTGGGAGGCTGAGGCACGAGAATTGCTTGAACCCAGGAGGCTGAGGC
TGCAGTGAGCCAAGATTGTGCCACTGCACTCCAGCCTGTGTGAGAGAGTGAGACTCTGTCTCAAAAGAGA
AAAAAAAAGAAAAGTAACCTTCAGAGATTCTTAGAAGAGTTGCTCATTCACACCCACGCCCTTGCCCAAG
GCTGGCCCACTTAGAGCGAAACTTAACTTTTGTCTGGATGGGAAGAGAAGTAAGTCTACCCCGAGGTTGC
CATGTTGAAGAGTGAGAGGTCCAAGTGATTCTGTGCATTGAAACCAAGACACCCCACCCAGAACACTTCT
TCCCTCCCTCAGCCCAAACCAAAGGCTGGGGTTCTCATCTCCAAGTGGCTGTTCTCCAACTTTCCCAAGC
CGCTTGCATTCCCCAGACTGGACTACTGTGGCGGTTAGGTTAGATTTGAAGACGGGGCCCAGGCTGGGTA
TGAACGGGTGCAGCCCTCTTCTCCTCTTCCCCCCCACATCTCTCATGAGAGAGGTAGTGGCATTTCCTTC
TCAGGGAGCTTCAATGGGAAAGGTCTCGAAAGCTTCAGGAGGAGCAGAATACCAACGCAGGGGGATGGCT
GTAACGATCTCACCGTCTCCTAACCTCAGTCCCTTTTTTGAGAGTGAATGGTGGAGGGTGGGAAGGGACC
CAGATTTGTAGATCTCTTTGTCTGGGGGAGGGGAAGGATGTGGTTTGCAGAGCGGAAGCAGAGTTTGGAA
ACGCATGAGAGCAGAGCTTCGTGTGTTCCCACCCTCAGTGAGGAGGTGTGAGTGGGGGTGCATATAGAGG
CAGTGCCTGCTGTGGGGTCACAACTGGTGCATGCCAGCGCCAAAGGGACCTGTCTTTAGGGGTCATTTCA
GCCAGCTCCTCCCATCACAGATGACAGCTCCAAGCCTAGAAGGGGCTCAGTGACAGGGCCAGGACAAGCC
CTCAGGACTGTGGCCTCCTGGCCCTTGGTTCCCCTGCCCCACAACATGGTCTCCACATGGCTGGCTGGCT
GGCTGTCCCTGTGTGTGTGTGACACACGGTGTGAGTGCAGGGCTGTGCCCGGGGTGGGAGGGTGTCTATG
TGGCACTGACTGTCTTAGCTCAGAGCTGGTGGATCCTCTCCATGGACAATGACACTTTAAGGATTGTCTT
GGTTTGTTTTTCCTATTTGTGGGGTATTTTCCCCCTCAGGCTCCTGGGTCTGCTGCTGCCTCAAGGTGTC
CTGACCTTGAGGCTGATGAGGGGACCCCTGCCTGTTTCCCCCATACTGAGTTCTAGGGAGGTGCTCACCC
CAGACTCTTAGGAAGGGTCTAGAGAAATGAGAGGAGCCCAAGCCAGGGGCCAGCTCCGAGAAAGGGTAAC
CTCCACGCTTCTCTCTCCCAAATTGGAAATGAAGACAGGTTTTCAAAGGCACAGGCTCCCCCTGCCAGCT
TCTAGGATCTTCCTTGGTGTGCAATGGGCCAGTTAGGGGTAGGCAGCTTGCACCCAGTTCTCCTTTATCT
CAACTTATTTTCCTGGGGAGAGGTGCCTAGAGGGATTGAGGTAACTTCAACTGGGAATTCCAAGGAAGGT
GGGCAAGTAGCCTTGGCTCTCTCCCACCATGTCCATCAGGATTGAGAGTGTGTCTAGCTCCCGACCACTT
TGTCTTGACCTACTGAAAAGTTGGGAACTGAGGGGTGCCTTCATTCCCCTTTGTTCACTTTCTCCAGCTC
AACTTGGGACTTGGGTGGTGGGACTGGAGACCTCACCCCTGCTCCCGTCCCGCCCCCTTTCTATCCCAAC
CTGTTTC**CATGTAGCAGACCCTTCCTAG**GGAGCAGGGAGGGGAAGCCACAGATTGCAAACCCAGGGGCTC
CTTTTTCATTCTTTCTAAAACCTTGATATCCTCAGCCCAAAGGCGATGCCCCCCTGCCACCTCCAAGCCT
GGAATTGTGCATAACCCGGATCTTGTATCTTTGTATAACGGATGTTATTTGTACGAAGGGCAGTTCGTAA

ACAGCACTTGTTCTTTTAATAAAAGAATGTTTTGCAAAAAAAAAAAAAAAAAAAAA

22) Entrez Gene ID 4303 = FOXO4, forkhead box O4 = BC026735

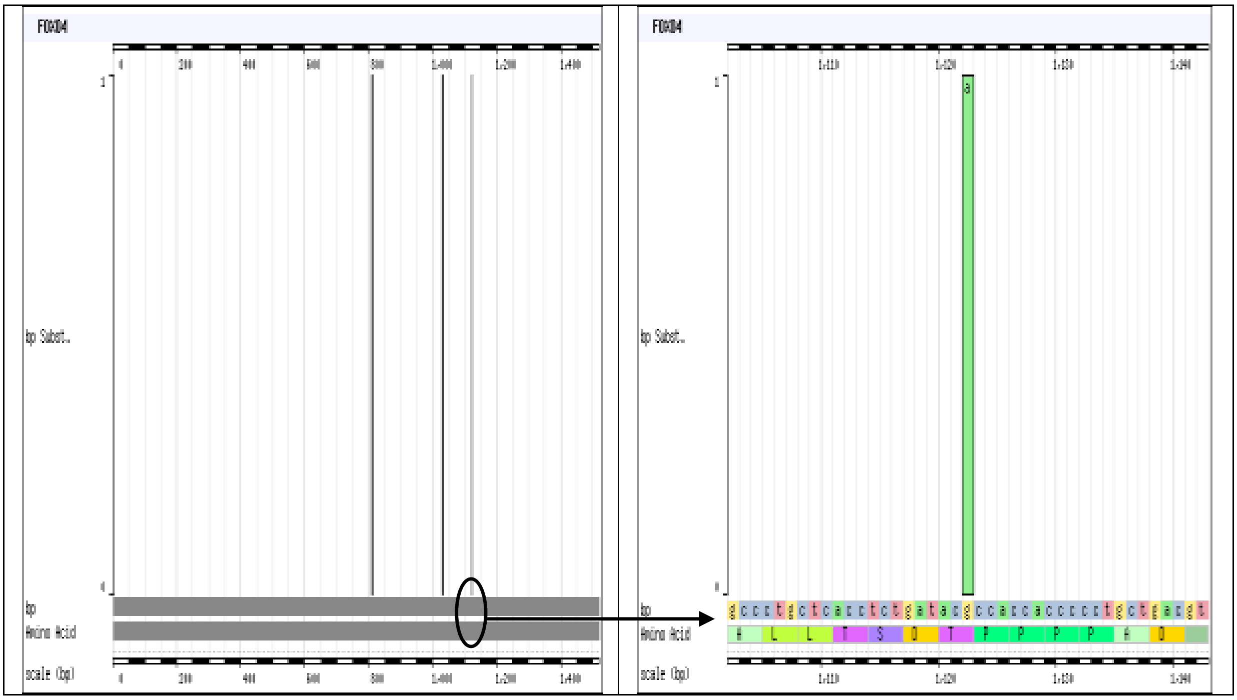

>gi|19934350|gb|BC026735.1| Homo sapiens forkhead box O4, mRNA (cDNA clone IMAGE:5106176)

CCACCGGCAAAAGCTCTTGGTGGATGCTGAACCCTGAGGGAGGCAAGAGCGGCAAAGCCCCCCGCCGCCG
GGCCGCCTCCATGGATAGCAGCAGCAAGCTGCTCCGGGGCCGCAGTAAAGCCCCCAAGAAGAAACCATCT
GTGCTGCCAGCTCCACCCGAAGGTGCCACTCCAACGAGCCCTGTCGGCCACTTTGCCAAGTGGTCAGGCA
GCCCTTGCTCTCGAAACCGTGAAGAAGCCGATATGTGGACCACCTTCCGTCCACGAAGCAGTTCAAATGC
CAGCAGTGTCAGCACCCGGCTGTCCCCCTTGAGGCCAGAGTCTGAGGTGCTGGCGGAGGAAATACCAGCT
TCAGTCAGCAGTTATGCAGGGGGTGTCCCTCCCACCCTCAATGAAGGTCTAGAGCTGTTAGATGGGCTCA
ATCTCACCTCTTCCCATTCCCTGCTATCTCGGAGTGGTCTCTCTGGCTTCTCTTTGCAGCATCCTGGGGT
TACCGGCCCCTTACACACCTACAGCAGCTCCCTTTTCAGCCCAGCAGAGGGGCCCCTGTCAGCAGGAGAA
GGGTGCTTCTCCAGCTCCCAGGCTCTGGAGGCCCTGCTCACCTCTGATAC**G**CCACCACCCCCTGCTGACG
TCCTCATGACCCAGGTAGATCCCATTCTGTCCCAGGCTCCGACTCTTCTGTTGCTGGGGGGGCTTCCTTC
CTCCAGTAAGCTGGCCACGGGCGTCGGCCTGTGTCCCAAGCCCCTAGAGGCTCCAGGCCCCAGCAGTCTG
GTTCCCACCCTTTCTATGATAGCACCACCTCCAGTCATGGCAAGTGCCCCCATCCCCAAGGCTCTGGGGA
CTCCTGTGCTCACACCCCCTACTGAAGCTGCAAGCCAAGACAGAATGCCTCAGGATCTAGATCTTGATAT
GTATATGGAGAACCTGGAGTGTGACATGGATAACATCATCAGTGACCTCATGGATGAGGGCGAGGGACTG
GACTTCAACTTTGAGCCAGATCCCTGAGTCATGCCTGGAAGCTTTGTCCCCTGCTTCAGATGTGGAGCCA
GGCGTGTTCATATCTACTCTTTACCCTTGAGCCCTCCCCAGGAATTTGGGACCCTGCTTTAGAGCTAGGG
TGGGGTCTGGTCACACACAGGTGTTGAAGAAATTATAAAGATAAAGCTGCCCCATCTGGGGACGATATGG
GGAGGGAGATGGGAGGGGAAAGGGGAGAGGGTTTTTCTCACTGTGCCAATTAGGGGGTAAGGCCCCCTCT
CAGGAGCCATCATCGGCTTTCCCCATTCCTACCCACTTAGGCTTTGTAGCAAGATGAGCAATGCTGTTGG
AAATGTGAAGTCACCAGTGGCCTTACCCCTGCCTTTGGGAGCAGGATTTTTTTGTAGAGAGTCTTATCTG
AGCTGAGCCAGGCTAGCTGGAGCCTGGGATTTCTATGCAGTGGCCCCTTAGGCCAGTGATGTGCGGTGGG
TGGGCTGTTTAGGGGATCTGGAAGGGCCAAGGTCTGAGCACTGGAGTGGCTCGCCAGGCCAAATCACCCT
TAGAAGGCTGCAGATAACAGAAAGGCTTTTTATAAACTTTTAAAGAAATATAAACACAAATATAGAGATT
TTTTAACCATGGCAGGGTGCTAGTGGTGGGCAGAATGCTTTTTTTTTCTTTCTGAAGGCTTTGTGATAGTG
ACATGATACAAACACTACAGACAATAAATATTAGGAGACACAGGGAAGTGGGGAGAGGTGGGGAGTAATA
GTAAACACAGGGAAGAGCTCCCCTACGGACCAGGTATAGAGAAAGGTCTATGCAGAAATAGGTTAGAGTT
TCCCTAACAAAAAAGCTAACCCAGGTCCCCTCATTCCTTCAACTTGTGCCTGGGAGTGTGTGGTGTTAGG

GTGCAGCCACACTCTTCTATGACCCAGCATGGGTTAGTGCTATGGTGGGAGAGTACATTGAAGGCCTGGA
ATTAGCTTGGGGCCAGGGAAGGGACTGGGAGGGGAGAGAAGAGAAGGAGGGAAGGATTTAGGATGGTAAA
GTTAGGTACAGAGACCTCCCTGTTCAAGGCCCCTGACAGCTGTCCCTGCCCTTCTTCCCCTTCCCTGACT
GCAGGGGTTATGTGGAAGTGTGTGTGGCAGCAGGCAGCGGGGAGGGGAGGAACAGGGAAGGGGGAGCTGG
GGAGCTTGGCTGAGGGTCTGGGAAATGAGCAGGGATGGGGGGGGATGTGGATCAGGTTTACTAGCACCTG
CCAGGGAGGCCATCTGGGGCTCCTTCTCCACCCCAGCCCCCAAAGCAGCCCTTCCCCCAGTGCCCTTTGC
ATCGTCCCCTCCCCCACCCCTGCTGTGGGTTCCCATCATTTCCTGTGTCAGCGCCTGGCCTACCCAGATT
GTAT**CATGTGCTAGATTGGAGTGGG**GAAGTGTGTCAAATCAATAAATGAATAAATTCAATAAATGCCTAT
AACCAAAAAAAAAAAAAAA

23) Entrez Gene ID 4615 = MYD88, myeloid differentiation primary response gene (88) = U70451

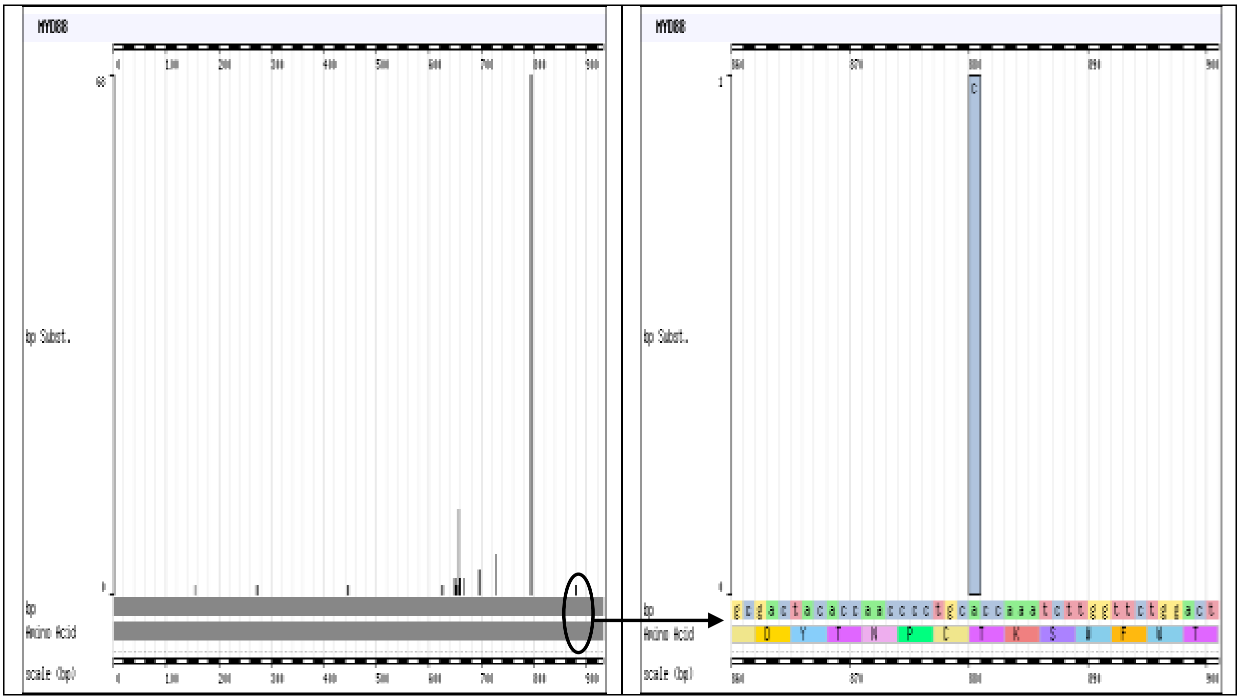

>gi|1763090|gb|U70451.1|HSU70451 Human myleoid differentiation primary response protein MyD88 mRNA, complete cds
GGGTAGACCCACGAGTCCGCCCACGGGTCTGCATGGCTGCAGGAGGTCCCGGCGCGGGGTCTGCGGCCCC
GGTCTCCTCCACATCCTCCCTTCCCCTGGCTGCTCTCAACATGCGAGTGCGGCGCCGCCTGTCTCTGTTC
TTGAACGTGCGGACACAGGTGGCGGCCGACTGGACCGCGCTGGCGGAGGAGATGGACTTTGAGTACTTGG
AGATCCGGCAACTGGAGACACAAGCGGACCCCACTGGCAGGCTGCTGGACGCCTGGCAGGGACGCCCTGG
CGCCTCTGTAGGCCGACTGCTCGAGCTGCTTACCAAGCTGGGCTGCGACGACGTGCTGCTGGAGCTGGGA
CCCAGCATTGAGGAGGATTGCCAAAAGTATATCTTGAAGCAGCAGCAGGAGGAGGCTGAGAAGCCTTTAC
AGGTGGCCGCTGTAGACAGCAGTGTCCCACGGACAGCAGAGCTGGCGGGCATCACCACACTTGATGACCC
CCTGGGGCATATGCCTGAGCGTTTCGATGCCTTCATCTGCTATTGCCCCAGCGACATCCAGTTTGTGCAG
GAGATGATCCGGCAACTGGAACAGACAAACTATCGACTGAAGTTGTGTGTGTCTGACCGCGATGTCCTGC
CTGGCACCTGTGTCTGGTCTATTGCTAGTGAGCTCATCGAAAAGAGGTGCCGCCGGATGGTGGTGGTTGT
CTCTGATGATTACCTGCAGAGCAAGGAATGTGACTTCCAGACCAAATTTGCACTCAGCCTCTCTCCAGGT
GCCCATCAGAAGCGACTGATCCCCATCAAGTACAAGGCAATGAAGAAAGAGTTCCCCAGCATCCTGAGGT
TCATCACTGTCTGCGACTACACCAACCCCTGC**A**CCAAATCTTGGTTCTGGACTCGCCTTGCCAAGGCCTT
GTCCCTGCCCTGAAGACTGTTCTGAGGCCCTGGGTGTGTGTGTATCTGTCTGCCTGTCCATGTACTTCTG
CCCTGCCTCCTCCTTTCGTTGTAGGAGGAATCTGTGCTCTACTTACCTCTCAATTCCTGGAGATGCCAAC
TTCACAGACACGTCTGCAGCAGCTGGACATCACATTTCATGTCCTGCATGGAACCAGTGGCTGTGAGTGG
CATGTCCACTTGCTGGATTATCAGCCAGGACACTATAGAACAGGACCAGCTGAGACTAAGAAGGACCAGC
AGAGCCAGCTCAGCTCTGAGCCATTCACACATCTTCACCCTCAGTTTCCTCACTTGAGGAGTGGGATGGG
GAGAACAGAGAGTAGCTGTGTTTGAATCCCTGTAGGAAATGGTGAAGCATAGCTCTGGGTCTCCTGGGGG
AGACCAGGCTTGGCTGCGGGAGAGCTGGCTGTTGCTGGACTACATGCTGGCCACTGCTGTGACCACGACA
CTGCTGGGGCAGCTTCTTCCACAGTGATGCCTACTGATGCTTCAGTGCCTCTGCACACCGCCCATTCCAC

TTCCTCCTTCCCCACAGGGCAGGTGGGGAAGCAGTTTGGCCCAGCCCAAGGAGACCCCACCTTGAGCCTT
ATTTCCTAATGGGTCCACCTCTCATCTGCATCTTTCACACCTCCCAGCTTCTGCCCAACCTTCAGCAGTG
ACAAGTCCCCAAGAGACTCGCCTGAGCAGCTTGGGCTGCTTTTCATTTCCACCTGTCAGGATGCCTGTGG
TCATGCTCTCAGCTCCACCTGGCATGAGAAGGGATCCTGGCCTCTGGCATATTCATCAAGTATGAGTTCT
GGGGATGAGTCACTGTAATGATGTGAGCAGGGAGCCTTCCTCCCTGGGCCACCTGCAGAGAGCTTTCCCA
CCAACTTTGTACCTTGATTGCCTTACAAAGTTATTTGTTTACAAACAGCGACCATATAAAAGCCTCCTGC
CCCAAAGCTTGTGGGCACATGGGCACATACAGACTCACATACAGACACACACATATATGTACAGACATGT
ACTCTCACACACACAGGCACCAGCATACACACGTTTTTCTAGGTACAGCTCCCAGGAACAGCTAGGTGGG
AAAGTCCCATCACTGAGGGAGCCTAACCATGTCCCTGAACAAAAATTGGGCACTCATCTATTCCTTTTCT
CTTGTGTCCCTACTCATTGAAACCAAACTCTGGAAAGGACCCAATGTACCAGTATTTATACCTCTAATGA
AGCACAGAGAGAGGAAGAGAGCTGCTTAAACTCACACAACAATGAACTGCAGACACAGCTGTTCTCTCCC
TCTCTCCTTCCCAGAGCAATTTATACTTTACCCTCAGGCTGTCCTCTGGGGAGAAGGTGCCATGGTCTTA
GGTGTCTGTGCCCCAGGACAGACCCTAGGACCCTAAATCCAATAGAAAATGCATATCTTTGCTCCACTTT
CAGCCAGGCTGGAGCAAGGTACCTTTTCTTAGGATCTTGGGAGGGAATGGATGCCCCTCTCTGCATGATC
TTGTTGAGGCATTTAGCTGCCATGCACCTGTCCCCCTTTAATACTGGGCATTTTAAAGCCATCTCAAGAG
GCATCTTCTA**CATGTTTTGTACGCATTAAAA**TAATTTCAAAGATATCTGAGAAAAGCCGATATTTGCCAT
TCTTCCTATATCCTGGAATATATCTTGCATCCTGAGTTTATAATAATAAATAATATTCTACCTTGGAAAA
AAAAAAAAAAAAAAAAAAAAAA

24) Entrez Gene ID 4780 = NFE2L2, nuclear factor (erythroid-derived 2)-like 2= BC011558

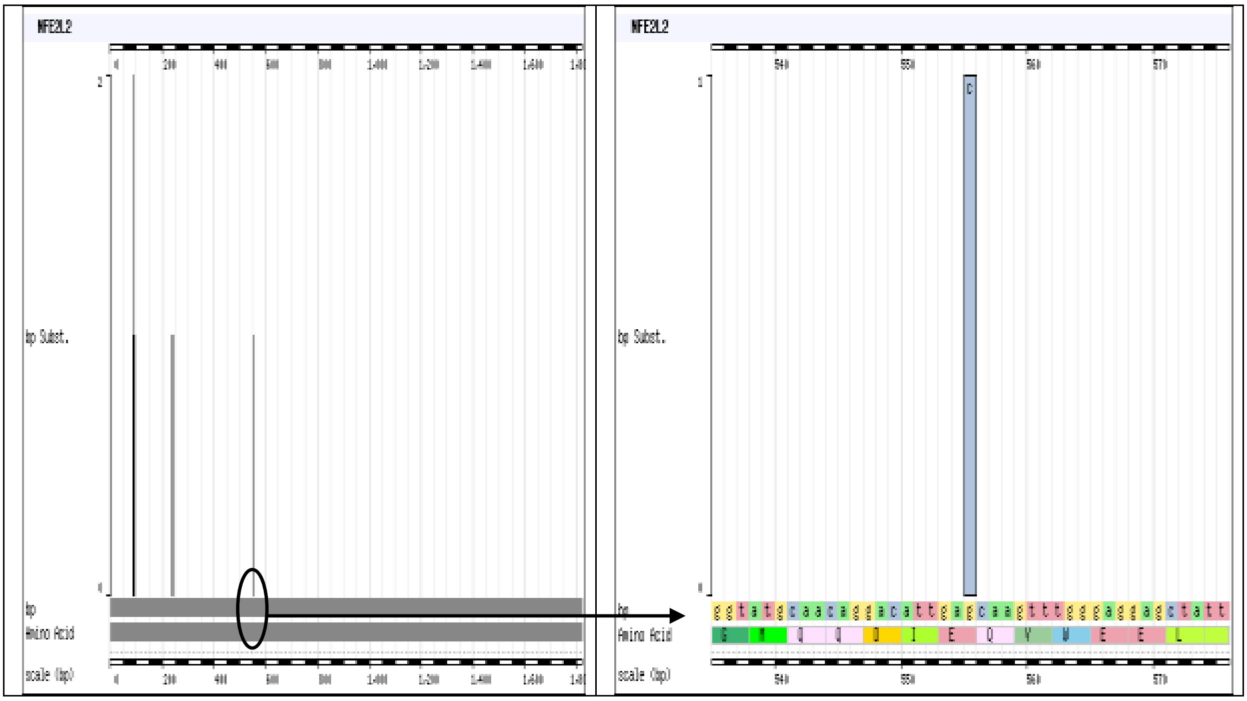

>gi|15079436|gb|BC011558.1| Homo sapiens nuclear factor (erythroid-derived 2)-like 2, mRNA (cDNA clone MGC:20033 IMAGE:4548874), complete cds
GCAGCCGCCACCGCCGCCGCCGCCGCCACCAGAGCCGCCCTGTCCGCGCCGCGCCTCGGCAGCCGGAACA
GGGCCGCCGTCGGGGAGCCCCAACACACGGTCCACAGCTCATCATGATGGACTTGGAGCTGCCGCCGCCG
GGACTCCCGTCCCAGCAGGACATGGATTTGATTGACATACTTTGGAGGCAAGATATAGATCTTGGAGTAA
GTCGAGAAGTATTTGACTTCAGTCAGCGACGGAAAGAGTATGAGCTGGAAAAACAGAAAAAACTTGAAAA
GGAAAGACAAGAACAACTCCAAAAGGAGCAAGAGAAAGCCTTTTTCGCTCAGTTACAACTAGATGAAGAG
ACAGGTGAATTTCTCCCAATTCAGCCAGCCCAGCACATCCAGTCAGAAACCAGTGGATCTGCCAACTACT
CCCAGGTTGCCCACATTCCCAAATCAGATGCTTTGTACTTTGATGACTGCATGCAGCTTTTGGCGCAGAC
ATTCCCGTTTGTAGATGACAATGAGGTTTCTTCGGCTACGTTTCAGTCACTTGTTCCTGATATTCCCGGT
CACATCGAGAGCCCAGTCTTCATTGCTACTAATCAGGCTCAGTCACCTGAAACTTCTGTTGCTCAGGTAG
CCCCTGTTGATTTAGACGGTATGCAACAGGACATTGA**G**CAAGTTTGGGAGGAGCTATTATCCATTCCTGA
GTTACAGTGTCTTAATATTGAAAATGACAAGCTGGTTGAGACTACCATGGTTCCAAGTCCAGAAGCCAAA

CTGACAGAAGTTGACAATTATCATTTTTACTCATCTATACCCTCAATGGAAAAAGAAGTAGGTAACTGTA
GTCCACATTTTCTTAATGCTTTTGAGGATTCCTTCAGCAGCATCCTCTCCACAGAAGACCCCAACCAGTT
GACAGTGAACTCATTAAATTCAGATGCCACAGTCAACACAGATTTTGGTGATGAATTTTATTCTGCTTTC
ATAGCTGAGCCCAGTATCAGCAACAGCATGCCCTCACCTGCTACTTTAAGCCATTCACTCTCTGAACTTC
TAAATGGGCCCATTGATGTTTCTGATCTATCACTTTGCAAAGCTTTCAACCAAAACCACCCTGAAAGCAC
AGCAGAATTCAATGATTCTGACTCCGGCATTTCACTAAACACAAGTCCCAGTGTGGCATCACCAGAACAC
TCAGTGGAATCTTCCAGCTATGGAGACACACTACTTGGCCTCAGTGATTCTGAAGTGGAAGAGCTAGATA
GTGCCCCTGGAAGTGTCAAACAGAATGGTCCTAAAACACCAGTACATTCTTCTGGGGATATGGTACAACC
CTTGTCACCATCTCAGGGGCAGAGCACTCACGTGCATGATGCCCAATGTGAGAACACACCAGAGAAAGAA
TTGCCTGTAAGTCCTGGTCATCGGAAAACCCCATTCACAAAAGACAAACATTCAAGCCGCTTGGAGGCTC
ATCTCACAAGAGATGAACTTAGGGCAAAAGCTCTCCATATCCCATTCCCTGTAGAAAAAATCATTAACCT
CCCTGTTGTTGACTTCAACGAAATGATGTCCAAAGAGCAGTTCAATGAAGCTCAACTTGCATTAATTCGG
GATATACGTAGGAGGGGTAAGAATAAAGTGGCTGCTCAGAATTGCAGAAAAAGAAAACTGGAAAATATAG
TAGAACTAGAGCAAGATTTAGATCATTTGAAAGATGAAAAAGAAAAATTGCTCAAAGAAAAAGGAGAAAA
TGACAAAAGCCTTCACCTACTGAAAAAACAACTCAGCACCTTATATCTCGAAGTTTTCAG**CATGCTACGT
GATGAAGATGG**AAAACCTTATTCTCCTAGTGAATACTCCCTGCAGCAAACAAGAGATGGCAATGTTTTCC
TTGTTCCCAAAAGTAAGAAGCCAGATGTTAAGAAAAACTAGATTTAGGAGGATTTGACCTTTTCTGAGCT
AGTTTTTTTGTACTATTATACTAAAAGCTCCTACTGTGATGTGAAATGCTCATACTTTATAAGTAATTCT
ATGCAAAATCATAGCCAAAACTAGTATAGAAAATAATACGAAACTTTAAAAAGCATTGGAGTGTCAGTAT
GTTGAATCAGTAGTTTCACTTTAACTGTAAACAATTTCTTAGGACACCATTTGGGCTAGTTTCTGTGTAA
GTGTAAATACTACAAAAACTTATTTATACTGTTCTTATGTCATTTGTTATATTCATAGATTTATATGATG
ATATGACATCTGGCTAAAAAGAAATTATTGCAAAACTAACCACTATGTACTTTTTTATAAATACTGTATG
GACAAAAAATGGCATTTTTTATATTAAATTGTTTAGCTCTGGCAAAAAAAAAAAAATTTTAAGAGCTGGTA
CTAATAAAGGATTATTATGACTGTTAAAAAAAAAAAAAAAAAAAAAAAAAAAAAAAAA

25) Entrez Gene ID 4841 = NONO, non-POU domain containing, octamer-binding = BC003129

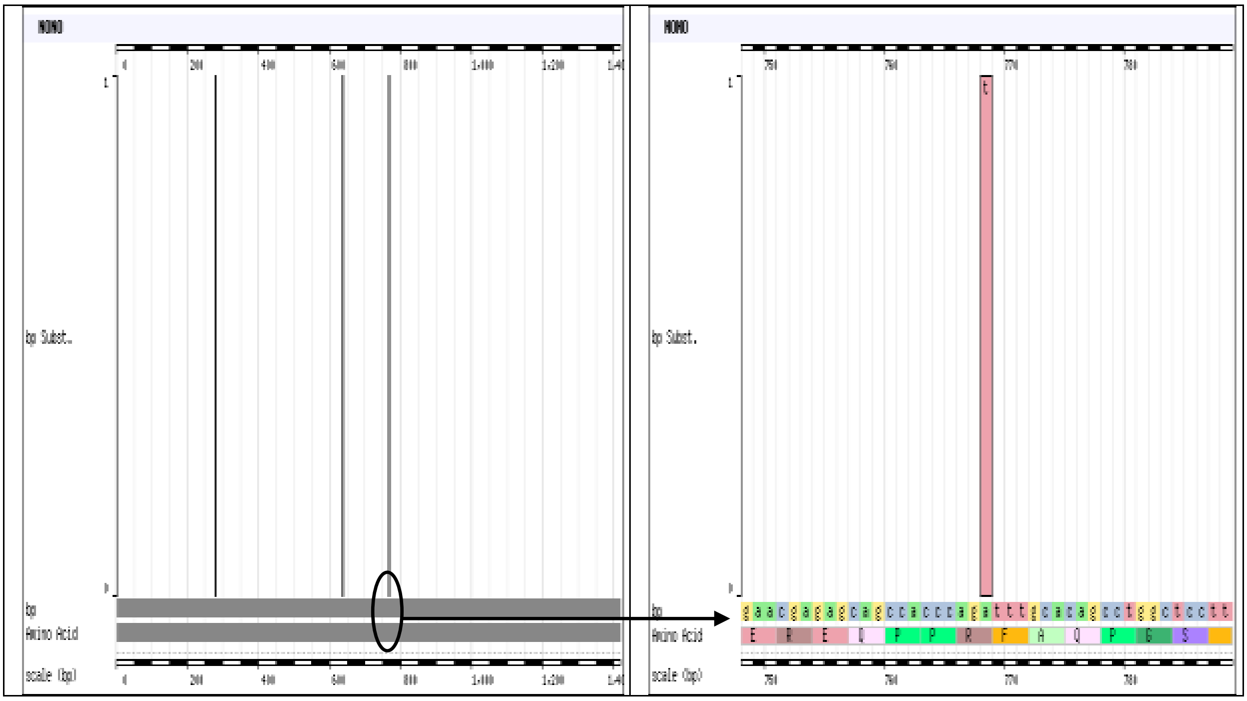

>gi|13111916|gb|BC003129.1| Homo sapiens non-POU domain containing, octamer-binding, mRNA (cDNA clone MGC:3380 IMAGE:2957871), complete cds
CTTTTCTCGGGACGGGAGAGGCCGTGTAGCGTCGCCGTTACTCCGAGGAGATACCAGTCGGTAGAGGAGA
AGTCGAGGTTAGAGGGAACTGGGAGGCACTTTGCTGTCTGCAATCGAAGTTGAGAGGCCCAGTATTTAGG
CGACAGTGAATTTATTACTCTGAAGAGGGTTCTGCACATATTTCCAAATTATATTGGTGGTCATCAGAAG
TAGGTGATAGGAAGAAATACTTCTCAAGGGTGCAAAAATGCAGAGTAATAAAACTTTTAACTTGGAGAAG
CAAAACCATACTCCAAGAAAGCATCATCAACATCACCACCAGCAGCAGCACCACCAGCAGCAACAGCAGC
AGCCGCCACCACCGCCAATACCTGCAAATGGGCAACAGGCCAGCAGCCAAAATGAAGGCTTGACTATTGA
CCTGAAGAATTTTAGAAAACCAGGAGAGAAGACCTTCACCCAACGAAGCCGTCTTTTTGTGGGAAATCTT
CCTCCCGACATCACTGAGGAAGAAATGAGGAAACTATTTGAGAAATATGGAAAGGCAGGCGAAGTCTTCA
TTCATAAGGATAAAGGATTTGGCTTTATCCGCTTGGAAACCCGAACCCTAGCGGAGATTGCCAAAGTGGA
GCTGGACAATATGCCACTCCGTGGAAAGCAGCTGCGTGTGCGCTTTGCCTGCCATAGTGCATCCCTTACA
GTTCGAAACCTTCCTCAGTATGTGTCCAACGAACTGCTGGAAGAAGCCTTTTCTGTGTTTGGCCAGGTAG
AGAGGGCTGTAGTCATTGTGGATGATCGAGGAAGGCCCTCAGGAAAAGGCATTGTTGAGTTCTCAGGGAA
GCCAGCTGCTCGGAAAGCTCTGGACAGATGCAGTGAAGGCTCCTTCCTGCTAACCACATTTCCTCGTCCT
GTGACTGTGGAGCCCATGGACCAGTTAGATGATGAAGAGGGACTTCCAGAGAAGCTGGTTATAAAAAACC
AGCAATTTCACAAGGAACGAGAGCAGCCACCCAGATTTGCACAGCCTGGCTCCTTTGAGTATGAATATGC
CATGCGCTGGAAGGCACTCATTGAGATGGAGAAGCAGCAGCAGGACCAAGTGGACCGCAACATCAAGGAG
GCTCGTGAGAAGCTGGAGATGGAGATGGAAGCTGCACGCCATGAGCACCAGGTCATGCTAATGAGACAGG
ATTTGATGAGGCGCCAAGAAGAACTTCGGAGGATGGAAGAGCTGCACAACCAAGAGGTGCAAAAACGAAA
GCAACTGGAGCTCAGGCAGGAGGAAGAGCGCAGGCGCCGTGAAGAAGAGATGCGGCGGCAGCAAGAAGAA
ATGATGCGGCGACAGCAGGAAGGATTCAAGGGAACCTTCCCTGATGCGAGAGAGCAGGAGATTCGGATGG
GTCAGATGGCTATGGGAGGTGCTATGGGCATAAACAACAGAGGTGCCATGCCCCCTGCTCCTGTGCCAGC
TGGTACCCCAGCTCCTCCAGGACCTGCCACTATGATGCCGGATGGAACTTTGGGATTGACCCCACCAACA
ACTGAACGCTTTGGTCAGGCTGCTACAATGGAAGGAATTGGGGCAATTGGTGGAACTCCTCCTGCATTCA
ACCGTGCAGCTCCTGGAGCTGAATTTGCCCCAAACAAACGTCGCCGATACTAATAAGTTGCAGTGTCTAG
TTTCTCAAAACCCTTAAAAGAAGGACCCTTTTTGGACTAGCCAGAATTCTACCCTGGAAAAGTGTTAGGG
ATTCCTTCCAATAGTTAGATCTACCCTGCCTGTACTACTCTAGGGAGTATGCTGGAGGCAGAGGGCAAGG
GAGGGGTGGTATTAAACAAGTCAATTCTGTGTGGTAAAAAAAAAAAAAAAAAAAAAAAAAAA

26) Entrez Gene ID 4851 = NOTCH1, notch 1 = BC013208

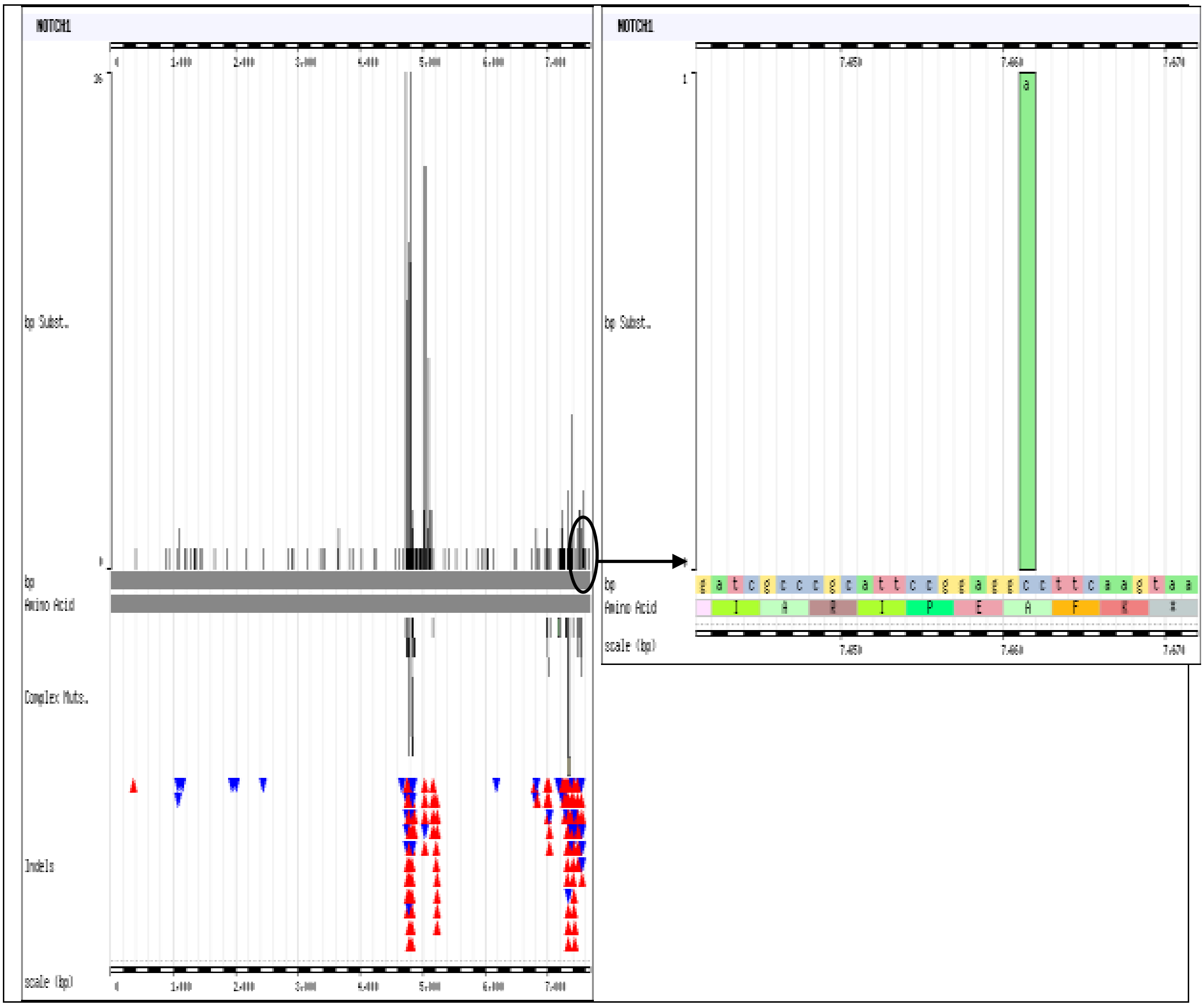

>gi|15301451|gb|BC013208.1| Homo sapiens Notch homolog 1, translocation-associated (Drosophila), mRNA (cDNA clone IMAGE:4156263)

CACGCTCTGATGCCGCCAAGCGCCTGCTGGAGGCCAGCGCAGATGCCAACATCCAGGACAACATGGGCCG
CACCCCGCTGCATGCGGCTGTGTCTGCCGACGCACAAGGTGTCTTCCAGATCCTGATCCGGAACCGAGCC
ACAGACCTGGATGCCCGCATGCATGATGGCACGACGCCACTGATCCTGGCTGCCCGCCTGGCCGTGGAGG
GCATGCTGGAGGACCTCATCAACTCACACGCCGACGTCAACGCCGTAGATGACCTGGGCAAGTCCGCCCT
GCACTGGGCCGCCGCCGTGAACAATGTGGATGCCGCAGTTGTGCTCCTGAAGAACGGGGCTAACAAAGAT
ATGCAGAACAACAGGGAGGAGACACCCCTGTTTCTGGCCGCCCGGGAGGGCAGCTACGAGACCGCCAAGG
TGCTGCTGGACCACTTTGCCAACCGGGACATCACGGATCATATGGACCGCCTGCCGCGCGACATCGCACA
GGAGCGCATGCATCACGACATCGTGAGGCTGCTGGACGAGTACAACCTGGTGCGCAGCCCGCAGCTGCAC
GGAGCCCCGCTGGGGGCACGCCCACCCTGTCGCCCCCGCTCTGCTCGCCCAACGGCTACCTGGGCAGCC
TCAAGCCCGGCGTGCAGGGCAAGAAGGTCCGCAAGCCCAGCAGCAAAGGCCTGGCCTGTGGAAGCAAGGA
GGCCAAGGACCTCAAGGCACGGAGGAAGAAGTCCCAGGATGGCAAGGGCTGCCTGCTGGACAGCTCCGGC
ATGCTCTCGCCCGTGGACTCCCTGGAGTCACCCCATGGCTACCTGTCAGACGTGGCCTCGCCGCCACTGC
TGCCCTCCCCGTTCCAGCAGTCTCCGTCCGTGCCCCTCAACCACCTGCCTGGGATGCCCGACACCCACCT
GGGCATCGGGCACCTGAACGTGGCGGCCAAGCCCGAGATGGCGGCGCTGGGTGGGGGCGGCCGGCTGGCC
TTTGAGACTGGCCCACCTCGTCTCTCCCACCTGCCTGTGGCCTCTGGCACCAGCACCGTCCTGGGCTCCA
GCAGCGGAGGGGCCCTGAATTTCACTGTGGGCGGGTCCACCAGTTTGAATGGTCAATGCGAGTGGCTGTC
CCGGCTGCAGAGCGGCATGGTGCCGAACCAATACAACCCTCTGCGGGGGAGTGTGGCACCAGGCCCCCTG
AGCACACAGGCCCCCTCCCTGCAGCATGGCATGGTAGGCCCGCTGCACAGTAGCCTTGCTGCCAGCGCCC
TGTCCCAGATGATGAGCTACCAGGGCCTGCCCAGCACCCGGCTGGCCACCCAGCCTCACCTGGTGCAGAC
CCAGCAGGTGCAGCCACAAAACTTACAGATGCAGCAGCAGAACCTGCAGCCAGCAAACATCCAGCAGCAG
CAAAGCCTGCAGCCGCCACCACCACCACCACAGCCGCACCTTGGCGTGAGCTCAGCAGCCAGCGGCCACC
TGGGCCGGAGCTTCCTGAGTGGAGAGCCGAGCCAGGCAGACGTGCAGCCACTGGGCCCCAGCAGCCTGGC
GGTGCACACTATTCTGCCCCAGGAGAGCCCCGCCCTGCCCACGTCGCTGCCATCCTCGCTGGTCCCACCC
GTGACCGCAGCCCAGTTCCTGACGCCCCCCTCGCAGCACAGCTACTCCTCGCCTGTGGACAACACCCCCA
GCCACCAGCTACAGGTGCCTGAGCACCCCTTCCTCACCCCGTCCCCTGAGTCCCCTGACCAGTGGTCCAG

CTCGTCCCCGCATTCCAACGTCTCCGACTGGTCCGAGGGCGTCTCCAGCCCTCCCACCAGCATGCAGTCC
CAGATCGCCCGCATTCCGGAGGCCTTCAAGTAAACGGCGCGCCCCACGAGACCCCGGCTTCCTTTCCCAA
GCCTTCGGGCGTCTGTGTGCGCTCTGTGGATGCCAGGGCCGACCAGAGGAGCCTTTTTAAAACACATGTT
TTTATACAAAATAAGAACAAGGATTTTAATTTTTTTTAGTATTTATTTATGTACTTTTATTTTACACAGA
AACACTGCCTTTTTATTTATATGTACTGTTTTATCTGGCCCCAGGTAGAAACTTTTATCTATTCTGAGAA
AACAAGCAAGTTCTGAGAGCCAGGGTTTTCCTACGTAGGATGAAAAGATTCTTCTGTGTTTATAAAATAT
AAACAAAGATTCATGATTTATAAATGCCATTTATTTATTGATTCCTTTTTTCAAAATCCAAAAAGAAATG
ATGTTGGAGAAGGGAAGTTGAACGAGCATAGTCCAAAAAGCTCCTGGGGCGTCCAGGCCGCGCCCTTTCC
CCGACGCCCACCCAACCCCAAGCCAGCCCGGCCGCTCCACCAGCATCACCTGCCTGTTAGGAGAAGCTGC
ATCCAGAGGCAAACGGAGGCAAAGCTGGCTCACCTTCCGCACGCGGATTAATTTGCATCTGAAATAGGAA
ACAAGTGAAAGCATATGGGTTAGATGTTGCCATGTGTTTTAGATGGTTTCTTGCCAGCATGCTTGTGAAA
ATGTGTTCTCGGAGTGTGTATGCCAAGAGTGCACCCATGGTACCAATCATGAATCTTTGTTTCAGGTTCA
GTATTATGTAGTTGTTCGTTGGTTATACAAGTTCTTGGTCCCTCCAGAACCACCCCGGCCCCCTGCCCGT
TCTTGAAATGTAGGCATCATGCATGTCAAACATGAGATGTGTGGACTGTGGCACTTGCCTGGGTCACACA
CGGAGGCATCCTACCCTTTTCTGGGGAAAGACACTGCCTGGGCTGACCCCGGTGGCGGCCCCAGCACCTC
AGCCTGCACAGTGTCCCCCAGGTTCCGAAGAAGATGCTCCAGCAACACAGCCTGGGCCCCAGCTCGCGGG
ACCCGACCCCCCGTGGGCTCCCGTGTTTTGTAGGAGACTTGCCAGAGCCGGGCACATTGAGCTGTGCAAC
GCCGTGGGCTGCGTCCTTTGGTCCTGTCCCCGCAGCCCTGGCAGGGGGCATGCGGTCGGGCAGGGGCTGG
AGGGAGGCGGGGGCTGCCCTTGGGCCACCCCTCCTAGTTTGGGAGGAGCAGATTTTTGCAATACCAAGTA
TAGCCTATGGCAGAAAAAATGTCTGTAAATATGTTTTTAAAGGTGGATTTTGTTTAAAAAATCTTAATGA
ATGAGTCTGTTGTGTGTCATGCCAGTGAGGGACGTCAGACTTGGCTCAGCTCGGGGAGCCTTAGCCGCCC
ATGCACTGGGGACGCTCCGCTGCCGTGCCGCCTGCACTCCTCAGGGCAGCCTCCCCCGGCTCTACGGGGG
CCGCGTGGTGCCATCCCCAGGGGGCATGACCAGATGCGTCCCAAGATGTTGATTTTTACTGTGTTTTATA
AAATAGAGTGTAGTTTACAGAAAAAGACTTTAAAAGTGATCTACATGAGGAACTGTAGATGATGTATTTT
TTTCATCTTTTTTGTTAACTGATTTGCAATAAAAATGATACTGATGGTGAAAAAAAAAAAAAAA

27) Entrez Gene ID 4853 = NOTCH2, notch 2 = AL049386

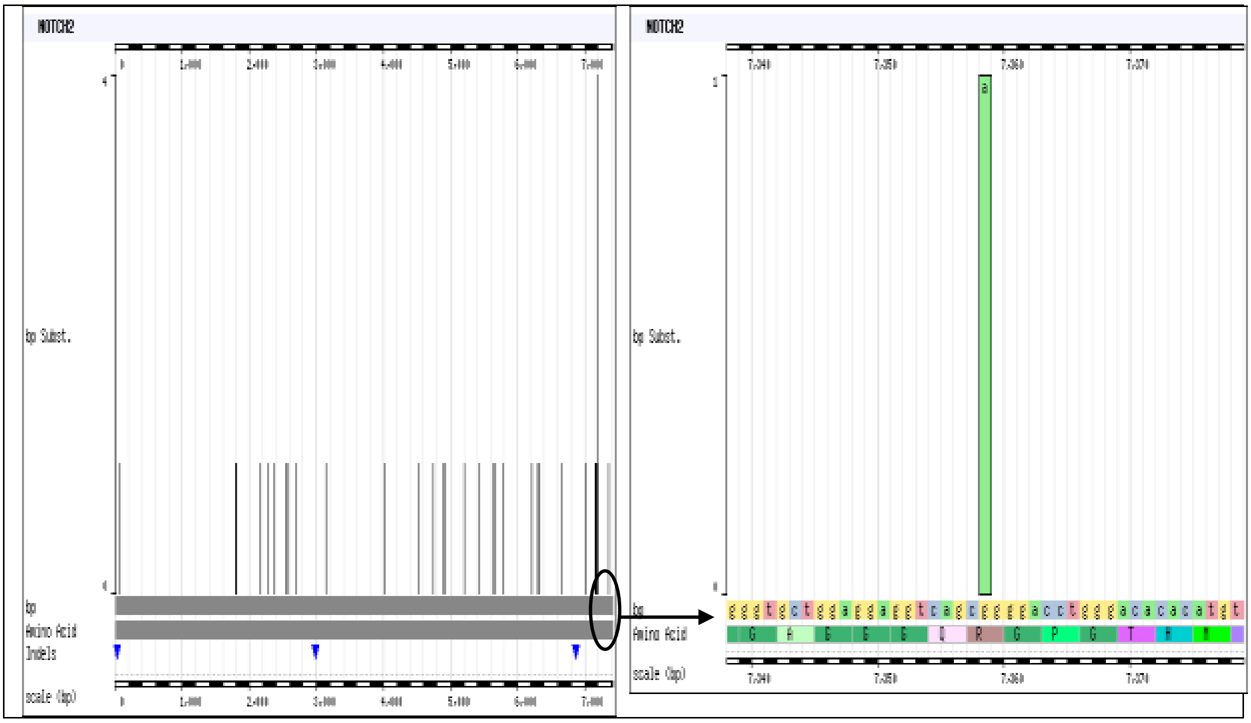

>gi|4500176|emb|AL049386.1| Homo sapiens mRNA; cDNA DKFZp586M0918 (from clone DKFZp586M0918)
CATATCCACAGAAGACACTGTCTCAAATGTTGTACCCTTGCCATTTAGGACTGAACTTTCCTTAGCCCAA
GGGACCCAGTGACAGTTGTCTTCCGTTTGTCAGATGATCAGTCTCTACTGATTATCTTGCTGCTTAAAGG
CCTGCTCACCAATCTTTCTTTCACACCGTGTGGTCCGTGTTACTGGTATACCCAGTATGTTCTCACTGAA
GACATGGACTTTATATGTTCAAGTGCAGGAATTGGAAAGTTGGACTTGTTTTCTATGATCCAAAACAGCC
CTATAAGAAGGTTGGAAAAGGAGGAACTATATAGCAGCCTTTGCTATTTTCTGCTACCATTTCTTTTCCT

CTGAAGCGGCCATGACATTCCCTTTGGCAACTAACGTAGAAACTCAACAGAACATTTTCCTTTCCTAGAG
TCACCTTTTAGATGATAATGGACAACTATAGACTTGCTCATTGTTCAGACTGATTGCCCCTCACCTGAAT
CCACTCTCTGTATTCATGCTCTTGGCAATTTCTTTGACTTTCTTTTAAGGGCAGAAGCATTTTAGTTAAT
TGTAGATAAAGAATAGTTTTCTTCCTCTTCTCCTTGGGCCAGTTAATAATTGGTCCATGGCTACACTGCA
ACTTCCGTCCAGTGCTGTGATGCCCATGACACCTGCAAAATAAGTTCTGCCTGGGCATTTTGTAGATATT
AACAGGTGAATTCCCGACTCTTTTGGTTTGAATGACAGTTCTCATTCCTTCTATGGCTGCAAGTATGCAT
CAGTGCTTCCCACTTACCTGATTTGTCTGTCGGTGGCCCCATATGGAAACCCTGCGTGTCTGTTGGCATA
ATAGTTTACAAATGGTTTTTTCAGTCCTATCCAAATTTATTGAACCAACAAAAATAATTACTTCTGCCCT
GAGATAAGCAGATTAAGTTTGTTCATTCTCTGCTTTATTCTCTCCATGTGGCAACATTCTGTCAGCCTCT
TTCATAGTGTGCAAACATTTTATCATTCTAAATGGTGACTCTCTGCCCTTGGACCCATTTATTATTCACA
GATGGGGAGAACCTATCTGCATGGACCTCTGTGGACCACAGCGTACCTGCCCCTTTCTGCCCTCCTGCTC
CAGCCCCACTTCTGAAAGTATCAGCTACTGATCCAGCCACTGGATATTTTATATCCTCCCTTTTCCTTAA
GCACAATGTCAGACCAAATTGCTTGTTTCTTTTTCTTGGACTACTTTAATTTGGATCCTTTGGGTTTGGA
GAAAGGGAATGTGAAAGCTGTCATTACAGACAACAGGTTTCAGTGATGAGGAGGACAACACTGCCTTTCA
AACTTTTTACTGATCTCTTAGATTTTAAGAACTCTTGAATTGTGTGGTATCTAATAAAAGGGAAGGTAAG
ATGGATAATCACTTTCTCATTTGGGTTCTGAATTGGAGACTCAGTTTTTATGAGACACATCTTTTATGC**C**
ATGTATAGATCCTCCCCTGCTATTTTTGGTTTATTTTTATTGTTATAAATGCTTTCTTTCTTTGACTCCT
CTTCTGCCTGCCTTTGGGGATAGGTTTTTTTGTTTGTTTATTTGCTTCCTCTGTTTTGTTTTAAGCATCA
TTTTCTTATGTGAGGTGGGGAAGGGAAAGGTATGAGGGAAAGAGAGTCTGAGAATTAAAATATTTTAGTA
TAAGCAATTGGCTGTGATGCTCAAATCCATTGCATCCTCTTATTGAATTTGCCAATTTGTAATTTTTGCA
TAATAAAGAACCAAAGGTGTAATGTTTTGTTGAGAGGTGGTTTAGGGATTTTGGCCCTAACCAATACATT
GAATGTATGATGACTATTTGGGAGGACACATTTATGTACCCAGAGGCCCCCACTAATAAGTGGTACTATG
GTTACTTCCTTGTGTACATTTCTCTTAAAAGTGATATTATATCTGTTTGTATGAGAAACCCAGTAACCAA
TAAAATGACCGCATATTCCTGACTAAACGTAGTAAGGAAAATGCACACTTTGTTTTTACTTTTCCGTTTC
ATTCTAAAGGTAGTTAAGATGAAATTTATATGAAAGCATTTTTATCACAAAATAAAAAAGGTTTGCCAAG
CTCAAAAAAAAAAAAAAAAA

28) Entrez Gene ID 4928 = NUP98, nucleoporin 98kDa = AB040538

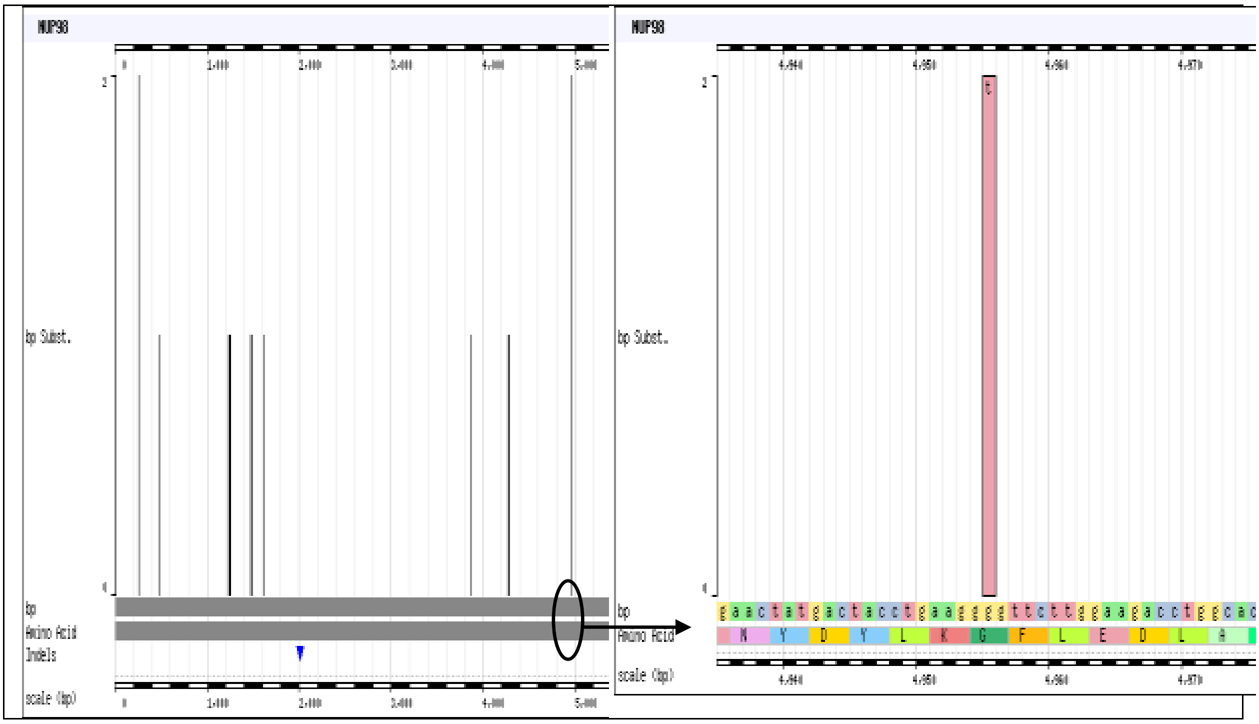

>gi|11414895|dbj|AB040538.1| Homo sapiens NUP98 mRNA for nucleoporin, complete cds

GGCCCTCTGCGCGCTGCGCCCGAAGCGGCGGTCGGTGGCAGGGGTGGTAGCGGCGGCGGCGACGGTTTCG
TGGGGGCCGCGCGCTGCTCTGTGAGCGGCGGGTGGCAGCAGGGGACTCCTGACACTTCCCCTTCCCCACC
GAACCGCGCTTTCTGAAACAAAGACTCATTTTGAAGATGTTTAACAAATCATTTGGAACACCCTTTGGGG
GTGGCACAGGTGGCTTTGGCACAACTTCAACATTTGGACAGAATACTGGCTTTGGCACTACTAGTGGAGG
GGCATTTGGAACATCTGCATTTGGTTCTAGCAACAATACTGGAGGCCTCTTTGGAAATTCACAGACTAAA
CCAGGAGGATTGTTTGGAACCAGTTCATTTAGCCAGCCAGCTACCTCCACAAGCACTGGCTTTGGGTTTG
GTACGTCAACAGGAACAGCAAATACCTTGTTTGGAACTGCAAGCACAGGGACCAGTCTCTTCTCATCCCA
AAACAATGCCTTTGCACAAAATAAACCAACTGGCTTTGGCAATTTTGGAACCAGTACTAGCAGTGGAGGA
CTCTTTGGAACCACAAATACCACCTCTAATCCTTTTGGCAGCACATCTGGCTCCCTCTTTGGGCCAAGTA
GTTTTACAGCTGCTCCTACTGGGACTACTATTAAATTTAACCCTCCAACTGGTACAGATACTATGGTCAA
AGCTGGAGTTAGCACTAACATAAGTACCAAGCACCAGTGTATTACTGCTATGAAAGAATATGAAAGCAAG
TCACTAGAGGAACTTCGTTTAGAGGATTATCAGGCTAACAGGAAGGGCCCACAGAACCAGGTGGGAGCAG
GTACCACAACTGGCTTGTTTGGGTCTTCTCCAGCCACTTCCAGCGCAACAGGACTCTTCAGCTCCTCCAC
CACTAATTCAGGCTTTGCATATGGTCAGAACAAAACTGCCTTTGGAACTAGTACAACTGGATTTGGAACA
AATCCAGGTGGTCTCTTTGGCCAACAGAATCAGCAGACTACCAGCCTCTTCAGCAAACCATTTGGCCAGG
CTACAACCACCCAGAACACTGGCTTTTCCTTTGGTAATACCAGCACCATAGGACAGCCAAGCACCAACAC
CATGGGATTATTTGGAGTAACCCAAGCCTCACAGCCTGGAGGTCTTTTTGGGACAGCTACAAACACCAGC
ACTGGGACAGCATTTGGAACAGGAACAGGTCTCTTTGGGCAGACCAATACTGGATTTGGTGCTGTTGGTT
CGACCCTGTTTGGCAATAACAAGCTTACTACATTTGGAAGCAGCACAACCAGTGCACCTTCATTTGGTAC
AACCAGTGGCGGGCTCTTTGGTAACAAACCAACCCTGACTTTAGGAACCAATACAAACACTTCTAATTTT
GGTTTTGGCACAAATACCAGTGGGAATAGTATTTTTGGAAGTAAACCAGCACCTGGGACTCTTGGAACTG
GGCTTGGTGCAGGATTTGGAACAGCTCTTGGTGCTGGACAGGCATCTTTGTTTGGGAACAACCAACCTAA
GATTGGAGGGCCTCTTGGTACAGGAGCCTTTGGGGCCCCTGGATTTAATACTACGACAGCCACTTTGGGC
TTTGGAGCCCCCCAGGCCCCAGTAGCTTTGACAGATCCAAATGCTTCTGCTGCCCAGCAGGCTGTTCTCC
AGCAGCACATCAATAGTCTAACATACTCACCTTTTGGAGACTCTCCTCTCTTCCGGAATCCGATGTCAGA
CCCTAAGAAGAAGGAAGAGAGATTGAAACCAACAAATCCAGCAGCCCAGAAGGCTCTTACTACACCTACT
CATTATAAACTGACACCCCGCCCTGCCACTAGAGTCCGGCCAAAGGCTTTACAAACAACAGGCACAGCCA
AGTCACATCTCTTTGATGGGCTGGATGACGATGAACCATCCCTAGCCAATGGAGCATTCATGCCCAAGAA
GAGCATTAAGAAGTTGGTTTTGAAGAACCTTAATAATAGCAATCTCTTTTCTCCTGTTAATCGTGATTCA
GAAAATCTAGCTTCACCATCTGAATATCCAGAAAATGGAGAGAGATTTAGTTTCCTAAGCAAACCTGTTG
ATGAGAATCACCAGCAGGATGGAGATGAAGATTCCCTTGTTTCACATTTTTATACTAACCCTATTGCCAA
ACCTATTCCTCAAACCCCAGAAAGTGCTGGAAATAAACACAGCAACAGCAACAGTGTGGATGATACCATT
GTTGCATTAAACATGCGTGCTGCTTTGCGAAATGGGCTGGAAGGAAGCAGTGAAGAAACGTCTTTTCATG
ATGAGTCACTTCAGGATGACCGAGAAGAAATAGAAAATAATTCTTACCATATGCACCCAGCAGGTATTAT
TCTCACTAAGGTTGGTTACTATACTATTCCATCTATGGATGACCTTGCTAAAATTACCAATGAAAAAGGA

GAGTGCATTGTCTCTGATTTCACTATTGGTCGGAAAGGTTATGGTTCAATCTATTTTGAAGGAGATGTGA
ATTTGACAAATCTAAATTTGGATGATATTGTGCATATCCGGAGGAAAGAAGTAGTTGTCTACTTAGATGA
TAACCAAAAACCACCTGTGGGTGAAGGGCTAAATAGGAAGGCTGAAGTTACATTGGATGGAGTTTGGCCA
ACAGATAAAACATCTCGTTGTTTAATAAAGAGCCCAGATCGCCTTGCTGATATCAACTATGAAGGAAGAT
TGGAAGCAGTTTCAAGGAAACAGGGAGCTCAATTCAAAGAATACCGGCCTGAAACTGGTTCTTGGGTGTT
TAAGGTCTCCCATTTTTCTAAGTATGGCCTTCAGGATTCTGATGAAGAGGAGGAGGAGCATCCGTCTAAA
ACTAGTACAAAGAAGTTGAAGACTGCTCCTTTGCCTCCTGCAAGCCAGACTACGCCCTTGCAGATGGCTC
TTAATGGCAAACCTGCACCTCCACCTCAGGTAGAGAAAAAAGGACAGTGAATTTGAATGGAATCCGTGAT
ACCGAAGTTGAAAGCAAGTCATTCAGCTAATACAAAGCTGTTTTATGACCCTTGGAACTTTGAAGAGTAC
AAACATTGGCAATCACGTTGAAACAAGTGCAAGGGAGGGCGTGAGGTCTTGCAGGCATCTGTCTTTTTAC
TGGAGAGATTTAAAGAATTCTCTTGCTGTTTGGATTATTCCTCTACAGATTGTCATTTTTAAACCCTTTG
TTCTCTCTCATTTGGACTTGCTGAATTCTCTGCTCAGTGATTAACTTAAGATTTGCTCATGTGGGTTCAT
GCACAGTAAATTCTGCCTTTATTGACTACCTGATGTGCAGTTTAATCTTTTTCTTTACCTCCATGGTTTT
TTAAAAGTTAAATTAGCTTTCTGAAAGGGTTTTTAATCTCCATTTTTTTAAAGTTGTTTGCTTATACTTC
GGGTAACCTTGATATTTGTATTTTAATAGTACATAATCTTTATGAAAAATAGTTTGGGAATGTAAATGAA
TTATTATTTGGCTTGGGGAGATTAGGGCCTACATTGTTTATCGCAATTACTTGTATCATTGATACGGGAT
TTCTTTGTAAAGCATCCTCTACCTCTCAGCTGCTGAAAGCTAGACCTTTGGTATTTTC**CATGCTATAATT**
CTTATGGCTGCTGAAATGTGTGGTTTTTATGATTTATTAAATAATCTCTTAGGAGGCAAAAAAAAAAAAA
AAAAAAAAAAAAAAAAAAAAAA

29) Entrez Gene ID 5049 = PAFAH1B2, platelet-activating factor acetylhydrolase 1b, catalytic subunit 2 (30kDa) = DQ836743

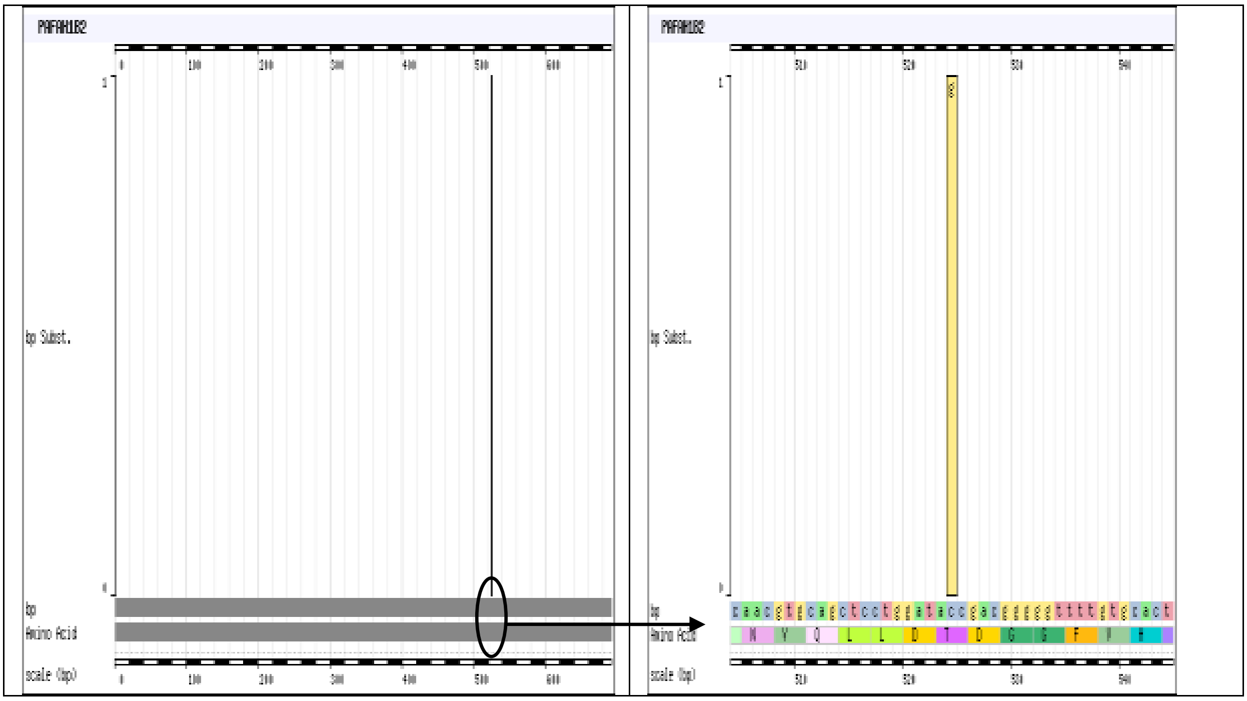

>gi|114228436|gb|DQ836743.1| Homo sapiens intracellular platelet-activating factor acetylhydrolase alpha 2 subunit (PAFAH1B2) mRNA, complete cds, alternatively spliced
GGGACCGAGCGAGCGACCGACGCGCCACCCGCCGACGCCTCAGCCGCTTGGGGCCCGCACGGACCCTCTA
CTTCAGTGTAGAATGAGCCAAGGAGACTCAAACCCAGCAGCTATTCCGCATGCAGCAGAAGATATTCAAG
GAGATGACCGATGGATGTCTCAGCACAACAGATTTGTTTTGGACTGTAAAGACAAAGAGCCTGATGTACT
GTTCGTGGGAGACTCCATGGTGCAGTTAATGCAGCAATATGAGATATGGCGAGAGCTTTTTTCCCCACTT
CATGCACTGAATTTTGGAATTGGGGGAGATACAACAAGACATGTTTTGTGGAGACTAAAGAATGGAGAAC
TGGAGAATATTAAGCCTAAGGTCATTGTTGTCTGGGTAGGAACAAATAACCACGAAAATACAGCAGAAGA
AGTAGCAGGTGGGATCGAGGCCATTGTACAACTTATCAACACAAGGCAGCCACAGGCCAAAATCATTGTA

TTGGGTTTGTTACCTCGAGGTGAGAAACCCAATCCTTTGAGGCAAAAGAACGCCAAGGTGAACCAACTCC
TCAAGGTTTCGCTGCCGAAGCTTGCCAACGTGCAGCTCCTGGATA**C**CGACGGGGGTTTTGTGCACTCGGA
CGGTGCCATCTCCTGCCACGACATGTTTGATTTTCTGCATCTGACAGGAGGGGGCTATGCAAAGATCTGC
AAACCCCTGCATGAACTGATCATGCAGTTGTTGGAGGAAACACCTGAGGAGAAACAAACCACCATTGCCT
GACTGGCTCTTATCAGTGTTAATAGCATCTCAGCTTCCTCAGATCAGTTCTATCACTGGCACTACAGAAT
CCTTCTCTTTCTTAAGGCACTTTGCATTGTAGAATGTTCCTGGATGTTCATATCTAGTGTTTGAAGGGGA
GGAGGGATTTAAACTGGTCCTGTACATAGAAGGTTTGTTTGACAGAGGAGAAAAATTAGCCAAGGAAGAT
TGTTGTTTAAATTCATTTGAAACCAGAAGGGGACTTTTTAGTTGTATGTGTAACACATTCATTGAATTAT
TATCACTGTTTTCTTGGGACAACATCAAGCCTAAATACTGAACAATATGAAGATTCTTTTCTTGGCCTTT
CTGTGGATTATGTCATATATAATAATTATCAGAATCATTCTACTTGGCTTTAAAACATGTTTTCTCCAAT
TTTTTTAAGGTTCATAATTTAGCCTTTTGTTTTTATGTTGCTTAGATTCTTATGTATACTGAATATTTTA
TTAACATGTAGCATCAGGTTGAACATGCTTGTCATTGATATATGGAAGATGCTATAGTTAGAAGTGAATT
TGTTCTGCTTTCTTAATCTTTTCCATGCTTAGCAGTGAAAAACAGGTTTTGCCCCAGTAGAGGGATTCTT
TGGAGGGTATTATTTTTTATGCTGCTGAATATCATGTCTATAATAGACCCGTGCATGCAGCCTTTCCTCC
TTATTCCCCTTCATGCCCCCCTTTCCCCTTCATTCCCCCCGCCACCCCGTTTGTTGTTTTTTTTTTTTTT
TTTTGGTTCTTGTTGACATTACAAGCTTTTAACACATTTTTGACCTAGGAGCCCTGTTGCTGGAAGTATA
GTCTCCAGCCAGTTACTCTCATGATAGTACTGCTATAAAACTCATTCTTGTGTGGTGTTCTGTGCTATAG
AGTCTGTGTATTGCTGTTCATATTCGGAGTTCTGGTTTTGTTTTTCCCTTAAAACCTGTTAACAGTTTTT
TTTGGGGGTGGGGGGATTCAGAACTCTTGTTTCCCATTCCATAGCACCTGACATTATTTCAAGTTTTATA
ATATCTTAAGGTGTATATTTTATTTTTTTTTATTGGCTTAGTTGTTTTTTGTTTTGTTTTGTTTGAGATGG
AGTTTCACTGTTGCCCAGGCTGGGGTGCAATGGTGTGATCTTGGCTCACTGCAGCCTCCACCTCCCGAGT
TCAAATGATTCTCCTGCCTCAGCTTCCTGAGTAGCTGGGATTACAGGTGCATGCCACCATGCCCGGCTAA
TTTTTATATTTTTATTAGAGACGGGATTTCGCCATGTTAACCAGGCTGGTCTCGAACTCCTGACCTCAGG
TGATCTGCCCGCCTTGGCCTCCCAAAGTGCTGGGATTACAGGCATGAGCCACCATGCCTGGCCTTATTGG
CTTAGTTTTTAAATTATCCTCCAAAAATTTTGGGCCTTTTTCTGTGGGGAAACAAGTGAAGCTGCTCTTC
AGCATAGACACTACCTTTATCCCATCATTTTAGTAAAAACTAGGTTTGTTTCACTTCTGAGGTGTCTTAT
TAATGTACTTCATCTGAGAATTTGTTGATCTTAATGTTCGAGCTATATAAGAACTGCCATTAAAAAAAAT
GGGATAATAGATGATTTTATCAGTATACCTGTGGAATATGTACAAACTGGATCTATAGATATTTTGAACT
GGACCAGGTGGGTATTGAAGTAACCCATCAAAATATGCTCTGCAGTGATTCCGCTTAATGTTTAAATTCA
GTAACGTACTTGAAAGGCAAATTTCAGTGCTTTTGTTATGTTGGAGGAGGGCTTACTGATGCGTGCTAAG
ACCGATTTCTGATTGAGGGATGAACCTTGGGCTCATTTTTTTCTTGTGAAGTCTCTTTCTAGAAAATTTT
TTGGTTTTGTTCTTTTTAAAAAATACATACTTTTTTGAATGTATCATGTCTTCATTAACAACAGAAAATC
CACATGGTGTTTACTAAACTTGTTTACGACATTAAAAATTTCCTTTTTATTTTTAGTAGCCCAGGTTGAG
TTTTTCACAAGAGATTTTTTTCTTAGCTGAGGTATAGTTGTATAGCAAGAAGAATTAAGCCAGATTTTTG
TGTGTGGAAAGACAGTTTTCTATCCACGTCTTTTTCTGTTTGTCAGAAGGTGGGAGTATGGTCCAAATAA
ATCCATTAGGTTACTCCTGCAGCATGCGCTTTTAGCTTCTCTCTTGACTGAGGATCAAATATCCCTTTGT
GAGCTGGCCCTCAGCTCCTTTGCTCATGTGTACAAACCTCAGATGTTACTACATTTTATATCTACCAGAG
CTATTCAAGCAATAGTATTTGAACCACTAGCCTTTTAAATAAAATTCTGCCCCATTACTGATGTGCAGAT
ATTGAGTTCACTTTCATTTTTTGCCAGATTTCTTTGCACTACTTTAGGTAAAAATAGTTAATCTATTTTT
CTTTGACATCCTAGTTTGCGTCAGTGACAGAACTTACTGCTTAGTCTTTGTACTTTTTAAAAAATCTATA
AATTTAATGCACTGTCCAAGTGAAATGTCCTAGTTGTCATTGTGATTAAGGGGCCAACTTTCCAGGCAGC
TAGCAGAGATACTATTCTCTTCCTCTCCCAGCAAATTTGTATTCCTTCGCCCACGCATTCCTGCTATACT
AGATGGCAGCCAGTGATGGAACTATAAAGATGTCTGTGGTCATATGTTGAATGTGGCAGCTTGAAGATGT
ACTGCCACGGGTGATCTAGGGCAGGCTGTCTTCCAGTCCATGTGTTCTCGGTCGCCGTAGACAGCGCTCT
GGCTACCACCGTGAGGCTACTTGAACTGTCAGGGGCATCTGCCTAAACCAGAATCTTTTGTCAGAAACCT
TAACCCAACAAAACAAATCTTGAGTAGCTCATGCCCGGCTCTTAGGAATTTTGTCTGTTTAAAAAAAAAA
AAAAAAAAAAAGTCCAACTTACTTTATTTTATTTTTTTAACCTAGTCACTGTTTACAATTGTATGCTAAA
GCCTGAAATATTGTCTGTGCTGTGGTGTATGAGCATTGCCAACTTTATATTTATTGCAGTGAAGAAGAAA
CTAAAAATATATGGAAATGAGGAGCATGTCCAAGCTCCTAAATCCGTGTGGGTG**CATGTGGAGAAGTGA**
GTTAGGGCCTCTTGAAAGGAGGCTTTTTGGAGAGGGGTCCCCCAGGTTTCTTGGTGTTCCTGCTTGGGGA
TCACTGCTGCTAGCTGACTGGACCTCCCCATTGGAAGTTTGTGATTTTGCTTTGGCAAAGTTTCATTGAC
TAGTAGAACTCATTCTGTTTTAGTGTATATTTCAATATAAATGTAAACATTTTGCTCAAAAAAAAAAAAA
AAAAAAAAAAA

30) Entrez Gene ID 5518 = PPP2R1A, protein phosphatase 2, regulatory subunit A, alpha = BC001537

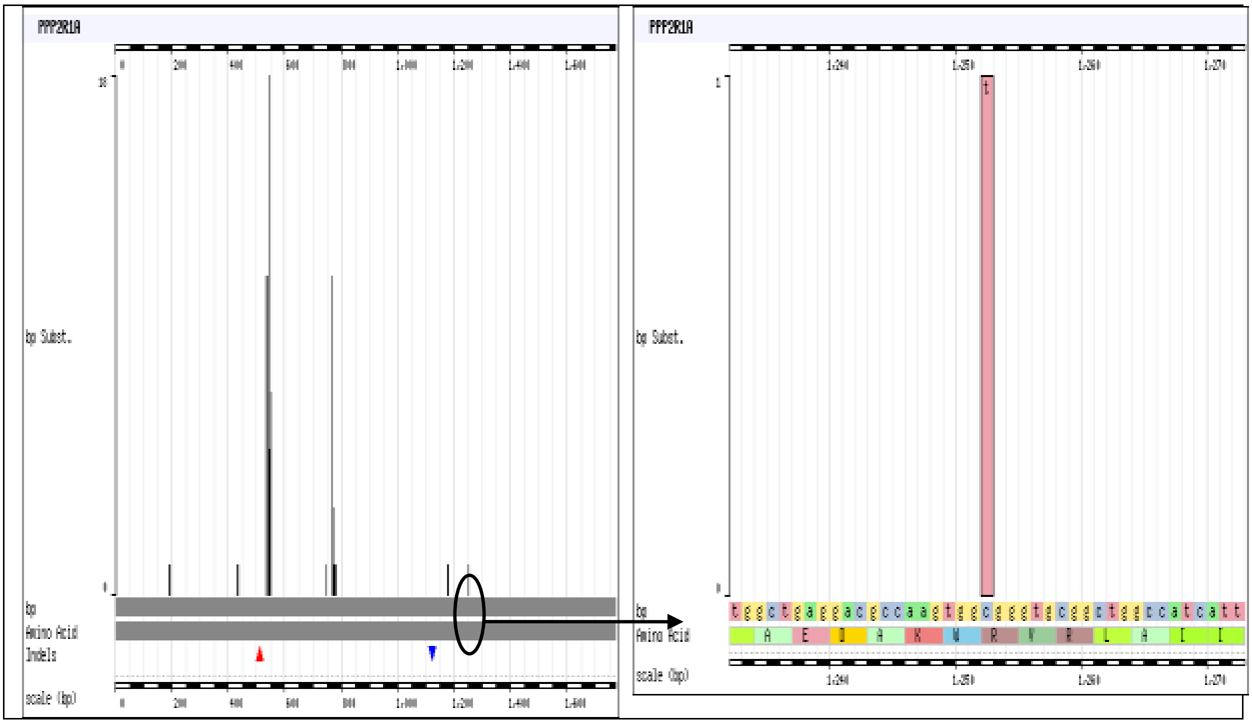

>gi|34783905|gb|BC001537.2| Homo sapiens protein phosphatase 2 (formerly 2A), regulatory subunit A, alpha isoform, mRNA (cDNA clone MGC:786 IMAGE:2987938), complete cds

CAGCGCTGGCCGCAGTCTGACAGGAAAGGGACGGAGCCAAGATGGCGGCGGCCGACGGCGACGACTCGCT
GTACCCCATCGCGGTGCTCATAGACGAACTCCGCAATGAGGACGTTCAGCTTCGCCTCAACAGCATCAAG
AAGCTGTCCACCATCGCCTTGGCCCTTGGGGTTGAAAGGACCCGAAGTGAGCTTCTGCCTTTCCTTACAG
ATACCATCTATGATGAAGATGAGGTCCTCCTGGCCCTGGCAGAACAGCTGGGAACCTTCACTACCCTGGT
GGGAGGCCCAGAGTACGTGCACTGCCTGCTGCCACCGCTGGAGTCGCTGGCCACAGTGGAGGAGACAGTG
GTGCGGGACAAGGCAGTGGAGTCCTTACGGGCCATCTCACACGAGCACTCGCCCTCTGACCTGGAGGCGC
ACTTTGTGCCGCTAGTGAAGCGGCTGGCGGGCGGCGACTGGTTCACCTCCCGCACCTCGGCCTGCGGCCT
CTTCTCCGTCTGCTACCCCCGAGTGTCCAGTGCTGTGAAGGCGGAACTTCGACAGTACTTCCGGAACCTG
TGCTCAGATGACACCCCCATGGTGCGGCGGGCCGCAGCCTCCAAGCTGGGGGAGTTTGCCAAGGTGCTGG
AGCTGGACAACGTCAAGAGTGAGATCATCCCCATGTTCTCCAACCTGGCCTCTGACGAGCAGGACTCGGT
GCGGCTGCTGGCGGTGGAGGCGTGCGTGAACATCGCCCAGCTTCTGCCCCAGGAGGATCTGGAGGCCCTG
GTGATGCCCACTCTGCGCCAGGCCGCTGAAGACAAGTCCTGGCGCGTCCGCTACATGGTGGCTGACAAGT
TCACAGAGCTCCAGAAAGCAGTGGGGCCTGAGATCACCAAGACAGACCTGGTCCCTGCCTTCCAGAACCT
GATGAAAGACTGTGAGGCCGAGGTGAGGGCCGCAGCCTCCCACAAGGTCAAAGAGTTCTGTGAAAACCTC
TCAGCTGACTGTCGGGAGAATGTGATCATGTCCCAGATCTTGCCCTGCATCAAGGAGCTGGTGTCCGATG
CCAACCAACATGTCAAGTCTGCCCTGGCCTCAGTCATCATGGGTCTCTCTCCCATCTTGGGCAAAGACAA
CACCATCGAGCACCTCTTGCCCCTCTTCCTGGCTCAGCTGAAGGATGAGTGCCCTGAGGTACGGCTGAAC
ATCATCTCTAACCTGGACTGTGTGAACGAGGTGATTGGCATCCGGCAGCTGTCCCAGTCCCTGCTCCCTG
CCATTGTGGAGCTGGCTGAGGACGCCAAGTGG**C**GGGTGCGGCTGGCCATCATTGAGTACATGCCCCTCCT
GGCTGGACAGCTGGGAGTGGAGTTCTTTGATGAGAAACTTAACTCCTTGTGCATGGCCTGGCTTGTGGAT
CATGTATATGCCATCCGCGAGGCAGCCACCAGCAACCTGAAGAAGCTAGTGGAAAAGTTTGGGAAGGAGT
GGGCCCATGCCACAATCATCCCCAAGGTCTTGGCCATGTCCGGAGACCCCAACTACCTGCACCGCATGAC
TACGCTCTTCTGCATCAATGTGCTGTCTGAGGTCTGTGGGCAGGACATCACCACCAAGCACATGCTACCC
ACGGTTCTGCGCATGGCTGGGGACCCGGTTGCCAATGTCCGCTTCAATGTGGCCAAGTCTCTGCAGAAGA
TAGGGCCCATCCTGGACAACAGCACCTTGCAGAGTGAAGTCAAGCCCATCCTAGAGAAGCTGACCCAGGA
CCAGGATGTGGACGTCAAATACTTTGCCCAGGAGGCTCTGACTGTTCTGTCTCTCGCCTGATGCTGGAAG
AGGAGCAAACACTGGCCTCTGGTGTCCACCCTCCAACCCCCACAAGTCCCTCTTTGGGGAGACACTGGGG
GGCCTTTGGCTGTCACTCCCTGTG**CATGGTCTGACCCCAGGCCCC**TTCCCCCAGCACGGTTCCTCCTCTC
CCCAGCCTGGGAAGATGTCTCACTGTCCACCTCCCAACGGGCTAGGGGAGCACGGGGTTGGACAGGACAG
TGACCTTGGGAGGAAGGGGCTACTCCGCCCACGTCAGGGAGAGATGTGAGCATCCCGGGTCACTGGATCC
TGCTGCTGTAATGGGAACCCCTCCCCCATTTACTTCTCCACCTCCCGTCCTCCCCATCATTGGTTTTTTT
TTGTGTGTCAACTGTGCCGTTTTTATTTTATTCCTTTTATTTTCCCCCTTTTCACAGAGAAATAAAGGTC
TAGAAGTAAAAAAAAAAAAAAAAAAAAAAAAAAAAAAAAAA

31) Entrez Gene ID 5546 = PRCC, papillary renal cell carcinoma (translocation-associated) = BC004913

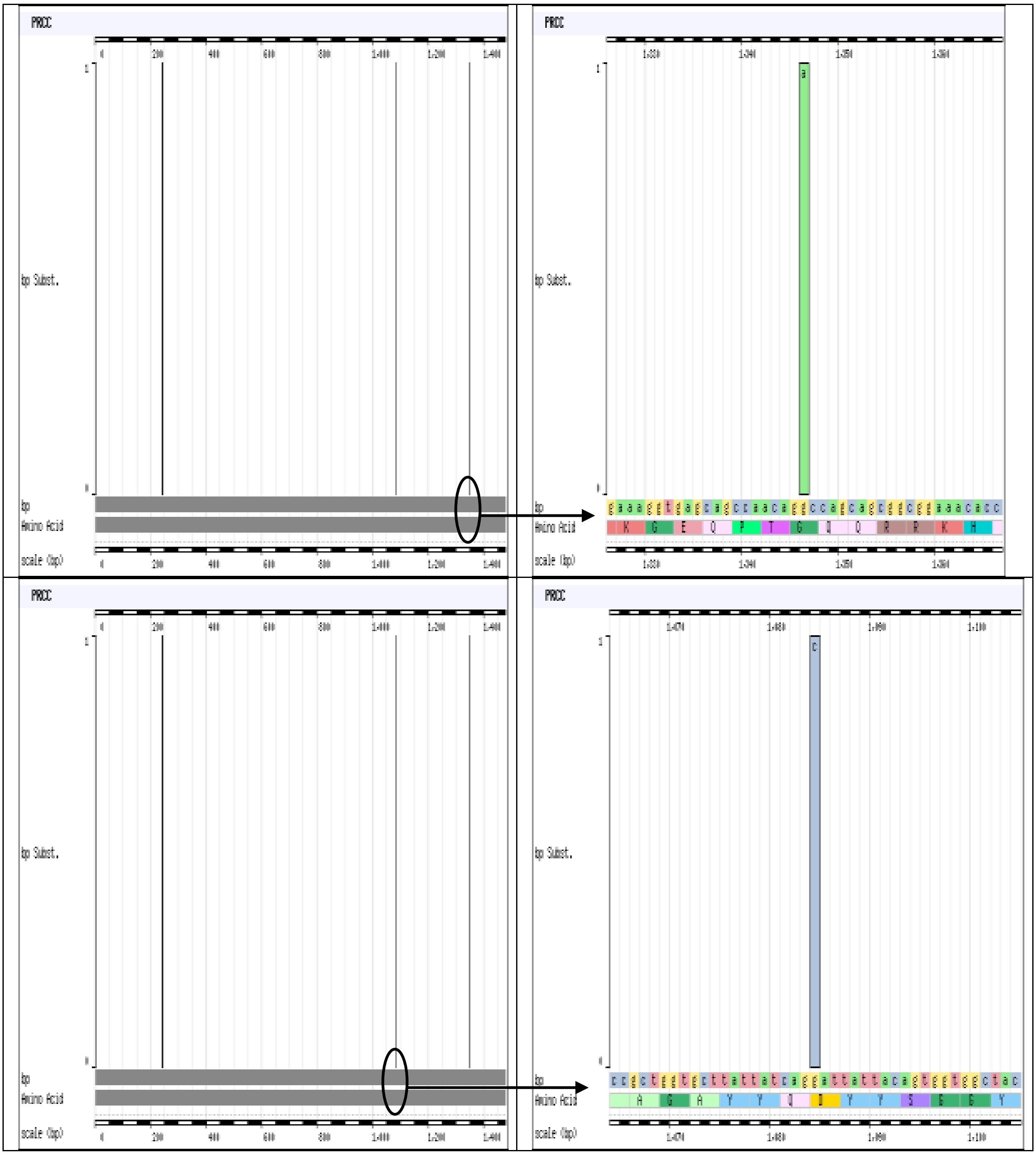

>gi|33873117|gb|BC004913.2| Homo sapiens papillary renal cell carcinoma (translocation-associated), mRNA (cDNA clone MGC:4723 IMAGE:3535413), complete cds
AGGAGCTTAAGTGAAGAGGTACGCCTTGTTCGGTGGAAATCAGCCGTAGCCATGAGTTTCTGCCGGGGCT
AGCCCTAGAGTACGGAGCAGGCGGACTTTTCGGTTCCCCGCCCCGCCAGGTGGCGGGGCCTACTAGGCCT
CCGGGCATCCCCGGTCTCAAGTAGGCCTCATCTGCCGGCAAGGGCGCCCGAAACGCGGGAGGCGCCATGT
CGCTGGTTGCTTACGCCAGCAGCGATGAGAGCGAGCCGGATGAGGCTGAGCCCGAGCCGGAGGAAGAGGA
GGCGGTGGCTCCTACATCTGGGCCCGCTTTAGGGGGCTTGTTCGCTTCTCTCCCTGCGCCCAAGGGTCCG

GCCTTGCTGCCTCCGCCCCCTCAGATGCTGGCGCCAGCCTTTCCCCCGCCGCTGTTGCTTCCCCCACCCA
CCGGAGACCCCAGGCTTCAGCCTCCTCCCCCCTTGCCCTTCGGCCTGGGAGGCTTCCCCCCACCTCCAGG
CGTGAGCCCGGCTGAAGCGGCGGGAGTTGGGGAGGGACTGGGATTGGGGTTGCCCTCGCCCCGAGGCCCT
GGCCTCAATCTGCCCCCTCCAATTGGCGGTGCCGGTCCCCCGCTGGGGCTTCCCAAGCCAAAGAAGAGGA
AAGAGCCCGTGAAGATCGCGGCGCCGGAGTTGCATAAGGGAGATTCAGATTCTGAGGAAGATGAACCCAC
AAAGAAGAAAACTATCCTTCAGGGATCCAGTGAGGGGACTGGTTTGTCTGCCTTGCTTCCCCAACCTAAA
AACCTGACTGTGAAAGAGACTAACAGGTTGCTCCTGCCCCATGCCTTCTCCCGCAAACCCTCGGATGGCT
CCCCTGATACTAAGCCCTCCAGACTGGCTTCTAAGACCAAGACTTCCTCTCTTGCCCCTGTTGTGGGCAC
CACAACCACCACTCCGTCGCCCTCTGCTATCAAGGCTGCTGCCAAGAGTGCTGCCCTGCAGGTGACAAAG
CAGATCACGCAGGAAGAAGACGACAGTGATGAGGAAGTAGCCCCCGAAAACTTTTTCTCCCTCCCTGAAA
AGGCTGAGCCACCTGGAGTTGAGCCATACCCTTACCCCATCCCCACTGTCCCTGAAGAGCTGCCTCCAGG
CACGGAACCAGAGCCGGCTTTCCAGGACGATGCAGCCAATGCCCCCCTTGAATTCAAGATGGCAGCAGGT
TCAAGTGGGGCCCCTTGGATGCCTAAGCCTGGGGACGACTACAGCTACAATCAGTTTTCCACATATGGCG
ATGCCAATGCCGCTGGTGCTTATTATCAGGATTATTACAGTGGTGGCTACTATCCTGCACAGGACCCGGC
CCTGGTCCCCCCCCAGGAAATTGCCCCAGATGCCTCCTTCATCGATGACGAAGCATTTAAGCGGCTGCAG
GGCAAGAGGAACCGAGGGAGAGAAGAAATCAACTTTGTGGAGATCAAAGGTGATGACCAGCTCAGTGGGG
CCCAGCAATGGATGACTAAGTCATTGACAGAAGAGAAAACCATGAAGTCATTCAGCAAAAAGAAAGGTGA
GCAGCCAACAGGCCAGCAGCGGCGGAAACACCAGATCACATATCTTATTCATCAGGCCAAGGAGCGGGAG
CTGGAACTGAAGAACACCTGGTCAGAGAACAAGCTCAGCCGCCGTCAGACCCAAGCCAAATATGGATTCT
AGGGCTCTGGAACTGATTGCTCCCAGGATCTCCTGCCAGCCCAGCTGGCCTGGCCCCCAGCTTCACCTCT
GGGACCCCAGCTGCTCTAAGCCCAGGATCTCTTTCCCCAAGGACCCAGCCCTCGCCTCTGCGAGAATGAA
CATATTTGATAGATTTTTCTTAACAAGTTAGAAAATTCAGCTCCTTTCTGTCCTGGAGCTAGCAAAGACT
TGTGTGATGCCTCCGAAGGGGCTCTGAGTTCTGGGGTGGGAGTTTTGCTCTCTGTCAGGTGTGATAAAAT
GTTGAACCCTCCCCACCACCACTTTTTTTTTTTTAAACCAGGGATGTCTGTTGAAATAAAACATTCAGTC
TGACAAAAAAAAAAAAAAAAAAAAAAAAAAAAAAAA

32) Entrez Gene ID 5573 = PRKAR1A, protein kinase, cAMP-dependent, regulatory, type I, alpha (tissue specific extinguisher 1) = BC036285

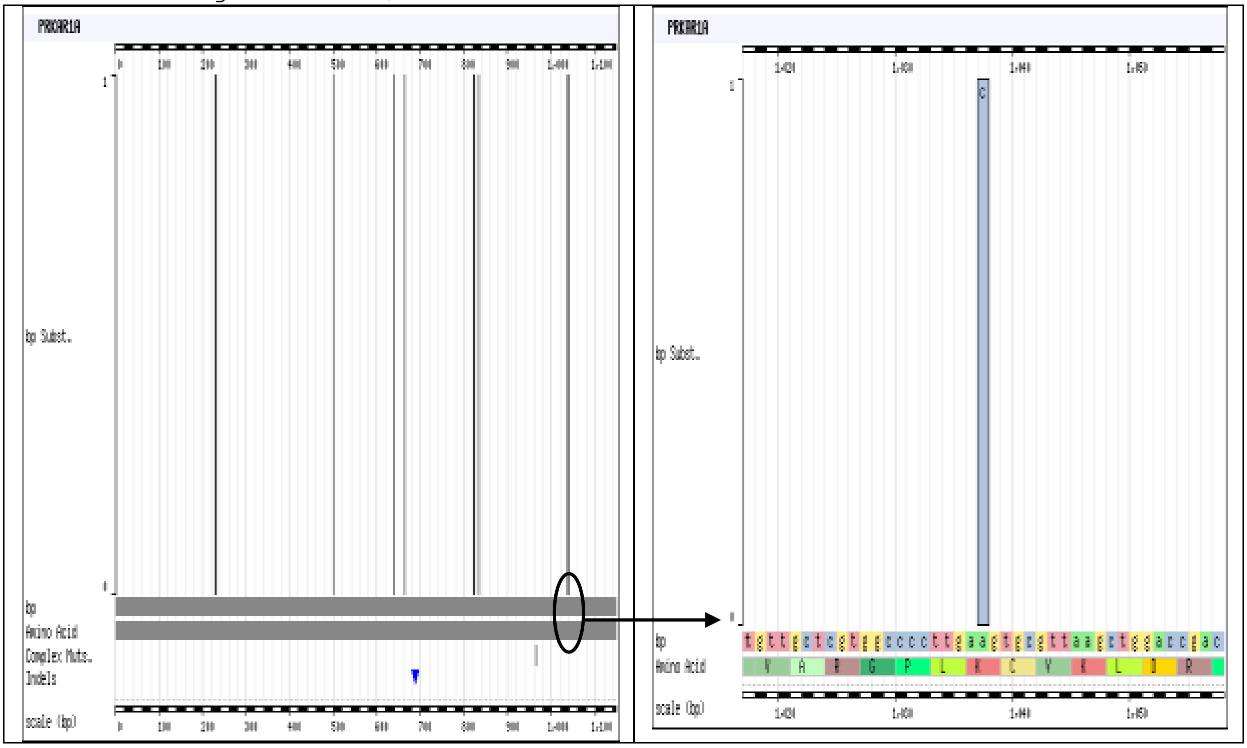

>gi|23273779|gb|BC036285.1| Homo sapiens protein kinase, cAMP-dependent, regulatory, type I, alpha (tissue specific extinguisher 1), mRNA (cDNA clone MGC:17251 IMAGE:4340015), complete cds
GGTGGAGCTGTCGCCTAGCCGCTATCGCAGAGTGGAGCGGGGCTGGGAGCAAAGCGCTGAGGGAGCTCGG
TACGCCGCCGCCTCGCACCCGCAGCCTCGCGCCCGCCGCCGCCCGTCCCCAGAGAACCATGGAGTCTGGC
AGTACCGCCGCCAGTGAGGAGGCACGCAGCCTTCGAGAATGTGAGCTCTACGTCCAGAAGCATAACATTC

AAGCGCTGCTCAAAGATTCTATTGTGCAGTTGTGCACTGCTCGACCTGAGAGACCCATGGCATTCCTCAG
GGAATACTTTGAGAGGTTGGAGAAGGAGGAGGCAAAACAGATTCAGAATCTGCAGAAAGCAGGCACTCGT
ACAGACTCAAGGGAGGATGAGATTTCTCCTCCTCCACCCAACCCAGTGGTTAAAGGTAGGAGGCGACGAG
GTGCTATCAGCGCTGAGGTCTACACGGAGGAAGATGCGGCATCCTATGTTAGAAAGGTTATACCAAAAGA
TTACAAGACAATGGCCGCTTTAGCCAAAGCCATTGAAAAGAATGTGCTGTTTTCACATCTTGATGATAAT
GAGAGAAGTGATATTTTTGATGCCATGTTTTCGGTCTCCTTTATCGCAGGAGAGACTGTGATTCAGCAAG
GTGATGAAGGGGATAACTTCTATGTGATTGATCAAGGAGAGACGGATGTCTATGTTAACAATGAATGGGC
AACCAGTGTTGGGGAAGGAGGGAGCTTTGGAGAACTTGCTTTGATTTATGGAACACCGAGAGCAGCCACT
GTCAAAGCAAAGACAAATGTGAAATTGTGGGGCATCGACCGAGACAGCTATAGAAGAATCCTCATGGGAA
GCACACTGAGAAAGCGGAAGATGTATGAGGAATTCCTTAGTAAAGTCTCTATTTTAGAGTCTCTGGACAA
GTGGGAACGTCTTACGGTAGCTGATGCATTGGAACCAGTGCAGTTTGAAGATGGGCAGAAGATTGTGGTG
CAGGGAGAACCAGGGGATGAGTTCTTCATTATTTTAGAGGGGTCAGCTGCTGTGCTACAACGTCGGTCAG
AAAATGAAGAGTTTGTTGAAGTGGGAAGATTGGGGCCTTCTGATTATTTTGGTGAAATTGCACTACTGAT
GAATCGTCCTCGTGCTGCCACAGTTGTTGCTCGTGGCCCCTTGA**A**GTGCGTTAAGCTGGACCGACCTAGA
TTTGAACGTGTTCTTGGCCCATGCTCAGACATCCTCAAACGAAACATCCAGCAGTACAACAGTTTTGTGT
CACTGTCTGTCTGAAATCTGCCTCCTGTGCCTCCCTTTTCTCCTCTCCCCAATCCATGCTTCACTCATGC
AAACTGCTTTATTTTCCCTACTTGCAGCGCCAAGTGGCCACTGGCATCGCAGCTTCCTGTCTGTTTATAT
ATTGAAAGTTGCTTTTATTGCACCATTTTCAATTTGGAGCATTAACTAAATGCTCATACACAGTTAAATA
AATAGAAAGAGTTCTATGGAGACTTTGCTGTTACTGCTTCTCTTTGTGCAGTGTTAGTATTCACCCTGGG
CAGTGAGTGCCATGCTTTTTGGTGAGGGCAGATCCCAGCACCTATTGAATTACCATAGAGTAATGATGTA
ACAGTGCAAGATTTTTTTTTAAGTGACATAATTGTCCAGTTATAAGCGTATTTAGACTGTGGCCATATA
TGCTGTATTTCTTTGTAGAATAAATGGTTTCTCATTAAACTCTAAAGATTAGGGAAAATGGATATAGAAA
ATCTTAGTATAGTAGAAAGACATCTGCCTGTAATTAAACTAGTTTAAGGGTGGAAAAATGCCCATTTTTG
CTAATTATCAATGGGATATGATTGGTTCAGTTTTTTTTTTCCAGAGTTGTTGTTTGCCAAGCTAATCTG
CCTGGTTTTATTTATATCTTGTTATTAATGTTTCTTCTCCAATTCTGAAATACTTTTGAGTATGGCTATC
TATACCTGCCTTTTAAGTTTGAAACTAACTCATAGATTGCAAATATTGGTTAGTATTTAACTACATCTGC
CTCGGCTCACAAATTCCGATTAGACCTTTATCCAGCTAGTGCCAAATAATTGATCAGATGCTGAATTGAG
AATAAGAATTTGAGGTCTACATTCTTGGTTGTTAATTTAGAGCGTTTGGTTAAAGTATGTCCTTCAGCTG
ACTCCAGTATAATCTCCTCTGCTCATTAAACTGATTCCAGGAGATTGGATTTGCTGTGACTAGATACAGA
TGGAGCAAATGTCCTAACAGAGAAATAGAGGTGATGCTGCTAAAGGGAGAAATGCCAGGCGGACAAAGTT
CAGTGTCGGGAATTTTCCCCGTGACATTCACTGGGGCATGAGATTTTGGAAGAAGTTTTTTACTTTGGTT
TAGTCTTTTTTTCCTTCCTTTTTATTCAGCTAGAATTTCTGGTGGGTTGATGGTAGGGTATAATGTGTCT
GTGTTGCTTCAAATTGGTCTGAAAGGCTATCCTGCGGAAAGTCCTGCTTTCCTATCTAGCATTTATTTCT
CTGGCAAACTTTTCTTTCTTTTCTTTTTTAAAGTAAACTTGTGTATTGAGTCTTAACTGTATTTCAGTAT
TTTCCAGCCTTATGTGTTACATTATTCCAATGATACCCAACAGTTTATTTTTATTATTTTTTTAAACAAA
ATTTCACAGTTCTGTAATGTAGGCACTTTTATTTTCATTGTGATTTATATATAAGGTAATGTAGGGTTAT
ATTTGGGAGTGACTGCAAGCATTTTTCCATCTGTGTGCAACTAACTGACTCTGTTATTGATCCCTTCTCC
TGCCCTTTCCCAGGTAATTTAAATTGGTCATGGTAGATTTTTTTCATAGATTTGAAAAACTTTTAGGTTG
TTACCAAGTATGAAGTATAAATCTGGGGAAGAGGTTTTATTTACATTTTAGGGTGGGTAAGAAAGCCACC
TTGTTACAAATTTTTTAATTTCCAAAATAATCTATATTAAATGAGGGTTTCTGATCTGTACTTTGTGTTT
AGCTACCTTTTTATATTTAAAAAATTAAAAATGAAAATTACGTTCTTACAAGCTTAAAGCTTGATTTGAT
CTTTGTTTAAATGCCAAAATGTACTTAAATGAGTTACTTAGAATGCCATAAAATTGCAGTTTCATGTATG
TATATAATCATGCTCATGTATATTTAGTTACGTATAATGCTTTCTGAGTGAGTTTTACTCTTAAATCATT
TGGTTAAATCATTTGGCTTGCTGTTTACTCCCTTCTGTAGTTTTTAATTAAAAACTTTAAAGATAAGTCT
ACATTAAACAATGATCACATCTAAAGCTTTATCTTTGTGTAATCTAAGTATATGTGAGAAATCAGAATTG
GCATAATTTGTCTTAGTTGATATTCAAGGCTTTAAAAGTCATTATTCCTGGGCTTGGTAAGTGAATTTAT
GAGATTTACTGCTCTAGAAAGTATAGATGGCGAAAGGACCGTTTTGTATTGCTTCCTGATTACCAGTCTG
ATTATAC**CATGTGTGCTAATATACTTTT**TTTGTTATAGATTGTCTTAATGGTAGGTCAAGTAATAAAAG
AGATGAAATAATTTAAAAAAAAAAAAAAAA

33) Entrez Gene ID 5728 = PTEN, phosphatase and tensin homolog = U96180

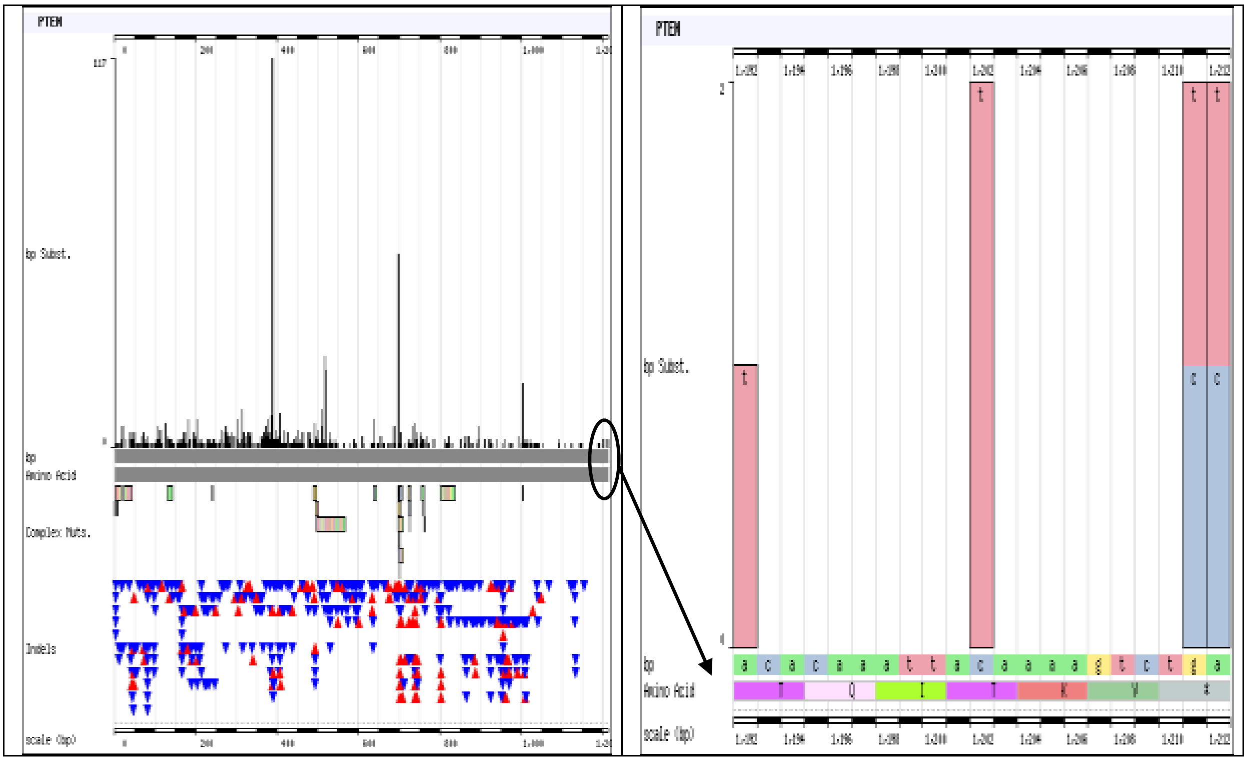

>gi|2039369|gb|U96180.1|HSU96180 Human protein tyrosine phosphatase (TEP1) mRNA, complete cds

GAATTCGGCACGAGGTGAGGCGAGGCCGGGCTCAGGCGAGGGAGATGAGAGACGGCGGCCGCGGCCC
GGAGCCCCTCTCAGCGCCTGTGAGCAGCCGCGGGGCAGCGCCCTCGGGGAGCCGGCCGGCCTGCGGCGG
CGGCAGCGGCGGCGTTTCTCGCCTCCTCTTCGTCTTTTCTAACCGTGCAGCCTCTTCCTCGGCTTCTCCT
GAAAGGGAAGGTGGAAGCCGTGGGCTCGGGCGGGAGCCGGCTGAGGCGCGGCGGCGGCGGCGGCACCTCC
CGCTCCTGGAGCGGGGGGGAGAAGCGGCGGCGGCGGCGGCCGCGGCGGCTGCAGCTCCAGGGAGGGGGTC
TGAGTCGCCTGTCACCATTTCCAGGGCTGGGAACGCCGGAGAGTTGGTCTCTCCCCTTCTACTGCCTCCA
ACACGGCGGCGGCGGCGGCGGCACATCCAGGGACCCGGGCCGGTTTTAAACCTCCCGTCCGCCGCCGCCG
CACCCCCCGTGGCCCGGGCTCCGGAGGCCGCCGGCGGAGGCAGCCGTTCGGAGGATTATTCGTCTTCTCC
CCATTCCGCTGCCGCCGCTGCCAGGCCTCTGGCTGCTGAGGAGAAGCAGGCCCAGTCGCTGCAACCATCC
AGCAGCCGCCGCAGCAGCCATTACCCGGCTGCGGTCCAGAGCCAAGCGGCGGCAGAGCGAGGGGCATCAG
CTACCGCCAAGTCCAGAGCCATTTCCATCCTGCAGAAGAAGCCCCGCCACCAGCAGCTTCTGCCATCTCT
CTCCTCCTTTTTCTTCAGCCACAGGCTCCCAGACATGACAGCCATCATCAAAGAGATCGTTAGCAGAAAC
AAAAGGAGATATCAAGAGGATGGATTCGACTTAGACTTGACCTATATTTATCCAAACATTATTGCTATGG
GATTTCCTGCAGAAAGACTTGAAGGCGTATACAGGAACAATATTGATGATGTAGTAAGGTTTTTGGATTC
AAAGCATAAAAACCATTACAAGATATACAATCTTTGTGCTGAAAGACATTATGACACCGCCAAATTTAAT
TGCAGAGTTGCACAATATCCTTTTGAAGACCATAACCCACCACAGCTAGAACTTATCAAACCCTTTTGTG
AAGATCTTGACCAATGGCTAAGTGAAGATGACAATCATGTTGCAGCAATTCACTGTAAAGCTGGAAAGGG
ACGAACTGGTGTAATGATATGTGCATATTTATTACATCGGGGCAAATTTTTAAAGGCACAAGAGGCCCTA
GATTTCTATGGGGAAGTAAGGACCAGAGACAAAAAGGGAGTAACTATTCCCAGTCAGAGGCGCTATGTGT
ATTATTATAGCTACCTGTTAAAGAATCATCTGGATTATAGACCAGTGGCACTGTTGTTTCACAAGATGAT
GTTTGAAACTATTCCAATGTTCAGTGGCGGAACTTGCAATCCTCAGTTTGTGGTCTGCCAGCTAAAGGTG
AAGATATATTCCTCCAATTCAGGACCCACACGACGGGAAGACAAGTTCATGTACTTTGAGTTCCCTCAGC
CGTTACCTGTGTGTGGTGATATCAAAGTAGAGTTCTTCCACAAACAGAACAAGATGCTAAAAAAGGACAA
AATGTTTCACTTTTGGGTAAATACATTCTTCATACCAGGACCAGAGGAAACCTCAGAAAAAGTAGAAAAT
GGAAGTCTATGTGATCAAGAAATCGATAGCATTTGCAGTATAGAGCGTGCAGATAATGACAAGGAATATC
TAGTACTTACTTTAACAAAAAATGATCTTGACAAAGCAAATAAAGACAAAGCCAACCGATACTTTTCTCC
AAATTTTAAGGTGAAGCTGTACTTCACAAAAACAGTAGAGGAGCCGTCAAATCCAGAGGCTAGCAGTTCA
ACTTCTGTAACACCAGATGTTAGTGACAATGAACCTGATCATTATAGATATTCTGACACCACTGACTCTG
ATCCAGAGAATGAACCTTTTGATGAAGATCAGCAT**A**CACAAATTA**C**AAAAGTCT**GA**ATTTTTTTTTATCA
AGAGGGATAAAACAC**CATGAAAATAAACTTGAATAA**ACTGAAAAAAAAAAAAAAAAAAA

34) Entrez Gene ID 5894 = RAF1, v-raf-1 murine leukemia viral oncogene homolog 1 = BC018119

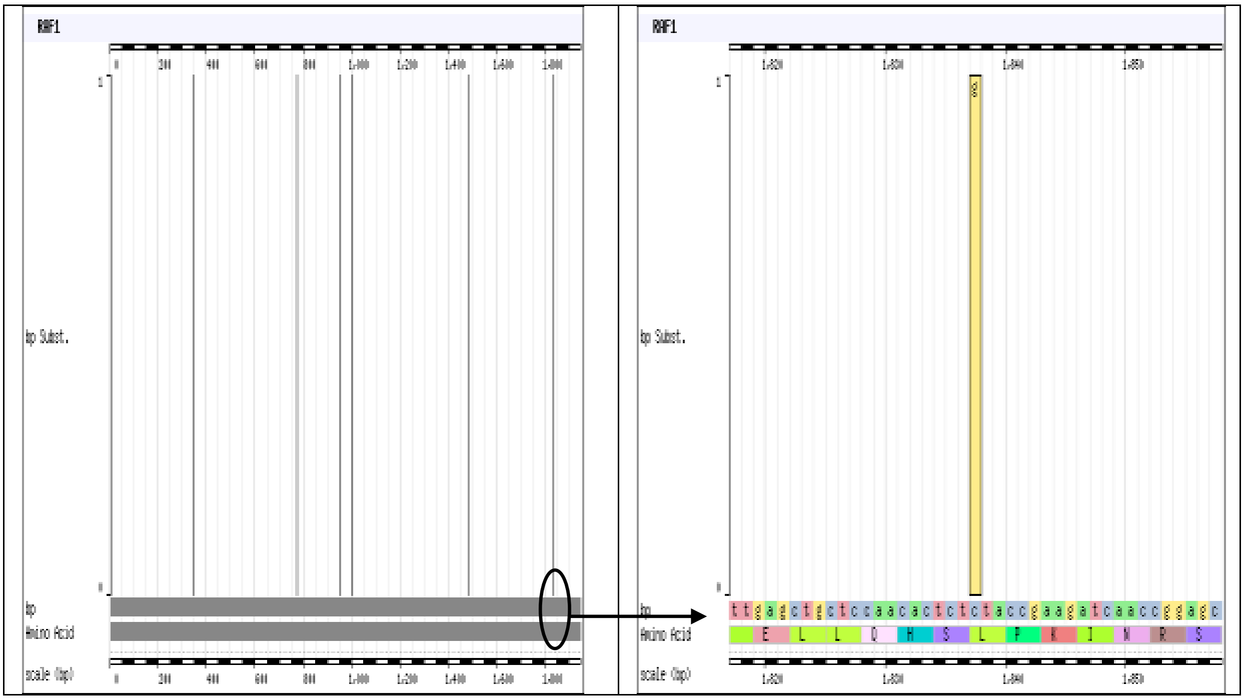

>gi|34190937|gb|BC018119.2| Homo sapiens v-raf-1 murine leukemia viral oncogene homolog 1, mRNA (cDNA clone MGC:9026 IMAGE:3904404), complete cds

TCGCGGGCGCTTGGGCCGCCATCTTAGATGGCGGGAGTAAGAGGAAAACGATTGTGAGGCGGGAACGGCT
TTCTGCTGCCTTTTTTGGGCCCCGAAAAGGGTCAGCTGGCCGGGCTTTGGGGCGCGTGCCCTGAGGCGCG
GAGCGCGTTTGCTACGATGCGGGGGCTGCTCGGGGCTCCGTCCCCTGGGCTGGGGACGCGCCGAATGTGA
CCGCCTCCCGCTCCCTCACCCGCCGCGGGGAGGAGGAGCGGGCGAGAAGCTGCCGCCGAACGACAGGACG
TTGGGGCGGCCTGGCTCCCTCAGGTTTAAGAATTGTTTAAGCTGCATCAATGGAGCACATACAGGGAGCT
TGGAAGACGATCAGCAATGGTTTTGGATTCAAAGATGCCGTGTTTGATGGCTCCAGCTGCATCTCTCCTA
CAATAGTTCAGCAGTTTGGCTATCAGCGCCGGGCATCAGATGATGGCAAACTCACAGATCCTTCTAAGAC
AAGCAACACTATCCGTGTTTTCTTGCCGAACAAGCAAAGAACAGTGGTCAATGTGCGAAATGGAATGAGC
TTGCATGACTGCCTTATGAAAGCACTCAAGGTGAGGGGCCTGCAACCAGAGTGCTGTGCAGTGTTCAGAC
TTCTCCACGAACACAAAGGTAAAAAAGCACGCTTAGATTGGAATACTGATGCTGCGTCTTTGATTGGAGA
AGAACTTCAAGTAGATTTCCTGGATCATGTTCCCCTCACAACACACAACTTTGCTCGGAAGACGTTCCTG
AAGCTTGCCTTCTGTGACATCTGTCAGAAATTCCTGCTCAATGGATTTCGATGTCAGACTTGTGGCTACA
AATTTCATGAGCACTGTAGCACCAAAGTACCTACTATGTGTGTGGACTGGAGTAACATCAGACAACTCTT
ATTGTTTCCAAATTCCACTATTGGTGATAGTGGAGTCCCAGCACTACCTTCTTTGACTATGCGTCGTATG
CGAGAGTCTGTTTCCAGGATGCCTGTTAGTTCTCAGCACAGATATTCTACACCTCACGCCTTCACCTTTA
ACACCTCCAGTCCCTCATCTGAAGGTTCCCTCTCCCAGAGGCAGAGGTCGACATCCACACCTAATGTCCA
CATGGTCAGCACCACCCTGCCTGTGGACAGCAGGATGATTGAGGATGCAATTCGAAGTCACAGCGAATCA
GCCTCACCTTCAGCCCTGTCCAGTAGCCCCAACAATCTGAGCCCAACAGGCTGGTCACAGCCGAAAACCC
CCGTGCCAGCACAAAGAGAGCGGGCACCAGTATCTGGGACCCAGGAGAAAAACAAAATTAGGCCTCGTGG
ACAGAGAGATTCAAGCTATTATTGGGAAATAGAAGCCAGTGAAGTGATGCTGTCCACTCGGATTGGGTCA
GGCTCTTTTGGAACTGTTTATAAGGGTAAATGGCACGGAGATGTTGCAGTAAAGATCCTAAAGGTTGTCG
ACCCAACCCCAGAGCAATTCCAGGCCTTCAGGAATGAGGTGGCTGTTCTGCGCAAAACACGGCATGTGAA
CATTCTGCTTTTCATGGGGTACATGACAAAGGACAACCTGGCAATTGTGACCCAGTGGTGCGAGGGCAGC
AGCCTCTACAAACACCTGCATGTCCAGGAGACCAAGTTTCAGATGTTCCAGCTAATTGACATTGCCCGGC
AGACGGCTCAGGGAATGGACTATTTGCATGCAAAGAACATCATCCATAGAGACATGAAATCCAACAATAT
ATTTCTCCATGAAGGCTTAACAGTGAAAATTGGAGATTTTGGTTTGGCAACAGTAAAGTCACGCTGGAGT
GGTTCTCAGCAGGTTGAACAACCTACTGGCTCTGTCCTCTGGATGGCCCCAGAGGTGATCCGAATGCAGG
ATAACAACCCATTCAGTTTCCAGTCGGATGTCTACTCCTATGGCATCGTATTGTATGAACTGATGACGGG
GGAGCTTCCTTATTCTCACATCAACAACCGAGATCAGATCATCTTCATGGTGGGCCGAGGATATGCCTCC
CCAGATCTTAGTAAGCTATATAAGAACTGCCCCAAAGCAATGAAGAGGCTGGTAGCTGACTGTGTGAAGA
AAGTAAAGGAAGAGAGGCCTCTTTTTCCCCAGATCCTGTCTTCCATTGAGCTGCTCCAACACTCT**C**TACC
GAAGATCAACCGGAGCGCTTCCGAGCCATCCTTGCATCGGGCAGCCCACACTGAGGATATCAATGCTTGC
ACGCTGACCACGTCCCCGAGGCTGCCTGTCTTCTAGTTGACTTTGCACCTGTCTTCAGGCTGCCAGGGGA
GGAGGAGAAGCCAGCAGGCACCACTTTTCTGCTCCCTTTCTCCAGAGGCAGAACACATGTTTTCAGAGAA

GCTGCTGCTAAGGACCTTCTAGACTGCTCACAGGGCCTTAACTTCATGTTGCCTTCTTTTCTATCCCTTT
GGGCCCTGGGAGAAGGAAGCCATTTGCAGTGCTGGTGTGTCCTGCTCCCTCCCCACATTCCCCATGCTCA
AGGCCCAGCCTTCTGTAGATGCGCAAGTGGATGTTGATGGTAGTACAAAAAGCAGGGGCCCAGCCCCAGC
TGTTGGCTACATGAGTATTTAGAGGAAGTAAGGTAGCAGGCAGTCCAGCCCTGATGTGGAGACACATGGG
ATTTGGAAATCAGCTTCTGGAGGAATGCATGTCACAGGCGGGACTTTCTTCAGAGAGTGGTGCAGCGCC
AGACATTTTGCACATAAGGCACCAAACAGCCCAGGACTGCCGAGACTCTGGCCGCCCGAAGGAGCCTGCT
TTGGTACTATGGAACTTTTCTTAGGGGACACGTCCTCCTTTCACAGCTTCTAAGGTGTCCAGTGCATTGG
GATGGTTTTCCAGGCAAGGCACTCGGCCAATCCGCATCTCAGCCCTCTCAGGGAGCAGTCTTCCATCATG
CTGAATTTTGTCTTCCAGGAGCTGCCCCTATGGGGCGGGGCCGCAGGGCCAGCCTTGTTTCTCTAACAAA
CAAACAAACAAACAGCCTTGTTTCTCTAGTCACATCATGTGTATACAAGGAAGCCAGGAATACAGGTTTT
CTTGATGATTTGGGTTTTAATTTTGTTTTTATTGCACCTGACAAAATACAGTTATCTGATGGTCCCTCAA
TTATGTTATTTTAATAAAATAAATTAAATTTAAAAAAAAAAAAAAAAAAAAAAAAAAAAAAAAAAA

35) Entrez Gene ID 5925 = RB1, retinoblastoma 1 = BC039060

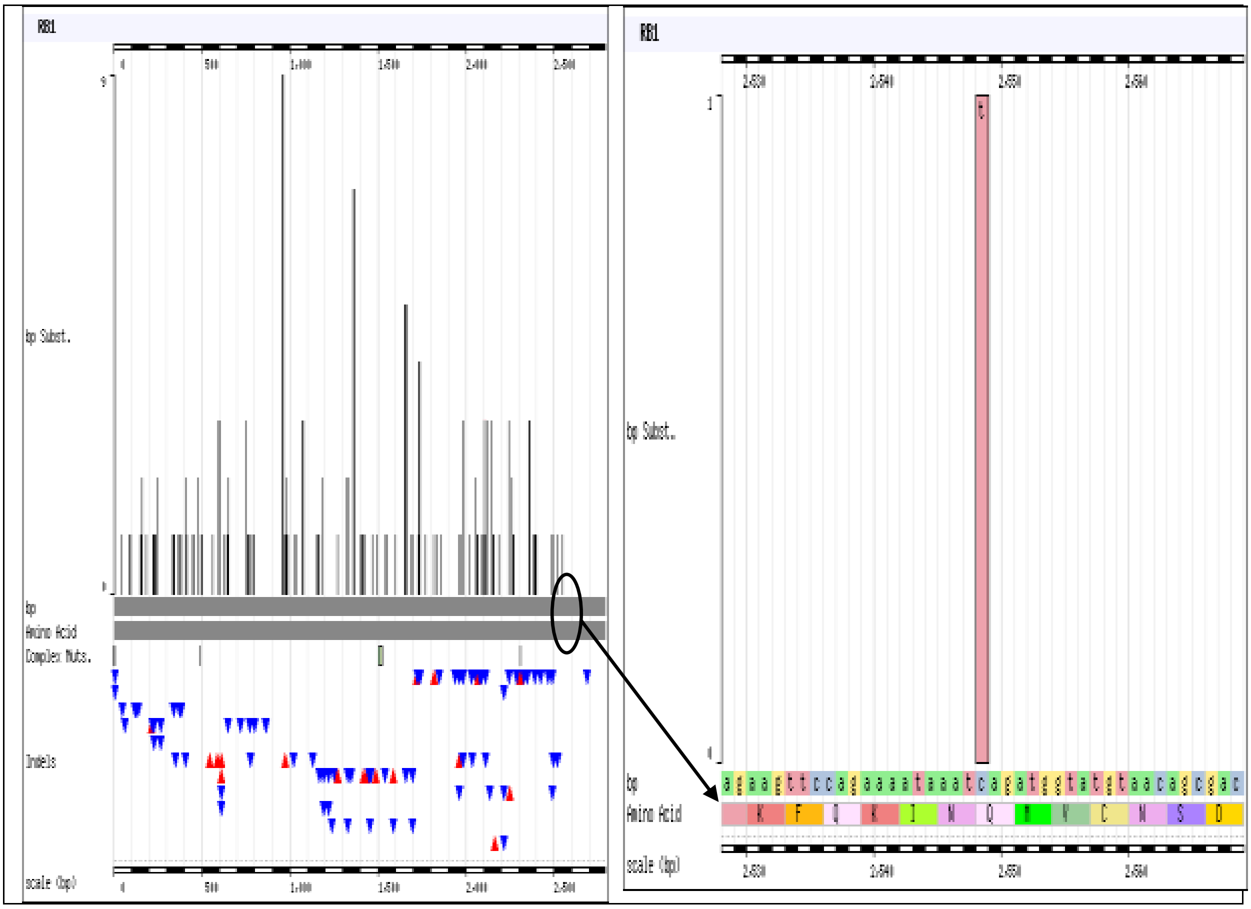

>gi|24660139|gb|BC039060.1| Homo sapiens retinoblastoma 1, mRNA (cDNA clone MGC:29887 IMAGE:5142020), complete cds
CGGGGGAGGCGCGTCCGGTTTTTCTCAGGGGACGTTGAAATTATTTTTGTAACGGGAGTCGGGAGAGGA
CGGGGCGTGCCCCGACGTGCGCGCGCGTCGTCCTCCCCGGCGCTCCTCCACAGCTCGCTGGCTCCCGCCG
CGGAAAGGCGTCATGCCGCCCAAAACCCCCCGAAAAACGGCCGCCACCGCCGCCGCTGCCGCCGCGGAAC
CCCCGGCACCGCCGCCGCCGCCCCCTCCTGAGGAGGACCCAGAGCAGGACAGCGGCCCGGAGGACCTGCC
TCTCGTCAGGCTTGAGTTTGAAGAAACAGAAGAACCTGATTTTACTGCATTATGTCAGAAATTAAAGATA
CCAGATCATGTCAGAGAGAGAGCTTGGTTAACTTGGGAGAAAGTTTCATCTGTGGATGGAGTATTGGGAG
GTTATATTCAAAAGAAAAAGGAACTGTGGGGAATCTGTATCTTTATTGCAGCAGTTGACCTAGATGAGAT
GTCGTTCACTTTTACTGAGCTACAGAAAAACATAGAAATCAGTGTCCATAAATTCTTTAACTTACTAAAA
GAAATTGATACCAGTACCAAAGTTGATAATGCTATGTCAAGACTGTTGAAGAAGTATGATGTATTGTTTG
CACTCTTCAGCAAATTGGAAAGGACATGTGAACTTATATATTTGACACAACCCAGCAGTTCGATATCTAC

TGAAATAAATTCTGCATTGGTGCTAAAAGTTTCTTGGATCACATTTTTATTAGCTAAAGGGGAAGTATTA
CAAATGGAAGATGATCTGGTGATTTCATTTCAGTTAATGCTATGTGTCCTTGACTATTTTATTAAACTCT
CACCTCCCATGTTGCTCAAAGAACCATATAAAACAGCTGTTATACCCATTAATGGTTCACCTCGAACACC
CAGGCGAGGTCAGAACAGGAGTGCACGGATAGCAAAACAACTAGAAAATGATACAAGAATTATTGAAGTT
CTCTGTAAAGAACATGAATGTAATATAGATGAGGTGAAAAATGTTTATTTCAAAAATTTTATACCTTTTA
TGAATTCTCTTGGACTTGTAACATCTAATGGACTTCCAGAGGTTGAAAATCTTTCTAAACGATACGAAGA
AATTTATCTTAAAAATAAAGATCTAGATGCAAGATTATTTTTGGATCATGATAAAACTCTTCAGACTGAT
TCTATAGACAGTTTTGAAACACAGAGAACACCACGAAAAAGTAACCTTGATGAAGAGGTGAATGTAATTC
CTCCACACACTCCAGTTAGGACTGTTATGAACACTATCCAACAATTAATGATGATTTTAAATTCAGCAAG
TGATCAACCTTCAGAAAATCTGATTTCCTATTTTAACAACTGCACAGTGAATCCAAAAGAAAGTATACTG
AAAAGAGTGAAGGATATAGGATACATCTTTAAAGAGAAATTTGCTAAAGCTGTGGGACAGGGTTGTGTCG
AAATTGGATCACAGCGATACAAACTTGGAGTTCGCTTGTATTACCGAGTAATGGAATCCATGCTTAAATC
AGAAGAAGAACGATTATCCATTCAAAATTTTAGCAAACTTCTGAATGACAACATTTTTCATATGTCTTTA
TTGGCGTGCGCTCTTGAGGTTGTAATGGCCACATATAGCAGAAGTACATCTCAGAATCTTGATTCTGGAA
CAGATTTGTCTTTCCCATGGATTCTGAATGTGCTTAATTTAAAAGCCTTTGATTTTTACAAAGTGATCGA
AAGTTTTATCAAAGCAGAAGGCAACTTGACAAGAGAAATGATAAAACATTTAGAACGATGTGAACATCGA
ATCATGGAATCCCTTGCATGGCTCTCAGATTCACCTTTATTTGATCTTATTAAACAATCAAAGGACCGAG
AAGGACCAACTGATCACCTTGAATCTGCTTGTCCTCTTAATCTTCCTCTCCAGAATAATCACACTGCAGC
AGATATGTATCTTTCTCCTGTAAGATCTCCAAAGAAAAAAGGTTCAACTACGCGTGTAAATTCTACTGCA
AATGCAGAGACACAAGCAACCTCAGCCTTCCAGACCCAGAAGCCATTGAAATCTACCTCTCTTTCACTGT
TTTATAAAAAAGTGTATCGGCTAGCCTATCTCCGGCTAAATACACTTTGTGAACGCCTTCTGTCTGAGCA
CCCAGAATTAGAACATATCATCTGGACCCTTTTCCAGCACACCCTGCAGAATGAGTATGAACTCATGAGA
GACAGGCATTTGGACCAAATTATGATGTGTTCCATGTATGGCATATGCAAAGTGAAGAATATAGACCTTA
AATTCAAAATCATTGTAACAGCATACAAGGATCTTCCTCATGCTGTTCAGGAGACATTCAAACGTGTTTT
GATCAAAGAAGAGGAGTATGATTCTATTATAGTATTCTATAACTCGGTCTTCATGCAGAGACTGAAAACA
AATATTTTGCAGTATGCTTCCACCAGGCCCCCTACCTTGTCACCAATACCTCACATTCCTCGAAGCCCTT
ACAAGTTTCCTAGTTCACCCTTACGGATTCCTGGAGGGAACATCTATATTTCACCCCTGAAGAGTCCATA
TAAAATTTCAGAAGGTCTGCCAACACCAACAAAAATGACTCCAAGATCAAGAATCTTAGTATCAATTGGT
GAATCATTCGGGACTTCTGAGAAGTTCCAGAAAATAAAT**C**AGATGGTATGTAACAGCGACCGTGTGCTCA
AAAGAAGTGCTGAAGGAAGCAACCCTCCTAAACCACTGAAAAAACTACGCTTTGATATTGAAGGATCAGA
TGAAGCAGATGGAAGTAAACATCTCCCAGGAGAGTCCAAATTTCAGCAGAAACTGGCAGAAATGACTTCT
ACTCGAACACGAATGCAAAAGCAGAAAATGAATGATAGCATGGATACCTCAAACAAGGAAGAGAAATGAG
GATCTCAGGACCTTGGTGGACACTGTGTACACCTCTGGATTCATTGTCTCTCACAGATGTGACTGTATAA
CTTTCCCAGGTTCTGTTTATGGCCACATTTAATATCTTCAGCTCTTTTTGTGGATATAAAATGTGCAGAT
GCAATTGTTTGGGTGATTCCTAAGCCACTTGAAATGTTAGTCATTGTTATTTATACAAGATTGAAAATCT
TGTGTAAATCCTGCCATTTAAAAAGTTGTAGCAGATTGTTTCCTCTTCCAAAGTAAAATTGCTGTGCTTT
ATGGATAGTAAGAATGGCCCTAGAGTGGGAGTCCTGATAACCCAGGCCTGTCTGACTACTTTGCCTTCTT
TTGTAGCATATAGGTGATGTTTGCTCTTGTTTTTATTAATTTATATGTATATTTTTTTAATTTAACATGA
ACACCCTTAGAAAATGTGTCCTATCTATCTTCCAAATGCAATTTGATTGACTGCCCATTCACCAAAATTA
TCCTGAACTCTTCTGCAAAAATGGATATTATTAGAAATTAGAAAAAAATTACTAATTTTACACATTAGAT
TTTATTTTACTATTGGAATCTGATATACTGTGTGCTTGTTTTATAAAATTTTGCTTTTAATTAAATAAAA
GCTGGAAGCAAAGTATAACCATATGATACTATCATACTACTGAAACAGATTTCATACCTCAGAATGTAAA
AGAACTTACTGATTATTTTCTTCATCCAACTTATGTTTTTAAATGAGGATTATTGATAGTACTCTTGGTT
TTTATACCATTCAGATCACTGAATTTATAAAGTACCCATCTAGTACTTGAAAAAGTAAAGTGTTCTGCCA
GATCTTAGGTATAGAGGACCCTAACACAGTATATCCCAAGTGCACTTTCTAATGTTTCTGGGTCCTGAAG
AATTAAGATACAAATTAATTTTACTCCATAAACAGACTGTTAATTATAGGAGCCTTAATTTTTTTTTCAT
AGAGATTTGTCTAATTGCATCTCAAAATTATTCTGCCCTCCTTAATTTGGGAAGGTTTGTGTTTTCTCTG
GAATGGTACATGTCTTCCATGTATCTTTTGAACTGGCAATTGTCTATTTATCTTTTATTTTTTTTAAGTC
AGTATGGTCTAACACTGGCATGTTCAAAGCCACATTATTTCTAGTCCAAAATTACAAGTAATCAAGGGTC
ATTATGGGTTAGGCATTAATGTTTCTATCTGATTTTGTGCAAAAGCTTCAAATTAAAACAGCTGCATTAG
AAAAAGAGGCGCTTCTCCCCTCCCCTACACCTAAAGGTGTATTTAAACTATCTTGTGTGATTAACTTATT
TAGAGATGCTGTAACTTAAAATAGGGGATATTTAAGGTAGCTTCAGCTAGCTTTTAGGAAAATCACTTTG
TCTAACTCAGAATTATTTTTAAAAAGAAATCTGGTCTTGTTAGAAAACAAAATTTTATTTTGTGCTCATT
TAAGTTTCAAACTTACTATTTTGACAGTTATTTTGATAACAATGACACTAGAAAACTTGACTCCATTTCA
TCATTGTTTCTG**CATGAATATCATACAAATCAG**TTAGTTTTTAGGTCAAGGGCTTACTATTTCTGGGTCT
TTTGCTACTAAGTTCACATTAGAATTAGTGCCAGAATTTTAGGAACTTCAGAGATCGTGTATTGAGATTT
CTTAAATAATGCTTCAGATATTATTGCTTTATTGCTTTTTTGTATTGGTTAAAACTGTACATTTAAAATT
GCTATGTTACTATTTTCTACAATTAATAGTTTGTCTATTTTAAAATAAATTAGTTGTTAAGAGTCAAAAA
AAAAAAAAAAAAAAAAAAAA

36) Entrez Gene ID 6146 = RPL22, ribosomal protein L22 = NM_000983

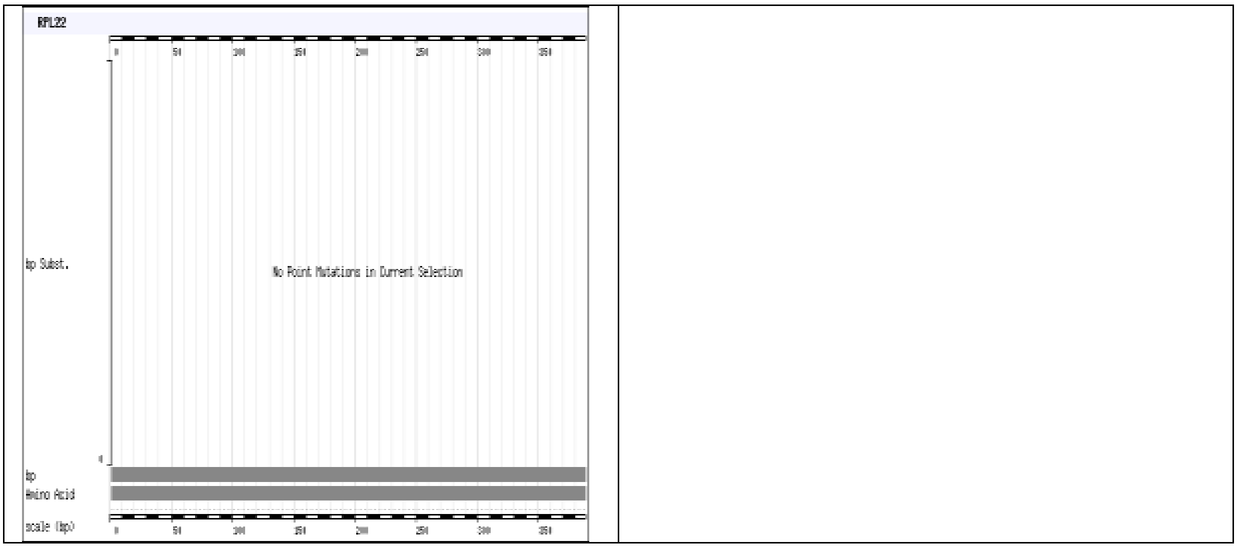

Although RPL22 belongs to the list of COSMIC (v56) somatically mutated genes in cancer, no point mutation has been recorded.

37) Entrez Gene ID 6416 = MAP2K4, mitogen-activated protein kinase kinase 4 = BC036032

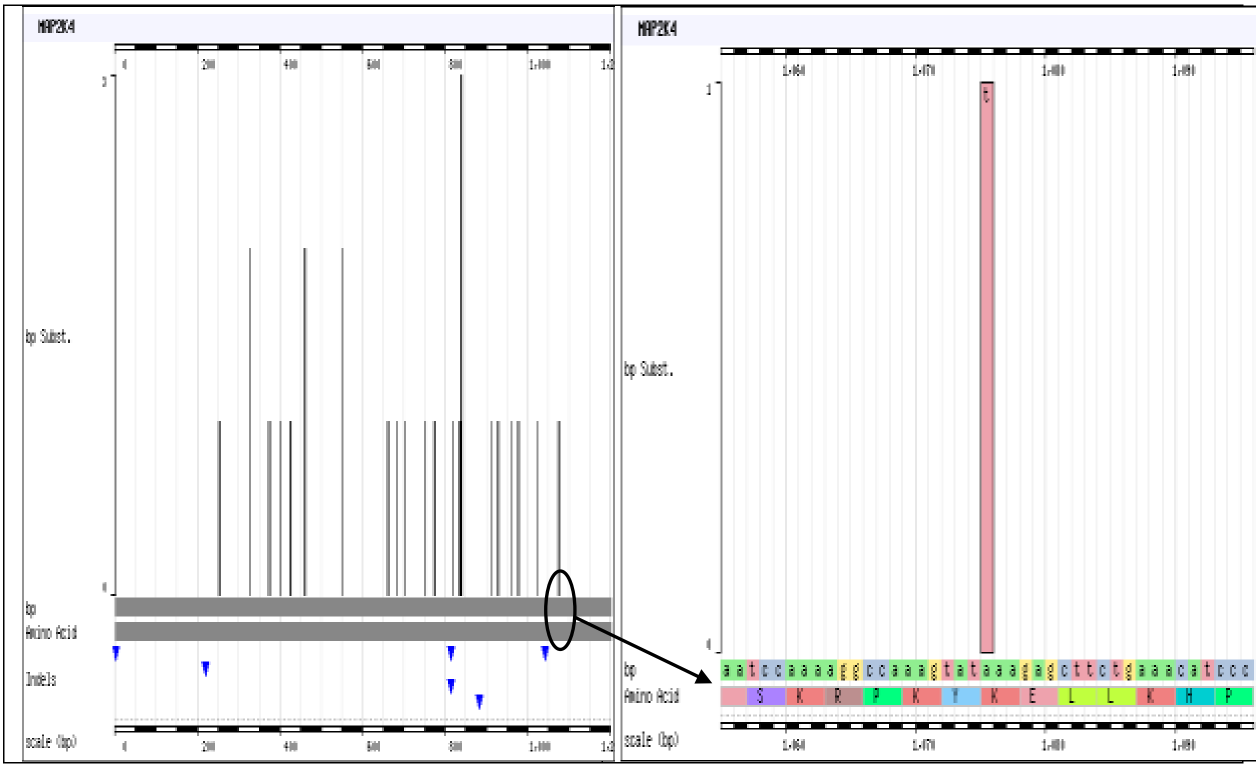

>gi|23241687|gb|BC036032.1| Homo sapiens mitogen-activated protein kinase kinase 4, mRNA (cDNA clone MGC:33126 IMAGE:5272439), complete cds
GGCCGTGCGAGAGGCCGAGCTTGCTGCATTGCAGCCGCCGCGGCGCCGCTCGGCTCTTCACTCCCAACAA
TGGCGGCTCCGAGCCCGAGCGGCGGCGGCGGCTCCGGGGGCGGCCGCGGCAGCGGCACCCCCGGCCCCGT

AGGGTCCCCGGCGCCAGGCCACCCGGCCGTCAGCAGCATGCAGGGTAAACGCAAAGCACTGAAGTTGAAT
TTTGCAAATCCACCTTTCAAATCTACAGCAAGGTTTACTCTGAATCCCAATCCTACAGGAGTTCAAAACC
CACACATAGAGAGACTGAGAACACACAGCATTGAGTCATCAGGAAAACTGAAGATCTCCCCTGAACAACA
CTGGGATTTCACTGCAGAGGACTTGAAAGACCTTGGAGAAATTGGACGAGGAGCTTATGGTTCTGTCAAC
AAAATGGTCCACAAACCAAGTGGGCAAATAATGGCAGTTAAAAGAATTCGGTCAACAGTGGATGAAAAAG
AACAAAAACAACTTCTTATGGATTTGGATGTAGTAATGCGGAGTAGTGATTGCCCATACATTGTTCAGTT
TTATGGTGCACTCTTCAGAGAGGGTGACTGTTGGATCTGTATGGAACTCATGTCTACCTCGTTTGATAAG
TTTTACAAATATGTATATAGTGTATTAGATGATGTTATTCCAGAAGAAATTTTAGGCAAAATCACTTTAG
CAACTGTGAAAGCACTAAACCACTTAAAAGAAAACTTGAAAATTATTCACAGAGATATCAAACCTTCCAA
TATTCTTCTGGACAGAAGTGGAAATATTAAGCTCTGTGACTTCGGCATCAGTGGACAGCTTGTGGACTCT
ATTGCCAAGACAAGAGATGCTGGCTGTAGGCCATACATGGCACCTGAAAGAATAGACCCAAGCGCATCAC
GACAAGGATATGATGTCCGCTCTGATGTCTGGAGTTTGGGGATCACATTGTATGAGTTGGCCACAGGCCG
ATTTCCTTATCCAAAGTGGAATAGTGTATTTGATCAACTAACACAAGTCGTGAAAGGAGATCCTCCGCAG
CTGAGTAATTCTGAGGAAAGGGAATTCTCCCCGAGTTTCATCAACTTTGTCAACTTGTGCCTTACGAAGG
ATGAATCCAAAAGGCCAAAGTAT**A**AAGAGCTTCTGAAACATCCCTTTATTTTGATGTATGAAGAACGTGC
CGTTGAGGTCGCATGCTATGTTTGTAAAATCCTGGATCAAATGCCAGCTACTCCCAGCTCTCCCATGTAT
GTCGATTGATATCGCTGCTACATCAGACTCTAGAAAAAAGGGCTGAGAGGAAGCAAGACGTAAAGAATTT
TCATCCCGTATCACAGTGTTTTTATTGCTCGCCCAGACACCATGTGCAATAAGATTGGTGTTCGTTTCCA
TCATGTCTGTATACTCCTGTCACCTAGAACGTGCATCCTTGTAATACCTGATTGATCACACAGTGTTAGT
GCTGGTCAGAGAGACCTCATCCTGCTCTTTTGTGATGAACATATTCATGAAATGTGGAAGTCAGTACGAT
CAAGTTGTTGACTGTGATTAGATCACATCTTAAATTCATTTCTAGACTCAAAACCTGGAGATGCAGCTAC
TGGAATGGTGTTTTGTCAGACTTCCAAATCCTGGAAGGACACAGTGATGAATGTACTATGTCTGAACATA
GAAACTCGGGCTTGAGTGAGAAGAGCTTGCACAGCCAACGAGACACATTGCCTTCTGGAGCTGGGAGACA
AAGGAGGAATTTACTTTCTTCACCAAGTGCAATAGATTACTGATGTGATATTCTGTTGCTTTACAGTTAC
AGTTGATGTTTGGGGATCGATGTGCTCAGCCAAATTTCCTGTTTGAAATATCATGTTAAATTAGAATGAA
TTTATCTTTACCAAAAACCATGTTGCGTTCAAAGAGGTGAACATTAAAATATAGAGACAGGACAGAATGT
GTTCTTTTCTCCTTTACCAGTCCTATTTTTCAATGGGAAGACTCAGGAGTCTGCCACTTGTCAAAGAAGG
TGCTGATCCTAAGAATTTTTCATTCTCAGAATTCGGTGTGCTGCCAACTTGATGTTCCACCTGCCACAAA
CCACCAGGACTGAAAGAAGAAAACAGTACAGAAGGCAAAGTTTACAGATGTTTTTAATTCTAGTATTTTA
TCTGGAACAACTTGTAGCAGCTATATATTTCCCCTTGGTCCCAAGCCTGATACTTTAGCCATCATAACTC
ACTAACAGGGAGAAGTAGCTAGTAGCAATGTGCCTTGATTGATTAGATAAAGATTTCTAGTAGGCAGCAA
AAGACCAAATCTCAGTTGTTTGCTTCTTGCCATCACTGGTCCAGGTCTTCAGTTTCCGAATCTCTTTCCC
TTCCCCTGTGGTCTATTGTCGCTATGTGACTTGCGCTTAATCCAATATTTTGCCTTTTTTCTATATCAAA
AAACCTTTACAGTTAGCAGGGATGTTCCTTACCAAGGATTTTTAGCCCCAAATCTCTCATATTCGCTAGT
GTTTAAAAGGCTAAGAATAGTGGGGCCCAGCCGATGTGGTAGGTGATAAAGAGGCATCTTTTCTAGAGAC
ACATTGAACCAGATGAGGATCCGAAACGGCAGCCTTTACGTTCATCACCTGCTAGAACCTCTCGTAGTCC
ATCACCATTTCTTGGCATTGGAATTCTACTGGAAAAAAATACAAAAAGCAAAACAAAACCCTCAGCACTG
TTACAAGAGGCCATTTAAGTATCTTGTGCTTCTTCACTTACCCATTAGCCAGGTTCTCATTAGGTTTTGC
TTGGGCCTCCCTGGCACTGAACCTTAGGCTTTGCATGACAGTGAAGCAGCACTGTGAGTGGTTCAAGCAC
ACTGGAATATAAAACAGTCATGGCCTGAGATGCAGGTGATGCCATTACAGAACCAAATCGTGGCACGTAT
TGCTGTGTCTCCTCTCAGAGTGACAGTCATAAATACTGTCAAACAATAAAGGGAGAATGGTGCTGTTTAA
AGTCACATCCCTGTAAATTGCAGAATTCAAAAGTGATTATCTCTTTGATCTACTTGCCTCATTTCCCTAT
CTTCTCCCCCACGGTATCCTAAACTTTAGACTTCCCACTGTTCTGAAAGGAGACATTGCTCTATGTCTGC
CTTCGACCACAGCAAGCCATCATCCTCCATTGCTCCCGGGGACTCAAGAGGAATCTGTTTCTCTGCTGTC
AACTTCCCATCTGGCTCAGCATAGGGTCACTTTGCCATTATGCAAATGGAGATAAAAGCAATTCTGACTG
TCCAGGAGCTAATCTGACCGTTCTATTGTGTGGATGACCACATAAGAAGGCAATTTTAGTGTATTAATCA
TAGATTATTATAAACTATAAACTTAAGGGCAAGGAGTTTATTACAATGTATCTTTATTAAAACAAAAGGG
TGTATAGTGTTCACAAACTGTGAAAATAGTGTAAGAACTGTACATTGTGAGCTCTGGTTATTTTTCTCTT
GTACCATAGAAAAATGTATAAAAATTATCAAAAAGCTAATGTGCAGGGATATTGCCTTATTTGTCTGTAA
AAAATGGAGCTCAGTAACATAACTGCTTCTTGGAGCTTTGGAATATTTTATCCTGTATTCTTGTTTGAAT
TCCTCCTCTATTTAAGATATATA**CATGGAATCGAAGTGTTTATG**TAATAGTTCTATCCTTTTGCCTGCAG
GTCAGTTGTAATAAATCTAGGATGTGATGAAGAAAAAAAAAAAAAAA

38) Entrez Gene ID 6418 = SET, SET nuclear oncogene = M93651

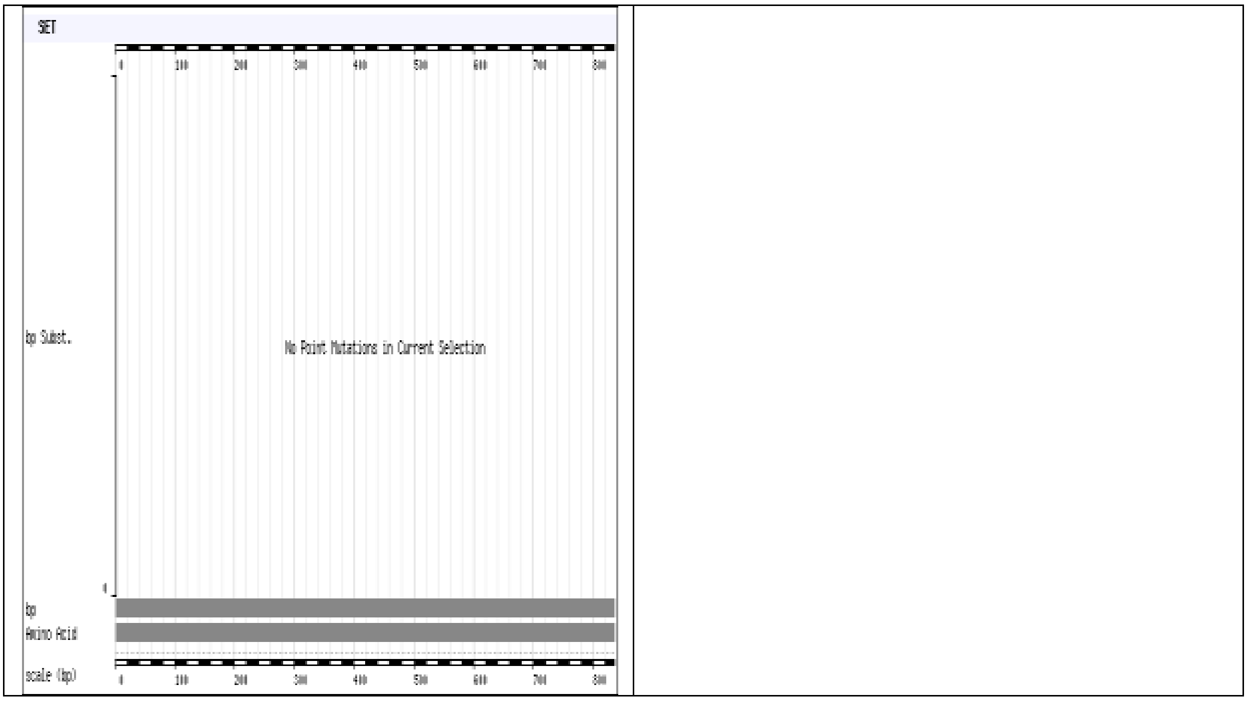

Although SET belongs to the list of COSMIC (v56) somatically mutated genes in cancer, no point mutation has been recorded.

39) Entrez Gene ID 6428 = SRSF3, serine/arginine-rich splicing factor 3 = AF107405

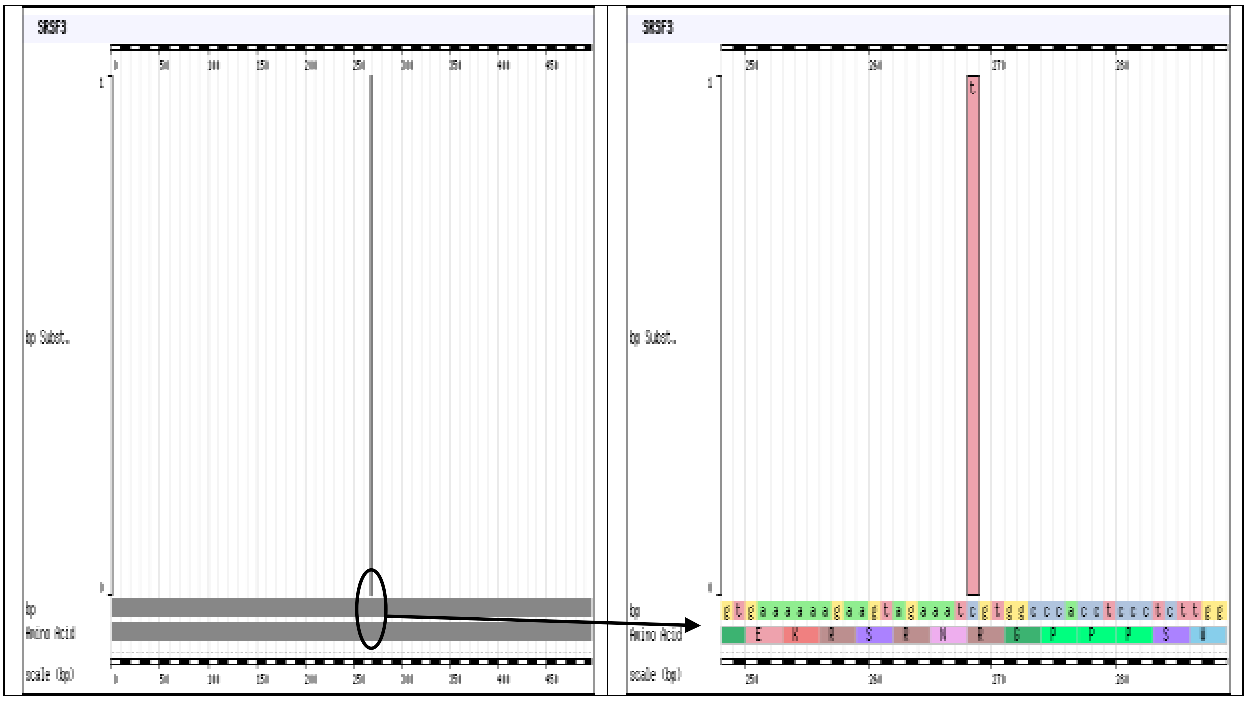

>gi|5531903|gb|AF107405.1| Homo sapiens pre-mRNA splicing factor (SFRS3) mRNA, complete cds
GAGAGAGTTGGTTGGTGTTGGGCCGGAGGAAAGCGGGAAGACTCATCGGAGCGTGTGGATTTGAGCCGCC
GCATTTTTTAACCCTAGATCTCGAAATGCATCGTGATTCCTGTCCATTGGACTGTAAGGTTTATGTAGGC
AATCTTGGAAACAATGGCAACAAGACGGAATTGGAACGGGCTTTTGGCTACTATGGACCACTCCGAAGTG

TGTGGGTTGCTAGAAACCCACCCGGCTTTGCTTTTGTTGAATTTGAAGATCCCCGAGATGCAGCTGATGC
AGTCCGAGAGCTAGATGGAAGAACACTATGTGGCTGCCGTGTAAGAGTGGAACTGTCGAATGGTGAAAAA
AGAAGTAGAAAT**C**GTGGCCCACCTCCCTCTTGGGGTCGTCGCCCTCGAGATGATTATCGTAGGAGGAGTC
CTCCACCTCGTCGCAGATCTCCAAGAAGGAGAAGCTTCTCTCGCAGCCGGAGCAGGTCCCTTTCTAGAGA
TAGGAGAAGAGAGAGATCGCTGTCTCGGGAGAGAAATCACAAGCCGTCCCGATCCTTCTCTAGGTCTCGT
AGTCGATCTAGGTCAAATGAAAGGAAATAGAAGACAGTTTGCAAGAGAAGTGGTGTACAGGAAATTACTT
CATTTGACAGGAGTATGTACAGAAAATTCAAGTTTTGTTTGAGACTTCATAAGCTTGGTGCATTTTTAAG
ATGTTTTAGCTGTTCAAATCTGTTTGTCTCTTGAAACAGTGACACAAAGGTGTAATTCTCTATGGTTTGA
AATGGATCATACGAGGCATGTAATACCAAGAATTGTTACTTTACAATGTTCCCTTAAGCAAAATTGAATT
TGCTTTGAACTTTTAGTTATGCACAGACTGATAATAAACCTCTAAACCTGCCCAGCGGAAGTGTGTTTTT
TTTTAAATTTAAATACAGAAACAACTGGCAAAAATGAACTAAGATTTACTTTTTTTTCCATAGCTGGGAT
ATAGGCTGCAGCTATAGTTGAACAAGCAGTCTTTAAAAACTGCTGTGAAACACAGGCCATCAGGGAAAAC
GAAATGCTGCACTATTAAATTAGAGGTTTTTGAAAAATCCAACTCTCATCCTGGGCAGAGGTTGCCTAGT
TGGTATAGAATGTTAAGTTTCAAGAAAGTTTACCTTTGCTTTAGGTCATAAGTTCCTTATTTGATTGCTG
TATATGGATACATGGCTGTTCGTGACATTCTTTATGTGCAAATTTGTGATTTCAAAAATGTCCTGCCAGT
TTAAGGGTACATTGTAGAGCCGAACTTTGAGTTACTGTGCAAGATTTTTTTTTCATGCTGTCATTTGTAA
TATGTTTTGTGAGAATCCTTGGGATTAAAGTTTTGGTTACAAATTGTTCTTTAACTTGAAAGCCTGTTTT
TCCTTGCAAACTCAAATCTGTGAGCTTGGTACCAAGTCCAGGTATAACATTCCTATTGGAAGCCATACTT
ATATTTTCTTGTAAAGTGCTTTTGAATTAATAAAATATTAGCATAATTGTGTATAGTCAGTTGAACCCAC
TGTTACCATTGTTCTTATCCCATGGGAAGCAGTTGGTTACACGATTCTTATTTTATAAGAAACAGCTGAG
AGGCACTATGGATTAGTCTTCTGAAGTGAAGGAAATATAGATGTCACCTAAGTGATAGTTAACCCATTTT
TTTTTTTTTTAGGCATAGAAGCCAGTTCAGGGTCCATAATATTTAGTGACCAACATTTTAAAGTATAGCA
GCAACCTGGTTCTTAAACACAAAGTAAGTTGCCCATTAACAAATGGCTTTTATCTTTAGCATGAAAACTT
TCCACAGGTCTAAAAATTGCTTCCATTTTATAATTTGAGGTGTTGCATGGGAATTCTAAGCTGATCCATC
ATGATGTAAAAGTTCACAATATGGTTCAAATGTAACAGTGCAGAATTGAATATGGAGG**CATGCATAACCT**
TCCTCTTAGAAAATGGCAGGTGTTGTAATTTCAAATTTTTGTGCAATTAGATTAAATCATAATGCAACAG
TCAAAAAAAAAAAAAAAAAAAAAAAAAAAA

40) Entrez Gene ID 6597 = SMARCA4, SWI/SNF related, matrix associated, actin dependent regulator of chromatin, subfamily a, member 4 = BC150298

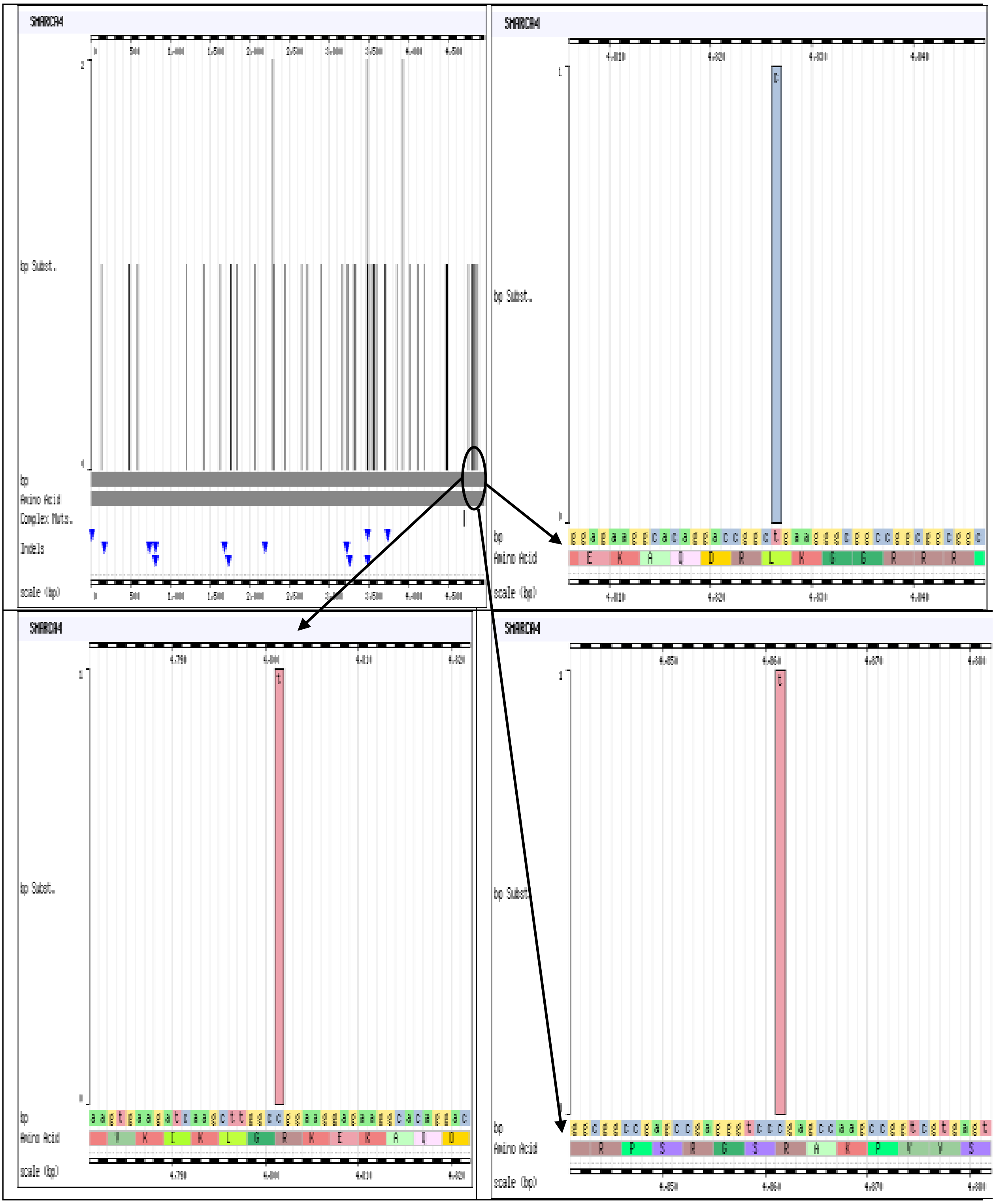
SMARCA4
bp Subst.
bp
Amino Acid
Complex Muts.
Indels
scale (bp)
SMARCA4
bp Subst.
bp
Amino Acid
scale (bp)
SMARCA4
bp Subst.
bp
Amino Acid
scale (bp)
SMARCA4
bp Subst.
bp
Amino Acid
scale (bp)

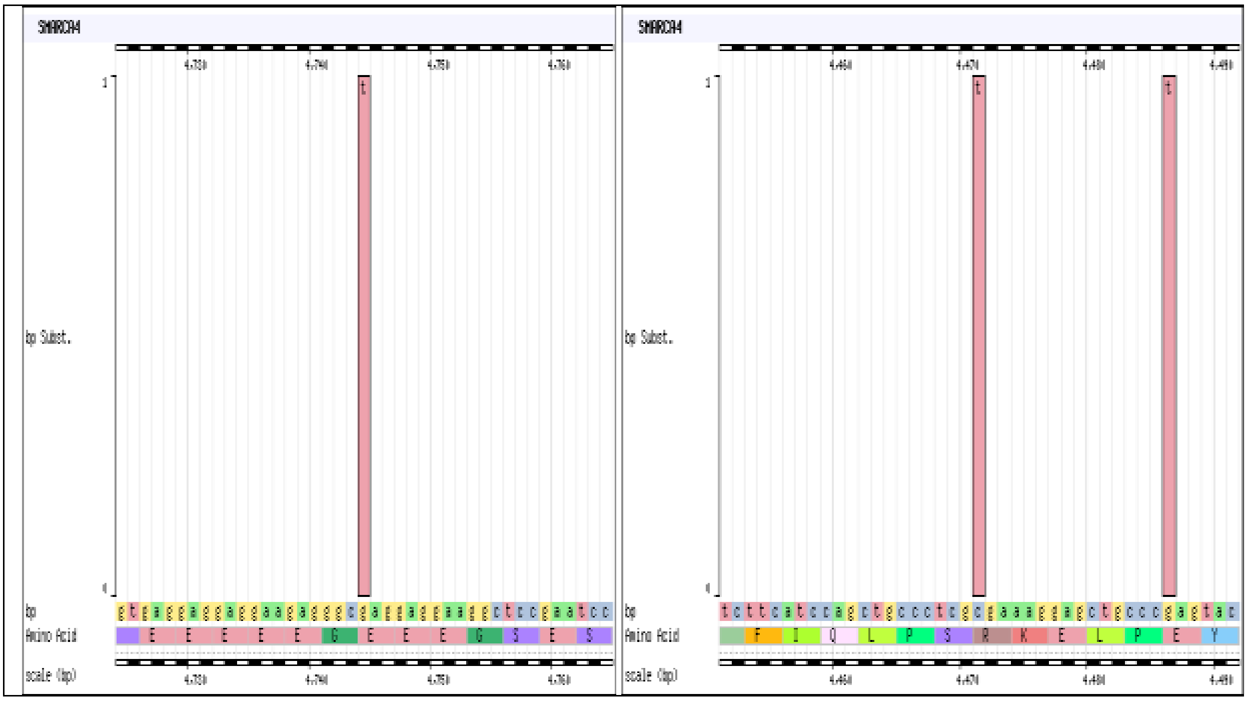

>gi|152013070|gb|BC150298.1| Homo sapiens SWI/SNF related, matrix associated, actin dependent regulator of chromatin, subfamily a, member 4, mRNA (cDNA clone MGC:167032 IMAGE:8860365), complete cds

GTGAAGGGGGGGCGGTGGCCGAGGCGGGCGGGCGCGCGCGAGGCTTCCCCTCGTTTGGCGGCGGCGGC
GGCTTCTTTGTTTCGTGAAGAGAAGCGAGACGCCCATTCTGCCCCCGGCCCCGCGCGGAGGGGCGGGGGA
GGCGCCGGGAAGTCGACGGCGCCGGCGGCTCCTGCAGGAGGCCACTGTCTGCAGCTCCCGTGAAGATGTC
CACTCCAGACCCACCCCTGGGCGGAACTCCTCGGCCAGGTCCTTCCCCGGGCCCTGGCCCTTCCCCTGGA
GCCATGCTGGGCCCTAGCCCGGGTCCCTCGCCGGGCTCCGCCCACAGCATGATGGGGCCCAGCCCAGGGC
CGCCCTCAGCAGGACACCCCATCCCCACCCAGGGGCCTGGAGGGTACCCTCAGGACAACATGCACCAGAT
GCACAAGCCCATGGAGTCCATGCATGAGAAGGGCATGTCGGACGACCCGCGCTACAACCAGATGAAAGGA
ATGGGGATGCGGTCAGGGGGCCATGCTGGGATGGGGCCCCCGCCCAGCCCCATGGACCAGCACTCCCAAG
GTTACCCCTCGCCCCTGGGTGGCTCTGAGCATGCCTCTAGTCCAGTTCCAGCCAGTGGCCCGTCTTCGGG
GCCCCAGATGTCTTCCGGGCCAGGAGGTGCCCCGCTGGATGGTGCTGACCCCCAGGCCTTGGGGCAGCAG
AACCGGGGCCCAACCCCATTTAACCAGAACCAGCTGCACCAGCTCAGAGCTCAGATCATGGCCTACAAGA
TGCTGGCCAGGGGGCAGCCCCTCCCCGACCACCTGCAGATGGCGGTGCAGGGCAAGCGGCCGATGCCCGG
GATGCAGCAGCAGATGCCAACGCTACCTCCACCCTCGGTGTCCGCAACAGGACCCGGCCCTGGCCCTGGC
CCTGGCCCCGGCCCGGGTCCCGGCCCGGCACCTCCAAATTACAGCAGGCCTCATGGTATGGGAGGGCCCA
ACATGCCTCCCCCAGGACCCTCGGGCGTGCCCCCCGGGATGCCAGGCCAGCCTCCTGGAGGGCCTCCCAA
GCCCTGGCCTGAAGGACCCATGGCGAATGCTGCTGCCCCCACGAGCACCCCTCAGAAGCTGATTCCCCCG
CAGCCAACGGGCCGCCCTTCCCCCGCGCCCCCTGCCGTCCCACCCGCCGCCTCGCCCGTGATGCCACCGC
AGACCCAGTCCCCCGGGCAGCCGGCCCAGCCCGCGCCCATGGTGCCACTGCACCAGAAGCAGAGCCGCAT
CACCCCCATCCAGAAGCCGCGGGGCCTCGACCCTGTGGAGATCCTGCAGGAGCGCGAGTACAGGCTGCAG
GCTCGCATCGCACACCGAATTCAGGAACTTGAAAACCTTCCCGGGTCCCTGGCCGGGGATTTGCGAACCA
AAGCGACCATTGAGCTCAAGGCCCTCAGGCTGCTGAACTTCCAGAGGCAGCTGCGCCAGGAGGTGGTGGT
GTGCATGCGGAGGGACACAGCGCTGGAGACAGCCCTCAATGCTAAGGCCTACAAGCGCAGCAAGCGCCAG
TCCCTGCGCGAGGCCCGCATCACTGAGAAGCTGGAGAAGCAGCAGAAGATCGAGCAGGAGCGCAAGCGCC
GGCAGAAGCACCAGGAATACCTCAATAGCATTCTCCAGCATGCCAAGGATTTCAAGGAATATCACAGATC
CGTCACAGGCAAAATCCAGAAGCTGACCAAGGCAGTGGCCACGTACCATGCCAACACGGAGCGGGAGCAG
AAGAAAGAGAACGAGCGGATCGAGAAGGAGCGCATGCGGAGGCTCATGGCTGAAGATGAGGAGGGGTACC
GCAAGCTCATCGACCAGAAGAAGGACAAGCGCCTGGCCTACCTCTTGCAGCAGACAGACGAGTACGTGGC
TAACCTCACGGAGCTGGTGCGGCAGCACAAGGCTGCCCAGGTCGCCAAGGAGAAAAAGAAGAAAAAGAAA
AAGAAGAAGGCAGAAAATGCAGAAGGACAGACGCCTGCCATTGGGCCGGATGGCGAGCCTCTGGACGAGA
CCAGCCAGATGAGCGACCTCCCGGTGAAGGTGATCCACGTGGAGAGTGGGAAGATCCTCACAGGCACAGA
TGCCCCCAAAGCCGGGCAGCTGGAGGCCTGGCTCGAGATGAACCCGGGGTATGAAGTAGCTCCGAGGTCT
GATAGTGAAGAAAGTGGCTCAGAAGAAGAGGAAGAGGAGGAGGAGGAAGAGCAGCCGCAGGCAGCACAGC
CTCCCACCCTGCCCGTGGAGGAGAAGAAGAAGATTCCAGATCCAGACAGCGATGACGTCTCTGAGGTGGA
CGCGCGGCACATCATTGAGAATGCCAAGCAAGATGTCGATGATGAATATGGCGTGTCCCAGGCCCTTGCA

CGTGGCCTGCAGTCCTACTATGCCGTGGCCCATGCTGTCACTGAGAGAGTGGACAAGCAGTCAGCGCTTA
TGGTCAATGGTGTCCTCAAACAGTACCAGATCAAAGGTTTGGAGTGGCTGGTGTCCCTGTACAACAACAA
CCTGAACGGCATCCTGGCCGACGAGATGGGCCTGGGGAAGACCATCCAGACCATCGCGCTCATCACGTAC
CTCATGGAGCACAAACGCATCAATGGGCCCTTCCTCATCATCGTGCCTCTCTCAACGCTGTCCAACTGGG
CGTACGAGTTTGACAAGTGGGCCCCCTCCGTGGTGAAGGTGTCTTACAAGGGATCCCCAGCAGCAAGACG
GGCCTTTGTCCCCCAGCTCCGGAGTGGGAAGTTCAACGTCTTGCTGACGACGTACGAGTACATCATCAAA
GACAAGCACATCCTCGCCAAGATCCGTTGGAAGTACATGATTGTGGACGAAGGTCACCGCATGAAGAACC
ACCACTGCAAGCTGACGCAGGTGCTCAACACGCACTATGTGGCACCCCGCCGCCTGCTGCTGACGGGCAC
ACCGCTGCAGAACAAGCTTCCCGAGCTCTGGGCGCTGCTCAACTTCCTGCTGCCCACCATCTTCAAGAGC
TGCAGCACCTTCGAGCAGTGGTTTAACGCACCCTTTGCCATGACCGGGGAAAAGGTGGACCTGAATGAGG
AGGAAACCATTCTCATCATCCGGCGTCTCCACAAAGTGCTGCGGCCCTTCTTGCTCCGACGACTCAAGAA
GGAAGTCGAGGCCCAGTTGCCCGAAAAGGTGGAGTACGTCATCAAGTGCGACATGTCTGCGCTGCAGCGA
GTGCTCTACCGCCACATGCAGGCCAAGGGCGTGCTGCTGACTGATGGCTCCGAGAAGGACAAGAAGGGCA
AAGGCGGCACCAAGACCCTGATGAACACCATCATGCAGCTGCGGAAGATCTGCAACCACCCCTACATGTT
CCAGCACATCGAGGAGTCCTTTTCCGAGCACTTGGGGTTCACTGGCGGCATTGTCCAAGGGCTGGACCTG
TACCGAGCCTCGGGTAAATTTGAGCTTCTTGATAGAATTCTTCCCAAACTCCGAGCAACCAACCACAAAG
TGCTGCTGTTCTGCCAAATGACCTCCCTCATGACCATCATGGAAGATTACTTTGCGTATCGCGGCTTTAA
ATACCTCAGGCTTGATGGAACCACGAAGGCGGAGGACCGGGGCATGCTGCTGAAAACCTTCAACGAGCCC
GGCTCTGAGTACTTCATCTTCCTGCTCAGCACCCGGGCTGGGGGGCTCGGCCTGAACCTCCAGTCGGCAG
ACACTGTGATCATTTTTGACAGCGACTGGAATCCTCACCAGGACCTGCAAGCGCAGGACCGAGCCCACCG
CATCGGGCAGCAGAACGAGGTGCGTGTGCTCCGCCTCTGCACCGTCAACAGCGTGGAGGAGAAGATCCTA
GCTGCAGCCAAGTACAAGCTCAACGTGGACCAGAAGGTGATCCAGGCCGGCATGTTCGACCAGAAGTCCT
CCAGCCATGAGCGGCGCGCCTTCCTGCAGGCCATCCTGGAGCACGAGGAGCAGGATGAGAGCAGACACTG
CAGCACGGGCAGCGGCAGTGCCAGCTTCGCCCACACTGCCCCTCCGCCAGCGGGCGTCAACCCCGACTTG
GAGGAGCCACCTCTAAAGGAGGAAGACGAGGTGCCCGACGACGAGACCGTCAACCAGATGATCGCCCGGC
ACGAGGAGGAGTTTGATCTGTTCATGCGCATGGACCTGGACCGCAGGCGCGAGGAGGCCCGCAACCCCAA
GCGGAAGCCGCGCCTCATGGAGGAGGACGAGCTCCCCTCGTGGATCATCAAGGACGACGCGGAGGTGGAG
CGGCTGACCTGTGAGGAGGAGGAGGAGAAGATGTTCGGCCGTGGCTCCCGCCACCGCAAGGAGGTGGACT
ACAGCGACTCACTGACGGAGAAGCAGTGGCTCAAGGCCATCGAGGAGGGCACGCTGGAGGAGATCGAAGA
GGAGGTCCGGCAGAAGAAATCATCACGGAAGCGCAAGCGAGACAGCGACGCCGGCTCCTCCACCCCGACC
ACCAGCACCCGCAGCCGCGACAAGGACGACGAGAGCAAGAAGCAGAAGAAGCGCGGGCGGCCGCCTGCCG
AGAAACTCTCCCCTAACCCACCCAACCTCACCAAGAAGATGAAGAAGATTGTGGATGCCGTGATCAAGTA
CAAGGACAGCAGCAGTGGACGTCAGCTCAGCGAGGTCTTCATCCAGCTGCCCTCGCGAAAGGAGCTGCCC
GAGTACTACGAGCTCATCCGCAAGCCCGTGGACTTCAAGAAGATAAAGGAGCGCATTCGCAACCACAAGT
ACCGCAGCCTCAACGACCTAGAGAAGGACGT**CATGCTCCTGTGCCAGAACGC**ACAGACCTTCAACCTGGA
GGGCTCCCTGATCTATGAAGACTCCATCGTCTTGCAGTCGGTCTTCACCAGCGTGCGGCAGAAAATCGAG
AAGGAGGATGACAGTGAAGGCGAGGAGAGTGAGGAGGAGGAAGAGGGC**G**AGGAGGAAGGCTCCGAATCCG
AATCTCGGTCCGTCAAAGTGAAGATCAAGCTTGGC**C**GGAAGGAGAAGGCACAGGACCGGC**T**GAAGGGCGG
CCGGCGGCGGCCGAGCCGAGGGTCC**C**GAGCCAAGCCGGTCGTGAGTGACGATGACAGTGAGGAGGAACAA
GAGGAGGACCGCTCAGGAAGTGGCAGCGAAGAAGACTGAGCCCCGACATTCCAGTCTCGACCCCGAGCCC
CTCGTTCCAGAGCTGAGATGGCATAGGCCTTAGCAGTAACGGGTAGCAGCAGATGTAGTTTCAGACTTGG
AGTAAAACTGTATAAACAAAAGAATCTTCCATATTTATACAGCAGAGAAGCTGTAGGACTGTTTGTGACT
GGCCCTGTCCTGGCATCAGTAGCATCTGTAACAGCATTAACTGTCTTAAAGAGAGAGAGAGAGAATTCCG
AAAAAAAAAAAAAAA

41) Entrez Gene ID 6794 = STK11, serine/threonine kinase 11 = BC007981

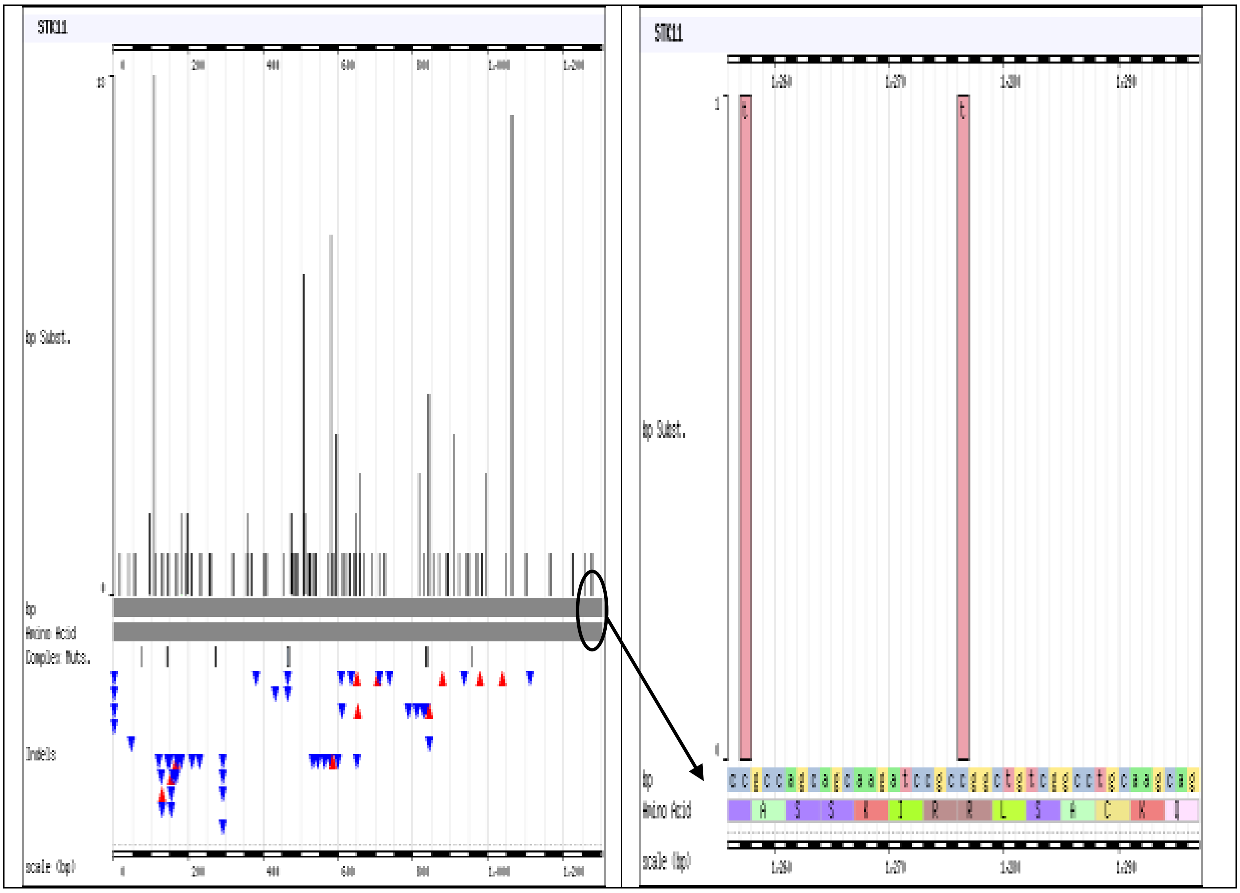

>gi|33872385|gb|BC007981.2| Homo sapiens serine/threonine kinase 11, mRNA (cDNA clone MGC:16254 IMAGE:3689780), complete cds
GGCCTGTGGGATGGGCGGCCCGGAGAAGACTGCGCTCGGCCGTGTTCATACTTGTCCGTGGGCCTGAGGT
CCCCGGAGGATGACCTAGCACTGAAAAGCCCCGGCCGGCCTCCCCAGGGTCCCCGAGGACGAAGTTGACC
CTGACCGGGCCGTCTCCCAGTTCTGAGGCCCGGGTCCCACTGGAACTCGCGTCTGAGCCGCCGTCCCGGA
CCCCCGGTGCCCGCCGGTCCGCAGACCCTGCACCGGGCTTGGACTCGCAGCCGGGACTGACGTGTAGAAC
AATCGTTTCTGTTGGAAGAAGGGTTTTTCCCTTCCTTTTGGGGTTTTTGTTGCCTTTTTTTTTTCTTTTT
TCTTTGTAAAATTTTGGAGAAGGGAAGTCGGAACACAAGGAAGGACCGCTCACCCGCGGACTCAGGGCTG
GCGGCGGGACTCCAGGACCCTGGGTCCAGCATGGAGGTGGTGGACCCGCAGCAGCTGGGCATGTTCACGG
AGGGCGAGCTGATGTCGGTGGGTATGGACACGTTCATCCACCGCATCGACTCCACCGAGGTCATCTACCA
GCCGCGCCGCAAGCGGGCCAAGCTCATCGGCAAGTACCTGATGGGGGACCTGCTGGGGGAAGGCTCTTAC
GGCAAGGTGAAGGAGGTGCTGGACTCGGAGACGCTGTGCAGGAGGGCCGTCAAGATCCTCAAGAAGAAGA
AGTTGCGAAGGATCCCCAACGGGGAGGCCAACGTGAAGAAGGAAATTCAACTACTGAGGAGGTTACGGCA
CAAAAATGTCATCCAGCTGGTGGATGTGTTATACAACGAAGAGAAGCAGAAAATGTATATGGTGATGGAG
TACTGCGTGTGTGGCATGCAGGAAATGCTGGACAGCGTGCCGGAGAAGCGTTTCCCAGTGTGCCAGGCCC
ACGGGTACTTCTGTCAGCTGATTGACGGCCTGGAGTACCTGCATAGCCAGGGCATTGTGCACAAGGACAT
CAAGCCGGGGAACCTGCTGCTCACCACCGGTGGCACCCTCAAAATCTCCGACCTGGGCGTGGCCGAGGCA
CTGCACCCGTTCGCGGCGGACGACACCTGCCGGACCAGCCAGGGCTCCCCGGCTTTCCAGCCGCCCGAGA
TTGCCAACGGCCTGGACACCTTCTCCGGCTTCAAGGTGGACATCTGGTCGGCTGGGGTCACCCTCTACAA
CATCACCACGGGTCTGTACCCCTTCGAAGGGGACAACATCTACAAGTTGTTTGAGAACATCGGGAAGGGG
AGCTACGCCATCCCGGGCGACTGTGGCCCCCCGCTCTCTGACCTGCTGAAAGGGATGCTTGAGTACGAAC
CGGCCAAGAGGTTCTCCATCCGGCAGATCCGGCAGCACAGCTGGTTCCGGAAGAAACATCCTCCGGCTGA
AGCACCAGTGCCCATCCCACCGAGCCCAGACACCAAGGACCGGTGGCGCAGCATGACTGTGGTGCCGTAC
TTGGAGGACCTGCACGGCGCGGACGAGGACGAGGACCTCTTCGACATCGAGGATGACATCATCTACACTC
AGGACTTCACGGTGCCCGGACAGGTCCCAGAAGAGGAGGCCAGTCACAATGGACAGCGCCGGGGCCTCCC
CAAGGCCGTGTGTATGAACGGCACAGAGGCGGCGCAGCTGAGCACCAAATCCAGGGCGGAGGGCCGGGCC
CCCAACCCTGCCCGCAAGGCCTGCTC**C**GCCAGCAGCAAGATCCGC**C**GGCTGTCGGCCTGCAAGCAGCAGT

GAGGCTGGCCGCCTGCAGCCCGTGTCCAGGAGCCCCGCCAGGTGCCCGCGCCAGGCCCTCAGTCTTCCTG
CCGGTTCCGCCCGCCCTCCCGGAGAGGTGGCCGCCATGCTTCTGTGCCGACCACGCCCCAGGACCTCCGG
AGCGCCCTGCAGGGCCGGGCAGGGGGACAGCAGGGACCGGGCGCAGCCCTCCCCCCTCGGCCGCCCGGCA
GTGCACGCGGCTTGTTGACTTCGCAGCCCCGGGCGGAGCCTTCCCGGGCGGGCGTGGGAGGAGGGAGGCG
GCCTCCATGCACTTTATGTGGAGACTACTGGCCCCGCCCGTGGCCTCGTGCTCCGCAGGGCGCCCAGCGC
CGTCCGGCGGCCCCGCCGCAGACCAGCTGGCGGGTGTGGAGACCAGGCTCCTGACCCCGC**CATGCATGCA**
GCGCCACCTGGAAGCCGCGCGGCCGCTTTGGTTTTTTGTTTGGTTGGTTCCATTTTCTTTTTTTCTTTTT
TTTTTTAAGAAAAAATAAAAGGTGGATTTGAAAAAAAAAAAAAAAAAAAAA

42) Entrez Gene ID 6938 = TCF12, transcription factor 12 = AL831981

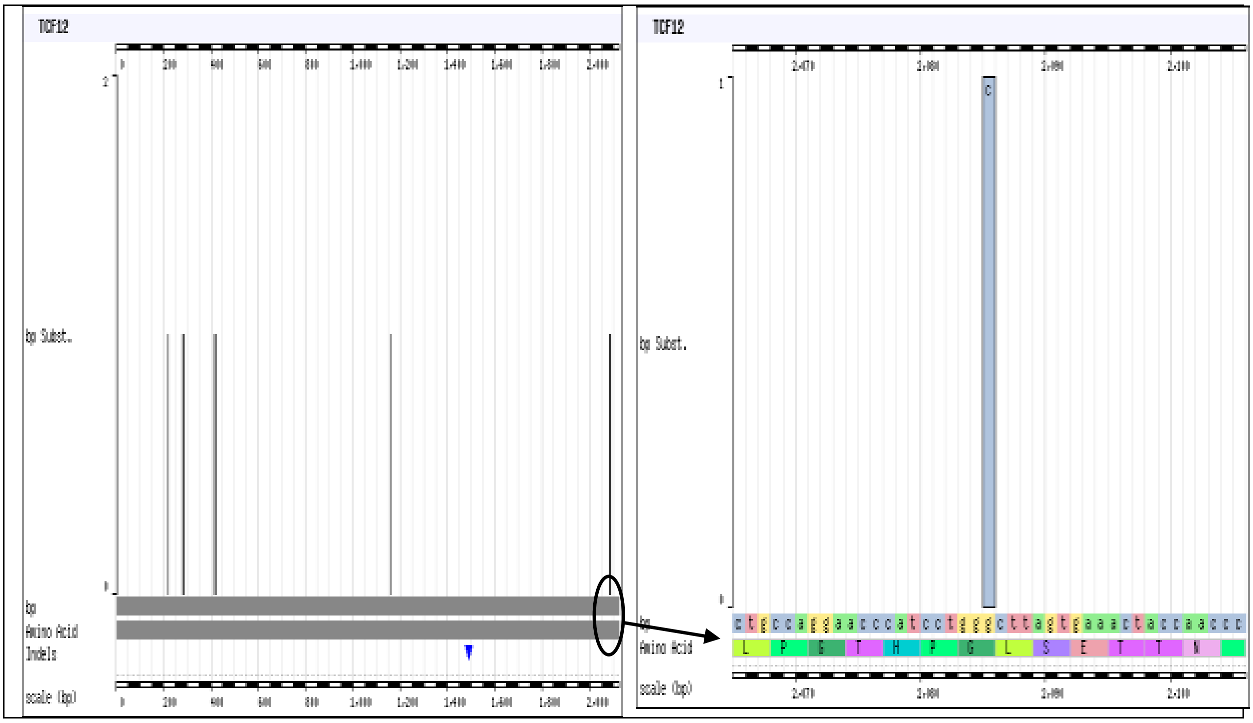

>gi|30268205|emb|AL831981.2| Homo sapiens mRNA; cDNA DKFZp451F163 (from clone DKFZp451F163); complete cds
GCCTCAGCGAAAAAAATGTCCGCCTGAAGAGACCCACAAGTTCTATTCGGGGGGACCGACAGCCCGCCCC
GGGAGGAAGGGGCGGCCAGGCCCGAAAGCCGCCTCCCCCTCCCAGACCCGAGAGCTCGTGCGGGGCAAAG
TGAACCGAGCCGCTGGGCGGTGCAAGGGGAAGCCCAAGCCCGTTCTCCCGGCCAAAGTGAACTTTAATCG
GGGTGGTTGGATGCGGAGACGGGGCGGCAGGACCTGCTAGAAGTGGCCGAAGATGAATCCCCAGCAACAA
CGCATGGCCGCTATAGGGACCGACAAGGAGCTGAGCGACCTACTGGACTTCAGTGCGATGTTTTCCCCAC
CTGTTAATAGTGGGAAAACTAGACCAACTACACTGGGAAGCAGTCAATTCAGTGGATCAGGTATTGATGA
AAGAGGAGGTACAACATCTTGGGGAACAAGTGGTCAACCAAGTCCTTCCTATGATTCATCTAGAGGTTTT
ACAGACAGCCCTCATTACAGTGATCACTTGAATGACAGTCGATTAGGAGCCCATGAAGGCTTGTCCCCAA
CACCTTTCATGAACTCAAATCTGATGGGAAAAACATCAGAGAGAGGCTCATTTTCCCTGTACAGCAGAGA
TACTGGATTACCAGGCTGTCAATCTAGTCTCCTGAGACAAGATCTGGGGCTTGGGAGCCCAGCACAGCTA
TCTTCTTCAGGAAAACCTGGGACAGCATACTATTCATTCTCTGCTACAAGTTCCAGGAGGAGACCACTCC
ATGACTCTGCAGCGCTTGATCCCTTGCAAGCAAAAAAAGTCAGAAAGGTGCCTCCTGGTTTGCCTTCTTC
TGTATATGCACCATCCCCAAATTCAGATGATTTCAACCGTGAATCTCCTAGTTATCCATCTCCTAAGCCA
CCAACCAGTATGTTCGCTAGCACTTTCTTTATGCAAGATGGGACCCACAATTCTTCTGACCTTTGGAGTT
CATCAAATGGGATGAGCCAGCCTGGTTTTGGTGGAATTCTGGGGACCTCCACTTCCCACATGTCTCAATC
CAGTAGTTATGGCAACCTTCATTCACATGACCGCTTGAGTTATCCTCCACACTCAGTTTCACCAACAGAC
ATAAACACGAGTCTTCCACCAATGTCCAGCTTTCATCGCGGCAGTACCAGCAGTTCACCTTACGTTGCTG
CCTCACACACTCCTCCCATCAATGGATCAGACAGCATTCTAGGAACCAGAGGGAATGCTGCTGGAAGCTC
ACAGACAGGTGATGCACTTGGAAAGGCTTTGGCATCTATTTATTCTCCTGACCATACCAGCAGTAGTTTT
CCGTCAAATCCATCAACACCAGTTGGATCACCTTCACCTCTCACAGGTACCAGTCAGTGGCCAAGACCTG
GAGGGCAAGCACCTTCATCCCCAAGCTATGAAAACTCACTCCACTCCCTGCAGTCTCGAATGGAGGATCG

TTTAGACAGACTGGATGATGCAATCCATGTGCTGCGGAACCATGCTGTGGGACCTTCCACCAGTTTGCCT
GCTGGTCACAGTGATATACATAGTTTATTGGGACCATCCCATAATGCACCAATTGGAAGCCTCAATTCAA
ACTATGGAGGATCAAGCCTTGTTGCAAGCAGTCGATCAGCTTCAATGGTTGGAACTCATCGGGAAGACTC
TGTCAGTCTCAATGGCAATCATTCAGTCCTGTCTAGTACAGTCACTACTTCAAGCACAGACCTGAACCAT
AAAACACAAGAAAATTATAGAGGTGGCTTGCAAAGTCAGTCTGGAACTGTTGTTACAACAGAAATCAAGA
CTGAAAACGAAGAAAAGGATGAAAACCTTCATGAACCTCCTTCATCAGATGACATGAAGTCAGATGATGA
ATCCTCCCAAAAAGATATCAAGGTTTCATCTAGAGGCAGAACAAGCAGTACTAATGAAGATGAGGATTTG
AACCCTGAACAGAAGATAGAAAGGGAGAAGGAGAGGCGGATGGCTAACAATGCCAGAGAACGCTTACGCG
TGCGGGATATTAATGAAGCATTCAAAGAGCTTGGCCGAATGTGTCAGCTTCACTTGAAGAGTGAAAAACC
CCAAACAAAACTCCTTATTCTTCATCAAGCCGTGGCAGTCATCCTTAGTCTAGAACAGCAAGTCAGAGAG
AGGAACCTTAACCCCAAAGCAGCCTGCCTTAAGAGAAGGGAAGAAGAAAAAGTTTCTGCCGTATCGGCAG
AGCCGCCAACCACACTGCCAGGAACCCATCCTGG**G**CTTAGTGAAACTACCAACCCTATGGGTCATATGTA
AACATCAGCCAGTTCCAGAGTTATCAGTAGGCTAGATAGAAGGTGACCTCTCCTCATAAGGACTTGGACA
ACTCAGATTATCTGAAGACACAAACCTGACAGGAGGGAGAAGAAAAAACAAAACACTTGAACCAAGAAAC
TCAAATGTAATCCTACGATCAAAGCAACTGGTCAACACTTCCATCAGAAGTGAAGATAGGAAGCTCATCA
GATAGAACATCAGCCCATGAGATGTTTGCAACAAATCTTTTGTTGCAAGCAGTGTGTCGCTTCTGCACAA
TCAGAGACTGTCTCGATCTCTCCACTCACCGTGGAAGTCGCCTTGTGCCTAAACTGAATTGACAAATGCA
TTGTAACTACAAATTTTATTTATTGTTATGAAACTGTAAGGTCTACATATAAAGGGAAAAAGTTAATGTG
GAAAGCTGATCTACACTCAGCTGATGCCAGCATACATTAAAGCGGTTCACGTGCAGAGAACAAAGCAGTG
ACAACCATTGGCCCTTAGCATTCCCGGCATACCTATTAGTGTCTTAAAAAGGAAGGGAAAAGTCTTTTGT
TGCCCTCTCCTATCCTCTTGCCATATGAATAGCGTTTTCCATGAAATAGGAAAATATTACTTGGTATAGC
ATTTCTCTTGCTCTCATTTTTTGATTTATTTTTATTTTCTCTTTGTGGGTGTTATATTTGATCTCTAAAT
CTGAACAGTTTATGGTCACAGTCCAGCCTCCTCCGTGCAGCCCTGTGTGCTTTGCACATTTACCTTACAG
TGGTAAGCAGAGACCATCTGTGACCATAGCCTAGCTAGCATTTTAAAAGGGGAAATTTTGTTCTCTAGGT
TTTCCCCCAAATAAACATTGCTTTATTTCTAATAATAACCAAGACTTTTCAAGCTTCTAGATCTCATAGG
AAAGCTTGTAATAGCAAAATTGTAAATTACAAGGGAAGAATCTACTTTTTAGAAATCGCTTTGTTTTCCA
AGCAGTAAGTACTACATACAGTACTTGTAAAGTGTTAGCTGTAAGTAAGCACAAAATACATTTAAAATAC
AAAGACGATTTTTTCAGGCTGTGATTATGGTGAACATAACAAAACCCAGTAGTCACCAAGGCAGGTAGTG
TGATAAATGAACACACCACTCTGAGGCTAATTACCTAATGGAATACAAGAGCAATGGTCACCCGTATTTC
CTTATCCTAGCCTTTATTTCTCTGTCATTTGGATGGCTGGTCAATGGGGAAGAATTGAGTGGGTGATTTA
ATCAACTGCAAACCATCTGCCCCTGTCCCAAAATGATGAGCCAGATTAGCATTAAACCAGTACTTGTCAG
TCCATCTTAATACTGTTCATTAAGGCACTCTCTGTCTCTAATCCTTAGGAGTTGTTTTAAAAGACATAAT
CACTTTGAACTTCCATGAAACCTGTCTTCCACCACAACAACCCTGGGAGAGAAAAA**CATGCTAAAGGAGG**
TATCTTGGCTTAATAATTCCTTATAGCCAATATCAACAGTGGCAATCAGCACACAGAGGAAAGGACCCAA
ATCACTATGTAGCTTAAAGATTTCTGTTAATTTGAAAGAACAAAAACAAGACAGAACTTCTGGTACTCTA
ATCAGGATGATTCCTAACAAGTCAGTCATTTGTGAACTTAGTGGACTTTTTGGTTACTTTAATTTGCATA
TATTCTCCAGTTACATCGGACTCTATCTGTGGCCTTGTTCTTCATTTCAGTGTTAATCAGCTAAACAGAA
GTTGTTGCTTATGATGTGTGAGTGAACATATGCCACTGCCTGGCCTTTTTTTCTTCAGAGCTTGTTGTCT
TTTTCGCTATATTAGACTTTGCAGTATGCCCAGAAGCTTTCCTTCATAAAATAGAAAGAAAAAAACATTT
GGCTTATTTTTCACTGTAGCTAGTCTTTTATACAATAATCTTGTAAGAAAATTTCTTGAATTCTAAATAT
TACTCTTTCTAGATTTTTGAAATCAAAAAGTTTTCAGTAAAAAGTTTCTTACTTTATTTTATTATATTAG
GTAGTAAAAAATGTAGGGTTATTTACCATAACCTGTTCATTAATATCAGAAATTTACAATAGCATTTTAA
GACCATAGTAGGATTCTAGCATACCGTGTAGTACCTATGGAGTATTGTAAGAGCTAATTGTTGGAGATGA
ATTGCTTCTCATCTTGTTCTCCAGTTTCCATTGTTGGTTTATTGCAGATTTGTATCCTGTGTCAAATTCA
AGGTATTATTGATAAACCTTTTCAACCAGCAGCAAGAAGTTCAAATTTTTTTCTGTCACTGTAACAGAAA
ACACAATATGTATATAACATTTATGTAGCAATAAATGTGCCATCTTTTTTTTAACACAGCAAAAAAAAAA
AAAAAAAAAAAA

43) Entrez Gene ID 7175 = TPR, translocated promoter region (to activated MET oncogene) = U69668

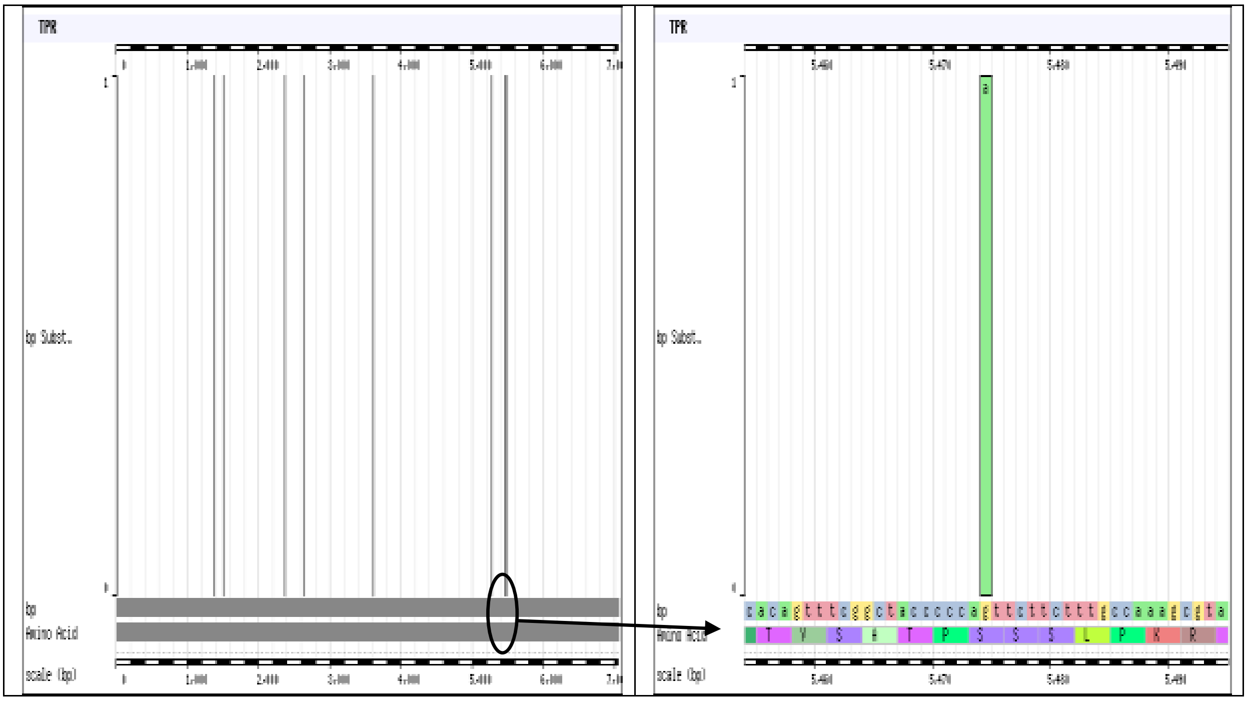

>gi|1850341|gb|U69668.1|HSU69668 Human nuclear pore complex-associated protein TPR (tpr) mRNA, complete cds
CCAGGCGTCTGGGTCCTGCTGGTCTTCGCCTTTCTTCTCCGCTTCTACCCCGTCGGCCGCTGCCACTGGG
GTCCCTGGCCCCACCGACATGGCGGCGGTGTTGCAGCAAGTCCTGGAGCGCACGGAGCTGAACAAGCTGC
CCAAGTCTGTCCAGAACAAACTTGAAAAGTTCCTTGCTGATCAGCAATCCGAGATCGATGGCCTGAAGGG
GCGGCATGAGAAATTTAAGGTGGAGAGCGAACAACAGTATTTTGAAATAGAAAAGAGGTTGTCCCACAGT
CAGGAGAGACTTGTGAATGAAACCCGAGAGTGTCAAAGCTTGCGGCTTGAGCTAGAGAAACTCAACAATC
AACTGAAGGCACTAACTGAGAAAAACAAAGAACTTGAAATTGCTCAGGATCGCAATATTGCCATTCAGAG
CCAATTTACAAGAACAAAGGAAGAATTAGAAGCTGAGAAAAGAGACTTAATTAGAACCAATGAGAGACTA
TCTCAAGAACTTGAATACTTAACAGAGGATGTTAAACGTCTGAATGAAAAACTTAAAGAAAGCAATACAA
CAAAGGGTGAACTTCAGTTAAAATTGGATGAACTTCAAGCTTCTGATGTTTCTGTTAAGTATCGAGAAAA
ACGCTTGGAGCAAGAAAAGGAATTGCTACATAGTCAGAATACATGGCTGAATACAGAGTTGAAAACCAAA
ACTGATGAACTTCTGGCTCTTGGAAGAGAAAAAGGGAATGAGATTCTAGAGCTTAAATGTAATCTTGAAA
ATAAAAAAGAAGAGGTTTCTAGACTGGAAGAACAAATGAATGGCTTAAAAACATCAAATGAACATCTTCA
AAAGCATGTGGAGGATCTGTTGACCAAATTAAAAGAGGCCAAGGAACAACAGGCCAGTATGGAAGAGAAA
TTCCACAATGAATTAAATGCCCACATAAAACTTTCTAATTTGTACAAGAGTGCCGCTGATGACTCAGAAG
CAAAGAGCAATGAACTAACCCGGGCAGTAGAGGAACTACACAAACTTTTGAAAGAAGCTGGTGAAGCCAA
CAAAGCAATACAAGATCATCTTCTAGAGGTGGAGCAATCCAAAGATCAAATGGAAAAAGAAATGCTTGAG
AAAATAGGGAGATTGGAGAAGGAATTAGAGAATGCAAATGACCTTCTTTCTGCCACAAAACGTAAAGGAG
CCATATTGTCTGAAGAAGAGCTTGCCGCCATGTCTCCTACTGCAGCAGCTGTAGCTAAGATAGTGAAACC
TGGGATGAAACTAACTGAGCTCTATAATGCTTATGTGGAAACTCAGGATCAGTTGCTTTTGGAGAAACTA
GAGAACAAAAGAATTAATAAGTACCTAGATGAAATAGTGAAAGAAGTGGAAGCCAAAGCACCAATTTTGA
AACGCCAGCGTGAGGAATATGAACGTGCACAGAAAGCTGTAGCAAGTTTATCTGTTAAGCTTGAACAAGC
TATGAAGGAGATTCAGCGATTGCAGGAGGACACTGATAAAGCCAACAAGCAATCATCTGTACTTGAGAGA
GATAATCGAAGAATGGAAATACAAGTAAAAGATCTTTCACAACAGATTAGAGTGCTTTTGATGGAACTTG
AAGAAGCAAGGGGTAACCACGTAATTCGTGATGAGGAAGTAAGCTCTGCTGATATAAGTAGTTCATCTGA
GGTAATATCACAGCATCTAGTATCTTACAGAAATATTGAAGAGCTTCAACAACAAAATCAACGTCTCTTA
GTGGCCCTTAGAGAGCTTGGGGAAACCAGAGAAAGAGAAGAACAAGAAACAACTTCATCCAAAATCACTG
AGCTTCAGCTCAAACTTGAGAGTGCCCTTACTGAACTAGAACAACTCCGCAAATCACGACAGCATCAAAT
GCAGCTTGTTGATTCCATAGTTCGTCAGCGTGATATGTACCGTATTTTATTGTCACAAACAACAGGAGTT
GCCATTCCATTACATGCTTCAAGCTTAGATGATGTTTCTCTTGCATCAACTCCAAAACGTCCAAGTACAT
CACAGACTGTTTCCACTCCTGCTCCAGTACCTGTTATTGAATCAACAGAGGCTATAGAGGCTAAGGCTGC
CCTTAAACAGTTGCAGGAAATTTTTGAGAACTACAAAAAAGAAAAAGCAGAAAATGAAAAAATACAAAAT
GAGCAGCTTGAGAAACTTCAAGAACAAGTTACAGATTTGCGATCACAAAATACCAAAATTTCTACCCAGC

TAGATTTTGCTTCTAAACGTTATGAAATGCTGCAAGATAATGTTGAAGGATATCGTCGAGAAATAACATC
ACTTCATGAGAGAAATCAGAAACTCACTGCCACAACTCAAAAGCAAGAACAGATTATCAATACGATGACT
CAAGATTTGAGAGGAGCAAATGAGAAGCTAGCTGTCGCAGAAATAAGAGCAGAAAATTTGAAGAAGGAAA
AGGAAATGCTTAAATTGTCTGAAGTTCGTCTTTCTCAGCAAAGAGAGTCTTTGTTAGCTGAACAAAGGGG
GCAAAACTTACTGCTAACTAATCTGCAAACAATTCAGGGAATACTGGAGCGATCTGAAACAGAAACCAAA
CAAAGGCTTAGTAGCCAGATAGAAAAACTGGAACATGAGATCTCTCATCTAAAGAAGAAGTTGGAAAATG
AGGTGGAACAAAGGCATACACTTACTAGAAATCTAGATGTTCAACTTTTAGATACAAAGAGACAACTGGA
TACAGAGACAAATCTTCATCTTAACACAAAAGAACTATTAAAAAATGCTCAAAAAGAAATTGCCACATTG
AAACGGCACCTCAGTAATATGGAAGTCCAAGTTGCTTCTCAGTCTTCACAGAGAACTGGTAAAGGTCAGC
CTAGCAACAAAGAAGATGTGGATGATCTTGTGAGTCAGCTAAGACAGACAGAAGAGCAGGTGAATGACTT
AAAGGAGAGACTCAAAACAAGTACGAGCAATGTGGAACAATATCAAGCAATGGTTACTAGTTTAGAAGAA
TCCCTGAACAAGGAAAAACAGGTGACAGAAGAAGTGCGTAAGAATATTGAAGTTCGTTTAAAAGAGTCAG
CTGAATTTCAGACACAGTTGGAAAAGAAGTTGATGGAAGTAGAGAAGGAAAAACAAGAACTTCAGGATGA
TAAAAGAAGAGCCATAGAGAGCATGGAACAACAGTTATCTGAATTGAAGAAAACACTTTCTAGTGTTCAG
AATGAAGTACAAGAAGCTCTTCAGAGAGCAAGCACAGCTTTAAGTAATGAGCAGCAAGCCAGACGTGACT
GTCAGGAACAAGCTAAAATAGCTGTGGAAGCTCAGAATAAGTATGAGAGAGAATTGATGCTGCATGCTGC
TGATGTTGAAGCTCTACAAGCTGCGAAGGAGCAGGTTTCAAAAATGGCATCAGTCCGTCAGCATTTGGAA
GAAACAACACAGAAAGCAGAATCACAGTTGTTGGAGTGTAAAGCATCTTGGGAGGAAAGAGAGAGAATGT
TAAAGGATGAAGTTTCCAAATGTGTATGTCGCTGTGAAGATCTGGAGAAACAAAACAGATTACTTCATGA
TCAGATCGAAAAATTAAGTGACAAGGTCGTTGCCTCTGTGAAGGAAGGTGTACAAGGTCCACTGAATGTA
TCTCTCAGTGAAGAAGGAAAATCTCAAGAACAAATTTTGGAAATTCTCAGATTTATACGACGAGAAAAAG
AAATTGCTGAAACTAGGTTTGAGGTGGCTCAGGTTGAGAGTCTGCGTTATCGACAAAGGGTTGAACTTTT
AGAAAGAGAGCTGCAGGAACTGCAAGATAGTCTAAATGCTGAAAGGGAGAAAGTCCAGGTAACTGCAAAA
ACAATGGCTCAGCATGAAGAACTGATGAAGAAAACTGAAACAATGAATGTAGTTATGGAGACCAATAAAA
TGCTAAGAGAAGAGAAGGAGAGACTAGAACAGGATCTACAGCAAATGCAAGCAAAGGTGAGGAAACTGGA
GTTAGATATTTTACCCTTACAAGAAGCAAATGCTGAGCTGAGTGAGAAAAGCGGTATGTTGCAGGCAGAG
AAGAAGCTCTTAGAAGAGGATGTCAAACGTTGGAAAGCACGTAACCAGCATCTAGTAAGTCAACAGAAAG
ATCCAGATACAGAAGAATATCGGAAGCTCCTTTCTGAAAAGGAAGTTCATACTAAGCGTATTCAACAATT
GACAGAAGAAATTGGTAGACTTAAAGCTGAAATTGCAAGATCAAATGCATCTTTGACTAACAACCAGAAC
TTAATTCAGAGTCTGAAGGAAGATCTAAATAAAGTAAGAACTGAAAAGGAAACCATCCAGAAGGACTTAG
ATGCCAAAATAATTGATATCCAAGAAAAAGTCAAAACTATTACTCAAGTTAAGAAAATTGGACGTAGGTA
CAAGACTCAATATGAAGAACTTAAAGCACAACAGGATAAGGTTATGGAGACATCGGCTCAGTCCTCTGGA
GACCATCAGGAGCAGCATGTTTCAGTCCAGGAAATGCAGGAACTCAAAGAAACGCTCAACCAAGCTGAAA
CAAAATCAAAATCACTTGAAAGTCAAGTAGAGAATCTGCAGAAGACATTATCTGAAAAAGAGACAGAAGC
AAGAAATCTCCAGGAACAGACTGTGCAACTTCAGTCTGAACTTTCACGACTTCGTCAGGATCTTCAAGAT
AGAACCACACAGGAGGAGCAGCTCCGACAACAGATAACTGAAAAGGAAGAAAAAACCAGAAAGGCTATTG
TAGCAGCAAAGTCAAAAATTGCACACTTAGCTGGTGTAAAAGATCAGCTAACTAAAGAAAATGAGGAGCT
TAAACAAAGGAATGGAGCCTTAGATCAGCAGAAAGATGAATTGGATGTTCGCATTACTGCGCTAAAGTCC
CAATATGAAGGTCGAATTAGTCGCTTGGAAAGAGAACTCAGGGAGCATCAAGAGAGACACCTTGAGCAGA
GAGATGAGCCTCAAGAACCTTCTAATAAGGTCCCTGAACAGCAGAGACAGATCACATTGAAAACAACTCC
AGCTTCTGGTGAAAGAGGAATTGCCAGCACATCAGACCCACCAACAGCCAATATCAAGCCAACTCCTGTT
GTGTCTACTCCAAGTAAAGTGACAGCTGCAGCTATGGCTGGAAATAAGTCAACACCCAGGGCTAGTATCC
GCCCAATGGTTACACCTGCAACTGTTACAAATCCCACTACTACCCCAACAGCTACAGTGATGCCCACTAC
ACAAGTGGAATCACAGGAAGCTATGCAGTCAGAAGGGCCTGTGGAACATGTTCCAGTTTTTGGAAGCACA
AGTGGATCCGTTCGTTCTACTAGTCCTAATGTCCAGCCTTCTATCTCTCAACCTATTTTAACTGTTCAGC
AACAAACACAGGCTACAGCTTTTGTGCAACCCACTCAACAGAGTCATCCTCAGATTGAGCCTGCCAATCA
AGAGTTATCTTCAAACATAGTAGAGGTTGTTCAGAGTTCACCAGTTGAGCGGCCTTCTACTTCCACAGCA
GTATTTGGCACAGTTTCGGCTACCCCCA**G**TTCTTCTTTGCCAAAGCGTACACGTGAAGAGGAAGAGGATA
GCACCATAGAAGCATCAGACCAAGTCTCTGATGATACAGTGGAAATGCCTCTTCCAAAGAAGTTGAAAAG
TGTCACACCTGTGGGAACTGAGGAAGAAGTTATGGCAGAAGAAAGTACTGATGGAGAGGTAGAGACTCAG
GTATACAACCAGGATTCTCAAGATTCCATTGGAGAAGGAGTTACCCAGGGAGATTATACACCTATGGAAG
ACAGTGAAGAAACCTCTCAGTCTCTACAAATAGATCTTGGGCCACTTCAATCAGATCAGCAGACGACAAC
TTCATCCCAGGATGGTCAAGGCAAAGGAGATGATGTCATTGTAATTGACAGTGATGATGAAGAAGAGGAT
GATGATGAAAATGATGGAGAACATGAGGATTATGAAGAGGATGAGGAAGATGATGATGATGATGAAGATG
ACACAGGGATGGGAGATGAGGGTGAAGATAGTAATGAAGGAACTGGTAGTGCCGATGGCAATGATGGTTA
TGAAGCTGATGATGCTGAGGGTGGTGATGGGACTGATCCAGGTACAGAAACAGAAGAAAGTATGGGTGGA
GGTGAAGGTAATCACAGAGCTGCTGATTCTCAAAACAGTGGTGAAGGAAATACAGGTGCTGCAGAATCTT
CTTTTTCTCAGGAGGTTTCTAGAGAACAACAGCCATCATCAGCATCTGAAAGACAGGCCCCTCGAGCACC
TCAGTCACCGAGACGCCCACCACATCCACTTCCCCCAAGACTGACCATTCATGCCCCACCTCAGGAGTTG
GGACCACCAGTTCAGAGAATTCAGATGACCCGAAGGCAGTCTGTAGGACGTGGCCTTCAGTTGACTCCAG
GAATAGGTGGCATGCAACAGCATTTTTTTGATGATGAAGACAGAACAGTTCCAAGTACTCCAACTCTTGT

GGTGCCACATCGTACTGATGGATTTGCTGAAGCAATTCATTCGCCGCAGGTTGCTGGTGTCCCTAGATTC
CGGTTTGGGCCACCTGAAGATATGCCACAAACAAGTTCTAGTCACTCTGATCTTGGCCAGCTTGCTTCTC
AAGGAGGTTTAGGAATGTATGAAACACCCCTGTTCCTAGCTCATGAAGAAGAGTCAGGTGGCCGAAGTGT
TCCCACTACTCCACTACAAGTAGCAGCCCCAGTGACTGTATTTACTGAGAGCACCACCTCTGATGCTTCG
GAACATGCCTCTCAATCTGTTCCAATGGTGACTACATCCACTGGCACTTTATCTACAACAAATGAAACAG
CAACAGGTGATGATGGAGATGAAGTATTTGTGGAGGCAGAATCTGAAGGTATTAGTTCAGAAGCAGGCCT
AGAAATTGATAGCCAGCAGGAAGAAGAGCCGGTTCAAGCATCTGATGAGTCAGATCTCCCCTCCACCAGC
CAGGATCCTCCTTCTAGCTCATCTGTAGATACTAGTAGTAGTCAACCAAAGCCTTTCAGACGAGTAAGAC
TTCAGACAACATTGAGACAAGGTGTCCGTGGTCGTCAGTTTAACAGACAGAGAGGTGTGAGCCATGCAAT
GGGAGGGAGAGGAGGAATAAACAGAGGAAATATTAATTAAATGGTCTGTAAACAATAACAACTGTGAATA
AGATTATCAAATCTGTTTTAGTGTAATGATTGTCAAGTTTAAAAACATTTTTATATATAAACTGGTATAC
T**CATGTCAATATTCTTTATTAA**TAAAATGTTTTTCAGTGTCAAAAAAAAAAAAAAAAA

44) Entrez Gene ID 7403 = KDM6A, lysine (K)-specific demethylase 6A = AF000992

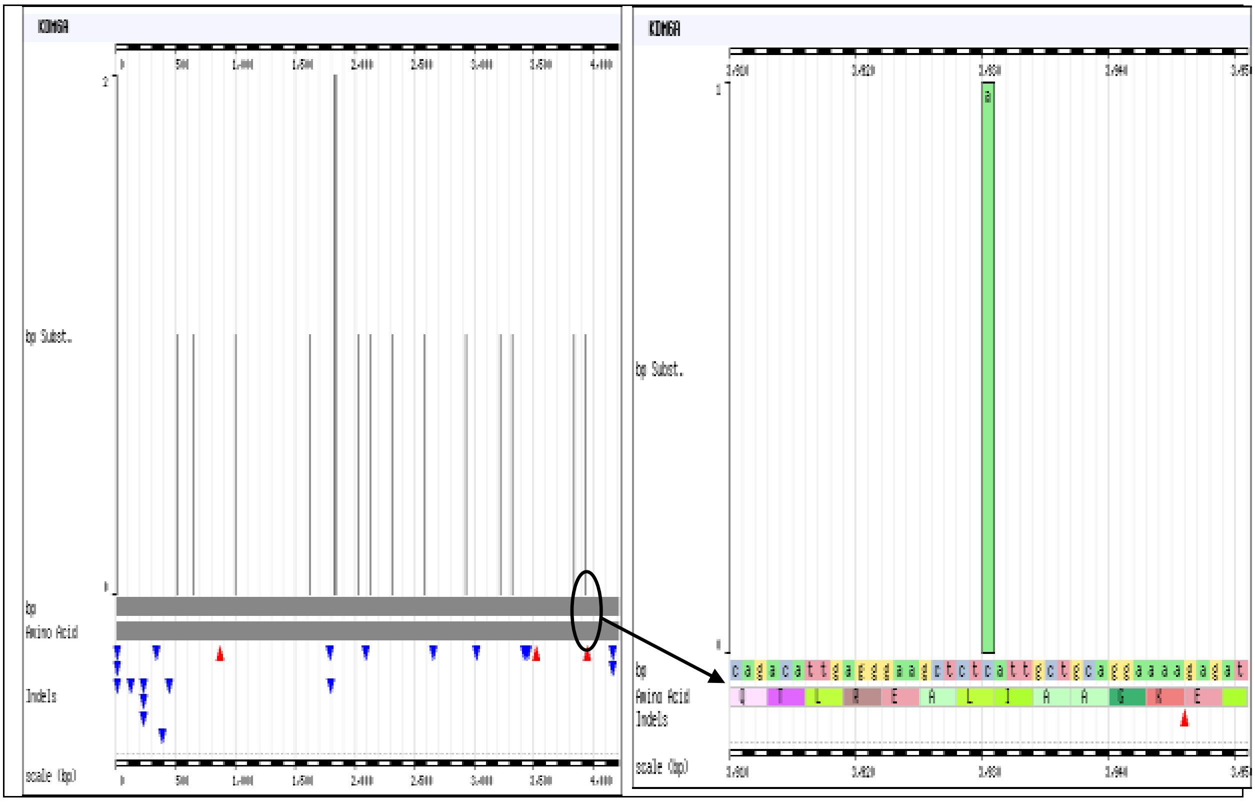

>gi|2580569|gb|AF000992.1|HSAF000992 Homo sapiens ubiquitous TPR motif, X isoform (UTX) mRNA, alternative transcript 1, complete cds
AAAGCAAAAGAATTCGCTGCGTTTCCATGAAATCCTGCGGAGTGTCGCTCGCTACCGCCGCCGCTGCCGC
CGCCGCTTTCGGTGATGAGGAAAAGAAAATGGCGGCGGGAAAAGCGAGCGGCGAGAGCGAGGAGGCGTCC
CCCAGCCTGACAGCCGAGGAGAGGGAGGCGCTCGGCGGACTGGACAGCCGCCTCTTTGGGTTCGTGAGAT
TTCATGAAGATGGCGCCAGGACGAAGGCCCTACTGGGCAAGGCTGTTCGCTGCTATGAATCTCTAATCTT
AAAAGCTGAAGGAAAAGTGGAGTCTGATTTCTTTTGTCAATTAGGTCACTTCAACCTCTTATTGGAAGAT
TATCCAAAAGCATTATCTGCATACCAGAGGTACTACAGTTTACAGTCTGACTACTGGAAGAATGCTGCCT
TTTTATATGGTCTTGGTTTGGTCTACTTCCATTATAATGCATTTCAGTGGGCAATTAAAGCATTTCAGGA
GGTGCTTTATGTTGATCCCAGCTTTTGTCGAGCCAAGGAAATTCATTTACGAGTTGGGCTTATGTTCAAA
GTGAACACAGACTATGAGTCTAGTTTAAAGCATTTTCAGTTAGCTTTGGTTGACTGTAATCCCTGCACTT
TGTCCAATGCTGAAATTCAATTTCACATTGCCCACTTATATGAAACCCAGAGGAAATATCATTCTGCAAA
AGAAGCTTATGAACAACTTTTGCAGACAGAGAATCTTTCTGCACAAGTAAAAGCAACTGTCTTACAACAG
TTAGGTTGGATGCATCACACTGTAGATCTCCTGGGAGATAAAGCCACCAAGGAAAGCTATGCTATTCAGT
ATCTCCAAAAGTCCTTGGAAGCAGATCCTAATTCTGGCCAGTCCTGGTATTTCCTCGGAAGGTGCTATTC
AAGTATTGGGAAAGTTCAGGATGCCTTTATATCTTACAGGCAGTCTATTGATAAATCAGAAGCAAGTGCA

GATACATGGTGTTCAATAGGTGTGCTATATCAGCAGCAAAATCAGCCCATGGATGCTTTACAGGCCTATA
TTTGTGCTGTACAATTGGACCATGGCCATGCTGCAGCCTGGATGGACCTAGGCACTCTCTATGAATCCTG
CAACCAGCCTCAGGATGCCATTAAATGCTACTTAAATGCAACTAGAAGCAAAAGTTGTAGTAATACCTCT
GCACTTGCAGCACGAATTAAGTATTTACAGGCTCAGTTGTGTAACCTTCCACAAGGTAGTCTACAGAATA
AAACTAAATTACTTCCTAGTATTGAGGAGGCGTGGAGCCTACCAATTCCCGCAGAGCTTACCTCCAGGCA
GGGTGCCATGAACACAGCACAGCAGAATACTTCTGACAATTGGAGTGGTGGACATGCTGTGTCACATCCT
CCAGTACAGCAACAAGCTCATTCATGGTGTTTGACACCACAGAAATTACAGCATTTGGAACAGCTCCGCG
CAAATAGAAATAATTTAAATCCAGCACAGAAACTGATGCTGGAACAGCTGGAAAGTCAGTTTGTCTTAAT
GCAACAACACCAAATGAGACCAACAGGAGTTGCACAGGTACGATCTACTGGAATTCCTAATGGGCCAACA
GCTGACTCATCACTGCCTACAAACTCAGTCTCTGGCCAGCAGCCACAGCTTGCTCTGACCAGAGTGCCTA
GCGTCTCTCAGCCTGGAGTCCGTCCTGCCTGCCCTGGGCAGCCTTTGGCCAATGGACCCTTTTCTGCAGG
CCATGTTCCCTGTAGCACATCAAGAACGCGGGGAAGTACAGACACTATTTTGATAGGCAATAATCATATA
ACAGGAAATGGAAGTAATGGAAACGTGCCTTACCTGCAGCGAAACGCACTCACTCTACCTCATAACCGCA
CAAACCTGACCAGCAGCGCAAAGGAGCCGTGGAAAAACCAACTATCTAACTCCACTCAGGGGCTTCACAA
AGGTCAGAGTTCACATTCGGCAGGTCCTAATGGTGAACGACCTCTCTCTTCCACTGGGCCTTCCCAGCAT
CTCCAGGCAGCTGGCTCTGGTATTCAGAATCAGAACGGACATCCCACCCTGCCTAGCAATTCAGTAACAC
AGGGGGCTGCTCTCAATCACCTCTCCTCTCACACTGCTACCTCAGGTGGACAACAAGGCATTACCTTAAC
CAAAGAGAGCAAGCCTTCAGGAAACATATTGACGGTGCCTGAAACAAGCAGGCACACTGGAGAGACACCT
AACAGCACTGCCAGTGTCGAGGGACTTCCTAATCATGTCCATCAGATGACGGCAGATGCTGTTTGCAGTC
CTAGCCATGGAGATTCTAAGTCACCAGGTTTACTAAGTTCAGACAATCCTCAGCTCTCTGCCTTGTTGAT
GGGAAAAGCCAATAACAATGTGGGTACTGGAACCTGTGACAAAGTCAATAACATCCACCCAGCTGTTCAT
ACAAAGACTGATAACTCTGTTGCCTCTTCACCATCTTCAGCCATTTCAACAGCAACACCTTCTCCAAAAT
CCACTGAGCAGACAACCACAAACAGTGTTACCAGCCTTAACAGCCCTCACAGTGGGCTACACACAATTAA
TGGAGAAGGGATGGAAGAATCTCAGAGCCCCATGAAAACAGATCTGCTTCTGGTTAACCACAAACCTAGT
CCACAGATCATACCATCAATGTCTGTGTCCATATACCCCAGCTCAGCAGAAGTTCTGAAGGCATGCAGGA
ATCTAGGTAAAAATGGCTTATCTAACAGTAGCATTTTGTTGGATAAATGTCCACCTCCAAGACCACCATC
TTCACCATACCCTCCCTTGCCAAAGGACAAGTTGAATCCACCTACACCTAGTATTTACTTGGAAAATAAA
CGTGATGCTTTCTTTCCTCCATTACATCAATTTTGTACAAATCCGAACAACCCTGTTACAGTAATACGTG
GCCTTGCTGGAGCTCTTAAGTTAGACCTGGGACTTTTCTCTACTAAAACTTTGGTGGAAGCTAACAATGA
ACATATGGTAGAAGTGAGGACACAGTTGTTGCAGCCAGCAGATGAAAACTGGGATCCCACTGGAACAAAG
AAAATCTGGCATTGTGAAAGTAATAGATCTCATACTACAATTGCTAAATATGCACAGTACCAGGCCTCCT
CATTCCAGGAATCATTGAGAGAAGAAAATGAAAAAAGAAGTCATCATAAAGACCACTCAGATAGTGAATC
TACATCGTCAGATAATTCTGGGAGGAGGAGGAAAGGACCCTTTAAAACCATAAAGTTTGGGACCAATATT
GACCTATCTGATGACAAAAAGTGGAAGTTGCAGCTACATGAGCTGACTAAACTTCCTGCTTTTGTGCGTG
TCGTATCAGCAGGAAATCTTCTAAGCCATGTTGGTCATACCATATTGGGCATGAACACAGTTCAACTATA
CATGAAAGTTCCAGGGAGCAGAACACCAGGTCATCAGGAAAATAACAACTTCTGTTCAGTTAACATAAAT
ATTGGCCCAGGTGACTGTGAATGGTTTGTTGTTCCTGAAGGTTACTGGGGTGTTTTGAATGACTTCTGTG
AAAAAAATAATTTGAATTTCCTAATGGGTTCTTGGTGGCCCAATCTTGAAGATCTTTATGAAGCAAATGT
TCCAGTGTATAGGTTTATTCAGCGACCTGGAGATTTGGTCTGGATAAATGCAGGCACTGTTCATTGGGTT
CAGGCTATTGGCTGGTGCAACAACATTGCTTGGAATGTTGGTCCACTTACAGCCTGCCAGTATAAATTGG
CAGTGGAACGGTACGAATGGAACAAATTGCAAAGTGTGAAGTCAATAGTACCCATGGTTCATCTTTCCTG
GAATATGGCACGAAATATCAAGGTCTCAGATCCAAAGCTTTTTGAAATGATTAAGTATTGTCTTCTAAGA
ACTCTGAAGCAATGTCAGACATTGAGGGAAGCTCT**C**ATTGCTGCAGGAAAAGAGATTATATGGCATGGGC
GGACAAAAGAAGAACCAGCTCATTACTGTAGCATTTGTGAAGTGGAGGTTTTTGATCTGCTTTTTGTCAC
TAATGAGAGTAATTCACGAAAGACCTACATAGTACATTGCCAAGATTGTGCACGAAAAACAAGCGGAAAC
TTGGAAAACTTTGTGGTGCTAGAACAGTACAAAATGGAGGACCTGATGCAAGTCTATGACCAATTTACAT
TAGCTCCTCCATTACCATCCGCCTCATCTTGATATTGTTCCATGGACATTAAATGAGACCTTTTCTGCTA
TTCAGGAAATAACCCAGTTCTGCACCACTGGTTTTTGTAGCTATCTCGTAAGGCTGCTGGCTGAAAACTG
TGTCTATGCAACCTTCCAAGTGCGGAGTGTCAACCAACTGGACGGGAGAGAGTACTGCTCCTACTCCAGG
ACTCTCACAAAGCTGATGAGCTGTACTTCAGAAAAAAATAATAATTTCCATGTTTTGTATATATCTGACA
AAACTGGCAACATCTTACAGACTACTGACTTGAAGACAACCTCTTTTATATTTCTCTATTTCTGGGCTGA
TGAATTTGTTTTCATCTGTCTTTTCCCCCTTCAGAATTTTCCTTGGAAAAAAAATACTAGCCTAGCTGGT
CATTTCTTTGTAAGGTAGTTAGCAATTTTAAGTCTTTCTTTGGTCAACTTTTTTTTAATGTGAAAAGTTA
GGTAAGACACTTTTTTACTGCTTTTATGTTTTTCTGTCTTGTTTTGAGAC**CATGATGGTTACACTTTTGG**
TTCCTAAATAAAATTTAAAAAATTAACAGCCAAGTCACAAAGGTAATGGATTGCACATAGACTAAGGAAT
AAACTTCAGATTTGTGAAAAAAAAAA

45) Entrez Gene ID 7514 = XPO1, exportin 1 (CRM1 homolog, yeast) = BX647758

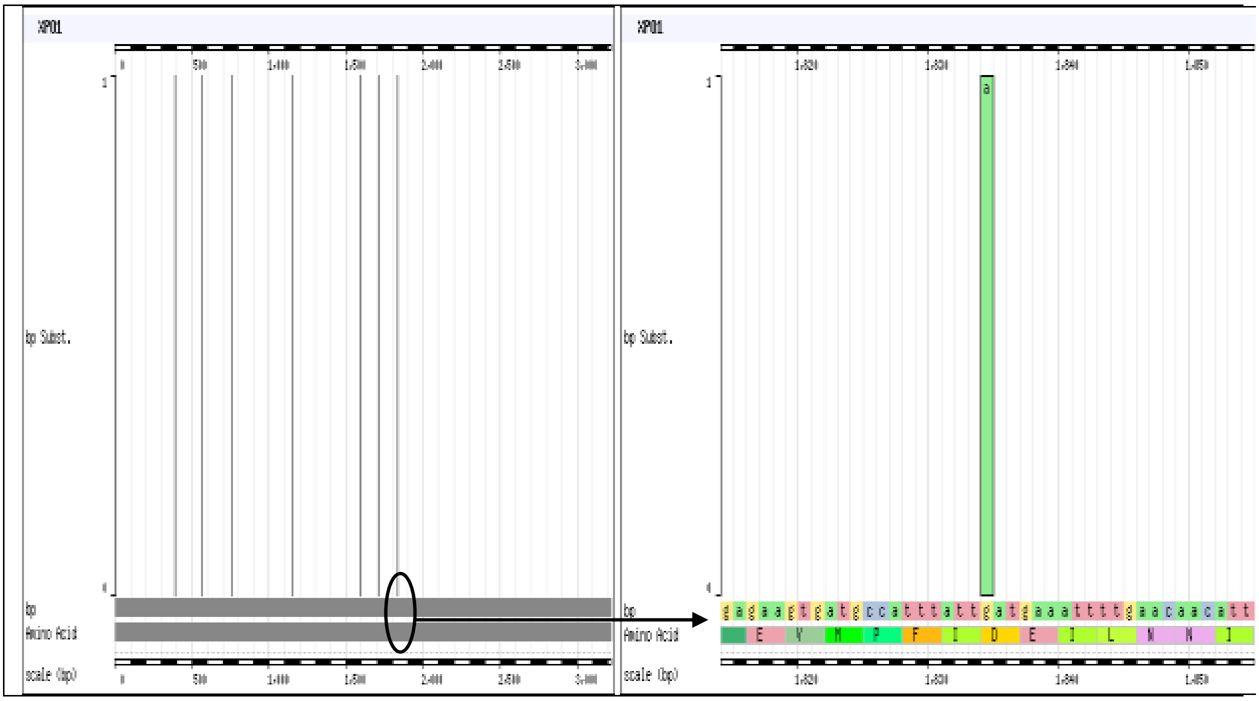

>gi|34366915|emb|BX647758.1| Homo sapiens mRNA; cDNA DKFZp686K0731 (from clone DKFZp686K0731)
GGAGGCACTAGGAGGAGGGGGAGAAGCGGCTGCAGCGGCCGCGGCAGGAGCAGCGGGAGCTACAGCATCA
GCAAGAGCAACAGTAGCTACAGCCCCGGCGGCGGTGCCTGTTCCAGTCTTTGCTGCTGCAGTCCGTGCAA
CCACCCAGAGGGGGAGGGGGGAACCACCAGTCGCTGAGGAACAAGAGAAGGGGGGAAAGTTTAGGCGAGC
CTTGGGGGGGGGGGGCAGCGCCGGAGCCGCGTGAGAGAGGGAGCCGTGTTTTGGTAGGGGGGAGTCGGAC
TGCAACTGGCAGCAGAGCGTCTCCCCGGCCGTGTGGACTCTACACCTCCTACTCCTGCCGCTTCTGCTGC
TGCCTGTGGCTGGAGGGTCCCCCTGGGGCTGAATCTTTGGGACTTGACCCCGTTCCCTCCCCCTTCCCTC
ACTCCCCAGCCGGGCGGGAGCATTTATTCCCCAGATTAATTCCCCTTTTGGGGGGGGGCGGGTGTGTGTG
TGTGTGTGTGTGTGTGTGTGTGTTGGGGGAAGCGTCCCTGAAATAGTAAATATTATTGAGCTCTTTTTGC
CCTTTTCCTGTCCGTTTTTTTAATTTCCTTTTTTGAGGTGGGAAAACTGAAACCCACCTTGATTCGTCCC
CTCTCCCCCCTCCCCACCTTCCCTCGCCCTAATCCCCCAACGAGGAAGGAAGGAGCAGTTGGTTCAATCT
CTGGTAATCTATGCCAGCAATTATGACAATGTTAGCAGACCATGCAGCTCGTCAGCTGCTTGATTTCAGC
CAAAAACTGGATATCAACTTATTAGATAATGTGGTGAATTGCTTATACCATGGAGAAGGAGCCCAGCAAA
GAATGGCTCAAGAAGTACTGACACATTTAAAGGAGCATCCTGATGCTTGGACAAGAGTCGACACAATTTT
GGAATTTTCTCAGAATATGAATACGAAATACTATGGACTACAAATTTTGGAAAATGTGATAAAAACAAGG
TGGAAGATTCTTCCAAGGAACCAGTGCGAAGGAATAAAAAAATACGTTGTTGGCCTCATTATCAAGACGT
CATCTGACCCAACTTGTGTAGAGAAAGAAAAGGTGTATATCGGAAAATTAAATATGATCCTTGTTCAGAT
ACTGAAACAAGAATGGCCCAAACATTGGCCAACTTTTATCAGTGATATTGTTGGAGCAAGTAGGACCAGC
GAAAGTCTCTGTCAAAATAATATGGTGATTCTTAAACTCTTGAGTGAAGAAGTATTTGATTTCTCTAGTG
GACAGATAACCCAAGTCAAATCTAAGCATTTAAAAGACAGCATGTGCAATGAATTCTCACAGATATTTCA
ACTGTGTCAGTTTGTAATGGAAAATTCTCAAAATGCTCCACTTGTACATGCAACCTTGGAAACATTGCTC
AGATTTCTGAACTGGATTCCCCTGGGATATATTTTTGAGACCAAATTAATCAGCACATTGATTTATAAGT
TCCTGAATGTTCCAATGTTTCGAAATGTCTCTCTGAAGTGCCTCACTGAGATTGCTGGTGTGAGTGTAAG
CCAATATGAAGAACAATTTGTAACACTATTTACTCTGACAATGATGCAACTAAAGCAGATGCTTCCTTTA
AATACCAATATTCGACTTGCGTACTCAAATGGAAAAGATGATGAACAGAACTTCATTCAAAATCTCAGTT
TGTTTCTCTGCACCTTTCTTAAGGAACATGATCAACTTATAGAAAAAAGATTAAATCTCAGGGAAACTCT
TATGGAGGCCCTTCATTATATGTTGTTGGTATCTGAAGTAGAAGAAACTGAAATCTTTAAAATTTGTCTT
GAATACTGGAATCATTTGGCTGCTGAACTCTATAGAGAGAGTCCATTCTCTACATCTGCCTCTCCGTTGC
TTTCTGGAAGTCAACATTTTGATGTTCCTCCCAGGGGACAGCTATATTTGCCCATGTTATTCAAGGTCCG
TTTATTAATGGTTAGTCGAATGGCTAAACCAGAGGAAGTATTGGTTGTAGAGAATGATCAAGGAGAAGTT
GTGAGAGAATTCATGAAGGATACAGATTCCATAAATTTGTATAAGAATATGAGGGAAACATTGGTTTATC
TTACTCATCTGGATTATGTAGATACAGAAAGAATAATGACAGAGAAGCTTCACAATCAAGTGAATGGTAC
AGAGTGGTCATGGAAAAATTTGAATACATTGTGTTGGGCAATAGGCTCCATTAGTGGAGCAATGCATGAA

GAGGACGAAAAACGATTTCTTGTTACTGTTATAAAGGATCTATTAGGATTATGTGAACAGAAAAGAGGCA
AAGATAATAAAGCTATTATTGCATCAAATATCATGTACATAGTAGGTCAATACCCACGTTTTTTGAGAGC
TCACTGGAAATTTCTGAAGACTGTAGTTAACAAGCTGTTCGAATTCATGCATGAGACCCATGATGGAGTC
CAGGATATGGCTTGTGATACTTTCATTAAAATAGCCCAAAAATGCCGCAGGCATTTCGTTCAGGTTCAGG
TTGGAGAAGTGATGCCATTTATT**G**ATGAAATTTTGAACAACATTAACACTATTATTTGTGATCTTCAGCC
TCAACAGGTTCATACGTTTTATGAAGCTGTGGGGTACATGATTGGTGCACAAACAGATCAAACAGTACAA
GAACACTTGATAGAAAAGTACATGTTACTCCCTAATCAAGTGTGGGATAGTATAATCCAGCAGGCAACCA
AAAATGTGGATATACTGAAAGATCCTGAAACAGTCAAGCAGCTTGGTAGCATTTTGAAAACAAATGTGAG
AGCCTGCAAAGCTGTTGGACACCCCTTTGTAATTCAGCTTGGAAGAATTTATTTAGATATGCTTAATGTA
TACAAGTGCCTCAGTGAAAATATTTCTGCAGCTATCCAAGCTAATGGTGAAATGGTTACAAAGCAACCAT
TGATTAGAAGTATGCGAACTGTAAAAAGGGAAACTTTAAAGTTAATATCTGGTTGGGTGAGCCGATCCAA
TGATCCACAGATGGTCGCTGAAAATTTTGTTCCCCCTCTGTTGGATGCAGTTCTCATTGATTATCAGAGA
AATGTCCCAGCTGCTAGAGAACCAGAAGTGCTTAGTACTATGGCCATAATTGTCAACAAGTTAGGGGGAC
ATATAACAGCTGAAATACCTCAAATATTTGATGCTGTTTTTGAATGCACATTGAATATGATAAATAAGGA
CTTTGAAGAATATCCTGAACATAGAACGAACTTTTTCTTACTACTTCAGGCTGTCAATTCTCATTGTTTC
CCAGCATTCCTTGCTATTCCACCTACACAGTTTAAACTTGTTTTGGATTCCATCATTTGGGCTTTCAAAC
ATACTATGAGGAATGTCGCAGATACGGGCTTACAGATACTTTTTACACTCTTACAAAATGTTGCACAAGA
AGAAGCTGCAGCTCAGAGTTTTTATCAAACTTATTTTTGTGATATTCTCCAGCATATCTTTTCTGTTGTG
ACAGACACTTCACATACTGCTGGTTTAACAATGCATGCATCAATTCTTGCATATATGTTTAATTTGGTTG
AAGAAGGAAAAATAAGTACATCATTAAATCCTGGAAATCCAGTTAACAACCAAATCTTTCTTCAGGAATA
TGTGGCTAATCTCCTTAAGTCGGCCTTCCCTCACATACAAGATGCTCAAGTAAAGCTCTTTGTGACAGGG
CTTTTCAGCTTAAATCAAGATATTCCTGCTTTCAAGGAACATTTAAGAGATTTCCTAGTTCAAATAAAGG
AATTTGCAGGTGAAGACACTTCTGATTTGTTTTTGGAAGAGAGAGAAATAGCCCTACGGCAGGCTGATGA
AGAGAAACATAAACGTCAAATGTCTGTCCCTGGCATCTTTAATCCACATGAGATTCCAGAAGAAATGTGT
GATTAAAATCCAAATTCATGCTGTTTTTTTTCTCTGCAACTCGTTAGCAGAGGAAAACAGCATGTGGGTA
TTTGTCGACCAAAATGATGCCAATTTGTAAATTAAAATGTCACCTAGTGGCCCTTTTTCTTATGTGTTTT
TTTGTATAAGAAATTTTCTGTGAAATATCCTTCCATTGTTTAAGCTTTTGTTTTGGTCATCTTTATTTAG
TTTGCATGAAGTTGAAAATTAAGGCATTTTTAAAAATTTTACTTCATGCCCATTTTTGTGGCTGGGCTGG
GGGGAGGAGGCAAATTCGATTTGAACATATACTTGTAATTCTAATGCAAAATTATACAATTTTTCCTGTA
AACAATACCAATTTTTAATTAGGGAGCATTTTCCTTCTAGTCTATTTCAGCCTAGAAGAAAAGATAATGA
GTAAAACAAATTGCGTTGTTTAAAGGATTATAGTGCTGCATTGTCTGAAGTTAGCACCTCTTGGACTGAA
TCGTTTGTCTAGACTACATGTATTACAAAGTCTCTTTGGCAAGATTGCAGCAAGAT**CATGTGCATATCAT**
CCCATTGTAAAGCGACTTCAAAAATATGGGAACACAGTTAGTTATTTTTACACAGTTCTTTTTGTTTTTG
TGTGTGTGTGTGCTGTCGCTTGTCGACAACAGCTTTTTGTTTTCCTCAATGAGGAGTGTTGCTCATTTGT
GAGCCTTCATTAACTCGAAGTGAAATGGTTAAAAATATTTATCCTGTTAGAATAGGCTGCATCTTTTTAA
CAACTCATTAAAAAACAAAACAACTCTGGCTTTTGAGATGACTTATACTAATTTACATTGTTTACCAAGC
TGTAGTGCTTTAAGAACACTACTTAAAAAGCAAAATAAACTTGGTTTACATTCAAAAAAAAAAAAAAAAA
AA

46) Entrez Gene ID 7750 = ZMYM2, zinc finger, MYM-type 2 = AF012126

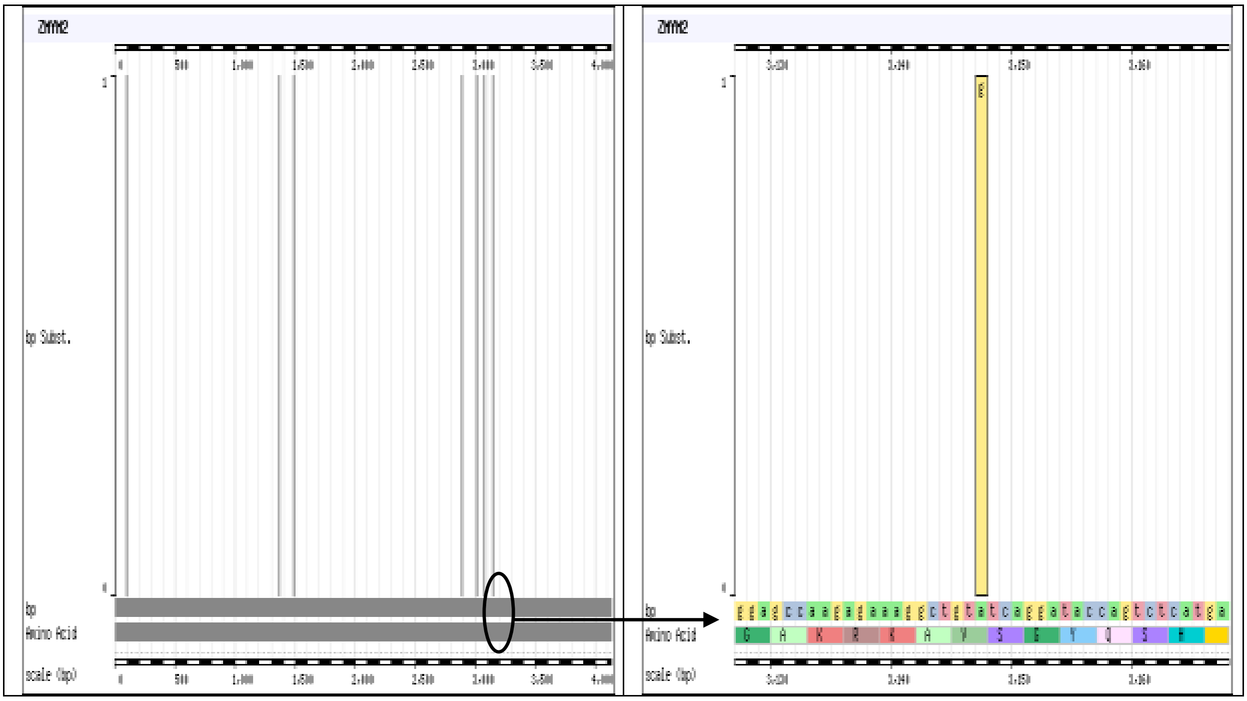

>gi|2832227|gb|AF012126.1| Homo sapiens zinc finger protein (ZNF198) mRNA, complete cds
GGTGTTGGCAGAGGCAAAAAGTGTAATGAAAAATTGGTAATTCTTTTCTAGTTAAGTTGTAGTCTTAAAA
ATTATCTTTTTATGTTTCAAATTTTAGAAATATGGAAAACTGACAACTTGTACTGGTTGCCGAACACAGT
GCAGGTTTTTTGATATGACTCAGTGTATAGGTCCTAATGGATATATGGAGCCATATTGTTCAACTGCTTG
TATGAACAGTCACAAGACAAAATATGCAAAATCACAAAGTTTGGGAATTATTTGCCATTTTTGTAAGCGA
AACTCTTTACCTCAATACCAAGCCACAATGCCTGATGGAAAACTGTACAACTTTTGCAATTCCAGTTGTG
TGGCTAAATTTCAGGGTATTACCCAAAGGTTCAAACCCGTGCATAAAGCCATGCTTCATGAGTTAATTCA
TCCAACAATTCTGCTTTATTCCAGGCTCTAAGTATGCAGTCATCTCCAAATGGCCAGTTTGTAGCGCCAA
GTGATATTCAGTTGAAATGCAACTACTGCAAAAATTCCTTTTGTTCAAAACCAGAAATCCTGGAATGGGA
GAACAAAGTGCATCAGTTCTGCAGCAAAACTTGTTCAGATGACTATAAGAAGTTGCATTGCATAGTTACA
TATTGCGAATACTGTCAAGAGGAGAAGACTCTTCATGAAACAGTAAATTTCTCTGGCGTTAAGAGACCTT
TCTGTAGTGAAGGCTGCAAATTATTATACAAACAGGATTTTGCCAGACGTTTAGGATTGAGATGTGTTAC
TTGCAACTATTGTTCTCAGCTATGTAAGAAGGGAGCAACTAAAGAACTCGATGGTGTTGTGAGAGATTTC
TGCAGTGAAGATTGCTGTAAAAAATTTCAGGATTGGTACTACAAGGCTGCAAGGTGTGACTGTTGTAAAT
CTCAAGGAACTCTTAAAGAGCGAGTTCAGTGGCGTGGGGAAATGAAACATTTCTGTGATCAACATTGCTT
ACTGCGTTTCTACTGTCAACAAAATGAGCCCAACATGACAACTCAGAAAGGACCTGAAAACTTACATTAT
GATCAGGGTTGTCAGACATCTCGAACCAAAATGACAGGTTCAGCACCACCCCCTTCTCCAACACCTAACA
AAGAGATGAAGAACAAAGCAGTTCTTTGCAAACCTTTAACAATGACAAAAGCTACTTACTGTAAACCTCA
CATGCAGACCAAATCTTGTCAGACAGATGATACTTGGAGGACAGAATATGTTCCAGTGCCTATCCCTGTG
CCTGTGTATATCCCAGTTCCTATGCACATGTACAGTCAGAATATTCCTGTTCCTACTACAGTTCCTGTTC
CTGTGCCAGTTCCTGTTTTTCTGCCTGCTCCATTGGACAGCAGTGAGAAGATTCCTGCAGCAATTGAGGA
GCTAAAAAGCAAGGTTTCTTCAGATGCTCTTGATACAGAGTTGCTTACAATGACGGATATGATGAGTGAA
GACGAGGGGAAAACAGAGACAACCAACATCAACAGTGTAATTATTGAAACAGATATAATTGGTTCAGACC
TTTTGAAGAACTCTGACCCAGAGACACAGTCCAGCATGCCTGATGTACCATATGAACCAGATTTGGATAT
CGAAATAGATTTTCCCAGAGCTGCTGAGGAGCTTGATATGGAAAATGAATTTTTATTACCACCTGTTTTT
GGCGAAGAATATGAGGAACAGCCCAGACCTCGATCTAAAAAAAAGGGAGCCAAGAGAAAGGCTGTATCAG
GATACCAGTCTCATGATGATAGTTCTGACAATTCAGAATGCAGCTTTCCTTTCAAATATACGTATGGCGT
AAATGCATGGAAACACTGGGTCAAAACTAGGCAACTTGATGAAGATCTTCTGGTATTAGATGAGTTAAAA
TCTTCTAAATCAGTAAAGTTAAAAGAGGATCTACTCTCTCACACCACAGCTGAGCTTAACTATGGGTTAG
CTCATTTTGTCAATGAGATCCGACGGCCAAATGGAGAGAATTATGCACCTGACAGCATCTATTACCTTTG
CCTTGGAATACAGGAGTATTTGTGTGGAAGTAATCGAAAAGACAACATATTTATTGATCCTGGATACCAA
ACATTTGAGCAAGAATTGAATAAAATACTGCGAAGCTGGCAACCAAGCATACTTCCAGATGGGTCAATAT
TCTCTCGAGTTGAAGAAGACTATCTCTGGAGGATAAAACAACTAGGATCACACTCTCCAGTAGCTCTTCT

GAATACACTGTTCTACTTTAACACTAAGTATTTTGGCCTGAAAACAGTGGAACAACACTTAAGACTTTCC
TTTGGCACTGTGTTTAGGCATTGGAAAAAAAATCCTTTAACGATGGAAAACAAAGCGTGTCTTCGATACC
AAGTGTCTTCCTTGTGTGGAACAGATAATGAAGATAAAATTACTACTGGAAAAAGAAAACATGAAGATGA
TGAGCCAGTATTTGAACAAATTGAAAACACAGCCAATCCTTCCAGATGTCCTGTGAAAATGTTTGAATGC
TACTTGTCTAAAAGTCCACAGAATCTTAATCAGAGGATGGATGTTTTTTATTTGCAACCAGAATGCTCTA
GTTCTACAGATAGCCCTGTCTGGTATACGTCTACTTCACTGGACCGAAACACCTTGGAAAATATGCTTGT
ACGGGTTCTTCTAGTAAAAGATATTTATGATAAAGACAATTATGAACTGGATGAAGACACAGACTAAAAA
GGAACGTTGCAGAAGCAATCGGGATAAAACAGCATTAGATAGTCATGCTGCTAGATCTTTATTATGGAAA
ACATTTCAAGTTTACTCCTTCTGTTTTGAGTTTTGTAGCAGTGTACCCACGCTGGGTATTACCATGTAAA
TAATCTGTGAGTGAAAGTTGCCATTATTCTATGTAGTGGTTTTAGGATACTTAACAAATACATTCAAATT
CTTTTTTTATTATTATTTATTTGATTAGGTATGTTTGTAACTTTTTACATTACAGAATATGAATGAGAAT
GTGC**CATGTATAATTTTTTTCTTGT**AGTAAGAAACATCCATATTGCACAACTCTACTGTTGCAAAGCTTC
CTTGGAAGGGGGCTCTTTTACTGGGTTCTTAACCAGATGGTTGTGTATGGGTAGCACTACTAAAAGTTTA
GAACTTGCAGTGTCTTTCGGAATTTTTAAAATAAACTGTAAACTAATAGGCTGGGGTTTTTGTTTTGTTT
TGGGGTTTTGTTTTGTTTGGTTTTACATTTTAGTTACTGAAGCCTTACAAGGTTATGTAGAGAGATACCA
TCTTCTGTACCAAAAATAGACAAGAGAATGCTGTCAATATTGGTGTACTGTAATGTGAATCTATGCTGGT
GAAAACAATTTTTTGTTCCCCTTATTAAAACCTTAGTGTCCTTTTCTCATTTGTGGCTTTCTGCATCACC
CAATCAATAAAAAACAAATATATATATGTAAAAAAAA

47) Entrez Gene ID 8028 = MLLT10, myeloid/lymphoid or mixed-lineage leukemia (trithorax homolog, Drosophila); translocated to, 10 = BX648210

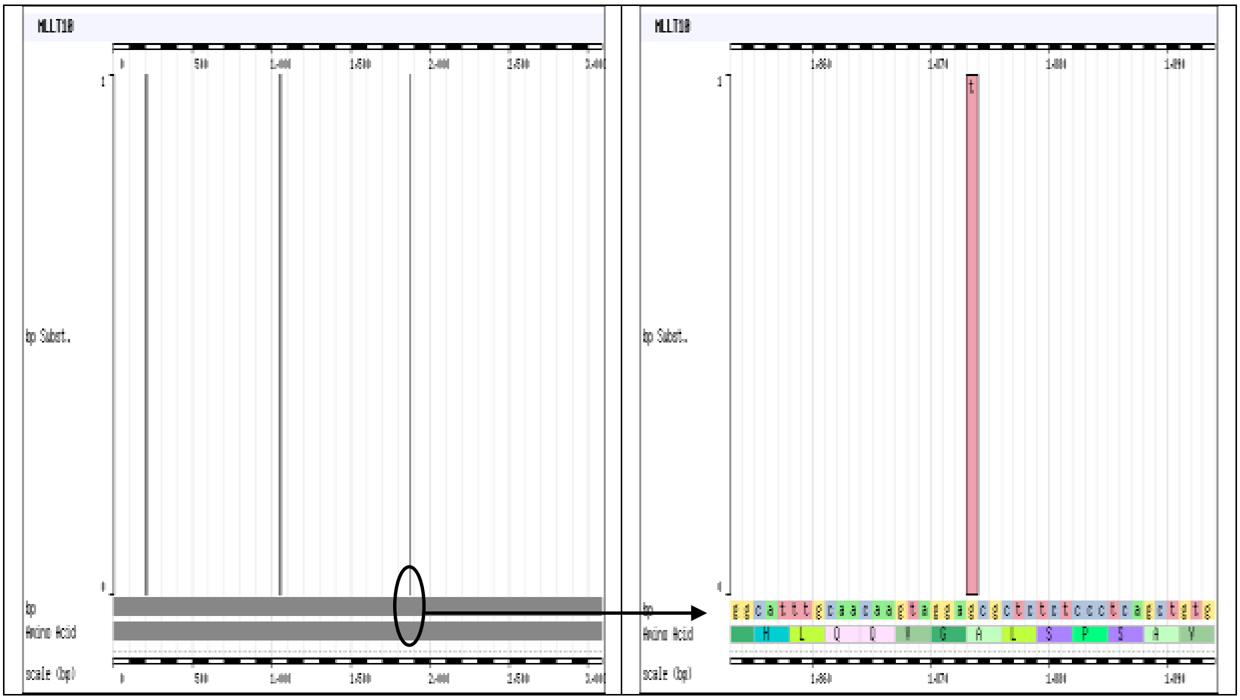

>gi|34367369|emb|BX648210.1| Homo sapiens mRNA; cDNA DKFZp779J0967 (from clone DKFZp779J0967)
GATAGGTCATATGATCAAAGTTTAAGTGATTCTTCCTCTCACTCTCAGGATAAACATCATGAGAAAGAGA
AAAAAAAATATAAAGAGAAGGACAAACACAAACAGAAACACAAGAAGCAGCCAGAACCATCACCTGCATT
GGTTCCATCCTTGACTGTTACTACAGAAAAAACTTATACAAGCACTAGCAACAACTCTATATCTGGATCA
TTGAAGCGCTTGGAAGATACTACTGCACGATTTACAAATGCAAATTTCCAGGAAGTCTCTGCACACACCT
CTAGTGGAAAAGATGTTTCAGAGACTAGAGGGTCAGAGGGCAAAGGGAAGAAATCTTCAGCTCACAGCTC
AGGTCAAAGGGGAAGAAAGCCTGGTGGTGGAAGAAATCCAGGAACAACTGTGTCAGCAGCTAGCCCTTTT
CCTCAAGCAGGATATAAGCGGGCTCAAACTTCTGGCATAGAAGAAGAAACTGTAAAGGAAAAGAAAAGGA
AAGGAAATAAACAAAGTAAGCATGGGCCTGGCAGACCCAAAGGAAACAAAAATCAAGAGAATGTTTCTCA
TCTCTCAGTTTCTTCTGCTTCACCAACATCATCTGTAGCATCAGCTGCAGGAAGCATAACAAGCTCTAGT

CTGCAGAAATCTCCTACATTGCTCAGGAATGGAAGTTTACAGAGCCTCAGTGTTGGCTCATCTCCAGTTG
GTTCAGAAATTTCCATGCAGTATCGGCATGATGGAGCTTGCCCAACAACTACGTTCTCAGAGTTGCTGAA
TGCAATACACAACGGTATTTATAACAGCAATGATGTAGCAGTATCGTTTCCAAATGTAGTATCTGGCTCG
GGATCTAGTACTCCTGTCTCCAGCTCTCACTTACCTCAGCAGTCTTCTGGGCATTTGCAACAAGTAGGA**G**
CGCTCTCTCCCTCAGCTGTGTCATCTGCAGCCCCTGCTGTTGCTACAACTCAGGCAAATACTCTATCTGG
ATCTTCTCTCAGTCAGGCACCATCTCATATGTATGGCAATAGATCAAATTCATCAATGGCAGCTCTTATA
GCTCAGTCTGAAAACAATCAAACAGATCAAGATCTTGGAGACAATAGCCGCAACCTAGTTGGCAGAGGAA
GCTCACCCCGAGGAAGTCTCTCGCCACGATCCCCTGTAAGCAGCTTACAGATTCGCTATGATCAACCAGG
CAACAGCAGTTTGGAAAATCTGCCTCCAGTAGCAGCCAGCATAGAACAGCTTTTGGAGAGGCAGTGGAGT
GAAGGACAGCAATTTTTACTAGAACAGGGTACTCCTAGTGACATTTTAGGAATGCTGAAGTCATTACACC
AACTTCAAGTTGAAAACCGAAGATTAGAGGAACAAATTAAAAACTTGACTGCCAAAAAGGAACGGCTTCA
GTTATTGAATGCACAGCTTTCAGTGCCTTTTCCAACAATAACAGCAAATCCTAGTCCGTCTCATCAAATA
CACACATTTTCAGCACAGACTGCTCCTACTACTGATTCCTTGAACAGCAGTAAGAGCCCTCATATAGGAA
ACAGCTTTTTACCTGATAATTCTCTTCCTGTATTAAATCAGGACTTAACCTCCAGTGGACAAAGTACCAG
CAGCTCATCAGCTCTTTCTACCCCACCTCCTGCTGGGCAGAGTCCGGCTCAACAAGGCTCAGGAGTGAGT
GGAGTTCAGCAGGTCAATGGCGTGACAGTGGGGGCACTAGCTAGTGGAATGCAGCCTGTAACTTCCACCA
TTCCTGCCGTGTCTGCAGTGGGTGGAATAATTGGAGCTTTGCCAGGTAACCAACTGGCAATTAATGGCAT
TGTAGGAGCTTTAAATGGGGTTATGCAGACTCCTGTCACAATGTCCCAGAACCCTACCCCTCTCACCCAC
ACAACCGTACCACCTAATGCAACACATCCAATGCCAGCTACACTGACTAACAGTGCCTCAGGACTAGGAT
TACTTTCTGACCAGCAACGACAAATACTTATTCATCAACAGCAGTTTCAGCAGTTGTTAAATTCTCAACA
GCTCACACCAGAACAACATCAAGCCTTTTTGTATCAGTTAATGCAACATCACCACCAGCAGCACCACCAA
CCTGAACTTCAGCAGCTGCAGATCCCTGGACCAACACAAATACCCATAAACAACCTTCTTGCAGGTACAC
AGGCACCCCCACTTCACACAGCTACCACCAACCCATTTCTCACCATCCATGGAGATAATGCAAGTCAGAA
AGTAGCAAGACTTAGTGATAAAACTGGGCCTGTAGCTCAAGAGAAAAGTTGACACCTGAGAAACATCTAG
AAATTGCCTATCCTGCTGTTCTAGCACTTCATCTGGCTGCCTTTGCAGTCCTTTTACTACAGCTATGAAG
AAACGCAACAAGAAACTCAATGCACAACAAAGGATTAATTGCTGCAAGGACATTCTTGTAAGGCTTTGAT
TAGTTTTCTTGTTGCTTTGTTGCACTGAAATGGAATTCCCATGCCCCTACCCCTTACCCCAGTTTTTTGA
ACATGGAAAGAAAATTTAATAACTTTTTAAAGTGACATAATTTACATGCAATATGTTTATCAACTCAAGA
ATTTAATATAGTTGGTACACAACTAGTTTTGTTTATAAATTGGAGATGCAAATAGCAAAACTAAATACTT
GCTCCATTTACAAACTACTTGATTTTATTGTACAAGTTGAAATATGCTCTTTTGTTTGGGTTACAGTATG
CTTGCTCTAAGTCAAATTCCAAGGAACTAATTTCTTCTCCTGGAGTTGCATTGATTCAGTATTACAAATA
TATAGCACATCACCTGGGACTTGGCAATCTTTGTTAAAAAAAAATTTCCTTTCTAATGGGATTTGGCCGA
TTTTGGTAATGAAGTTAGGATGGTAATGTCTGCATCTGCTAAAGGTAATTTTCTTTTGAGAATTGCTTTC
TTTAGTGTTAAGACCTACTCATATTTTGAAGAAATCTTGAGTTAAGTGAGTTCTGAGGCTGCTGGGGGAA
CCAGATCAATTCAAAGCTAAATACTTCTTTCAGAAAGGGGCCACTGTGGAAAGTGCTGGTGGGGTTTGCC
CTTGATCAAGTGCCTCCATTGTGCTGCAAGGGCTGTAGACAGCAGGGTGGGACAGTCAGTCCTCCGAGCA
GCAGGAATCATCCCGTCACCTGCAGCCTTCCCATGCTTCCGCCTTTATTCAGAACTTTCTGTGCCACTGT
AGATAGCTCAGGCAAAACTATTACCTGGGTATTTATCCACTAATGAGTCACAAGAAAAGGAGTGGATTTG
GTAAGAATAGAGATTTGTTTTATTTAAACCACTTCCCATTACTGACCATTAAAAGCTCACCACTAGAGTT
CCTGAAACAGGTGAAACCTGTATGACAGCCCTTCCACTTTGGGGAGCCACGCTTTTGATGTGACAGTACC
GCAGAGTGATTCCCCCACTGAGGATGTCATCATCAAACTCTTCTTTGGTGTGTGAATTATTAGTGGAAAA
GACCCAGCTGTAATTAGACCTCCACTGTGTACTTAGCTGGAAGAACATGTTAATTCTGCAATATGTTTCT
TGGTTAAACATTGCACAGTTCTTACCTCATTTCTGTAAATAAAGTTTTGTGAATCTGTTTTGTATTGTGA
CAAATTCATAAGATAACATTGATATTTTGATTTGTAATATTTCTAATTGGTAGATTTAATTGAAAAGTAA
AATTAATTTATTTTTATATGTTCAGGGGAATTTTAAAGTCAAATCTTTTGTAGATAATTTAAAAAATCAG
TGTGGTTTATTTTACTTATTTAACCCACTGGTTGTTATTCTGTAACAGTTTGTATAAATGGTAAATTTTG
AATGTGTTGTTATTTTACCTAGATGTAAAATTCCA**CATGTATTAAAATGTACAAAA**TGTTTTGTTAATAA
AATTTAATAATGTTCAAAAAAAAAA

48) Entrez Gene ID 8030 = CCDC6, coiled-coil domain containing 6 = BC064391

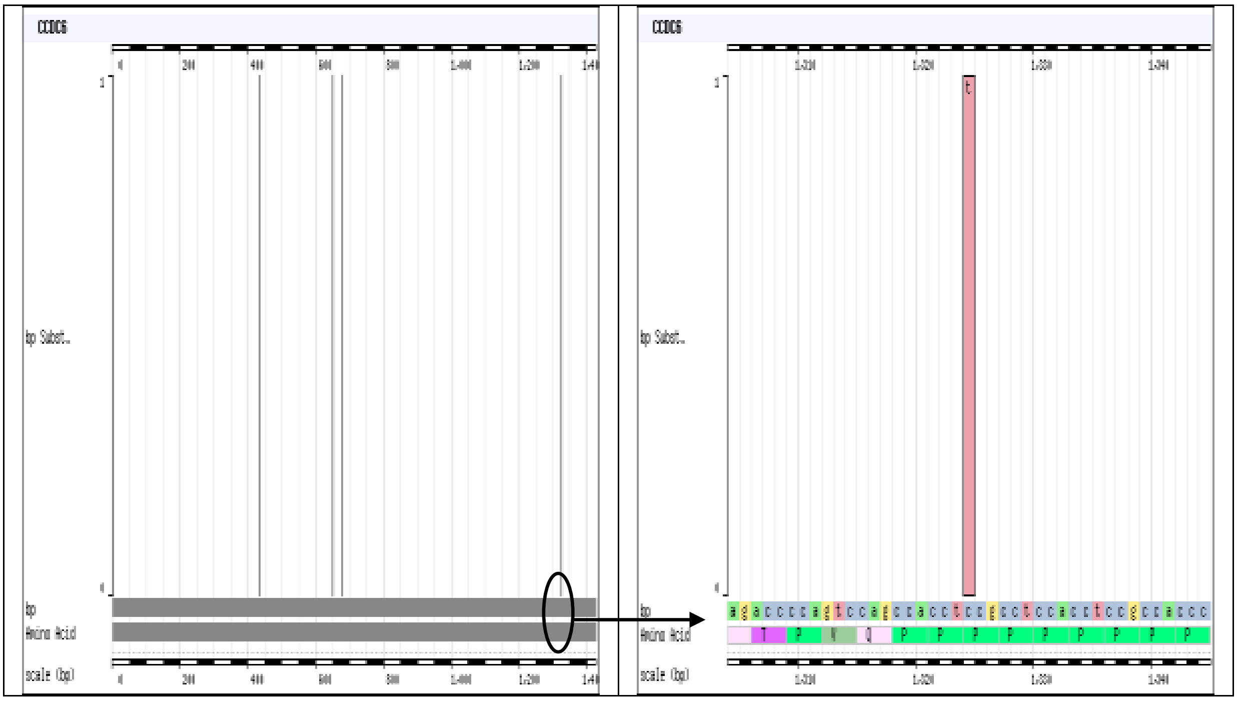

>gi|39963680|gb|BC064391.1| Homo sapiens coiled-coil domain containing 6, mRNA (cDNA clone MGC:75097 IMAGE:5745698), complete cds

GCCTTCGTCGCCGCCGCCTCCCTGCTGCTCCTCCTCCTTTCCCCAGCCCGCCGCGGCCATGGCGGACAGC
GCCAGCGAGAGCGACACGGACGGGGCGGGGGGCAACAGCAGCAGCTCGGCCGCCATGCAGTCGTCCTGCT
CGTCGACCTCGGGCGGCGGCGGTGGCGGCGGGGGAGGCGGCGGCGGTGGGAAGTCGGGGGGCATTGTCAT
CTCGCCGTTCCGCCTGGAGGAGCTCACCAACCGCCTGGCCTCGCTGCAGCAAGAGAACAAGGTGCTGAAG
ATAGAGCTGGAGACCTACAAACTGAAGTGCAAGGCACTGCAGGAGGAGAACCGCGACCTGCGCAAAGCCA
GCGTTACCATCCAAGCCAGGGCTGAGCAGGAAGAAGAATTCATTAGTAACACTTTATTCAAGAAAATTCA
GGCTTTGCAGAAGGAGAAAGAAACCCTTGCTGTAAATTATGAGAAAGAAGAAGAATTCCTCACTAATGAG
CTCTCCAGAAAATTGATGCAGTTGCAGCATGAGAAAGCCGAACTAGAACAGCATCTTGAACAAGAGCAGG
AATTTCAGGTCAACAAACTGATGAAGAAAATTAAAAAACTGGAGAATGACACCATTTCTAAGCAACTTAC
ATTAGAACAGTTGAGACGGGAGAAGATTGACCTTGAAAATACATTGGAACAAGAACAAGAAGCACTAGTT
AATCGCCTCTGGAAAAGGATGGATAAGCTTGAAGCTGAAAAGCGAATCCTGCAGGAAAAATTAGACCAGC
CCGTCTCTGCTCCACCATCGCCTAGAGATATCTCCATGGAGATTGATTCTCCAGAAAATATGATGCGTCA
CATCAGGTTTTTAAAGAATGAAGTGGAACGGCTGAAGAAGCAACTGAGAGCTGCTCAGTTACAGCATTCA
GAGAAAATGGCACAGTATCTGGAGGAGGAACGTCACATGAGAGAAGAGAACTTGAGGCTCCAGAGGAAGC
TGCAGAGGGAGATGGAGAGAAGAGAAGCCCTCTGTCGACAGCTCTCCGAGAGTGAGTCCAGCTTAGAAAT
GGACGACGAAAGGTATTTTAATGAGATGTCTGCACAAGGATTAAGACCTCGCACTGTGTCCAGCCCGATC
CCTTACACACCTTCTCCGAGTTCAAGCAGGCCTATATCACCTGGTCTATCATATGCAAGTCACACGGTTG
GTTTCACGCCACCAACTTCACTGACTAGAGCTGGAATGTCTTATTACAATTCCCCGGGTCTTCACGTGCA
GCACATGGGAACATCCCATGGTATCACAAGGCCTTCACCACGGAGAAGCAACAGTCCTGACAAATTCAAA
CGGCCCACGCCGCCTCCATCTCCCAACACACAGACCCCAGTCCAGCCACCTCCACCTCCACCT**C**CGCCAC
CCATGCAGCCCACGGTCCCCTCAGCAGCCACCTCGCAGCCTACTCCTTCGCAACATTCGGCGCACACCTC
CTCCCAGCCTTAATGCATGAGCTTAGTCTGAATTTCAAGTTGGGACTCATCCAATGGAGCCGTCTACTCA
ACGCCAAAGGCTTCCTTCTCTGGCATATTTGGATATGACTTATTTGCACTGAGGTTATCTAGGCTTCACT
ATCCATTGTGTTGTAAATGTTTGTCAGAAATGCAGCCAGTGTTGTGGGTCTACAACACTAACCAGACGAC
TTTTTCCATCAGTGTTTTACTTGAATCTTCATGTACGTCCATTCCCTGGCTGGAACCTTCGCTGTTTGGT
ATTTGGTATTTCAGCAGCAGTGTGCAATTTTTGCTTGGCCCAGAGCTTCATTCTCCTGGCTTTTAGGTTT
GTAAAAGAAAAAGGGATATCTTTTTTATATTTTTTTCCATGAATCTGCAGAAAATTACTGAGCTGTTGTT
ACCCTCCTCTCATTATAATAGTGTTTACCAAACATACCAATAATTCAGCACTACAATTCAGACCTTTGAA
AATCTGGCTTTCAGTGTAGAACAGAAAGTTAGATGAATCAGTGCCCAAGACATATTTTCTGTTTAACAGA
ACTTTCTACAGATACATTTTTTACAGGTTATTTTCATTGTGTTATTGACATCCATGTCTCTCGTAAAACA
GATGGCCCAAAGTAATGAATCATGTGGCTGTACCTTCTCCACATAAATGGGATGGATAATTATCGTATAT
TAAGATGTGATTCTCTTTTTTATCCTTAATGTTAATCTACTTAACCTGGCCCCCTCTAA**CATGAGTCGAT**

AAATGTTGTCCTACTCACCGGTGGTTTCAATGGCTAATTAGAATGTGTTATTTGATTTCTGCTGCAGAAG
GCAGTGTGATTGTAACAAAAACAATGCGGCTTCCCCCTTTCGTACTTCATTTGTGTTCTCTTAAAATAGA
GTTTGAACAAATATTTTAAAGGTGCAAAATACCATTAGAAAATACTATTTGAAATGGACATTATCGCATT
ATCTTGGCATAATGGCCAGAAAATATTGTATTGCTTGGCAGAAAAGAAAATAAGGTCTAAAGGAAAGTAG
CACATTAGCATTGATGGCTGTTCATTTCACCCAGTATAAGCAAGTGCAGTGTACAAAGAAGTATATTCTG
AATACATTATTTCCATTCATTTAGCACAAATAAATCATTTGGTTTCACTTTGCAGTGGAAAAAAAAAAAA
AAAAAAAAAAAAAAAAAAAAAAAAAAAAAAAAAAAAAAA

49) Entrez Gene ID 8242 = KDM5C, lysine (K)-specific demethylase 5C = BC054499

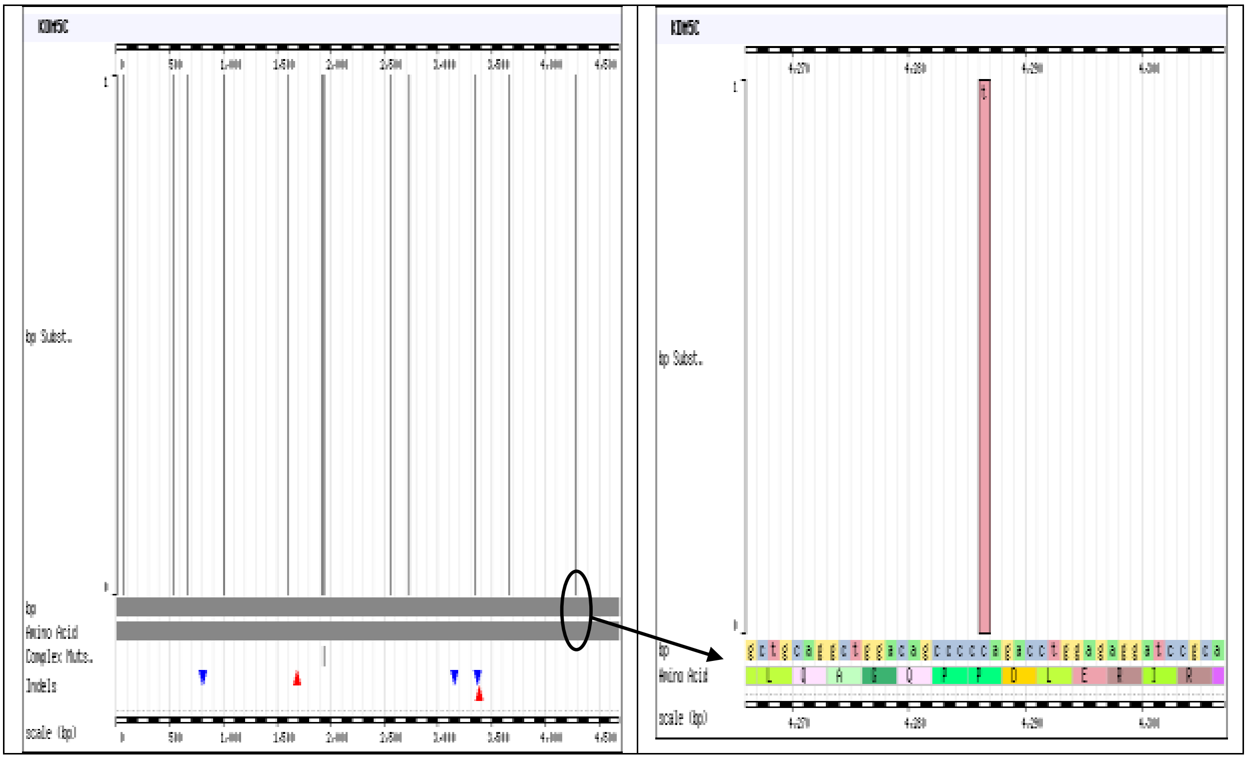

>gi|34194594|gb|BC054499.2| Homo sapiens jumonji, AT rich interactive domain 1C, mRNA (cDNA clone MGC:57577 IMAGE:5492114), complete cds
GGCCGCGGCGTTTGGGAGGTAACTGTGGTGGCGAAGGCTGCGGTAGTGGGGAGGCAACCACACAGTTTGG
AAGAAACGGAGCGGGACGAAGAGGCGGTAGCAGTAGAGTCAGCCTGAGACTCTCAGAGCACGACGGCCAC
ACGCCCCCTTAGGCCCTCGGCGGGCGGCGGCTGCCCCGCTTAGGGCCTAGCCTCCGAGCATTGCCTCGGC
TTCAAACAGCGGCGGCGCCATGAGTCCTTAAGGGCGGTCCAAGCCTCCCGATCCCTGGCCCAGACCTCGG
GCCCACCATGGAGCCGGGGTCCGACGATTTCCTACCGCCACCGGAGTGCCCGGTGTTCGAGCCTAGCTGG
GCCGAGTTCCGAGACCCTCTTGGCTACATCGCGAAAATCAGGCCCATCGCAGAGAAATCGGGCATTTGCA
AGATCCGCCCACCCGCGGACTGGCAGCCACCCTTTGCTGTGGAAGTGGACAACTTCAGGTTTACCCCCCG
AATCCAGAGGCTGAATGAGCTAGAGGCCCAGACGAGAGTGAAACTGAACTACTTGGACCAGATTGCCAAA
TTCTGGGAAATCCAGGGCTCCTCCTTAAAGATTCCCAATGTAGAACGGCGGATCTTGGACCTCTACAGTC
TCAGCAAAATTGTGGTGGAGGAAGGTGGTTATGAAGCTATCTGCAAGGACCGTCGGTGGGCTCGGGTAGC
CCAGCGCCTCAACTATCCACCAGGCAAAAATATTGGCTCCTTGCTACGCTCCCACTACGAACGCATTGTT
TATCCCTATGAAATGTACCAGTCTGGAGCCAACCTTGTGTGTAACACACGTCCATTTGATAATGAGGAGA
AGGACAAGGAATACAAACCCCACAGCATCCCCCTACGACAGTCTGTGCAGCCTTCCAAGTTCAACAGCTA
TGGCCGGCGGGCCAAGAGACTGCAGCCTGATCCGGAACCCACAGAGGAAGACATTGAGAAGAATCCAGAG
CTGAAAAAGCTACAGATCTATGGGGCAGGCCCCAAGATGATGGGCCTGGGCCTCATGGCCAAAGACAAGA
CTCTGCGGAAGAAAGATAAGGAGGGGCCTGAGTGTCCCCCCACAGTAGTGGTGAAGGAGGAGTTAGGTGG
GGATGTGAAGGTGGAGTCAACATCGCCTAAGACCTTCCTGGAGAGCAAGGAGGAGCTGAGTCACAGCCCA
GAACCCTGCACCAAGATGACCATGAGGCTACGGAGGAACCACAGCAATGCCCAGTTTATTGAGTCATATG
TCTGCCGGATGTGTTCTCGAGGGGATGAGGATGACAAGCTCCTGCTGTGTGATGGCTGTGATGACAACTA
CCACATCTTCTGCCTGCTGCCTCCTCTGCCTGAGATCCCCAAGGGTGTCTGGCGGTGCCCAAAGTGTGTC

ATGGCGGAGTGTAAGCGGCCCCCAGAAGCCTTTGGCTTTGAGCAGGCTACCCGGGAATACACTCTGCAGA
GCTTTGGCGAGATGGCCGACTCCTTTAAAGCTGACTACTTCAACATGCCCGTGCATATGGTGCCCACAGA
ACTTGTGGAGAAGGAGTTCTGGAGGCTGGTAAATAGCATTGAGGAAGATGTGACTGTTGAGTATGGAGCT
GACATCCATTCCAAAGAATTTGGCAGCGGTTTCCCTGTCAGTGACAGTAAACGGCACCTAACCCCCGAAG
AGGAGGAGTATGCTACCAGTGGTTGGAACCTAAATGTGATGCCGGTGTTGGAACAGTCTGTACTGTGCCA
CATCAATGCAGATATCTCTGGCATGAAGGTGCCCTGGCTCTACGTGGGCATGGTCTTCTCAGCCTTTTGC
TGGCATATTGAGGATCACTGGAGTTACTCCATTAACTACCTCCACTGGGGTGAGCCGAAGACCTGGTATG
GGGTGCCCTCACTTGCAGCAGAACATTTGGAAGAAGTGATGAAGAAGCTGACACCTGAACTATTTGATAG
CCAGCCTGACCTCCTGCACCAACTTGTCACCCTCATGAATCCCAACACCCTCATGTCCCATGGTGTGCCA
GTTGTCCGCACAAACCAGTGTGCAGGAGAGTTTGTCATCACCTTCCCCCGTGCTTACCACAGCGGCTTCA
ACCAAGGCTACAACTTTGCCGAGGCTGTCAACTTTTGCACTGCTGACTGGTTGCCTGCTGGGCGCCAGTG
CATTGAGCACTACCGCCGGCTCCGGAGATACTGCGTCTTCTCCCATGAGGAGCTTATCTGCAAGATGGCT
GCCTGCCCAGAGAAGCTAGACCTGAACCTGGCGGCAGCTGTGCATAAGGAGATGTTCATCATGGTGCAAG
AAGAGCGGCGTCTACGAAAGGCCCTGCTGGAGAAGGGTATCACAGAGGCTGAGCGAGAGGCTTTCGAGCT
GCTCCCAGATGATGAGCGCCAGTGTATCAAGTGCAAGACTACGTGTTTCCTGTCAGCCCTGGCCTGCTAC
GACTGCCCAGACGGCCTTGTCTGCCTTTCCCACATCAATGATCTCTGCAAGTGCTCCAGTAGCCGGCAGT
ACCTGCGGTATCGGTATACCTTGGATGAGCTTCCTGCCATGCTGCATAAGCTGAAGGTTCGGGCTGAGTC
CTTTGACACCTGGGCCAACAAAGTGCGAGTGGCCCTGGAGGTGGAGGATGGGCGGAAGCGCAGCCTTGAA
GAACTGAGGGCACTAGAGTCTGAAGCCCGTGAGCGGAGGTTTCCTAATAGTGAGCTGCTGCAGCAACTAA
AGAACTGCCTGAGTGAGGCAGAGGCTTGCGTGTCCCGAGCTCTGGGACTGGTCAGCGGCCAGGAAGCTGG
CCCCCACAGGGTGGCTGGTCTACAGATGACCCTGACTGAGCTCCGGGCCTTTCTGGACCAGATGAACAAC
CTGCCTTGCGCCATGCACCAGATTGGGGATGTCAAGGGTGTTCTGGAACAGGTGGAGGCCTACCAGGCTG
AGGCTCGTGAGGCCCTGGCCTCACTGCCCTCCAGTCCAGGGCTACTGCAGTCCCTGTTGGAGAGGGGGCG
GCAGCTGGGGGTGGAGGTGCCTGAGGCCCAGCAGCTCCAGCGGCAGGTGGAACAGGCGCGATGGCTGGAT
GAGGTGAAACGCACACTGGCCCCCTCAGCCCGAAGGGGCACCTTGGCTGTCATGCGAGGACTGTTGGTCG
CGGGTGCCAGTGTAGCCCCTAGCCCTGCTGTGGATAAAGCCCAGGCCGAGCTGCAGGAACTGCTGACCAT
TGCTGAACGCTGGGAGGAGAAAGCCCACCTCTGCCTGGAGGCCAGGCAGAAGCATCCACCAGCCACACTT
GAGGCCATAATCCGTGAAGCGGAAAACATCCCTGTTCACCTGCCCAACATCCAGGCTCTCAAGGAGGCTC
TTGCTAAGGCCCGGGCCTGGATTGCTGATGTTGATGAGATCCAAAATGGTGACCACTACCCCTGCCTGGA
TGACTTGGAGGGCCTAGTAGCTGTGGGCCGGGACCTACCTGTGGGGCTGGAGGAGCTGAGACAGCTAGAG
CTACAGGTACTGACAGCGCACTCCTGGAGGGAGAAGGCCTCCAAGACCTTCCTCAAGAAAAATTCTTGCT
ACACGCTGCTGGAGGTTCTCTGCCCATGTGCAGATGCCGGCTCAGACAGCACCAAGCGCAGCCGGTGGAT
GGAGAAGGAGCTGGGGTTGTACAAATCTGACACAGAGCTGCTGGGGCTGTCTGCGCAGGACCTCAGGGAC
CCAGGCTCTGTGATCGTGGCCTTCAAGGAGGGGGAACAGAAGGAGAAGGAGGGTATCCTGCAGCTGCGTC
GCACCAATTCGGCCAAGCCCAGTCCACTGGCATCATCGAGCACGGCCTCCTCTACAACCTCTATCTGTGT
GTGTGGGCAGGTGCTGGCTGGGGCGGGAGCTCTGCAGTGTGACCTGTGTCAGGACTGGTTCCATGGGCGG
TGTGTGTCAGTGCCTCGCCTCCTCAGCTCTCCGAGGCCCAATCCCACCTCATCCCCACTGCTGGCCTGGT
GGGAATGGGACACCAAATTCCTGTGTCCACTGTGTATGCGCTCAAGGCGCCCGCGCCTGGAGACCATCCT
GGCACTGCTGGTAGCCCTGCAGAGACTGCCTGTGCGGCTGCCCGAGGGCGAGGCCCTGCAGTGCCTCACA
GAGAGGGCCATCAGCTGGCAAGGCCGCGCCAGGCAGGCTCTGGCCTCTGAAGATGTGACTGCTCTTTTGG
GACGGCTGGCTGAGCTCCGCCAACGGCTACAGGCTGAACCTAGACCTGAGGAGCCTCCTAACTACCCTGC
AGCCCCTGCTTCTGACCCCCTCAGAGAGGGCAGTGGCAAGGATATGCCTAAGGTCCAGGGCTTACTGGAG
AATGGAGACAGTGTGACCAGTCCTGAGAAGGTAGCCCCGGAGGAGGGCTCAGGTAAGAGAGATCTGGAGC
TGCTGTCCTCGCTGTTGCCACAGTTGACTGGCCCTGTGTTGGAACTGCCTGAGGCAACCCGGGCCCCCTT
GGAGGAGCTCATGATGGAGGGGGACCTGCTCGAGGTGACCCTGGATGAGAACCACAGCATATGGCAGCTG
CTGCAGGCTGGACAGCCCC**C**AGACCTGGAGAGGATCCGCACACTTCTGGAGCTGGAGAAGGCAGAGCGTC
ACGGGAGTCGGGCTCGGGGCCGGGCCCTGGAGAGGCGGCGGCGGAAGGTGGATCGGGGTGGGGAGGG
CGATGACCCAGCCCGAGAGGAGCTAGAGCCAAAGAGGGTACGGAGCTCAGGGCCAGAGGCTGAGGAGGTC
CAGGAGGAGGAAGAGCTGGAGGAGGAGACTGGGGGTGAGGGCCCCCCTGCACCCATCCCCACCACTGGCA
GCCCCAGCACCCAGGAGAACCAGAATGGCTTGGAACCGGCGGAAGGGACCACTTCAGGCCCCTCGGCCCC
TTTCTCCACTCTGACTCCCCGGCTGCATCTGCCCTGCCCACAGCAGCCGCCTCAGCAACAGTTGTGACAG
TGGCTGAGCCTAGCACAGACCCTGACAGAGACCCCCCTCGGCCTCAAGGATCCTCTTTCTGACCATCAAG
CCTGCTTCTTGGGGGGTGGGCGGGTAGGGGGGGTGGCCATCCCTGCTACCCGCCCACCCCTGAGTCCCTT
GACTTTTGTATTCTGACTCCAAGGTATTGTTCAGACCTCAGCTCCTGGGGGCCGGCCCCTGGAGTCTTCC
CTCCCTGGTAGCCTCTAACCAGCATTCCCAGACACCTGAGGCAGATAGATGGATGGGCTGGTGGGCAGGG
GGGTGGCTGGGGCTGGGCCATCACCATTCCAGAGACAAGGCCAGTGTATATGCAAACTGGGGGACTCTCC
TCCCTTCTCTCCCCAGTTCTGGTCCTGGCCAGGCCATGCTACACTAACCCCTGCCCCCACTCTCCTCCCC
TCTTTTCCTTCCTTCCTACCCCCTTCTCCCTCTCCCTTCCCCTGACTGTTCCACCCAGGAGGAGGAAACT
TCACATAGCCGTGCTCACAGTTTTTTATTTTAAAGGAATTTGGCTGGGGAGCTGAACAGGGCTCCCTGTG
ATCTGAAGAAAGCTTTTGGTGCTTGTCCTCACAACCACCTCAGTCCTCCCTCCCTGTCCTCCCCTGTCTC
CTTTCCTCCTCCTGGGTT**CATGTTGTAATAAAAGAAGAT**TGTTGGTGTGTAATTAATTTGTTCAAAAAAA

AAAAAAAA

50) Entrez Gene ID 9709 = HERPUD1, homocysteine-inducible, endoplasmic reticulum stress-inducible, ubiquitin-like domain member 1 = AF055001

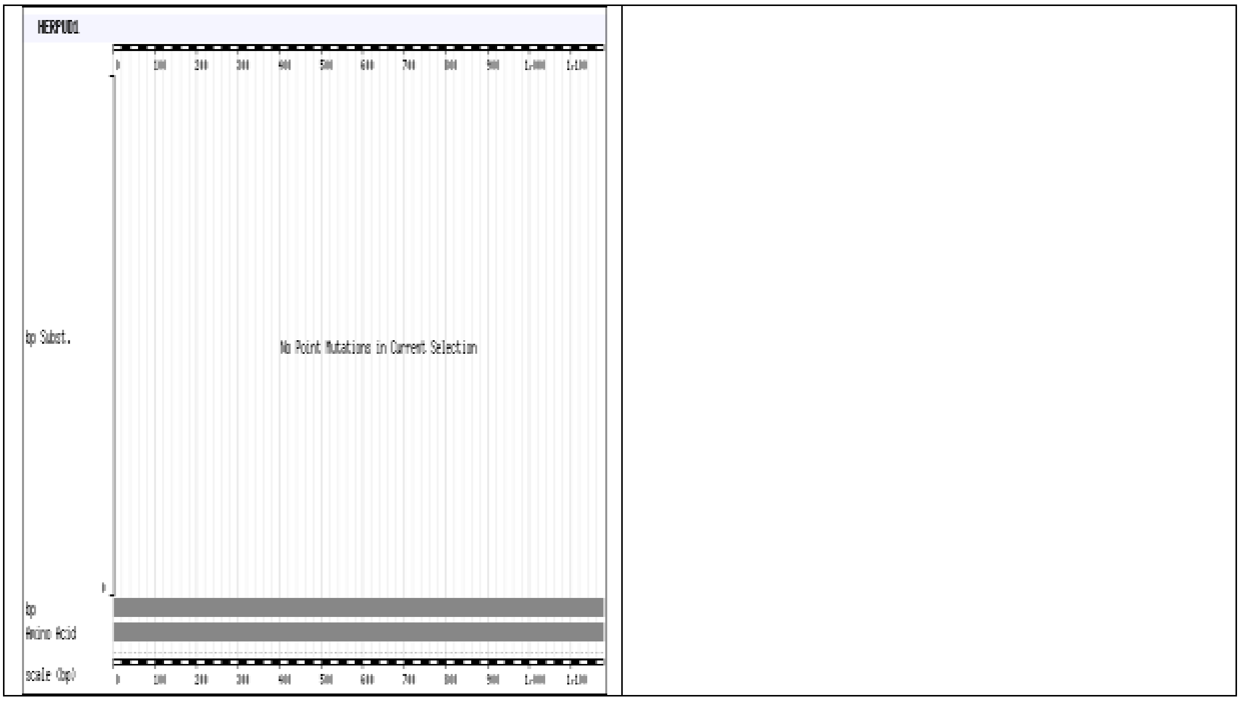

51) Entrez Gene ID 9968 = MED12, mediator complex subunit 12 = AF071309

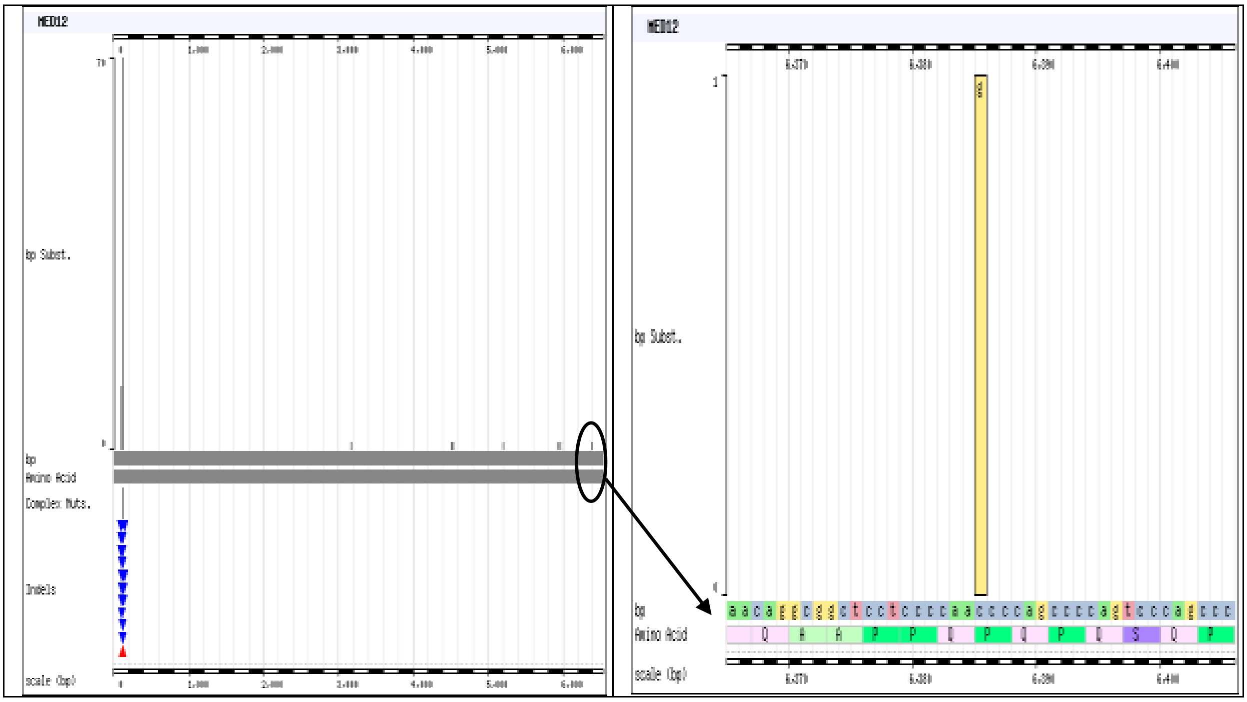

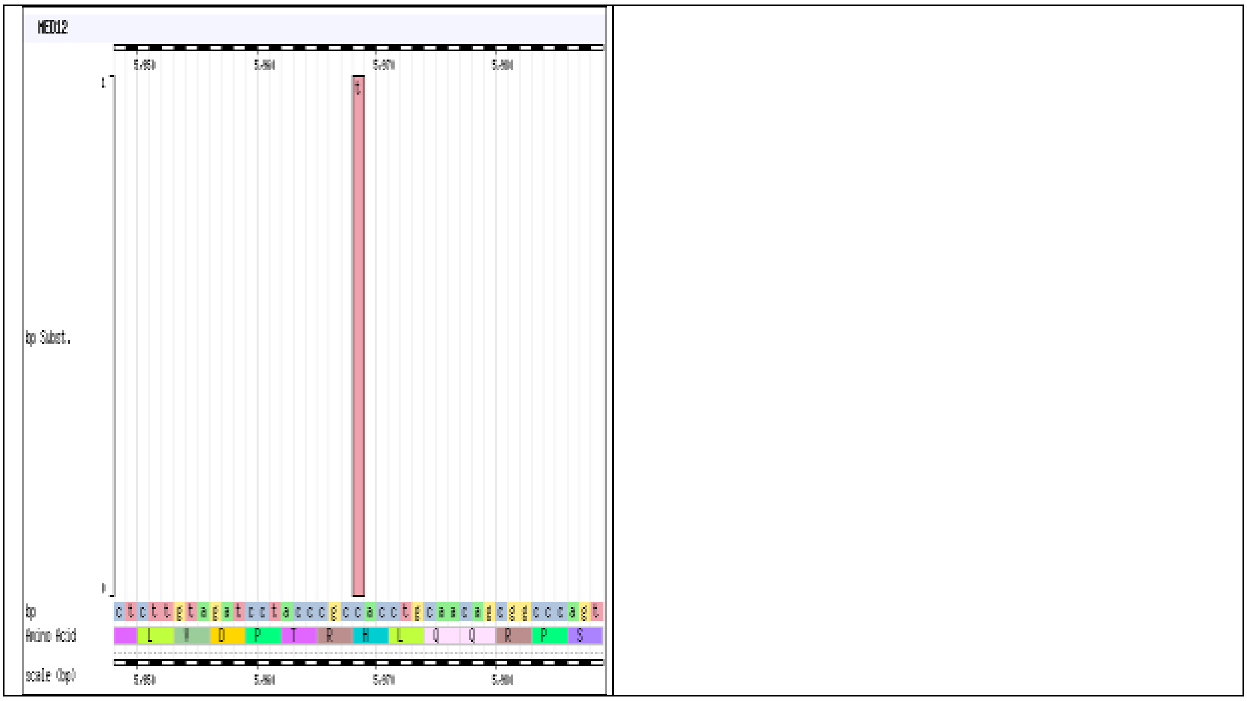

>gi|3426319|gb|AF071309.1| Homo sapiens OPA-containing protein mRNA, complete cds
GCGGCCGCGTCGACGGCGGCCTTCGGGATCTTGAGCTACGAACACCGGCCCCTGAAGCGGCCGCGGCTGG
GGCCTCCCGATGTTTACCCTCAGGACCCCAAACAGAAGGAGGATGAACTGACGGCCTTGAATGTAAAACA
AGGTTTCAATAACCAGCCTGCTGTCTCTGGGGATGAGCATGGCAGTGCCAAGAACGTCAGCTTCAATCCT
GCCAAGATCAGTTCCAACTTCAGCAGCATTATTGCAGAGAAATTACGTTGTAATACCCTTCCTGACACTG
GTCGCAGGAAGCCCCAAGTGAACCAGAAGGATAACTTCTGGCTGGTGACTGCACGATCCCAGAGTGCCAT
TAACACTTGGTTCACTGACTTGGCTGGCACCAAGCCACTCACGCAACTAGCCAAAAAGGTCCCCATTTTC
AGTAAGAAGGAAGAGGTGTTTGGGTACTTAGCCAAATACACAGTGCCTGTGATGCGGGCTGCCTGGCTCA
TTAAGATGACCTGTGCCTACTATGCAGCAATCTCTGAGACCAAGGTTAAGAAGAGACATGTTGACCCTTT
CATGGAATGGACTCAGATCATCACCAAGTACTTATGGGAGCAGTTACAGAAGATGGCTGAATACTACCGG
CCAGGGCCTGCAGGAAGTGGGGGCTGTGGTTCCACGATAGGGCCCTTGCCCCATGATGTAGAGGTGGCAA
TCCGGCAGTGGGATTACACCGAGAAGCTGGCCATGTTCATGTTTCAGGATGGAATGCTGGACAGACATGA
GTTCCTGACCTGGGTGCTTGAGTGTTTTGAGAAGATCCGCCCTGGAGAGGATGAATTGCTTAAACTGCTG
CTGCCTCTGCTTCTCCGATACTCTGGGGAATTTGTTCAGTCTGCATACCTGTCCCGCCGGCTTGCCTACT
TCTGTACACGGAGACTGGCCCTGCAGCTGGATGGTGTGAGCAGTCACTCATCTCATGTTATATCTGCTCA
GTCAACAAGCACGCTACCCACCACCCCTGCTCCTCAGCCCCCAACTAGCAGCACACCCTCGACTCCCTTT
AGTGACCTGCTTATGTGCCCTCAGCACCGGCCCCTGGTTTTTGGCCTCAGCTGTATCCTACAGACCATCC
TCCTGTGCTGTCCTAGTGCCTTGGTTTGGCACTACTCACTGACTGATAGCAGAATTAAGACCGGCTCACC
ACTTGACCACTTGCCTATTGCCCCGTCCAACCTGCCCATGCCAGAGGGTAACAGTGCCTTCACTCAGCAG
GTCCGTGCAAAGTTGCGGGAGATCGAGCAGCAGATCAAGGAGCGGGGACAGGCAGTTGAAGTTCGCTGGT
CTTTCGATAAATGCCAGGAAGCTACTGCAGGCTTCACCATTGGACGGGTACTTCATACTTTGGAAGTGCT
GGACAGCCATAGTTTTGAACGCTCTGACTTCAGCAACTCTCTTGACTCCCTTTGTAACCGAATCTTTGGA
TTGGGACCTAGCAAGGATGGGCATGAGATCTCCTCAGATGATGATGCTGTGGTGTCATTGCTATGTGAAT
GGGCTGTCAGCTGCAAGCGTTCTGGTCGGCATCGTGCTATGGTGGTAGCCAAGCTCCTGGAGAAGAGACA
GGCGGAGATTGAGGCTGAGCGTTGTGGAGAATCAGAAGCCGCAGATGAGAAGGGTTCCATCGCCTCTGGC
TCCCTTTCTGCTCCCAGTGCTCCCATTTTCCAGGATGTCCTCCTGCAGTTTCTGGATACACAGGCTCCCA
TGCTGACGGACCCTCGAAGTGAGAGTGAGCGGGTGGAATTCTTTAACTTAGTACTGCTGTTCTGTGAACT
GATTCGACATGATGTTTTCTCCCACAACATGTATACTTGCACTCTCATCTCCCGAGGGGACCTTGCCTTT
GGAGCCCCTGGTCCCCGGCCTCCCTCTCCCTTTGATGATCCTGCCGATGACCCAGAGCACAAGGAGGCTG
AAGGCAGCAGCAGCAGCAAGCTGGAAGATCCAGGGCTCTCAGAATCTATGGACATTGACCCTAGTTCCAG
TGTTCTCTTTGAGGACATGGAGAAGCCTGATTTCTCATTGTTCTCCCCTACTATGCCCTGTGAGGGGAAG
GGCAGTCCATCCCCTGAGAAGCCAGATGTCGAGAAGGAGGTGAAGCCCCCACCCAAGGAGAAGATTGAAG
GGACCCTTGGGGTTCTTTACGACCAGCCACGACACGTGCAGTACGCCACCCATTTTCCCATCCCCCAGGA
GGAGTCATGCAGCCATGAGTGCAACCAGCGGTTGGTCGTACTGTTTGGGGTGGGAAAGCAGCGAGATGAT
GCCCGCCATGCCATCAAGAAAATCACCAAGGATATCTTGAAGGTTCTGAACCGCAAAGGGACAGCAGAAA
CTGACCAGCTTGCTCCTATTGTGCCTCTGAATCCTGGAGACCTGACATTCTTAGGTGGGGAGGATGGGCA

GAAGCGGCGACGCAACCGGCCTGAAGCCTTCCCCACTGCTGAAGATATCTTTGCTAAGTTCCAGCACCTT
TCACATTATGACCAACACCAGGTCACGGCTCAGGTCTCCCGGAATGTTCTGGAGCAGATCACGAGCTTTG
CCCTTGGCATGTCATACCACTTGCCTCTGGTGCAGCATGTGCAGTTCATCTTCGACCTCATGGAATATTC
ACTCAGCATCAGTGGCCTCATCGACTTTGCCATTCAGCTGCTGAATGAACTGAGTGTAGTTGAGGCTGAG
CTGCTTCTCAAATCCTCGGATCTGGTGGGCAGCTACACTACTAGCCTGTGCCTGTGCATCGTGGCTGTCC
TGCGGCACTATCATGCCTGCCTCATCCTCAACCAGGACCAGATGGCACAGGTCTTTGAGGGGCTGTGTGG
CGTCGTGAAGCATGGGATGAACCGGTCCGATGGCTCCTCTGCAGAGCGCTGTATCCTTGCTTATCTCTAT
GATCTGTACACCTCCTGTAGCCATTTAAAGAACAAATTTGGGGAGCTCTTCAGCGACTTTTGCTCAAAGG
TGAAGAACACCATCTACTGCAACGTGGAGCCATCGGAATCAAATATGCGCTGGGCACCTGAGTTCATGAT
CGACACTCTAGAGAACCCTGCAGCTCACACCTTCACCTACACGGGGCTAGGCAAGAGTCTTAGTGAGAAC
CCTGCTAACCGCTACAGCTTTGTCTGCAATGCCCTTATGCACGTCTGTGTGGGGCACCATGATCCCGATA
GGGTGAATGACATCGCAATCCTGTGTGCAGAGCTGACCGGCTATTGCAAGTCACTGAGTGCAGAATGGCT
AGGAGTGCTTAAGGCCTTGTGCTGCTCCTCTAACAATGGCACTTGTGGTTTCAACGATCTCCTCTGCAAT
GTTGATGTCAGTGACCTATCTTTTCATGACTCGCTGGCTACTTTTGTTGCCATCCTCATCGCTCGGCAGT
GTTTGCTCCTGGAAGATCTGATTCGCTGTGCTGCCATCCCTTCACTCCTTAATGCTGCTTGTAGTGAACA
GGACTCTGAGCCAGGGGCCCGGCTTACCTGCCGCATCCTCCTTCACCTTTTCAAGACACCGCAGCTCAAT
CCTTGCCAGTCTGATGGAAACAAGCCTACAGTAGGAATCCGCTCCTCCTGCGACCGCCACCTGCTGGCTG
CCTCCCAGAACCGCATCGTGGATGGAGCCGTGTTTGCTGTTCTCAAGGCTGTGTTTGTACTTGGGGATGC
GGAACTGAAAGGTTCAGGCTTCACTGTGACAGGAGGAACAGAAGAACTTCCAGAGGAGGAGGGAGGAGGT
GGCAGTGGTGGTCGGAGGCAGGGTGGCCGCAACATCTCTGTGGAGACAGCCAGTCTGGATGTCTATGCCA
AGTACGTGCTGCGCAGCATCTGCCAACAGGAATGGGTAGGAGAACGTTGCCTTAAGTCTCTGTGTGAGGA
CAGCAATGACCTGCAAGACCCAGTGTTGAGTAGTGCCCAGGCGCAGCGCCTCATGCAGCTCATTTGCTAT
CCACATCGACTGCTGGACAATGAGGATGGGGAAAACCCCCAGCGGCAGCGCATAAAGCGCATTCTCCAGA
ACTTGGACCAGTGGACCATGCGCCAGTCTTCCTTGGAGCTGCAGCTCATGATCAAGCAGACCCCTAACAA
TGAGATGAACTCCCTCTTGGAGAACATCGCCAAGGCCACAATCGAGGTTTTCCAACAGTCAGCAGAGACA
GGGTCATCTTCTGGAAGTACTGCAAGCAACATGCCCAGCAGCAGCAAGACCAAGCCTGTGCTCAGCTCTC
TAGAGCGCTCTGGTGTATGGCTGGTGGCCCCCCTCATTGCTAAACTGCCCACCTCAGTCCAGGGACATGT
GTTAAAGGCTGCTGGGGAAGAATTGGAGAAGGGTCAGCACCTGGGTTCCTCTTCACGCAAAGAACGTGAT
CGACAAAAGCAGAAGAGCATGTCCCTATTGAGCCAGCAGCCCTTCTTATCGCTGGTGCTAACATGTCTGA
AAGGGCAGGATGAACAACGCGAGGGACTCCTTACCTCCCTCTACAGCCAGGTGCACCAGATTGTGAATAA
TTGGCGAGATGACCAGTACTTAGATGATTGCAAACCAAAGCAGCTTATGCATGAGGCACTCAAACTGCGG
CTCAACCTGGTGGGGGGCATGTTTGACACGGTGCAGCGCAGCACCCAGCAGACCACGGAGTGGGCCATGC
TCCTCCTGGAGATCATCATCAGCGGCACTGTCGACATGCAGTCCAACAATGAGCTCTTCACTACTGTGTT
GGACATGCTGAGCGTGCTCATCAATGGGACATTGGCTGCAGACATGTCTAGCATCTCGCAAGGTAGCATG
GAGGAAAACAAGCGTGCATACATGAACCTGGCGAAGAAGTTGCAGAAGGAGTTGGGGGAGCGCCAGTCAG
ACAGTCTGGAAAAGGTTCGCCAGCTGCTGCCACTGCCCAAGCAGACCCGAGATGTCATCACGTGTGAGCC
ACAGGGCTCCCTTATCGATACCAAGGGCAACAAGATTGCTGGCTTCGATTCCATCTTCAAGAAGGAGGGT
CTACAGGTTTCCACCAAACAGAAGATCTCGCCCTGGGATCTTTTTGAGGGGTTGAAGCCGTCAGCACCAC
TCTCTTGGGGCTGGTTTGGAACAGTCCGAGTGGACCGGCGAGTGGCTCGAGGAGAGGAGCAGCAGCGGTT
GCTGCTCTACCACACACACCTGAGGCCCCGGCCCCGCGCCTATTACCTGGAGCCACTGCCACTGCCCCCA
GAAGATGAGGAGCCGCCTGCTCCTACCCTGCTAGAGCCTGAGAAAAAGGCTCCAGAGCCCCCCAAAACTG
ACAAACCGGGGGCTGCTCCACCCAGTACTGAGGAACGCAAGAAGAAGTCCACCAAGGGCAAGAAACGCAG
CCAGCCAGCTACCAAGACAGAGGACTATGGAATGGGCCCGGGTCGGAGCGGCCCTTATGGTGTGACAGTG
CCTCCGGACCTCCTGCACCACCCAAACCCTGGTTCTATAACACACCTTAACTACAGGCAAGGCTCCATAG
GCCTGTACACCCAGAACCAGCCACTACCTGCAGGTGGCCCTCGTGTGGACCCATACCGTCCTGTGCGCTT
ACCAATGCAGAAGCTGCCCACCCGACCAACTTACCCTGGAGTGCTGCCCACAACCATGACTGGCGTCATG
GGTTTAGAACCCTCCTCTTATAAGACCTCTGTGTACCGGCAGCAGCAACCTGCGGTGCCCCAAGGACAGC
GCCTTCGCCAACAGCTCCAGAGTCAGGGCATGTTGGGACAGTCATCTGTCCATCAGATGACTCCCAGCTC
TTCCTACGGTTTGCAGACTTCCCAGGGCTATACTCCTTATGTTTCTCATGTGGGATTGCAGCAACACACA
GGCCCTGCAGGTACCATGGTGCCCCCCAGCTACTCCAGCCAGCCTTACCAGAGCACCCACCCTTCTACCA
ATCCTACTCTTGTAGATCCTACCCGC**C**ACCTGCAACAGCGGCCCAGTGGCTATGTGCACCAGCAGGCCCC
CACCTATGGACATGGACTGACCTCCACTCAAAGGTTTTCACACCAGACACTGCAGCAGACACCCATGATA
AGTAC**CATGACTCCAATGAGTGCCCA**GGGCGTCCAGGCAGGCGTCCGTTCAACAGCCATCCTACCTGAGC
AGCAGCAGCAGCAGCAACAGCAGCAACAGCAACAGCAGCAGCAGCAGCAACAGCAACAGCAGCAGCAGCA
GCAGCAGTACCACATCCGGCAGCAGCAGCAGCAGCAGATCCTGCGGCAGCAGCAGCAACAGCAACAGCAG
CAGCAGCAGCAGCAGCAACAGCAACAGCAGCAGCAGCAACAGCAACAACAGCAACACCAGCAGCAACAGC
AGCAACAGGCGGCTCCTCCCCAA**C**CCCAGCCCCAGTCCCAGCCCCAGTTCCAGCGCCAGGGGCTTCAGCA
GACCCAGCAGCAGCAACAGACAGCAGCTTTGGTCCGGCAACTTCAACAACAGCTCTCTAATACCCAGCCA
CAGCCCAGTACCAACATATTTGGACGCTACTGAGCCACCTGGAGGAACTGCTTGTGCACTGGATGTGGCC
CCACCCTTTCCTCTTAATTCCCAATCCCATTCCTGGGCTAGCACCAGTAGTGGTTGGGGCCCTCCCCTCA
GGCTCCATTTTTAATAAGTTTTTAGTATTTTTGTTAATGTGAGGCATTGAGCTGTTGGGTTTTGTATATT

ATTTATATAGAGACCCCAGAGCTGTTGCACCCAATACACAGAGCTTCTTTGCAAAAAAAAAAAAAAAA

52) Entrez Gene ID 10006 = ABI1, abl-interactor 1 = AF006516

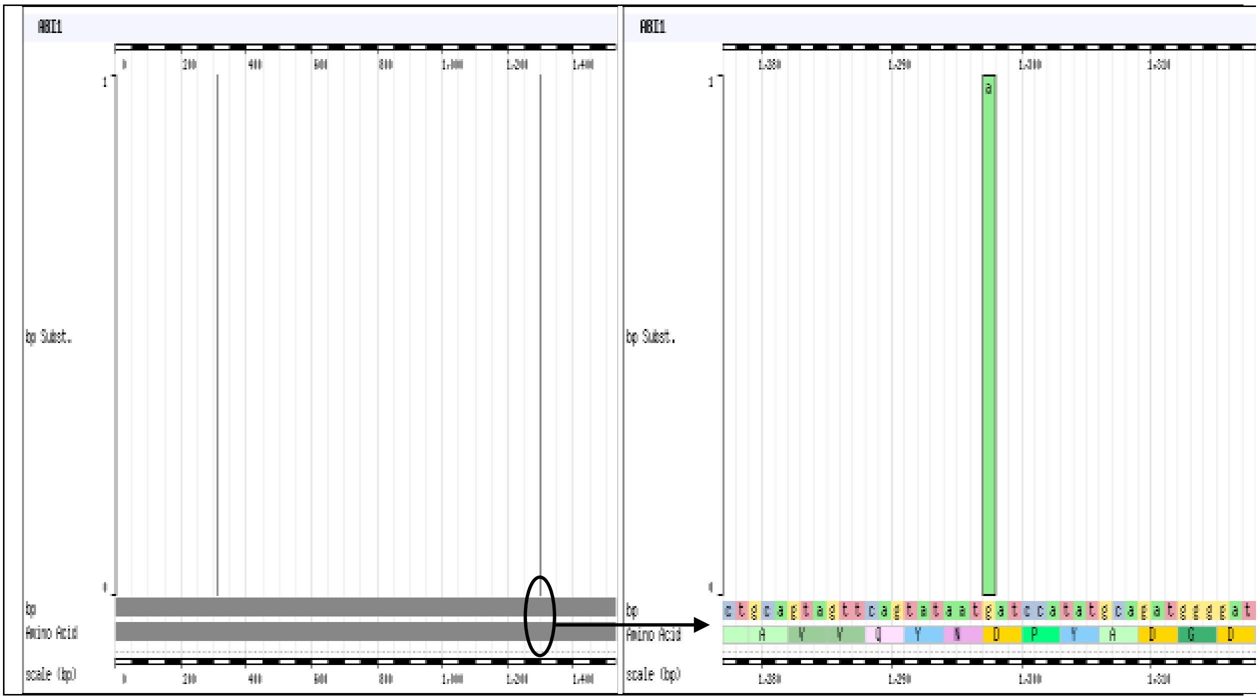

>gi|2245670|gb|AF006516.1| Homo sapiens eps8 binding protein e3B1 mRNA, complete cds
AGCCTCGGCGGACCTTGCTGCCTCTGTCTCTTTAACGCGAGAGGAAGCGATGCAGAGGGGTGGAAAATGG
CAGAGCTGCAGATGTTACTAGAGGAGGAGATCCCGTCTGGCAAGAGGGCGCTGATCGAGAGTTACCAGAA
CCTGACTCGGGTGGCAGACTACTGTGAAAACAACTACATACAGGCTACAGACAAGAGAAAAGCTTTAGAG
GAGACCAAAGCCTATACAACCCAATCTCTAGCTAGTGTTGCTTATCAAATAAATGCATTGGCCAACAATG
TACTCCAGTTGCTGGATATCCAAGCCTCTCAGCTTCGGAGAATGGAGTCTTCCATCAATCATATCTCACA
GACTGTGGATATTCATAAGGAGAAAGTGGCACGAAGAGAGATTGGTATTTTGACAACAAATAAGAATACA
TCAAGAACTCACAAAATAATAGCACCTGCGAATATGGAGCGCCCTGTAAGGTATATTCGGAAACCTATCG
ATTACACAGTTCTGGATGATGTGGGCCATGGTGTCAAGTGGCTAAAAGCCAAGCATGGAAATAACCAGCC
TGCAAGAACTGGCACACTGTCGAGAACAAATCCTCCTACTCAGAAACCGCCAAGTCCTCCCATGTCAGGC
CGGGGAACACTGGGACGGAATACTCCTTATAAAACCCTGGAACCTGTTAAACCCCCAACAGTTCCTAATG
ACTATATGACCAGTCCTGCTAGGCTTGGAAGTCAGCATAGTCCAGGCAGGACAGCATCTTTAAATCAGAG
ACCAAGGACACACAGTGGAAGTAGTGGAGGAAGTGGAAGTCGAGAAAACAGTGGTAGCAGTAGTATTGGC
ATTCCCATTGCTGTGCCTACACCTTCGCCACCCACTATTGGACCAGCCCCGGGCTCAGCTCCTGGTTCCC
AGTATGGCACAATGACCAGGCAGATATCTCGACACAACTCTACTACTTCTTCGACATCTTCTGGTGGATA
CAGACGAACTCCCTCTGTGACTGCTCAATTTTCTGCTCAGCCTCATGTTAATGGAGGTCCACTTTATTCT
CAAAATTCAATTTCTATTGCTCCACCCCCTCCCCCTATGCCTCAGTTGACTCCACAGATACCTCTCACAG
GCTTCGTGGCCAGGGTGCAGGAAAACATTGCTGATAGTCCAACTCCACCGCCACCACCTCCACCAGATGA
CATTCCCATGTTTGATGACTCTCCACCTCCCCCACCACCACCACCAGTGGATTATGAAGATGAGGAGGCT
GCAGTAGTTCAGTATAAT**G**ATCCATATGCAGATGGGGATCCTGCTTGGGCCCCCAAGAATTATATTGAGA
AAGTTGTTGCAATATATGATTATACAAAAGACAAGGATGATGAGCTGTCATTTATGGAGGGTGCAATCAT
TTATGTTATAAAGAAGAATGATGATGGCTGGTATGAAGGAGTCTGCAATCGAGTGACTGGTCTGTTCCCT
GGGAACTATGTTGAATCAATCATGCACTATACTGATTAATTTTTTTTTTTCTTTTGAAGTAGATTCTTAT
TACTCAGTCATACTGTGGGACTATTATGGTTAACAGAACTGTCTTAATATGTTTTAAAATGTGCCCATAT
TTTCAGAACATGCTGTTTTATTGGTAAATTGAATGTCTACCTGTAAGCATAAATCTTTGAGGCAGTTTAT
GTATTGCTGAATAGCAATTTATACAAGAAGCTGTCCATAACTGATTATGCTTATGTACTTACTTACACAT
TTTTAACTTTATGACCAGCCTAAATATTCTGGGGGAAGTGGGGTATAATATTTAACGAATCATGATTCAG
ATTGTACCATTACATGTTTCAGTGCAGCATGGTTACTAACGCTATGTCAGACTAATATTAAAATCAGAAA
ATTTAAATGCTGGTGCTGGTCAGACTTTTTTTGTTAGATTCTCTCATTTAAAAAAAATACTGTTTGTTTA

AAGCATGCATAAAAATTTATGTATTGAAATATACTTAAAAATTCAAGATGCTTCCCATTTGTGTAATATT
TACCTGGAGGACTCGTACTTAGGTGTCTTAACGTGAATTGAGTCTCCAAGGTCTCCATGTGAAACAAAAG
CAGCAAAAAGAGAATTATCTGTAATGTTGTAATTTGTACCTAAGTTTTTTAATGAGTGAAATTTGCATTA
TAAACTTTTTCCATTCATAAATACATAAGTGAACCAAAGGTTTTTGTCCTTTCCTTCACTGATTTGCTTT
AAAAAAAATAAAAGATAATGATTTATTGCAGAATTATGATTCTATTTTCTCAATATGTTAACTTGGAAAA
AAATTTTAGCCTTATCTTAATCTGTCCCAACAGCAATGTGACGGATTTTTGCAGATTCAAAATCTGCAAT
GGTTATTTACAAGTCAATCTACTGAATTCCTTTTTTAAATAATCTTTTGAAACTAAGAAAATGTGTCAAA
TTGTGTGCATTCATTCTGTAGGTAAAATTCTTAAGGATTGCCATGTCAGTCCTTCAGTTGTACAGTGAAC
TGTTGTACACTGGTTCAATGAAAGGAAGTTAAAAGTACCTTTTACATATTGTAAAAGTGGATACAGTTGA
TTTGTGAGTAGGCACTCTTTAATCCATTACCTGGCACTAGCAACATTAGAATTTTAAAATAAAATAATTG
GGAAAGAAGGTGGGTCATGTATTAATCAGTGAACAGAGATTTACCTAACCAACAGACTTGGATTGTCTTT
TGACATAATCAAAATGCAACA**CATGCACTTTGTGTGTCTCCT**CTTAATTGAAGGGAGGGCTGAGGGATGT
TTTCTCTTCTTGTCTTGTGTATAATTCTCTATTGCTTAGGATATTAAAGTAGAGCACTCAAGTGTGGGTT
TCTGTGTTATTGAGGATTTGTTTGGAATTCAAATTACAGTTATGTACTGGATGCTACAGACTTATAACAG
CATAGTGAATGGTAAGACTAGTGCAAAACAGTTATTTCTGAAAATTAAAGACCATTATTGCTACCAAATC
AATGTGACTATTTCATATGCATTTTGCCTTTGTTAATTTTAAACAAACAAAGTATCATTAGTGATCAGCT
AGCTACCTTCTACTTTCCATTTTTCAAGTGGATTGTTCTCTTAATTAGTATATAACCTGTTGTCTAAATT
TTATGTACAGTCTTTTATAATAAACCATTCTCCTATATGAAAAAAAAAAAAAAAAAAAAAAAAAAAAAAA
AAAAAAAAAAAAA

53) Entrez Gene ID 10142 = AKAP9, A kinase (PRKA) anchor protein (yotiao) 9 = BC027455

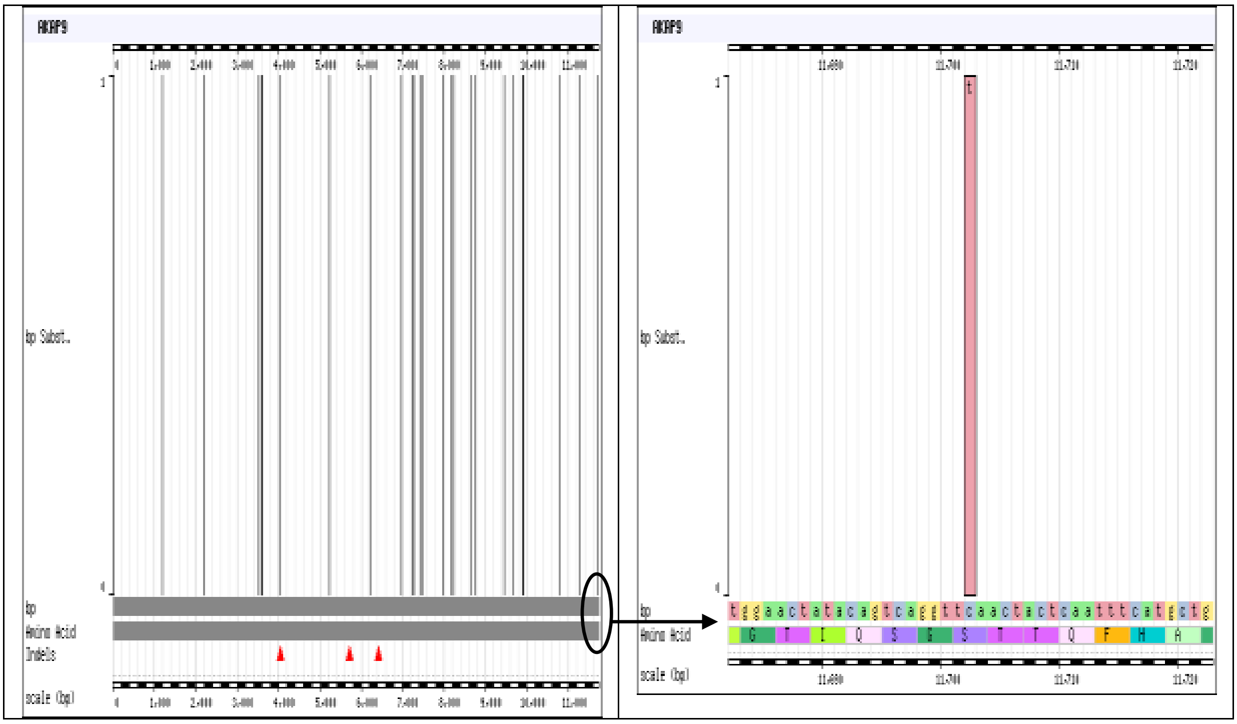

>gi|20073238|gb|BC027455.1| Homo sapiens A kinase (PRKA) anchor protein (yotiao) 9, mRNA (cDNA clone IMAGE:4510491), with apparent retained intron
CCCAGCAAAAAATAGAATCACAGAGAATGCTATATGATGCCCAGTTGTCAGAAGAACAAGGTCGAAACTT
AGAGCTTCAGGTACTTCTTGAATCTGAGAAAGTTCGAATTCGGGAAATGAGTAGTACCCTAGATAGGGAG
CGGGAATTGCACGCACAGCTGCAGAGCAGTGATGGTACTGGACAGTCTCGGCCACCCTTGCCCTCAGAGG
ACCTACTGAAAGAGCTGCAGAAACAGCTAGAGGAAAAACACAGTCGCATAGTAGAATTGTTAAATGAGAC
TGAAAAATATAAACTGGATTCTTTGCAAACACGACAGCAAATGGAAAAAGATAGGCAGGTTCACAGGAAA
ACACTGCAGACAGAACAGGAGGCCAACACTGAGGGACAGAAAAAAATGCATGAGCTCCAGTCCAAAGTGG
AAGATCTTCAGCGCCAGCTGGAAGAGAAAAGACAACAAGTTTATAAGTTAGACCTTGAAGGACAGCGACT
ACAAGGAATCATGCAGGAATTCCAGAAGCAAGAACTAGAACGAGAAGAAAAACGAGAAAGTAGAAGAATT
CTGTATCAGAACCTTAATGAGCCAACCACGTGGAGCTTAACCAGTGATCGAACTAGAAATTGGGTTCTTC

AACAGAAAATAGAAGGAGAAACAAAAGAATCAAACTACGCTAAATTGATTGAAATGAATGGAGGAGGAAC
CGGCTGTAATCATGAATTAGAAATGATCAGACAAAAGCTTCAATGTGTAGCTTCAAAACTACAGGTTCTA
CCCCAGAAAGCCTCTGAGAGACTACAGTTTGAAACAGCAGATGATGAAGATTTCATTTGGGTTCAGGAAA
ATATTGATGAAATTATTTTACAACTACAGAAATTAACTGGCCAGCAAGGTGAAGAGCCCAGCTTGGTGTC
CCCAAGTACTTCTTGTGGCTCATTGACTGAAAGACTACTGAGACAAAATGCTGAGCTGACAGGGCATATC
AGTCAACTGACTGAAGAGAAGAATGACTTAAGGAACATGGTTATGAAGCTGGAAGAGCAGATCAGGTGGT
ATCGACAGACAGGAGCTGGTAGAGATAATTCTTCCAGGTTTTCATTGAATGGTGGTGCCAACATTGAAGC
CATCATTGCCTCTGAAAAAGAAGTATGGAACAGAGAAAAATTGACTCTCCAGAAATCTTTGAAAAGGGCA
GAGGCTGAAGTATACAAACTGAAAGCTGAACTAAGAAATGACTCTTTACTTCAAACTCTGAGCCCTGATT
CTGAACATGTCACTTTAAAGAGAATTTATGGTAAATACTTGAGGGCAGAAAGTTTTCGAAAGGCTCTCAT
TTACCAGAAGAAATACCTGCTGCTGTTACTGGGTGGGTTCCAGGAATGTGAAGATGCCACCTTGGCCCTG
CTTGCCCGGATGGGGGGGCAGCCAGCTTTCACGGATCTAGAGGTGATCACCAATCGCCCAAAGGGCTTCA
CCAGGTTTCGGTCGGCCGTCAGAGTATCCATTGCAATTTCCAGAATGAAATTTTTGGTTCGACGGTGGCA
TCGAGTCACAGGTTCTGTTTCCATCAATATTAACAGAGATGGCTTTGGACTGAATCAAGGTGCAGAAAAG
ACTGACTCATTTTATCATTCTTCTGGTGGGCTGGAGTTATATGGAGAACCAAGACATACTACGTATCGCT
CAAGATCAGATCTGGACTATATTAGGTCCCCTTTACCATTTCAGAATAGGTACCCAGGCACTCCAGCTGA
TTTCAATCCTGGTTCTTTAGCATGTTCTCAGCTTCAGAATTACGATCCTGACAGAGCCCTAACAGATTAT
ATCACTCGGCTAGAGGCACTGCAAAGACGACTTGGAACTATACAGTCAGGTTCAACTACTCAATTTCATG
CTGGCATGAGAAGATAATCCTTTGAAACATCATTAATTGAAGTGATTTTAAATAGATTTCCTTTTGTAAA
TCAATGGTTCTTTTGTGCTTTTGTATTGTGAATATTCAATGGGACCAATATGAACACAGCTTATGATTGT
ATACAAATCCCTTGCCAGCACATGAAAACAAACTGGAATTTGTATATATAAGCATTGTGTATGTATTCAT
GCACATAATTATTGAATTACCTGTATATTTGTGGAATGCTAATTTAAAACATTAAATTATAAACCTTGTG
TATTTATCAAATGGGTGAAAAGATTAAACTTTTACGCATTACAATACTGCTGAATGTGTAGCTCGAGGTG
TCCTGCACTTTTCTTATAAGGCTACTGAAGTTA**CATGTTTTGCCTAATATATTC**TACTGGTGATGAAGAC
AGATAATATCACTTGTAGAGACCTATTTTTGTATAATGGTAGAAGTTTTGAATTTTATGGGGTATTTTGT
CAAGTACTGAAATAAAAATGACTTCACCATTTTCAAAAAAAAAAAAAAAAAAAA

54) Entrez Gene ID 10342 = TFG, TRK-fused gene = BC001483

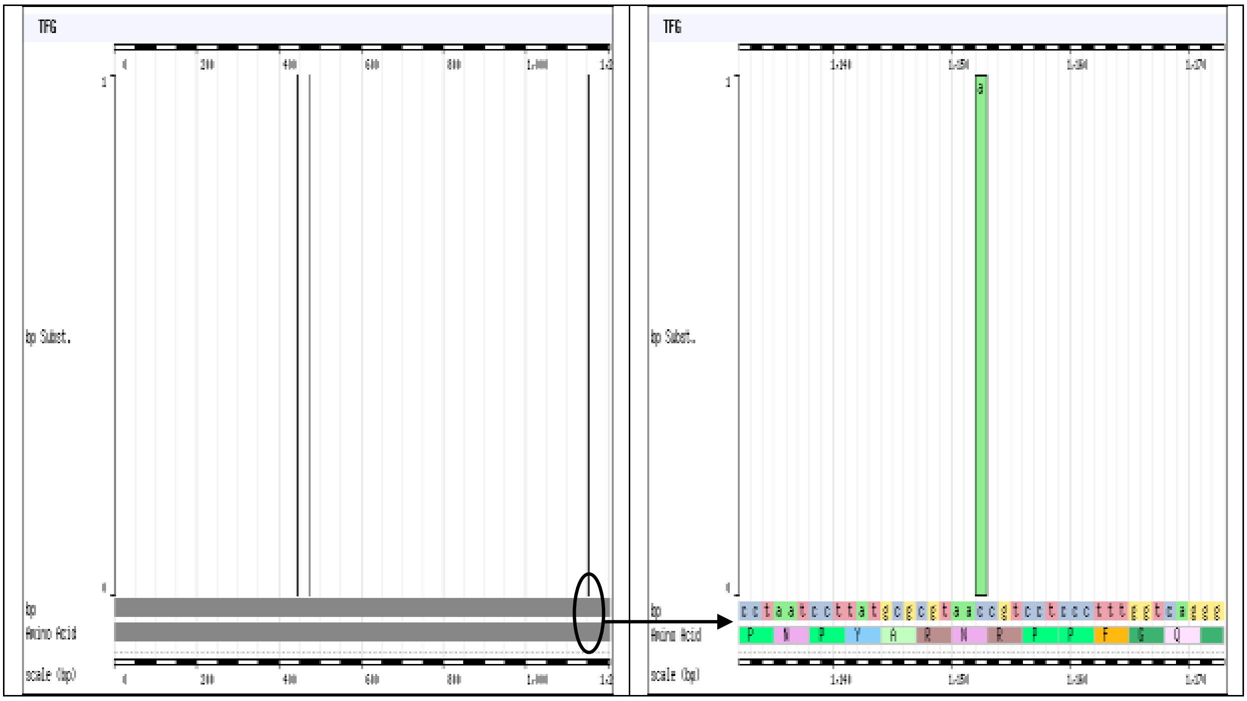

>gi|33876274|gb|BC001483.2| Homo sapiens TRK-fused gene, mRNA (cDNA clone MGC:1364 IMAGE:3507440), complete cds
GTTGGGTCAGCGCGATTGGCCGGGGCCCGCGCGAGCCTGCGAGCGAGTCTTTCTCTAGAGTTGTATATAT
AGAACATCCTGGAGTCCACCATGAACGGACAGTTGGATCTAAGTGGGAAGCTAATCATCAAAGCTCAACT
TGGGGAGGATATTCGGCGAATTCCTATTCATAATGAAGATATTACTTATGATGAATTAGTGCTAATGATG
CAACGAGTTTTCAGAGGAAAACTTCTGAGTAATGATGAAGTAACAATAAAGTATAAAGATGAAGATGGAG

ATCTTATAACAATTTTTGATAGTTCTGACCTTTCCTTTGCAATTCAGTGCAGTAGGATACTGAAACTGAC
ATTATTTGTTAATGGCCAGCCAAGACCCCTTGAATCAAGTCAGGTGAAATATCTCCGTCGAGAACTGATA
GAACTTCGAAATAAAGTGAATCGTTTATTGGATAGCTTGGAACCACCTGGAGAACCAGGACCTTCCACCA
ATATTCCTGAAAATGATACTGTGGATGGTAGGGAAGAAAAGTCTGCTTCTGATTCTTCTGGAAAACAGTC
TACTCAGGTTATGGCAGCAAGTATGTCTGCTTTTGATCCTTTAAAAAACCAAGATGAAATCAATAAAAAT
GTTATGTCAGCGTTTGGCTTAACAGATGATCAGGTTTCAGGGCCACCCAGTGCTCCTGCAGAAGATCGTT
CAGGAACACCCGACAGCATTGCTTCCTCCTCCTCAGCAGCTCACCCACCAGGCGTTCAGCCACAGCAGCC
ACCATATACAGGAGCTCAGACTCAAGCAGGTCAGATTGAAGGTCAGATGTACCAACAGTACCAGCAACAG
GCCGGCTATGGTGCACAGCAGCCGCAGGCTCCACCTCAGCAGCCTCAACAGTATGGTATTCAGTATTCAG
CAAGCTATAGTCAGCAGACTGGACCTCAACAACCTCAGCAGTTCCAGGGATATGGCCAGCAACCAACTTC
CCAGGCACCAGCTCCTGCCTTTTCTGGTCAGCCTCAACAACTGCCTGCTCAGCCGCCACAGCAGTACCAG
GCGAGCAATTATCCTGCACAAACTTACACTGCCCAAACTTCTCAGCCTACTAATTATACTGTGGCTCCTG
CCTCTCAACCTGGAATGGCTCCAAGCCAACCTGGGGCCTATCAACCAAGACCAGGTTTTACTTCACTTCC
TGGAAGTACCATGACCCCTCCTCCAAGTGGGCCTAATCCTTATGCGCGTAA**C**CGTCCTCCCTTTGGTCAG
GGCTATACCCAACCTGGACCTGGTTATCGATAAGGAGGCTCCTCTACACCAATTAATGTAGCTGCTAGCT
ATTGGCCTCCCAAAAGACTCCAGTACTATTTTAATTTGTATTGAAGAAGTTCAGAAATTTAAAAGCAGAG
CATTTTTTATGATATCATTGTTGGTGTTAATTGAAAGTATAATTTGCTGGAACACAAAGACCAAAATGAA
AGTTTTTTCCTCCCTGCTTAAAAATGTAGCAGCTTCTTAGTTACTTTGGAACACTACTCTTACATGTATA
AAGTGATTGACTTGACTTTCTAGCTTCCCTTGTCCGGAGGATATTAAAATGCTAGGGTGAGGTTTAGCCA
TCTTACTTGGCTTTTTACTATTAA**CATGATGTACTAAAGTAGAGC**CCTTTGAGAATACAAGATATTATGT
ATAAAATGTAACACTGATGATAGGTTAATAAAGATGATTGAATCCAAAAAAAAAAAAAAAAAAAAAA

55) Entrez Gene ID 10397 = NDRG1, N-myc downstream regulated 1 = D87953

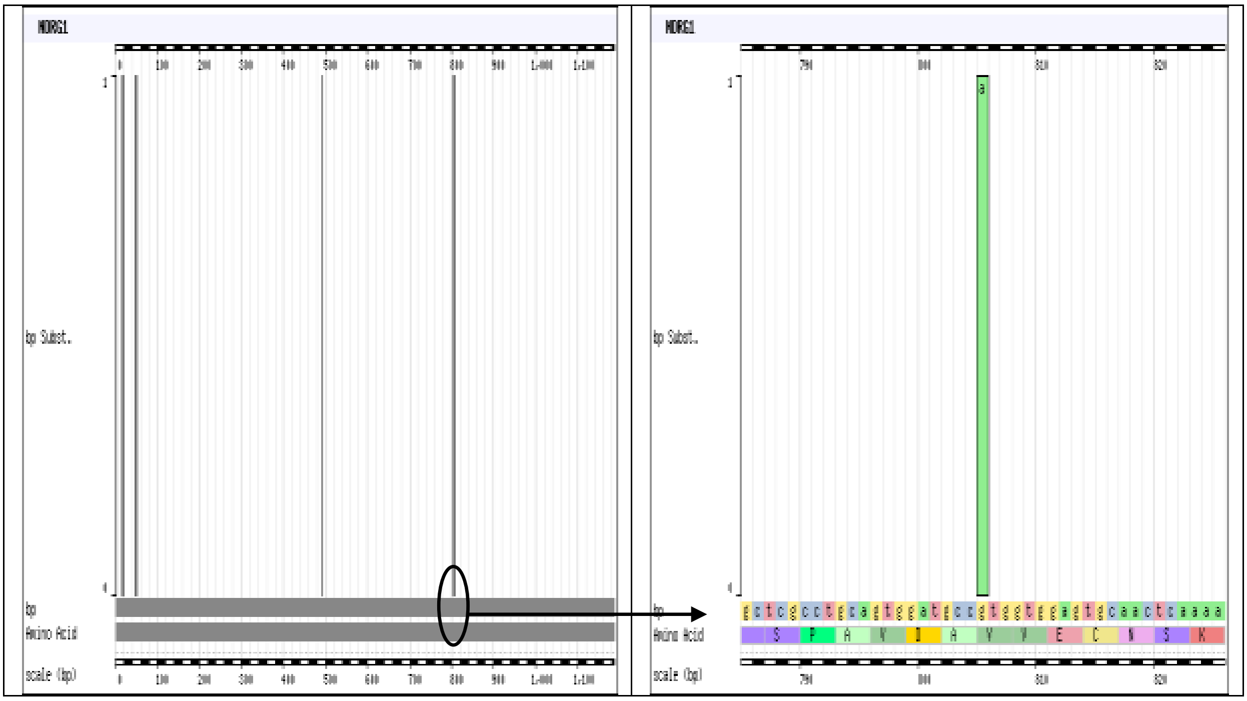

>gi|1596166|dbj|D87953.1| Homo sapiens mRNA for RTP, complete cds
CCCAGCTGGTGCTGAAGCTCGTCAGTTCACCATCCGCCCTCGGCTTCCGCGGGGCGCTGGGCCGCCAGCC
TCGGCACCGTCCTTTCCTTTCTCCCTCGCGTTAGGCAGGTGACAGCAGGGACATGTCTCGGGAGATGCAG
GATGTAGACCTCGCTGAGGTGAAGCCTTTGGTGGAGAAAGGGGAGACCATCACCGGCCTCCTGCAAGAGT
TTGATGTCCAGGAGCAGGACATCGAGACTTTACATGGCTCTGTTCACGTCACGCTGTGTGGGACTCCCAA
GGGAAACCGGCCTGTCATCCTCACCTACCATGACATCGGCATGAACCACAAAACCTGCTACAACCCCCTC
TTCAACTACGAGGACATGCAGGAGATCACCCAGCACTTTGCCGTCTGCCACGTGGACGCCCCTGGCCAGC
AGGACGGCGCAGCCTCCTTCCCCGCAGGGTACATGTACCCCTCCATGGATCAGCTGGCTGAAATGCTTCC
TGGAGTCCTTCAACAGTTTGGGCTGAAAAGCATTATTGGCATGGGAACAGGAGCAGGCGCCTACATCCTA
ACTCGATTTGCTCTAAACAACCCTGAGATGGTGGAGGGCCTTGTCCTTATCAACGTGAACCCTTGTGCGG

AAGGCTGGATGGACTGGGCCGCCTCCAAGATCTCAGGATGGACCCAAGCTCTGCCGGACATGGTGGTGTC
CCACCTTTTTGGGAAGGAAGAAATGCAGAGTAACGTGGAAGTGGTCCACACCTACCGCCAGCACATTGTG
AATGACATGAACCCCGGCAACCTGCACCTGTTCATCAATGCCTACAACAGCCGGCGCGACCTGGAGATTG
AGCGACCAATGCCGGGAACCCACACAGTCACCCTGCAGTGCCCTGCTCTGTTGGTGGTTGGGGACAGCTC
GCCTGCAGTGGATGCC**G**TGGTGGAGTGCAACTCAAAATTGGACCCAACAAAGACCACTCTCCTCAAGATG
GCGGACTGTGGCGGCCTCCCGCAGATCTCCCAGCCGGCCAAGCTCGCTGAGGCCTTCAAGTACTTCGTGC
AGGGCATGGGATACATGCCCTCGGCTAGCATGACCCGCCTGATGCGGTCCCGCACAGCCTCTGGTTCCAG
CGTCACTTCTCTGGATGGCACCCGCAGCCGCTCCCACACCAGCGAGGGCACCCGAAGCCGCTCCCACACC
AGCGAGGGCACCCGCAGCCGCTCGCACACCAGCGAGGGGGCCCACCTGGACATCACCCCCAACTCGGGTG
CTGCTGGGAACAGCGCCGGGCCCAAGTCCATGGAGGTCTCCTGCTAGGCGGCCTGCCCAGCTGCCGCCCC
CGGACTCTGATCTCTGTAGTGGCCCCCTCCTCCCCGGCCCCTTTTCGCCCCCTGCCTGCCATACTGCGCC
TAACTCGGTATTAATCCAAAGCTTATTTTGTAAGAGTGAGCTCTGGTGGAGACAAATGAGGTCTATTACG
TGGGTGCCCTCTCCAAAGGCGGGGTGGCGGTGGACCAAAGGAAGGAAGCAAGCATCTCCGCATCGCATCC
TCTTCCATTAACCAGTGGCCGGTTGCCACTCTCCTCCCCTCCCTCAGAGACACCAAACTGCCAAAAACAA
GACGCGTAGCAGCACACACTTCACAAAGCCAAGCCTAGGCCGCCCTGAGCATCCTGGTTCAAACGGGTGC
CTGGTCAGAAGGCCAGCCGCCCACTTCCCGTTTCCTCTTTAACTGAGGAGAAGCTGATCCAGTTTCCGGA
AACAAAATCCTTTTCTCATTTGGGGAGGGGGGTAATAGTGACATGCAGGCACCTCTTTTAAACAGGCAAA
ACAGGAAGGGGGAAAAGGTGGGATTCATGTCGAGGCTAGAGGCATTTGGAACAACAAATCTACGTAGTTA
ACTTGAAGAAACCGATTTTTAAAGTTGGTGCATCTAGAAAGCTTTGAATGCAGAAGCAAACAAGCTTGAT
TTTTCTAGCATCCTCTTAATGTGCAGCAAAAGCAGGCAACAAAATCTCCTGGCTTTACAGACAAAAATAT
TTCAGCAAACGTTGGGCATCATGGTTTTTGAAGGCTTTAGTTCTGCTTTCTGCCTCTCCTCCACAGCCCC
AACCTCCCACCCCTGATACATGAGCCAGTGATTATTCTTGTTCAGGGAGAAGATCATTTAGATTTGTTTT
GCATTCCTTAGAATGGAGGGCAACATTCCACAGCTGCCCTGGCTGTGATGAGTGTCCTTGCAGGGGCCGG
AGTAGGAGCACTGGGGTGGGGGCGGAATTGGGGTTACTCGATGTAAGGGATTCCTTGTTGTTGTGTTGAG
ATCCAGTGCAGTTGTGATTTCTGTGGATCCCAGCTTGGTTCCAGGAATTTTGTGTGATTGGCTTAAATCC
AGTTTTCAATCTTCGACAGCTGGGCTGGAACGTGAACTCAGTAGCTGAACCTGTCTGACCCGGTCACGTT
CTTGGATCCTCAGAACTCTTTGCTCTTGTCGGGGTGGGGGTGGGAACTCACGTGGGGAGCGGTGGCTGAG
AAAATGTAAGGATTCTGGAATACATATTC**CATGGGACTTTCCTTCCCTCT**CCTGCTTCCTCTTTTCCTGC
TCCCTAACCTTTCGCCGAATGGGGCAGCACCACTGACGTTTCTGGGCGGCCAGTGCGGCTGCCAGGTTCC
TGTACTACTGCCTTGTACTTTTCATTTTGGCTCACCGTGGATTTTCTCATAGGAAGTTTGGTCAGAGTGA
ATTGAATATTGTAAGTCAGCCACTGGGACCCGAGGATTTCTGGGACCCCGCAGTTGGGAGGAGGAAGTAG
TCCAGCCTTCCAGGTGGCGTGAGAGGCAATGACTCGTTACCTGCCGCCCATCACCTTGGAGGCCTTCCCT
GGCCTTGAGTAGAAAAGTCGGGGATCGGGGCAAGAGAGGCTGAGTACGGATGGGAAACTATTGTGCACAA
GTCTTTCCAGAGGAGTTTCTTAATGAGATATTTGTATTTATTTCCAGACCAATAAATTTGTAACTTTGCA
GCGGAAAAAAAAAAAAAAAAAAAAAAAAAAAAAAAAAAAAAAAAAA

56) Entrez Gene ID 10892 = MALT1, mucosa associated lymphoid tissue lymphoma translocation gene 1 = AL137399

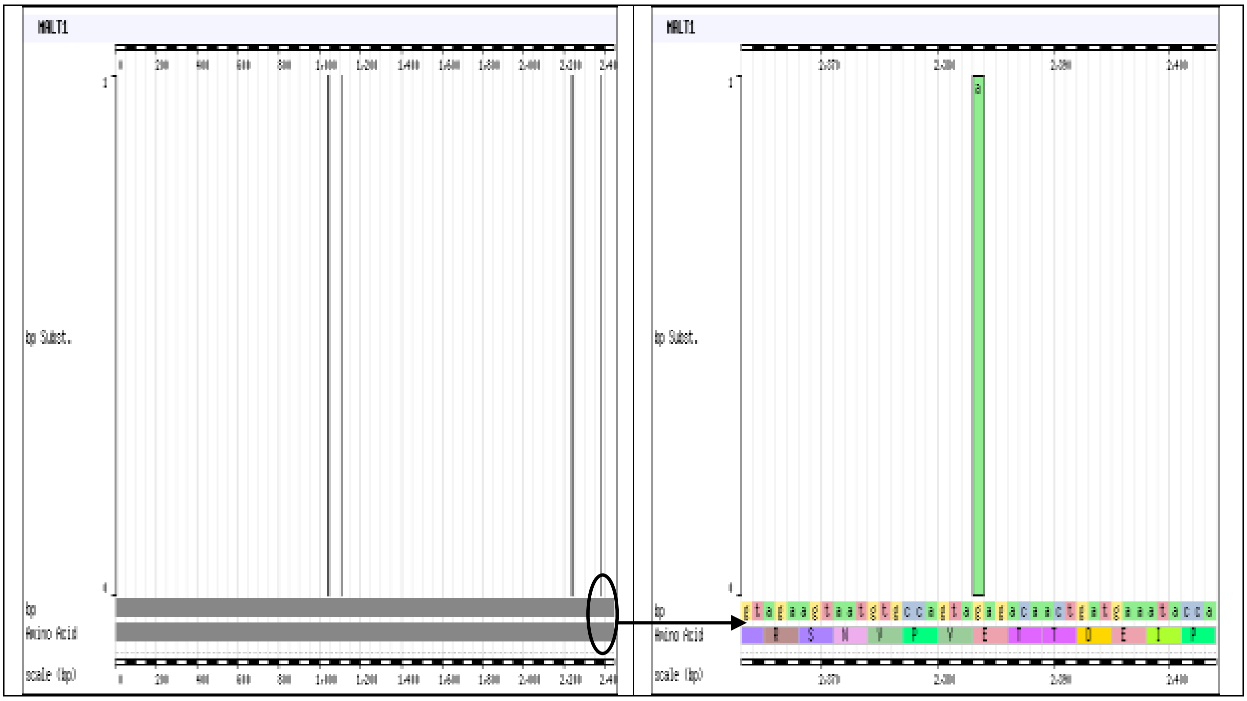

>gi|6807945|emb|AL137399.1| Homo sapiens mRNA; cDNA DKFZp434L132 (from clone DKFZp434L132)
TGTTCAGATTCAATTAGGATTTGCAGCTGAGTTTTCCAATGTCATGATCATCTATACAAGTATAGTTTAC
AAACCACCGGAGATAATAATGTGTGATGCCTACGTTACTGATTTTCCACTTGATCTAGATATTGATCCAA
AAGATGCAAATAAAGGCACACCTGAAGAAACTGGCAGCTACTTGGTATCAAAGGATCTTCCCAAGCATTG
CCTCTATACCAGACTCAGTTCACTGCAAAAATTAAAGGAACATCTAGTCTTCACAGTATGTTTATCATAT
CAGTACTCAGGATTGGAAGATACTGTAGAGGACAAGCAGGAAGTGAATGTTGGGAAACCTCTCATTGCTA
AATTAGACATGCATCGAGGTTTGGGAAGGAAGACTTGCTTTCAAACTTGTCTTATGTCTAATGGTCCTTA
CCAGAGTTCTGCAGCCACCTCAGGAGGAGCAGGGCATTATCACTCATTGCAAGACCCATTCCATGGTGTT
TACCATTCACATCCTGGTAATCCAAGTAATGTTACACCAGCAGATAGCTGTCATTGCAGCCGGACTCCAG
ATGCATTTATTTCAAGTTTCGCTCACCATGCTTCATGTCATTTTAGTAGAAGTAATGTGCCAGTAGAGAC
AACTGATGAAATACCATTTAGTTTCTCTGACAGGCTCAGAATTTCTGAAAAATGACCTCCTTGTTTTTGA
AAGTTAGCATAATTTTAGATGCCTGTGAAATAGTACTGCACTTACATAAAGTGAGACATTGTGAAAAGGC
AAATTTGTATATGTAGAGAAAGAATAGTAGTAACTGTTTCATAGCAAACTTCAGGACTTTGAGATGTTGA
AATTACATTATTTAATTACAGACTTCCTCTTTCTAAGATTTTGTGAATTGGTTGAATAGTTCTATACAAA
TGAAGTATGGAGGTGTGTATGTTTATATGTATATAACAAAATATTTTCATTGTGACCACTCTGAAGTAAG
AGCAATGGGAATGGCATTATTGTAGAATAAGTCATTGTATTTTTAACACCAGAAAGAACCTTGCCGATCA
CCAGGCATAACCTAATTTTATCCATGGAAGAAACACAGAAAGGCATCTAAGTTAGAGCTGGCACCAGAAC
TGAGACCTCCAGAAATCTATTCCAGTATTTTTTCCACTACACAACTGCCTTCCTGACAGGTTCTGAGATA
AGTGTTATGTTTGTAGATAGAGTGAAATATATTTATATATATATAAATATATACAGATACATATCTGTGT
ATTATCTCAAGGAATGTACAAACTTTAGTTTTTGATTATAAGGACTTCACTGCAAGTTTTAGTTAAGAGG
TTTGTATATAAATCTGTTATAGAACAGGCTGAAATTTCTTGTTCATAAGATTATGAAACCACATGAGAAG
TGATAAAATGTTTGTTAAAGCTAGATAGAGGTTAAGAATCAAGATATAATGGATAATTTTCATAGCTGCC
TATCAGAATTTCCCAAATATTTAGCATCTTCCTTGATAATATGTATTTTCTTCTTGAATTTCACTGGCCT
AATGAGATAATACTCTTATCTTTGGCTCTACCTAAAAGTTGGTTAAAAATGCAATTGGCATTAACAAGGA
AAAATACTGAATTAGTAATTTTAAAAGTCTCACAAAGAAAATCCCAGGCCTAGATGGCTGCATTGTTGAA
TTCTGCCAAACATTAAAATTAGCACTAATTTTTTGCACACTGTTTCTAAAAGTAGGAGAGGAAAGAACAC
TTCCCAACTTACTCTAGGTCAGTATTACCCTGATACTAGACTAGACATCACAAGAAAACTATAAGCCAAT
ATTCCTTATTAATACAAACACAAAAATCATTAACAAAAATATTAGCAAACTGAATCCAGCAACCTATAAA
AAGGATTCTATATCATGACCAAGTGGAATTTATCCCAGGAATTCAAGGTTGGTTCAACATCTAAAAATCA
AATAAGCTAATATACAGTCAGTTCTCATTATTCACAGTAATTATGTTCTACAGAATATTCTCCCATAAAC
ACTGAATTAAATATGGAACAACTGCTTTTAGGAGAAAGTGTATTTGTGTATATGTGTGTATACATATGTT
TATCTCACACACATTATGAGCTTGAATTCTTAATTCATCCTAGCAAATTCTACCTTATTTTACAGAAGTG
AAAGTGAGGTATCAGAAGTGTTAAGTGACATGCCTGAGGCACCCCCCTAACAGGTGTCAGAGCTGAAATT

CAAACCCCATCCAGCTGGCCCCGGAGCCAGAGCTTCTTGTACTACACAGAATTGCCCCTGCCATTTCCAC
CCTCCAGTCATTTCTCTATGAGACTGAAGCAGGAAGGCAGAGCATCATCTTGTTCAGCCTCAGCTGGGAA
CATGTGTACTGGGTGACTCAAATTTTTCACCCATTTACACATATCCACAAATGACTGCAAAAGTGCCACG
GATATCAATTTGAGGGTTATAAATTTTAGCAAGTTGGTAAATTCACAAATACATAACCTTGAATAATGAG
GATCAACTGTACCATATTTAATAAAGCACAAAACCCACACAGATTGTCTTATTACAGCATTTGATAAAAT
CCAAAACTCTTTCATAAAAACACTCAACAAACTTAGGAATAAAAGGAATCTTCCTAGATATGATAAATAT
AACATCTATGAAAAGCCCACACCTAACATTATACTT**CATGGTGATAGACTGAAGGCT**GAATGTTTTCCCC
TTAAGATTGGGAAGAAGGACAAGGATGTTCACTCGGCACTACTTCTATTCAGCATTGTACTTGAAGTTCT
AGCCACAGCAGTTAGGTTAGGAATTCAAGGTTTGTTCAACATCTAAAAATCAAATAAGCTAATAAAGAAA
AGAGGTTTATACTGCAAAAGAAGTGAAACTATATGTATTCACAGTTGATACATAGTTGTATATAGAAAAT
GCTAAAGAATCCATAAAAAGTAATAAATGAGTTCAACAGGTTAAAAAAAAAAAAAAAA

57) Entrez Gene ID 10962 = MLLT11, myeloid/lymphoid or mixed-lineage leukemia (trithorax homolog, Drosophila); translocated to, 11 = BC006471

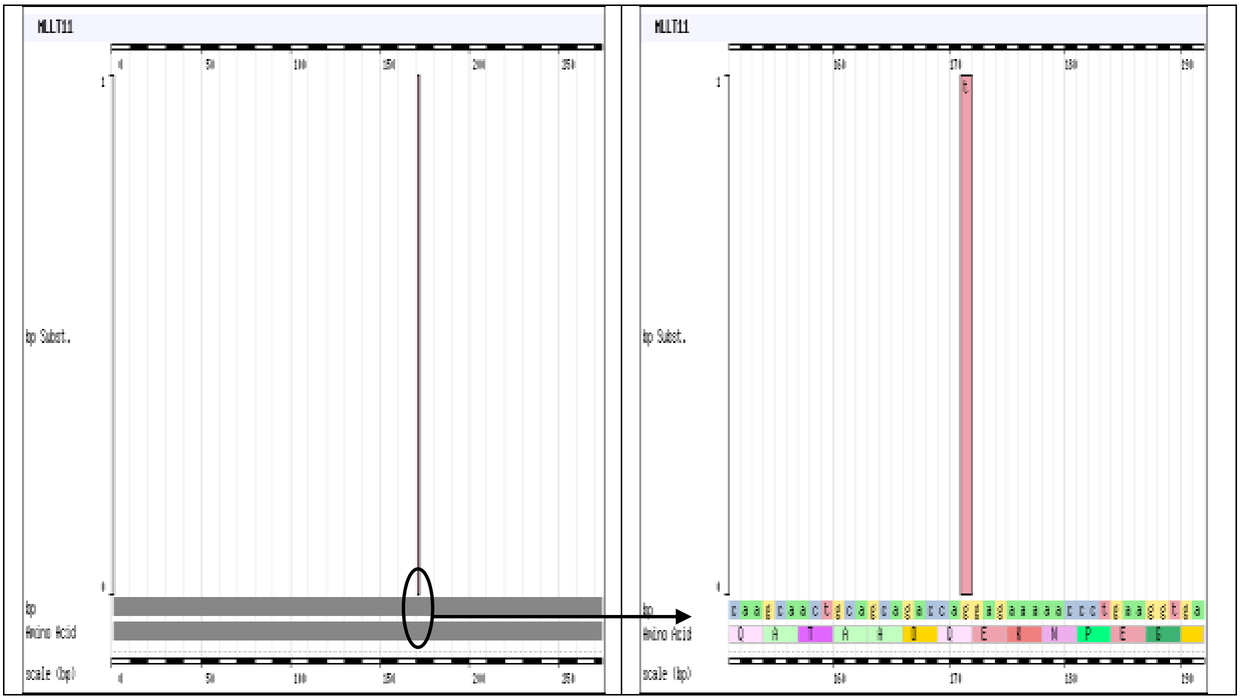

>gi|34782804|gb|BC006471.2| Homo sapiens myeloid/lymphoid or mixed-lineage leukemia (trithorax homolog, Drosophila); translocated to, 11, mRNA (cDNA clone MGC:4013 IMAGE:2823316), complete cds
GGAAGCTATGAGGGACCCTGTGAGTAGCCAGTACAGTTCCTTTCTTTTCTGGAGGATGCCCATCCCAGAA
CTGGATCTGTCGGAGCTGGAAGGCCTGGGTCTGTCAGATACAGCCACCTACAAGGTCAAAGACAGCAGCG
TTGGCAAAATGATCGGGCAAGCAACTGCAGCAGACCA**G**GAGAAAAACCCTGAAGGTGATGGCCTCCTTGA
GTACAGCACCTTCAACTTCTGGAGAGCTCCCATTGCCAGCATCCACTCCTTCGAACTGGACTTGCTCTAA
GGCCAAGACTTCTCTCTCCCATCACCTTGCCCTCATTGTCTTCCCTCTCAAGCCCCTTCCTTTCCACTCC
TTTCCCATTTTAATCTTGTTCTCTCCCTACTGTGTTGGTGGTGCTGATGAATCTGCCAGAGTTGAGTTCT
ATGTATTTATTTATCTATCTGTCTACTCCATTTCTCTCAAAAGCCCTCAAGTCACAAAGTAAATGGTTCA
AGCAATGGAGTACTGGGTCACAGGGATTCCTCCTTTCCCCCCCAAATATTAACTCCAGAAACTAGGCCTG
ACTGGGGACACCTGAGAGTAGTATAGTAGTGCAAAATGGAAGACTGATTTTTGACTCTATTATAATCAGC
TTCAGAGATTCCTTAAACCTTCCTAATTTCCTGCTCCAGGGCAGTAAACACAAATATTTCTTCAAGGGGT
GATGAAAACCTCGGAAGTTTTAATTTGAGGTTATCTGCTACGAAACAGTATTTCTAAAAGGCTAAAGTGA
TAAGTCTCTTGCTTTTTTTTGATCCTGCTCTTATATTCTTTTTTTTCCTCAGAGAAATCAGGAGGGTAGT
TAGAGGTATAAAACAGGAGGAAATATTATGGAAAATGAAAATAGGGAAAATAATTGAATCATTTTAGAAG
TAGCTAATTTCTTTTCTCAAAAGAGTGTCCCTTCTTCACACCTACTCACTTTACAACTTTGCTCCTAACT
GTGGGTTGAAAACTCTAGCTAAAGAAAGTTATCAAATCTTAA**CATGCATTCCTACTATTATGA**TAGTTTT

TAAGGTTTCAATTCAATCTTCTGAACGGCATAAGTCCTATTTTAGCCTTACCTCCTGCATTTGCAATACG
TAATACTGATCAGTGGGCACAGTTCTTCAGCTACATTGAGACCCTGAAATGAACAATTATATTCTGACTC
GACATCTTGTCCCCAATCCTTCCAAAAATATTGATGGTGATTTGTGCTACCATTTACTCGTTTATTTAAT
AAAGACATTCAATCCCAGGAAAAAAAAAAAAAAAAAAAAAAA

58) Entrez Gene ID 10978 = CLP1, cleavage and polyadenylation factor I subunit, homolog (S. cerevisiae) = BC000446

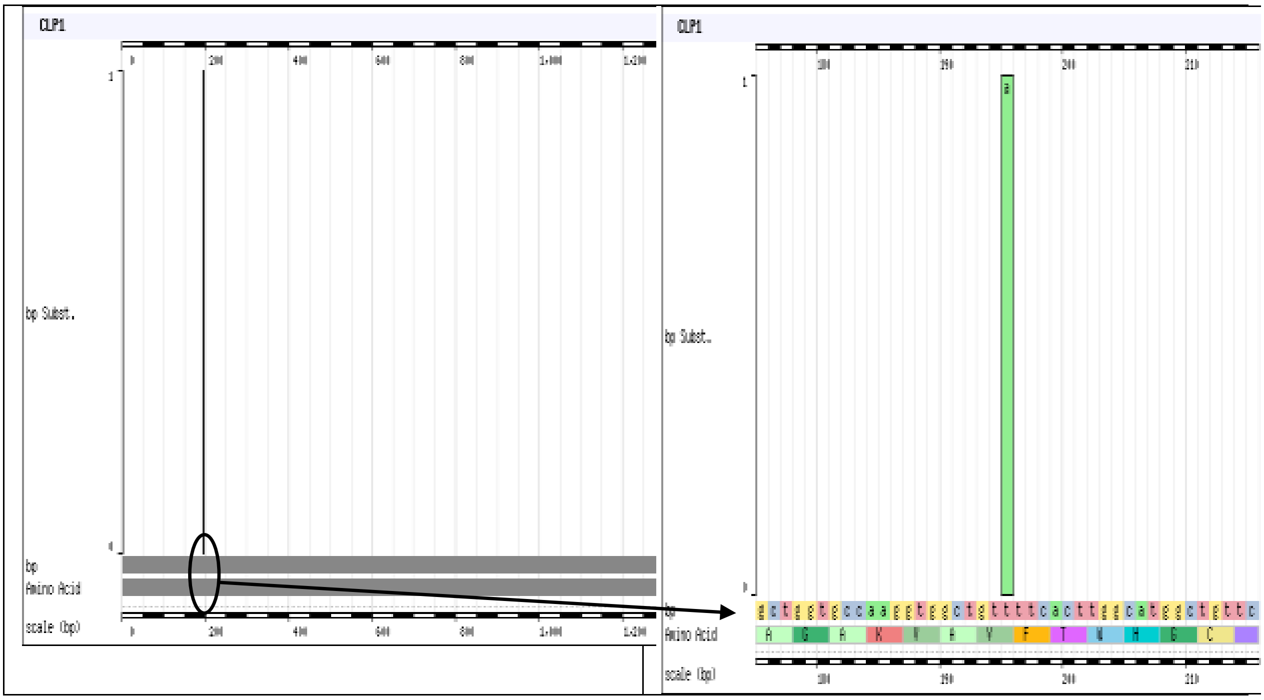

>gi|12653352|gb|BC000446.1| Homo sapiens CLP1, cleavage and polyadenylation factor I subunit, homolog (S. cerevisiae), mRNA (cDNA clone MGC:8442 IMAGE:2821321), complete cds
GGGCACGAGGCCGGCGTGGGTCCGGGCAAGAACCGCTTGTAGTTTGGTTTAAATTCTGCACGGGAGGACC
TTCTGAGTTTACCTGTTGGGCTCCTGGCTGCGCAGGCACAGCAGCTACACAGAAGAGATGGGAGAAGAGG
CTAATGATGACAAGAAGCCAACCACTAAATTTGAACTAGAGCGAGAAACAGAACTTCGCTTTGAGGTGGA
GGCATCTCAGTCAGTTCAGTTGGAGTTGTTGACTGGCATGGCAGAGATCTTTGGCACAGAGCTGACCCGA
AACAAGAAATTCACCTTTGATGCTGGTGCCAAGGTGGCTGT**T**TTCACTTGGCATGGCTGTTCTGTGCAAC
TGAGCGGCCGCACTGAGGTGGCTTATGTCTCCAAGGACACTCCTATGTTGCTTTACCTCAACACTCACAC
AGCCTTGGAACAGATGCGGAGGCAAGCGGAAAAGGAAGAAGAGCGAGGTCCCCGAGTGATGGTAGTGGGC
CCCACTGATGTGGGCAAGTCTACAGTGTGTCGCCTTCTGCTCAACTACGCAGTGCGTTTGGGCCGCCGTC
CCACTTATGTGGAGCTGGATGTGGGCCAGGGTTCTGTGTCCATCCCTGGTACCATGGGGGCCCTCTACAT
CGAGCGGCCTGCAGATGTCGAAGAGGGTTTCTCTATCCAGGCCCCTCTGGTGTATCATTTTGGTTCCACC
ACTCCTGGCACTAACATCAAGCTTTATAATAAGATTACATCTCGTTTAGCAGATGTGTTCAACCAAAGGT
GTGAGGTGAACCGAAGGGCATCTGTGAGTGGCTGTGTCATTAACACCTGTGGCTGGGTCAAGGGCTCTGG
TTACCAGGCTCTGGTGCATGCAGCCTCAGCTTTTGAGGTGGATGTCGTTGTTGTTCTGGATCAAGAACGA
CTGTACAATGAACTGAAACGGGACCTCCCCCACTTTGTACGCACTGTGCTGCTCCCTAAATCTGGGGGTG
TGGTGGAGCGCTCCAAGGACTTCCGGCGGGAATGTAGGGATGAGCGTATCCGTGAGTATTTTTATGGATT
CCGAGGCTGTTTCTATCCCCATGCCTTCAATGTCAAATTTTCAGATGTGAAAATCTACAAAGTTGGGGCA
CCCACCATCCCAGACTCCTGTTTACCTTTGGGCATGTCTCAAGAGGATAATCAGCTCAAGCTAGTACCTG
TCACTCCTGGGCGAGATATGGTGCACCACCTACTGAGTGTTAGCACTGCCGAGGGTACAGAGGAGAACCT
GTCCGAGACAAGTGTAGCTGGCTTCATTGTGGTGACCAGTGTGGACCTGGAGCATCAGGTGTTTACTGTT
CTGTCTCCAGCCCCTCGCCCACTGCCTAAGAACTTCCTTCTCATCATGGATATCCGGTTCATGGATCTGA
AGTAGAGATCAGCAGGAAGCCTTGCTGCCTGGGACATAGAGATCATCTGGCCACCCCTAGAGGCAGATGG
GCTGAGATAAAAGACTGTTGGGGCCACCTGACCAGTAAACTGTGGACTAGTAGAAAGTTCATATTCTACC

TCTAAAAACAGGTAGTGGTAACCTGACTCTTCTAATCTTGAACCAAAAGGAAAAC**CATGAGACTGTAATT**
GGTTTCTTAGACCACCTAAGATGCCACTTTGAATTCTCTAAGACCCTGGAGAATTGCATTTCTTTCACTG
TGCTACTATGTGGTTTTTAAAAAATCAATGCTTTATATTCCATATGTGGTTCTTACCCATTTATCTAGGA
TGAAAGTGTGAATTAGAGGGACTCCTTCCAATAAAGTTCAAACTTAAAAAAAATCATTTTAATAAATATT
TTTGCCATATCATAAAAAAAAAAAAAAAAAAA

59) Entrez Gene ID 23365 = ARHGEF12, Rho guanine nucleotide exchange factor (GEF) 12 = BC031784

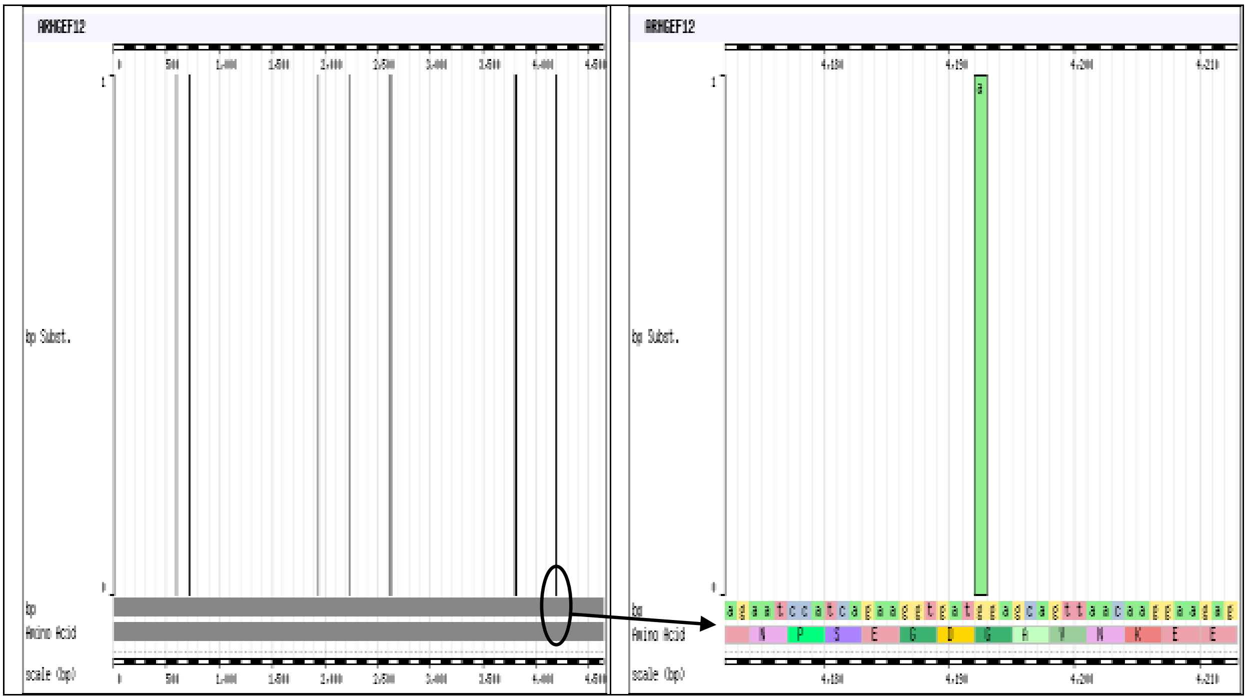

>gi|22749563|gb|BC031784.1| Homo sapiens Rho guanine nucleotide exchange factor (GEF) 12, mRNA (cDNA clone IMAGE:4538083), with apparent retained intron
GTTCAATTTCTCCTTGGAACTCTAACAGGACTAACTGGAAAAATACTTTGACCATAGCTCTAGTACTTCC
AAATAAATTCATATCTAGATTTTCAAACAAAGCCCCAAGATGAAGTCTAATTCTGAAAATATCTCACAAT
TTTTGAATGTTCTTTTTATCTACCGACCTAACTTCTCTTTTCCCTCCCAACTTGGCCTCCCACACGTTCC
ATTTCATTTTGGCCATTATAGTCACATCCGTTTGACTTTGGTTGTGACATCTTCCTTTCAGGATGATGGA
ATTAGATGCAGTCTGTCTTTGAGTGAGACTGGTCACCATCAGAGGAGTTTTTTCTAAGTGACGTGACTAG
AAGTTGAAATCTACTTCCCTGCTAAAGGGCACAGGGGTGGTGTACAAAGAAAGCTACCTTCCCAGAGCAA
GCAGGCTGCATTTTAGCTATGGAAGTGACCTGCTGTGACACTGTGCTCTCTTCTGTGGGCTTAATGGTTC
CTTTGCTATGAAGTGGCAAATTACATGTAGAGTGTCTCCTTCCTTTTCAGAGAACAGTTAATCAAGGCAA
ATCAGCAAGCCCCCAAAGTGCTGTAATTTAACATCATGATTACCACCTTCGAAGCTATATATTTTGCATA
CTTTAAAATCACCTAACTTGGACTGCTTGAATTACATTGGCTTTTAGAACCGAAATTGTAACTATGTATT
GTATTTCATGGGAGGTATATTTTATGAGCTTTTTGGCTTTCTTTTTTCCCACAGCAACTGCCAATGAAGG
ATGAATCCCCTTTTTAAAAAGTTGTTGTTGTTGTTGTTTATTTGATTTTGAGTTAGGAGGGATAATAGAG
AAGTCCATTTAAAAATTATTTTTAGAAGCTAAAGAAAGTAATTATGCTTCCTGTGAATTGTCTTTTACTG
GCATCTTTGTTTTCCTCTTTGATGTTAGTAAATTTGGTGTAATACGTGGGGCTTCCATATTTCAAAGTGG
AAGCTTTCTTCTCTGAAGTCGATATATGGTTTTGAATTACTAGAGCTTTGGTCAGTATTTCCTTCCCTAT
ATGTCACAGAGGGCACCACTGAGAACTGCGTGCATAGGACCTCAAAATACAAAATTAGCAGGGCCTCACA
GTCAGCTTCCTCATGGCTAGTTTTTCCCCCTTATATTACAATTTTTGTTTTATAAGTCATTTTTTTCCTG
ATATTTCCACCACTTTTCAGAGTCATCTACAAAATTTTTCTTTCCTCAAGAAAAGAGTTCCTTTTGCCTT
ATTCCTTATGCCTTCCCCACTGGTATTGAGGGTTTTGATAATAAATTGGTAGGAAAAAAAAGTACCTCCT
AGAAGGAAGCCTTCCCCACCATTTCCAGGTGCCAACTGCTAAGCAGATATATTCCAAAAATGGTAACTGT
CATGTGCACACTGTTGGTTATTTTTAATAAGCCTCTTCCTACTAGAACATTTTATTTTCCTTGTTCACCA
TACAATCATGTACTCTTTAACAGAAATTGCTTTTAAAAAATATCTGGAACTATCTTTAAAAAAACTTTAT
TAATAAT**CATGTATTTTTACTGATCACA**TTTTGAAATGCCTAAAAGACTTTATTGTTCTAATTATCCAGA
TGTACCTTTGTAAAATAGCTCTTTTATGAATTAGCTGATAAGGCTGTATGTTTCTGGAACAAAATATTGG

TCATCTAAAAACTTTCTGTTTTCTGGGGTCTGGGAAAATAGAAAATAAGATTTCAAATATTAAATAAGCT
TAAAAAAAAAAAAAAAAAAAAAAAAAAAAAAAAAAAAAAAA

60) Entrez Gene ID 23512 = SUZ12, suppressor of zeste 12 homolog (Drosophila) = BC018583

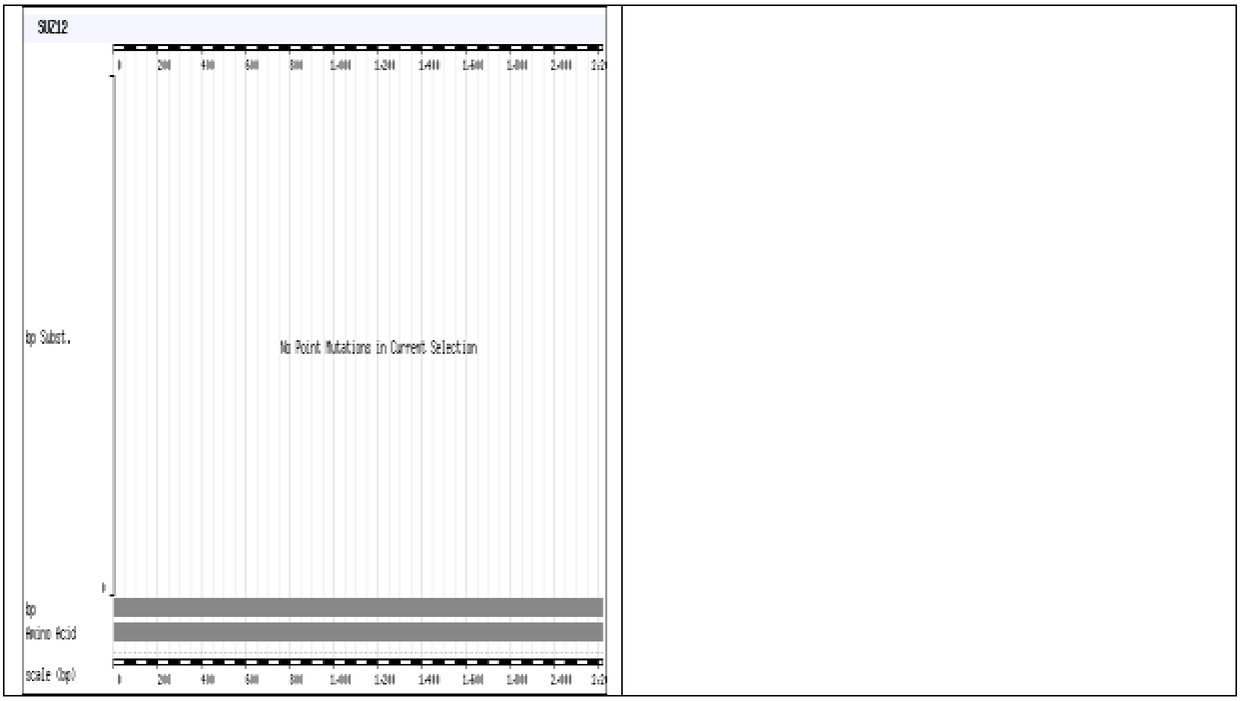

61) Entrez Gene ID 26039 = SS18L1, synovial sarcoma translocation gene on chromosome 18-like 1 = BC034494

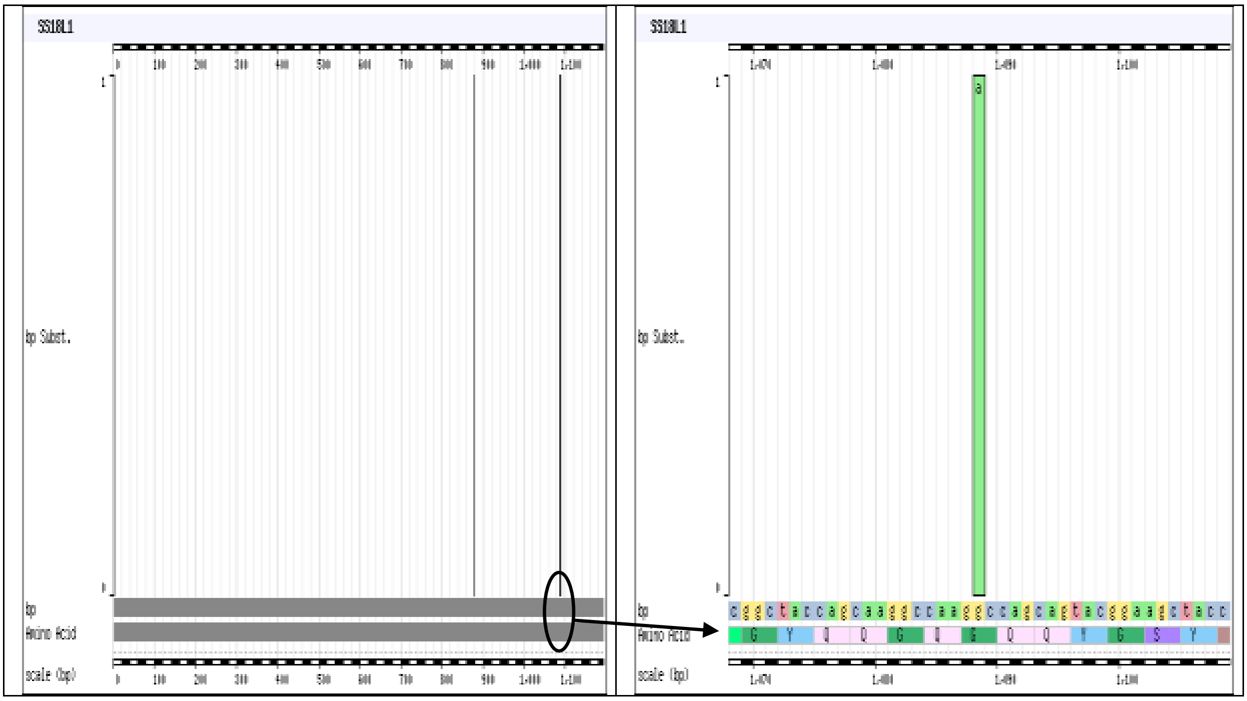

>gi|34192441|gb|BC034494.2| Homo sapiens synovial sarcoma translocation gene on chromosome 18-like 1, mRNA (cDNA clone MGC:26711 IMAGE:4823298), complete cds

AGGGCAGCCGGAGTATCCACCTCGATGACCACGGGCTGAGCCCCGCGCCGCCACCATGTCCGTGGCCTTC
GCGTCTGCCCGGCCAAGAGGCAAAGGGGAGGTTACGCAGCAAACCATCCAGAAGATGCTGGACGAGAACC
ACCACCTGATCCAGTGCATCCTGGAGTACCAGAGCAAGGGCAAGACGGCCGAGTGCACGCAGTACCAGCA
GATCCTGCACCGGAACCTGGTATACCTGGCCACGATCGCAGACTCCAACCAGAACATGCAGTCCCTGCTT
CCTGCCCCGCCCACGCAGAACATGAACCTGGGCCCTGGAGCCCTGACTCAGAGCGGCTCCAGCCAGGGCC
TGCACTCTCAGGGCAGCCTGAGTGACGCCATCAGCACGGGCCTGCCACCCTCCTCCCTCCTGCAGGGCCA
GATTGGCAACGGGCCGAGCCACGTGTCCATGCAGCAGACGGCGCCTAACACGCTGCCCACCACCTCCATG
AGCATCTCTGGGCCCGGCTACAGCCACGCGGGACCCGCCTCGCAGGGCGTCCCCATGCAGGGGCAAGGCA
CCATCGGCAACTACGTGTCTCGGACCAACATCAACATGCAGTCCAACCCAGTCTCCATGATACAGCAGCA
GGCGGCCACGTCGCACTACAGCTCGGCGCAGGGCGGCAGCCAGCACTACCAGGGCCAGTCGTCCATCGCC
ATGATGGGGCAGGGCAGCCAGGGGAGCAGCATGATGGGGCAGCGGCCCATGGCGCCCTACCGGCCCTCCC
AGCAAGGCTCTTCCCAGCAGTACCTGGGCCAGGAGGAGTACTATGGCGAGCAGTACAGCCACAGCCAGGG
CGCCGCGGAGCCCATGGGCCAGCAGTACTACCCCGACGGCCATGGCGATTACGCCTACCAGCAGTCATCC
TACACGGAGCAGAGCTACGACCGGTCCTTCGAGGAGTCCACGCAGCACTACTATGAGGGGGGAAACTCCC
AGTACAGCCAGCAGCAGGCCGGGTACCAGCAGGGTGCCGCGCAGCAGCAGACGTACTCCCAGCAGCAGTA
CCCCAGCCAGCAGAGCTACCCCGGGCAGCAGCAGGGCTACGGGTCTGCCCAGGGAGCCCCGTCACAGTAC
CCCGGCTACCAGCAAGGCCAAG**G**CCAGCAGTACGGAAGCTACCGAGCACCGCAGACAGCGCCGTCTGCCC
AGCAGCAGCGGCCCTACGGCTATGAACAGGGCCAGTATGGAAATTACCAGCAGTAAGGGACACACATTCT
GGCTGGAGCCCTTGTGGTAGCGTGTTCATCCAGGGGCCGGATGGGCTGGCGGCAGCTCTGGTGAATTGTG
ACATGTTGGTTACCTGTTCGCCCAGTGCCACGTCTGCATGTGAAGCGTGCTCATTTCATGCTGGGACCTA
CTTGCTCAGATCTCCAAGCAAGCATTTCTTTTCTTTTAGGGATGTCTGAAAGTCACATCCAGTTACATTA
CTGTGTTCTTTCTAATGAAAAGTAAAGGTTTTATATAGAGAAACTTGAGTAATTTTTACATTTCTAAGAC
ATTAAATCCCATTTAAATTCTGTGTGAACATTAAAGACAGCACACTTGCAAAAGTATGGTCAAAGGAAAA
AAATCCCACATTTCAATTAACAAGTAGCATGGACATTTGATCAACCTTTAGTTGGAATAATAATATTCAT
ATTTGCTATGAATCCTTTTAAAAAAATCTTTGGATAAATGCTGACAGATTTCCAAGAACTACCAAGAAAA
TACAAGAGATATCCAATGCTTGATATATGAGGCCTAGTAATAACGATATTTCTCTTTAATTGATGTTTTG
TTTTAAAAGTTAAAAGTAATTCTTGGCGTGGTGGTTCACGCCTGTAATCCCAGCACTTTGGGAGGCCGAA
GCGGGCGGATCACCTGAGGTCGGGAGTTCGAGACCAGCCTGACCAACATGGAGAAACCCCGTCCCTACTA
AAAATACAAAATTAGCCAGGTATGGTGGTGCATACCTGTAATCCCAGCTACTCGGGAACCTGAGGCAGGA
GAATGGCTTGAACCCAGGAGACAGAGGTTGTGGTGGGGCAAGATCGCACCATTGCACCCGAGCCTAGGCA
ACAAGAGTGAAATTCCGTCTCAAAAAAATAAATAAATAAATAAGTTAAAATTAATTCTTTATCCAGAGTC
GGGTGCTTTAGAATTTATAAGTCACTTATGTGTTTTGCTTGAATTAATTCTGACAGCCCCTATGAGGAAA
TCTGGAGGCAGGTAACAGTTCCCATTTTAGAGATGAAGAACTGAGGCACAGATTAAAGGACTTGCCTGTG
TTGAATACCAGTCCTGTTCTAGGACATTCTCCCCTCTCCTAGGAGACGGATGTCACGCACAAATGGGGAG
AGAAGTGTTTATTTTGTAGGCACTAAGGGTTTCTAAAACCCTTAACACTGGTAAGGGCTCAAAAATAAAC
GTATGTGTTCATATTCGATCACCGAAATGAGAGTTCTTAATTGCTAATTGACAAACGCGTTAGCAATTTC
AGTTAGGGAGTCATCTCCCTTGATTGTGTTCTTTTCCTGTCAATTTTCATAGACCTAATTTGCAAACTCA
ATCGGGGACTAAAATTTCCCACTGAAAATGTTAAACATTTTAGATAACTGTGAAGATAGTTTATTTTTAT
TCCTTGCCAATCTGGGAATATGCCTTTTTTGTGTGTTTGTGTGTTTTTTTAAGTGCTGTATTAATAATAC
TTTCTGAAAGAAAAGGACACTTACCCCAAAACTTCAATCTGAAATGTCTTACATTAAGAATATCTTGAAT
GTTGTGTATATATTTTAAAAAGCACTTTGCAAAATAGTTTGTACATTTATTTCCTAATTTATACATGATT
TTTGGTGTTAATATATTTAATGATTAATAACAGAATGTTTATTTAATGTGCTGTCCATTTTTATGTAATA
TTATGGGGAAAGTGATGCCAGCAGTTCCTTTTCATTATTCTATCTTCTGTCATATGAATGTTGAGCAAAA
CTTAGGCCAACATGAATTGTTTGTGAAGTGTGGTTGATGGTGCTTTGTTTTTTTCTGACTACTTCTATGG
AAGGCCAGTGAAGAAGCAAAGGAAGACATGAAAATTGACGCTCATTCTTCTTCCTATTGTTCCCTGACAT
CCAGCAAATTGTGAATTTGAAAAATGATGGCCAGTTTTCAGAAGTGCTGACAAATTCATATTGGTATGCA
AAAGCTCATCACCCATTAAGGTTTGTTGTTGAATCAACAGTACTCAGCATATTAAAACAGTACATCAGAA
CT**CATGCCAACAGTCTTTATGAT**GGGATTAAGGTGGACAAGATCTCCTAAGATCTGTGAATGGGATTAAG
GTGGACAAGATCTCCTAAGATCTGAAAAGAAACCTTAATACGCTCATATGGTTGGAGTGTTAAGTGAACC
TCTGATTTTGTCAGGGTTTTTCTACGTGTAGGCGTGAATAGGGGGCACCCCTTCAAAACTGTACAAAGAA
GACGACTGTTTTCCATTTCCATTTAAACATTTTTAGCCACTTCATTTCTATTTATTGAACAGGTCAAATT
TGTCTTGTTATTTGTGAGTACAGTACATTTAAAAAACATCCTTATCGGTTATTTTTTTTTCAGTCGGAGT
TTGACGTATAAATTGTTTATGCTTTTGGTGTAATCTCTTAATAAACTGGTTCTTCAAAAATCAAAAAAAA
AAAAAAAA

62) Entrez Gene ID 27086 = FOXP1, forkhead box P1 = AF250920

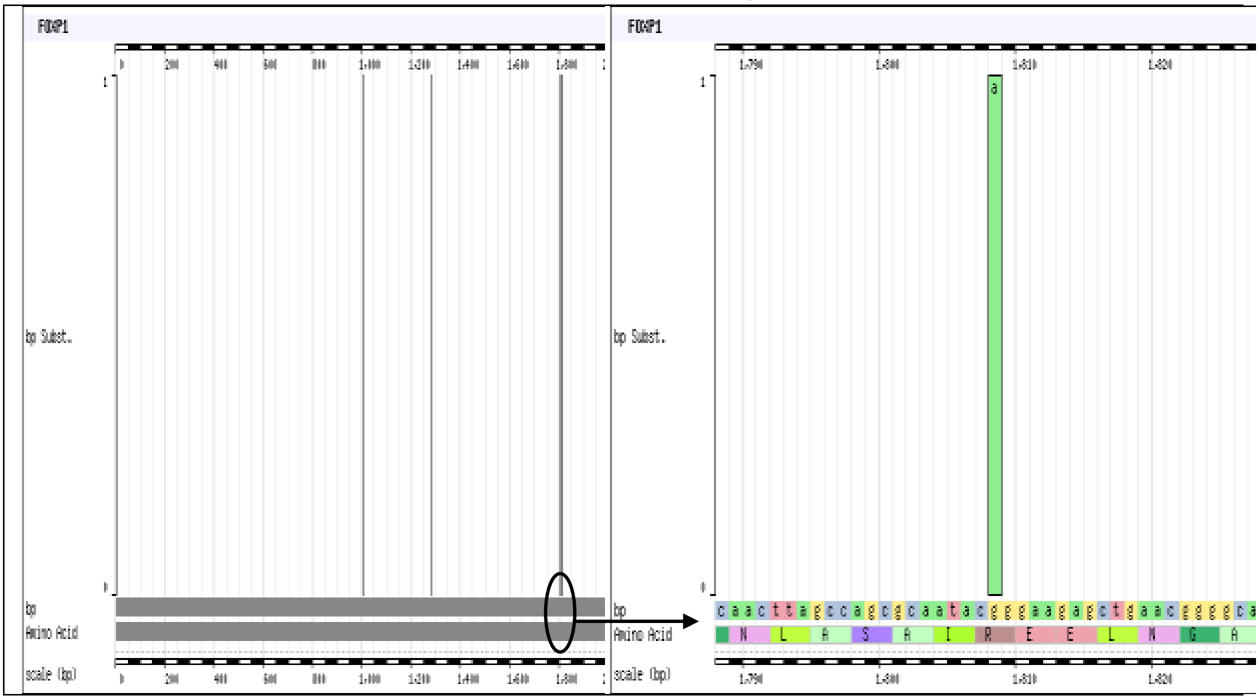

>gi|18032255|gb|AF250920.1| Homo sapiens 12CC4 mRNA, complete cds
ATCACAGCTTGGTGCTGGGGCTCAGCATGGCCAGGTGGCTAACCAGGGCTTGGTGTCAACTACATTTGGG
ATGATGACACTTGGAGGACACAGAGCCACTGTGCAGCAGGAGTCGCCACTGCTAACAGCCAGAGTGTCCC
AGAAGCTTTTGTTGACAATTCCAGTTTCCGAACAAAACATTTCGGCAATGGTGAGGGCTTCGATCCCTTC
TCTGATTTGCTGTCAGCCATGAACGGATGGATGTGATGCCTGCTAGCCAAAAGGCTTCCCTCTGTGTGTT
GCAGTCCTGTGGCATTATGCATGCCCCCTCCCAGTGACCCCAGGCTTTTTATGGCTGTGAGACACGTTAA
AATTTCAGGGGTAAGACGTGACCTTTTGAGGTGACTATAACTGAAGATTGCTTTACAGAAGCCAAAAAAG
GTTTTTGAGTCATGATGCAAGAATCTGGGACTGAGACAAAAAGTAACGGTTCAGCCATCCAGAATGGGTC
GGGCGGCAGCAACCACTTACTAGAGTGCGGCGGTCTTCGGGAGGGGCGGTCCAACGGAGAGACGCCGGCC
GTGGACATCGGGGCAGCTGACCTCGCCCACGCCCAGCAGCAGCAGCAACAGGCACTTCAGGTGGCAAGAC
AGCTCCTTCTTCAGCAGCAACAGCAGCAGCAAGTTAGTGGATTAAAATCTCCCAAGAGGAATGACAAACA
ACCAGCTCTTCAGGTTCCCGTGTCAGTGGCTATGATGACACCTCAAGTTATCACTCCCCAGCAAATGCAG
CAGATCCTCCAGCAACAAGTGCTGAGCCCTCAGCAGCTCCAGGTTCTCCTCCAGCAGCAGCAGGCCCTCA
TGCTTCAACAGCAGCAGCTTCAAGAGTTTTATAAAAAACAACAGGAACAGTTGCAGCTTCAACTTTTACA
ACAACAACATGCTGGAAAACAGCCTAAAGAGCAACAGCAGGTGGCTACCCAGCAGTTGGCTTTTCAGCAG
CAGCTTTTACAGATGCAGCAGTTACAGCAGCAGCACCTCCTGTCTTTGCAGCGCCAAGGCCTTCTGACAA
TTCAGCCCGGGCAGCCTGCCCTTCCCCTTCAACCTCTTGCTCAAGGCATGATTCCAACAGAACTGCAGCA
GCTCTGGAAAGAAGTGACAAGTGCTCATACTGCAGAAGAAACCACAGGCAACAATCACAGCAGTTTGGAT
CTGACCACGACATGTGTCTCCTCCTCTGCACCTTCCAAGACCTCCTTAATAATGAACCCACATGCCTCTA
CCAATGGACAGCTCTCAGTCCACACTCCCAAAAGGGAAAGTTTGTCCCATGAGGAGCACCCCCATAGCCA
TCCTCTCTATGGACATGGTGTATGCAAGTGGCCAGGCTGTGAAGCAGTGTGCGAAGATTTCCAATCATTT
CTAAAACATCTCAACAGTGAGCATGCGCTGGACGATAGAAGTACAGCCCAATGTAGAGTACAAATGCAGG
TTGTACAGCAGTTAGAGCTACAGCTTGCAAAAGACAAAGAACGCCTGCAAGCCATGATGACCCACCTGCA
TGTGAAGTCTACAGAACCCAAAGCCGCCCCTCAGCCCTTGAATCTAGTATCAAGTGTCACTCTCTCCAAG
TCCGCATCGGAGGCTTCTCCACAGAGCTTACCTCATACTCCAACGACCCCAACCGCCCCCCTGACTCCCG
TCACCCAAGGCCCCTCTGTCATCACAACCACCAGCATGCACACGGTGGGACCCATCCGCAGGCGGTACTC
AGACAAATACAACGTGCCCATTTCGTCAGCAGATATTGCGCAGAACCAAGAATTTTATAAGAACGCAGAA
GTTAGACCACCATTTACATATGCATCTTTAATTAGGCAGGCCATTCTCGAATCTCCAGAAAAGCAGCTAA
CACTAAATGAGATCTATAACTGGTTCACACGAATGTTTGCTTACTTCCGACGCAACGCGGCCACGTGGAA
GAATGCAGTGCGTCATAATCTTAGTCTTCACAAGTGTTTTGTGCGAGTAGAAAACGTTAAAGGGGCAGTA
TGGACAGTGGATGAAGTAGAATTCCAAAAACGAAGGCCACAAAAGATCAGTGGTAACCCTTCCCTTATTA
AAAACATGCAGAGCAGCCACGCCTACTGCACACCTCTCAATGCAGCTTTACAGGCTTCAATGGCTGAGAA
TAGTATACCTCTATACACTACCGCTTCCATGGGAAATCCCACTCTGGGCAACTTAGCCAGCGCAATAC**G**G
GAAGAGCTGAACGGGGCAATGGAGCATACCAACAGCAACGAGAGTGACAGCAGTCCAGGCAGATCTCCTA
TGCAAGCCGTGCATCCTGTACACGTCAAAGAAGAGCCCCTCGATCCAGAGGAAGCTGAAGGGCCCCTGTC
CTTAGTGACAACAGCCAACCACAGTCCAGATTTTGACCATGACAGAGATTACGAAGATGAACCAGTAAAC

GAGGACATGGAGTGACTATCGGGGCGGGCCAACCCCGAGAATGAAGATTGGAAAAAGGAAAAAAAAAAAA
CACGTCAAAAGTTAGCAGTGAAATTGTTCTCCATTTGTTGTACAGTCTGGAGGATTTTCACTACGTTTTG
ACAACTCTGAAATGTGTTAACTCTTAGTGCCATCAAGAACCCCATTTGGGAGTATTTTTGATTTTTCTAC
TTTTTGTTGAAAAAAGGAATTTGTACTCTGTGCATTGGATGGACTTGTTTGGTACTTGGGATTTTCCTCT
CTTAACCGTCAACATCAGTGTTGTAAATTTGCTAAACTGATTCACTTTTAGCAGCAGACTTTGAACTGCA
GTCCTGCCAACGTTGGACACTGAGGACGCCCGACAGAGCTTGTGCACCTAAGCTGCAGACCAAGCCTTTG
CCCAGAATTTAAGGATTCCAATGGACGACCTATTTGCACAGTACTGCATGTTGATTATCACTGCCTTTAC
TCCTTTTTTTTTTTTTTTTTTTTTTTTTGCTTCCAGTTGGGATGGGGAAGGCCTTTGTGTGTGTATTGGGG
GGAGGGGTTAAAAAATAATTATCCCAAACTTTTTAATGTATTGCTTTTTTTTTTTTTTTCCTTCTACTAT
ACCATTTTAAGTTCTGACCTCAGGCCTCCATTTGGGCCGATGGCCTCTTGGAGGCTTAAAGTTTTCTGTA
CCTTGTGATGAATGTTAATAGGTGTTTTTATTATACAAAGCTGAATGTCATTTCTCGTTTGTAGCTTTCT
GTCACTCATTCCATCTTCCTTCAGACATCACCACGTTTCTCTAAAGTCAGAAAACATTCCGTTTTGGTCT
TTTTCAAAAAGGTCCCAAATGCTGCACTCTACACATGAAGGCCCTCTCACACAGACGTGACGTCCTGCCA
GAAAGAGAATGAATGACAGAAAAAAAAAAGAGAGACAAACTCTAGGAACAATGCCGATTCATTCCACGCA
GCAGTATTGGGGGTGGTTCGGGGGAGGGGTGTTTCGGATTTTCTTTTTTTCTTTTCTTTTATTTTTTTTT
TTTTGCAGCAACCATTAATAAATGCCACCACATTCTACCAGCACAAGGAAACATAGGCAGCACTGAAAAA
AAAAAAAAAGCTCATATTAATTAGACTGACAATATGGCCTTGGAAGGCTCTCCCTTGTGGAACCAAGTTG
CCATGGGCCTTGGGTGCTCTGCGATAACGGGTGTGGGTTGGTTTTGTTTGCAAAATGGCCAAAAAAAAAA
CCGGCTTCCCCGAGCAGCTGCCCTGAAAGTAGGGGTGGCGGCGGCGGCGCTGAGTTTATACATTAGTTCA
GACCTACTTGGTGGCATTAAACTGTTTGAATGCAAATTCGATTTCAGATTGAACTTGTTAAGGGAGTTAA
CGAGGGCTGAGTTCAGCAAATGCTAAAGTGTTAATTTCAAATATGCAAATTTGGTACTGCAGTTTGTTAT
GCAATATTATATCACCAACCCAGTATCACAAAAACTCATAGAAGATATCATGTAGGCCCTGGGCTTTGGG
GGGGTCCCAAA**CATGGTATGCAGAAATGTGAT**GGTTACAGGTCAGTACAACCTCAGTCCTTAGAACCCCT
CCACACTTCAGCTCTGCACCCACTTTCCTGTCATTTATTTATATAGGACTGTAGTTTTTTTTAGTTCGAG
AGCCTTTCGAAGCTTAATTTATATTCTTTCTTTGTACCTTTTTTCTAAAATTACCAAAGATATTACACAA
AGGTAAATTATGTTCTCTGTTTTATGCTTTATCTGATGAAGCCAAATATCCTCTTATTGTTGATCAAAGG
AGGCAAAAGAATTTAGAGGCAAATGACAAGCGATAGGCTATTGCAACCTGAGAAAGAGAACTGCTCCTTC
ATCGTAAATTTAGAAGACCAAGTAGATAATGGAACCAAAGTTGTACTTTTTTCTAGTAGTTATTTTTCCT
TTTTCTTTTTGTGTACCTCTACAGAGACCAAAACTCATTCTCTTAAAGAGATTTTATGGGGCTACTGCAG
ATAAAAATAGGACACAATATTAAAGGAGCTACAGAAGGAAGGGAGTCCCATCTCAAAAAAAAAATGAATG
TATGCCACTGCAATTAGAGTATCCAATAAAGGAGACAGTTTAGAGTCAGGACAGAAAAGCTTCCATAATT
GAACTAGATTACATAATAGTATTTCTAGAAAAAGAGATATTTTTAGATTGTATGCCACTTTTGTTTAAGA
ACTGTGCTGTGATCACTGTATTAATTTTGGTTTATCTTGGCATATATCCTTCAGTTTGTTTTTATTTTTA
ATTTTTCCTTTTTTTCCGATTAGGCTTTGGTCAGCATTTTTCATTTAAAGAAAAGTAACACTCCCATCCA
CTCATAAGCTTGGTACAAAAACTTCTCTGGCAGTTACTTTTGAAGCTTCACTCTGCTTTCTGTATAAAGG
GCAGTCTGTGGTCACGCAAGACTTAAAAAAAAAAAAAAAAAAAAAAAAAAAAAA

63) Entrez Gene ID 51517 = NCKIPSD, NCK interacting protein with SH3 domain = AF303581

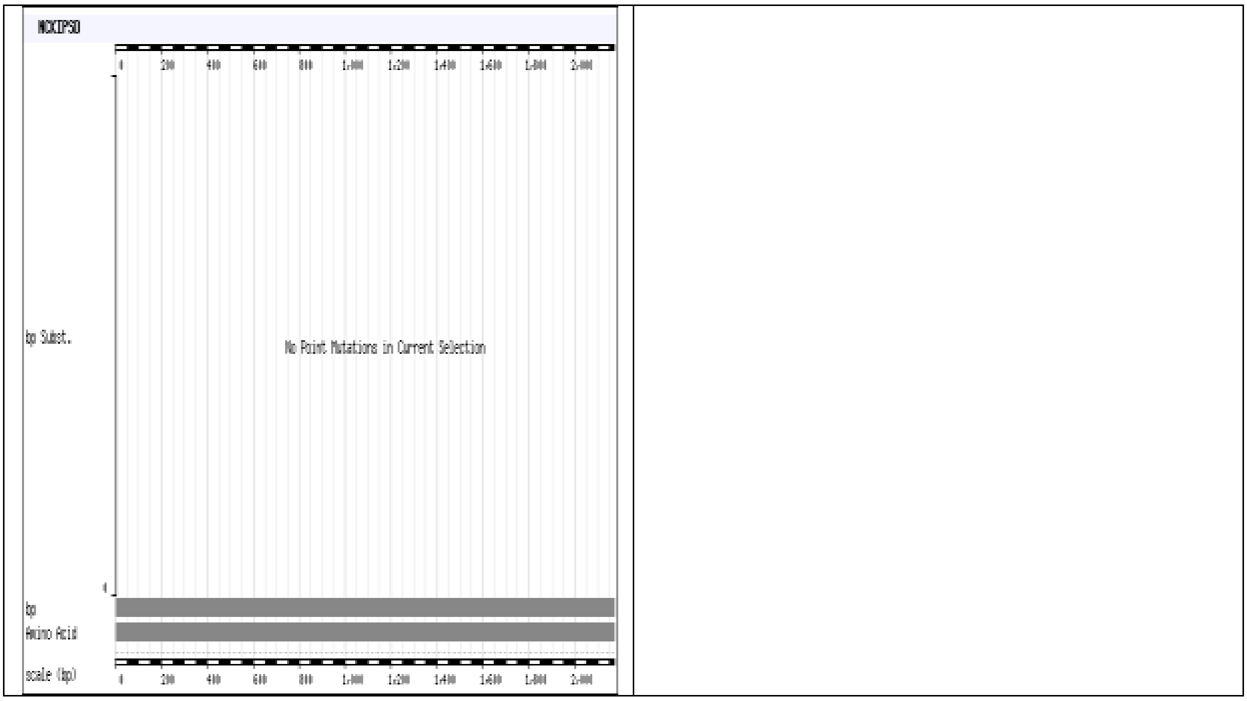

64) Entrez Gene ID 57120 = GOPC, golgi-associated PDZ and coiled-coil motif containing = AY033606

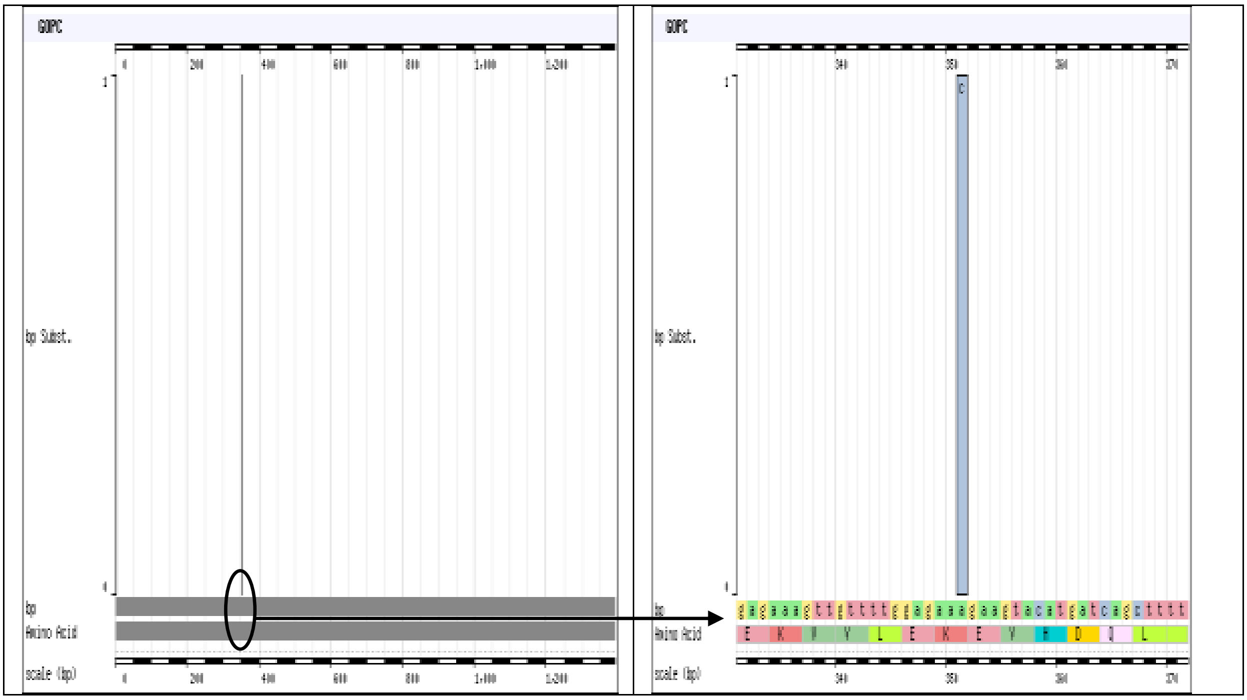

>gi|14289128|gb|AY033606.1| Homo sapiens fused in glioblastoma mRNA, complete cds
ATTCGGCACGAGCCGCCGTTGCCGCCTCAGCTTCAGCCTCGTTACTCCTGCGGTTCTGTGGCTGTGCTGC
TGGCGTTAACGGCGCGAGGTGAAGGGAGGTGACGGAGTGTGCCCGCGCGCGCGGGGGTCCCCTCAGTCCC
AGCAGTTCCCTTCGTGCGCGGGGGGCGGCGAGGTCTTCAGCAGTCGGGAGAGGCCCCTTGACGGCGCCATG
TCGGCGGGCGGTCCATGCCCAGCAGCAGCCGGAGGGGGCCCAGGGGGCGCCTCCTGCTCCGTGGGGGCCC

CTGGCGGGGTATCCATGTTCCGGTGGCTGGAGGTGCTGGAGAAGGAGTTCGACAAAGCTTTTGTGGATGT
GGATCTGCTCCTGGGAGAGATCGATCCAGACCAAGCGGACATCACTTATGAGGGGCGACAGAAGATGACC
AGCCTGAGCTCCTGCTTTGCACAGCTTTGCCACAAAGCCCAGTCTGTGTCTCAAATCAACCCACAAGCTGG
AGGCACAGTTGGTGGATCTGAAATCTGAACTGACAGAAACCCAAGCAGAGAAAGTTGTTTTGGAGAA**A**GA
AGTACATGATCAGCTTTTACAGCTGCACTCTATTCAGCTGCAGCTTCATGCTAAAACTGGTCAAAGTGCT
GACTCTGGTACCATTAAGGCAAAATTGGAAAGAGAGCTTGAGGCAAACAAAAAAGAAAAAATGAAAGAAG
CACAACTTGAAGCTGAAGTGAAATTGTTGAGAAAAGAGAATGAAGCCCTTCGTAGACATATAGCTGTTCT
CCAGGCTGAAGTATATGGGGCGAGACTAGCTGCCAAGTACTTGGATAAGGAACTGGCAGGAAGGGTCCAA
CAGATACAATTGTTAGGACGAGATATGAAGGGACCTGCTCATGATAAGCTTTGGAACCAATTAGAAGCTG
AAATACATTTGCATCGTCACAAAACTGTGATCCGAGCCTGCAGAGGACGTAATGACTTGAAACGACCAAT
GCAAGCACCACCAGGCCATGATCAAGATTCCCTAAAGAAAAGCCAAGGTGTTGGTCCAATTAGAAAAGTT
CTCCTCCTTAAGGAAGATCATGAAGGCCTTGGCATTTCAATTACAGGTGGGAAAGAACATGGTGTTCCAA
TCCTCATCTCTGAGATCCATCCGGGGCAACCTGCTGATAGATGCGGAGGGCTGCACGTTGGGGATGCTAT
TTTGGCAGTCAACGGAGTTAACCTAAGGGACACAAAGCATAAAGAAGCTGTAACTATTCTTTCTCAGCAG
AGAGGAGAGATTGAATTTGAAGTAGTTTATGTGGCTCCTGAAGTGGATTCTGATGATGAAAACGTAGAGT
ATGAAGATGAGAGTGGACATCGTTACCGTTTGTACCTTGATGAGTTAGAAGGAGGTGGTAACCCTGGTGC
TAGTTGCAAAGACACAAGTGGGGAAATCAAAGTATTACAAGGATTTAATAAGAAGGCAGTAACTGACACA
CATGAAAATGGAGACCTGGGCACTGCAAGTGAAACTCCGCTAGATGACGGTGCTTCAAAATTAGATGATC
TGCACACTCTGTATCATAAAAAATCTTATTAAATTGACCATATCTCCAGACAAGATTGTTAATCAGACTA
TTCTGAATTTGGGGCACTGGGGAAGATGGTGACAAAGACTACAAAATCAGGGGAGGCTGTTGTTGCCTAA
ATGAAAGGCGGGTACCTCAGGGCTTCATGTGAACAATTCTCAATGCATAAAACACCCTGTTTTCTGTGGT
AGACCATAATTAGCTATTGCATGTAAAGCAATTTTGATTTTCTTGAACTTGTAAGCACCAAAGCATGTGT
TTCCCAAAGGGGTATTACTGGGCTCTTAAGTACCAAATGAAACCCTACTGTTTTACTTGGTTCCTTTTCC
CTTTAGTAGGAGGACAGTGCTAACTGGAATTTTATAAGGAGTACCCTTTAAAATACAGAGAGTAAGTGAA
TTCATATTTTATATTCTAGAATGTTAACTGTGGTATTTCAGAAGACAGAGCTTGACTCAATGAATTGTAC
AAATATAGGCTTGACTTAATTTTTGTAACTGAAAGGCTTTAGACTTAAAAAAATTTATTGTGCATCCTAA
AATCTAAACCAATTATTAATGATCATTTATTGTAATGAGTTTGCATTTCAGCTTTATTCAATGCAGTACA
AAATAGTTTTCTTTAAGCAGTCTTTTATCAATCAATGCTGCACTAGAAATAGATTCTCAAAAGTTACTAA
ACATTTTACTTTTTTTGTTTTTTAAAAGAAATGCAGATGAGTGTAAAACATCTGTTCTCAATTATGTTGA
TCTGTGTGCGCAGTACTGGAGCATTTACCCATTCATGTTGAGCCTCAAATGCTTGTTTTCTGGGGTCCAC
AAAAGACAGTTTTATACATTTTGAGTTGTTCATAAAGTTTGTCTTGTGATAGTCCTGGCACTTAAAGACA
AATTTTTCTGGTAGTAAAAGTTCAGATTTATTACTATGTCATGAAACACAGTACATTCAAATCAAACGGC
AGTTTTCTTTCTAAGTAAATGATTTCCAGTCATCTAAAAGGTGGGCAAGATGAGATAAAGACATTTTGAT
ACAGTAATTGTTTTGGTTGGGTTTTCATGTCAGTTTATGTTTGACTAAAGCTCTCTTCATATGCAGGTTT
ATAAATTTGTTAGGTCTGTTGTCCCATGATTAAACATGGAGTGCCTCCTCTCTGATTTAATATTCTGCAG
GTCATTGTAACCTGCTAGGCAAAGTCACAACATTGCATTAAAGAGGTGATAGCTTTGCTAATATCACTGT
TTTAAAGGACGTACAGTTAAAGGAATATTAAGTGGGAGAAAGCCTACAAGGTTTTAGAATATTATCAGTA
TCTTCATTTCTGGTATTCAGATGTTATGTGATAAAACACATTTTTTTTGGCTTTCCCAGATACACTATAT
ATTTGTTCAAGGGTAAATCTATAAAATGTATATACTTTATTTTGTGGTTTTGCTATTTATAAATTTAATG
TTTTAACTGTTGCTCATTTATGGTTTGTTTTGGGTGGTGGTGTTCATCTGTATATCACCATGTTAATTTG
TAATGGAAGTGCACTTCGTAGTGTATATTGTTACTGACATTAAAATACTTTATAGCATTGTCTCTGAGCA
AAAGCTAGTATTTAATTGTACAAATGAATAAGCAAGTTACATGTTATTGTTTGCTCTTGACAGGGTAGGC
CTCTTAAAAGAAAAAAAACAACTTGTTTTTTCTTTATGAATCCCCTATGCCAAACACATACCTTCCATGC
ATGACATGAGATCTGCAAACTGGATTTTAGCCACCGTATTTATTTAGTCAAAAAAATTGTCCATTGTAGC
AGACCCGAAAACCTTTTTGCTGTGACATGAAACCATGTTATTCTTATCTTCTTAAAACACAGCCTGGGAT
GGAATGGCCATGGCATTTTTTTCAGAGAACATCCTTTATCTGCTATGACTGAATCCTTAGGAAATGTAAG
CTATAACCCTTTGATTTTCAAGAACTACCGAATAAGTGTATGAAGAGGTGGTTTTTTAAAACTTCAAGTT
GGAATTTTTATGAGGTCACTGTGTAATTTGAAGAATTGTGTGAGATTGTCATGATATAAATTCCTTTTAA
GGACTGATAAATAGAATGAAAAGTTTCCAGGTAGTTTAAAACTCCACAGGTCAGTTTCCTTTTCATTCCT
GCTTCACTGTGGTTTATAAGCCTACGGGAGAGCACCGTTGCTCAGATGCTACTGTGAGCTTCCTGTCCGG
TGTTAGAAAGTAACTAGTTAAAAGTTCATTTTAGAATGTATGGTTTTTGGGGATGAACTAAGAATTAGTT
ATTAGTTCCAAAGGACTGAGAACCAATTTTAATATTTTCACATTTATAGGAAAGAATTCATATGTCCCTG
AAACTTCTAGGACAAAACCAAACAAGTAAGGAGGGAACTGTTGCAAAGCCATTTCATCGAGAAGGGGACA
GAAGGAGAAATACACACATGTATACACAAACAGAATGGTTGAGAAAACGTTTTAATAAAATGTGAGGGTT
GTATGTGTGCGTGTATATATTTACACTTAACCTCTAAAATTCTCTTCTACAGTATCTCTGTTATGAATAT
GATGGAAAAGCAACATTTTGGTGGTGAGACTATTGTTAAAATAAATTTGAGAAAGACGAAAATTTTGTGA
GTCTTTGATAATTACAAGTCAACAGCTATCGAAAGTTAGCACAGCTTGTCTGTGGTGCTGTTTTTTTCCC
CACTGCAGTGGACTTATGCTGTTTTCATGTTTAGAAACAAAAAGGTTTCATGTGATT**CATGTGTAAGATG**
CACAGTATTTGACATCCTGATTATGTAATCCCTATTCCATCAATCCAGTCTTACACTTATGGTTGGCCTC
AAATCTATTGCATTTATGATAATGTATTATATCTAGTTGAGTTTAATATTTTTTTATTAGCCTGTAAATA
AAGATGGCATCTTCTACATTAAAATGATATTGATCTCATTTTTTTAAATAAACATTTTGTTTCCTTGACG

TTAAAAAAAAAAAAAAA

65) Entrez Gene ID 79145 = CHCHD7, coiled-coil-helix-coiled-coil-helix domain containing 7 = AY070434

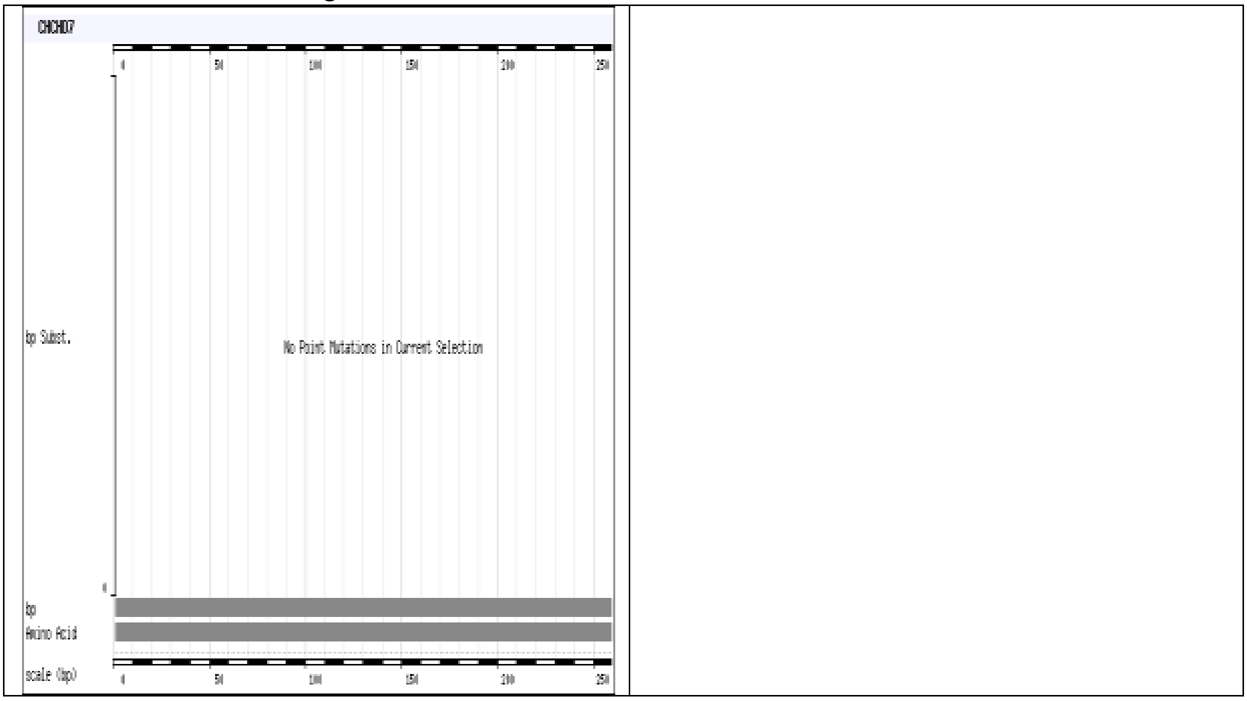

66) Entrez Gene ID 81608 = FIP1L1, FIP1 like 1 (S. cerevisiae) = BC026724

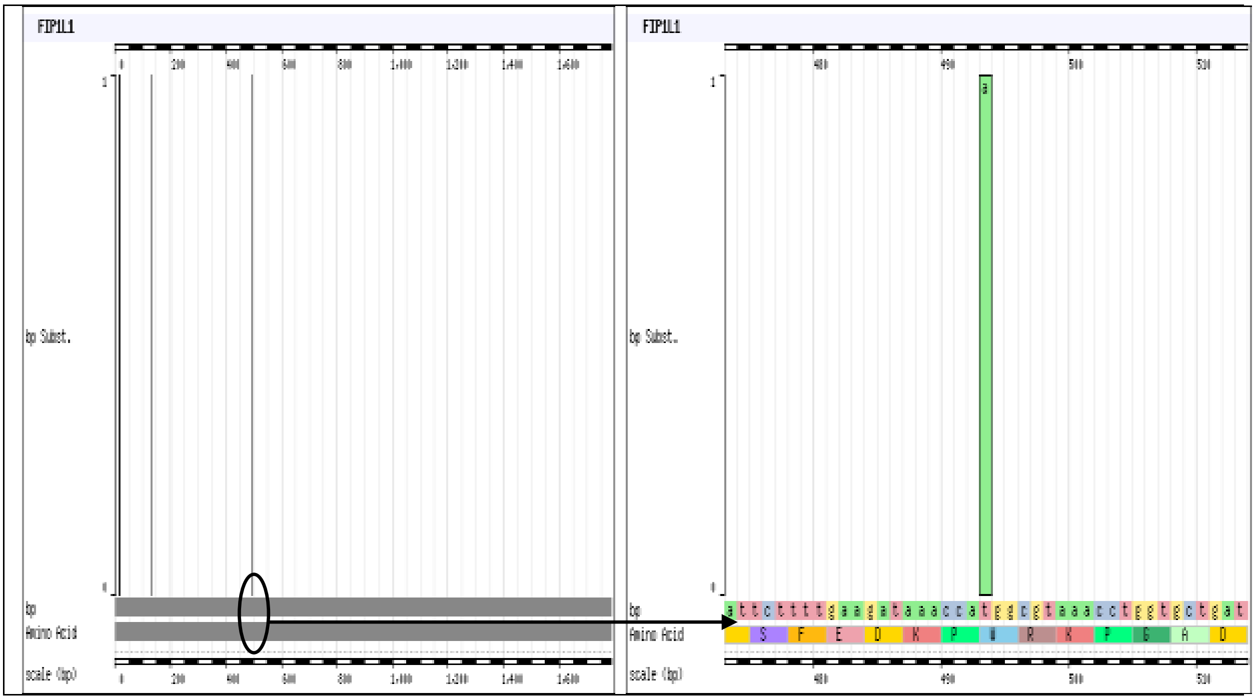

>gi|19934310|gb|BC026724.1| Homo sapiens FIP1 like 1 (S. cerevisiae), mRNA (cDNA clone IMAGE:3918771)
GGCAAATGAGAACAGCAACATACAGGTCCTTTCTGAAAGATCTGCTACTGAAGTAGACAACAATTTTAGC
AAACCACCTCCGTTTTTCCCTCCAGGAGCTCCTCCCACTCACCTTCCACCTCCTCCATTTCTTCCACCTC

CTCCGACTGTCAGCACTGCTCCACCTCTGATTCCACCACCGGGTTTTCCTCCTCCACCAGGCGCTCCACC
TCCATCTCTTATACCAACAATAGAAAGTGGACATTCCTCTGGTTATGATAGTCGTTCTGCACGTGCATTT
CCATATGGCAATGTTGCCTTTCCCCATCTTCCTGGTTCTGCTCCTTCGTGGCCTAGTCTTGTGGACACCA
GCAAGCAGTGGGACTATTATGCCAGAAGAGAGAAAGACCGAGATAGAGAGAGAGACAGAGACAGAGAGCG
AGACCGTGATCGGGACAGAGAAAGAGAACGCACCAGAGAGAGAGAGAGGGAGCGTGATCACAGTCCTACA
CCAAGTGTTTTCAACAGCGATGAAGAACGATACAGATACAGGGAATATGCAGAAAGAGGTTATGAGCGTC
ACAGAGCAAGTCGAGAAAAAGAAGAACGACATAGAGAAAGACGACACAGGGAGAAAGAGGAAACCAGACA
TAAGTCTTCTCGAAGTAATAGTAGACGTCGCCATGAAAGTGAAGAAGGAGATAGTCACAGGAGACACAAA
CACAAAAAATCTAAAAGAAGCAAAGAAGGAAAAGAAGCGGGCAGTGAGCCTGCCCCTGAACAGGAGAGCA
CCGAAGCTACACCTGCAGAATAGGCATGGTTTTGGCCTTTTGTGTATATTAGTACCAGAAGTAGATACTA
TAAATCTTGTTATTTTTCTGGATAATGTTTAAGAAATTTACCTTAAATCTTGTTCTGTTTGTTAGTATGA
AAAGTTAACTTTTTTTCCAAAATAAAAGAGTGAATTTTT**CATGTTAAGTTAAAAATCTTT**GTCTTGTACT
ATTTCAAAATAAAAAGACAGCAATGACTTTATATCCAAAAAAAAAAAAAAAAA

67) Entrez Gene ID 84441 = MAML2, mastermind-like 2 (Drosophila) = CR627398

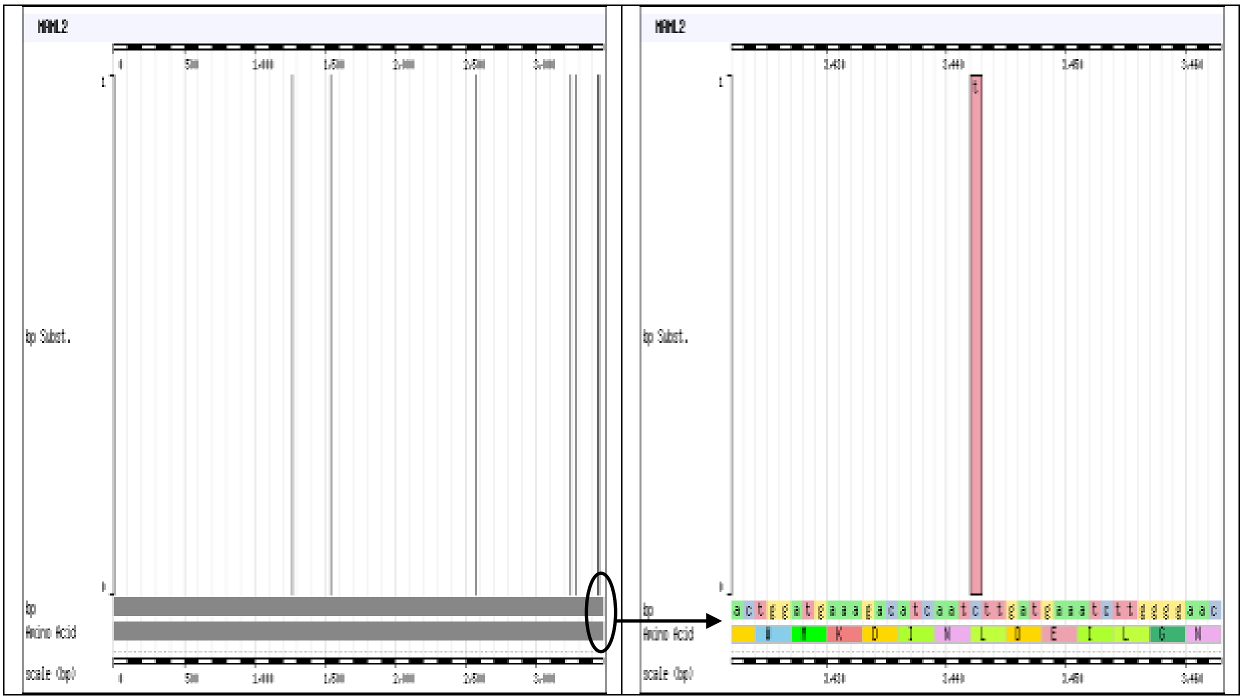

>gi|50949887|emb|CR627398.1| Homo sapiens mRNA; cDNA DKFZp686N0150 (from clone DKFZp686N0150)
AGCTTTGAAAAAGCTGGGCGCCCTTTGGAATACTATACACAATTCAAGGTGGATATTGAGGTTTTTCTTT
TCCTACTCACATAAAAGGATCCCAGGCACAGTAGTCTAGGGCTGGCTTGCTGGATTTCAAGCATCTTTTA
TCTTTCCACTCCATCTCTTTCAGCATGTGGCTTCTATCCTAAGGGCTGCCTGGTAGCTACCACATCACTA
GTCTCATACCTGTATTCCAGGTAGGAAGAGGAAAGAAAAGAAATCTGATTCCCTTAGCCAGAACATATTC
ACCTGGACCCTCCCGCCCACCCCATCGACAAGGAAGTTTGGGGAATGTGGTTTTTCAGCTGAGCACATTG
CTCTGTTTCTAAAGAAGGCTCTGTTTCTAAAACCAAAGCTCTGTTTCGAAGGAATATAGAGAGGTTGGAT
ATTGGGTAGGCACCTAGCAGTCTCTTCCACAGTGAATATTGGTGTTTTTCTAAGGGAGAAATCCTTAGCA
TCCAACTGCTGACACTCAAGTGGGTCAGAATCATTATTCTAGAGCATTGCGCTTCCCATGTCTTCTGATG
ATGAAATAAGCAACAAAACGCAATACAAACTCTTTTTAAAATCATTTTTTATTTTAATCTTGGTACAATA
ATGATTACCTTTCCCAGATTTTGATATTAAAAATGGAGTATGAAAAGCACCTGGAACAGTCTCTGTCACC
TGTTAGGAGCTTTTAAACATTAACTGTTACTATTTTGTGATCTTTGTGGAAATTCTCTCTATTCTGGTTG
TTTAAAAAGATTGTTTTCCTTATAAACCTTCTCAGGCCATATCATTAAAATACAGGGCGACTACAGGTGA
TAATTTTTAAAACCTCTACATTTTTTTCCACAGCATTTTCATAATATAAATTCCCTTGATATGAAACTTT
CAGATACAAAGATGATATTGCTGAATCTTACTGGCATTGTATGTCATCTCAGTTATTGTTCAAGTGTCTC
CTACCTGGCTGTTATGTCTCTTCATCAAACACTGCCCACTACACAATGCACTACACAGATACAAGGAGAT
ACAAAAGACTGTTCTTGCCTTCAAGGGGTATGTAGTCTAGTTGGGGGCATATTTATAAATAAATAGCAAA

GCTAATGTGTTCATTTGGCCCTCAGTTTTTCAGGATGTATACCACCTACGTATCCTTAAGCAGCAGGGGA
TAGCCCACGTTTACAATTGCAGACATGCTCTCCCCATCCACTGACATTGTGATATTCTTATGTGTATTTA
GCTCTGTATTTCTCAGAATCTCTGACTCTCTGGGCCTCCCTGATCATCTTTGCCTCCAAAAATATTTCCT
CGTGATGACTTTAACCTCCAGAAGTCAAGAAGATTTCAATATGTTGATTAGTCACTATCTGTTGTGTGGA
GCCTCTTATTACACAGCCTGTGGCTTAGGCACTTGTTCCATATTGTTGCAGAACTTTCTCCTTAGTTCAG
CTAAAACCAGGCTCTCGTCACATGGCAAGGAAAGATTAGGTTCGTGGACACATGGAAGGATGAGGAAAAA
TGGAATTTATTCGGCAAAAAGGAAAAATAACTCTCAGCAAAGTGAGAGAGAGTCCTGCTAGCAGGTTTCC
CACCTCACAGATTGAATTCCAGGTCACCACACAGGAACAGGAGAGGCCAGGCTCCTCACTGCTGCAAATG
GCACGAACTTCCCAAGGCTCCACCCTGTCTTCCCAGTGCACAGGTGGGCATTATTCAGAAAGAGTCAGTC
GGAAGCGGTCTTCATCCAGGACCAGCAGTCCAGTTTTTCAGCCTTCAGGCTGTGTTAGGTTTGAAGGCGT
GGGGGAGACCCTTGTCTGCCTCCTGGGTCTCTGTCAATATCCTATCAGTTATTGACAGGCATCAGTTTAA
CTGGCAGGCACTTTGAGACTCCACAGATTGCCCAATACTAAGTGTTAAATGAAAGATTCGAAGGGCAAAT
ACTGCTGGAGAGAGGAGGCTGGAAGATAAAAGAAAGTTTAATGGAGGAGCTTCGGCCTTAGCCTGAACCT
TGAGGAAAATATAGGGCTCAGTGAGGAAGGGAAATCCCATAAGCAAAGGTGGCTCACAGTGCATTGAGTA
ACAATTTGATAGTAACTACACCTCCTGGAACTGAAGAATTTCTTTATGAGATAATTTCTAGAGCACTGAA
TTCCAGATGTTTAAAGAGATGGAGATTTACCCTTTGAACACTGGGGAGTCACTGGGGTAGCTTCTTGAAT
GGGGATGATTCAAAGAATGGGACATGATTTTCAAGGCCATAGTTTGGGAATATTAATCTAGGAGCCGTGT
GTAGCATTCATCCACACTTTCTCATTTGGACCTGGGGTAAACAGTAACATGGATTTCATGAGAGCTGACT
AATCAAGACTGTAGTCTCTGGACTTGGAACATAGTAAAGCTAATTCAAAATGGAAGATTGACTATTACCT
CCCTTCAATGACTTCAGAAGAGGAATAAAATAAAACCTACAAGATAGAACTATTCTATTTTAGTCTTCTT
TTTGTAATATGTAAACTTCTGAACCAAAAAGTGACCTCAGGTGCTCCTTCCTCCTAAGAGCTGTCTTGTT
CCTTTTATATAATTTATTTGATCATTATTTCGCCTAAATTGTCTTACACCTGAAAGCAGTTTCAGATTGA
TGAGTTATTGAAAAGTGCAGTTACAGAGGAGATAACATGCTGCTACGAACTAATAAAATTCTCTTCTTTT
TCTCAGGTGGCTCATCCACAATAAGCTTAAACTCTAACCAGGCTTTGGCAAACCCAATTTCAACACACAC
CATTTTAACTCCCAATTCCAGCCTCCTGTCTACTTCTCACGGGACAAGAATGCCATCATTATCTACAACA
GTTCAGAATATGGGGATGTATGGAAATCTGCCTTGTAATCAACCTAACACATACAGTGTCACTTCAGGAA
TGAATCAATTGACCCAACAGAGAAACCCAAAGCAATTGTTAGCAAATCAAAACAACCCTATGATGCCACG
GCCACCTACCTTAGGGCCAAGTAATAATAACAATGTAGCCACTTTTGGAGCTGGATCTGTTGGTAATTCA
CAACAATTGAGACCAAATTTAACCCATAGTATGGCAAGCATGCCACCACAGAGAACATCAAACGTAATGA
TCACATCCAACACAACTGCACCAAACTGGGCCTCTCAAGAAGGAACAAGCAAACAGCAAGAAGCCCTGAC
GTCTGCAGGAGTCCGCTTCCCCACAGGTACACCTGCAGCCTATACCCCAAATCAGTCACTGCAACAGGCA
GTAGGTAGCCAGCAATTTTCCCAGAGGGCAGTGGCTCCTCCTAACCAGTTAACACCAGCAGTGCAAATGA
GACCCATGAACCAAATGAGCCAAACACTAAATGGGCAAACCATGGGTCCCCTCAGGGGTCTGAATCTCAG
ACCCAATCAGCTAAGCACACAGATTTTGCCTAATTTGAATCAGTCAGGAACAGGGTTGAATCAGTCGAGG
ACGGGCATCAACCAGCCACCATCCCTGACGCCCAGCAATTTTCCTTCACCCAACCAAAGTTCCAGGGCTT
TTCAAGGAACTGACCACAGCAGTGACTTAGCTTTTGACTTCCTCAGCCAACAAAATGATAACATGGGCCC
TGCCCTAAACAGTGATGCTGATTTCATTGATTCTTTATTGAAGACAGAGCCTGGTAATGATGACTGGATG
AAAGACATCAAT**C**TTGATGAAATCTTGGGGAACAATTCCTAAAGAAGAAAGGGAAGACAATTTACAAACT
CCAAGCACTAAAAGGCAGTATATTACAGAAACTCTGTAGAGGCTGAACTGTTGATGTTCAGGTGGACTAC
ATGAAGATAACATGCTTAAAAATGGAAAGCAGAAAGTAACTGCAGTGATGAACATTTTGGTCCAAATTCT
TGTTTTAAATCTTACACCTGAAAGTAAAATATTGGGATCACTTTTCCCTGTCTAAACTCCAGGATACAGT
ATCCAATTTATCCAAACAGAACTGTGGTGTCAATGTGTAATTAATTGTGTAAAATAGCCTTCCCAAGTTT
CTTTTTCCCTGGAAAATAAAAAAGGTAATAGAACTTGTAGTTTATTTAAACCCCATGTCATGAGGAGGTA
CTAGTTCCAAGCAACAAACTCCTTAATTTGCTCTAATAGATAGGTATGGTTTAATCTTTCCATTGTGTCT
TTTCATTTAATTTTCCTGAAGCTTGCAGGATAGATTGAAATGTTATAGGTTTGTTTGGAGTAACCAAACA
GTATGCAAATTAAGAAAAAGCCAGAGAACCTAGAAAACATCCAGTGGATTACAGAATTTCTTCCCCATAT
TCACTCCTCACTTTTACAATTTTCCCACAATCCTCTACTTCAGTGGGACGCTGTGTCTAGTGATTAAACA
AAAATATAGAGCTGTGCAATTTGATTTTGGCTTCCACAACGAATATCTGAATCCATTCCAAATGAAATTT
TAGATATAACAAAGACTTGTCCTAATCATACTGAAATATTGGTGCACACCTCTCTGCATTAGATTTCACT
TTTTTAAAAAACCCAGTGGACATTGCTATAAATAAGATTTATTTGGCTACAAATAACCTGGGATGTTGCT
TATTATGATTGATGCCTGCTGGTTTGTTCCCAAGCTGAGTGAAATTGAACCTCGTCCTCCCTACTCATTT
TGATGACTGAGGCTGGTTTATAAGAAAAGGAAGTTTGGAGAAGAAAACCGAGATTAGAAAATATCATGTT
TTGGTTGGAGATAAGAACCAGGGATGGCAAGTACCAGTGTGTACAAATGTATTTCACGGAGTTTGAAGGA
ACGCATAATCAAGAGGGAAAACAATTTGTCCTTCATTGGACGTATTATTTGGATTTGGGTGAGCAACAAA
ATGGAATGTGGTCTGTTAGGAGCATTCTGTTTGTTCTTTTGTCCCTGATGTGATGAATCATTGCCACATG
CTAGATGGACTCTTCATATCCAGGTTTTGTCCCTCAGGGCTGAGCACTGTATTAAAGAGTTTTTGTTGAG
TCATTTAACCTTAGTGTCCACATCCAGATCAGCTGTAAAATGGGGAAGACGTGTGCTGATTTGGAATGAA
TGCAAAATATCACTATCATTTTCCTAATTACAGAGGAGCAAAGGTTATCTTCAGCCCTTTCAGTTCTATG
CTCACATATTCAAATATCAAATGTAATTTAGCTGAAGTTATTTAATAATCAAGTCTTTCAATATCTGTTC
AAAGAAAAAGAACACACTTTGAAAATTCTGCAAAGCTGTCTCCCAGTCTTTAAAATGTCTGGAAGCACTC
TCCTTCTTTACAATACCAACATCACTGGCCCAGAATCTTCCCTGTGCTAGTTTGTAAATATAAATAAATT

ACTTGTTTTGTAAACTTTTGTAAAGAATATTTTGGTAGAAATACTTCAAACATATTCTTTGGGTTATATT
TATACATATGTGAAATAAATATACTATCAAAAGGTTATATTTTATACAAAAAGTAAATTGCTACCTTTTG
TATGCTAATATGCAAAGTTTTGTATAATATGATGGTTTATTTTTAGCTCTACACTTAAACCATAGGTGGT
TGAGTGGGAACTTTTGAAAACTATCAAGAGGCTTGTTAGACAAATTTATATTCTGAAACCTCAATAAGAA
AGCATTCCAGGTTTCAATCCTTGTTTTTTGTCCTGCTCCCAAATTCTTTTTTAAACCCATAGTTCTTGTG
TCTTATTTGATTCTTCTGCTGTGCACATTGTATTGGTCCTTGTTGCATGTAGTCTACTGTGTGTTTTCCG
ATTTTATAAGGCAGCATTTCTCCATACAAAAAGAAAAAAAATGATGTACATATAAACGCTTTTGTTGTAT
GGCTCCTC**CATGTTACTGTATATATCTGC**CAGCACTTCCCAGTTACACTCCTGTGAGTCAGCTTATTTTT
ACCCTAACATAAATAGTATGTTTTGTAGTAGTTATCAAATTTAAGAGATAAAGCAATCAGAATGTTTGGA
TTTTCTTCTATCTTAATGTGAATTTCATAATTAATGTCTATTTATTCAGCTATTCATTAAAATACAGGAT
TCTTTGGGAAAACAATAAAAAAAAAAAAAAAAAAA

68) Entrez Gene ID 116028 = RMI2, RecQ mediated genome instability 2, homolog (S. cerevisiae) = BC013040

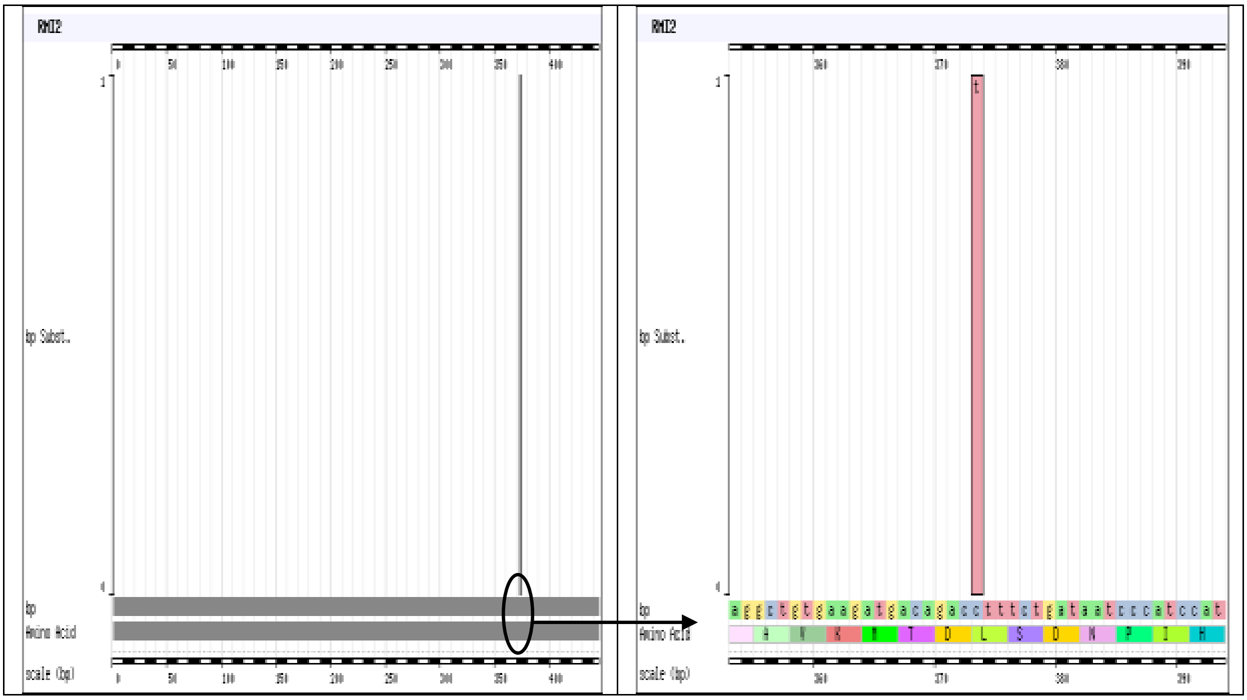

>gi|38197470|gb|BC013040.2| Homo sapiens chromosome 16 open reading frame 75, mRNA (cDNA clone MGC:4772 IMAGE:3542171), complete cds
GAATGGCGGCGGCTGCGGACTCGTTCTCAGGCGGCCCCGCGGGGGTGCGGCTTCCGAGGTCGCCGCCACT
CAAGGTGCTGGCGGAGCAGCTGCGGCGCGACGCGGAGGGCGGCCCGGGCGCGTGGCGGCTGTCACGGGCG
GCGGCGGGCCGCGGGCCGCTGGACCTGGCGGCCGTGTGGATGCAGGGCAGGGTAGTGATGGCGGACCGCG
GCGAGGCTCGGCTGAGGGACCCGAGCGGGGACTTCTCGGTCCGCGGCCTGGAGCGGGTGCCGCGCGGGCG
GCCCTGTCTAGTCCCAGGAAAGTATGTGATGGTGATGGGAGTGGTTCAGGCCTGCAGCCCTGAGCCCTGC
CTGCAGGCTGTGAAGATGACAGAC**C**TTTCTGATAATCCCATCCATGAAAGTATGTGGGAACTGGAGGTAG
AAGATTTACACAGGAATATTCCTTAGAGTATGTTGGAACTGTCGTTAAAAACAACCAAAATCCCGAAACT
ATTTAGAAGCTTATAATGATGTGGGTTTCATGGACACTTTTCAATGCGTATTTTTCAAATGCTTCTCAGA
GAGCCTTGCTTTGGTTGACCAAGGAGTCCGGATGTAGGAATGTTTAAATCCTCGGATACTTCAGTGACAC
AGCCTCTGCTGCCCCTTGCTTTGCCTGTGTTTGCTGATGAAAAGCAGATGCTTGTGTTTCATTTTCCTTC
CTGGTTTGTGTGTGTTAATTCTCTCTCTCTCTCAGACACAGAAGTCTCATGTTGCATTTTCCAAATTT
TATGAGTGATGATACTTTTTCCATTACTGCTGCGTCCCTGTTTTACAATGCAAAATTTAAGTACGGTCAT
TGCCCATGGTGATTAAAGTGTGGTTATGGGCAGGAAGACAGACTGTGTAAAAAAGGAATGACATCCTGGC
TCCTCATCTTCTTCATCAGCAACTACCATAACCAGTTTGCGAGTCAAATGGCATTTCCTAACGGCAGGCA
TGGCGGCCCCTGAAAGACAACAGCTCCCTTTCTGCTTCGGACACCACTCAAACATTTAGACGCAGCTCTA
TCCCTTTTCCTAGCTAGAGAAGGTGATGCCTTCTTCCATTACTCAGAGATGTTGAGACGTTTTCAGAATT
TCTTGTTGAAATGAAAAACATCAAGATAAAGGACGCCTTTCAGGCATTAGCTAAACTTCCACTTCATAAC
TTTCGGCGAGACGTGGTGAGCCTCCTGGTGTAGAGTTCTTTTGTCTTTGTATGGAATGACTTTTTGCTGT
GATGGTTTTGAATGTTGGGTTTCTGCTGTCTGCTTAGTACCCATGCCTGAATTTTTTGAGATTGTAAATA

TCAAAGGAGTTAGATTGTGTCCTGA**CATGGTTGTAGACTTTCACCT**GGATTATTGATATTCTACCTCTAA
TAAATTTTTAATAGGCAAAAAAAAAAAAAAAAAAAAAAAAAAAAAAAAAAAAAAAAAAAAAAAAAAAAAA
AAAAAAAAAAAAAAAAAAAAAAAAAAA
