# Supplementary material for: Increased frequency of single base substitutions in a population of transcripts expressed in cancer cells
Source: BMC Cancer. 2012 Nov 8;12:509. doi: 10.1186/1471-2407-12-509 (PMC3522053; doi:10.1186/1471-2407-12-509)
Supplement: Additional file 8 — Manual checking of possible APOBEC1 RNA-editing targets altering RT with greater SBS frequencies in cancer than in healthy cells (L-SAGE). [file 1471-2407-12-509-S8.pdf]

# Additional_result_file_3 to support figure 3a

APOBEC1 RNA-editing targets (predicted or experimentally validated by Rosenberg B.R. *et al*, 2011) x L-SAGE_372_RT_CgtH_FDR
=> 5 transcripts were in common between both lists, namely 4942, 9741, 10049, 10492 and 27018 (NCBI Gene IDs)

NB: Rosenberg B.R. *et al*. study was carried out in mouse

## Orthology table:

| Index | APOBEC1 RNA-editing target(mouse) | Gene Symbol | Description | Human orthologue (blastn first hit) | Blast Score | E value | Gene ID (human) |
|---|---|---|---|---|---|---|---|
| 1 | NM_016978 | OAT | Ornithine aminotransferase | NM_000274 | 1027 | 0.0 | 4942 |
| 2 | NM_008640 | LAPTM4A | lysosomal protein transmembrane 4 alpha | NM_014713 | 1039 | 0.0 | 9741 |
| 3 | NM_001037940 | DNAJB6 | DnaJ (Hsp40) homolog, subfamily B, member 6 | NM_058246 | 860 | 0.0 | 10049 |
| 4 | NM_019796 | SYNCRIP | synaptotagmin binding, cytoplasmic RNA interacting protein | NM_001159677 | 5765 | 0.0 | 10492 |
| 5 | NM_001110234 | NGFRAP1 | nerve growth factor receptor (TNFRSF16) associated protein 1 | NM_206917 | 329 | $7e^{-89}$ | 27018 |

## RNA-editing table:

| Index | APOBEC1 RNA-editing target(mouse) | RNA editing status | Mouse mooring sequence in transcript | Human orthologue | Human mooring sequence (By similarity) | 17 base *NlaIII* tag (RT) In human transcript | 17 base NlaIII tag edition by APOBEC1 |
|---|---|---|---|---|---|---|---|
| 1 | NM_016978 | predicted | acaguuaaaucauuau | NM_000274 | aaagtgaaatcattgttat (conserved) | TGTACTTCCTAAGGTGA | No, because NlaIII tag is too distant from the mooring sequence |
| 2 | NM_008640 | predicted | ucauugguaauuacuau | NM_014713 | tcatcggtaattactat (conserved) | TTTCTAGTTTGTATATG | No, because NlaIII tag is 3' to the mooring sequence |
| 3 | NM_001037940 | predicted | acuucaaaucaauau | NM_058246 | acttcaaatcgatat (conserved) | TATTTTAAATGCCACCT | No, because NlaIII tag is 3' to the mooring sequence |
| 4 | NM_019796 | predicted | ucaggaaaauuauuau<br>ucauagaaauuaguau | NM_001159677 | aaagtgaaatcattgttat (conserved) | GCAAACAATCATTTTGC | No,because NlaIII tag is 3' to the mooring sequence |
| 5 | NM_001110234 | predicted | acugaauaaucauuau | NM_206917 | not conserved | GAAAAATTTAAAGCTAA | Unlikely, because the mooring sequence is not conserved between mouse and human |

## Legend:

Red nucleotides = mouse mooring sequence
Violet nucleotides = human mooring sequence (by similarity)
Green nucleotides = 17 base *NlaIII* tag in human transcript
Blue nucleotides = *NlaIII* “CATG” recognition site
Underlined nucleotides = Stretch of bases in which C to U edition could occur (3’ to the mooring sequence)

### 1) OAT, Ornithine amino transférase

```
NM_016978      1795 ---acagttaaatcattataatcaatgaatgtt---aagttgattgaagg   1838    (Mm)
                       |.|||.||||||||.|.||   |||||.||   |||  ||||.|.||
NM_000274      1847 tataaagtgaaatcattgttat---tgaattttaggaag--gattaatgg   1891    (Hs)
```

```
>gi|284507296|ref|NM_000274.3| Homo sapiens ornithine aminotransferase (OAT), nuclear gene encoding
mitochondrial protein, transcript variant 1, mRNA
TTGGGGGCGGGGCAGAATCAGCCTTTAAGTTGCAGTGACTCTCCGGCGTCACTGTTGCGCTTCATAGACG
CCGCGTGTACCCGGTTGTCCTCAGGCGCTGTCAGATCTGTGGTTTTTCTACTTGAAGGACACAATGTTTT
CCAAACTAGCACATTTGCAGAGGTTTGCTGTACTTAGTCGCGGAGTTCATTCTTCAGTGGCTTCTGCTAC
ATCTGTTGCAACTAAAAAAACAGTCCAAGGCCCTCCAACCTCTGATGACATTTTTGAAAGGGAATATAAG
TATGGTGCACACAACTACCATCCTTTACCTGTAGCCCTGGAGAGAGGAAAAGGTATTTACTTATGGGATG
TAGAAGGCAGAAAATATTTTGACTTCCTGAGTTCTTACAGTGCTGTCAACCAAGGGCATTGTCACCCCAA
GATTGTGAATGCTCTGAAGAGTCAAGTGGACAAATTGACCTTAACATCTAGAGCTTTCTATAATAACGTA
CTTGGTGAATATGAGGAGTATATTACTAAACTTTTCAACTACCACAAAGTTCTTCCTATGAATACAGGAG
TGGAGGCTGGAGAGACTGCCTGTAAACTAGCTCGTAAGTGGGGCTATACCGTGAAGGGCATTCAGAAATA
CAAAGCAAAGATTGTTTTTGCAGCTGGGAACTTCTGGGGTAGGACGTTGTCTGCTATCTCCAGTTCCACA
GACCCAACCAGTTACGATGGTTTTGGACCATTTATGCCGGGATTCGACATCATTCCCTATAATGATCTGC
CCGCACTGGAGCGTGCTCTTCAGGATCCAAATGTGGCTGCGTTCATGGTAGAACCAATTCAGGGTGAAGC
AGGCGTTGTTGTTCCGGATCCAGGTTACCTAATGGGAGTGCGAGAGCTCTGCACCAGGCACCAGGTTCTC
TTTATTGCTGATGAAATACAGACAGGATTGGCCAGAACTGGTAGATGGCTGGCTGTTGATTATGAAAATG
TCAGACCTGATATAGTCCTCCTTGGAAAGGCCCTTTCTGGGGGCTTATACCCTGTGTCTGCAGTGCTGTG
TGATGATGACATCATGCTGACCATTAAGCCAGGGGAGCATGGGTCCACATACGGTGGCAATCCACTAGGC
TGCCGAGTGGCCATCGCAGCCCTTGAGGTTTTAGAAGAAGAAAACCTTGCTGAAAATGCAGACAAATTGG
GCATTATCTTGAGAAATGAACTCATGAAGCTACCTTCTGATGTTGTAACTGCCGTAAGAGGAAAAGGATT
```

ATTAAACGCTATTGTCATTAAAGAAACCAAAGATTGGGATGCTTGGAAGGTGTGTCTACGACTTCGAGAT
AATGGACTTCTGGCCAAGCCAACCCATGGCGACATTATCAGGTTTGCGCCTCCGCTGGTGATCAAGGAGG
ATGAGCTTCGAGAGTCCATTGAAATTATTAACAAGACCATCTTGTCTTTCTGAGGGTAGCCAGCTGTTTT
CAGTGGTCCCTGGGAGCCAGCTGGAGACAGGTGGTCCTGTAAAAGCTTTATTCCTAATGTGGGCACATTC
CACTCCCATGAGTCTTCAAAAACTTTTTTTTTGAATATATTTTTTTCAGTTGATACATAATAGAACAACG
TTTATGAACCTGCCGTTTGCTTTGTAACGTAACTAAATAATGTAATGGCATCTATATTCAGTTGAAGTGT
TTTGATGTG**CATGTGTACTTCCTAAGGTGA**AATGCATCTATATACAGACAGCCTCTAAATCAAGTCCTTC
AGTATAATTGATATATGTTTTTATAATTTCCTCACTGGTATAAGTGTTTCATATTTGAAAAAGTTATCTC
TGGGTATTGCATAAAAGGCTTCATCTTAT**AAAGTGAAATCATTGTTAT**TGAATTTTAGGAAGGATTAATG
GTTAAGTGTATATAAAATACTAATATTAAGTAAACTTCATATTGGCCAACACCAGGGTTGTATTCTATGG
ATGTCATTATTTTGAATTAAGAATTAGTGTTTAACATTCCTAAATTGTTTTGAGTGCTTGATTATAATTT
GTAAAAAATGTTTATTTTCAATACTTCTTTAAATTTAAAATAAAGCTTATATTTCAAATGTCAAAAAAAA
AA

**2) LAPTM4A, lysosomal protein transmembrane 4 alpha**

NM_008640 1065 agtatgaag**tcattggtaattactat**tcatctgagagaatggctgataat 1114 (Mm)
|||||||||||||.||||||||||||||.|||||||||||||||||||||
NM_014713 696 agtatgaag**tcatcggtaattactat**tcgtctgagagaatggctgataat 745 (Hs)

>gi|196049387|ref|NM_014713.4| Homo sapiens lysosomal protein transmembrane 4 alpha (LAPTM4A), mRNA
ATCCAGTACGTGGCACACATTGCTCAGAAAAGCCCCTTGTGCCCGCTTTCTGCCATCCGCGCTGTCTCGT
GTCTCCCTTTTTCCATTAAATGCCTCTTTTCTTGCGGGTCTCATCTCGGGAATAGTGCACTACGGGGACA
TACCTATCCCCAACTATCCTAGGCCCGAGAACCAGCCCTTGCCTTCGCGTAACAGGCGGAGACTCGCTGA
GGCGAGTTGCACTTCTAATTGGGCGTGAGGTCTTGTCAATCCCCAAGTTCTTCCAATCAGAAGTCCGGTC
CATCCAGCCTTCCGCTCCCCATTGGCCTGTGTGGAGGAAGAGGGGTGGGTAAGCCGAAGTCGCTGCGCTC
AGTGCGCAGGCGCGAAGAAGCTGGCAGGGGCACGAGCCGGGGGCGGGTTTGAAGACGCGTCGTTGGGTTT
TGGAGGCCGTGAAACAGCCGTTTGAGTTTGGCTGCGGGTGGAGAACGTTTGTCAGGGGCCCGGCCAAGAA
GGAGGCCCGCCTGTTACGATGGTGTCCATGAGTTTCAAGCGGAACCGCAGTGACCGGTTCTACAGCACCC
GGTGCTGCGGCTGTTGCCATGTCCGCACCGGGACGATCATCCTGGGGACCTGGTACATGGTAGTAAACCT
ATTGATGGCAATTTTGCTGACTGTGGAAGTGACTCATCCAAACTCCATGCCAGCTGTCAACATTCAGTAT
GAAG**TCATCGGTAATTACTAT**TCGTCTGAGAGAATGGCTGATAATGCCTGTGTTCTTTTTGCCGTCTCTG
TTCTTATGTTTATAATCAGTTCAATGCTGGTTTATGGAGCAATTTCTTATCAAGTGGGTTGGCTGATTCC
ATTCTTCTGTTACCGACTTTTTGACTTCGTCCTCAGTTGCCTGGTTGCTATTAGTTCTCTCACCTATTTG
CCAAGAATCAAAGAATATCTGGATCAACTACCTGATTTTCCCTACAAAGATGACCTCCTGGCCTTGGACT
CCAGCTGCCTCCTGTTCATTGTTCTTGTGTTCTTTGCCTTATTCATCATTTTTAAGGCTTATCTAATTAA
CTGTGTTTGGAACTGCTATAAATACATCAACAACCGAAACGTGCCGGAGATTGCTGTGTACCCTGCCTTT

GAAGCACCTCCTCAGTACGTTTTGCCAACCTATGAAATGGCCGTGAAAATGCCTGAAAAAGAACCACCAC
CTCCTTACTTACCTGCCTGAAGAAATTCTGCCTTTGACAATAAATCCTATACCAGCTTTTTGTTTGTTTA
TGTTACAGAATGCTGCAATTCAGGGCTCTTCAAACTTGTTTGATATAAAATATGTTGTCTTTTGTTTAAG
CATTTATTTTCAAACACTAAGGAGCTTTTTGACATCTGTTAAACGTCTTTTTGTTTTTTTGTTAAGTCTT
TTACATTTTAATAGTTTTTGAAGACAATCTAGGTTAAGCAAGAGCAAAGTGCCATTGTTTGCCTTTAATT
GGGGGGTGGGAAGGGAAAGAGGGTACTTGCCACATAGTTTCCTTTTTAACTGCACTTTCTTTATATAATC
GTTTGCATTTTGTTACTTGCTACCCTGAGTACTTTCAGGAAGACTGACTTAAATATTCGGGGTGAGTAAG
TAGTTGGGTATAAGATCTGAACTTTTCATCTGCAGAGGCAAGAAAAATATTTGACATTGTGACTTGACTG
TGGAAGATGATGGTTG**CATGTTTCTAGTTTGTATATG**TTTCCATCTTTGTGATAAGATGATTTAATAAAT
CTCTTTAAATACTTAAAAAAAAAAAAAAAAAAA

**3) DNAJB6, DnaJ (Hsp40) homolog, subfamily B, member 6**

```
NM_001037940     780 acgtcatttggcggcagtggaatgggcaacttcaaatcaatatcaacttc    829   (Mm)
                     ||||||||||||.||.|||||.||||||||||||||||||.|||||||||||
NM_058246        686 acgtcatttggtggtagtggcatgggcaacttcaaatcgatatcaacttc    735   (Hs)
```

>gi|34328906|ref|NM_058246.3| Homo sapiens DnaJ (Hsp40) homolog, subfamily B, member 6 (DNAJB6), transcript variant 1, mRNA
GAGAAAGGAGAGAAAGGAAAGCGCGAGGAGCCGCCGCCACCACCAGCGCAGCAGTCCTGGAGCTGTGAGG
AGATTCGGGCCGTCACCCTGCCTCCCCTGCGTCCCGCCACCGGCCGCTTCTGTCCTCGGACCCATTCCAA
CAATCTCGTAAAACATGGTGGATTACTATGAAGTTCTAGGCGTGCAGAGACATGCCTCACCCGAGGATAT
TAAAAAGGCATATCGGAAACTGGCACTGAAGTGGCATCCAGATAAAAATCCTGAGAATAAAGAAGAAGCA
GAGAGAAAATTCAAGCAAGTAGCGGAGGCATATGAAGTGCTGTCGGATGCTAAGAAACGGGACATCTATG
ACAAATATGGCAAAGAAGGATTAAATGGTGGAGGAGGAGGTGGAAGTCATTTTGACAGTCCATTTGAATT
TGGCTTCACATTCCGTAACCCAGATGATGTCTTCAGGGAATTTTTTGGTGGAAGGGACCCATTTTCATTT
GACTTCTTTGAAGACCCTTTTGAGGACTTCTTTGGGAATCGAAGGGGTCCCCGAGGAAGCAGAAGCCGAG
GGACGGGGTCGTTTTTCTCTGCGTTCAGTGGATTTCCGTCTTTTGGAAGTGGATTTTCTTCTTTTGATAC
AGGATTTACTTCATTTGGGTCACTAGGTCACGGGGGCCTCACTTCATTCTCTTCCACGTCATTTGGTGGT
AGTGGCATGGGCA**ACTTCAAATCGATAT**CAACTTCAACTAAAATGGTTAATGGCAGAAAAATCACTACAA
AGAGAATTGTCGAGAACGGTCAAGAAAGAGTAGAAGTTGAAGAAGATGGCCAGTTAAAGTCCTTAACAAT
AAATGGTGTGGCCGACGACGATGCCCTCGCTGAGGAGCGCATGCGGAGAGGCCAGAACGCCCTGCCAGCC
CAGCCTGCCGGCCTCCGCCCGCCGAAGCCGCCCCGGCCTGCCTCGCTGCTGAGACACGCGCCTCACTGTC
TCTCTGAGGAGGAGGGCGAGCAGGACCGACCTCGGGCACCCGGGCCCTGGGACCCCCTCGCGTCCGCAGC
AGGATTGAAAGAAGGTGGCAAGAGGAAGAAGCAGAAGCAGAGAGAGGAGTCGAAGAAGAAGAAGTCGACC
AAAGGCAATCACTAGACCGGACTTGAGGCACGCGGTGCACCCCCAGACGCTGGCGCTCCACCGTGCTCGG

CATGCGGTCGTGCACACGCGCTAGGTAGCAGCGTCGGTCAGGACTGTCTCGAGGCCACACTCGCTCGGCA
GGATTATGCGATCACGGATCAGTCAGAGCAGGGTCAGGAGACGGGGCTGACGGCACGGGTGGCGGGGACA
GACGTTTGGGACTTGGCCGCGACTCTCTGCTTCTCTCCAGCTCTCAATCTGCTGCATTTTCCTCTAGTGC
TTCCGGATCCTCTTCATTCTTTTCGGCTACTCAACCACTCCGCATGCTGCTGGAATATTTCTGGCTTTAG
AAGTACAGGAGGGCGCAGATGGCTAACTGAGTAACATTCATGAAATGAGGCTTTCTGTGGCGGCGTAGTG
TTTGGAATTAGAAGGTAATTCAGTAGAGTGTAACTTAGAGAATATTGCAAGTGACACATTGAATCCTGCC
CGTCAGGGCACCTTTTCCTCAGAGCAATCCGGCCACACGAATAGAAGGCTGTCGTGAATCACATCAGATG
TAAAATCATTCCTTCTGTTTACTCTTTTAATTTTCATCCTTTGCAGGTAGTGCAAATTCAACTTCAAATA
TGGTGTAGGTTTTGCTAGATTCCATATTTTTTTCTTGGATTTTTGCTAATTATTTTTAGCAAAAAATTTT
TGCTCAGTGGCACTCTCCCTAGTGTCCATGGGTTAGGGCCATGCTGGGGAAAACGGGCCGGTATTTACAC
ACGCGCAAAACACCCAGAGACGGCACAAGGAGGTTGAACTCATGTTTCAGTTCGCGAACATTGACTCCTT
ACGAAAGTCACTTCATTCTAACTAGATGCGCCCACTTCCGGTCATTATTTCGTTTGCATGATGTATTGCT
TCTTCACGTTTTGTTTTTATTGAGCACGGAGTAGAATTCCAGGGCTGCCTTGACTTCTTCCCTGCATGCT
CCCTCCCAGTGACTTTCCTTCCCTTTCACATGAGGATCTGCCGTTCATGTTGCTTTCTCCTTTGTCCTCT
TGGACTTGAGGGCATTGTGAAAAGCTTTGCTGTGATTTAAAAATGCCAGCAATTTTAATCTAGCAGTGTT
GAAGCTGGGAATTTTTTGGCGCAATCCATGTAGCAGTGACCCAGGCTTGGGAGCCAGAAACAAGTGTGAC
CTGGGATTTTATTTAACACAACTGTTGCCAAAGAGTTGGCTTTGTTTATTTGGTTTTGGCGGGGAGAGGA
GTGGTATTTGATGCTTTCTGTGGACAATGTAACCCTAAACACAT**CATGTATTTTAAATGCCACCT**ACATA
AATAAAACATAAGCATATTGAATACAAAAAAAAAAAAAAAAAAA

**4) SYNCRIP, synaptotagmin binding, cytoplasmic RNA interacting protein**

ucaggaaaauuauuau mooring sequence could not be located in NM_019796 mouse transcript

NM_019796 3058 ttgaagtttttcataaa**tcatagaaattagtat**atggctgtagtttagc 3107 (Mm)
||||||||||||||||||||||||||||||||||||||||||||||||||
NM_001159677 3240 ttgaagtttttcataaa**tcatagaaattagtat**atggctgtagtttagc 3289 (Hs)

>gi|228008399|ref|NM_001159677.1| Homo sapiens synaptotagmin binding, cytoplasmic RNA interacting protein (SYNCRIP), transcript variant 6, mRNA
AGCGCGGGAGAGAGAAAGAGAGGAGCCGACTCGGCAGGGACTGGGGGACCGGGCCGAGAGTGCGAGCGAG
CGAGGGAGGGAGTGAGGGAGCGTGCGAGCCAGAAGGGGAAAGGCGGCCACTCGTGCCTGAGCGACCGCAG
AGGGGAGTGGGAGCAGTGGGGTAAAGGAGCGGGGGGCGGGAATAAGAAAGGCCGAGAGAAGGCGGACAGA
GGCTAGTGGTGGTGGTGGTGGTAGGGGGAGAAGGAGGAGCTGGAGGAGGGCAGGGGCTGAGGGAGTGAGT
GAAGCGGACGCGCGAGGGAGGGGAGGGAAGGGAAGGGAAGGGAAGGGGGGGTCACGCGGGGGCGCGCGCG
CGCACCGGGAGCGCGCTCGGAGGCGAGTGGAACTGGATCGGGTTTGCTGCCAGCGGCGTGAGCTTCGGCC
GCCATTTTACAACAGCTCCACTCGCGCCGGACACAGGGAGCAGCGAGCACGCGTTTCCCGCAACCCGATA
CCATCGGACAGGATTTCTCCGCCTCAGCCCAACGGGGAGATCTCTGGAAACATGGCTACAGAACATGTTA

ATGGAAATGGTACTGAAGAGCCCATGGATACTACTTCTGCAGTTATCCATTCAGAAAATTTTCAGACATT
GCTTGATGCTGGTTTACCACAGAAAGTTGCTGAAAAACTAGATGAAATTTACGTTGCAGGGCTAGTTGCA
CATAGTGATTTAGATGAAAGAGCTATTGAAGCTTTAAAAGAATTCAATGAAGACGGTGCATTGGCAGTTC
TTCAACAGTTTAAAGACAGTGATCTCTCTCATGTTCAGAACAAAAGTGCCTTTTTATGTGGAGTCATGAA
GACTTACAGGCAGAGAGAAAAACAAGGGACCAAAGTAGCAGATTCTAGTAAAGGACCAGATGAGGCAAAA
ATTAAGGCACTCTTGGAAAGAACAGGCTACACACTTGATGTGACCACTGGACAGAGGAAGTATGGAGGAC
CACCTCCAGATTCCGTTTATTCAGGTCAGCAGCCTTCTGTTGGCACTGAGATATTTGTGGGAAAGATCCC
AAGAGATCTATTTGAGGATGAACTTGTTCCATTATTTGAGAAAGCTGGACCTATATGGGATCTTCGTCTA
ATGATGGATCCACTCACTGGTCTCAATAGAGGTTATGCGTTTGTCACTTTTTGTACAAAAGAAGCAGCTC
AGGAGGCTGTTAAACTGTATAATAATCATGAAATTCGTTCTGGAAAACATATTGGTGTCTGCATCTCAGT
TGCCAACAATAGGCTTTTTGTGGGCTCTATTCCTAAGAGTAAAACCAAGGAACAGATTCTTGAAGAATTT
AGCAAAGTAACAGAGGGTCTTACAGACGTCATTTTATACCACCAACCGGATGACAAGAAAAAAAACAGAG
GCTTTTGCTTTCTTGAATATGAAGATCACAAAACAGCTGCCCAGGCAAGGCGTAGGTTAATGAGTGGTAA
AGTCAAGGTCTGGGGGAATGTTGGAACTGTTGAATGGGCTGATCCTATAGAAGATCCTGATCCTGAGGTT
ATGGCAAAGGTAAAAGTGCTGTTTGTACGCAACCTTGCCAATACTGTAACAGAAGAGATTTTAGAAAAGG
CATTTAGTCAGTTTGGGAAACTGGAACGAGTGAAGAAGTTAAAAGATTATGCGTTCATTCATTTTGATGA
GCGAGATGGTGCTGTCAAGGCTATGGAAGAAATGAATGGCAAAGACTTGGAGGGAGAAAATATTGAAATT
GTTTTTGCCAAGCCACCAGATCAGAAAAGGAAAGAAAGAAAAGCTCAGAGGCAAGCAGCAAAAAATCAAA
TGTATGACGATTACTACTATTATGGTCCACCTCATATGCCCCCTCCAACAAGAGGTCGAGGGCGTGGAGG
TAGAGGTGGTTATGGATATCCTCCAGATTATTATGGATATGAAGATTATTATGATTATTATGGTTATGAT
TACCATAACTATCGTGGTGGATATGAAGATCCATACTATGGTTATGAAGATTTTCAAGTTGGAGCTAGAG
GAAGGGGTGGTAGAGGAGCAAGGGGTGCTGCTCCATCCAGAGGTCGTGGGGCTGCTCCTCCCCGCGGTAG
AGCCGGTTATTCACAGAGAGGAGGTCCTGGATCAGCAAGAGGCGTTCGAGGTGCGAGAGGAGGTGCCCAA
CAACAAAGAGGCCGCGGGCAGGGAAAAGGGGTCGAGGCCGGTCCTGACCTGTTACAATGAAGACTGACTT
GCTATGTGGGATTACACCAGAAGCTTGCAGTGGAGTAATGGTAAGGAAATCAAGCAACCTTAAATATGTC
GGCTGTATAGGAGCATATTCTATTGCAGAAGACCTTCCTATGAAGATCATGGAATCAAATACGGGACATT
GAACTAATACTTGGACTTTGATATGAATTTCTTTAACAATTTTCTCTGCAGTGCAAGTTATTAAACTAAA
GCTACTCTATTTTCAAAATGTGTTCCAACAGAAATCCTTCATAACTCCTAGCATGGTATCTTAATAAAGA
ATAAAGTTCTTTTAAAAATCTGCTCTAAGTAGATTTTTCCCCTTTTTTAAATTAAGGATCCCAACAGTGG
TATTTTGAAATATTCTCTTGAATTTGTGCATTTAAATTTTATTGCAGTGGTATAGATGAATGCCACTGAT
GGTATCCTTAAATTTTATTTCTGCTCACCAAGGTTAATCATGATTGTCTATATCTTTTTTATAGTGATCA
CTTTTGAATTGTGTTCAGATATGCAGTTTCAGGTGTAATCATCAGAGCTGGTTAGTCAGGCATTCCAGAT
AGTGGTTCTTTTCAGAACCTTTTTAAAAGGGTTGGTTAACTACCTCAGTAGCAGAGGATTGAACTATACC
CTGTCTGTACTGTACATAGAAAATCTTTGTAGATAAAAGCAAGGCTTGTTAAATATGATATGAGGGTAAG
ATTTTAATATACCAAATGTAACATTCTTAGTTGCCTTTAGTTTCAGAGGCTTGTAAGACTTCCTCATGAC
CATCATAACAGGCCTTGCTTTTGTCGTATTTTGTGGCTGAAAAAGCAGCCTTGCTTCTTCAGATATTGTA
GTTATTTGGATGTATAATAGTTTAGCAAGATGTTACTTTTGTAAGACATCAGATGTTCAAAAAAGTGCAT
CCGAACTTGTACTAAATACTGCAGTGTCCCTTTATAAAAAGTCAGACTAAAACTGACAATTGTACAGCGA

AGCCTGACATTTGGATATTTTGAAGTTTTTTCATAAA**TCATAGAAATTAGTAT**ATGGCTGTAGTTTAGCT
TTTTAGGTAAAAGGTATGTTTCATTAGTGCATTTCTTCCTGCTGATCACTGTAAACATGTGAATCAGCTT
TCCATTTCTTATGCAGGTCATGATAACTTGTAGAGTAGAGTACAATCATTTGTGCTATGTTTTTAATTTT
CTAAAGCACCTTGATGACAGTGAGTGTCCAGTGGTGAAGCATCCTCTATTGAACCACCCTCAAAAATTTT
TTTGCCAAGTCCTAAGTTGATAGCTTAAAGTAAAAAGTGAAAATTATAGTTTCATTAGGACTTGGTGTAA
AGAAATCCCCTCCCCCCTTCCCCAAAGGGATACTGCAGTTATATCACATACCCAATAGGCACCACGATGA
AGATCAGAGCTTATACTTAATTAAGGTTTTATACACACCAGTTCCCCAGTAAATGCAAATTTAACAAGAA
AATCAGACATGTCATATGTTCAAAATGCTCATG**GCAAACAATCATTTTGC**ATTCCTGCAAATAAAATTGT
TTTATACTGTAAGCTGGAGGCGAGTGTAACTTATTTTTGTAATAAAGTTTTTATTTTTTTTATGTGTCAT
TAATATAAATGTGTGTTAGTGTAGAAATCTTCTGGTTTAAAAACTTAGAATTGCACACATTTCAGTATGT
TTATTTGTACTTACATAATTTTAGAATAGTGGTTGCCAATAGCCTGTATGTTTCACATTAATTGGTTTTT
TGTTATCTAAATAAATCATTTTAGTATGTTGTATGTCAGTTACTGGGATAGCTGGGACATAGAGTGTAAT
TTAAAATTTGTCAATAAGTATTCATTGGAATATATGTAAATGTGCCTTGCCGGTTATTGAAACTTATCTA
CAAAATGAGTATGGGGTGACAAAAATTAGTTCCTGGTGCTTAATGAAACTTTCTGCCACTGATTTTATAT
ATTACCCCGTGCTTTTTTAAAGTACATCTCTCTCAAAACTTAGTGTAAGTTTGAGGGCTACACAAAACAT
TTACATTTCATTCTAACATAATGAATATAATAGGTTGTGGAAAGTGGGTAAACTAAATGTAGCCTTCAGT
AAAATTGAATCTCAGTGTAATCCTTGGTGCTGGCATTTCTCAGTTCCGAGGAGTTAAATGATCCCATCTA
AGAGGTCATTGCCATGCCTATTGGCACTTTACTGTCATAGCATTTTTAAGGGACACTGTCAAGGTGTTTA
AGTTCTCAGAATTACTTGTTGGGATTTTAGGACAGGTTTGTTTACTTAAAGTAAGAACTGCATTGTCAAA
GTTGAAAGAGGAACACTTTTGTGAGTTCACAAATGTGTTCTTAAGAAAACATTAAAATATGGAGCTCTGG
GTTTTCAAGACTATTTGGCATTCTTAATTTGGGGACTTGGGAGGGAAACTGATAAAAAGAAATTGAAGAA
TTGATGGTTATACTTAAAGAAGGGTAATGTAAACAGTGGTGATGAAATATATACACATCAAGTGAAATTA
CTTGACAGTGTTCATTTGAATGACTTTGAATTCAAGCCATTATAATTACTTTTAAAATTAAATATCATTT
GCACTGTTCTGATAATGGGTGCAGTTTTTGAGCAATATAATCAGAGCTAAATATGCATGTAGTGATTAGT
GATGTGAACAATTAACGTTCTGAGAAGAAATACTAACTGTGGTATTTTCAAACTTAAATTTCTGTAGTAA
AATCAGTATCAAAGTCTTATCAGATCAAGGAAAAACAGGCAATGCATATAAACATACTTTTGAATGTTGT
GTGGCCTATAAAGCAATAATGCAATTTATATGGAATGTCATGGGATATGAGAAATGGAAATGCAAAAATA
ACTAATCCTTTAGTAAAAATGTCAACATGTTAAAGGGGGAATGTTAACTAATGTAGGTTATTGCTATTTG
TGATTTGTTTATGGGTTCTTGGCTTTGACAGCTTCAAAGAATGGACAGTGATAAGTTAAAAGAAATTTTG
TATATTGTCAAGGAAAGGGTCTTAAATCCGAGTCAAGTCCCTTCCTTGGGGTAAAAAATGTATTCTTAAA
GCATTCTGATGTTAAAAAGAAAACTTAAGTTATCTAACCAAAACAGACGCAAGATTTTGTTTCTGCAGAC
TACTTGGCAATCAAAAGTGATCATAAATTTAGGTTATCAGTTTTCAGAAAGTTGCTTTGTGAGAAAATTT
TGTTAGATATATTCTCCCAAGCATGCTTTTTGTGGAAGGTTTTCAGCCATTGCCACTGAATCAGATGTTA
AAAATGAAGGGAAAATTGAGTGTGCACACACACAACTGTTGTACACTCATGATTGCAGTTTTTAGCTTAA
GAAACTTTTCTACCAGTTACTGTGAATCTGACTTAAAATGTAAAGTTTCCTCATGATAAAATAGGAACAA
CATAGAAATGGATTGATGGGGTGATCTGAGTTATTGTATATAAAAGTTTTTAAAGAATAGAATGAACATC
AAGCTAGATAGGCAAAAATTGACACATTCAGAACAGCTTTTTTGACTGCGAAGCCAAAAGTTGTCAGAAA
CAGCAAAAGATCCCTTATTATTACAGAGTATTTTACGTAGTCTCTATTTTAAGGAGAGAAATTAAATAGA

AGGGCTTCATGCATTTAGGGGAGGGTGCTAAAACTTCTCAAGTTCGTCAAACTTACAGGAATACCCACCA
TGATCATTTTCTCTCTAATTATGTATACCACAAAATTTTCATCTGGCCATAGGAATTCACTGGTGGGTGT
AAAATTAATGACTAAAGAAATTAAGTGACAAATACATAAAAGAAACAGACTTGTGGGGATATTGTTTTAA
GGTGTATTAATTACTCAGTGATGATACCACTCAATAGGGCATGCCACTACTTTTCTTAAGATGCTAATTA
TGAAGCAGTGCTCACAGGCATTTTTTAACTAGCAAATTAGTAGATGGACTTTTGGGGTCTGTCACTTTTT
AAAAGTATTTAAGACTTAAATTCTATTAGCACCACAGTCTGCCTTCAGTAATACACCTAAAATATTTTTC
AGGACCAGAAGCATTCAGTTTGAAAATTTGCAGATGCAAACCAGTATTATTACTAACGCTCTGGGTCAAA
GATTAGGTTTTTAATATTAACAGTAGTCTGGTAAATATTTAGAAGTCTGGCATTGAGAAACAAAAGCTTG
TACCTGACTAGTATTTTTATTTAAAAAAATTAGTTCTGTTAGCTTATTTAAATTGTGTTTTATTTATCCG
TAGAATTTATATTTATTTCATTCCTTTCATCTCACTGAAAACTGTCTGCAGGCCCTTTGATTTGGATTAG
ATGTGTGAAGTACTGTCTTTTGCCAAAAACCTCAAATTACCTGTTCTTTTCAACGTAGTGGGTTTGTGCT
TGTTTGGAGATCAGTTCAAAAACTATCTGTACTATCTGTACTGCCTCTGATGTTAAGATTTTATGTATAG
CATAAGGAAGCTAGCTCTGACTATATTTTCCTAAGAATAAAGACCTATTTTTGTAGCATGTCTTAGGATC
TCCAGGAGTCCAAGAATTATTGTGGGTGTCCTCCAATTCATCACTCTTCACTTAACAGCTTTTAAGTAGA
CACTTGGAATCTTTAGAGGTCTGTCGCCCTTTGATTATCCATACATTCGAAGTAACTAGCCAATGGTGAA
AAATTCCTCAAGATATCCTCAGTTGCAATCACATTACTGGAAGATGAATAGAATAAATGTATTAGGCTGG
TCTTAATTTTTGATGGAAATATTCTGTTGTCCCGTACTTGCCATTGGATTTGATAAAGTTAGTGGTAATT
TGGAAAGAATCGGGGACTTGCCAATATATTTGTGGGTTTTAGCTTATACCCCTAGGATTTCTTGGTTGCG
GGACGAGCAGTTTTGGCCACTTCCATCAGGACAAGACTTTTTAGGTCACTTAGTGCAGGTTTTAGTTTCT
ATTTTGGATTAACAACATTTATATTGATTATCGAAAAGAAGCTTTCATCATTTCAGAACAGTCCTGGAAG
TTTGACTTTGAGTGTGGGAGAAGTCCTAATAAACCATTTTGGAAATTAAAAAAAAAA

### 5) NGFRAP1, nerve growth factor receptor (TNFRSF16) associated protein 1

```
NM_001110234      786 ccattagcatggaaagcaatgtcaagtcaataaagcaactgaataatcat    835   (Mm)
                      |||||.|||||||||  |||.|.|||..||||||||||.|.||
NM_206917         870 ccatttgcatggaaa--aatttaaagctaataaagcaatttaa-------    910   (Hs)

NM_001110234      836 tattgtgtccttaaagcataaataaacagttaagattaaaaaaaa    880   (Mm)
                                  ||||||.|||.|||           ||||||||
NM_206917         911 ------------aaagcaaaaaaaaa-----------aaaaaaaa    932   (Hs)
```

Mooring sequence is not conserved

>gi|46094061|ref|NM_206917.1| Homo sapiens nerve growth factor receptor (TNFRSF16) associated protein 1 (NGFRAP1), transcript variant 1, mRNA
GGCTGTGCGCTCTGGGGCACCTCATGCAGACGGGAGTAGGGAGGCTGTTTTTTGCGCGTGCATATGGCGG
TGGCGGGTGGGGGGAAGGGGGAGATCCTGCTGCACTGGCCGCCCAAGTTGGGGGGCGAGCTCGGTGGTGA

```
CGCGCGGCCCTCACGTGACCCAGAGCTGCAGAGCGACGCAGCCTTCGGTGCAGTCGTCACTCGCGTCTGG
CTACCAGCTCCCCGCTGCCCTGAGCTCGGCGGGCTGGCATTCGGCCCGGGGAAAAGCGGAGCAGGTCTGC
GAGGCTAAGTGTCTCCGCGGCGCACCTCGCGGCGAGAATCCGGAGGAGAAGGAGACTGCAAGGATAGGCC
CAGGAAAACGAAGAGATGGAGCAGCCTATGCAGAATGGAGAGGAAGACCGCCCTTTGGGAGGAGGTGAAG
GCCACCAGCCTGCAGGAAATCGACGGGGACAGGCTCGCCGACTTGCCCCTAATTTTCGATGGGCCATACC
CAATAGGCAGATCAATGATGGGATGGGTGGAGATGGAGATGATATGGAAATATTCATGGAGGAGATGAGA
GAAATCAGAAGAAAACTTAGGGAGCTGCAGTTGAGGAATTGTCTGCGTATCCTTATGGGGGAGCTCTCTA
ATCACCATGACCATCATGATGAATTTTGCCTTATGCCTTGACTCCTGCCATTTATCATGAGATTAATACT
GTGATTCCCGCTGTTTTCTTTTTCCTTGCATTTTCCTAATATGCCTTTACTGATCCGTTTGCTGTGAACC
CTATGTTATTTCCATGTGTCAAGTGGGTCTTGTGTTGCCAGCTTCTATTTGAAGATTGCCTTTGCACTCA
GTGTAAGTTTCTGTCAGCAGTAGTTTCACCCATTTGCATGGAAAAATTTAAAGCTAATAAAGCAATTTAA
AAAGCAAAAAAAAAAAAAAA
```

## Supplementary information for figure 3b

APOBEC1 RNA-editing targets (predicted or experimentally validated by Rosenberg B.R. *et al*, 2011) x L-SAGE_17_RT_HgtC_FDR
=> 1 transcript in common between both lists, namely 567 (NCBI Gene ID)

### Orthology table:

| **Index** | **APOBEC1 RNA-editing target(Mouse)** | **Gene Symbol** | **Description** | **Human orthologue** (blastn first hit) | **Blast Score** | **E value** | **Entrez Gene ID (human)** |
|---|---|---|---|---|---|---|---|
| 1 | NM_009735 | B2M | beta-2-microglobulin | NM_004048 | 205 | 9e -50 | 567 |

### RNA-editing table:

| **Index** | **APOBEC1 RNA-editing target(Mouse)** | **RNA editing status** | **Mouse mooring sequence in transcript** | **Human orthologue** | **Human mooring sequence** (By similarity) | **17 base *NlaIII* tag (RT) In human transcript** | **17 base *NlaIII* tag edition by APOBEC1** |
|---|---|---|---|---|---|---|---|
| 1 | NM_009735 | exp. validated | ucauuacuu | NM_004048 | Not conserved | **TGTACTTCCTAAGGTGA** | Unlikely, because the mooring sequence is not conserved between mouse and human |

1) **B2M, Homo sapiens beta-2-microglobulin (B2M)**

```
NM_009735        657 tcata-------tgccaa-----accctctg-------------------    675   (Mm)
                     .||||       |.||||     |..|||||
NM_004048        759 gcataagttaacttccaatttacatactctgcttagaatttgggggaaaa    808   (Hs)


NM_009735        676 ----------tacttctc---attacttggatgca----gttactcat--    706   (Mm)
                               ||.||..|   |||| |||||   |    |||| |.||
NM_004048        809 tttagaaatataattgacaggatta-ttgga---aatttgtta-taatga    853   (Hs)
```

The mooring sequence is not conserved

>gi|37704380|ref|NM_004048.2| Homo sapiens beta-2-microglobulin (B2M), mRNA
AATATAAGTGGAGGCGTCGCGCTGGCGGGCATTCCTGAAGCTGACAGCATTCGGGCCGAGATGTCTCGCT
CCGTGGCCTTAGCTGTGCTCGCGCTACTCTCTCTTTCTGGCCTGGAGGCTATCCAGCGTACTCCAAAGAT
TCAGGTTTACTCACGTCATCCAGCAGAGAATGGAAAGTCAAATTTCCTGAATTGCTATGTGTCTGGGTTT
CATCCATCCGACATTGAAGTTGACTTACTGAAGAATGGAGAGAGAATTGAAAAAGTGGAGCATTCAGACT
TGTCTTTCAGCAAGGACTGGTCTTTCTATCTCTTGTACTACACTGAATTCACCCCCACTGAAAAAGATGA
GTATGCCTGCCGTGTGAACCATGTGACTTTGTCACAGCCCAAGATAGTTAAGTGGGATCGAGACATGTAA
GCAGCATCATGGAGGTTTGAAGATGCCGCATTTGGATTGGATGAATTCCAAATTCTGCTTGCTTGCTTTT
TAATATTGATATGCTTATACACTTACACTTTATGCACAAAATGTAGGGTTATAATAATGTTAACATGGAC
ATGATCTTCTTTATAATTCTACTTTGAGTGCTGTCTCCATGTTTGATGTATCTGAGCAGGTTGCTCCACA
GGTAGCTCTAGGAGGGCTGGCAACTTAGAGGTGGGGAGCAGAGAATTCTCTTATCCAACATCAACATCTT
GGTCAGATTTGAACTCTTCAATCTCTTGCACTCAAAGCTTGTTAAGATAGTTAAGCGTGCATAAGTTAAC
TTCCAATTTACATACTCTGCTTAGAATTTGGGGGAAAATTTAGAAATATAATTGACAGGATTATTGGAAA
TTTGTTATAATGAATGAAACATTTTGTCATATAAGATTCATATTTACTTCTTATACATTTGATAAAGTAA
GG**CATGGTTGTGGTTAATCTGGT**TTATTTTTGTTCCACAAGTTAAATAAATCATAAAACTTGATGTGTTA
TCTCTTA
