# Supplementary material for: Increased frequency of single base substitutions in a population of transcripts expressed in cancer cells
Source: BMC Cancer. 2012 Nov 8;12:509. doi: 10.1186/1471-2407-12-509 (PMC3522053; doi:10.1186/1471-2407-12-509)
Supplement: Additional file 9 — Manual checking of possible APOBEC1 RNA-editing targets altering RT with greater SBS frequencies in cancer than in healthy cells (Tag-seq). [file 1471-2407-12-509-S9.pdf]

# Additional_result_file_4 to support figure 3c

APOBEC1 RNA-editing targets (predicted or experimentally validated by Rosenberg B.R. *et al*, 2011) x Tag-seq_4289_RT_CgtH_FDR
=> 53 transcripts in common between both lists (NCBI Gene IDs)

351, 567, 648, 987, 1386, 1810, 1997, 2313, 3615, 4942,
5218, 5358, 5567, 5796, 5925, 6102, 6319, 6428, 6601, 8539,
8924, 9191, 9373, 9403, 9510, 9730, 9741, 9849, 9989, 10001
10009, 22861, 22903, 23132, 23142, 23272, 23635, 26136, 27018, 29761
51742, 54776, 54918, 55754, 57120, 57132, 57515, 57799, 80218, 84162
116254, 133746, 552889 (underlined Gene ID represent experimentally validated APOBEC1 editing targets)

NB: Alteration of the NlaIII tag by APOBEC1 was checked only for experimentally validated APOBEC1 editing targets

| Index | APOBEC1 RNA-editing target(Mouse) | Gene Symbol | Description | Human orthologue (blastn first hit) | Blast Score | E value | Entrez Gene ID (human) |
|---|---|---|---|---|---|---|---|
| 1 | NM_007471 | APP | amyloid beta (A4) precursor protein | NM_201414 | 3646 | 0.0 | 351 |
| 2 | NM_009735 | B2M | beta-2-microglobulin | NM_004048 | 205 | 9e -50 | 567 |
| 3 | NM_001025093 | ATF2 | activating transcription factor 2 | NM_001880 | 2678 | 0.0 | 1386 |
| 4 | NM_009029 | RB1 | retinoblastoma-associated protein | NM_000321 | 3439 | 0.0 | 5925 |
| 5 | NM_053102 | SEP15 | 15 kDa selenoprotein precursor | NM_004261 | 797 | 0.0 | 9403 |
| 6 | NM_013918 | USP25 | ubiquitin carboxyl-terminal hydrolase 25 | NM_013396 | 4353 | 0.0 | 29761 |
| 7 | NM_133718 | TMEM30A | transmembrane protein 30A | NM_018247 | 1797 | 0.0 | 55754 |
| 8 | NM_019760 | SERINC1 | serine incorporator 1 precursor | NM_020755 | 1849 | 0.0 | 57515 |

RNA-editing table:

| **Index** | **APOBEC1 RNA-editing target(Mouse)** | **RNA editing status** | **Mouse mooring sequence in transcript** | **Human orthologue** | **Human mooring sequence** (By similarity) | **17 base *NlaIII* tag (RT) In human transcript** | **17 base *NlaIII* tag edition by APOBEC1** |
|---|---|---|---|---|---|---|---|
| 1 | NM_007471 | exp. validated | ugaucacuau gaaucauugc | NM_201414 | tgatcactat conserved<br><br>gaatcattgc conserved | ATCGCTTTCTACACTGT | No, because NlaIII tag is 3' to the mooring sequence |
| 2 | NM_009735 | exp. validated | ucauuacuu | NM_004048 | Not conserved | TGTACTTCCTAAGGTGA | Unlikely, because the mooring sequence is not conserved between mouse and human |
| 3 | NM_001025093 | exp. validated | aaauuacau | NM_001880 | Not conserved | AGTAAACCATCAGAGAA | Unlikely, because the mooring sequence is not conserved between mouse and human |
| 4 | NM_009029 | exp. validated | aaauugcuau | NM_000321 | aaattgctat conserved | AATATCATACAAATCAG | No, because NlaIII tag is 3' to the mooring sequence |
| 5 | NM_053102 | exp. validated | gcaugcuuac | NM_004261 | gcttgcttcc conserved | TGCTGTGTGCTCTTTGA | No, because NlaIII tag is 3' to the mooring sequence |
| 6 | NM_013918 | exp. validated | ugauuauuu | NM_013396 | tgattattt | ACTACAGCCATTTAAGT | No, because NlaIII tag is too distant from the mooring sequence |
| 7 | NM_133718 | exp. validated | agauuaauau | NM_018247 | tgattaatat | CACCATTCAGTAAACAT | No, because NlaIII tag is 3' to the mooring sequence |
| 8 | NM_019760 | exp. validated | acuagaaaauuaguau | NM_020755 | Not conserved | TAATTGAAATATATAGA | Unlikely, because the mooring sequence is not conserved between mouse and human |

# Legend:

Red nucleotides = mouse mooring sequence
Violet nucleotides = human mooring sequence (by similarity)
Green nucleotides = 17 base *NlaIII* tag in human transcript
Blue nucleotides = *NlaIII* "CATG" recognition site
Underlined nucleotides = Stretch of bases in which C to U edition could occur (3' to the mooring sequence)

## 1) APP, amyloid beta (A4) precursor protein

```
NM_007471       2690 --cctacgtattcttttcctgatcactatgcatt------ttgaacattt   2731   (Mm)
                       ||||.||||||.||||||||||||||||||||      ||.||||  |
NM_201414       2721 tgcctaagtattcctttcctgatcactatgcattttaaagttaaaca--t   2768   (Hs)


NM_007471       3020 ttctccaaaaattaattttctgcaggatgattgtacagaatcattgctta   3069   (Mm)
                     ||||||||||  .|||||||||||||||||||||||||||||||||||||
NM_201414       3054 ttctccaaaa--caattttctgcaggatgattgtacagaatcattgctta   3101   (Hs)
```

```
>gi|228008405|ref|NM_201414.2| Homo sapiens amyloid beta (A4) precursor protein (APP), transcript variant
3, mRNA
GGATCAGCTGACTCGCCTGGCTCTGAGCCCCGCCGCCGCGCTCGGGCTCCGTCAGTTTCCTCGGCAGCGG
TAGGCGAGAGCACGCGGAGGAGCGTGCGCGGGGGCCCCGGGAGACGGCGGCGGTGGCGGCGCGGGCAGAG
CAAGGACGCGGCGGATCCCACTCGCACAGCAGCGCACTCGGTGCCCCGCGCAGGGTCGCGATGCTGCCCG
GTTTGGCACTGCTCCTGCTGGCCGCCTGGACGGCTCGGGCGCTGGAGGTACCCACTGATGGTAATGCTGG
CCTGCTGGCTGAACCCCAGATTGCCATGTTCTGTGGCAGACTGAACATGCACATGAATGTCCAGAATGGG
AAGTGGGATTCAGATCCATCAGGGACCAAAACCTGCATTGATACCAAGGAAGGCATCCTGCAGTATTGCC
AAGAAGTCTACCCTGAACTGCAGATCACCAATGTGGTAGAAGCCAACCAACCAGTGACCATCCAGAACTG
GTGCAAGCGGGGCCGCAAGCAGTGCAAGACCCATCCCCACTTTGTGATTCCCTACCGCTGCTTAGTTGGT
GAGTTTGTAAGTGATGCCCTTCTCGTTCCTGACAAGTGCAAATTCTTACACCAGGAGAGGATGGATGTTT
GCGAAACTCATCTTCACTGGCACACCGTCGCCAAAGAGACATGCAGTGAGAAGAGTACCAACTTGCATGA
CTACGGCATGTTGCTGCCCTGCGGAATTGACAAGTTCCGAGGGGTAGAGTTTGTGTGTTGCCCACTGGCT
GAAGAAAGTGACAATGTGGATTCTGCTGATGCGGAGGAGGATGACTCGGATGTCTGGTGGGGCGGAGCAG
ACACAGACTATGCAGATGGGAGTGAAGACAAAGTAGTAGAAGTAGCAGAGGAGGAAGAAGTGGCTGAGGT
GGAAGAAGAAGAAGCCGATGATGACGAGGACGATGAGGATGGTGATGAGGTAGAGGAAGAGGCTGAGGAA
CCCTACGAAGAAGCCACAGAGAGAACCACCAGCATTGCCACCACCACCACCACCACCACAGAGTCTGTGG
AAGAGGTGGTTCGAGTTCCTACAACAGCAGCCAGTACCCCTGATGCCGTTGACAAGTATCTCGAGACACC
TGGGGATGAGAATGAACATGCCCATTTCCAGAAAGCCAAAGAGAGGCTTGAGGCCAAGCACCGAGAGAGA
ATGTCCCAGGTCATGAGAGAATGGGAAGAGGCAGAACGTCAAGCAAAGAACTTGCCTAAAGCTGATAAGA
AGGCAGTTATCCAGCATTTCCAGGAGAAAGTGGAATCTTTGGAACAGGAAGCAGCCAACGAGAGACAGCA
GCTGGTGGAGACACACATGGCCAGAGTGGAAGCCATGCTCAATGACCGCCGCCGCCTGGCCCTGGAGAAC
TACATCACCGCTCTGCAGGCTGTTCCTCCTCGGCCTCGTCACGTGTTCAATATGCTAAAGAAGTATGTCC
GCGCAGAACAGAAGGACAGACAGCACACCCTAAAGCATTTCGAGCATGTGCGCATGGTGGATCCCAAGAA
AGCCGCTCAGATCCGGTCCCAGGTTATGACACACCTCCGTGTGATTTATGAGCGCATGAATCAGTCTCTC
TCCCTGCTCTACAACGTGCCTGCAGTGGCCGAGGAGATTCAGGATGAAGTTGATGAGCTGCTTCAGAAAG
AGCAAAACTATTCAGATGACGTCTTGGCCAACATGATTAGTGAACCAAGGATCAGTTACGGAAACGATGC
```

TCTCATGCCATCTTTGACCGAAACGAAAACCACCGTGGAGCTCCTTCCCGTGAATGGAGAGTTCAGCCTG
GACGATCTCCAGCCGTGGCATTCTTTTGGGGCTGACTCTGTGCCAGCCAACACAGAAAACGAAGTTGAGC
CTGTTGATGCCCGCCCTGCTGCCGACCGAGGACTGACCACTCGACCAGGTTCTGGGTTGACAAATATCAA
GACGGAGGAGATCTCTGAAGTGAAGATGGATGCAGAATTCCGACATGACTCAGGATATGAAGTTCATCAT
CAAAAATTGGTGTTCTTTGCAGAAGATGTGGGTTCAAACAAAGGTGCAATCATTGGACTCATGGTGGGCG
GTGTTGTCATAGCGACAGTGATCGTCATCACCTTGGTGATGCTGAAGAAGAAACAGTACACATCCATTCA
TCATGGTGTGGTGGAGGTTGACGCCGCTGTCACCCCAGAGGAGCGCCACCTGTCCAAGATGCAGCAGAAC
GGCTACGAAAATCCAACCTACAAGTTCTTTGAGCAGATGCAGAACTAGACCCCCGCCACAGCAGCCTCTG
AAGTTGGACAGCAAAACCATTGCTTCACTACCCATCGGTGTCCATTTATAGAATAATGTGGGAAGAAACA
AACCCGTTTTATGATTTACTCATTATCGCCTTTTGACAGCTGTGCTGTAACACAAGTAGATGCCTGAACT
TGAATTAATCCACACATCAGTAATGTATTCTATCTCTCTTTACATTTTGGTCTCTATACTACATTATTAA
TGGGTTTTGTGTACTGTAAAGAATTTAGCTGTATCAAACTAGTGCATGAATAGATTCTCTCCTGATTATT
TATCACATAGCCCCTTAGCCAGTTGTATATTATTCTTGTGGTTTGTGACCCAATTAAGTCCTACTTTACA
TATGCTTTAAGAATCGATGGGGGATGCTTCATGTGAACGTGGGAGTTCAGCTGCTTCTCTTGCCTAAGTA
TTCCTTTCC**TGATCACTAT**GCATTTTAAAGTTAAACATTTTTAAGTATTTCAGATGCTTTAGAGAGATTT
TTTTTCCATGACTGCATTTTACTGTACAGATTGCTGCTTCTGCTATATTTGTGATATAGGAATTAAGAGG
ATACACACGTTTGTTTCTTCGTGCCTGTTTTATGTGCACACATTAGGCATTGAGACTTCAAGCTTTTCTT
TTTTTGTCCACGTATCTTTGGGTCTTTGATAAAGAAAAGAATCCCTGTTCATTGTAAGCACTTTTACGGG
GCGGGTGGGGAGGGGTGCTCTGCTGGTCTTCAATTACCAAGAATTCTCCAAAACAATTTTCTGCAGGATG
ATTGTACAGAATCATTGCTTATGA**CATGATCGCTTTCTACACTGT**ATTACATAAATAAATTAAATAAAAT
AACCCCGGGCAAGACTTTTCTTTGAAGGATGACTACAGACATTAAATAATCGAAGTAATTTTGGGTGGGG
AGAAGAGGCAGATTCAATTTTCTTTAACCAGTCTGAAGTTTCATTTATGATACAAAAGAAGATGAAAATG
GAAGTGGCAATATAAGGGGATGAGGAAGGCATGCCTGGACAAACCCTTCTTTTAAGATGTGTCTTCAATT
TGTATAAAATGGTGTTTT**CATG**TAAATAAATACATTCTTGGAGGAGCAAAAAAAAAAAAAAAA

## 2) B2M, beta-2-microglobulin

```
NM_009735         657 tcata-------tgccaa-----accctctg------------------    675   (Mm)
                      .||||       |.||||     |..|||||
NM_004048         759 gcataagttaacttccaatttacatactctgcttagaatttggggaaaa    808   (Hs)


NM_009735         676 ----------tacttctc---attacttggatgca----gttactcat--    706   (Mm)
                                ||.||..|   |||| |||||   |    |||| |.||
NM_004048         809 tttagaaatataattgacaggatta-ttgga---aatttgtta-taatga    853   (Hs)
```

The mooring sequence is not conserved

### 3) ATF2, activating transcription factor 2

Mooring sequence is not conserved between human and mouse B2M transcripts (transcripts do not align)

### 1) RB1, retinoblastoma associated protein

```
NM_009029       4533 tgtaccggttgaaactataca-ttcaaattgctatgttcctattttcta-   4580
                     ||||..||||.|||||.|||| ||.|||||||||||||.||||||||||
NM_000321       4674 tgtattggttaaaactgtacatttaaaattgctatgttactattttctac   4723
```

```
>gi|108773786|ref|NM_000321.2| Homo sapiens retinoblastoma 1 (RB1), mRNA
GCTCAGTTGCCGGGCGGGGGAGGGCGCGTCCGGTTTTTCTCAGGGGACGTTGAAATTATTTTTGTAACGG
GAGTCGGGAGAGGACGGGGCGTGCCCCGACGTGCGCGCGCGTCGTCCTCCCCGGCGCTCCTCCACAGCTC
GCTGGCTCCCGCCGCGGAAAGGCGTCATGCCGCCCAAAACCCCCCGAAAAACGGCCGCCACCGCCGCCGC
TGCCGCCGCGGAACCCCCGGCACCGCCGCCGCCGCCCCCTCCTGAGGAGGACCCAGAGCAGGACAGCGGC
CCGGAGGACCTGCCTCTCGTCAGGCTTGAGTTTGAAGAAACAGAAGAACCTGATTTTACTGCATTATGTC
AGAAATTAAAGATACCAGATCATGTCAGAGAGAGAGCTTGGTTAACTTGGGAGAAAGTTTCATCTGTGGA
TGGAGTATTGGGAGGTTATATTCAAAAGAAAAAGGAACTGTGGGGAATCTGTATCTTTATTGCAGCAGTT
GACCTAGATGAGATGTCGTTCACTTTTACTGAGCTACAGAAAAACATAGAAATCAGTGTCCATAAATTCT
TTAACTTACTAAAAGAAATTGATACCAGTACCAAAGTTGATAATGCTATGTCAAGACTGTTGAAGAAGTA
TGATGTATTGTTTGCACTCTTCAGCAAATTGGAAAGGACATGTGAACTTATATATTTGACACAACCCAGC
AGTTCGATATCTACTGAAATAAATTCTGCATTGGTGCTAAAAGTTTCTTGGATCACATTTTTATTAGCTA
AAGGGGAAGTATTACAAATGGAAGATGATCTGGTGATTTCATTTCAGTTAATGCTATGTGTCCTTGACTA
TTTTATTAAACTCTCACCTCCCATGTTGCTCAAAGAACCATATAAAACAGCTGTTATACCCATTAATGGT
TCACCTCGAACACCCAGGCGAGGTCAGAACAGGAGTGCACGGATAGCAAAACAACTAGAAAATGATACAA
GAATTATTGAAGTTCTCTGTAAAGAACATGAATGTAATATAGATGAGGTGAAAAATGTTTATTTCAAAAA
TTTTATACCTTTTATGAATTCTCTTGGACTTGTAACATCTAATGGACTTCCAGAGGTTGAAAATCTTTCT
AAACGATACGAAGAAATTTATCTTAAAAATAAAGATCTAGATGCAAGATTATTTTTGGATCATGATAAAA
CTCTTCAGACTGATTCTATAGACAGTTTTGAAACACAGAGAACACCACGAAAAAGTAACCTTGATGAAGA
GGTGAATGTAATTCCTCCACACACTCCAGTTAGGACTGTTATGAACACTATCCAACAATTAATGATGATT
TTAAATTCAGCAAGTGATCAACCTTCAGAAAATCTGATTTCCTATTTTAACAACTGCACAGTGAATCCAA
AAGAAAGTATACTGAAAAGAGTGAAGGATATAGGATACATCTTTAAAGAGAAATTTGCTAAAGCTGTGGG
ACAGGGTTGTGTCGAAATTGGATCACAGCGATACAAACTTGGAGTTCGCTTGTATTACCGAGTAATGGAA
TCCATGCTTAAATCAGAAGAAGAACGATTATCCATTCAAAATTTTAGCAAACTTCTGAATGACAACATTT
TTCATATGTCTTTATTGGCGTGCGCTCTTGAGGTTGTAATGGCCACATATAGCAGAAGTACATCTCAGAA
TCTTGATTCTGGAACAGATTTGTCTTTCCCATGGATTCTGAATGTGCTTAATTTAAAAGCCTTTGATTTT
TACAAAGTGATCGAAAGTTTTATCAAAGCAGAAGGCAACTTGACAAGAGAAATGATAAAACATTTAGAAC
```

GATGTGAACATCGAATCATGGAATCCCTTGCATGGCTCTCAGATTCACCTTTATTTGATCTTATTAAACA
ATCAAAGGACCGAGAAGGACCAACTGATCACCTTGAATCTGCTTGTCCTCTTAATCTTCCTCTCCAGAAT
AATCACACTGCAGCAGATATGTATCTTTCTCCTGTAAGATCTCCAAAGAAAAAAGGTTCAACTACGCGTG
TAAATTCTACTGCAAATGCAGAGACACAAGCAACCTCAGCCTTCCAGACCCAGAAGCCATTGAAATCTAC
CTCTCTTTCACTGTTTTATAAAAAAGTGTATCGGCTAGCCTATCTCCGGCTAAATACACTTTGTGAACGC
CTTCTGTCTGAGCACCCAGAATTAGAACATATCATCTGGACCCTTTTCCAGCACACCCTGCAGAATGAGT
ATGAACTCATGAGAGACAGGCATTTGGACCAAATTATGATGTGTTCCATGTATGGCATATGCAAAGTGAA
GAATATAGACCTTAAATTCAAAATCATTGTAACAGCATACAAGGATCTTCCTCATGCTGTTCAGGAGACA
TTCAAACGTGTTTTGATCAAAGAAGAGGAGTATGATTCTATTATAGTATTCTATAACTCGGTCTTCATGC
AGAGACTGAAAACAAATATTTTGCAGTATGCTTCCACCAGGCCCCCTACCTTGTCACCAATACCTCACAT
TCCTCGAAGCCCTTACAAGTTTCCTAGTTCACCCTTACGGATTCCTGGAGGGAACATCTATATTTCACCC
CTGAAGAGTCCATATAAAATTTCAGAAGGTCTGCCAACACCAACAAAAATGACTCCAAGATCAAGAATCT
TAGTATCAATTGGTGAATCATTCGGGACTTCTGAGAAGTTCCAGAAAATAAATCAGATGGTATGTAACAG
CGACCGTGTGCTCAAAAGAAGTGCTGAAGGAAGCAACCCTCCTAAACCACTGAAAAAACTACGCTTTGAT
ATTGAAGGATCAGATGAAGCAGATGGAAGTAAACATCTCCCAGGAGAGTCCAAATTTCAGCAGAAACTGG
CAGAAATGACTTCTACTCGAACACGAATGCAAAAGCAGAAAATGAATGATAGCATGGATACCTCAAACAA
GGAAGAGAAATGAGGATCTCAGGACCTTGGTGGACACTGTGTACACCTCTGGATTCATTGTCTCTCACAG
ATGTGACTGTATAACTTTCCCAGGTTCTGTTTATGGCCACATTTAATATCTTCAGCTCTTTTTGTGGATA
TAAAATGTGCAGATGCAATTGTTTGGGTGATTCCTAAGCCACTTGAAATGTTAGTCATTGTTATTTATAC
AAGATTGAAAATCTTGTGTAAATCCTGCCATTTAAAAAGTTGTAGCAGATTGTTTCCTCTTCCAAAGTAA
AATTGCTGTGCTTTATGGATAGTAAGAATGGCCCTAGAGTGGGAGTCCTGATAACCCAGGCCTGTCTGAC
TACTTTGCCTTCTTTTGTAGCATATAGGTGATGTTTGCTCTTGTTTTTATTAATTTATATGTATATTTTT
TTAATTTAACATGAACACCCTTAGAAAATGTGTCCTATCTATCTTCCAAATGCAATTTGATTGACTGCCC
ATTCACCAAAATTATCCTGAACTCTTCTGCAAAAATGGATATTATTAGAAATTAGAAAAAAATTACTAAT
TTTACACATTAGATTTTATTTTACTATTGGAATCTGATATACTGTGTGCTTGTTTTATAAAATTTTGCTT
TTAATTAAATAAAAGCTGGAAGCAAAGTATAACCATATGATACTATCATACTACTGAAACAGATTTCATA
CCTCAGAATGTAAAAGAACTTACTGATTATTTTCTTCATCCAACTTATGTTTTTAAATGAGGATTATTGA
TAGTACTCTTGGTTTTTATACCATTCAGATCACTGAATTTATAAAGTACCCATCTAGTACTTGAAAAAGT
AAAGTGTTCTGCCAGATCTTAGGTATAGAGGACCCTAACACAGTATATCCCAAGTGCACTTTCTAATGTT
TCTGGGTCCTGAAGAATTAAGATACAAATTAATTTTACTCCATAAACAGACTGTTAATTATAGGAGCCTT
AATTTTTTTTTCATAGAGATTTGTCTAATTGCATCTCAAAATTATTCTGCCCTCCTTAATTTGGGAAGGT
TTGTGTTTTCTCTGGAATGGTACATGTCTTCCATGTATCTTTTGAACTGGCAATTGTCTATTTATCTTTT
ATTTTTTTAAGTCAGTATGGTCTAACACTGGCATGTTCAAAGCCACATTATTTCTAGTCCAAAATTACAA
GTAATCAAGGGTCATTATGGGTTAGGCATTAATGTTTCTATCTGATTTTGTGCAAAAGCTTCAAATTAAA
ACAGCTGCATTAGAAAAAGAGGCGCTTCTCCCCTCCCCTACACCTAAAGGTGTATTTAAACTATCTTGTG
TGATTAACTTATTTAGAGATGCTGTAACTTAAAATAGGGGATATTTAAGGTAGCTTCAGCTAGCTTTTAG
GAAAATCACTTTGTCTAACTCAGAATTATTTTTAAAAAGAAATCTGGTCTTGTTAGAAAACAAAATTTTA
TTTTGTGCTCATTTAAGTTTCAAACTTACTATTTTGACAGTTATTTTGATAACAATGACACTAGAAAACT

TGACTCCATTTCATCATTGTTTCTGCATGAATATCATACAAATCAGTTAGTTTTTAGGTCAAGGGCTTAC
TATTTCTGGGTCTTTTGCTACTAAGTTCACATTAGAATTAGTGCCAGAATTTTAGGAACTTCAGAGATCG
TGTATTGAGATTTCTTAAATAATGCTTCAGATATTATTGCTTTATTGCTTTTTTGTATTGGTTAAAACTG
TACATTTAAAATTGCTATGTTACTATTTTCTACAATTAATAGTTTGTCTATTTTAAAATAAATTAGTTGT
TAAGAGTCTTAA

**2) SEP15, 15kDa selenoprotein precursor**

```
NM_053102        842 taacttagttttgcatgcttacattggtcatccttttatgtacat----    887
                     |||.||||||||||.|||||.|||||.|||..||||     |||.|
NM_004261        879 taatttagttttgcttgcttccattgatcagtcttt-----tacttgagg
```

>gi|42741647|ref|NM_004261.3| Homo sapiens 15 kDa selenoprotein (SEP15), transcript variant 1, mRNA
GGAGAAGAAGGCGGGGCTAAAACTGGCGAAGGCGTGGCTTCTTGGCTGCTTGACGAAGTGTCGTGAATAA
AAGAAAGGAGACCGCAGAAGTAAAGAAGTGGGGAGTTTAGGCAAGTGCCTGATTTGGGTAATCGAAAGCA
CCCAGTGATTGTATTTGATGACTTTTAAGCTTTCATATGCCGTTATTTAATACCTGTCACTTCCAAATGA
GAGATGTAAGGGCAACGGCCGTTAGCGTTCTGTTTTGGATCAGGCTCTGGAGTGGACGCCCCTAGCTTAG
GGGTCCTTCTAGGCAGCCAGAAACCTGCGGAAAATGGTAGCGATGGCGGCTGGGCCGAGTGGGTGTCTGG
TGCCGGCGTTTGGGCTACGGTTGTTGTTGGCGACTGTGCTTCAAGCGGTGTCTGCTTTTGGGGCAGAGTT
TTCATCGGAGGCATGCAGAGAGTTAGGCTTTTCTAGCAACTTGCTTTGCAGCTCTTGTGATCTTCTCGGA
CAGTTCAACCTGCTTCAGCTGGATCCTGATTGCAGAGGATGCTGTCAGGAGGAAGCACAATTTGAAACCA
AAAAGCTGTATGCAGGAGCTATTCTTGAAGTTTGTGGATGAAAATTGGGAAGGTTCCCTCAAGTCCAAGC
TTTTGTTAGGAGTGATAAACCCAAACTGTTCAGAGGACTGCAAATCAAGTATGTCCGTGGTTCAGACCCT
GTATTAAAGCTTTTGGACGACAATGGGAACATTGCTGAAGAACTGAGCATTCTCAAATGGAACACAGACA
GTGTAGAAGAATTCCTGAGTGAAAAGTTGGAACGCATATAAATCTTGCTTAAATTTTGTCCTATCCTTTT
GTTACCTTATCAAATGAAATATTACAGCACCTAGAAAATAATTTAGTTTTGCTTGCTTCCATTGATCAGT
CTTTTACTTGAGGCATTAAATATCTAATTAAATCGTGAAATGGCAGTATAGTCCATGATATCTAAGGAGT
TGGCAAGCTTAACAAAACCCATTTTTTATAAATGTCCATCCTCCTGCATTTGTTGATACCACTAACAAAA
TGCTTTGTAACAGACTTGCGGTTAATTATGCAAATGATAGTTTGTGATAATTGGTCCAGTTTTACGAACA
ACAGATTTCTAAATTAGAGAGGTTAACAAGACAGATGATTACTATGCCTCATGTGCTGTGTGCTCTTTGA
AAGGAATGACAGCAGACTACAAAGCAAATAAGATATACTGAGCCTCAACAGATTGCCTGCTCCTCAGAGT
CTCTCCTATTTTTGTATTACCCAGCTTTCTTTTTAATACAAATGTTATTTATAGTTTACAATGAATGCAC
TGCATAAAAACTTTGTAGCTTCATTATTGTAAAACATATTCAAGATCCTACAGTAAGAGTGAAACATTCA
CAAAGATTTGCGTTAATGAAGACTACACAGAAAACCTTTCTAGGGATTTGTGTGGATCAGATACATACTT

GGCAAATTTTTGAGTTTTACATTCTTACAGAAAAGTCCATTTAAAAGTGATCATTTGTAAGACCAAAATA
TAAATAAAAAGTTTCAAAAATCTATCTGAATTTGGAATTCTTCTGGTTTGTTCTTTCATGTTTAAAAATG
ATGTTTTTCAATGCATTTTTTTCATGTAAGCCCTTTTTTTAGCCAAAATGTAAAAATGGCTGTAATATTT
AAAACTTATAACATCTTATTGTTGGTAATAGTGCTTTATATTTGTCTGATTTTATTTTTCAAAGTTTTTT
CATTTATGAACACATTTTCATTGGTATATTATTTAAGGAATATCTCTTGATATAGAATTTTTATATTAAA
AATGATTTTTCTTTGCTTAAAAAAAAAAAAAA

### 3) USP25, carboxyl-terminal hydrolase 25

```
NM_013918      3902 -cttttctgtatgtataaataata-----caaggtgattatttcaagaga   3945
                     ||||| |..||||||..|.||||     |||||||||||||||||||||
NM_013396      4129 actttt-taaatgtatttacaatatacagcaaggtgattatttcaagaga   4177
```

>gi|50312665|ref|NM_013396.3| Homo sapiens ubiquitin specific peptidase 25 (USP25), mRNA
GCGGCCGCCGTGGCCCTCACAGTCGGCGTTTCGCCGCCTGCCCGCGGTGCCCGCGCACGCCGGCCGCCAT
CGCCTTCGCGCCTGGCTGGCGGGGGCGCTGTCCTCCCAGGCCGTCCGCGCCGCTCCCTGGAGCTCGGCGG
AGCGCGGCAGCCAGGGCCGGCGGAGGCGCGAGGAGCCGGGCGCCACCGCCGCCGCCGCCGCCGCCGCCGC
GGGGGCCATGACCGTGGAGCAGAACGTGCTGCAGCAGAGCGCGGCGCAGAAGCACCAGCAGACGTTTTTG
AATCAACTGAGAGAAATTACGGGGATTAATGACACCCAGATACTACAGCAAGCCTTGAAGGATAGTAATG
GAAACTTGGAATTAGCAGTGGCTTTCCTTACTGCGAAGAATGCTAAGACCCCTCAGCAGGAGGAGACAAC
TTACTACCAAACAGCACTTCCTGGCAATGATAGATACATCAGTGTGGGAAGCCAAGCAGATACAAATGTG
ATTGATCTCACTGGAGATGATAAAGATGATCTTCAGAGAGCAATTGCCTTGAGTTTGGCCGAATCAAACA
GGGCATTCAGGGAGACTGGAATAACTGATGAGGAACAAGCCATTAGCAGAGTTCTTGAAGCCAGCATAGC
AGAGAATAAAGCATGTTTGAAGAGGACACCTACAGAAGTTTGGAGGGATTCTCGAAACCCTTATGATAGA
AAAAGACAGGACAAAGCTCCCGTTGGGCTAAAGAATGTTGGCAATACTTGTTGGTTTAGTGCTGTTATTC
AGTCATTATTTAATCTTTTGGAATTTAGAAGATTAGTTCTGAATTACAAGCCTCCATCAAATGCTCAAGA
TTTACCCCGAAACCAAAAGGAACATCGGAATTTGCCTTTTATGCGTGAGCTGAGGTATCTATTTGCACTT
CTTGTTGGTACCAAAAGGAAGTATGTTGATCCATCAAGAGCAGTTGAAATTCTTAAGGATGCTTTCAAAT
CAAATGACTCACAGCAGCAAGATGTGAGTGAGTTTACACACAAATTATTAGATTGGTTAGAAGATGCCTT
CCAAATGAAAGCTGAAGAGGAGACGGATGAAGAGAAGCCAAAGAACCCCATGGTAGAGTTGTTCTATGGC
AGATTCCTGGCTGTGGGAGTACTTGAAGGTAAAAAATTTGAAAACACTGAAATGTTTGGTCAGTACCCAC
TTCAGGTCAATGGGTTCAAAGATCTGCATGAGTGCCTAGAAGCTGCAATGATTGAAGGAGAAATTGAGTC
TTTACATTCAGAGAATTCAGGAAAATCAGGCCAAGAGCATTGGTTTACTGAATTACCACCTGTGTTAACA
TTTGAATTGTCAAGATTTGAATTTAATCAGGCATTGGGAAGACCAGAAAAAATTCACAACAAATTAGAAT
TTCCCCAAGTTTTATATTTGGACAGATACATGCACAGAAACAGAGAAATAACAAGAATTAAGAGGGAAGA
GATCAAGAGACTGAAAGATTACCTCACGGTATTACAACAAAGGCTAGAAAGATATTTAAGCTATGGTTCC
GGTCCCAAACGATTCCCCTTGGTAGATGTTCTTCAGTATGCATTGGAATTTGCCTCAAGTAAACCTGTTT

GCACTTCTCCTGTTGACGATATTGACGCTAGTTCCCCACCTAGTGGTTCCATACCATCACAGACATTACC
AAGCACAACAGAACAACAGGGAGCCCTATCTTCAGAACTGCCAAGCACATCACCTTCATCAGTTGCTGCC
ATTTCATCGAGATCAGTAATACACAAACCATTTACTCAGTCCCGGATACCTCCAGATTTGCCCATGCATC
CGGCACCAAGGCACATAACGGAGGAAGAACTTTCTGTGCTGGAAAGTTGTTTACATCGCTGGAGGACAGA
AATAGAAAATGACACCAGAGATTTGCAGGAAAGCATATCCAGAATCCATCGAACAATTGAATTAATGTAC
TCTGACAAATCTATGATACAAGTTCCTTATCGATTACATGCCGTTTTAGTTCACGAAGGCCAAGCTAATG
CTGGGCACTACTGGGCATATATTTTTGATCATCGTGAAAGCAGATGGATGAAGTACAATGATATTGCTGT
GACAAAATCATCATGGGAAGAGCTAGTGAGGGACTCTTTTGGTGGTTATAGAAATGCCAGTGCATACTGT
TTAATGTACATAAATGATAAGGCACAGTTCCTAATACAAGAGGAGTTTAATAAAGAAACTGGGCAGCCCC
TTGTTGGTATAGAAACATTACCACCGGATTTGAGAGATTTTGTTGAGGAAGACAACCAACGATTTGAAAA
AGAACTAGAAGAATGGGATGCACAACTTGCCCAGAAAGCTTTGCAGGAAAAGCTTTTAGCGTCTCAGAAA
TTGAGAGAGTCAGAGACTTCTGTGACAACAGCACAAGCAGCAGGAGACCCAGAATATCTAGAGCAGCCAT
CAAGAAGTGATTTCTCAAAGCACTTGAAAGAAGAAACTATTCAAATAATTACCAAGGCATCACATGAGCA
TGAAGATAAAAGTCCTGAAACAGTTTTGCAGTCGGCAATTAAGTTGGAATATGCAAGGTTGGTTAAGTTG
GCCCAAGAAGACACCCCACCAGAAACCGATTATCGTTTACATCATGTAGTGGTCTACTTTATCCAGAACC
AGGCACCAAAGAAAATTATTGAGAAAACATTACTAGAACAATTTGGAGATAGAAATTTGAGTTTTGATGA
AAGGTGTCACAACATAATGAAAGTTGCTCAAGCCAAACTGGAAATGATAAAACCTGAAGAAGTAAACTTG
GAGGAATATGAGGAGTGGCATCAGGATTATAGGAAATTCAGGGAAACAACTATGTATCTCATAATTGGGC
TAGAAAATTTTCAAAGAGAAAGTTATATAGATTCCTTGCTGTTCCTCATCTGTGCTTATCAGAATAACAA
AGAACTCTTGTCTAAAGGCTTATACAGAGGACATGATGAAGAATTGATATCACATTATAGAAGAGAATGT
TTGCTAAAATTAAATGAGCAAGCCGCAGAACTCTTCGAATCTGGAGAGGATCGAGAAGTAAACAATGGTT
TGATTATCATGAATGAGTTTATTGTCCCATTTTTGCCATTATTACTGGTGGATGAAATGGAAGAAAAGGA
TATACTAGCTGTAGAAGATATGAGAAATCGATGGTGTTCCTACCTTGGTCAAGAAATGGAACCACACCTC
CAAGAAAAGCTGACAGATTTTTTGCCAAAACTGCTTGATTGTTCTATGGAGATTAAAAGTTTCCATGAGC
CACCGAAGTTACCTTCATATTCCACGCATGAACTCTGTGAGCGATTTGCCCGAATCATGTTGTCCCTCAG
TCGAACTCCTGCTGATGGAAGATAAACTGCACACTTTCCCTGAACACACTGTATAAACTCTTTTTAGTTC
TTAACCCTTGCCTTCCTGTCACAGGGTTTGCTTGTTGCTGCTATAGTTTTTAACTTTTTTTTATTTTAAT
AACTGCAAAAGACAAAATGACTATACAGACTTTAGTCAGACTGCAGACAATAAAGCTGAAAATCGCATGG
CGCTCAGACATTTTAACCGGAACTGATGTATAATCACAAATCTAATTGATTTTATTATGGCAAAACTATG
CTTTTGCCACCTTCCTGTTGCAGTATTACTTTGCTTTTATCTTTTCTTTCTCAACAGCTTTCCATTCAGT
CTGGATCCTTCCATGACTACAGCCATTTAAGTGTTCAGCACTGTGTACGATACATAATATTTGGTAGCTT
GTAAATGAAATAAAGAATAAAGTTTTATTTATGGCTACCTATGTGTTTGTAAGCAGGTATATTGTATATT
AGTGTATTAGTAATACTAGATAAATGAATTTTGTCTGGGGATTAAGATTGGATAGTTAATAGATTAATAC
AATCTTTTAATTCTGCTCTAATGCTAGCAAATTGGAAAATGTTTAAGTCTTTGACACTTAAATTTATCTA
TATTTTTAACAAAGTTCTTGAACTTAGTATGGCACCGGAACCTGTTTTGAATTCAGTCAGGTTTTTACTC
AAGTAAGTGGTTGATTTTTTTTAAGTCAAACTACACTGAAACTTTTATCCTTTTCTTAGATTAATCTTAC
TTTTTAAATGTATTTACAATATACAGCAAGG**TGATTATTT**CAAGAGAATCCCAAAGTACTTGAATAAGGG
CTATTGTAAAATTTAAAAGAAATATTTATATATACACATATATACACATACACACATGTATATATATATT

CTTCATAATGGAGGACAATGTTTTGCAATATATAAATCATTCTATTTTTGTAAATTGTATATCACTTTAA
TTGAAAATGTTCTCTACTAATTAATACTGTGAAACAAAATTGATGTTGTTTAACTAGAAGTTATGAGTAT
CTTAACTGCCTTTATTCCTTTTCAAAAAGGAAAAAGCTGTAGAACATTTTGTAGATGAAACTACTGTTTA
AGATTAATGAATTAATATTGTGAATGAAAATCAAAATCCATACTTTAAAGGTAATCATGTTACTAACAAC
CTATTTTTGAATTCATAAAAATTTCTTTATAAATGATGTTTTGTGAACATAGTAAAATAGACCATTATAC
TATGTGTATGTTTGATACAGCGTCGCCAAAACTAGTGTTCTTTATTAGTGCCTCTCACAAAAGATCCTGG
ATGGAGGAGTAAGATGAAATATTATGCTATTATATGATGCTGTTTGTAAAGGTATTAATGTACTAGTAAG
GTGTTAATGACAAGGAATTAGTACTATTCCTGTTGTAAAGTTAGATTTTGCATATTGTATCTATCAAAAT
ATGTTTGGGTTTAGATTTTAAGTTGTCTACTGAGCAGATTTCTGCATTGGTTTTCCAGTCCTGTTAAAAG
TTTAGAAACTTCATATGTGTCATCACAGCTTTTGTAAAGAAAGTATCCTTAATATTTTATGACATTCT

### 4) TMEM30A, transmembrane protein 30A

```
NM_133718       2767 tgggtaacatatcagtttagattaatatgtgctt-------aaaagaaga   2809
                     |||||||..||| |.|||.|||||||||.|.|||       ||||.|| |
NM_018247       2824 tgggtaatgtat-acttttgattaatatatactttttttaaaaaacaa-a   2871
```

>gi|221219060|ref|NM_018247.3| Homo sapiens transmembrane protein 30A (TMEM30A), transcript variant 1, mRNA
ATCCTCCTCCCCTTTCCCCTCCCCATGTCCCTCCTTCTTCTCCCTCCTCCCTCTCTCCCTCCCTCTGGCG
TCTCCAGCCGACTTTCGCTTCCCGTCAGCAGCCTTTGGCGGAAGAGTGTCCCGCCTCTTCCGCTCTACAG
CGGAGGTGGCTGTGGCGGTGGCGCTGGTGGCTGCGGCGGCGGCGGCAGCGGCGCTCGAGCGGTTCCT
GTCAGGGTCAGCCGGCGGGCCCCCTGGGTGGTCCACCTGCAAATCGCGGAGCGGCGCCCCAGGGATCGAT
GGCGATGAACTATAACGCGAAGGATGAAGTGGACGGTGGGCCCCCGTGTGCTCCGGGGGGCACCGCGAAG
ACTCGGAGACCGGATAACACGGCCTTCAAACAGCAACGGCTGCCAGCTTGGCAGCCCATCCTTACGGCTG
GCACGGTGCTACCTATTTTCTTCATCATCGGTCTCATCTTCATTCCCATCGGCATTGGCATTTTTGTCAC
CTCCAACAACATCCGCGAGATCGAGATTGATTATACCGGAACAGAGCCTTCCAGTCCCTGTAATAAATGT
TTATCTCCGGATGTGACACCTTGCTTTTGTACCATTAACTTCACACTGGAAAAGTCATTTGAGGGCAACG
TGTTTATGTATTATGGACTGTCTAATTTCTATCAAAACCATCGTCGTTACGTGAAATCTCGAGATGATAG
TCAACTAAATGGAGATTCTAGTGCTTTGCTTAATCCCAGTAAGGAATGTGAACCTTATCGAAGAAATGAA
GACAAACCAATTGCTCCTTGTGGAGCTATTGCCAACAGCATGTTTAATGATACATTAGAATTGTTTCTCA
TTGGCAATGATTCTTATCCTATACCTATCGCTTTGAAAAAGAAAGGTATTGCTTGGTGGACAGATAAAAA
TGTGAAATTCAGAAATCCCCCTGGAGGAGACAACCTGGAAGAACGATTTAAAGGTACAACAAAGCCTGTG
AACTGGCTTAAACCAGTTTACATGCTGGATTCTGACCCAGATAATAATGGATTCATAAATGAGGATTTTA
TTGTTTGGATGCGTACTGCAGCATTACCTACTTTTCGCAAGTTGTATCGTCTTATAGAAAGGAAAAGTGA
TTTACATCCAACATTACCAGCTGGCCGATACTCTTTGAATGTCACATACAATTACCCTGTACATTATTTT
GATGGACGAAAACGGATGATCTTGAGCACTATTTCATGGATGGGAGGAAAAAATCCATTTTTGGGGATTG

CTTACATCGCTGTTGGATCCATCTCCTTCCTTCTGGGAGTTGTACTGCTAGTAATTAATCATAAATATAG
AAACAGTAGTAATACAGCTGACATTACCATTTAATTTTATATTATGAAAGCAAATCATCTGCATGTGCAT
CAAGGCCAGTCCTATTCAACCTAGCTTTCGAATGCTGATATCTGGTTAGTATGTCATTTTGAAGTTGGCA
CATAACTTTTCTAAAAAAAAGCAGTCTTTGTTGTTTGCTTCTTCCCTACGGATGACTTCTAAAAATATAT
GACGGGTATAAAAAAATTAGCTATATTGATCATATCAACACTGTAACTGCTGAAATGGCATTCTAATGTT
TGCTTTTTATTCGGACAGGCCACATGATGCATAGAGCCTCTTTCATGTGACTTGTGTCTACTGCTTAAAT
CTTTATGCTGTGTTGATGATATTATATTGACATATGAAGCTGTATATGTGTATGTATTTTGTGGAGAAAG
GGATTACAAGATGTATGAGTATAATGACTTGCTAACCTTTCAGGATTCAGAGAAAGATGAAGAAAGACCA
TATCTAAATAATACACTTCATCATTTTCATGTGTATAAATGCTTAAAGTACCATCTTTGTTGAGGTGGTT
CATGTATCCAGTTTATCCAGTACAGTTATTTGTCAAGCTTAGCTTTGATTTCAAAGGACACGCTTACCTT
GTCTGGCATAAGAATTAATGCTCATGTCTGCAGTGGTTGGGTAGGTCCTGCTTAGGAGAATTAAAAAATT
CCTCTTTCCGTTTGGTTGAATGTTGCAGTCAGGAACCCCAACTCACTTGGAATGTTTTTATATGTAATCA
TTTCCCTTGAAGCTTATACTTTATAAGGGAAGAAAGAATTCAGGTGATATGGGAAAACTGCTTGGCAGAC
CTTCATCTTCTGCCTCAACTGTAAACCACATGTAAGTGCTTAATGGAGACTGTTTTCATTCTTGTGATAT
TTAACATTCAGAAAATTACTTCAGCTTTGGAAATACTCAGGCTGTTTTTATTCTGCAGGTAAGTGTTTTG
ACTTAAGTACTAATATTCCAGAAATTTTTGAAAGCAGTAACCTTAATTTCCTATGTATTTCATTCCACTT
TTGCATATAGGTCAAATAGCAATGTGTATGCACATTCTCTTTAGTTAAGGCACCAATTGTTTTGGTTGGT
TTTCCTAAGACATACTTTAAAAAGATGTTCTATAAATTTCCTAATTAAATTATGGGGATTTTGGAGTATG
TACATGATAAATTATAATATGTATATGGTTGAAGTTATTTTATTTTTTACTAATGAATTATTTTAATATT
CCTTATTGAATAAATGCTGTAACTTGTTTGCTATGGAACTTATTCTTAAAGTTCTAGTTAAAAATAATTT
TTCCACATGCATGAAAATATGTATTAATCAGAGGTGGCTTAATTACATTGAAATTGCTTTTTTGTTGTTG
TTTTTTTACTGAAATAACTCATGTTTGTGTAGAAGAATGCCTGTTTACTCAGAGTTTATATTTTCCTTCA
GTTATATTTTAAATCAAAAGGTCTGGGTAATGTATACTTTTGATTAATATATACTTTTTTTAAAAAACAA
AAAACAATGTAATGGTTAATAGTAGAAATGTGCCACACTTTTCAAGTTTTATATAACATATGAAATTCAG
TTAAAAGAATGTGTGTTTCATAATGACTTTTAACTGGTAAAAATATTACTTGCACGAAGTACTTGATGTA
TGGTTATCCTGAAATTTCGGAGTATTTGGTGTGTTCTTTGTCTAAAAATAGTCTGTTTTGTCAGTCCTTC
AGAATATTATTTATTCTGAAGATTGTCCCTCTTGCACTTGGCAGTTTATTTTCGGGGATACATTGTTGGG
GGAGAGGGGTTTCTGCCACTCTTTCCAGATTGAGTCTGTGCTGTTTAAGGAGGACTACCATCCTGCAACT
CTTTTTCTAACTGGGGCACAGAGGATGTCGCTAAAGAAAAGTTGAAGAGCCCTTTCAGCACTTTCTCATC
TGTGGAGAAGATGGAATCTTAAAATACATTTGGAGTTTTATCTGTTTTACAAGTCCATTGATGGCCTAAG
TTCCTCCTGTTTTCTGCTGTTTGATCTCTAAGGAACTCCTGTTGCTAAATATGAAGAGTATGGAACATTC
GTATAGTCTCTGTGAAGCATGGGGGGAGGGAAGACATTTCTTTTTCTTATAGGCTTTATGCTCAAATGTC
ATAGTCTCCTTTCAAAGAATTGTGTTGCATTTTAAATGCACCCAGCTTAAGTAGAAGACATTGAAGGATG
CATTAATTTTCAGGAACTATTTTGAATTATGAAAAGATTCCCAATTGAAAAAATTATTCAACAAGTAAAA
GCTAAGAAATTTCATTGAAATCATAAGGCAGTTTAAGCATAAATTGATAAAAATAGCTGTGTACTACTAA
TTAATAGAAAATCATTCAACCAAGAGAAGAGTCAAGTGAATATCGTTTGTTTATTTGCTAGTGAGTTTCT
TTGTAACGTTGATTTTATTAAATGATAATATTTGGTTAGTATGTCCTATGTTAATAAAAATGAACAAAAT
TAATTTTGCTATGTTCAGGTGTCTTGATAAAATAACAATGCTCCAGTGTTGTTGCTTACATTTAGCACTA

```
AATTTTAACACAGCGTCAGTGAGTCCAGGTTTTAACTTCTTCATGCCTGGATGGGATAAAATGTAATTCA
TTGTTAAATTAATTCATATTTGTATTTATTAATCACTGTGACAACATTAACCATTTGTTCTTACCAGGAA
GTGGTCAGATTATCATCTGAGTTACAGTTAGACTGGCTAAGTTTGGTATTAGATCAAGGGGAATGTCCAG
TAAACAGAGAGGTAAGCATGATGGAAATAATGAAGTGGGGTACACAGGAAAAACCTGACTAGTGAGGAGG
AGCAGCTGAGAGATAGGGTCAGTGAATGCGGTTCAGCCTGCTACCTCTCCTGTCTTCATAGAACCATTGC
CTTAGAATTATTGTATGACACATTTTTTGTTGGTTAAGCTGTAAGGTTTTGTTCTTTGTGAACATGGGTA
TTTTGAGGGGAGGGTGGAGGGAGTAGGGAAGTGGTCCTTTTACAAGAATTTTGATGCATAAGTGTCTATT
GTAGGGTTTGGATGATCTAGTAAAGTGTTTTAGAACCCCTTTTTATCCCATGCACCATTCAGTAAACATA
AAAATCACAATTCTGCTAATGTCATTTGGAACTTCAAAATAAATATCTTGTCTAAAAACAAATC
```

### 5) SERINC1, serine incorporator 1 precursor

```
NM_019760       2644 catgcaattga-----------tatagtaa---atcgactagaaaat---   2676
                     ||||.||||||           |||.||||   .||.||..||||||
NM_020755       2870 catgtaattgaaatatatagattattgtaacctttcaacctgaaaatcaa   2919

NM_019760       2677 -tagtatgccacttaattaagataaaaatgcttagttatt--tatgt---   2720
                      .||||||           |||       |.|||||||||  |||||
NM_020755       2920 gcagtatg-----------aga-------gtttagttatttgtatgtgtc   2951
```

Mooring sequence is not conserved between human and mouse

# Supplementary information for Figure 3d

APOBEC1 RNA-editing targets (predicted or experimentally validated by Rosenberg B.R. et al, 2011) x Tag-seq_1123_RT_CgtH_FDR
=> 12 transcripts in common, namely 3251, 7009, 8904, 10056, 10237, 26999, 55610, 64770, 79868, 84726, 170506 and 219771 (NCBI Gene IDs)

Orthology table:

| Index | APOBEC1 RNA-editing target(Mouse) | Gene Symbol | Description | Human orthologue (blastn first hit) | Blast Score | E value | Gene ID (human) |
|---|---|---|---|---|---|---|---|
| 1 | NM_013556 | HPRT1 | hypoxanthine phosphoribosyltransferase 1 | NM_000194 | 1279 | 0.0 | 3251 |
| 2 | NM_026669 | TMBIM6 | transmembrane BAX inhibitor motif containing 6 | NM_003217 | 985 | 0.0 | 7009 |

| | | | | | | | |
|---|---|---|---|---|---|---|---|
| 3 | NM_170588 | CPNE1 | copine I | NM_152928 | 1679 | 0.0 | 8904 |
| 4 | NM_011811 | FARSB | phenylalanyl-tRNA synthetase, beta subunit | NM_005687 | 1731 | 0.0 | 10056 |
| 5 | NM_016752 | SLC35B1 | solute carrier family 35, member B1 | NM_005827 | 1178 | 0.0 | 10237 |
| 6 | NM_133769 | CYFIP2 | cytoplasmic FMR1 interacting protein 2 | NM_001037333 | 4785 | 0.0 | 26999 |
| 7 | NM_024260 | CCDC132 | coiled-coil domain containing 132 | NM_017667 | 3376 | 0.0 | 55610 |
| 8 | NM_172824 | CCDC14 | coiled-coil domain containing 14 | NM_022757 | 222 | 5e-56 | 64770 |
| 9 | NM_177130 | ALG13 | asparagine-linked glycosylation 13 homolog | NM_018466 | 440 | e-122 | 79868 |
| 10 | NM_001159634 | PRRC2B | proline-rich coiled-coil 2B | NM_013318 | 7685 | 0.0 | 84726 |
| 11 | NM_028136 | DHX36 | DEAH (Asp-Glu-Ala-His) box polypeptide 36 | NM_020865 | 3190 | 0.0 | 170506 |
| 12 | NM_026484 | CCNY | cyclin Y | NM_145012 | 1350 | 0.0 | 219771 |

RNA-editing table:

| **Index** | **APOBEC1 RNA-editing target(Mouse)** | **RNA editing status** | **Mouse mooring sequence in transcript** | **Human orthologue** | **Human mooring sequence** (By similarity) | **17 base *NlaIII* tag (RT) In human transcript** | **17 base *NlaIII* tag edition by APOBEC1** |
|---|---|---|---|---|---|---|---|
| 1 | NM_013556 | exp. validated | ugauuugcac | NM_000194 | ttatttgcac conserved | **TATCTTCTAAGAATTTT** | No, because NlaIII tag is too distant from the mooring sequence |
| 2 | NM_026669 | exp. validated | cuauuuugaa | NM_003217 | ctattttga conserved | CCACCCCGAATGGCTCA | No, because NlaIII tag is too distant from the mooring sequence |
| 3 | NM_170588 | predicted | ucaauaaaauuaauau | NM_152928 (U83246) | Not conserved | GAGCAGCTGGACGCTGA | No, the mooring sequence is not conserved |
| 4 | NM_011811 | predicted | ucucuggaaaucaauau | NM_005687 | tccctagaaatca atgt (rather conserved) | CCCTGCTCCTCCCTAGA | **Possible. NlaIII tag and APOBEC1 putative editing sites are overlapping.** |
| 5 | NM_016752 | predicted | acacuuaaauuauuau | NM_005827 | atatttaagttat tat (rather | CAGTGGGTGGGCACTGT | No, because NlaIII tag is too distant from the mooring sequence |

| | | | | | | | |
|---|---|---|---|---|---|---|---|
| | | | | | conserved) | | |
| 6 | NM_133769 | predicted | ucuguguugauuacuau | NM_001037333 | tctgtgtggatta cta (conserved) | CTGGTTTCTCCATAGCA | No, because NlaIII tag is too distant from the mooring sequence |
| 7 | NM_024260 | Predicted | acucucauaauuauuau | NM_017667 | Not conserved | TAAGTAAAATAGTTGAA | No, the mooring sequence is not conserved |
| 8 | NM_172824 | predicted | acuacaagaucauuau | NM_022757 | attgcaagatcat tat | TAAGCAGCACGTTTTAA | No, because NlaIII tag is too distant from the mooring sequence |
| 9 | NM_177130 | predicted | acaauauaaaucaauau | NM_018466 | ataatataattaa taa | AAGTGCGTGTTTGTTAC | No, because NlaIII tag is too distant from the mooring sequence |
| 10 | NM_001159634 | predicted | ucaccaaaaucaauau | NM_013318 | Not conserved | GCCAGACCCCTCCCACC | No, the mooring sequence is not conserved |
| 11 | NM_028136 | predicted | acucuaaaauuauuau | NM_020865 | Not conserved | CCCACTCTTTGGGAGTA | No, the mooring sequence is not conserved |
| 12 | NM_026484 | predicted | ucuaucuagauuaauau | NM_145012 | tctagatcacatg | GCGCAGACTTCCAAATA | No, because NlaIII tag is too distant from the mooring sequence |

**1) HPRT1, hypoxanthine phosphoribosyltransferase 1**

```
NM_013556        961 cgtccccagactttTGATTTGCACtatgagcctataggc---cagcctac   1007 (Mm)
                     |.|.||.|.|||.||.|||||||||||||||||||||.|   |||  |.|
NM_000194        985 cattcctaaactgtTTATTTGCACtatgagcctatagactatcag--ttc   1032 (Hs)
```

```
>gi|164518913|ref|NM_000194.2| Homo sapiens hypoxanthine phosphoribosyltransferase 1 (HPRT1), mRNA
GGCGGGGCCTGCTTCTCCTCAGCTTCAGGCGGCTGCGACGAGCCCTCAGGCGAACCTCTCGGCTTTCCCG
CGCGGCGCCGCCTCTTGCTGCGCCTCCGCCTCCTCCTCTGCTCCGCCACCGGCTTCCTCCTCCTGAGCAG
TCAGCCCGCGCGCCGGCCGGCTCCGTTATGGCGACCCGCAGCCCTGGCGTCGTGATTAGTGATGATGAAC
CAGGTTATGACCTTGATTTATTTTGCATACCTAATCATTATGCTGAGGATTTGGAAAGGGTGTTTATTCC
TCATGGACTAATTATGGACAGGACTGAACGTCTTGCTCGAGATGTGATGAAGGAGATGGGAGGCCATCAC
ATTGTAGCCCTCTGTGTGCTCAAGGGGGGCTATAAATTCTTTGCTGACCTGCTGGATTACATCAAAGCAC
TGAATAGAAATAGTGATAGATCCATTCCTATGACTGTAGATTTTATCAGACTGAAGAGCTATTGTAATGA
CCAGTCAACAGGGGACATAAAAGTAATTGGTGGAGATGATCTCTCAACTTTAACTGGAAAGAATGTCTTG
ATTGTGGAAGATATAATTGACACTGGCAAAACAATGCAGACTTTGCTTTCCTTGGTCAGGCAGTATAATC
CAAAGATGGTCAAGGTCGCAAGCTTGCTGGTGAAAAGGACCCCACGAAGTGTTGGATATAAGCCAGACTT
TGTTGGATTTGAAATTCCAGACAAGTTTGTTGTAGGATATGCCCTTGACTATAATGAATACTTCAGGGAT
```

TTGAATCATGTTTGTGTCATTAGTGAAACTGGAAAAGCAAAATACAAAGCCTAAGATGAGAGTTCAAGTT
GAGTTTGGAAACATCTGGAGTCCTATTGACATCGCCAGTAAAATTATCAATGTTCTAGTTCTGTGGCCAT
CTGCTTAGTAGAGCTTTTTG**CATGTATCTTCTAAGAATTTT**ATCTGTTTTGTACTTTAGAAATGTCAGTT
GCTGCATTCCTAAACTGT**TTATTTGCAC**TATGAGCCTATAGACTATCAGTTCCCTTTGGGCGGATTGTTG
TTTAACTTGTAAATGAAAAAATTCTCTTAAACCACAGCACTATTGAGTGAAACATTGAACTCATATCTGT
AAGAAATAAAGAGAAGATATATTAGTTTTTTAATTGGTATTTTAATTTTTATATATGCAGGAAAGAATAG
AAGTGATTGAATATTGTTAATTATACCACCGTGTGTTAGAAAAGTAAGAAGCAGTCAATTTTCACATCAA
AGACAGCATCTAAGAAGTTTTGTTCTGTCCTGGAATTATTTTAGTAGTGTTTCAGTAATGTTGACTGTAT
TTTCCAACTTGTTCAAATTATTACCAGTGAATCTTTGTCAGCAGTTCCCTTTTAAATGCAAATCAATAAA
TTCCCAAAAATTTAAAAAAAAAAAAAAAAAAAAAA

## 2） TMBIM6, transmembrane BAX inhibitor motif containing 6

```
NM_026669        928 gataatg-aaagcatcaggaaagcttttgtacttagtggttttctctatt    976   (Mm)
                     ||||||| ||||||||||.|||||||||||||||.|||||||.|||||||
NM_003217        934 gataatgaaaagcatcagaaaagcttttgtactttgtggtttcctctatt    983   (Hs)

NM_026669        977 ttgaaatttttgatc-aaaaactgattag-tgaatatagtttggagttt   1024   (Mm)
                     ||| ||||||||||| ||||||||||||| .||||||||||||||||||
NM_003217        984 ttg-aatttttgatcaaaaaactgattagcagaatatagtttggagttt   1032   (Hs)
```

>gi|148746208|ref|NM_003217.2| Homo sapiens transmembrane BAX inhibitor motif containing 6 (TMBIM6), transcript variant 1, mRNA
GAGAGGCGAACGCCGGGTAAAAGATCGGGAGCGGAAGTGGGCGAGTCAGAGCACATCCGGTGTTAGAAGC
GCTGGTAGGCCTTGGAGAGGCGGGTTAGGAAGAGTGGAGACTGCTGCACGGACTCTGGAACCATGAACAT
ATTTGATCGAAAGATCAACTTTGATGCGCTTTTAAAATTTTCTCATATAACCCCGTCAACGCAGCAGCAC
CTGAAGAAGGTCTATGCAAGTTTTGCCCTTTGTATGTTTGTGGCGGCTGCAGGGGCCTATGTCCATATGG
TCACTCATTTCATTCAGGCTGGCCTGCTGTCTGCCTTGGGCTCCCTGATATTGATGATTTGGCTGATGGC
AACACCTCATAGCCATGAAACTGAACAGAAAAGACTGGGACTTCTTGCTGGATTTGCATTCCTTACAGGA
GTTGGCCTGGGCCCTGCCCTGGAGTTTTGTATTGCTGTCAACCCCAGCATCCTTCCCACTGCTTTCATGG
GCACGGCAATGATCTTTACCTGCTTCACCCTCAGTGCACTCTATGCCAGGCGCCGTAGCTACCTCTTTCT
GGGAGGTATCTTGATGTCAGCCCTGAGCTTGTTGCTTTTGTCTTCCCTGGGGAATGTTTTCTTTGGATCC
ATTTGGCTTTTCCAGGCAAACCTGTATGTGGGACTGGTGGTCATGTGTGGCTTCGTCCTTTTTGATACTC
AACTCATTATTGAAAAGGCCGAACATGGAGATCAAGATTATATCTGGCACTGCATTGATCTCTTCTTAGA
TTTCATTACTGTCTTCAGAAAACTCATGATGATCCTGGCCATGAATGAAAAGGATAAGAAGAAAGAGAAG
AAATGAAGTGACCATCCAGCCTTTCCCAATTAGACTTCCTCTCCTTCCACCCCTCATTTCCTTTTTGCAC

ACATTACAGGTGGTGTGTTCTGTGATAATGAAAAGCATCAGAAAAGCTTTTGTACTTTGTGGTTTCCT**CT**
**ATTTTGA**ATTTTTTGATCAAAAAACTGATTAGCAGAATATAGTTTGGAGTTTGGCTTCATCTTCCTGGGG
TTCCCCTCACTCCCTTTTTTGTCAACCCCATCTGTAGCCTCTTCCTCTACTCAGGCAGTCGACCCGCCAC
GATGAGAAGTGGGACCAGCAGAGGGCGCCAACTTCAGGAGTCCGCTTTCCCACCAGGCTTCATTCACCCA
GTGGACCTGAACTGTTTGGTAGAGCCACCCGGCCCTTCCTTCCTCATTGTTGTTTGGTATGCGCACAGTT
CCTGTGGGACTGGGCCGTGAGTTTTCCATTGGAAAGAAGTTCAGTGGTCCCATTGTTAACTCAGCCTCAA
ATCTCAACTGTCAGGCCCTACAAAGAAAATGGAGAGCCTCTTCTGGTGGATGCTTTGCTCCCTCTGAGCT
GCCCATGCTGGTCTGGCAAACACACCTTTCTGCTTTGCCTTCACAAAAGTAATGTGTTCCCTTTCCCACC
CCTTGCCTGACCCTCAGGGAGTCAGCCTGCTTCCATCCATGGGTGGGAAGACTTCAGCACAAAGGAAAGA
CTAATTCTTGTCAGGCATTTTTGAAAAGGCTGATTATGTGTATCAAGGTACAGCATCGTAGGGTTCCCCT
AAACTTGCCCTGTTTTTGTTTTTTTAGTTTGTTATCCCCTTACTGAGCGGCCTCTACTAGGTGGCTGTGA
TTAAATGTCCCAAGCAAGGATAGGGAAGGGGAATGGTTGAGCCTCTGGAGATCATTGTAACCAATCCTGC
CAGACCTGTTTGGGGCAGTGGGGAGCAAACCTAGATAAGGACCTGTTTGGGGCAGCAGGGAGCAAAATCT
CCTTTAACAACCAAGCAGTTCCTCATTCACATCAACAGAGCGAGGCTGTGATAACTTAGGAGGCAGCAAT
CCTAATAGTCCTTCAGTGCATTTTAGTCTGTCTCCAACTGGACACCAGTAGGTAGTGTCAAGCCAGAGAT
TCGGGGCAGTAGATAAATGTTCATTTTACTGATGCACTTTAGTTTTTGGTCTGTTACCTGTTTTCCAGAA
ATTTGTGGCCTTTTAGGCGGGAGTTAGGCGACCAAACCAGTGAGAGCCCCAATCCCTGCAGTTTTGTGGC
TTCAAGTGTGGGTGGACAGTCCTAATGGGGATCTCCAGCTCCTTCCTGTGGGCTGCCACAGACAGCTACC
CCCAGAAGGGTCAATGTTGGGAGTGGTTGTGGCTCTGAGCTGCTCTACAGAGCTTCAGTGTGAGAGGATC
GAGCCATTGAAAGCTCATTACCAGTAGGACATAATTTTTGGCTCTCCCTATTCACAACCAGTGCACAGTT
TGACACAGTGGCCTCAGGTTCACAGTGCACCATGTCACTGTGCTATCCTACGAAATCATTTGTTTCTAAG
TTGTGTTTATTCCTGGAGTGA**CATGCCACCCCGAATGGCTCA**CTTTCACTGAGGATGCTGTCCTCTGATT
TAGCTGCTGCCTCCAGCCTCTGGCTTGAGAACTTACTAAAGGCACTTCCTTCCTGTTAAACCCCTGTTAA
CTCTCCATAAATTTGGTGATTCTCTGCTAGGCCTAAGATTTTGAGTTAACATCTCTTGAAGCCAAACTCC
ACCTTCTGTGCTTTTTGCTTGGGATAATGGAGTTTTTCTTTAGAAACAGTGCCAAGAATGACAAGATATT
AAAAAAAAAAAAGAAAGAAAAAAAAAAAAAACACCTACTTTTAAAGAAAATACCTAACAGATTTTTAATAT
AGTTATCTCTACCACTTTCTTTTCTAGTTTCTTGATTTTCAGCTCAGGCTGCATTCTAACTCATACTGTG
AAGACAAAGGTGTTTTTGATTCAGAAATATATGAAATCTGCATAGTCTTAATTTGTAAAAAATAAAGAAA
ATTCCTTAACCTTTA

**3) CPNEI, copine I**

NM_170588 3476 aaaatatatctatgtatcaataaaattaatatatccacctcctcaaaaaa 3525 (Mm)

U83246 1985 -------------------------------------------------- 1985 (Hs)

NM_170588 3526 aaaaaaaaaaaaaaaaa 3542

The mouse NM_170588 transcript is longer than the human NM_152928 (U83246). The mooring sequence is close to the 3’ boundary.

⇨ The mooring sequence is not conserved

**4) FARSB, phenylalanyl-tRNA synthetase, beta subunit**

```
NM_011811       1699 tcctgatgttatcaccaaatttgagctgaccatgccctgttcctctctgg   1748 (Mm)
                     ||||||.||||||||||||||||||||||||||||||||.|||||.||.|
NM_005687       1730 tcctgacgttatcaccaaatttgagctgaccatgccctgctcctccctag   1779 (Hs)

NM_011811       1749 aaatcaatattgagccctttttgtg-aggttggtctctg-----------   1786 (Mm)
                     ||||||||.|||..||||||||||| ||.||||||||||
NM_005687       1780 aaatcaatgttggaccctttttgtgaagattggtctctgtggtgtgattc   1829 (Hs)
```

```
>gi|124028524|ref|NM_005687.3| Homo sapiens phenylalanyl-tRNA synthetase, beta subunit (FARSB), mRNA
AGCTCGCTGCGCAGGCGCAGTGAGTTCGACACACCATGCCGACTGTCAGCGTGAAGCGTGATCTGCTCTT
CCAAGCCCTGGGCCGCACCTACACTGACGAAGAATTTGATGAACTATGTTTTGAATTTGGTCTGGAGCTT
GATGAAATTACATCTGAGAAGGAAATAATAAGTAAAGAACAAGGTAATGTAAAGGCAGCAGGAGCCTCTG
ATGTTGTTCTTTACAAAATTGACGTCCCTGCCAATAGATATGATCTCCTGTGTCTGGAAGGATTGGTTCG
AGGACTTCAGGTCTTCAAAGAAAGGATAAAGGCTCCAGTGTATAAACGGGTAATGCCTGATGGAAAAATC
CAGAAATTGATTATCACAGAAGAGACAGCTAAGATACGTCCTTTTGCGGTAGCAGCAGTTCTCCGTAATA
TAAAGTTTACTAAAGATCGATATGACAGCTTCATTGAACTTCAGGAGAAATTACATCAGAATATTTGCAG
GAAAAGAGCACTGGTTGCCATTGGTACCCATGATTTGGACACTTTGTCGGGCCCATTTACTTATACTGCA
AAGCGTCCTTCAGATATCAAATTCAAGCCTCTAAATAAGACCAAGGAGTATACAGCCTGTGAACTGATGA
ACATATACAAGACTGACAATCACCTGAAACATTATTTACATATCATTGAAAACAAACCCCTGTATCCAGT
TATCTATGATAGCAATGGTGTCGTCCTTTCAATGCCTCCCATCATCAATGGGGATCATTCCAGAATAACA
GTAAATACTAGAAATATTTTTATTGAATGCACGGGAACTGACTTTACTAAGGCAAAAATAGTTCTTGATA
TTATTGTCACCATGTTCAGTGAATATTGTGAGAATCAATTTACGGTCGAAGCTGCTGAAGTGGTTTTTCC
TAATGGAAAATCACATACCTTTCCAGAATTAGCTTACCGAAAGGAGATGGTGAGAGCTGACCTAATTAAC
AAAAAAGTTGGAATCAGAGAAACTCCAGAAAATCTTGCCAAACTTCTGACCAGGATGTATTTAAAATCAG
AAGTCATAGGTGATGGGAATCAGATTGAGATTGAAATCCCTCCAACCAGAGCTGACATTATCCATGCATG
TGATATTGTAGAAGATGCAGCTATTGCTTATGGATATAACAACATTCAGATGACTCTCCCGAAAACTTAC
ACCATAGCTAATCAATTTCCTCTTAATAAGCTCACTGAACTTCTCCGACATGACATGGCAGCCGCTGGCT
TCACTGAAGCACTTACCTTTGCCCTGTGCTCCCAAGAAGATATTGCTGATAAACTAGGTGTGGATATCTC
TGCAACAAAGGCAGTCCACATAAGTAATCCTAAAACAGCTGAATTTCAGGTGGCACGCACTACCCTTCTT
CCTGGCCTCCTGAAGACCATAGCAGCAAATCGTAAGATGCCCCTTCCACTGAAACTGTTTGAAATCTCTG
ACATTGTAATAAAAGATTCTAATACAGATGTAGGTGCAAAAAACTACAGACATCTCTGTGCTGTTTATTA
CAACAAGAATCCTGGGTTTGAGATCATTCATGGGCTGCTGGACAGAATTATGCAGTTGCTCGATGTGCCT
CCTGGTGAAGACAAGGGGGGATATGTGATCAAAGCATCAGAAGGGCCTGCTTTCTTCCCCGGGCGATGTG
```

```
CAGAGATCTTTGCCAGGGGTCAAAGCGTCGGGAAGCTTGGGGTCCTTCATCCTGACGTTATCACCAAATT
TGAGCTGACCATGCCCTGCTCCTCCCTAGAAATCAATGTTGGACCCTTTTTGTGAAGATTGGTCTCTGTG
GTGTGATTCTCTTCCCAGGTGTCCCTTTCTCCTCCCCTAGTGTCCTTAAGTCCTCCTCCACAGGGAACAT
CTATTTGGGCTTTGATGTTTAATAAAGTAGAAAGCACTGTCTGGCTGTGTGGGTAGAGACCATCCTTTCC
CTGCATATTAGGCCAGCTTGTGCCATATACCAGTGTGGTGTCTGTGTGTGAAGCTGCATTGTTGGGTAAA
AGCCCCGTGGAGTGCTGGAGAAATGCACTAGCAGAGTGCAGGATCTGTTCTGAAAGGCAGACGTGCTCCT
CAGACATCAGAACATCACATTGGAACGGATTACTCCTGCATAACAAGATCCCATTTCTTTCCTTATTGAT
AAAACAAGATAATCGATGAGAATTCATGTTGCATGAGTTCGAGATAACTGAGGGGTTTAGCTT
```

**5) SLC35B1, solute carrier family 35, member B1**

```
NM_016752       1050 cccactaggaagaaagaggcttcctctac-tccagaaacacttaaattat   1098 (Mm)
                     |||||||||||||.||||.||.||||.|| ||.|| ||.|.||||.||||
NM_005827       1049 cccactaggaagagagagactacctccacatcaag-aatatttaagttat   1097 (Hs)

NM_016752       1099 tatctcgaacagtggca--tcttgggaaaatggactcgggtcat---gat   1143 (Mm)
                     ||||||.|||||||.||  |||||||||||||||||    |.||   .||
NM_005827       1098 tatctcaaacagtgacatctcttgggaaaatggact----taataggaat   1143 (Hs)
```

```
>gi|5032212|ref|NM_005827.1| Homo sapiens solute carrier family 35, member B1 (SLC35B1), mRNA
GATGTCCGGCTGGAGCTGTCGCCTCCGCCGCCGCTGCTGCCGGTGCCGGTTGTGAGCGGGTCTCCAGTCG
GCTCCTCTGGGCGTCTCATGGCCTCTAGCAGCTCCCTGGTGCCCGACCGGCTGCGCCTGCCGCTCTGCTT
CCTGGGTGTCTTTGTCTGCTATTTTTACTATGGGATCCTGCAGGAAAAGATAACAAGAGGAAAGTATGGG
GAAGGAGCCAAGCAGGAGACGTTCACCTTTGCCTTAACTTTGGTCTTCATTCAATGTGTGATCAATGCTG
TGTTTGCCAAGATCTTGATCCAGTTTTTTGACACTGCCAGGGTGGATCGTACCCGGAGCTGGCTCTATGC
TGCCTGTTCTATCTCCTATCTGGGTGCCATGGTCTCCAGCAATTCAGCACTACAGTTTGTCAACTACCCA
ACTCAGGTCCTTGGTAAATCCTGCAAGCCAATCCCAGTCATGCTCCTTGGGGTGACCCTCTTGAAGAAGA
AGTACCCGTTGGCCAAGTACCTGTGTGTGCTGTTAATTGTGGCTGGAGTGGCCCTTTTCATGTACAAACC
CAAGAAAGTTGTTGGGATAGAAGAACACACAGTCGGCTATGGAGAGCTACTCTTGCTATTATCGCTGACC
CTGGATGGACTGACTGGTGTTTCCCAGGACCACATGCGGGCTCATTACCAAACAGGCTCCAACCACATGA
TGCTGAACATCAACCTTTGGTCGACATTGCTGCTGGGAATGGGAATCCTGTTCACTGGGGAGCTCTGGGA
GTTCTTGAGCTTTGCTGAAAGGTACCCTGCCATCATCTATAACATCCTGCTCTTTGGGCTGACCAGTGCC
CTGGGTCAGAGCTTCATCTTTATGACGGTTGTGTATTTTGGTCCCCTGACCTGCTCCATCATCACTACAA
CTCGAAAGTTCTTCACAATTTTGGCCTCTGTGATCCTCTTCGCCAATCCCATCAGCCCCATGCAGTGGGT
GGGCACTGTGCTTGTGTTCCTGGGTCTTGGTCTTGATGCCAAGTTTGGGAAAGGAGCTAAGAAGACATCC
CACTAGGAAGAGAGAGACTACCTCCACATCAAGAATATTTAAGTTATTATCTCAAACAGTGACATCTCTT
GGGAAAATGGACTTAATAGGAATATGGGACTGAGTTCCAGTCTTTTTTAATAAAATAAAATCAAGC
```

**6) CYFIP2, cytoplasmic FMR1 interacting protein 2**

```
NM_133769        857 tgaggagctgctggcagacattgtcaacatctgtgttgattactatgaga    906 (Mm)
                     |||||||||||||||.||||||||||||||||||||.||||||||.||||
NM_001037333     840 tgaggagctgctggctgacattgtcaacatctgtgtggattactacgaga    889 (Hs)
```

>gi|82617633|ref|NM_001037333.1| Homo sapiens cytoplasmic FMR1 interacting protein 2 (CYFIP2), transcript variant 1, mRNA

CGGGGCCGGGGCGGGGCCGAGCGCGGCGCAGCGGAGCGGGGCAGAGCATCCTGCGCCCCGGCGCGGGGCC
CTGCGGTAGCCTCAGGCCCCTCCCCTGGACCCGCCGCAGAGCCAGTGCAGAATACAGAAACTGCAGCCAT
GACCACGCACGTCACCCTGGAAGATGCCCTGTCCAACGTGGACCTGCTTGAAGAGCTTCCCCTCCCCGAC
CAGCAGCCATGCATCGAGCCTCCACCTTCCTCCATCATGTACCAGGCTAACTTTGACACAAACTTTGAGG
ACAGGAATGCATTTGTCACGGGCATTGCAAGGTACATTGAGCAGGCTACAGTCCACTCCAGCATGAATGA
GATGCTGGAGGAAGGACATGAGTATGCGGTCATGCTGTACACCTGGCGCAGCTGTTCCCGGGCCATTCCC
CAGGTGAAATGCAACGAGCAGCCCAACCGAGTAGAGATCTATGAGAAGACAGTAGAGGTGCTGGAGCCGG
AGGTCACCAAGCTCATGAAGTTCATGTATTTTCAGCGCAAGGCCATCGAGCGGTTCTGCAGCGAGGTGAA
GCGGCTGTGCCATGCCGAGCGCAGGAAGGACTTTGTCTCTGAGGCCTACCTCCTGACCCTTGGCAAGTTC
ATCAACATGTTTGCTGTCCTGGATGAGCTAAAGAACATGAAGTGCAGCGTCAAGAATGACCACTCTGCCT
ACAAGAGGGCAGCACAGTTCCTGCGGAAGATGGCAGATCCCCAGTCTATCCAGGAGTCGCAGAACCTTTC
CATGTTCCTGGCCAACCACAACAGGATCACCCAGTGTCTCCACCAGCAACTTGAAGTGATCCCAGGCTAT
GAGGAGCTGCTGGCTGACATTGTCAACA**TCTGTGTGGATTACTAC**GAGAACAAGATGTACCTGACTCCCA
GTGAGAAACATATGCTCCTCAAGGTGATGGGCTTTGGCCTCTACCTAATGGATGGAAATGTCAGTAACAT
TTACAAACTGGATGCCAAGAAGAGAATTAATCTTAGCAAAATTGATAAATTCTTTAAGCAGCTGCAGGTG
GTGCCCCTTTTCGGCGACATGCAGATAGAGCTGGCCAGATACATTAAGACCAGTGCTCACTATGAAGAGA
ACAAGTCCAAGTGGACGTGCACCCAGAGCAGCATCAGCCCCCAGTACAATATCTGCGAGCAGATGGTTCA
GATCCGGGATGACCACATCCGCTTCATCTCCGAGCTCGCTCGCTACAGCAACAGTGAGGTGGTGACGGGC
TCAGGGCTGGACAGCCAGAAGTCAGACGAGGAGTATCGCGAGCTCTTCGACCTAGCCCTGCGGGGTCTGC
AGCTTCTATCCAAGTGGAGCGCCCACGTCATGGAGGTGTACTCTTGGAAGCTGGTTCATCCCACAGACAA
GTTCTGCAACAAGGACTGTCCTGGCACCGCGGAGGAATATGAGAGAGCCACACGCTACAATTACACCAGT
GAGGAAAAATTTGCCTTCGTTGAGGTGATCGCCATGATCAAAGGCCTGCAGGTGCTCATGGGCAGGATGG
AGAGCGTCTTCAACCAGGCCATCAGGAACACCATCTACGCGGCATTGCAGGACTTCGCCCAGGTGACGCT
GCGTGAGCCCCTGCGGCAGGCGGTACGGAAGAAGAAGAATGTCCTCATCAGCGTCCTACAGGCAATTCGA
AAGACCATCTGTGACTGGGAGGGAGGGCGAGAGCCCCCTAATGACCCATGCTTGAGAGGGGAGAAGGACC
CCAAAGGTGGATTTGATATCAAGGTGCCCCGGCGTGCTGTGGGGCCATCCAGCACACAGCTGTACATGGT
GCGGACCATGCTTGAATCACTCATTGCAGACAAAAGCGGCTCCAAGAAGACCCTGAGGAGCAGCCTGGAT
GGACCCATTGTCCTCGCCATAGAGGACTTTCACAAACAGTCCTTCTTCTTCACACATCTGCTCAACATCA
GTGAAGCCCTGCAGCAGTGTTGTGACCTCTCCCAGCTCTGGTTCCGAGAATTCTTCCTGGAGTTAACCAT
GGGCCGACGAATCCAGTTCCCCATCGAGATGTCCATGCCCTGGATTCTAACGGACCATATCCTGGAAACC
AAAGAACCTTCCATGATGGAGTATGTCCTCTACCCTCTGGATCTGTACAACGACAGCGCCTACTATGCTC
TGACCAAGTTTAAAAAGCAGTTCCTGTACGATGAGATAGAAGCTGAGGTGAACCTGTGTTTTGATCAGTT

TGTCTACAAGCTGGCAGACCAGATCTTTGCTTACTACAAAGCCATGGCTGGCAGTGTCCTGTTGGATAAA
CGTTTTCGAGCTGAGTGTAAGAATTATGGCGTCATCATTCCGTATCCACCGTCCAATCGCTATGAAACAC
TGCTGAAGCAGAGACACGTCCAGCTGTTGGGTAGATCAATTGACTTGAACAGACTCATTACCCAGCGCAT
CTCTGCCGCCATGTATAAATCCTTGGACCAAGCTATCAGCCGCTTTGAGAGTGAGGACCTGACCTCCATT
GTGGAGCTGGAGTGGCTGCTGGAGATTAACCGGCTCACGCATCGGCTGCTCTGTAAGCATATGACGCTGG
ACAGCTTCGATGCCATGTTCCGAGAGGCCAATCACAATGTGTCCGCCCCCTATGGCCGTATCACCCTGCA
TGTCTTCTGGGAACTGAACTTTGACTTTCTCCCCAACTACTGCTACAATGGGTCCACTAACCGTTTTGTG
CGGACTGCCATTCCTTTCACCCAAGAACCACAACGAGACAAACCTGCCAACGTCCAGCCTTATTACCTCT
ATGGATCCAAGCCTCTCAACATTGCCTACAGCCACATCTACAGCTCCTACAGGAATTTCGTGGGGCCACC
TCATTTCAAGACTATCTGCAGACTCCTGGGTTATCAGGGCATCGCTGTGGTCATGGAGGAACTGCTAAAG
ATTGTGAAGAGCTTGCTCCAAGGAACCATTCTCCAGTATGTGAAAACACTGATAGAGGTGATGCCCAAGA
TATGCCGCTTGCCCCGACATGAGTATGGCTCCCCAGGGATCCTGGAGTTCTTCCACCACCAGCTGAAGGA
CATCATTGAGTACGCAGAGCTCAAAACAGACGTGTTCCAGAGCCTGAGGGAAGTGGGCAATGCCATCCTC
TTCTGCCTCCTCATAGAGCAAGCTCTGTCTCAGGAGGAGGTCTGCGATTTGCTCCATGCCGCACCCTTCC
AAAACATCTTGCCTAGAGTCTACATCAAAGAGGGGGAGCGCCTGGAGGTCCGGATGAAACGTCTGGAAGC
CAAGTATGCCCCGCTCCACCTGGTCCCTCTGATCGAGCGGCTGGGGACCCCTCAGCAAATCGCCATTGCT
CGCGAGGGTGACCTCCTGACCAAGGAGCGGCTGTGCTGTGGCCTGTCCATGTTCGAGGTCATCCTGACCC
GCATTCGGAGCTACCTGCAGGACCCCATCTGGCGGGGCCCACCGCCCACCAATGGCGTCATGCACGTCGA
TGAGTGTGTGGAGTTCCACCGGCTGTGGAGCGCCATGCAGTTCGTGTACTGCATCCCTGTGGGAACCAAC
GAGTTCACAGCTGAGCAGTGTTTCGGCGATGGCTTGAACTGGGCTGGTTGCTCCATCATTGTCCTGCTGG
GCCAGCAGCGTCGCTTTGACCTGTTCGACTTCTGTTACCACCTGCTAAAAGTGCAGAGGCAGGACGGGAA
GGATGAAATCATTAAGAATGTGCCCCTGAAGAAGATGGCCGACCGGATCAGGAAGTATCAGATCTTGAAC
AATGAGGTTTTTGCCATCCTGAACAAATACATGAAGTCCGTGGAGACAGACAGTTCCACTGTGGAGCATG
TGCGCTGCTTCCAGCCACCCATCCACCAGTCCTTGGCCACCACTTGCTAAGCAGAAGATCCTGCAGACCC
TTATCTGGAGGAGGAAGAGAAGCAGGAGAGAGAAAGCCACAGCCAGCCTGCCATAGGATCCAACTGGACA
ACGTGTGGGATGGACCTGGAAACAAGCACCTCCCCAAACACATCACCACTCCCTAGGGCGGGGCCTGTGC
ATGCTCTCCCATGACATCTC**CATGCTGGTTTCTCCATAGCA**TAAATGAAAAAAAAAAAAAAAAAGTAAAC
AGGGCAGTGTGTGCTTTTTCTTTTCTCCCCCCTCAACTATATTAAGAACTCCTAGTTTCACCCTTTCTCC
ATCCCATCATCCCACCTATCTGTGGTTGCTTCCCAAGACCTCCTCCCAAGATAGACATCTCCTACCCAGT
GCCCTTGTGTGACCCCAGGACTCAAGTCTCAGACTGTGAACAGATGTGGCCATGCCCAGAGACGCCAGCC
TGGCCAGAAGGGCATGCCTCAGCTTACTACTTCATCTCTCCTGGTTCCCTCCCTGCAGTGCCCCGGGTGT
CATCTTCTCCCACTCTGGGTACCAGGGATTCTACCACATAGGCTTCCCAAAGCCCCATTCTAACTCCCCT
CTCTCAGGGAAGCCCTAGAGAGAGGTCCAAAAAGCATTCACAGCTGTATCACACTCTATGCAGGTGGGGT
AGGAGACTGATCAGGCCTGCTGTGGGGAAGCAGTATGTATGAACACAGCCAGAAATGTCATAGTCCAAAC
AGGATGCTTTCAGGCCATCTCAGCTGCTTGATGGTGAGATGGTTCCCTTATTCCTTCAGGAAAGGCTTAG
CATTGGGCCACATAGGGGAAGCAGCTTTGAACAAATCAGTCATAGCACTGCCTATAGCATTAGCCAGTGA
CCAAATTAGGGACAACGTCTTGGCACAGAATTGCTTATCAAGGAACATTTCCACAAGAAAGAAAATATTA
AGGGGTTATTTCCACAGAAGCCCAAAACGTCTTGGAAACACAGAGGTGAGGAGGAGGAATAGTAATTGTC

AATGAGCTTTTAATACCAAGATACACCCCCTGCCCCCAAAGAAGAGTCCTCTTTTAGGGAATCAGAACCT
TCATTGTCCTAGAAGCTGAAAGATTCTTGGAACATTTTAGCTTTTACTCTCAACTTGCTGTTCTCTTTAC
ATTCCTTAAGTTAGACTTTCGGGTGTGGCTTCTCTCCCAGGGGTAACATTTACTTCCATTTTCTAGACTG
AACCAAAAGTCTTCTGCAGAATCTCCCACCGAGTGTGGTAAGAAGGAAGGACAAAAGGCTTTAGGATATA
AATTTCATGTTACAGAGCATGTCATTGTCAAAGGAAATCTGTGGCCCTGAGATTTTAAGAACATAAAATG
TGACATTTGATATTTCTCCAGCCCAGGGAAGTAAGATGGTTAGCAATGGTTGCCTTAATCAAATGGTCCC
ATTTTTAACCCCAAAGGAAGTGCCCACAGCAAGAGGTTTGTGTGATGCACTTATGTCCTCCGGTGAGGAA
AGGGGGCCACATATGAAAGGCCCCTTAGGTCAGATCCTGAGAGTAGCACATTTGAGTGCAGATTCCTGGG
CCCCACCTCAAACCTACTAATTCTGAATCTCTGGGAATAGGGCCAGGAAATCTGCCCTTTCTACAAACTA
CCCAAGTTGTTCTGTTGCACATCAATGTTTGGGAACCACTGCTGTAAGGGAATCATTCTGGTCACCTTGA
GCTTTGAGCTACCACTAAGCCATGAAAGAAAATACATCATACAGGGAAGAGAGAAGGGAGGAGGTTCCAA
GTAGTAACTGGCAGATCCTCCTGTCTGGAGGTACCACCTTCTATTCTGGTTTCTGACTTTTCCTTCTTGA
TGACCATAGATGTGTTCCAGAGGCAAAAGAGACACATTATCCCAGATGGCAGAACATGCTTTCAAAACAT
ATAAAATGTCAAAGTTCCAGATCCTTCTACATCTTTAGTCCTGTCTGAGGATGGTAGCTGGCTCTCTGTA
GCTGATAGATGGCTAGAGTTCCATCCAAATCCTTGACCACGACTTCATGGAGATTTGAATAATCTATTTG
ATGAGATTTCTATTTCAATAACCCACCTCTCTCACCCCACATTCATATCCCTAAATTTGACCCTCTGGGC
CGAGTCACATTACCTTCAGGAGACTTGATCCCAGTAGACTGAGGTCTTCCCTTTCAGCAGAAAGATTTCA
TTTCCCTGGCTTGCCAGTGGCACTGATTTCCGAACACCCAATGAGTTTAATATTCTTTCCTCCTTGGCAT
TACTGCCCCAGCCTCTTTTTATTTTTTTTGTGTGTGTCTAATAACCAGGAAAAAAATAAAGCTTAGGTTT
TAAAAAGTTTTAAAAATAATCTGTTTCAGAAACTGTCAAATGTACCATATTTGTATTAAGAGTTGTTGGG
AATTTTTGTACAATGAATTTACATTTATTTATGGTGACATATTTACGCTTGTGATCAAATAATGATGTTA
AATTCTTAAATCATATTTGCTATGCAGCTGAAGATGATATTTTGATTTGTATTTTGGGGGTACCTGTGTT
GAGTTGATAAACATTTCCATCTTCATTAAAACTGCTTCCAAACTAGTAAAACCAGCAA

**7) CCDC132, coiled-coil domain containing 132**

```
NM_024260       3234 ccttttcctgaaattatcttac-----tctcat---aattattatgtgac   3275 (Mm)
                           .|||||||.||.||.|     |.||||   |||..|||||||||
NM_017667       3236 ------tctgaaatgatgttgccgccttgtcataacaatggttatgtgac   3279 (Hs)

NM_024260       3276 tacaattacacatgttgcaaaagagtgtactataaatacatagttggaag   3325 (Mm)
                     ||||.|||.||||.||.||.||||.|||||.||||.||.|||.||.|||
NM_017667       3280 tacagttatacattttacagaagaatgtaccataagtatataattagaa-   3328 (Hs)
```

The mooring sequence is not conserved

**8) CCDC14, coiled-coil domain containing 14**

```
NM_172824        583 ------gacac-----------agcacagcgttggtcactacaagatcat    615 (Mm)
                           |||||           ||||.|.|  ||||||.|.|||||||||
NM_022757        515 gaaagagacacatcagacctagagcaaaac--tggtcattgcaagatcat    562 (Hs)

NM_172824        616 tatagaatgtactcaccgataatataccaagccctctgtgagcatgtgca    665 (Mm)
                     |||||||||||.|||||.||||||||||||||||||||||||||.|||||
NM_022757        563 tatagaatgtattcacccataatataccaagccctctgtgagcacgtgca    612 (Hs)
```

>gi|169646280|ref|NM_022757.4| Homo sapiens coiled-coil domain containing 14 (CCDC14), mRNA
GACAAGCCGGGAAGCTTGGAGGGGTCTATTGCGTACGCCCAAGGCGCTGGGCCTGGAGCAGGAGAAAGGG
AGTGGAGGCGCGTCGGAGACTATGAAGCGCGGCATTCGGCGGGATCCTTTCCGGAAGCGGAAGCTCGGCG
GGCGGGCCAAGAAGGTCCGGGAGCCCACGGCGGTTAATTCTTTTTACCGTGAGGCTTCACTTCCCTCGGT
CTGGGCTTCTCTGAGGCGGCGAGAGATGGTCAGGTCTGGAGCTCGACCGGGCCAGGTGTTATCTTCAGGA
AGGCACACTGGACCTGCTAAATTAACAAATGGAAAGAAAGCGACCTATTTAAGAAAAATACCACGTTTTA
ATGCAGATTCTGGCTATTCCATCCATTCTGATTCAGAAAGTCAGGCTGAAACTGTACACGGGCTTGATGG
TTGTGCTTCTTTGCTGAGGGACATTTTGAGAAATGAAGATTCAGCATCTTCAGATAATAAGAAACAGATA
CCTAATGAAGCTTCTGCTAGAAGTGAAAGAGACACATCAGACCTAGAGCAAAACTGGTCATTGCAAGATC
ATTATAGAATGTATTCACCCATAATATACCAAGCCCTCTGTGAGCACGTGCAGACTCAGATGTCACTGAT
GAATGACTTGACTTCAAAGAACATCCCTAATGGAATTCCTGCTGTACCATGCCATGCTCCCTCTCATTCT
GAATCTCAGGCAACTCCTCATTCTAGTTATGGCTTATGTACCTCCACCCCAGTCTGGTCACTTCAGCGGC
CACCCTGCCCTCCAAAGGTTCATTCTGAAGTTCAAACTGATGGCAACAGTCAGTTTGCATCACAAGGTAA
AACAGTTTCTGCAACCTGTACTGATGTTCTACGGAATTCATTTAATACCAGTCCTGGAGTTCCATGTAGC
CTGCCCAAAACTGACATATCAGCTATTCCAACATTGCAGCAACTGGGCCTTGTTAATGGAATTCTGCCAC
AACAAGGAATTCATAAGGAAACAGACCTACTAAAATGTATTCAAACATATTTGTCTCTTTTTCGATCTCA
TGGAAAAGAAACGCATCTGGACAGTCAGACACACCGAAGCCCTACTCAGTCACAACCAGCTTTCTTGGCC
ACTAATGAAGAAAAATGTGCCAGAGAGCAAATTAGAGAGGCCACAAGTGAAAGAAAGGATTTAAACATAC
ATGTGCGAGATACAAAAACAGTGAAGGATGTACAGAAGGCAAAAAATGTGAACAAGACAGCTGAAAAAGT
TAGAATTATAAAATATTTGTTGGGAGAGCTCAAGGCCCTGGTAGCAGAACAAGAGGATTCAGAAATTCAG
AGGTTGATTACAGAAATGGAGGCATGTATATCTGTACTTCCAACAGTAAGTGGAAACACAGATATTCAAG
TTGAGATAGCACTGGCCATGCAACCATTAAGAAGTGAGAATGCTCAGTTACGAAGGCAGTTGAGAATTTT
GAACCAGCAACTCAGAGAACAACAGAAAACTCAAAAACCATCTGGTGCTGTGGATTGCAACCTTGAATTG
TTTTCTCTTCAGTCATTGAATATGTCACTGCAAAATCAATTGGAGGAGTCACTAAAGAGCCAGGAATTAC
TGCAGAGTAAAAATGAAGAGCTGTTAAAAGTGATTGAAAATCAGAAAGATGAAAACAAAAAATTTAGTAG
TATATTTAAAGACAAAGATCAAACTATACTTGAAAATAAACAGCAATATGATATTGAGATAACAAGAATA
AAAATTGAATTGGAGGAAGCCCTAGTCAATGTGAAAAGCTCCCAGTTTAAGTTAGAAACTGCTGAAAAGG

AAAACCAGATATTGGGGATAACATTACGTCAGCGTGATGCTGAGGTGACTCGACTAAGAGAATTAACCAG
AACTTTACAGACTAGCATGGCAAAGCTTCTCTCCGATCTTAGTGTGGACAGTGCTCGCTGCAAGCCTGGG
AATAACCTTACCAAATCACTCTTGAACATTCATGATAAACAACTTCAACATGACCCAGCTCCTGCTCACA
CTTCCATAATGAGCTATCTAAATAAGTTAGAAACAAATTACAGTTTTACACATTCAGAGCCACTTTCTAC
AATTAAAAATGAGGAAACCATAGAGCCAGACAAAACCTATGAAAATGTTCTGTCCTCCAGAGGCCCTCAA
AATAGTAACACTAGGGGCATGGAGGAAGCATCTGCACCTGGAATTATTTCTGCCCTTTCAAAACAGGATT
CTGATGAAGGGAGTGAAACTATGGCTTTAATAGAAGATGAGCATAATTTGGATAATACAATTTACATTCC
TTTTGCTAGAAGCACTCCTGAAAAGAAATCACCACTTTCTAAGAGACTATCCCCTCAGCCACAAATAAGA
GCAGCTACAACACAGCTAGTCAGCAACAGCGGACTTGCTGTCTCTGGAAAAGAAAATAAACTGTGTACAC
CTGTAATCTGTTCCTCTTCAACAAAGGAAGCAGAAGATGCACCTGAAAAACTTTCCAGAGCATCTGATAT
GAAGGACACACAGCTCCTCAAGAAAATAAAGGAAGCAATTGGTAAGATCCCTGCTGCCACCAAGGAGCCA
GAGGAACAAACTGCATGTCATGGCCCATCAGGTTGTCTTAGCAACAGCCTTCAAGTGAAAGGCAATACTG
TCTGTGATGGTAGTGTTTTCACTTCTGACTTGATGTCTGACTGGAGCATCTCTTCGTTTTCAACGTTCAC
TTCTCGTGATGAACAAGACTTCAGAAATGGCCTTGCGGCATTAGATGCCAACATAGCTAGACTCCAGAAG
TCTTTAAGGACTGGTCTTCTGGAGAAATGAATTCAGAAGAAAACTCATCAGGTGCTTCTTTTTAAAACTA
GAACTTGGCTATATTGAATGTGTATTTTTCTTTAGTGAAATGATGTTTTATGTTATTATGTGTGAAGTAA
TATATTGTACAAGTAATAAATGTATTGTTGAGATATATTGACACTGAGGAGCTTATAAAAACAAGTCATC
TTAAGTTCACAATTGCTACAAGAAGAAAGTTGTGGATAACTAGGAAATTATTGTAAGTAATGTTTTATTT
CAGTACTTAGCAATTAGAGTTCTTTTATTAAGATGTATCTGCTGGATTAAGGGTACAGGTTGAAATAGTT
CTGTGGCTGTCCTAAGAAATAATGGGAAAAGAATCTCTGGATGTAAGTTTTTCTGTTGAAACTAGAGGGT
TTTTTTTTTCTGTTTACATATACTTTTTTTTAATAGCAATGTGTTTTTATTAAACATGCTGTGTGCCACA
GGCCAGTGTTGTTGGTGAAATATATAAACATTTATTTAAAGAGAAAAGTTACCAGTATCTACACCTCTTA
AAAAACATTGATTGGTCTAAAAAATATATAGATAACATCCTAAGTTAACATATGGCTTCTTAAAACTTGG
GCACTTTTATTTGTTTTTATCCCAAATTCATGTTTTAAGGCCTTTAAAGAATAGTCAGACTGATAAAGAA
GTGCTAACAGATAAGCTATAGTTGGGGAAATTTGTGGGTTTTTTTTAAATAAGAAATGTTTATTTTTGTC
CTTATATTTAAACATGATGGAATTTGTAAATCTTGGCATTGATTGTAATTCTGCCTTTTTGGAAGAATTT
TTTCTCCCAGCATGTTAGCTGAGAATATTCTCTATTTTATAAATAATATGAAGTAGGTTGGTCTCTCTGC
TTCTCTATACCAGGACTTCTTAGCTCAGTATCATCTCCCTT**CATGTAAGCAGCACGTTTTAA**CTCTTAGG
AAGCTGAATGTTGTGTTATCACTAATACTTTGTACAGGTCACCTGCCTACTCTAATTGTCCTTAGTACTT
GGACAGGCTTTATCATTAAAGAGTGTTCTCCTAATCCCAGAACCTCCTCGTGTTTTTTGTTTTTTTGGTG
GTTTTTTTTTTTTTTTTAGGTGTTAGTGCTACAGCGTTTGAAGGAGCCAGCTGTAGTCTCTTTGATATGT
TCAGATTTTCCAGATAGAGATTTAAGTCTAAAAATTTATTAAGTGAATTCTAAACTATTTCACACAAGAG
TCCATTAGCATCTTTAATTTTATTTAGTATTCATGAATTAATTTATTGGCATCTTATTTGGAGGTAATAG
GCCACAACTCTTTAACAAATAAGTTCAGAAATAAAAAATAATAAAAAAGGAAAAAAA

**9) ALG13, asparagine-linked glycosylation 13 homolog**

NM_177130 991 tatttgaagaacaatataaatcaatatatttactata-----aaaatata 1035 (Mm)

```
                      |||||..|.||.||||| |||.||||.|.||.|||.|     |.|||.||
NM_018466        682 tatttacaaaataatat-aattaataaacttcctacatctatagaatgta    730 (Hs)
```

>gi|270309164|ref|NM_018466.4| Homo sapiens asparagine-linked glycosylation 13 homolog (S. cerevisiae) (ALG13), transcript variant 2, mRNA

ACGGATTTCCGCCTCCCTCACAGAAGGCAGTCACTGCAACGTGCGTGGCCTCAGTTGCGTCATATCCGGC
CCTTGCGATCAGGGCTTGAGGAACCCGCGC**CATG**AAGTGCGTGTTTGTTACCGTAGGGACCACCAGCTTT
GACGACCTCATTGCGTGTGTGTCGGCGCCCGACAGTCTGCAAAAAATCGAGAGCCTTGGTTACAACCGAC
TTATCCTGCAAATTGGTAGAGGAACGGTGGTACCTGAACCCTTCAGTACTGAGTCGTTTACTCTGGATGT
TTACAGGTACAAGGATTCCTTGAAAGAAGACATTCAGAAAGCAGATCTTGTTATTAGTCACGCAGGTGCA
GGAAGCTGTTTGGAGACTCTGGAAAAAGGAAAGCCACTCGTAGTGGTTATAAACGAAAAGTTGATGAACA
ATCATCAGCTGGAACTGGCAAAGCAGCTACACAAAGAGGGTCATCTCTTCTATTGTACCTGCAGCACGCT
TCCTGGGCTGTTACAGTCAATGGACTTATCAACACTGAAATGTTATCCTCCTGGCCAGCCAGAAAAATTT
TCTGCATTTTTGGATAAAGTTGTTGGATTACAAAAATAAACACTAAACTCTCAACACTTTAAAAGCAACC
CCCAAATTCAACCTATGTAATGTGATTAAATCAAATTAAATACATAATATATATTTACAAAATAATATAA
TTAATAAACTTCCTACATCTATAGAATGTATAGGAAATTTTATGTTGTGCACATTTGCATTGGGTCCAAA
TCCTAATTGCAGTTTAGTGAAATTACTAGATTTTTTTCTAATCTTCAGTATGTAGTGAGCATTCACAGGT
TTTCTCCTAGAATCTCAAGTGGAATTGGAATCATAAAAATGAATGAGATTTGAAAACTTTCACAAAGATT
ATAAATGTAGTTAGTTTGATACTCCTTAGAATAAAAAAATGAAACAGTAACAACTTTTATGTAAAAAAAT
TTTACATAAGAGTTAGCATCTTTAGCTTTATAATCTAAGGAACCAAATTTAAAAAATGATGGATTATGCT
CTGTGAAATGAAAAGTTCAATCATTGCCTAAAAAAGATGATCAAGTTAGTTGATCTCTTGTACTTGGAAA
AGCTACTTTTTTCCTAAAGAACAGAGTGGAGTGGATATATTCACTTTTTTTTTCTAAAACATACAAAGTG
ATAAAATAATCGTGCCTAATGTCAAAACTTTACTGGTTTGAGAAGAATAATCAAAAAGGACTTATACCAA
TAGGACTTACTATTGTTTTTTAAGGATCTCCTCTCACAGTGGTAATGAAACATTAACTGTAACAGAAATT
TACAATTTATGTGCTTATAAAATTAGTTTTATGAATGAACAATAAACTGATGCATATAATGGCTTAAATA
TATGGTTTTAAAATTGACTTAACTTGAGCAAGATACATTATGAAGTTCTTTCTGATTAGAGTGTGTGGTC
TCAAGACCTTTACAGTAGATTATCCTTAAGTGTCCATAGCTCTCTGGTACAATGTTTAGATATAGTTTAT
CTATCAAAATAGAACATTCACTATAAAGACCTCTCTTCCATTCCAATCATATTATCCCAACATGCTAAAT
GTTCCAAACATTTTATTTGGAATGCATTTTATCATGTTTTTATAGGTTTATTTTTCTGATTGTGTTTTAT
TTAGGCTAATATTAAAAAAATGCTTACTAGTGGTAGAAGGTGGTGACAAAGAAATACTCTGGGTAGCAGG
AGGAAGCCAACTTGGATTAAAATTTTGCTGAGAATAATCTAAAAAGTGAGTAATCTGGTTGAGAATTCAC
AGCACCATAATCATAATTGTATATCAATGTTATGTTTTAGGAAAGGTCCTATTCAAATCCAGGGAGAGAA
TCTTTAGTGTTTTCTGGGCCCTCATAAATACGTTTTTCTCATTTGAGAAAATTGTGACTTTATGTTGGTC
ACTTAGTTTTTATAGTTTATGATGAAGAATTAAAGAGAAACAAATGAAAGGCAAGGTCTACTCAAATATC
AGACAACTAAAAGGCTATACAGCCCCTAAATCCTTTGCTATCTACTTCCCCAGAATAATGCATAACCTAA
ATTCCATTATTTCATTTAGCAGAGGTTTAAGCCGTTGTTGTCAATTGTGTGCAGTAACGCCTCTGACTTC
CCTTTTGTGGAATTTTGTATCGATTTTGGATTTTTAAGTCAAAGGAGACTTTAAAATCATTTTGATTTGG
CACGGATTCTTGAGATTATCTAGTCCAATTCCATCATATTCAGATTTTACCATTTGCTTAAAGTATAGCT

```
TTTGGCAGAACTGCCCCTAGGAGCAGCTCCTTGATTCCCAGTCCCCTGCTCTTTCTACCCTGCATAATAT
ATGTTGTAAGCTTTTTGTTTCTTTATAGTTGTTTGCCTAGTGTTCATTTTAGTTACTCGAATTCCTTGCA
AGGTAGGTTTTGTTTGTTTATTTATGGAAGTTATTTAGTAGGAATTTCACACTTAACTGGGTTTTCCTTT
GGCGTATGACAACCTTACCTAGGACTTAGAAATCTGTGATTTCTGAGTTTTTGGAATTTAGGAAAATGCT
CTGGGTAGGATTGATAGTAGATTGAATAAATTAAGACTTTAAGACAGAATTGTAGACTTTTGGAGTTGGG
AGATAATTTAAAGAATATTTTAAACCACCAGTTTTACAGATGAGGAAACATGCTAAATTAATCACCACAT
TTACATCTTATTAGTCCTTATTAAAGAATGGTAACCAAAAGTCTGTTTGAAATGTTTGCTCTATTTGTTT
GGTTTCATTACATGACAGAGCTGCAGGTTCTTGATATTAATGAGTAAGTGGAATGCAGTTTATTTAGCAG
TGTTCTATTAGAGGTACTGAAATTTTCAGGGATAAGGAAATGAAAGAGAGTTTAGCATTGGGAAATTTGC
ACCATGGATAGATTAAGAGGTAGACAGTTAATAGAACATTTACGGGTTGGAGT
```

**10) PRRC2B, proline-rich coiled-coil 2B**

```
NM_001159634    7814 cttatcaccaaaatca-------atataaggacacaggtgaacacctat-   7855 (Mm)
                     ||.|||...||.|.||       ||..|.|||     |||.||.|.|.|
NM_013318       7893 ctcatcgtgaacaccacttggtgatggaggga-----gtggacccgtgtg   7937 (Hs)
```

The mooring sequence is not conserved

**11) DHX36, DEAH (Asp-Glu-Ala-His) box polypeptide 36**

```
NM_028136       4881 ttgtaaaacaaagtctactctaaaattattatttggtatctgcacccagg   4930 (Mm)

NM_020865       3625 --------------------------------------------------   3625 (Hs)
```

The mooring sequence is not conserved

NM_028136 and NM_020865 do not align

**12) CCNY, cyclin Y**

```
NM_026484       2258 ctatccctctcctgcctagtt-------ccaagaacattctatctagatt   2300 (Mm)
                     .|||||||.|||..|||..||       ||||||||||    |||||| |
NM_145012       1907 gtatccctttccctccttattcctcaccccaagaacat----tctaga-t   1951 (Hs)

NM_026484       2301 aatatgggtgcttgtgccttccagttttcttgcaattggttgttcttggt   2350 (Mm)
```

```
                          .|.|||||||||||||||||||..||||||||||.|||
NM_145012       1952 cacatgggtgcttgtgccttccgattttcttgcatttg------------   1989 (Hs)
```

>gi|190341111|ref|NM_145012.4| Homo sapiens cyclin Y (CCNY), transcript variant 1, mRNA
AGGCCCCGCCGCCGCCGCTGCTGACCCGGCGGCCGGCCGCCGTTCCGCCCCCTCCCGTGGCGGCGAG
CGGGCGGGCCTCCCCACACGCCCCCGCCGCCCGCGCCCCGCGTCCACCCGCGCCCCGCTCCCGGGGACTG
GGAGAACAGGATAGCAGCAGGAGTCGGGGGGCCGCCGAAGATGGGGAACACTACCTCGTGCTGCGTGTCG
TCCAGTCCCAAGCTCCGGAGGAATGCCCACTCCCGGCTGGAGTCCTACCGGCCAGACACGGACCTGAGCC
GCGAGGACACGGGCTGCAACCTGCAGCACATCAGCGACCGGGAGAACATAGACGATTTGAACATGGAATT
CAATCCTTCAGATCATCCTCGGGCCAGCACAATATTCCTCAGTAAATCTCAGACGGACGTGAGAGAAAAA
CGCAAGAGTCTCTTCATTAACCATCATCCTCCAGGACAAATAGCAAGGAAATACAGTTCCTGCTCCACCA
TTTTCCTAGATGATAGCACAGTCAGTCAACCAAACCTCAAGTATACAATTAAATGTGTCGCTCTTGCAAT
ATATTATCACATCAAAAACAGGGACCCAGATGGAAGGATGCTCTTAGATATTTTTGATGAAAATCTTCAC
CCTCTTTCGAAATCCGAAGTGCCACCAGATTATGACAAACACAACCCAGAGCAGAAGCAGATTTACCGGT
TCGTTCGGACACTGTTCAGTGCTGCTCAGCTGACGGCTGAATGTGCCATCGTCACCCTGGTGTACCTTGA
AAGACTTTTAACATACGCAGAGATAGATATCTGTCCGGCCAACTGGAAGCGGATTGTTTTAGGGGCGATC
CTGCTGGCCTCCAAGGTGTGGGATGACCAGGCTGTATGGAATGTGGATTACTGCCAGATCCTGAAAGACA
TCACGGTGGAGGACATGAACGAGCTAGAGCGACAGTTTCTTGAATTGCTGCAGTTCAACATCAATGTTCC
TTCCAGTGTCTATGCCAAGTATTATTTTGATCTTCGTTCTCTGGCAGAAGCGAACAACCTGAGCTTTCCC
TTGGAGCCCCTGAGCAGGGAGAGGGCTCACAAGCTTGAGGCCATCTCTCGCCTCTGCGAGGACAAGTACA
AGGACCTAAGAAGATCCGCGAGGAAGCGCTCAGCCAGTGCAGACAACCTGACTCTGCCCCGGTGGTCCCC
AGCCATCATCTCTTAACTACGGAGGCCCGCCGGAGGCCACACCATCCCTTAGTTTCTCCTTTAGTTTGAG
AAAAGACAGACTTGGGGTGGGTTTGTTTTTGTTTTTTCTTTCCTTTTCTTTTTTTACGCATAGCTCCGTC
AAGCTGCCTGGATGAGCGCCCATGCAGCAAGGCTTGGAGGAAGCGTCAGTGCCCTGGAGATCCCAGCTCG
CTCTCCCCACTGTCAGCAACAGCACTTCCTTCGTGGAGGAAGTGGACTCGAATCCTGGAGGAGGAAATAA
AGGGAAAGGGAAGTCGTGGAGAGGCAGGGAAAATGGTTAAGCAGCCCGGCCCTCTGGAGTCCCCATGGGG
GCGGTAGCTGAAGTTGGCGAGCGCAGCGGTGGATGCAGAGCTGGCTGCACCCAGGGCTGGGCCAGTGTGT
CCTGTAAGACTTCTTGCATTCCTTCTGCTGCTTTTTTGGGATGGGGGTATTTTTGTTCATTTGTTTTTGC
CCTGTTTTGATTTTGGTCCCACAGAGCAGGGGATGTAGTTTGTACCCACCATG**GCGCAGACTTCCAAATA**
AATAGTACTGGTCATTTCTCTCTGCACTATTTCTGCGCGCTGTCTTCTGAGCTTTCTTCCTCACCAGTGG
GCTGTGCTTGTTCCATTTCTGTACACCCTTATTTTATACCGTTTTTCTTCAACAATGGCGAAATTGACTG
TAGTGCTGAACCAAAAGTATCCCTTTCCCTCCTTATTCCTCACCCCAAGAACATTCTAGATCACATGGGT
GCTTGTGCCTTCCGATTTTCTTGCATTTGTTTTTTTCCTGACCTGAAGTTGTTGTTACAAAATCAGTCAG
ACTTTGTGGGCTGAAGGACACGGTGCAGCAGAGGGTGTCCCTGTGAGAGTTCTGCAGAGTGCTGGGCATG
TGCCTGGAACTACCGAGTAGGAGCCATTTCTTTGTACCCCTGCCTAATCCATTCCTCTCCTTCCAAGTCC
ATTGTTGCAAGCAATATTCTTCTCAATTTTTATATGTTTACTTTAAATCAAAGTTAGTCTATTTGTATAA
AATTTTTAAAAAATCTAAAAGAAAAAGAAAAAAAAACAAGGGTGGGTTGGGTATTCCAGTGGGAAGATCAT
TGAAGGGAAAAATGTTACTATTTACTGAGGTATTTTTCACAGAATGATTGAATTAAAAAAAACTCAACTT

GCATTTTCATTGTGGGTGCTTAGAGAAGTTCTACAAAATTCAAACTGTGAAGGTTTATGCTTCATTAATT
ATGACCATACTTTTTCATTTTCTGACGATCCCTGATTTCCTTATGGACTCAATAAGAATCTTTTATATCA
AGTAAAATGAAAGTTGATGATAGAATATCAAATAGTGAATTTGATATGAAATCTAAGAGTGTGAGTCACT
TGCATATGTAATGCTTTGGGTTTTTGCATATTTTCTTAGGTCACAGTGACAGGTTTCTGAGTTGTTTCCC
TTTTCAAGTCTCTGGGAGGCCAGTGGCAGCAGCAGGTCTGTGTTTCACCTTCTTCCCATGTGAGCTTGGA
AAACTTTGGCATCTTAATTAGTCTTGAATCATACACCTGATCCCCAAACATTGCCTGCTCTCAATGAAAC
ATGCTTTGGAAATGGAAGGGACTCAGAAAGTCGAAATCCCAAGAACTCCAAGCCAAAAGTGTTAGGTTTT
AGTGAAATTTGTGTCAGGTATAAGCATACCTGTTTGGGCCAGAATTATTATCACAGCCTTTCTGACCCAA
ACTTTCAAAAGCAAATAAACAGCATATAAAACGTTAATTATCTTTCCATTGTAATTGTGATGATCTACTC
TGTATGGGTACCAAAATCACCTTCTCTCTTCAAGTCAAAGTTGAATTTAGAACTTTGAATACAGGTCAAA
AAATGTAGAAAATATCATTTTATATTCCCTAATCTATATAGCTGAAAAAGGGTTGTATTTTCTTAGCTGA
ATATGAATACCTCTTAAACATAAATTAGTTCCTCTCCAGTGAAGTATCTTATTTTACCAATCCATTTCAG
GAAAGAGGTTCTGCTGCCGTAAAGGCAGAAATTTTATTTTTATCCCTTTTATTTGAATGAGAGATTTGAA
AATCGAATTATGTAAATATTTCAATGCATCTGCTATTATTTTGTGGAGTTTATTAAACTACTTTAAAAAA
ATTGTATAGTCTATTTATTGTATGTATGTACATACAGTCTGATACCTAAAATTTAAAGTGGATTCCATAA
AACCAGACTCAAGTCTTGTTTAGACTACTGAATACTGTTTTAGCCTGTGCCCTGGATTCTACCTCTGTAT
AGGAAAATTTTCCATATTCATTAATTAGTGTTGATCTGTATTAATTGGTTATGTTTCTTGCACTAAGGAT
GTTTAAAACTCCGCTGTAAGTGTAGATTTTAGTTTTACTGGCAGGATGAATCACTTGACACATAGGTAGA
TACTTGAGGTTCATTTTCTTTTCTTTTTCTACATGATGATGAATAGTTATGTTTTCTTCTCTGATTTGTT
CAAAGTTTGCCAAAAGAAAACACAAAACCAGTACAGTACTTACTGATAAATCGTAGTGACTGTAATTTGT
TTGTAAGGCGAATTAATGCTCTACAATTAAAGCTGGATTACTATTGAGGAACACATTGTCTTAGTGTAGT
TTTTGAATGGAAAGTTTTTGTCATTAAGGATGAAGGAAACGACACAATTTGTTACTTTAATTTTATATTT
GATTTAAACAACAAATAAAGTCATTGGGACAATAAAAAAAAAAAAAAA
